# Modular synthesis of chiral 1,2-dihydropyridines via Mannich/Wittig/cycloisomerization sequence that internally reuses waste

**DOI:** 10.1038/s41467-021-22374-y

**Published:** 2021-04-08

**Authors:** Bo-Shuai Mu, Xiao-Yuan Cui, Xing-Ping Zeng, Jin-Sheng Yu, Jian Zhou

**Affiliations:** 1 Shanghai Engineering Research Center of Molecular Therapeutics and New Drug Development, Shanghai Key Laboratory of Green Chemistry and Chemical Processes, East China Normal University, Shanghai, 200062 China; 2 Key Laboratory of Tropical Medicinal Resource Chemistry of Ministry of Education, Hainan Normal University, Haikou, 571158 China; 3 State Key Laboratory of Organometallic Chemistry, Shanghai Institute of Organic Chemistry, CAS, Shanghai, 200032 China

**Keywords:** Homogeneous catalysis, Synthetic chemistry methodology

## Abstract

1,2-Dihydropyridines are valuable and reactive synthons, and particularly useful precursors to synthesize piperidines and pyridines that are among the most common structural components of pharmaceuticals. However, the catalytic enantioselective synthesis of structurally diverse 1,2-dihydropyridines is limited to enantioselective addition of nucleophiles to activated pyridines. Here, we report a modular organocatalytic Mannich/Wittig/cycloisomerization sequence as a flexible strategy to access chiral 1,2-dihydropyridines from *N*-Boc aldimines, aldehydes, and phosphoranes, using a chiral amine catalyst. The key step in this protocol, cycloisomerization of chiral *N*-Boc δ-amino α,β-unsaturated ketones recycles the waste to improve the yield. Specifically, recycling by-product water from imine formation to gradually release the true catalyst HCl via hydrolysis of SiCl_4_, whilst maintaining a low concentration of HCl to suppress side reactions, and reusing waste Ph_3_PO from the Wittig step to modulate the acidity of HCl. This approach allows facile access to enantioenriched 2-substituted, 2,3- or 2,6-*cis*-disubstituted, and 2,3,6-*cis*-trisubstituted piperidines.

## Abstract (web summary)

1,2-Dihydropyridines are valuable precursors for the synthesis of biologically relevant piperidines and pyridines, but the methods for their synthesis are underdeveloped. Here, the authors report a modular organocatalytic Mannich/Wittig/cycloisomerization sequence to access chiral 1,2-dihydropyridines from *N*-Boc aldimines, aldehydes and phosphoranes, using a chiral amine catalyst.

## Introduction

**Fig. 1:**
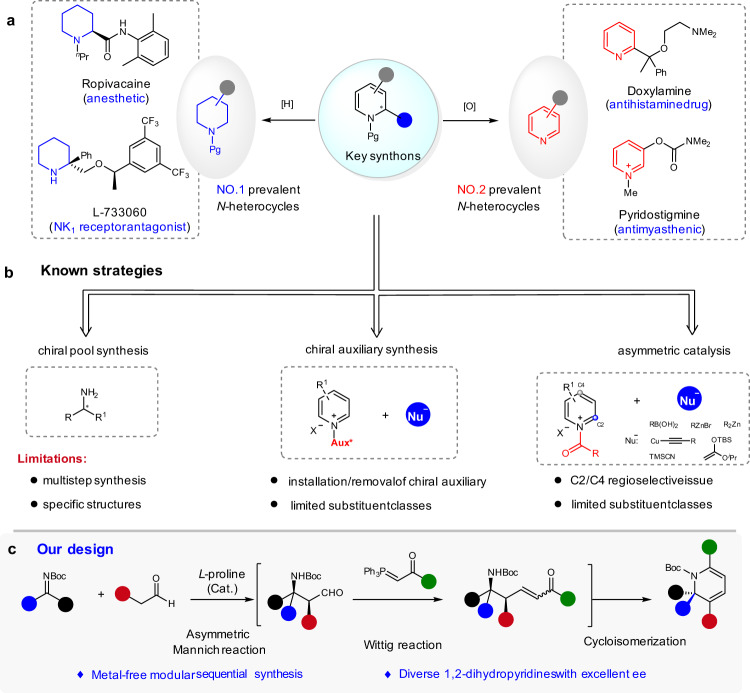
Reaction development. **a** Selected drugs featuring a piperidine or pyridine core. **b** Known strategies to chiral 1,2-dihydropyridines. **c** Organocatalytic Mannich/Wittig/cycloisomerization sequence to chiral 1,2-dihydropyridines.

Piperidines and pyridines are among the most important azacycles present in pharmaceutically active compounds, drugs, and agrochemical targets (Fig. 1a)^1–3^. They constitute the top two prevalent structural motifs in small-molecule drugs that contain *N*-heterocycles, as revealed by a recent analysis of FDA approved drugs^4^. The facile and economical synthesis of structurally diverse piperidines and pyridines is important for future advances in chemistry, medicine, and biology. Despite intensive studies and significant achievements^5–7^, the predictable and modular assembly of substituted piperidines in high diastereomeric excess (de) and enantiomeric excess (ee) from readily available starting materials, using inexpensive chiral catalysts, remains a challenge; the tailor-made metal-free synthesis of substituted pyridines with high chemo- and regioselectivity are also limited^5^. In this context, the 1,2-dihydropyridines are common precursors for the synthesis of piperidines or pyridines, via reduction or oxidation, respectively. Furthermore, they are versatile building blocks for the synthesis of complex molecules^8–10^ and natural products^11,12^.

Nevertheless, the efficient and economical synthesis of optically active 1,2-dihydropyridines with high structural diversity remains undeveloped^13,14^. Several enantioselective methods have been developed, on the basis of three major approaches (Fig. 1b). Traditional strategies using stoichiometric amounts of chiral sources, either in the step-wise synthesis from chiral amine derivatives or diastereoselective functionalization of pyridines with chiral auxiliaries, usually require tedious multistep reaction processes^15–17^. Since the pioneering work of Shibasaki et al.^18^, the enantioselective addition of nucleophiles to activated pyridines, such as pyridinium salts, has emerged as an attractive method^12,18–24^. However, this strategy requires control of the regioselectivity of the attack at C2/C4 position^13^ and it suffers from limited substitution patterns.

Here, we report an organocatalytic strategy for the modular synthesis of chiral 1,2-dihydropyridines from readily available starting materials, namely, a Mannich/Wittig/cycloisomerization sequence (Fig. 1c)^25^. This approach takes advantage of the proline-catalyzed Mannich reaction of *N*-Boc imines and aldehydes, established by List and coworkers^26–28^, to induce a very high level of asymmetry. Advantages expected from this strategy include the use of inexpensive chiral catalysts, achieving high structural diversity by varying the substituents of each substrate, no contamination from transition metals, and the use of an easily removable Boc protecting group.

## Results

### Proof-of-principle study and optimization of the reaction conditions

**Fig. 2:**
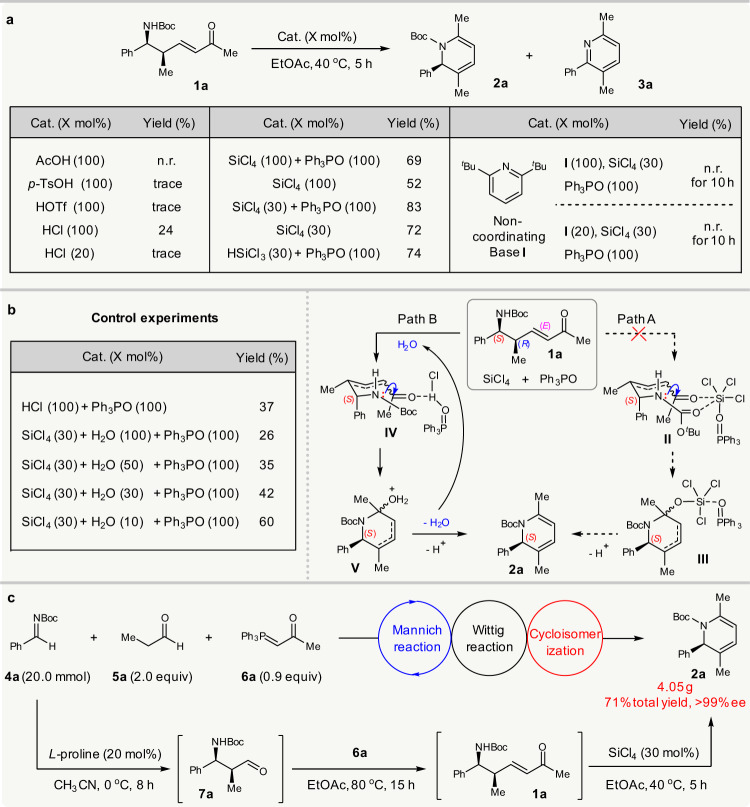
Proof-of-principle study. **a** Optimization of conditions for the cycloisomerization of **1a**. **b** Control experiments and possible mechanism. **c** Gram-scale asymmetric Mannich/Wittig/cycloisomerization sequence. Boc: *tert*-butyloxycarbonyl. EtOAc: ethyl acetate.

The success of this strategy relies on exploiting a cycloisomerization of chiral δ-amino α,β-unsaturated ketones **1** to obtain 1,2-dihydropyridines. The condensation of amines with ketones usually takes place under acid catalysis^29^. However, with a bulky acid-sensitive *N*-Boc protecting group, the cycloisomerization of **1** deemed to be not easy. There appears to be only one earlier report on a similar transformation; Donohoe et al. utilized a stoichiometric amount of trifluoroacetic acid (TFA) to promote the cycloisomerization of racemic *N*-tosyl δ-amino enones without a γ-substituent, at 80 °C^30^. In our study, the use of 100 mol% TFA to mediate the cycloisomerization of **1a** at 80 or 40 °C for 10 h resulted in ca. 7% NMR yield of target **2a**, with 63% or 37% conversion of **1a** and 33% or 14% NMR yield of pyridine by-product **3a** (see Supplementary Table 1 for details) due to the deprotection of the *N*-Boc group (Fig. 2a).

Further screening of typical Brønsted acids revealed that only HCl could promote the reaction to give the desired **2a** in 24% isolated yield, along with **3a** in 46% NMR yield. The generation of **3a** should be achieved due to *N*-Boc deprotection, but almost no reaction occurred when using 20 mol% HCl. To suppress the side deprotection, it was therefore necessary to identify a milder acid catalyst. Despite several efforts, including the use of more than 20 metal salts, no mediation of this cycloisomerization was achieved (see Supplementary Tables 1 and 2 for details of optimization). Then, inspired by the advances in Lewis base activation of SiCl_4_ for carbonyl transformations^31^, we tried the use of SiCl_4_ to mediate the reaction, since our designed tandem sequence will stoichiometrically produce Ph_3_PO. Pleasingly, the merger of SiCl_4_ and Ph_3_PO (each 100 mol%) mediated the reaction to afford **2a** in 69% yield, which was further improved to 83% if reducing the SiCl_4_ to 30 mol%. The presence of Ph_3_PO benefitted the yield, since the use of 100 mol% or 30 mol% SiCl_4_ alone resulted in lower yields of **2a**. The merger of HSiCl_3_ with Ph_3_PO also led to the formation of chiral **2a** in 74% yield, and no conjugate reduction of enone **1a** occurred^32^.

The high yield achieved from the combination of SiCl_4_ and Ph_3_PO in this cycloisomerization prompted us to then study the reaction mechanism. Based on available knowledge, two possible roles of SiCl_4_ could be rationalized, despite it waits for further studies when and how the alkene isomerization proceeds (Fig. 2b). One is that it serves as a Lewis acid catalyst, activated by Lewis base Ph_3_PO, to promote the reaction (path A), and the other is that it acts as a hidden Brønsted acid^33–35^, releasing HCl as the true catalyst via hydrolysis (path B), since HCl could mediate the reaction as well (Fig. 2b). However, it was found that the addition of either 0.2 or 1.0 equiv of noncoordinating base 2,6-di-*tert*-butylpyridine terminated the reaction, and ^29^Si NMR analysis showed almost no interaction between it and SiCl_4_ (see Supplementary Fig. 1 for details). These findings implied that the HCl generated via the hydrolysis of SiCl_4_ was the true catalyst. Furthermore, it was found that the addition of 10, 30, 50, and 100 mol% H_2_O to the reaction mediated by 30 mol% SiCl_4_ and 1.0 equiv Ph_3_PO led to a gradually decreased yield of **2a**. Similarly, using 100 mol% HCl as the catalyst led to only 37% yield in the presence of Ph_3_PO. This implied that the more HCl present at the initial stage of the reaction, the lower the yield of **2a** due to more side deprotection. These findings agreed with the concept of hidden Brønsted acid catalysis, as the gradual generation of HCl from SiCl_4_ inhibited side deprotection to improve the yield. In this cycloisomerization, the hydrolysis of SiCl_4_ by the trace H_2_O in the reaction system produced HCl to initiate the reaction, and the subsequent dehydration generated H_2_O that was internally used to hydrolyze SiCl_4_ (Fig. 2b). Such reuse of the by-product H_2_O for the gradual generation of HCl as the reaction proceeded meant that the concentration of HCl was kept at a low level, which effectively suppressed side reactions. The role of Ph_3_PO in improving the yield was possibly due to its acid–base interaction with HCl, which modulated the acidity of HCl, as confirmed by ^31^P NMR analysis (see Supplementary Figs. 2 and 3 for details).

Having established the conditions for the key cycloisomerization step, we next integrated it with the proline-catalyzed Mannich/Wittig reaction into a tandem sequence. The procedure finally proved to be convenient. The Mannich reaction of aldimine **4a** and propanal **5a** mediated by 20 mol% *L*-proline was performed in MeCN. On completion of this reaction, the crude solid adduct **7a** was easily collected and subjected to the reaction with phosphorane **6a** in EtOAc at 80 °C. Once the synthesis of *N*-Boc δ-amino enone **1a** was completed, 30 mol% SiCl_4_ was directly added to facilitate the cycloisomerization, at 40 °C. Remarkably, this sequence could be run at a 20 mmol scale, giving 1,2-dihydropyridine **2a** (4.05 g) in 71% overall yield and >99% ee (Fig. 2c).

Notably, this sequence constitutes a rare example of an asymmetric tandem reaction that internally recycles waste to facilitate the downstream step^36–47^: by-product water from the imine formation step was reused to produce the true catalyst HCl and waste Ph_3_PO from the Wittig step was recycled as a modulator. In particular, the gradual reuse of byproduct with the proceeding of the reaction, to generate the true catalyst in situ at a low concentration to suppress side reactions, is unknown. This suggests such sustainable sequential reactions are worthwhile to explore.

### Scope of asymmetric Mannich/Wittig/cycloisomerization sequence

**Fig. 3:**
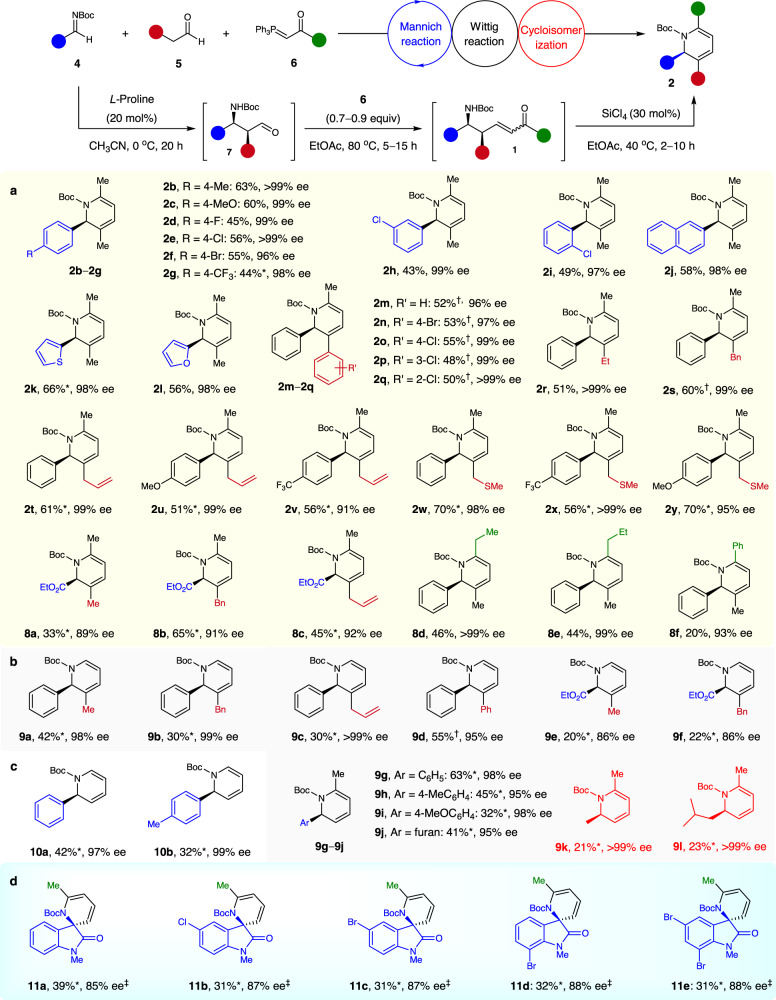
Scope of asymmetric Mannich/Wittig/cycloisomerization sequence. Reaction conditions. Mannich step: imines **4** (0.5 mmol), aldehydes **5** (1.0 mmol), *L*-proline (20 mol%), CH_3_CN (10 mL), 0 °C, 20 h. Wittig step: ylides **6** (1.0 equiv based on the crude **7**), EtOAc (2 mL), 80 °C, 15 h. Cycloisomerization step: SiCl_4_ (30 mol%), EtOAc (2 mL), 40 °C, 2–10 h. Unless otherwise noted, overall yields for 3 steps are given. Ee values were determined by chiral HPLC analysis. *These reactions begin from the corresponding isolated chiral β-amino aldehydes **7** and the yields for two steps are given. †Using 1.0 equiv of **5**. ‡Using SiCl_4_ (45 mol%) instead of SiCl_4_ (30 mol%). For details, see the Supplementary Information. **a** Synthesis of 2,3,6-trisubstituted 1,2-dihydropyridines. **b** Synthesis of 2,3- and 2,6-disubstituted 1,2-dihydropyridines. **c** Synthesis of 2-substituted 1,2-dihydropyridines. **d** Typical examples with isatin-derived *N*-Boc ketimines.

Next, the generality of the synthesis of 2,3,6-trisubstituted chiral 1,2-dihydropyridines with respect to differently substituted imines, aldehydes, and phosphoranes was determined (Fig. 3a). A wide range of aryl *N*-Boc aldimines were compatible with the sequence with propanal **5a** and acetone-derived ylide **6a**. Regardless of the nature and position of the substituent on the phenyl ring, the desired adducts **2b–i** were obtained in 43–63% yields with 96–>99% ee. 2-Naphthyl-, 2-thienyl-, and 2-furanyl-substituted *N*-Boc aldimines also readily afforded the desired products **2j–l** in 56–66% yields with 98% ee. The scope of aldehydes **5** was satisfactory; the targets **2m–y** were obtained in 48–70% yields with 92–>99% ee. This enabled the facile incorporation of diverse substituents on the C3 position of chiral 1,2-dihydropyridines (including aryl, alkyl, allyl, and (methylthio)methyl groups)—offering the hope of further modification. Moreover, α-imino ester was a viable substrate^48^; it afforded 2-ethylcarboxylate-substituted 1,2-dihydropyridines **8a–c** in 33–65% yields with 89–92% ee. Ethyl-, propyl-, and phenyl-substituted phosphoranes **6** were also tolerated under optimized conditions, giving the 2,3,6-trisubstituted dihydropyridines **8d**–**f** in moderate yields with 93–99% ee. This permitted a variation of the C6 substituent of the products.

The modular synthesis of enantioenriched 2,3- and 2,6-disubstituted 1,2-dihydropyridines was also achieved by using acetaldehyde-derived ylide **6** or/and acetaldehyde, as demonstrated by the preparation of **9a–l** with 86–>99% ee (Fig. 3b). As anticipated, the sequence in which use was made of *N*-Boc aldimines, acetaldehyde-derived ylide **6**, and acetaldehyde **5** enabled the smooth preparation of *N*-Boc 2-aryl-substituted 1,2-dihydropyridines **10a**,**b** in 32–42% yields with 97–99% ee (Fig. 3c). Remarkably, the isatin-derived *N*-Boc ketimines have emerged as useful substrates, as well as in the current sequence^49^, affording the valuable chiral dihydropyridine-based spirooxindoles **11a–e** featuring a tetrasubstituted carbon stereocenter with 85–88% ee, albeit in modest yields (Fig. 3d).

### Synthetic utility

**Fig. 4:**
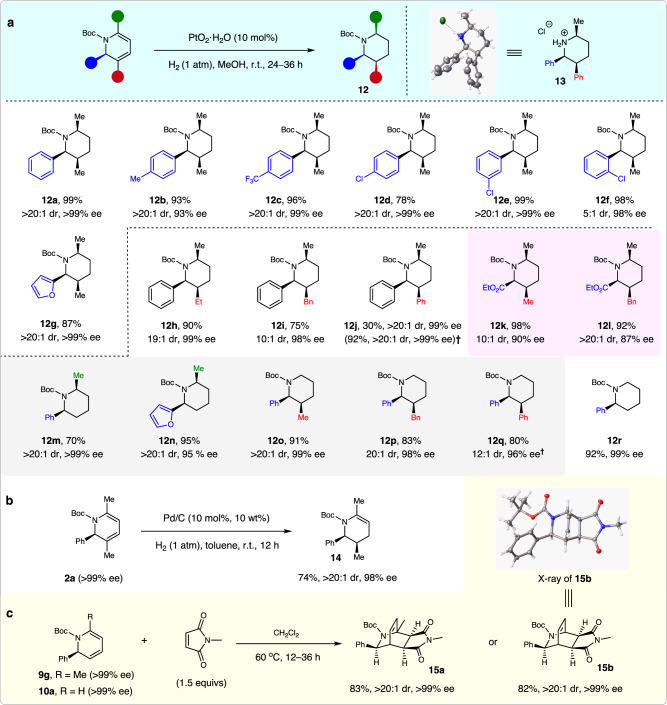
Synthetic utility. **a** Diverse synthesis of *cis*-polysubstituted chiral piperidines **12**. **b** Synthesis of chiral tetrahydropyridine **14**. **c** Diels-Alder reaction to bridged rings **15**. †Using Pd/C instead of PtO_2_·H_2_O.

This sequence was further extended for the synthesis of polysubstituted pyridines, because the addition of MeOH to quench the remaining SiCl_4_ and its hydrolyzed derivatives would release HCl that could be reused for *N*-Boc deprotection^50^. The resulting unprotected 1,2-dihydropyridines could then be easily oxidized to afford pyridines^51^. Interestingly, the tunable synthesis of both pyridines and 3-hydroxyl pyridines was achieved by varying the quenching procedures (See the Supplementary Information for details). More importantly, the synthetic value of this protocol was highlighted by the diverse product transformations. First, the thus obtained structurally diverse enantioenriched 1,2- dihydropyridines could be used for the facile synthesis of piperidines with one, two, or three stereocenters, via hydrogenation, using Pd/C or PtO_2_·H_2_O as the catalyst (Fig. 4a). For example, *N*-Boc 2,3,6-*cis*-trisubstituted piperidines **12a–l** were obtained in 75–99% yields, and with 5:1 to >20:1 dr and 87–>99% ee. The relative and absolute configuration of product **12j** was assigned based on its hydrochloride **13**. These piperidines had substantial structural diversity, because the C2 substituent could be a phenyl, 2-furanyl, or ester group, whereas the C3 substituent could be an aryl or aliphatic group. It should be mentioned that methods to access chiral C3 substituted azacycles are very limited^52^. Furthermore, optically active *cis*-polysubstituted piperidines are generally difficult to access in excellent ee values^53–59^. Given the importance of piperidines in drug discovery^4^, these chiral piperidines should be very useful/valuable in medicinal chemistry research.

The 2,6- and 2,3-*cis*-disubstituted *N*-Boc piperidines could also be readily accessed via the Pt_2_O-catalyzed hydrogenation of the corresponding chiral 1,2-dihydropyridines, as exemplified by the facile access to piperidines **12m–q** in 70–95% yields, and with 12:1–>20:1 dr and 95–>99% ee values. Furthermore, starting from 2-phenyl dihydropyridine **10a**, the corresponding piperidine **12r** was obtained in 92% yield with 99% ee; it is a key intermediate for NK_1_ receptor antagonist synthesis^60,61^. Notably, chiral 1,2-dihydropyridine **2a** could be selectively reduced to enantioenriched tetrahydropyridine **14** in 74% yield with >20:1 dr and 98% ee value (Fig. 4b). In addition, upon treatment with *N*-methylmaleimide, chiral 1,2-dihydropyridines **9g** and **10a** could smoothly deliver the corresponding bridged rings **15a** and **15b** in high yields with excellent dr and ee values (Fig. 4c). The structure and configuration of **15b** was confirmed by its X-ray analysis (see Supplementary Table 17 for details).

## Discussion

The ability to modularly assemble 1,2-dihydropyridines from simple substrates efficiently and enantioselectively, using inexpensive and readily available organocatalysts, should have a substantial impact on the accessibility of piperidine derivatives, particularly the otherwise difficult-to-prepare *cis*-polysubstituted pyridines, for medicinal research. This operationally friendly Mannich/Wittig/cycloisomerization sequence should also lead to its more widespread adoption for the diversity-oriented synthesis of polysubstituted pyridines and 3-hydroxylpyridines. In a broader sense, this research reveals the merit of sustainable tandem reactions that internally reuse waste, and the recycling of gradually produced by-products to generate the true catalyst could keep the concentration of the catalyst at a low level to suppress side reactions. We expect that our findings will inspire the future development of such sustainable sequential reactions for the economical and efficient synthesis of value-added compounds.

## Methods

General procedure for the Mannich/Wittig/cycloisomerization sequence. Under nitrogen atmosphere, to a 50 mL flask were added *N*-Boc aldimines **4** (0.5 mmol, 1.0 equiv), anhydrous acetonitrile (10.0 mL) and *L-*proline (11.5 mg, 0.1 mmol, 20 mol%). The resulting solution was cooled to 0 °C, and the corresponding aldehydes **5** (1.0 mmol, 2.0 equiv) was added. After being stirred at the same temperature for 20 h, water (10 mL) was added to the reaction mixture and acetonitrile was evaporated under vacuo. The resulting suspensions was extracted with EtOAc (10 mL × 3). The combined organic layers were dried over Na_2_SO_4_, filtered, concentrated and dried under vacuo to afford the crude chiral β-amino aldehydes **7** as white solid, which was used directly for the next step. To a 10 mL oven-dried Schlenk tube (with high vacuum valve) was added the above crude aldehydes **7**, phosphorus ylide **6** (1.0 equiv, based on **7**), and anhydrous EtOAc (2.0 mL). After being stirred at 80 °C for 15 h till full conversion of **7** by TLC analysis, SiCl_4_ (18 μL, 0.15 mmol, 30 mol%) was then added in one portion at ambient temperature, and continued to stir at 40 °C for 5–10 h till full conversion of the intermediate **1** by TLC analysis. The reaction mixture was dropwise added to saturated NaHCO_3_ (aq., 10 mL) at 0 °C and extracted with EtOAc (10 mL × 3). The combined organic phases were washed with brine, dried over Na_2_SO_4_, and concentrated under reduced pressure to give the residue, which was purified by flash column chromatography using PE/Et_2_O (20/1, v/v) as the elution to afford the products. Full experimental details and characterization of compounds can be found in the Supplementary Information.

## Supplementary information

Supplementary Information

## Data Availability

X-ray crystallographic data for compound **3e** (CCDC 2018605), **13** (CCDC 2018709) and **15b** (CCDC 2044545) are freely available from the Cambridge Crystallographic Data Centre. Copies of the data can be obtained free of charge via https://www.ccdc.cam.ac.uk/structures/. All other data in support of the findings of this study are available within the Article and its Supplementary Information or from the corresponding author upon reasonable request.
