# Supplementary material for: Modular synthesis of chiral 1,2-dihydropyridines via Mannich/Wittig/cycloisomerization sequence that internally reuses waste
Source: Nat Commun. 2021 Apr 8;12:2219. doi: 10.1038/s41467-021-22374-y (PMC8032725; doi:10.1038/s41467-021-22374-y)
Supplement: Supplementary file 1 — Supplementary Information [file 41467_2021_22374_MOESM1_ESM.pdf]

Supplementary Information

Modular synthesis of chiral 1,2-dihydropyridines via Mannich/Wittig/cycloisomerization sequence that internally reuses waste

Mu et al.

Supplementary Methods

General information

Reactions were monitored by thin layer chromatography using UV light or $KMnO_4$ to visualize the course of reaction. Purification of reaction products was carried out by flash chromatography on silica gel. Chemical yields refer to pure isolated substances. The $[\alpha]_D$ was recorded using PolAAr 3005 High Accuracy Polarimeter. Infrared (IR) spectra were obtained using a Bruker tensor 27 infrared spectrometer. Chiral HPLC analysis was performed on a Shimadzu LC-20AD instrument using Daicel chiral columns at 25 °C and a mixture of HPLC-grade hexanes and isopropanol as eluent. ^{1}H, ^{13}C, ^{19}F NMR, ^{31}P NMR and ^{19}Si NMR spectra were obtained using a Bruker DPX-400 and 500 MHz spectrometer. Chemical shifts were reported in ppm from tetramethylsilane with the solvent resonance as the internal standard. The following abbreviations were used to designate chemical shift multiplicities: s = singlet, d = doublet, t = triplet, q = quartet, m = multiplet.

Unless mentioned, all reactions were performed under N_2 atmosphere. Anhydrous CH_2Cl_2 and CH_3CN were prepared by distillation over P_2O_5 and then from CaH_2. Anhydrous EtOAc was prepared by distillation over activated calcium sulfate and 5Å MS prior use. Anhydrous Et_2O, THF, and toluene were prepared by distillation over sodium-benzophenone ketyl prior to use. Tetrachlorosilane ($SiCl_4$) was purchased from TCI (Shanghai) Development Co., Ltd. Both aldehydes **5** and ylides Ph_3P=CH-C(O)Me and Ph_3P=CH-C(O)Ph were commercially available. *N*-Boc imines **4**[1] and ylides Ph_3P=CH-C(O)R (R = Et, ^{n}Pr)[2] were synthesized according to the corresponding literatures.

Selected condition optimization

Given the success of this cascade relies on exploiting an unprecedented cycloisomerization of chiral δ-amino α,β-unsaturated enones **1** to obtain 1,2-dihydropyrindines, we initiated the study by developing a suitable condition of the cycloisomerization by using *N*-Boc-protected δ-amino enone **1a** as the model substrate.

While the condensation of amines with ketones usually takes place under acid catalysis,[3] the cycloisomerization of **1** deemed to be not easy, due to the presence of a bulky acid-sensitive *N*-Boc group. A literature search directed us to the only one earlier report on a similar transformation; Donohoe et al. utilized a stoichiometric amount of TFA to promote the cycloisomerization of racemic *N*-tosyl δ-amino enones without a γ-substituent, at 80 °C.[4] However, the use of TFA in our study to mediate the cycloisomerization of **1a** at 80 or 40 °C for 10 h resulted in ca. 7% NMR yield of target **2a**, with 63% or 37% conversion of **1a** and 33% or 14% NMR yield of pyridine by-product **3a** due to the deprotection of the *N*-Boc group (entries 1–2). Further screening of typical Brønsted acids, such as HCl, HOTf, *p*-TsOH and AcOH (entries 3–7), revealed that only HCl could promote the reaction to afford the desired **2a** in 24% yield, along with the formation of **3a** in 46% NMR yield (entry 3). Similarly, the reaction could also afford 29% yield of **2a** by using 100 mol% of *in-situ* generated HCl from CH_3COCl and MeOH (entry 4). To suppress the side deprotection, it would be necessary to identify a milder acid catalyst. The use of more than 20 metal salts, including triflates, chlorides, tetrafluoroborates and perchlorates, all failed to mediate this cycloisomerization (entries 8–11).

Then, inspired by the good results achieved with Lewis base activation of $SiCl_4$ for carbonyl transformations,[5] we considered the use of $SiCl_4$ to mediate the reaction, since our designed sequence will stoichiometrically produce Ph_3PO. Pleasingly, the combination of $SiCl_4$ and Ph_3PO (100 mol%, each) mediated the reaction to afford **2a** in 69% yield (entry 12), which was further improved to 83% when the loading of $SiCl_4$ was reduced to 30 mol% (entry 13). However, only 37% yield of **2a** was obtained when reducing the loading of $SiCl_4$ to 10 mol% (entry 14). The presence of Ph_3PO obviously benefitted the yield, because the use of 100 mol% or 30 mol% $SiCl_4$ alone resulted in lower yields of **2a** (entries 15 and 16). Interestingly, the combination of $HSiCl_3$ with Ph_3PO also led to the formation of chiral **2a** in 74% yield (entry 17), and no conjugate reduction of enone **1a** occurred.

Supplementary Table 1. Influence of acids

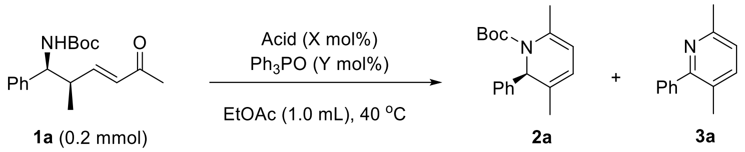

Entry	Acid	X	Y	Time (h)	Conv. of **1a** (%)[a]	Yield of **2a** (%)[b]	Yield of **3a** (%)[a]
1[c]	TFA	100	0	5	63	7[a]	33
2	TFA	100	0	5	37	7[a]	14
3	HCl [d]	100	0	5	>99	24	46
4	CH_3COCl+MeOH	100	0	5	-	29	-
5	HOTf	100	0	5	-	trace	-
6	*p*-TsOH	100	0	5	-	trace	-
7	AcOH	100	0	5	-	n.r.	-
8	$M_x(OTf)_n$ [M = Sc^{III}, Zn^{II}, Mg^{II}, Yb^{III}, Ag^{I}]	10	0	10	-	n.r.	-
9	M_xCl_n (M = In^{III}, Fe^{III}, Cu^{II}, Al^{III})	10	0	10	-	n.r.	-
10	$M_x(BF_4)_n$ [M = K^{I}, Ag^{I}, Zn^{II}, Co^{II}, Li^{I}]	10	0	10	-	n.r.	-
11	$M_x(ClO_4)_n$ [M = Cd^{II}, Mg^{II}, Ba^{II}, Mn^{II}, Ca^{II}]	10	0	10	-	n.r.	-
12	$SiCl_4$	100	100	5	-	69	-
13	$SiCl_4$	30	100	5	-	83	-
14	$SiCl_4$	10	100	10	-	37	-
15	$SiCl_4$	100	0	5	-	52	-
16	$SiCl_4$	30	0	5	-	72	-
17	$HSiCl_3$	30	100	5	-	74	-

[a] Determined by ^{1}H NMR using CH_2Br_2 as internal standard; [b] Isolated yield; [c] In toluene, at 80 °C; [d] HCl (2.0 mol/L EtOAc solution) was used.

Subsequently, the solvent effect of the cycloisomerization was examined in the presence of $SiCl_4$ (30 mol%) and Ph_3PO (100 mol%) at 25 °C, and the use of EtOAc still proved to be the best choice, affording the desired product **2a** in 80% yield (Table S2, entry 5 *vs* entries 1–4). Additionally, the evaluation of reaction temperature (entries 5–8) and concentration (entries 7, 9–10) were conducted, revealing that the optimal reaction temperature and the concentration is 40 °C and 0.2 mol/L (entry 7).

Supplementary Table 2. Influence of solvents and temperature

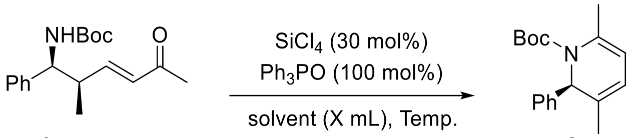

1a (0.2 mmol) **2a**

Entry	Solvent	X (mL)	Temp. (°C)	Time (h)	Yield (%)[a]
1	CH_2Cl_2	1.0	25	5	66
2	Toluene	1.0	25	30	63
3	THF	1.0	25	30	47
4	Acetone	1.0	25	18	36
5	EtOAc	1.0	25	18	80
6	EtOAc	1.0	0	30	56
7	EtOAc	1.0	40	5	83
8	EtOAc	1.0	60	3	68
9	EtOAc	0.5	40	6	69
10	EtOAc	2.0	40	18	73

[a] Isolated yield.

Gram-scale preparation of chiral 1,2-dihydropyridine 2a

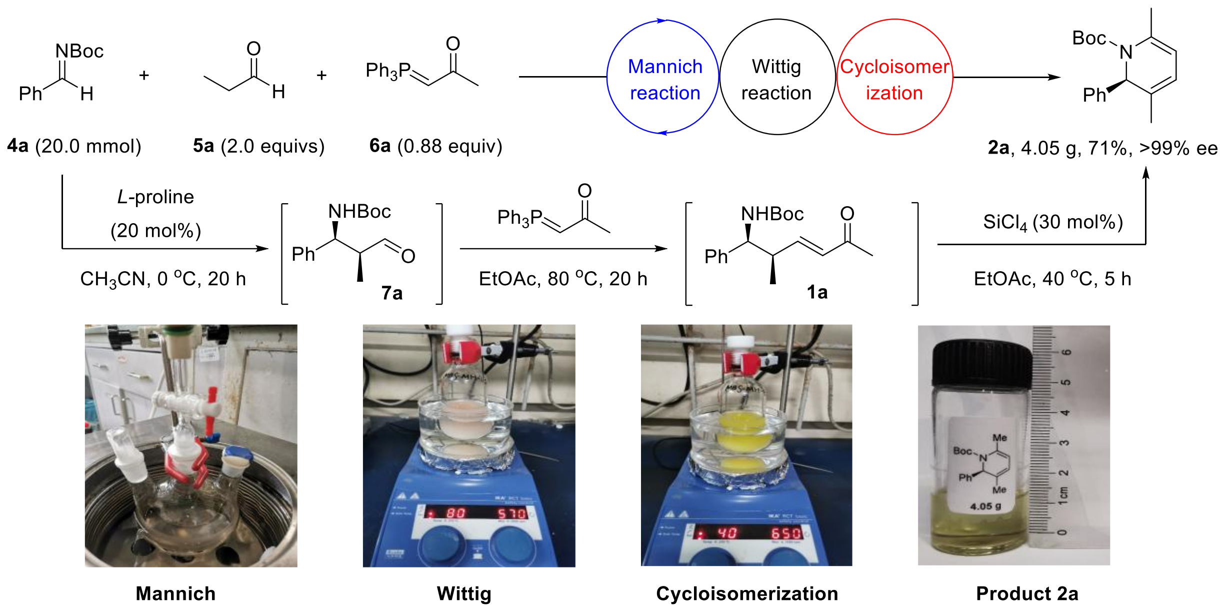

To a 500 mL three-necked flask were added *N*-Boc aldimines **4a** (4.10 g, 20.0 mmol, 1.0 equiv), anhydrous acetonitrile (200 mL) and *L*-proline (460 mg, 4.0 mmol, 20 mol%). The mixture was cooled to 0 °C, and freshly distilled propionaldehyde **5a** (2.32 g, 40.0 mmol, 2.0 equiv) was added. After being stirred at 0 °C for 20 h, water (50 mL) was added to the reaction mixture and acetonitrile was evaporated under vacuo. The resulting suspension was extracted with EtOAc (100 mL × 3). The combined organic layers were dried over Na_2SO_4, filtered, concentrated and dried under vacuo to afford the crude chiral β-amino aldehydes **7a** (4.63 g, 17.6 mmol) as white solid. To a 150 mL thick-walled pressure bottle was added the above crude aldehydes **7a**, phosphorus ylide **6a** [5.60 g, 17.6 mmol (1.0 equiv, based on **7a**)] and anhydrous EtOAc (80 mL). After being stirred at 80 °C for 20 h, $SiCl_4$ (685 μL, 6.0 mmol, 30 mol%) was then added at ambient temperature, and continued to stir at 40 °C for 5 h till full conversion of the resulting intermediate **1a** by TLC analysis. The reaction mixture was poured into saturated $NaHCO_3$ (aq., 250 mL) at 0 °C, and extracted with EtOAc (100 mL × 3). The combined organic phases were washed with brine, dried over Na_2SO_4 and concentrated under reduced pressure to give the residue, which was purified by flash column chromatography using PE/Et_2O (20/1, v/v) as the elution to afford 4.05 g of chiral 1,2-dihydropyridine **2a** as yellowish oil in 71% yield. The absolute configuration of **2a** was determined to be *S* according to the configuration of **7a** reported in the literature.[6] HPLC analysis (Chiralpak AD-H, ^{i}PrOH/hexane = 0.5/99.5, 1.0 mL/min, 205 nm; t_r (minor) = 5.13 min, t_r (major) = 6.55 min) gave the isomeric composition of the product: >99% ee; $[\alpha]_D^{20}$ = -533.5 (*c* = 1.00, $CHCl_3$); ^1H NMR (400 MHz, $CDCl_3$): δ 7.38-7.36 (m,

2H), 7.32-7.26 (m, 3H), 5.85 (d, *J* = 4.8 Hz, 1H), 5.63 (s, 1H), 5.34 (d, *J* = 5.2 Hz, 1H), 1.98 (s, 3H), 1.81 (s, 3H), 1.53 (s, 9H); ^{13}C NMR (100 MHz, $CDCl_3$): δ 153.96, 139.57, 132.16, 131.81, 128.34, 127.68, 127.60, 118.99, 112.09, 81.28, 59.15, 28.49, 21.91, 20.92; IR (ATR): 2974, 1693, 1607, 1454, 1367, 1329, 1255, 1223 cm^{-1}; GC-MS (EI): 285 (M^+, 15), 229 (12), 214 (10), 184 (28), 170 (12), 152 (64), 108 (100), 57 (72); HRMS (EI): Exact mass calcd for $C_{18}H_{23}NO_2$ $[M]^+$: 285.1729, Found: 285.1732.

General procedure for the synthesis of chiral 1,2-dihydropyridines

General procedure for synthesis of 2b–f, 2h–j, 2l–s, 8d–f, 9d

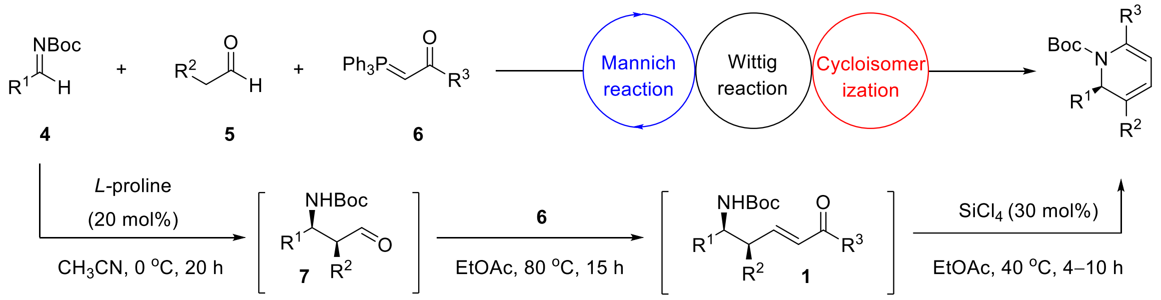

To a 50 mL flask were added *N*-Boc aldimines **4** (0.5 mmol, 1.0 equiv), anhydrous acetonitrile (10.0 mL) and *L*-proline (11.5 mg, 0.1 mmol, 20 mol%). The resulting solution was cooled to 0 °C, and the corresponding aldehydes **5** (1.0 mmol, 2.0 equiv) was added. After being stirred at the same temperature for 20 h, water (10 mL) was added to the reaction mixture and acetonitrile was evaporated under vacuo. The resulting suspensions was extracted with EtOAc (10 mL × 3). The combined organic layers were dried over Na_2SO_4, filtered, concentrated and dried under vacuo to afford the crude chiral β-amino aldehydes **7** as white solid, which was used directly for the next step. To a 10 mL oven-dried Schlenk tube (with high vacuum valve) was added the above crude aldehydes **7**, phosphorus ylide **6** (1.0 equiv, based on **7**) and anhydrous EtOAc (2.0 mL). After being stirred at 80 °C for 15 h till full conversion of **7** by TLC analysis, $SiCl_4$ (18 μL, 0.15 mmol, 30 mol%) was then added in one portion at ambient temperature, and continued to stir at 40 °C for 5-10 h till full conversion of the intermediate **1** by TLC analysis. The reaction mixture was dropwise added to saturated $NaHCO_3$ (aq., 10 mL) at 0 °C and extracted with EtOAc (10 mL × 3). The combined organic phases were washed with brine, dried over Na_2SO_4 and concentrated under reduced pressure to give the residue, which was purified by flash column chromatography using PE/Et_2O (20/1, v/v) as the elution to afford the products. [Note: In the case of **2m**–**q**, **2s**, and **9d**, more convenient work-up procedure and 1.0 equiv of the corresponding aldehydes **5** were used in the first asymmetric Mannich

step. The simplified work-up procedure is as follow: the resulting white precipitate reaction mixture was directly filtered and washed with iced PE to afford the crude aldehydes **7**, which was then used for next step after drying under vacuo]. *The racemic samples are indeed prepared by using racemic proline following the standard procedure.*

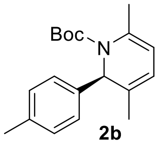

2b

Product **2b** was obtained in 63% yield as yellowish oil. HPLC analysis (Chiralpak AD-H, ^{i}PrOH/hexane = 0.5/99.5, 1.0 mL/min, 205 nm; t_r (minor) = 6.31 min, t_r (major) = 10.26 min) gave the isomeric composition of the product: >99% ee; $[\alpha]_D^{20}$ = -519.4 (*c* = 1.10, $CHCl_3$); ^{1}H NMR (400 MHz, $CDCl_3$): δ 7.26 (d, *J* = 7.6 Hz, 2H), 7.10 (d, *J* = 7.6 Hz, 2H), 5.83 (d, *J* = 4.8 Hz, 1H), 5.59 (s, 1H), 5.34 (d, *J* = 5.2 Hz, 1H), 2.32 (s, 3H), 1.98 (s, 3H), 1.79 (s, 3H), 1.52 (s, 9H); ^{13}C NMR (100 MHz, $CDCl_3$): δ 153.95, 137.32, 136.59, 132.08, 131.98, 129.05, 127.57, 118.83, 112.04, 81.20, 58.94, 28.50, 21.93, 21.21, 20.87; IR (ATR): 2972, 1691, 1509, 1329, 1245, 1159, 1035, 781 cm^{-1}; GC-MS (EI): 299 (M^+, 16), 243 (94), 228 (26), 198 (35), 184 (19), 152 (43), 108 (100), 57 (65); HRMS (EI): Exact mass calcd for $C_{19}H_{25}NO_2$ $[M]^+$: 299.1885, Found: 299.1889.

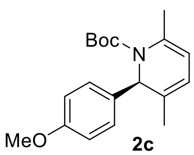

2c

Product **2c** was obtained in 60% yield as yellowish oil. HPLC analysis (Chiralpak AD-H, ^{i}PrOH/hexane = 0.5/99.5, 1.0 mL/min, 205 nm; t_r (minor) = 7.35 min, t_r (major) = 10.51 min) gave the isomeric composition of the product: 99% ee; $[\alpha]_D^{20}$ = -553.6 (*c* = 1.10, $CHCl_3$); ^{1}H NMR (400 MHz, $CDCl_3$): δ 7.30 (d, *J* = 8.4 Hz, 2H), 6.82 (d, *J* = 8.4 Hz, 2H), 5.83 (d, *J* = 4.8 Hz, 1H), 5.58 (s, 1H), 5.34 (d, *J* = 5.2 Hz, 1H), 3.78 (s, 3H), 1.97 (s, 3H), 1.78 (s, 3H), 1.52 (s, 9H); ^{13}C NMR (100 MHz, $CDCl_3$): δ 159.16, 153.94, 132.08, 131.97, 131.63, 128.93, 118.76, 113.66, 111.97, 81.19, 58.64, 55.24, 28.50, 21.92, 20.77; IR (ATR): 2972, 1691, 1607, 1509, 1456, 1366, 1329, 1159 cm^{-1}; GC-MS (EI): 315 (M^+, 14), 259 (100), 244 (50), 214 (36), 200 (32), 152 (14), 108 (99), 57 (66); HRMS (EI): Exact mass calcd for $C_{19}H_{25}NO_3$ $[M]^+$: 315.1834, Found: 315.1838.

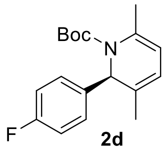

2d

Product **2d** was obtained in 45% yield as yellowish oil. HPLC analysis (Chiralpak AD-H, ^{i}PrOH/hexane = 0.5/99.5, 1.0 mL/min, 205 nm; t_r (minor) = 4.64 min, t_r (major) = 6.58 min) gave the isomeric composition of the product: 99% ee; $[\alpha]_D^{20}$ = -512.9 (*c* = 1.00, $CHCl_3$); ^{1}H NMR (400 MHz, $CDCl_3$): δ 7.36-7.32 (m, 2H), 6.99-6.94 (m, 2H), 5.85 (d, *J* = 4.8 Hz, 1H), 5.60 (s, 1H), 5.33 (d, *J* = 5.2 Hz, 1H), 1.96 (s, 3H), 1.78 (s, 3H), 1.52 (s, 9H); ^{13}C NMR (100 MHz, $CDCl_3$): δ 162.43 (d, *J* = 244), 153.92, 135.20 (d, *J* = 3 Hz), 132.00, 131.62,

129.42 (d, J = 8 Hz), 119.15, 115.16 (d, J = 21 Hz), 111.95, 81.44, 58.39, 28.49, 21.91, 20.76; ^{19}F NMR (376 MHz, $CDCl_3$): δ -115.00 (s, F); IR (ATR): 2972, 1686, 1604, 1506, 1450, 1390, 1367, 1332 cm^{-1}; GC-MS (EI): 303 (M^+, 12), 247 (79), 232 (15), 202 (26), 188 (14), 152 (38), 108 (91), 57 (100); HRMS (EI): Exact mass calcd for $C_{18}H_{22}NO_2F$ $[M]^+$: 303.1635, Found: 303.1637.

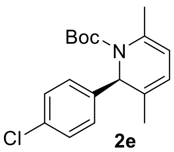

2e

Product **2e** was obtained in 56% yield as yellowish oil. HPLC analysis (Chiralpak AD-H, ^{i}PrOH/hexane = 0.5/99.5, 1.0 mL/min, 205 nm; t_r (minor) = 5.51 min, t_r (major) = 8.24 min) gave the isomeric composition of the product: >99% ee; $[\alpha]_D^{20}$ = -454.1 (c = 1.00, $CHCl_3$); ^{1}H NMR (400 MHz, $CDCl_3$): δ 7.30 (d, J = 8.4 Hz, 2H), 7.25 (d, J = 8.4 Hz, 2H), 5.86 (d, J = 5.2 Hz, 1H), 5.59 (s, 1H), 5.33 (d, J = 5.2 Hz, 1H), 1.96 (s, 3H), 1.78 (s, 3H), 1.52 (s, 9H); ^{13}C NMR (100 MHz, $CDCl_3$): δ 153.90, 137.97, 133.48, 132.05, 131.28, 129.16, 128.53, 119.31, 111.95, 81.54, 58.41, 28.47, 21.93, 20.81; IR (ATR): 2974, 1696, 1608, 1487, 1450, 1390, 1368, 1330 cm^{-1}; GC-MS (EI): 321 $[M(^{37}Cl)^+$, 4], 319 $[M(^{35}Cl)^+$, 8], 263 (59), 248 (12), 218 (22), 152 (44), 108 (98), 57 (100); HRMS (EI): Exact mass calcd for $C_{18}H_{22}NO_2{}^{35}Cl$ $[M]^+$: 319.1339, Found: 319.1336.

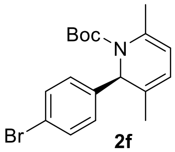

2f

Product **2f** was obtained in 55% yield as yellowish oil. HPLC analysis (Chiralpak AD-H, ^{i}PrOH/hexane = 0.5/99.5, 1.0 mL/min, 205 nm; t_r (minor) = 5.28 min, t_r (major) = 7.58 min) gave the isomeric composition of the product: 96% ee; $[\alpha]_D^{20}$ = -439.7 (c = 1.00, $CHCl_3$); ^{1}H NMR (400 MHz, $CDCl_3$): δ 7.41 (d, J = 8.8 Hz, 2H), 7.25 (d, J = 8.8 Hz, 2H), 5.85 (d, J = 4.4 Hz, 1H), 5.58 (s, 1H), 5.33 (d, J = 5.2 Hz, 1H), 1.96 (s, 3H), 1.78 (s, 3H), 1.52 (s, 9H); ^{13}C NMR (100 MHz, $CDCl_3$): δ 153.88, 138.49, 132.05, 131.48, 131.17, 129.51, 121.70, 119.34, 111.93, 81.53, 58.47, 28.46, 21.91, 20.78; IR (ATR): 2974, 1693, 1607, 1485, 1454, 1367, 1329, 1222 cm^{-1}; GC-MS (EI): 365 $[M(^{81}Br)^+$, 6], 363 $[M(^{79}Br)^+$, 6], 307 (46), 292 (10), 262 (16), 152 (47), 108 (100), 57 (90); HRMS (EI): Exact mass calcd for $C_{18}H_{22}NO_2{}^{79}Br[M]^+$: 363.0834, Found: 363.0828.

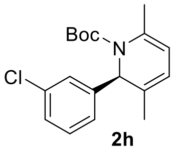

2h

Product **2h** was obtained in 43% yield as yellowish oil. HPLC analysis (Chiralpak AD-H, ^{i}PrOH/hexane = 0.5/99.5, 1.0 mL/min, 205 nm; t_r (minor) = 5.12 min, t_r (major) = 6.67 min) gave the isomeric composition of the product: 99% ee; $[\alpha]_D^{20}$ = -500.3 (c = 1.00, $CHCl_3$); ^{1}H NMR (400 MHz, $CDCl_3$): δ 7.32-7.22 (m, 4H), 5.87 (d, J = 4.8 Hz, 1H), 5.60 (s, 1H), 5.34 (d, J = 5.2 Hz, 1H), 1.98 (s, 3H), 1.80 (s, 3H), 1.53 (s, 9H); ^{13}C NMR (100 MHz, $CDCl_3$): δ 153.89, 141.64, 134.19, 132.20, 130.97, 129.69, 127.93, 127.75, 125.98, 119.52, 112.02, 81.60, 58.66, 28.48, 21.90, 20.86; IR (ATR): 2979, 1685, 1601, 1571, 1454, 1368, 1280, 1155 cm^{-1}; GC-MS (EI):

321 [M(^{37}Cl)$^+$, 2], 319 [M(^{35}Cl)$^+$, 7], 263 (44), 248 (4), 218 (16), 204 (5), 152 (54), 108 (100), 57 (75); HRMS (EI): Exact mass calcd for $C_{18}H_{22}NO_2{}^{35}Cl$ [M]$^+$: 319.1339, Found: 319.1334.

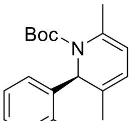

2i

Product **2i** was obtained in 49% yield as yellowish oil. HPLC analysis (Chiralpak AD-H, ^{i}PrOH/hexane = 0.5/99.5, 1.0 mL/min, 205 nm; t_r (minor) = 9.60 min, t_r (major) = 11.38 min) gave the isomeric composition of the product: 97% ee; $[\alpha]_D^{20}$= -492.0 (*c* = 1.04, $CHCl_3$); ^{1}H NMR (400 MHz, $CDCl_3$): δ 7.52-7.49 (m, 1H), 7.35-7.33 (m, 1H), 7.21-7.14 (m, 2H), 6.08 (s, 1H), 5.73 (d, *J* = 5.2 Hz, 1H), 5.37 (d, *J* = 5.2 Hz, 1H), 2.09 (s, 3H), 1.77 (s, 3H), 1.48 (s, 9H); ^{13}C NMR (100 MHz, $CDCl_3$): δ 153.79, 138.57, 133.51, 133.30, 132.37, 129.53, 128.98, 128.81, 127.33, 118.27, 112.04, 81.37, 56.75, 28.47, 22.10, 20.39; IR (ATR): 2927, 1689, 1610, 1474, 1393, 1368, 1325, 1288 cm^{-1}; GC-MS (EI): 321 [M(^{37}Cl)$^+$, 2], 319 [M(^{35}Cl)$^+$, 7], 263 (41), 246 (4), 218 (17), 204 (7), 152 (53), 108 (100), 57 (59); HRMS (EI): Exact mass calcd for $C_{18}H_{22}NO_2{}^{35}Cl$ [M]$^+$: 319.1339, Found: 319.1344.

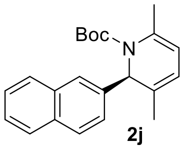

2j

Product **2j** was obtained in 58% yield as yellowish oil. HPLC analysis (Chiralpak AD-H, ^{i}PrOH/hexane = 0.5/99.5, 1.0 mL/min, 205 nm; t_r (minor) = 6.59 min, t_r (major) = 9.27 min) gave the isomeric composition of the product: 98% ee; $[\alpha]_D^{20}$ = -544.7 (*c* = 1.00, $CHCl_3$); ^{1}H NMR (400 MHz, $CDCl_3$): δ 7.82-7.77 (m, 3H), 7.73 (s, 1H), 7.58 (dd, *J* = 8.4, 1.2 Hz, 1H), 7.46-7.44 (m, 2H), 5.92 (d, *J* = 4.8 Hz, 1H), 5.80 (s, 1H), 5.38 (d, *J* = 5.2 Hz, 1H), 1.96 (s, 3H), 1.85 (s, 3H), 1.54 (s, 9H); ^{13}C NMR (100 MHz, $CDCl_3$): δ 154.06, 136.79, 133.24, 133.14, 132.18, 131.74, 128.26, 127.61, 126.33, 126.21, 125.92, 125.88, 119.30, 112.22, 81.39, 59.30, 28.53, 21.91, 20.96; IR (ATR): 2970, 1691, 1606, 1453, 1366, 1327, 1221, 1159 cm^{-1}; GC-MS (EI): 335 (M$^+$, 12), 279 (77), 264 (20), 232 (37), 220 (16), 207 (30), 152 (27), 108 (100), 57 (48); HRMS (EI): Exact mass calcd for $C_{22}H_{25}NO_2$ [M]$^+$: 335.1885, Found: 335.1887.

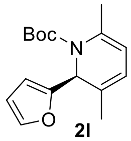

2l

Product **2l** was obtained in 56% yield as yellowish oil. HPLC analysis (Chiralpak AD-H, ^{i}PrOH/hexane = 0.5/99.5, 1.0 mL/min, 205 nm; t_r (minor) = 5.66 min, t_r (major) = 8.04 min) gave the isomeric composition of the product: 98% ee; $[\alpha]_D^{20}$ = -413.9 (c = 1.10, $CHCl_3$); ^{1}H NMR (400 MHz, $CDCl_3$): δ 7.36 (d, *J* = 0.8 Hz 1H), 6.27-6.26 (m, 1H), 6.11 (d, *J* = 3.2 Hz, 1H), 5.76 (d, *J* = 4.8 Hz, 1H), 5.73 (s, 1H), 5.34 (d, *J* = 5.2 Hz, 1H), 2.04 (s, 3H), 1.86 (s, 3H), 1.53 (s, 9H); ^{13}C NMR (100 MHz, $CDCl_3$): δ 153.61, 152.65, 142.51, 132.13, 129.77, 119.40, 111.65, 110.00, 107.32, 81.52, 53.73, 28.46, 21.69, 20.57; IR (ATR): 2926, 2111, 1719, 1599, 1569, 1442,

1379, 1326 cm^{-1}; GC-MS (EI): 275 (M$^+$, 17), 219 (85), 204 (39), 174 (62), 160 (62), 146 (10), 108 (22), 57 (100); HRMS (EI): Exact mass calcd for $C_{16}H_{21}NO_3$ [M]$^+$: 275.1521, Found: 275.1525.

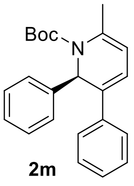

2m

Product **2m** was obtained in 52% yield as yellowish oil. HPLC analysis (Chiralpak AY-H, ^{i}PrOH/hexane = 0.5/99.5, 1.0 mL/min, 205 nm; t_r (minor) = 6.86 min, t_r (major) = 8.36 min) gave the isomeric composition of the product: 96% ee; $[\alpha]_D^{20}$ = -217.9 (*c* = 0.28, $CHCl_3$); ^{1}H NMR (400 MHz, $CDCl_3$): δ 7.44-7.42 (m, 2H), 7.38-7.37 (m, 2H), 7.31-7.18 (m, 6H), 6.60 (d, *J* = 5.6 Hz, 1H), 6.48 (s, 1H), 5.57 (d, *J* = 5.6 Hz, 1H), 2.05 (s, 3H), 1.56 (s, 9H); ^{13}C NMR (100 MHz, $CDCl_3$): δ 153.79, 139.71, 138.31, 134.87, 131.93, 128.73, 128.24, 127.61, 127.52, 127.25, 125.16, 119.88, 113.23, 81.76, 55.96, 28.50, 21.97; IR (ATR): 2975, 1694, 1367, 1335, 1221, 1160, 1107, 694 cm^{-1}; GC-MS (EI): 347 (M$^+$, 10), 291 (76), 244 (60), 214 (47), 202 (10), 170 (100), 128 (8), 57 (51); HRMS (EI): Exact mass calcd for $C_{23}H_{25}NO_2$ [M]$^+$: 347.1885, Found: 347.1883.

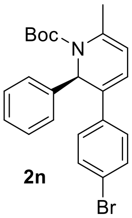

2n

Product **2n** was obtained in 53% yield as yellowish oil. HPLC analysis (Chiralcel OD-H, ^{i}PrOH/hexane = 0.5/99.5, 1.0 mL/min, 230 nm; t_r (minor) = 6.12 min, t_r (major) = 6.93 min) gave the isomeric composition of the product: 97% ee; $[\alpha]_D^{20}$ = -145.0 (*c* = 1.58, $CHCl_3$); ^{1}H NMR (400 MHz, $CDCl_3$): δ 7.41-7.39 (m, 2H), 7.35-7.33 (m, 2H), 7.29-7.24 (m, 5H), 6.59 (d, *J* = 5.2 Hz, 1H), 6.42 (s, 1H), 5.56-5.55 (m, 1H), 2.05 (s, 3H), 1.55 (s, 9H); ^{13}C NMR (100 MHz, $CDCl_3$): δ 153.73, 139.69, 139.32, 138.29, 137.19, 135.37, 131.81, 130.70, 128.71, 128.33, 128.23, 127.78, 127.60, 127.51, 127.43, 127.24, 126.68, 125.15, 121.04, 120.35, 119.87, 113.22, 113.01, 81.94, 81.74, 55.80, 29.78, 28.48, 22.77, 22.04; IR (ATR): 2972, 1696, 1492, 1452, 1328, 1160, 1109, 764 cm^{-1}; HRMS (ESI): Exact mass calcd for $C_{23}H_{24}{}^{79}BrNNaO_2$ [M+Na]$^+$: 448.0883; found: 448.0883. (The distinct rotameric isomers were observed in the NMR spectra, due to the presence of sterically bulky Boc group in the product).

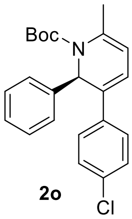

2o

Product **2o** was obtained in 55% yield as yellowish oil. HPLC analysis (Chiralpak AS-H, ^{i}PrOH/hexane = 0.5/99.5, 1.0 mL/min, 230 nm; t_r (minor) = 4.99 min, t_r (major) = 5.94 min) gave the isomeric composition of the product: 99% ee; $[\alpha]_D^{20}$ = -211.8 (*c* = 1.24, $CHCl_3$); ^{1}H NMR (400 MHz, $CDCl_3$): δ 7.35-7.33 (m, 4H), 7.29-7.24 (m, 5H), 6.58 (d, *J* = 5.6 Hz, 1H), 6.43 (s, 1H), 5.57-5.55 (m, 1H), 2.05 (s, 3H), 1.56 (s, 9H); ^{13}C NMR (100 MHz, $CDCl_3$): δ 153.76, 139.35, 136.78, 135.31, 132.94, 130.72, 128.88, 128.34, 127.78, 127.45, 126.38, 120.30, 113.03, 81.94, 55.88, 28.49, 22.03; IR (ATR): 2974, 1695, 1574, 1452, 1328, 1160,

1109, 734 cm^{-1}; GC-MS (EI): 383 [M(^{37}Cl)$^{+}$, 4], 381 [M(^{35}Cl)$^{+}$, 11], 325 (78), 281 (32), 278 (97), 248 (46), 204 (100), 121 (9), 57 (83); HRMS (EI): Exact mass calcd for $C_{23}H_{24}NO_2{}^{35}Cl$ [M]$^{+}$: 381.1496, Found: 381.1495.

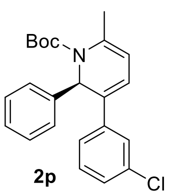

2p

Product **2p** was obtained in 48% yield as yellowish oil. HPLC analysis (Chiralpak AY-H, ^{i}PrOH/hexane = 0.5/99.5, 1.0 mL/min, 230 nm; t_r (minor) = 6.21 min, t_r (major) = 6.80 min) gave the isomeric composition of the product: 99% ee; $[\alpha]_D^{20}$ = -227.1 (*c* = 1.28, $CHCl_3$); ^{1}H NMR (400 MHz, $CDCl_3$): δ 7.44-7.42 (m, 1H), 7.36-7.34 (m, 2H), 7.29-7.15 (m, 6H), 6.60 (d, *J* = 5.2 Hz, 1H), 6.44 (s, 1H), 5.57-5.56 (m, 1H), 2.06 (s, 3H), 1.56 (s, 9H); ^{13}C NMR (100 MHz, $CDCl_3$): δ 153.73, 140.21, 139.70, 139.27, 138.30, 135.72, 134.78, 130.52, 129.94, 128.72, 128.34, 128.23, 127.79, 127.61, 127.51, 127.42, 127.25, 127.18, 125.22, 125.15, 123.27, 121.02, 119.88, 113.22, 112.95, 81.99, 81.75, 55.79, 28.49, 22.06; IR (ATR): 2974, 1695, 1574, 1452, 1328, 1160, 1109, 734 cm^{-1}; GC-MS (EI): 383 [M(^{37}Cl)$^{+}$, 2], 381 [M(^{35}Cl)$^{+}$, 6], 325 (42), 280 (55), 278 (100), 248 (26), 204 (68), 121 (12), 57 (58); HRMS (EI): Exact mass calcd for $C_{23}H_{24}NO_2{}^{35}Cl$ [M]$^{+}$: 381.1496, Found: 381.1493. (The distinct rotameric isomers were observed in the NMR spectra, due to the presence of sterically bulky Boc group in the product).

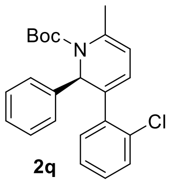

2q

Product **2q** was obtained in 50% yield as yellowish oil. HPLC analysis (Chiralpak AY-H, ^{i}PrOH/hexane = 3/97, 0.5 mL/min, 230 nm; t_r (minor) = 8.43 min, t_r (major) = 10.56 min) gave the isomeric composition of the product: >99% ee; $[\alpha]_D^{20}$ = -317.2 (*c* = 1.11, $CHCl_3$); ^{1}H NMR (400 MHz, $CDCl_3$): δ 7.38-7.34 (m, 3H), 7.28-7.20 (m, 4H), 7.18-7.14 (m, 2H), 6.43 (s, 1H), 6.27 (d, *J* = 5.2 Hz, 1H), 5.56-5.54 (m, 1H), 2.12 (s, 3H), 1.57 (s, 9H); ^{13}C NMR (100 MHz, $CDCl_3$): δ 153.39, 139.56, 138.83, 135.41, 132.55, 131.53, 131.05, 130.19, 128.72, 128.52, 128.37, 128.21, 127.57, 127.33, 126.89, 125.15, 124.51, 112.14, 81.65, 57.40, 28.46, 21.88; IR (ATR): 2977, 1700, 1590, 1454, 1323, 1160, 1106, 731 cm^{-1}; GC-MS (EI): 383 [M(^{37}Cl)$^{+}$, 2], 381 [M(^{35}Cl)$^{+}$, 7], 325 (54), 278 (37), 248 (40), 204 (100), 167 (6), 122 (6), 57 (64); HRMS (EI): Exact mass calcd for $C_{23}H_{24}NO_2{}^{35}Cl$ [M]$^{+}$: 381.1496, Found: 381.1499. (The distinct rotameric isomers were observed in the NMR spectra, due to the presence of sterically bulky Boc group in the product).

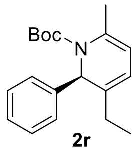
2r

Product **2r** was obtained in 51% yield as yellowish oil. HPLC analysis (Chiralpak AD-H, ^{i}PrOH/hexane = 0.5/99.5, 1.0 mL/min, 230 nm; t_r (minor) = 4.52 min, t_r (major) = 6.45 min) gave the isomeric composition of the product: 99% ee; $[\alpha]_D^{20}$ = -473.6 (*c* = 0.50, $CHCl_3$); ^1H NMR (400 MHz, $CDCl_3$): δ 7.36-7.34 (m, 2H), 7.30-7.24 (m, 3H), 5.86 (d, *J* = 5.2 Hz, 1H), 5.69 (s, 1H), 5.39 (d, *J* = 5.2 Hz, 1H), 2.16-2.05 (m, 2H), 1.97 (s, 3H), 1.53 (s, 9H), 1.06 (t, *J* = 7.2 Hz, 3H); ^{13}C NMR (100 MHz, $CDCl_3$): δ 153.88, 139.90, 137.54, 132.45, 128.28, 127.67, 127.63, 117.00, 112.30, 81.25, 58.00, 28.52, 27.19, 21.80, 11.87; IR (ATR): 2970, 1694, 1605, 1454, 1367, 1308, 1154, 714 cm^{-1}; GC-MS (EI): 299 (M^+, 12), 243 (71), 214 (25), 196 (33), 182 (10), 166 (62), 122 (100), 57 (58); HRMS (EI): Exact mass calcd for $C_{19}H_{25}NO_2$ $[M]^+$: 299.1885, Found: 299.1884.

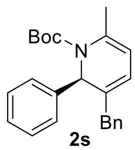
2s

Product **2s** was obtained in 60% yield as yellowish oil. HPLC analysis (Chiralpak AD-H, ^{i}PrOH/hexane = 0.5/99.5, 1.0 mL/min, 205 nm; t_r (minor) = 9.69 min, t_r (major) = 11.95 min) gave the isomeric composition of the product: 99% ee; $[\alpha]_D^{20}$ = -262.9 (*c* = 1.00, $CHCl_3$); ^1H NMR (400 MHz, $CDCl_3$): δ 7.38-7.36 (m, 2H), 7.33-7.25 (m, 5H), 7.22-7.15 (m, 3H), 5.78 (s, 1H), 5.63 (s, 1H), 5.38 (d, *J* = 5.2 Hz, 1H), 3.45 (d, *J* = 15.6 Hz, 1H), 3.28 (d, *J* = 15.6 Hz, 1H), 1.98 (s, 3H), 1.41 (s, 9H); ^{13}C NMR (100 MHz, $CDCl_3$): δ 153.48, 139.49, 138.95, 134.92, 133.23, 129.26, 128.50, 128.41, 127.80, 127.74, 126.41, 119.95, 112.16, 81.16, 57.40, 40.76, 28.32, 21.52; IR (ATR): 2974, 1702, 1604, 1454, 1334, 1158, 762, 699 cm^{-1}; GC-MS (EI): 361 (M^+, 13), 305 (85), 260 (22), 228 (58), 214 (32), 184 (32), 170 (16), 91 (39), 57 (45); HRMS (EI): Exact mass calcd for $C_{24}H_{27}NO_2$ $[M]^+$: 361.2042, Found: 361.2050.

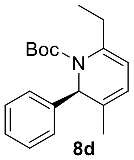
8d

Product **8d** was obtained in 46% yield as yellowish oil. HPLC analysis (Chiralpak AD-H, ^{i}PrOH/hexane = 0.5/99.5, 1.0 mL/min, 230 nm; t_r (minor) = 5.13 min, t_r (major) = 6.55 min) gave the isomeric composition of the product: >99% ee; $[\alpha]_D^{20}$ = -395.2 (*c* = 1.15, $CHCl_3$); ^1H NMR (400 MHz, $CDCl_3$): δ 7.36-7.34 (m, 2H), 7.29-7.22 (m, 3H), 5.90 (d, *J* = 5.2 Hz, 1H), 5.70 (s, 1H), 5.41 (d, *J* = 5.2 Hz, 1H), 2.65-2.60 (m, 1H), 2.18-2.08 (m, 1H), 1.81 (s, 3H), 1.53 (s, 9H), 0.54 (t, 3H); ^{13}C NMR (100 MHz, $CDCl_3$): δ 153.92, 138.47, 137.39, 132.28, 128.16, 128.11, 127.68, 119.34, 111.68, 81.16, 58.72, 28.48, 27.38, 20.78, 12.11; IR (ATR): 2972, 1689, 1604, 1454, 1390, 1366, 1326, 1159 cm^{-1}; GC-MS (EI): 299 (M^+, 16), 243 (82), 214 (15), 196 (9), 166 (69), 122 (100), 57 (69); HRMS (EI): Exact mass calcd for $C_{19}H_{25}NO_2$ $[M]^+$: 299.1885, Found: 299.1888.

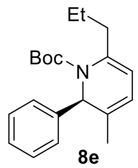
8e

Product **8e** was obtained in 44% yield as yellowish oil. HPLC analysis (Chiralpak AD-H, ^{i}PrOH/hexane = 0.5/99.5, 1.0 mL/min, 230 nm; t_r (minor) = 4.39 min, t_r (major) = 5.97 min) gave the isomeric composition of the product: 99% ee; $[\alpha]_D^{20}$ = -372.6 (*c* = 0.50, $CHCl_3$); ^{1}H NMR (400 MHz, $CDCl_3$): δ 7.36-7.35 (m, 2H), 7.29-7.22 (m, 3H), 5.91 (d, *J* = 5.2 Hz, 1H), 5.72 (s, 1H), 5.40 (d, *J* = 5.2 Hz, 1H), 2.77-2.70 (m, 1H), 1.98-1.89 (m, 1H), 1.81 (s, 3H), 1.53 (s, 9H), 1.11-1.02 (m, 1H), 0.82-0.74 (m, 1H), 0.47 (t, *J* = 7.2 Hz, 3H); ^{13}C NMR (100 MHz, $CDCl_3$): δ 154.09, 138.49, 135.97, 131.97, 128.20, 127.71, 119.49, 112.31, 81.20, 58.59, 36.51, 28.50, 20.84, 20.72, 13.36; IR (ATR): 2961, 1689, 1602, 1366, 1327, 1159, 1125, 740 cm^{-1}; GC-MS (EI): 313 (M^+, 14), 257 (75), 212 (24), 183 (16), 180 (74), 136 (100), 57 (59); HRMS (EI): Exact mass calcd for $C_{20}H_{27}NO_2$ $[M]^+$: 313.2042, Found: 313.2040.

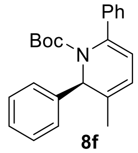
8f

In this case, the cycloisomerization reaction was carried out in anhydrous toluene (2.0 mL) with $SiCl_4$ (60 mol%), at 60 °C. Product **8f** was obtained in 20% yield as yellowish oil. HPLC analysis (Chiralpak AD-H, ^{i}PrOH/hexane = 0.5/99.5, 1.0 mL/min, 240 nm; t_r (minor) = 8.30 min, t_r (major) = 9.05 min) gave the isomeric composition of the product: 93% ee; $[\alpha]_D^{20}$ = -189.9 (*c* = 0.41, $CHCl_3$); ^{1}H NMR (400 MHz, $CDCl_3$): δ 7.46-7.44 (m, 2H), 7.34-7.25 (m, 3H), 7.20-7.15 (m, 3H), 7.04-7.01 (m, 2H), 6.12-6.10 (m, 1H), 5.86 (s, 1H), 5.80 (d, *J* = 5.2 Hz, 1H), 1.90 (s, 3H), 1.06 (s, 9H); ^{13}C NMR (100 MHz, MeOD-d_4): δ 154.31, 139.54, 138.11, 134.81, 134.13, 128.17, 127.74, 127.58, 127.54, 126.73, 124.97, 119.69, 113.24, 80.86, 58.65, 26.58, 19.47; IR (ATR): 2978, 1697, 1390, 1368, 1325, 1223, 912, 737 cm^{-1}; HRMS (ESI): Exact mass calcd for $C_{23}H_{25}NNaO_2$ $[M]^+$: 370.1777, Found: 370.1786.

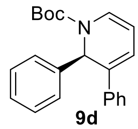
9d

Product **9d** was obtained in 55% yield as yellowish oil. HPLC analysis (Chiralpak AD-H, ^{i}PrOH/hexane = 0.5/99.5, 1.0 mL/min, 254 nm; t_r (minor) = 5.74 min, t_r (major) = 7.70 min) gave the isomeric composition of the product: 95% ee; $[\alpha]_D^{20}$ = -146.0 (*c* = 1.00, $CHCl_3$); ^{1}H NMR (400 MHz, $CDCl_3$): δ 7.49-7.43 (m, 2H), 7.41-7.34 (m, 2H), 7.29-7.23 (m, 5H), 7.21-7.16 (m, 1H), 6.88-6.61 (m, 2H), 6.54-6.25 (m, 1H), 5.58-5.47 (m, 1H), 1.55-1.51 (m, 9H); ^{13}C NMR (100 MHz, $CDCl_3$): δ 152.83, 152.65, 139.95, 139.54, 138.63, 138.21, 130.92, 130.59, 128.60, 128.45, 128.00, 127.93, 127.62, 127.37, 125.42, 125.29, 124.96, 119.83, 119.33, 106.81, 82.16, 81.82, 57.22, 55.07, 28.35; IR (ATR): 2978, 1705, 1368, 1163, 1115, 910, 758, 696 cm^{-1}; HRMS (ESI): Exact mass calcd for $C_{22}H_{23}NNaO_2$ $[M+Na]^+$: 356.1621, Found: 356.1615. (The distinct rotameric isomers were observed in the NMR spectra, due to the presence of sterically bulky Boc group).

General procedure for synthesis of 2g, 2k, 2t–y, 8a–c, 8g, 8h, 9a–c, 9e–j, and 10a–b

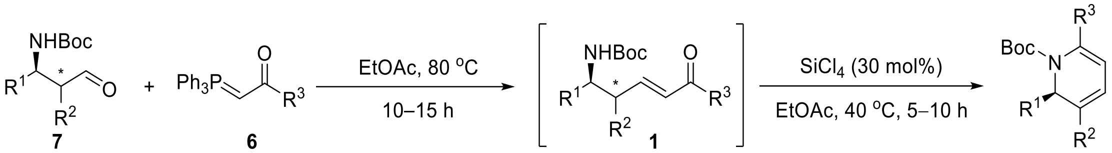

Chiral aldehydes **7** were synthesized according to literature method.[6–9] To a 10 mL oven-dried Schlenk tube (with high vacuum valve) was added chiral aldehyde **7** (0.5 mmol), phosphorus ylide **6** (0.5 mmol) and anhydrous EtOAc (2.0 mL). After being stirred at 80 °C for 10–15 h till full conversion of **7** by TLC analysis, $SiCl_4$ (18 μL, 0.15 mmol, 30 mol%) was then added in one portion at ambient temperature, and continued to stir at 40 °C for 5–10 h till full conversion of **1** by TLC analysis. The reaction mixture was dropwise added to saturated $NaHCO_3$ (aq., 15 mL) at 0 °C and extracted with EtOAc (10 mL × 3). The combined organic phases were washed with brine, dried over Na_2SO_4 and concentrated under reduced pressure to give the residue, which was purified by flash column chromatography using PE/Et_2O (20/1, v/v) as the elution to afford the corresponding products. [Note: In the case of **8a**–**c** and **9e**–**f**: After full conversion, the reaction mixture of the Wittig reaction was filtered through a short pad of silica gel and washed with PE/Et_2O (5/1, v/v), and concentrated and dried in vacuo to afford crude enones **1**, which was then subjected into a 10 mL oven-dried Schlenk tube, followed by the addition of Ph_3PO (139.0 mg, 0.5 mmol), anhydrous EtOAc (2.0 mL), and $SiCl_4$ (18 μL, 0.15 mmol, 30 mol%) for the next cycloisomerization.]

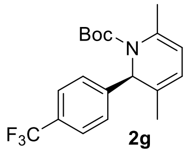

Product **2g** was obtained in 44% yield as yellowish oil. HPLC analysis (Chiralpak AD-H, ^{i}PrOH/hexane = 0.5/99.5, 1.0 mL/min, 205 nm; t_r (major) = 8.67 min, t_r (minor) = 11.15 min) gave the isomeric composition of the product: 98% ee; $[\alpha]_D^{20}$ = -375.8 (*c* = 1.00, $CHCl_3$); ^{1}H NMR (400 MHz, $CDCl_3$): δ 7.55 (d, *J* = 8.4 Hz, 2H), 7.48 (d, *J* = 8.4 Hz, 2H), 5.89 (d, *J* = 4.4 Hz, 1H), 5.67 (s, 1H), 5.35 (d, *J* = 5.2 Hz, 1H), 1.98 (s, 3H), 1.81 (s, 3H), 1.53 (s, 9H); ^{13}C NMR (100 MHz, $CDCl_3$): δ 153.93, 143.56, 132.26, 130.88, 129.90 (q, *J* = 32 Hz), 127.99, 125.36 (q, *J* = 3 Hz), 124.28 (q, *J* = 270 Hz), 119.62, 111.97, 81.73, 58.66, 28.46, 21.91, 20.86; ^{19}F NMR (376 MHz, $CDCl_3$): δ -62.48 (s, 3F); IR (ATR): 2980, 1689, 1608, 1368, 1322, 1222, 1159, 1129 cm^{-1}; GC-MS (EI): 353 (M^+, 6), 297 (44), 280 (6), 250 (20), 238 (6), 152 (46), 108 (82), 57 (100); HRMS (EI): Exact mass calcd for $C_{19}H_{22}NO_2F_3$ $[M]^+$: 353.1603, Found: 353.1592.

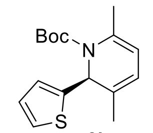
2k

Product **2k** was obtained in 66% yield as yellowish oil. HPLC analysis (Chiralpak AD-H, ^{i}PrOH/hexane = 0.5/99.5, 1.0 mL/min, 230 nm; t_r (minor) = 5.24 min, t_r (major) = 7. 53 min) gave the isomeric composition of the product: 98% ee; $[\alpha]_D^{20}$ = -544.4 (c = 1.00, $CHCl_3$); ^{1}H NMR (400 MHz, $CDCl_3$): δ 7.19-7.18 (m, 1H), 6.97 (d, J = 3.2 Hz, 1H), 6.92-6.90 (m, 1H), 5.84 (s, 1H), 5.78 (d, J = 5.2 Hz, 1H), 5.36 (d, J = 5.2 Hz, 1H), 2.03 (s, 3H), 1.85 (s, 3H), 1.54 (s, 9H); ^{13}C NMR (100 MHz, $CDCl_3$): δ 153.29, 142.87, 131.95, 131.57, 126.28, 125.47, 125.16, 118.93, 111.63, 81.59, 55.25, 28.47, 21.85, 20.54; IR (ATR): 2975, 1694, 1607, 1366, 1328, 1225, 1158, 700 cm^{-1}; GC-MS (EI): 291 (M^+, 18), 235 (100), 220 (43), 190 (51), 176 (42), 108 (54), 57 (90); HRMS (EI): Exact mass calcd for $C_{16}H_{21}NO_2S$ $[M]^+$: 291.1293, Found: 291.1287.

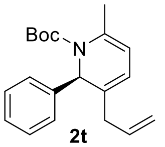
2t

Product **2t** was obtained in 61% yield as yellowish oil. HPLC analysis (Chiralpak AD-H, ^{i}PrOH/hexane = 0.5/99.5, 1.0 mL/min, 230 nm; t_r (minor) = 5.58 min, t_r (major) = 6.86 min) gave the isomeric composition of the product: 99% ee; $[\alpha]_D^{20}$ = -466.6 (c = 0.50, $CHCl_3$); ^{1}H NMR (400 MHz, $CDCl_3$): δ 7.37-7.34 (m, 2H), 7.30-7.26 (m, 3H), 5.88 (d, J = 5.2 Hz, 1H), 5.83-5.74 (m, 1H), 5.70 (s, 1H), 5.39 (d, J = 5.2 Hz, 1H), 5.08-5.03 (m, 2H), 2.90-2.74 (m, 2H), 1.98 (s, 3H), 1.52 (s, 9H); ^{13}C NMR (100 MHz, $CDCl_3$): δ 153.74, 139.61, 135.15, 133.64, 133.10, 128.34, 127.72, 127.70, 119.27, 116.93, 112.18, 81.29, 57.48, 38.75, 28.50, 21.75; IR (ATR): 2975, 1696, 1604, 1454, 1389, 1367, 1158, 700 cm^{-1}; GC-MS (EI): 311 (M^+, 11), 255 (66), 214 (23), 208 (13), 194 (10), 178 (55), 134 (100), 91 (5), 57 (71); HRMS (EI): Exact mass calcd for $C_{20}H_{25}NO_2$ $[M]^+$: 311.1885, Found: 311.1881.

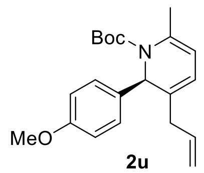
2u

Product **2u** was obtained in 51% yield as yellowish oil. HPLC analysis (Chiralpak AD-H, ^{i}PrOH/hexane = 0.5/99.5, 1.0 mL/min, 230 nm; t_r (minor) = 11.19 min, t_r (major) = 15.84 min gave the isomeric composition of the product: 99% ee; $[\alpha]_D^{20}$ = -156.6 (c = 0.50, $CHCl_3$); ^{1}H NMR (400 MHz, $CDCl_3$): δ 7.29-7.21 (m, 2H), 6.84-6.80 (m, 2H), 5.87-5.75 (m, 2H), 5.65 (s, 1H), 5.38 (d, J = 5.6 Hz, 1H), 5.07-5.02 (m, 2H), 3.78 (s, 3H), 2.87-2.71 (m, 2H), 1.98 (s, 3H), 1.52 (s, 9H); ^{13}C NMR (100 MHz, $CDCl_3$): δ 159.49, 159.21, 153.71, 135.19, 134.17, 133.91, 132.87, 131.62, 130.12, 129.00, 128.92, 121.66, 118.98, 117.63, 116.84, 113.99, 113.68, 111.99, 81.69, 81.18, 56.98, 55.24, 55.21, 38.62, 38.04, 28.49, 28.43, 21.76; IR (ATR): 2976, 1694, 1508, 1389, 1327, 1171, 912, 733 cm^{-1}; HRMS (ESI): Exact mass calcd for $C_{21}H_{27}NNaO_3$ $[M]^+$: 364.1883, Found: 364.1883. (The distinct rotameric isomers were observed in the NMR spectra, due to the presence of sterically bulky Boc group in the product).

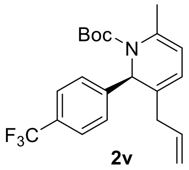

2v

Product **2v** was obtained in 56% yield as yellowish oil. HPLC analysis (Chiralpak AD-H, ^{i}PrOH/hexane = 0.5/99.5, 1.0 mL/min, 205 nm; t_r (minor) = 5.13 min, t_r (major) = 5.76 min gave the isomeric composition of the product: 91% ee; $[\alpha]_D^{20}$ = -132.3 (*c* = 0.41, $CHCl_3$); ^{1}H NMR (400 MHz, $CDCl_3$): δ 7.54 (d, *J* = 8.4 Hz, 2H), 7.47 (d, *J* = 8.4 Hz, 2H), 5.93 (d, *J* = 5.2 Hz, 1H), 5.81-5.73 (m, 2H), 5.39 (d, *J* = 5.2 Hz, 1H), 5.10-5.04 (m, 2H), 2.90-2.74 (m, 2H), 1.98 (s, 3H), 1.53 (s, 9H); ^{13}C NMR (100 MHz, $CDCl_3$): δ 153.67, 143.61, 134.79, 133.18, 132.72, 129.96 (q, *J* = 32 Hz), 128.07, 125.34 (q, *J* = 3 Hz), 124.28 (q, *J* = 270 Hz), 119.89, 117.24, 112.04, 81.73, 56.98, 38.77, 28.45, 21.76; ^{19}F NMR (376 MHz, $CDCl_3$): δ -62.49 (s, 3F); IR (ATR): 2978, 1417, 1386, 1368, 1321, 1159, 1122, 737 cm^{-1}; HRMS (ESI): Exact mass calcd for $C_{21}H_{24}F_3NNaO_2$ $[M]^+$: 402.1651, Found: 402.1653.

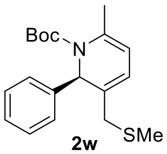

2w

Product **2w** was obtained in 70% yield as yellowish oil. HPLC analysis (Chiralpak AD-H, ^{i}PrOH/hexane = 0.5/99.5, 1.0 mL/min, 230 nm; t_r (minor) = 9.81 min, t_r (major) = 11.18 min) gave the isomeric composition of the product: 98% ee; $[\alpha]_D^{20}$ = -143.7 (*c* = 0.36, $CHCl_3$); ^{1}H NMR (400 MHz, $CDCl_3$): δ 7.37-7.27 (m, 5H), 6.04 (s, 1H), 5.98 (d, *J* = 5.2 Hz, 1H), 5.41 (d, *J* = 5.6 Hz, 1H), 3.22 (d, *J* = 14.0 Hz, 1H), 3.06 (d, *J* = 14.0 Hz, 1H), 2.03 (s, 3H), 1.98 (s, 3H), 1.54 (s, 9H); ^{13}C NMR (100 MHz, $CDCl_3$): δ 153.60, 139.44, 134.58, 130.45, 128.38, 127.81, 127.51, 121.37, 111.67, 81.41, 56.17, 37.63, 28.43, 21.64, 14.69; IR (ATR): 2976, 1715, 1391, 1366, 1252, 1221, 1176, 737 cm^{-1}; HRMS (ESI): Exact mass calcd for $C_{19}H_{25}NNaO_2S$ $[M+Na]^+$: 354.1498, Found: 354.1492.

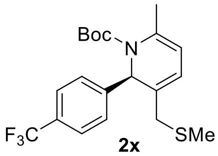

2x

Product **2x** was obtained in 56% yield as yellowish oil. HPLC analysis (Chiralpak AD-H, ^{i}PrOH/hexane = 0.5/99.5, 1.0 mL/min, 205 nm; t_r (minor) = 9.59 min, t_r (major) = 8.44 min gave the isomeric composition of the product: >99% ee; $[\alpha]_D^{20}$ = -108.2 (*c* = 0.50, $CHCl_3$); ^{1}H NMR (400 MHz, $CDCl_3$): δ 7.55 (d, *J* = 8.0 Hz, 2H), 7.48 (d, *J* = 8.0 Hz, 2H), 6.09 (s, 1H), 6.02 (d, *J* = 5.2 Hz, 1H), 5.41 (d, *J* = 5.2, 1H), 3.24 (d, *J* = 14.0 Hz, 1H), 3.05 (d, *J* = 14.0 Hz, 1H), 2.02 (s, 3H), 1.98 (s, 3H), 1.54 (s, 9H); ^{13}C NMR (100 MHz, $CDCl_3$): δ 153.51, 143.48, 134.70, 129.94 (q, *J* = 32 Hz), 129.62, 127.89, 125.39 (q, *J* = 3 Hz), 124.24 (q, *J* = 270 Hz), 121.94, 111.55, 81.87, 55.73, 37.66, 28.41, 21.67, 14.72; ^{19}F NMR (376 MHz, $CDCl_3$): δ -62.52 (s, 3F); IR (ATR): 1715, 1391, 1323, 1221, 1165, 11cm^{-1}; HRMS (ESI): Exact mass calcd for $C_{20}H_{24}F_3NNaO_2S$ $[M+Na]^+$: 422.1372, Found: 422.1377.

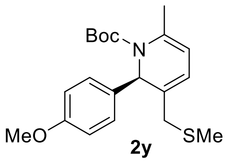

2y

Product **2y** was obtained in 70% yield as yellowish oil. HPLC analysis (Chiralpak AD-H, ^{i}PrOH/hexane = 0.5/99.5, 1.0 mL/min, 254 nm; t_r (minor) = 8.64 min, t_r (major) = 7.73 min) gave the isomeric composition of the product: 95% ee; $[\alpha]_D^{20}$ = -139.9 (*c* = 0.56, $CHCl_3$); ^{1}H NMR (400 MHz, $CDCl_3$): δ 7.35 (d, *J* = 8.8 Hz, 2H), 6.82 (d, *J* = 8.8 Hz, 2H), 5.98 (s, 1H), 5.96 (d, *J* = 5.2 Hz, 1H), 5.40 (d, *J* = 5.2, 1H), 3.78 (s, 3H), 3.19 (d, *J* = 14.0 Hz, 1H), 3.03 (d, *J* = 14.0 Hz, 1H), 2.02 (s, 3H), 1.97 (s, 3H), 1.54 (s, 9H); ^{13}C NMR (100 MHz, $CDCl_3$): δ 159.30, 153.61, 134.38, 131.49, 130.76, 128.88, 121.10, 113.74, 111.52, 81.33, 55.75, 55.27, 37.55, 28.46, 21.67, 14.74; IR (ATR): 2974, 1508, 1389, 1368, 1246, 1161, 1124, 733 cm^{-1}; HRMS (ESI): Exact mass calcd for $C_{20}H_{27}NNaO_3S$ $[M+Na]^+$: 384.1604, Found: 384.1607.

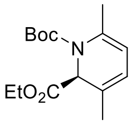

8a

Product **8a** was obtained in 33% yield as yellowish oil. HPLC analysis (Chiralpak AD-H, ^{i}PrOH/hexane = 0.5/99.5, 1.0 mL/min, 230 nm; t_r (minor) = 6.11 min, t_r (major) = 9.26 min) gave the isomeric composition of the product: 89% ee; $[\alpha]_D^{25}$ = -104.0 (*c* = 2.00, $CHCl_3$); ^{1}H NMR (400 MHz, $CDCl_3$): δ 5.68 (d, *J* = 4.8 Hz, 1H), 5.24 (d, *J* = 5.2 Hz, 1H), 5.19 (s, 1H), 4.22-4.10 (m, 2H), 2.18 (s, 3H), 2.00 (s, 3H), 1.51 (s, 9H), 1.25 (t, *J* = 7.2 Hz, 3H); ^{13}C NMR (100 MHz, $CDCl_3$): δ 170.47. 153.31, 134.35, 127.63, 119.80, 110.64, 81.87, 61.09, 59.22, 28.40, 21.69, 21.53, 14.19; IR (ATR): 2978, 2360, 1734, 1699, 1339, 1242, 912, 737 cm^{-1}; GC-MS (EI): 281 (M^+, 1), 208 (16), 153 (4), 152 (39), 109 (8), 108 (100), 107 (11), 106 (6), 57 (86); HRMS (EI): Exact mass calcd for $C_{15}H_{23}NO_4$ $[M]^+$: 281.1627, Found: 281.1631.

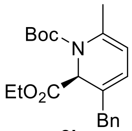

8b

Product **8b** was obtained in 65% yield as yellowish oil. HPLC analysis (Chiralpak IA, ^{i}PrOH/hexane = 0.5/99.5, 0.5 mL/min, 230 nm; t_r (minor) = 18.00 min, t_r (major) = 20.68 min) gave the isomeric composition of the product: 91% ee; $[\alpha]_D^{25}$ = -95.5 (*c* = 1.00, $CHCl_3$); ^{1}H NMR (400 MHz, $CDCl_3$): δ 7.31-7.27 (m, 2H), 7.23-7.20 (m, 3H), 5.60 (s, 1H), 5.28 (d, *J* = 5.2 Hz, 1H), 5.18 (s, 1H), 4.24-4.12 (m, 2H), 3.71 (d, *J* = 16.0 Hz, 1H), 3.61 (d, *J* = 16.0 Hz, 1H), 2.19 (s, 3H), 1.37 (s, 9H), 1.29 (t, *J* = 7.2 Hz, 3H); ^{13}C NMR (100 MHz, $CDCl_3$): δ 170.65, 152.77, 138.79, 135.40, 131.01, 129.30, 128.54, 126.48, 120.45, 110.69, 81.65, 61.17, 57.34, 41.18, 28.16, 22.72, 14.24; IR (ATR): 2980, 2361, 2342, 1740, 1699, 1342, 912, 737 cm^{-1}; GC-MS (EI): 357 (M^+, 1), 284 (11), 228 (46), 209 (2), 185 (14), 184 (100), 91 (11), 57 (82); HRMS (EI): Exact mass calcd for $C_{21}H_{27}NO_4$ $[M]^+$: 357.1940, Found: 357.1942.

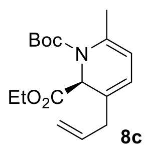

8c

Product **8c** was obtained in 45% yield as yellowish oil. HPLC analysis (Chiralpak AD-H, ^{i}PrOH/hexane = 0.5/99.5, 0.8 mL/min, 205 nm; t_r (minor) = 8.42 min, t_r (major) = 10.10 min) gave the isomeric composition: 92% ee; $[\alpha]_D^{25}$ = -243.4 (*c* = 1.00, $CHCl_3$); ^{1}H NMR (500 MHz, $CDCl_3$): δ 5.85-5.77 (m, 1H), 5.70 (d, *J* = 5.5 Hz, 1H), 5.30 (d, *J* = 5.0 Hz, 1H), 5.26 (s, 1H), 5.13-5.10 (m, 2H), 4.19-4.12 (m, 2H), 3.13-3.03 (m, 2H), 2.19 (s, 3H), 1.50 (s, 9H), 1.25 (t, *J* = 7.0 Hz, 3H); ^{13}C NMR (125 MHz, $CDCl_3$): δ 170.62, 153.00, 134.98, 129.64, 119.92, 118.23, 117.16, 110.71, 81.84, 61.13, 57.34, 39.11, 28.33, 21.57, 14.17; IR (ATR): 2978, 2359, 2342, 1730, 1699, 1458, 1340, 735 cm^{-1}; GC-MS (EI): 307 (M^+, 1), 234 (16), 179 (5), 178 (45), 135 (10), 134 (95), 132 (5), 57 (100); HRMS (EI): Exact mass calcd for $C_{17}H_{25}NO_4$ $[M]^+$: 307.1784, Found: 307.1786.

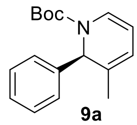

9a

Product **9a** was obtained in 42% yield as yellowish oil. HPLC analysis (Chiralpak AD-H, ^{i}PrOH/hexane = 0.5/99.5, 1.0 mL/min, 235 nm; t_r (minor) = 6.10 min, t_r (major) = 6.60 min) gave the isomeric composition of the product: 98% ee; $[\alpha]_D^{20}$ = -110.6 (*c* = 0.90, $CHCl_3$); ^{1}H NMR (400 MHz, $CDCl_3$): δ 7.46-7.38 (m, 2H), 7.31-7.23 (m, 3H), 6.83-6.56 (m, 1H), 5.83-5.20 (m, 3H), 1.70-1.64 (m, 3H), 1.45-1.39 (m, 9H); ^{13}C NMR (100 MHz, $CDCl_3$): δ 153.21, 152.60, 141.75, 140.27, 131.10, 131.00, 128.42, 128.30, 127.88, 127.79, 127.21, 123.57, 123.33, 117.49, 117.10, 104.92, 104.13, 81.50, 81.34, 61.66, 59.19, 28.33, 20.95; IR (ATR): 1715, 1690, 1391, 1366, 1221, 1176, 914, 737 cm^{-1}; HRMS (EI): Exact mass calcd for $C_{17}H_{21}NNaO_2$ $[M]^+$: 294.1464, Found: 294.1464. (The distinct rotameric isomers were observed in the NMR spectra, due to the presence of sterically bulky Boc group in the product).

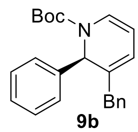

9b

Product **9b** was obtained in 30% yield as yellowish oil. HPLC analysis (Chiralpak AD-H, ^{i}PrOH/hexane = 0.5/99.5, 1.0 mL/min, 230 nm; t_r (minor) = 7.24 min, t_r (major) = 7.56 min) gave the isomeric composition of the product: 99% ee; $[\alpha]_D^{20}$ = -142.6 (*c* = 0.68, $CHCl_3$); ^{1}H NMR (400 MHz, $CDCl_3$): δ 7.49-7.39 (m, 2H), 7.32-7.20 (m, 6H), 7.12 (d, *J* = 7.2 Hz, 2H), 6.68 (dd, *J* = 81.6, 7.6 Hz, 1H), 5.72-5.42 (m, 2H), 5.23 (dt, *J* = 36.4, 6.8 Hz, 1H), 3.32-3.11 (m, 2H), 1.43-1.34 (m, 9H); ^{13}C NMR (100 MHz, $CDCl_3$): δ 153.04, 152.36, 141.18, 140.25, 138.46, 138.17, 134.70, 134.32, 129.44, 129.33, 128.48, 128.08, 127.57, 126.47, 124.04, 123.87, 118.37, 118.30, 104.57, 81.42, 59.30, 57.99, 40.51, 28.31, 28.20; IR (ATR): 2361, 1705, 1368, 1327, 1163, 1115, 1255, 910 cm^{-1}; HRMS (ESI): Exact mass calcd for $C_{23}H_{25}NNaO_2$ $[M+Na]^+$: 370.1777, Found: 370.1765. (The distinct rotameric isomers were observed in the NMR spectra, due to the presence of sterically bulky Boc group in the product).

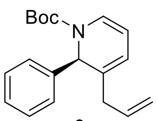

9c

Product **9c** was obtained in 30% yield as yellowish oil. HPLC analysis (Chiralpak AD-H, ^{i}PrOH/hexane = 0.5/99.5, 1.0 mL/min, 205 nm; t_r (minor) = 5.77 min, t_r (major) = 5.49 min) gave the isomeric composition of the product: >99% ee; $[\alpha]_D^{20}$ = -160.6 (*c* = 0.49, $CHCl_3$); ^{1}H NMR (500 MHz, $CDCl_3$): δ 7.46-7.38 (m, 2H), 7.31-7.26 (m, 3H), 6.82-6.58 (m, 1H), 5.89-5.81 (m, 1H), 5.77-5.47 (m, 2H), 5.30-5.23 (m, 1H), 5.08-5.03 (m, 2H), 2.77-2.58 (m, 2H), 1.45-1.41 (m, 9H); ^{13}C NMR (125 MHz, $CDCl_3$): δ 153.17, 152.50, 141.40, 140.17, 134.93, 134.67, 133.19, 132.98, 128.45, 128.34, 127.98, 127.43, 124.13, 123.92, 117.61, 117.50, 117.45, 117.23, 104.84, 104.40, 81.60, 81.44, 59.66, 57.62, 38.68, 38.48, 28.35, 28.27; IR (ATR): 2978, 1709, 1339, 1323, 1134, 1155, 914, 735 cm^{-1}; HRMS (ESI): Exact mass calcd for $C_{19}H_{23}NNaO_2$ $[M+Na]^+$: 320.1621, Found: 320.1602. (The distinct rotameric isomers were observed in the NMR spectra, due to the presence of sterically bulky Boc group in the product).

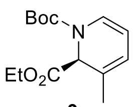

9e

Product **9e** was obtained in 20% yield as yellowish oil. HPLC analysis (Chiralpak IA, ^{i}PrOH/hexane = 1/99, 0.8 mL/min, 230 nm; t_r (minor) = 6.79 min, t_r (major) = 10.66 min) gave the isomeric composition of the product: 86% ee; $[\alpha]_D^{25}$ = -57.7 (*c* = 1.00, $CHCl_3$); ^{1}H NMR (400 MHz, $CDCl_3$): δ 6.86-6.71 (m, 1H), 5.74 (d, *J* = 5.2 Hz, 1H), 5.17-5.02 (m, 2H), 4.26-4.13 (m, 2H), 1.94 (d, *J* = 16.4 Hz, 3H), 1.50 (d, *J* = 14.4 Hz, 9H), 1.26 (t, *J* = 7.2 Hz, 3H); ^{13}C NMR (125 MHz, $CDCl_3$): δ 169.77, 169.73, 152.62, 152.55, 125.45, 124.73, 124.20, 124.05, 119.99, 119.48, 103.67, 103.27, 82.07, 61.29, 60.42, 58.70, 28.27, 28.17, 21.65, 21.49, 14.36, 14.24; IR (ATR): 2978, 2359, 1734, 1699, 1458, 1340, 912, 735 cm^{-1}; GC-MS (EI): 267 (M^+, 2), 194 (24), 139 (8), 138 (83), 95 (19), 94 (100), 93 (18), 57 (95); HRMS (EI): Exact mass calcd for $C_{14}H_{21}NO_4$ $[M]^+$: 267.1471, Found: 267.1468.

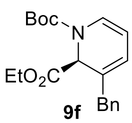

9f

Product **9f** was obtained in 22% yield as yellowish oil. HPLC analysis (Chiralpak IA, ^{i}PrOH/hexane = 1/99, 0.8 mL/min, 230 nm; t_r (minor) = 9.10 min, t_r (major) = 10.46 min) gave the isomeric composition of the product: 86% ee; $[\alpha]_D^{25}$ = -17.3 (*c* = 1.00, $CHCl_3$); ^{1}H NMR (500 MHz, $CDCl_3$): δ 7.33-7.22 (m, 5H), 6.85-6.72 (m, 1H), 5.70-5.61 (m, 1H), 5.22-5.04 (m, 2H), 4.25-4.16 (m, 2H), 3.69-3.54 (m, 2H), 1.44 (d, *J* = 49.5 Hz, 9H), 1.30 (t, *J* = 7.0 Hz, 3H); ^{13}C NMR (125 MHz, $CDCl_3$): δ 170.01, 169.79, 152.50, 152.38, 138.34, 138.12, 129.44, 129.35, 128.58, 128.55, 126.60, 124.83, 124.72 120.81, 120.32, 103.86, 103.40, 82.16, 82.02, 61.41, 61.36, 57.95, 57.10, 41.10, 40.99, 28.25, 28.06, 14.40, 14.32; IR (ATR): 2978, 2359, 1734, 1699, 1456, 1340, 1213, 912, 737 cm^{-1}; GC-MS (EI): 343 (M^+, 1), 270 (12), 215 (11), 214 (71), 171 (13), 170 (100), 168 (8),

57 (78); HRMS (EI): Exact mass calcd for $C_{20}H_{25}NO_4$ $[M]^+$: 343.1784, Found: 343.1789.

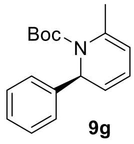

9g

Product **9g** was obtained in 63% yield as colorless oil. HPLC analysis (Chiralpak AD-H, ^{i}PrOH/hexane = 0.5/99.5, 1.0 mL/min, 230 nm; t_r (minor) = 7.08 min, t_r (major) = 9.14 min) gave the isomeric composition of the product: 98% ee; $[\alpha]_D^{25}$ = -635.4 (*c* = 1.10, $CHCl_3$); ^{1}H NMR (400 MHz, $CDCl_3$): δ 7.37-7.22 (m, 5H), 6.08-6.05 (m, 1H), 5.90-5.84 (m, 2H), 5.38 (d, *J* = 5.2 Hz, 1H), 2.07 (s, 3H), 1.53 (s, 9H); ^{13}C NMR (100 MHz, $CDCl_3$): δ 153.90, 140.88, 135.22, 128.30, 127.45, 126.80, 123.06, 122.35, 111.83, 81.47, 54.43, 28.44, 22.33; IR (ATR): 2975, 1694, 1476, 1454, 1380, 1332, 1166, 1103 cm^{-1}; HRMS (ESI): Exact mass calcd for $C_{17}H_{21}NNaO_2$ $[M+Na]^+$: 294.1464; Found: 294.1461.

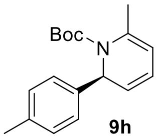

9h

Product **9h** was obtained in 45% yield as colorless oil. HPLC analysis (Chiralpak AD-H, ^{i}PrOH/hexane = 0.5/99.5, 1.0 mL/min, 254 nm; t_r (minor) = 5.91 min, t_r (major) = 11.15 min) gave the isomeric composition of the product: 95% ee; $[\alpha]_D^{20}$ = -79.0 (*c* = 2.00, $CHCl_3$); ^{1}H NMR (400 MHz, $CDCl_3$): δ 7.25 (d, *J* = 8.0 Hz, 2H), 7.10 (d, *J* = 8.0 Hz, 2H), 6.07-6.03 (m, 1H), 5.86-5.82 (m, 2H), 5.37 (d, *J* = 5.2 Hz, 1H), 2.31 (s, 3H), 2.06 (s, 3H), 1.52 (s, 9H); ^{13}C NMR (100 MHz, $CDCl_3$): δ 153.91, 137.91, 137.11, 135.18, 129.01, 126.82, 122.89, 122.56, 111.80, 81.39, 54.29, 28.46, 22.33, 21.19; IR (ATR): 2978, 2359, 1383, 1337, 1173, 1051, 912, 737 cm^{-1}; GC-MS (EI): 285 (M^+, 6), 229 (59), 185 (10), 184 (43), 170 (8), 138 (14), 94 (89), 57 (100); HRMS (EI): Exact mass calcd for $C_{18}H_{23}NO_2$ $[M]^+$: 285.1729, Found: 285.1725.

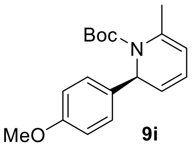

9i

Product **9i** was obtained in 32% yield as colorless oil. HPLC analysis (Chiralpak AD-H, ^{i}PrOH/hexane = 0.5/99.5, 1.0 mL/min, 230 nm; t_r (minor) = 8.56 min, t_r (major) = 19.42 min) gave the isomeric composition of the product: 98% ee; $[\alpha]_D^{20}$ = -38.7 (*c* = 2.00, $CHCl_3$); ^{1}H NMR (400 MHz, $CDCl_3$): δ 7.29 (d, *J* = 8.8 Hz, 2H), 6.83 (d, *J* = 8.8 Hz, 2H), 6.06 (dd, *J* = 8.4, 5.6 Hz, 1H), 5.85-5.79 (m, 2H), 5.38 (d, *J* = 5.2 Hz, 1H), 3.78 (s, 3H), 2.04 (s, 3H), 1.53 (s, 9H); ^{13}C NMR (100 MHz, $CDCl_3$): δ 159.07, 153.92, 135.06, 132.89, 128.31, 122.89, 122.58, 113.68, 111.73, 81.39, 55.28, 53.98, 28.48, 22.32; IR (ATR): 2976, 2359, 1730, 1699, 1383, 1342, 912, 737 cm^{-1}; GC-MS (EI): 301 (M^+, 5), 245 (44), 201 (17), 200 (47), 199 (10), 121 (11), 94 (63), 57 (100); HRMS (EI): Exact mass calcd for $C_{18}H_{23}NO_3$ $[M]^+$: 301.1678, Found: 301.1676.

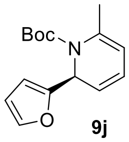

9j

Product **9j** was obtained in 41% yield as colorless oil. HPLC analysis (Chiralpak AD-H, ^{i}PrOH/hexane = 0.5/99.5, 1.0 mL/min, 230 nm; t_r (minor) = 6.59 min, t_r (major) = 9.06 min) gave the isomeric composition of the product: 95% ee; $[\alpha]_D^{25}$ = -106.8 (*c* = 2.00, $CHCl_3$); ^1H NMR (400 MHz, $CDCl_3$): δ 7.36 (s, 1H), 6.27-6.25 (m, 1H), 6.15 (d, *J* = 3.2 Hz, 1H), 6.02 (dd, *J* = 9.2, 5.2 Hz, 1H), 5.98 (d, *J* = 6.4 Hz, 1H), 5.73 (dd, *J* = 9.2, 6.4 Hz, 1H), 5.39 (d, *J* = 5.2, 1H), 2.07 (s, 3H), 1.53 (s, 9H); ^{13}C NMR (100 MHz, $CDCl_3$): δ 153.47, 153.20, 142.50, 135.16, 124.06, 119.48, 111.18, 109.98, 107.50, 81.62, 49.25, 28.41, 22.04; IR (ATR): 2978, 2359, 1699, 1383, 1338, 1172, 910, 735 cm^{-1}; GC-MS (EI): 261 (M^+, 10), 205 (53), 161 (15), 160 (53), 159 (14), 146 (27), 132 (9), 57 (100); HRMS (EI): Exact mass calcd for $C_{15}H_{19}NO_3$ $[M]^+$: 261.1365, Found: 261.1363.

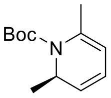

9k

Product **9k** was obtained in 21% yield as yellowish oil. HPLC analysis (Chiralpak AD-H, ^{i}PrOH/hexane = 0.5/99.5, 1.0 mL/min, 254 nm; t_r (major) = 4.80 min) gave the isomeric composition: >99% ee; $[\alpha]_D^{25}$ = -604.4 (*c* = 0.15, $CHCl_3$); ^1H NMR (400 MHz, CD_2Cl_2): δ 5.74-5.71 (m, 1H), 5.53-5.49 (m, 1H), 5.25-5.23 (m, 1H), 4.71-4.65 (m, 1H), 2.01 (s, 3H), 1.39 (s, 9H), 0.93 (d, *J* = 6.8 Hz, 3H); ^{13}C NMR (100 MHz, CD_2Cl_2): δ 153.08, 134.07, 124.70, 121.25, 110.42, 80.63, 48.45, 28.04, 21.99, 17.08; IR (ATR): 2972, 2926, 1707, 1587, 1389, 1128, 1061, 716 cm^{-1}; HRMS (ESI): Exact mass calcd for $C_{12}H_{19}NNaO_2$ $[M+Na]^+$: 232.1308, Found: 232.1308.

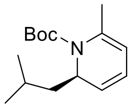

9l

Product **9l** was obtained in 23% yield as yellowish oil. HPLC analysis (Chiralpak AD-H, ^{i}PrOH/hexane = 0.1/99.9, 0.5 mL/min, 254 nm; t_r (major) = 10.00 min) gave the isomeric composition: >99% ee; $[\alpha]_D^{25}$ = -353.0 (*c* = 0.08, $CHCl_3$); ^1H NMR (400 MHz, CD_2Cl_2): δ 5.76-5.72 (m, 1H), 5.58-5.54 (m, 1H), 5.31-5.29 (m, 1H), 4.70-4.64 (m, 1H), 2.01 (s, 3H), 1.57-1.52 (m, 1H), 1.39 (s, 9H), 1.28-1.23 (m, 1H), 1.16-1.11 (m, 1H), 0.85 (d, *J* = 6.8 Hz, 3H), 0.82 (d, *J* = 6.8 Hz, 3H); ^{13}C NMR (125 MHz, CD_2Cl_2): δ 153.37, 134.31, 124.57, 121.59, 111.49, 80.52, 50.54, 40.32, 28.00, 23.70, 22.66, 22.58, 21.68; IR (ATR): 2957, 2928, 2359, 1389, 1336, 1175, 1080, 714 cm^{-1}; HRMS (ESI): Exact mass calcd for $C_{15}H_{25}NNaO_2$ $[M+Na]^+$: 274.1777, Found: 274.1773.

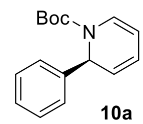

Product **10a** was obtained in 42% yield as yellowish oil. HPLC analysis (Chiralcel OD-H, ^{i}PrOH/hexane = 0.5/99.5, 1.0 mL/min, 230 nm; t_r (minor) = 5.92 min, t_r (major) = 6.69 min) gave the isomeric composition of the product: 97% ee; $[\alpha]_D^{20}$ = -163.1 (*c* = 0.9, $CHCl_3$); ^1H NMR (400 MHz, $CDCl_3$): δ 7.41-7.35 (m, 2H), 7.33-7.23 (m, 3H), 6.96-6.73 (m, 1H), 6.02-5.61 (m, 3H), 5.23 (s, 1H), 1.47-1.36 (m, 9H); ^{13}C NMR (100 MHz, $CDCl_3$): δ 153.26, 152.56, 143.25, 141.74, 128.53, 127.68, 126.86, 126.19, 122.60, 122.33, 121.03, 120.45, 104.35, 103.75, 81.60, 57.28, 54.90, 28.29, 28.16; IR (ATR): 1735, 1512, 1368, 1163, 1115, 978, 914, 735 cm^{-1}; HRMS (ESI): Exact mass calcd for $C_{16}H_{19}NNaO_2$ $[M+Na]^+$: 280.1308, Found: 280.1301. (The distinct rotameric isomers were observed in the NMR spectra, due to the presence of sterically bulky Boc group in the product).

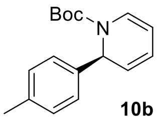

Product **10b** was obtained in 32% yield as yellowish oil. HPLC analysis (Chiralcel OD-H, ^{i}PrOH/hexane = 0.5/99.5, 1.0 mL/min, 230 nm; t_r (minor) = 5.43 min, t_r (major) = 5.03 min) gave the isomeric composition of the product: 99% ee; $[\alpha]_D^{20}$ = -163.1 (*c* = 0.90, $CHCl_3$); ^1H NMR (500 MHz, $CDCl_3$): δ 7.35-7.25 (m, 2H), 7.11 (d, *J* = 8.0 Hz, 2H), 6.93-6.72 (m, 1H), 6.01-5.59 (m, 3H), 5.22 (s, 1H), 2.32 (s, 3H), 1.43 (d, *J* = 42.0 Hz, 9H); ^{13}C NMR (125 MHz, $CDCl_3$): δ 153.30, 152.56, 140.08, 138.75, 137.37, 129.18, 126.89, 126.26, 126.10, 122.72, 122.44, 120.93, 120.43, 104.37, 103.94, 81.52, 56.78, 54.58, 28.31, 28.21, 21.21; IR (ATR): 2974, 1456, 1366, 1321, 1252, 1161, 976, 773 cm^{-1}; HRMS (ESI): Exact mass calcd for $C_{17}H_{21}NNaO_2$ $[M+Na]^+$: 294.1464, Found: 294.1452. (The distinct rotameric isomers were observed in the NMR spectra, due to the presence of sterically bulky Boc group in the product).

General procedure for synthesis of chiral dihydropyridine-based spirooxindole 11

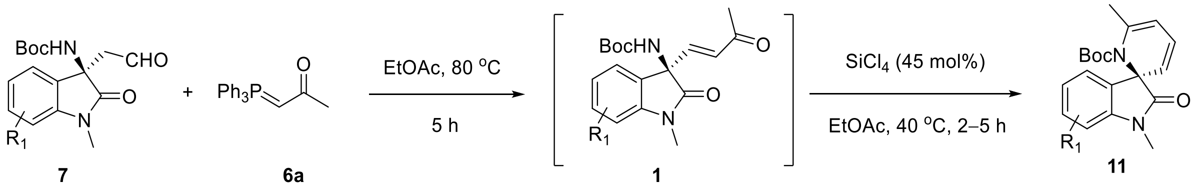

(*R*)-Configurated oxindole based chiral aldehydes **7** were obtained by literature method.[10] To a 10 mL oven-dried Schlenk tube was added chiral aldehyde **7** (0.3 mmol), phosphorus ylide **6a** (95.4 mg, 0.3 mmol) and anhydrous EtOAc (2.0 mL). The reaction was stirred at 80 °C till full consumption of **7** by TLC analysis. Then $SiCl_4$ (16 μL, 0.14 mmol, 45 mol%) was added at 25 °C. The mixture was kept stirring at 40 °C for 2–5 h till full consumption of **1**. The mixture was dropwise added to saturated

$NaHCO_3$ (aq., 15 mL) at 0 °C and extracted with EtOAc (10 mL × 3). The combined organic phases were washed with brine, dried over Na_2SO_4 and concentrated to give the residue, which was purified by flash column chromatography using PE/EtOAc (5/1, v/v) as the elution.

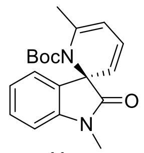

11a

Product **11a** was obtained in 39% yield as yellowish oil. HPLC analysis (Chiralpak AD-H, ^{i}PrOH/hexane = 1/99, 1.0 mL/min, 230 nm; t_r (minor) = 29.44 min, t_r (major) = 32.04 min) gave the isomeric composition of the product: 85% ee; $[\alpha]_D^{25}$ = -4.8 (*c* = 1.00, $CHCl_3$); ^1H NMR (400 MHz, $CDCl_3$): δ 7.40 (d, *J* = 7.2 Hz, 1H), 7.29 (td, *J* = 7.6, 1.2 Hz, 1H), 7.05 (t, *J* = 7.2 Hz, 1H), 6.80 (d, *J* = 8.0 Hz, 1H), 5.92 (dd, *J* = 9.2, 5.6 Hz, 1H), 5.06 (d, *J* = 6.0 Hz, 1H), 4.95 (d, *J* = 9.6 Hz, 1H), 3.22 (s, 3H), 2.24 (s, 3H), 1.20 (s, 9H); ^{13}C NMR (100 MHz, $CDCl_3$): δ 176.63, 152.14, 140.93, 136.55, 133.86, 129.05, 123.23, 122.84, 122.17, 119.54, 108.08, 104.62, 82.45, 67.66, 27.77, 26.60, 22.96; IR (ATR): 2976, 2361, 1730, 1699, 1383, 1340, 1175, 737 cm^{-1}; HRMS (ESI): Exact mass calcd for $C_{19}H_{22}N_2NaO_3$ $[M+Na]^+$: 349.1523, Found: 349.1509.

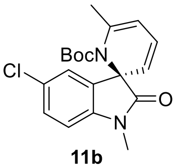

11b

Product **11b** was obtained in 31% yield as yellowish oil. HPLC analysis (Chiralcel OJ-H, ^{i}PrOH/hexane = 5/95, 1.0 mL/min, 230 nm; t_r (minor) = 21.31 min, t_r (major) = 10.92 min) gave the isomeric composition of the product: 87% ee; $[\alpha]_D^{25}$ = -0.8 (*c* = 0.50, $CHCl_3$); ^1H NMR (400 MHz, $CDCl_3$): δ 7.38 (d, *J* = 7.2 Hz, 1H), 7.28-7.25 (m, 2H), 6.73 (d, *J* = 8.0 Hz, 1H), 5.95 (dd, *J* = 9.2, 5.6 Hz, 1H), 5.09 (d, *J* = 6.0 Hz, 1H), 4.94 (d, *J* = 9.6 Hz, 1H), 3.21 (s, 3H), 2.24 (s, 3H), 1.27 (s, 9H); ^{13}C NMR (100 MHz, CDCl3): δ 176.24, 152.09, 139.51, 136.51, 134.98, 128.98, 128.42, 123.40, 122.74, 118.87, 109.09, 104.86, 82.91, 67.56, 27.89, 26.76, 23.02; IR (ATR): 1730, 1703, 1599, 1340, 1383, 1300, 1271, 760 cm^{-1}; HRMS (ESI): Exact mass calcd for $C_{19}H_{21}{}^{35}ClN_2NaO_3$ $[M+Na]^+$: 383.1133, Found: 383.1131.

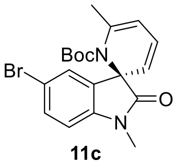

11c

Product **11c** was obtained in 31% yield as yellowish oil. HPLC analysis (Chiralcel OJ-H, ^{i}PrOH/hexane = 5/95, 1.0 mL/min, 230 nm; t_r (minor) = 24.80 min, t_r (major) = 12.11 min) gave the isomeric composition of the product: 87% ee; $[\alpha]_D^{25}$ = -8.6 (*c* = 0.15, $CHCl_3$); ^1H NMR (400 MHz, $CDCl_3$): δ 7.50 (d, *J* = 2.0 Hz, 1H), 7.42 (dd, *J* = 8.4, 2.0 Hz, 1H), 6.68 (d, *J* = 8.4 Hz, 1H), 5.95 (dd, *J* = 9.2, 5.6 Hz, 1H), 5.08 (d, *J* = 5.6 Hz, 1H), 4.93 (d, *J* = 9.6 Hz, 1H), 3.20 (s, 3H), 2.24 (s, 3H), 1.27 (s, 9H); ^{13}C NMR (100 MHz, $CDCl_3$): δ 176.10, 152.07, 139.97, 136.48, 135.29, 131.89, 126.10, 122.75, 118.81, 115.64, 109.63, 104.84, 82.92, 67.49, 29.78, 27.88, 26.73; IR (ATR): 2978, 2361, 1730, 1697, 1342, 1051, 912, 737 cm^{-1};

HRMS (ESI): Exact mass calcd for $C_{19}H_{22}{}^{79}BrN_2O_3$ $[M+H]^+$: 405.0808, Found: 405.0804.

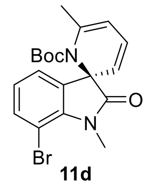

11d

Product **11d** was obtained in 32% yield as yellowish oil. HPLC analysis (Chiralpak AD-H, ^{i}PrOH/hexane = 1/99, 1.0 mL/min, 230 nm; t_r (minor) = 12.37 min, t_r (major) = 16.68 min) gave the isomeric composition: 88% ee; $[\alpha]_D^{25}$ = -0.3 (c = 0.50, $CHCl_3$); ^1H NMR (500 MHz, $CDCl_3$): δ 7.39 (dd, J = 8.0, 1.0 Hz, 1H), 7.34 (dd, J = 7.0, 1.0 Hz, 1H), 6.89 (dd, J = 8.0, 7.5 Hz, 1H), 5.92 (dd, J = 9.5, 5.5 Hz, 1H), 5.05 (dt, J = 6.0, 1.0 Hz, 1H), 4.92 (d, J = 9.5 Hz, 1H), 3.60 (s, 3H), 2.24 (s, 3H), 1.24 (s, 9H); ^{13}C NMR (125 MHz, $CDCl_3$): δ 177.05, 151.96, 138.24, 136.98, 136.57, 134.49, 124.53, 122.32, 121.90, 118.97, 104.37, 102.45, 82.91, 67.30, 30.13, 27.78, 23.00; IR (ATR): 1732, 1703, 1605, 1699, 1578, 1298, 1103, 754 cm^{-1}; HRMS (ESI): Exact mass calcd for $C_{19}H_{21}{}^{79}BrN_2NaO_3$ $[M+Na]^+$: 427.0628, Found: 427.0623.

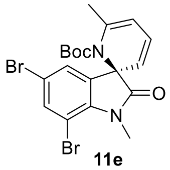

11e

Product **11e** was obtained in 31% yield as yellowish oil. HPLC analysis (Chiralpak AD-H, ^{i}PrOH/hexane = 1/99, 1.0 mL/min, 230 nm; t_r (minor) = 9.45 min, t_r (major) = 13.52 min) gave ee value: 88% ee; $[\alpha]_D^{25}$ = -3.8 (c = 0.50, $CHCl_3$); ^1H NMR (500 MHz, $CDCl_3$): δ 7.55 (d, J = 2.0 Hz, 1H), 7.43 (d, J = 1.5, Hz, 1H), 5.95 (dd, J = 9.5, 6.0 Hz, 1H), 5.08-5.06 (m, 1H), 4.91 (d, J = 9.5 Hz, 1H), 3.58 (s, 3H), 2.24, (s, 3H), 1.30 (s, 9H); ^{13}C NMR (125 MHz, $CDCl_3$): δ 176.56, 151.93, 137.87, 137.50, 136.49, 136.39, 125.09, 122.88, 118.30, 115.84, 104.58, 102.91, 83.34, 67.22, 30.16, 27.87, 23.08; IR (ATR): 2994, 1740, 1705, 1600, 1458, 1369, 1074, 758 cm^{-1}; HRMS (ESI): Exact mass calcd for $C_{19}H_{20}{}^{79}Br_2N_2NaO_3$ $[M+Na]^+$: 504.9733, Found: 504.9724.

Sequence to polysubstituted pyridines and 3-hydroxyl pyridines

The synthesis of polysubstituted pyridines 3a–d

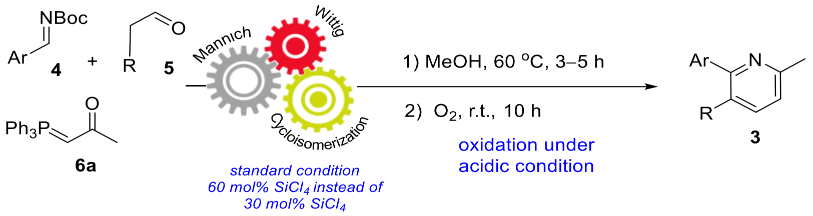

The Mannich/Wittig/cycloisomerization sequence involving *N*-Boc imine **4** (0.5 mmol), aldehyde **5** (1.0 mmol for synthesis of **3a**, 0.5 mmol for synthesis of **3b**–**d**) and phosphorane **6a** (1.0 equiv, based on intermediate **7**) was conducted using the general procedures in the section 4, except using 60 mol% $SiCl_4$. After the completion of cycloisomerization, anhydrous MeOH (1.0 mL) was added to the reaction mixture at ambient temperature and stirred at 60 °C for 3–5 h till full deprotection of intermediate **2** by TLC analysis. The resulting mixture was stirred for further 10 h at ambient temperature under O_2 atmosphere, then quenched with saturated Na_2CO_3 (aq., 2 mL) and extracted with EtOAc (8 mL × 3). The combined organic phases were washed with brine, dried over Na_2SO_4 and concentrated under reduced pressure to give the residue, which was purified by flash column chromatography using PE/EtOAc (10/1, v/v) as the elution to afford the products **3a**–**d**.

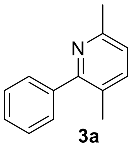

3a

Product **3a** was obtained in 41% overall yield as colorless oil.[11] ^{1}H NMR (400 MHz, $CDCl_3$): δ 7.50-7.33 (m, 6H), 7.04 (d, *J* = 8.0 Hz, 1H), 2.57 (s, 3H), 2.28 (s, 3H); ^{13}C NMR (100 MHz, $CDCl_3$): δ 158.07, 155.45, 141.01, 138.79, 129.01, 128.22, 127.77, 127.49, 121.73, 24.29, 19.62.

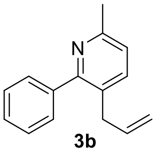

3b

Product **3b** was obtained in 26% overall yield as colorless oil. ^{1}H NMR (400 MHz, $CDCl_3$): δ 7.50-7.34 (m, 6H), 7.09 (d, *J* = 8.0 Hz, 1H), 5.93-5.83 (m, 1H), 5.08-4.92 (m, 1H), 4.97-4.92 (m, 1H), 3.34 (d, *J* = 6.4 Hz, 2H), 2.60 (s, 3H); ^{13}C NMR (100 MHz, $CDCl_3$): δ 158.24, 155.87, 140.66, 138.26, 137.07, 129.45, 128.94, 128.20, 127.86, 121.92, 116.43, 36.49, 24.35; IR (ATR): 1566, 1460, 1441, 1057, 914, 829, 739, 698 cm^{-1}; GC-MS (EI): 209 (M^+, 42), 208 (100), 194 (54), 181 (15), 132 (19), 103 (9), 77 (9); HRMS (EI): Exact mass calcd for $C_{15}H_{15}N$ $[M]^+$: 209.1204, Found: 209.1202.

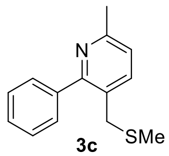

3c

Product **3c** was obtained in 44% overall yield as colorless oil. ^{1}H NMR (400 MHz, $CDCl_3$): δ 7.70 (d, J = 8.0 Hz, 1H), 7.56-7.54 (m, 2H), 7.46-7.37 (m, 3H), 7.13 (d, J = 8.0 Hz, 1H), 3.66 (s, 2H), 2.59 (s, 3H), 1.96 (s, 3H); ^{13}C NMR (100 MHz, $CDCl_3$): δ 158.31, 156.74, 140.17, 138.37, 129.11, 128.33, 128.27, 128.08, 122.05, 35.19, 24.43, 15.59; IR (ATR): 1715, 1599, 1526, 1448, 1323, 1117, 788, 758 cm^{-1}; HRMS (ESI): Exact mass calcd for $C_{14}H_{16}NS$ $[M+H]^+$: 230.0998, Found: 230.0997.

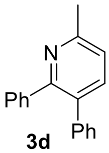

3d

Product **3d** was obtained in 40% overall yield as colorless oil.[12] ^{1}H NMR (400 MHz, $CDCl_3$): δ 7.62 (d, J = 8.0 Hz, 1H), 7.35-7.33 (m, 2H), 7.24-7.14 (m, 9H), 2.66 (s, 3H); ^{13}C NMR (100 MHz, $CDCl_3$): δ 157.18, 156.55, 140.48, 140.11, 138.83, 133.14, 129.99, 129.67, 128.27, 127.96, 127.65, 126.96, 121.79, 24.53.

The synthesis of polysubstituted 3-hydroxyl pyridines 3e–h

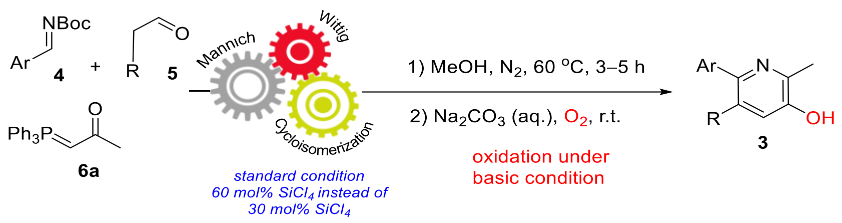

The Mannich/Wittig/cycloisomerization sequence involving *N*-Boc imine **4** (0.5 mmol), aldehyde **5** (1.0 mmol for synthesis of **3e**, 0.5 mmol for synthesis of **3f**–**h**) and phosphorane **6a** (1.0 equiv, based on intermediate **7**) was performed according to the general procedures in the section 4, except using 60 mol% of $SiCl_4$. After the completion of cycloisomerization, anhydrous MeOH (1.0 mL) was added **under N_2 atmosphere** to the reaction mixture at ambient temperature and stirred at 60 °C for 3–5 h till full deprotection of intermediate **2** by TLC analysis. The resulting mixture was treated by saturated Na_2CO_3 (aq., pH = 10, 2 mL), and stirred for further 10 h at ambient temperature under O_2 atmosphere. After extracting with EtOAc (8 mL × 3), the combined organic phases were washed with brine, dried over Na_2SO_4 and concentrated under reduced pressure to give the residue, which was purified by flash column chromatography using PE/acetone (5/1, v/v) as the elution to afford the products **3e**–**h**.

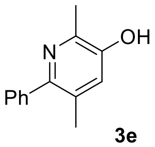

3e

Product **3e** was obtained in 43% overall yield as white solid. Mp: 71-74 °C; ^{1}H NMR (400 MHz, $CDCl_3$): δ 7.45-7.30 (m, 6H), 6.85 (s, 1H), 2.50 (s, 3H), 2.22 (s, 3H); ^{13}C NMR (125 MHz, Acetone-d_6): δ 150.11, 148.76, 143.30, 141.31, 129.21, 128.75, 127.69, 126.90, 123.50, 18.97, 18.18; IR (ATR): 2921, 1581, 1474, 1444, 1353, 1229, 1177, 1140 cm^{-1}; HRMS (ESI): Exact mass calcd for $C_{13}H_{14}ON$ $[M+H]^+$: 200.1070; found: 200.1065. The structure of **3e** was confirmed by the X-ray diffraction analysis (see section 8).

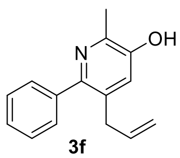

3f

Product **3f** was obtained in 33% overall yield as white solid. Mp: 85-88 °C; ^{1}H NMR (400 MHz, DMSO-d_6): δ 9.77 (s, 1H), 7.41-7.34 (m, 5H), 7.03 (s, 1H), 5.94-5.84 (m, 1H), 5.07 (d, *J* = 10.0 Hz, 1H), 4.97 (dd, *J* = 17.2, 1.6 Hz, 1H), 3.29 (d, *J* = 6.4 Hz, 2H), 2.35 (s, 3H); ^{13}C NMR (100 MHz, DMSO-d_6): δ 150.81, 148.23, 144.00, 140.87, 137.74, 130.84, 129.41, 128.26, 127.54, 122.93, 116.79, 36.49, 19.25; IR (ATR): 1566, 1460, 1441, 993, 914, 829, 739, 700 cm^{-1}; GC-MS (EI): 225 (M^+, 75), 224 (100), 210 (48), 197 (11), 148 (19), 112 (10), 77 (10); HRMS (EI): Exact mass calcd for $C_{15}H_{15}NO$ $[M]^+$: 225.1154, Found: 225.1157.

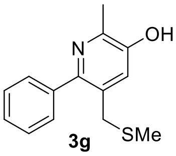

3g

The product **3g** was obtained in 46% overall yield as white solid. Mp: 102-105 °C; ^{1}H NMR (400 MHz, DMSO-d_6): δ 9.87 (s, 1H), 7.49-7.36 (m, 5H), 7.22 (s, 1H), 3.63 (s, 2H), 2.34 (s, 3H), 1.95 (s, 3H); ^{13}C NMR (100 MHz, DMSO-d_6): δ 150.75, 148.20, 144.89, 140.49, 129.86, 129.52, 128.35, 127.70, 122.99, 34.94, 19.32, 15.45; IR (ATR): 1705, 1578, 1508, 1459, 1441, 1176, 849, 758 cm^{-1}; HRMS (ESI): Exact mass calcd for $C_{14}H_{16}NSO$ $[M+H]^+$: 246.0947, Found: 246.0942.

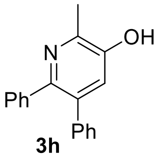

3h

The product **3h** was obtained in 35% overall yield as white solid. Mp: 189-192 °C; ^{1}H NMR (400 MHz, Acetone-d_6): δ 8.78 (s, 1H), 7.30-7.27 (m, 5H), 7.19-7.16 (m, 6H), 2.50 (s, 3H); ^{13}C NMR (100 MHz, Acetone-d_6): δ 151.18, 148.21, 146.14, 141.82, 141.39, 135.40, 130.91, 130.48, 129.24, 128.39, 127.93, 127.67, 124.29, 19.39; IR (ATR): 1587, 1444, 1402, 1325, 1230, 1178, 750, 727 cm^{-1}; GC-MS (EI): 261 (M^+, 40), 260 (55), 213 (54), 212 (100), 211 (36), 157 (24), 44 (82); HRMS (EI): Exact mass calcd for $C_{18}H_{15}ON$ $[M]^+$: 261.1154, Found: 261.1157.

Synthetic utility

Synthesis of chiral *cis*-polysubstituted piperidines 12

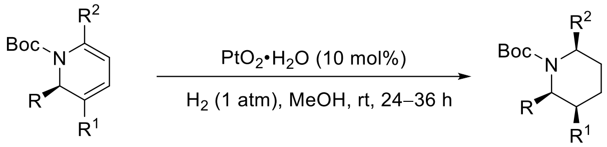

Except 12j and 12q, the synthesis of 12 was achieved by using $PtO_2·H_2O$. To a 10 mL flask was added chiral 1,2-dihydropyridine **2** (0.1 mmol), $PtO_2·H_2O$ (2.5 mg, 0.01 mmol, 10 mol%) and MeOH (2.0 mL), then the flask was in a vacuum and back-filled with H_2 (1 atm). The reaction mixture was stirred vigorously at room temperature for 24–36 h under an atmosphere of H_2 balloon until full conversion of **2** by TLC analysis. The mixture was filtered and concentrated under reduced pressure to give the residue, which was purified by flash column chromatography (PE/Et_2O, 30/1, v/v) to afford the desired piperidines **12**. The dr values was determined by ^{1}H NMR analysis of the crude residue.

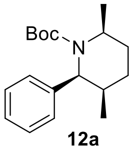

12a

Product **12a** was obtained in 99% yield as colorless oil; >20:1 dr; HPLC analysis (Chiralpak AD-H, ^{i}PrOH/hexane = 0.5/99.5, 1.0 mL/min, 230 nm; t_r (minor) = 5.70 min, t_r (major) = 6.62 min) gave the isomeric composition of the product: >99% ee; $[\alpha]_D^{20}$ = -22.4 (*c* = 0.72, $CHCl_3$); ^{1}H NMR (400 MHz, $CDCl_3$): δ 7.46-7.44 (m, 2H), 7.28-7.18 (m, 3H), 5.13 (d, *J* = 6.8 Hz, 1H), 4.42-4.35 (m, 1H), 2.05-2.01 (m, 1H), 1.87-1.81 (m, 2H), 1.68-1.64 (m, 1H), 1.48-1.46 (m, 1H), 1.38 (s, 9H), 1.14 (d, *J* = 6.8 Hz, 3H), 0.84 (d, *J* = 6.8 Hz, 3H); ^{13}C NMR (100 MHz, $CDCl_3$): δ 155.52, 141.58, 129.43, 127.66, 126.53, 79.43, 59.04, 45.78, 34.58, 31.02, 28.52, 23.48, 21.47, 19.58; IR (ATR): 2972, 1684, 1354, 1312, 1171, 1109, 878, 733, 700 cm^{-1}; HRMS (ESI): Exact mass calcd for $C_{18}H_{27}NNaO_2$ $[M+Na]^+$: 312.1934, Found: 312.1922.

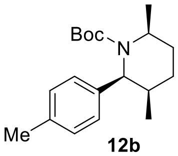

12b

The product **12b** was obtained in 93% yield as colorless oil; >20:1 dr; HPLC analysis (Chiralpak AD-H, ^{i}PrOH/hexane = 0.5/99.5, 1.0 mL/min, 240 nm; t_r (minor) = 5.04 min, t_r (major) = 5.76 min) gave the isomeric composition: 93% ee; $[\alpha]_D^{20}$ = -37.2 (*c* = 0.66, $CHCl_3$); ^{1}H NMR (400 MHz, $CDCl_3$): δ 7.35 (d, *J* = 8.0 Hz, 2H), 7.06 (d, *J* = 8.0 Hz, 2H), 5.11 (d, *J* = 6.4 Hz, 1H), 4.41-4.35 (m, 1H), 2.31 (s, 3H), 2.05-1.97 (m, 1H), 1.92-1.80 (m, 2H), 1.67-1.61 (m, 1H), 1.50-1.45 (m, 1H), 1.40 (s, 9H), 1.11 (d, *J* = 6.8 Hz, 3H), 0.85 (d, *J* = 7.2 Hz, 3H); ^{13}C NMR (100 MHz, $CDCl_3$): δ 155.51, 138.44, 136.01, 129.44, 128.37, 79.37, 58.49, 45.73, 34.65, 31.07, 28.57, 23.56, 21.37, 21.06, 19.62; IR (ATR): 2972, 1683, 1354, 1175, 1109, 910, 752, 735 cm^{-1}; HRMS (ESI): Exact mass calcd for $C_{19}H_{29}NNaO_2$ $[M+Na]^+$: 326.2090, Found: 326.2094.

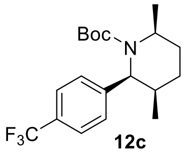

12c

Product **12c** was obtained in 96% yield as colorless oil; >20:1 dr; HPLC analysis (Chiralpak AD-H, ^{i}PrOH/hexane = 0.5/99.5, 1.0 mL/min, 205 nm; t_r (minor) = 5.52 min, t_r (major) = 6.97 min) gave the isomeric composition of the product: 99% ee; $[\alpha]_D^{20}$ = -23.4 (*c* = 0.48, $CHCl_3$); ^{1}H NMR (400 MHz, $CDCl_3$): δ 7.58-7.52 (m, 4H), 5.17 (d, *J* = 6.8 Hz, 1H), 4.42-4.37 (m, 1H), 2.12-2.01 (m, 1H), 1.85-1.66 (m, 3H), 1.52-1.48 (m, 1H), 1.37 (s, 9H), 1.16 (d, *J* = 6.8 Hz, 3H), 0.82 (d, *J* = 7.2 Hz, 3H); ^{13}C NMR (100 MHz, $CDCl_3$): δ 155.40, 145.81, 129.58, 128.84 (q, *J* = 32 Hz), 124.65 (q, *J* = 3 Hz), 124.35 (q, *J* = 270 Hz), 79.80, 58.90, 45.84, 34.27, 30.80, 28.48, 23.33, 21.71, 19.47; ^{19}F NMR (376 MHz, $CDCl_3$): δ -62.43 (s, 3F); IR (ATR): 2974, 1684, 1356, 1323, 1163, 1111, 1068, 735 cm^{-1}; HRMS (ESI): Exact mass calcd for $C_{19}H_{26}F_3NNaO_2$ $[M+Na]^+$: 380.1808, Found: 380.1802.

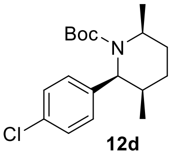

12d

Product **12d** was obtained in 78% yield as colorless oil; >20:1 dr; HPLC analysis (Chiralpak AD-H, ^{i}PrOH/hexane = 0.5/99.5, 1.0 mL/min, 205 nm; t_r (minor) = 5.04 min, t_r (major) = 5.78 min) gave the isomeric composition of the product: >99% ee; $[\alpha]_D^{20}$ = -37.1 (*c* = 0.80, $CHCl_3$); ^{1}H NMR (400 MHz, $CDCl_3$): δ 7.39 (d, *J* = 8.4 Hz, 2H), 7.23 (d, *J* = 8.4 Hz, 2H), 5.09 (d, *J* = 6.8 Hz, 1H), 4.40-4.33 (m, 1H), 2.08-1.97 (m, 1H), 1.87-1.64 (m, 3H), 1.50-1.45 (m, 1H), 1.38 (s, 9H), 1.13 (d, *J* = 6.8 Hz, 3H), 0.82 (d, *J* = 6.8 Hz, 3H); ^{13}C NMR (100 MHz, $CDCl_3$): δ 155.42, 140.16, 132.35, 130.79, 127.86, 79.66, 58.40, 45.75, 34.37, 30.88, 28.54, 23.37, 21.61, 19.52; IR (ATR): 2972, 1684, 1365, 1356, 1173, 1109, 770, 737 cm^{-1}; HRMS (ESI): Exact mass calcd for $C_{18}H_{26}{}^{35}ClNNaO_2$ $[M+Na]^+$: 346.1544, Found: 346.1539.

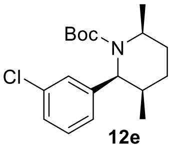

12e

Product **12e** was obtained in 99% yield as colorless oil; >20:1 dr; HPLC analysis (Chiralpak AD-H, ^{i}PrOH/hexane = 0.5/99.5, 1.0 mL/min, 230 nm; t_r (minor) = 5.00 min, t_r (major) = 5.96 min) gave the isomeric composition of the product: >99% ee; $[\alpha]_D^{20}$ = -23.4 (*c* = 0.18, $CHCl_3$); ^{1}H NMR (400 MHz, $CDCl_3$): δ 7.44 (s, 1H), 7.35-7.33 (m, 1H), 7.20-7.19 (m, 2H), 5.07 (d, *J* = 6.8 Hz, 1H), 4.41-4.34 (m, 1H), 2.07-2.00 (m, 1H), 1.83-1.76 (m, 2H), 1.69-1.65 (m, 1H), 1.50-1.47 (m, 1H), 1.39 (s, 9H), 1.16 (d, *J* = 7.2 Hz, 3H), 0.84 (d, *J* = 6.8 Hz, 3H); ^{13}C NMR (100 MHz, $CDCl_3$): δ 155.39, 143.77, 133.57, 129.68, 128.95, 127.43, 126.76, 79.77, 58.82, 45.76, 34.36, 30.84, 28.52, 23.38, 21.60, 19.51; IR (ATR): 2976, 2253, 1690, 1367, 1219, 1176, 912, 737 cm^{-1}; HRMS (ESI): Exact mass calcd for $C_{18}H_{26}{}^{35}ClNNaO_2$ $[M+Na]^+$: 346.1544, Found: 346.1548.

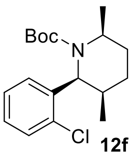

12f

Product **12f** was obtained in 98% yield as colorless oil; 5:1 dr; HPLC analysis (Chiralcel OD-H, ^{i}PrOH/hexane = 0.2/99.8, 0.8 mL/min, 254 nm; t_r (minor) = 9.23 min, t_r (major) = 9.60 min) gave the isomeric composition: 98% ee; $[\alpha]_D^{20}$ = 75.0 (*c* = 0.29, $CHCl_3$); ^{1}H NMR (400 MHz, $CDCl_3$): δ 7.42-7.40 (m, 1H), 7.32-7.30 (m, 1H), 7.22-7.18 (m, 1H), 7.15-7.11 (m, 1H), 5.55 (d, *J* = 6.8 Hz, 1H), 4.36-4.32 (m, 1H), 2.21-2.14 (m, 1H), 1.92-1.83 (m, 1H), 1.69-1.63 (m, 1H), 1.54-1.48 (m, 2H), 1.43 (d, *J* = 6.8 Hz, 3H), 1.22 (s, 9H), 0.71 (d, *J* = 6.8 Hz, 3H); ^{13}C NMR (100 MHz, $CDCl_3$): δ 155.73, 140.52, 134.22, 129.46, 128.49, 127.41, 126.49, 79.68, 57.07, 46.21, 33.16, 29.67, 28.33, 23.58, 23.06, 17.56; IR (ATR): 2972, 1686, 1383, 1354, 1310, 1173, 733 cm^{-1}; HRMS (ESI): Exact mass calcd for $C_{18}H_{26}{}^{35}ClNNaO_2$ $[M+Na]^+$: 346.1544, Found: 346.1539.

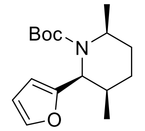

12g

Product **12g** was got in 87% yield as colorless oil; >20:1 dr; HPLC analysis (Chiralpak AD-H, ^{i}PrOH/hexane = 0.5/99.5, 0.5 mL/min, 230 nm; t_r (minor) = 5.52 min, t_r (major) = 6.50 min) gave the ee value: >99%; $[\alpha]_D^{20}$ = 0.8 (*c* = 0.12, $CHCl_3$); ^{1}H NMR (400 MHz, $CDCl_3$): δ 7.32 (s, 1H), 6.29-6.25 (m, 2H), 5.17 (d, *J* = 3.6 Hz, 1H), 4.32-4.27 (m, 1H), 2.09-1.98 (m, 1H), 1.96-1.87 (m, 1H), 1.81-1.72 (m, 1H), 1.63-1.61 (m, 2H), 1.46 (s, 9H), 1.01 (d, *J* = 6.8 Hz, 3H), 0.87 (d, *J* = 6.8 Hz, 3H); ^{13}C NMR (100 MHz, $CDCl_3$): δ 155.11, 154.68, 140.67, 109.97, 109.20, 79.50, 51.57, 45.24, 34.22, 30.89, 28.61, 23.77, 18.63, 18.58; IR (ATR): 2972, 1686, 1391, 1348, 1175, 912, cm^{-1}; HRMS (ESI): Exact mass calcd for $C_{16}H_{25}NNaO_3$ $[M+Na]^+$: 302.1727, Found: 302.1721.

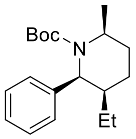

12h

Product **12h** was obtained in 90% yield as colorless oil; 19:1 dr; HPLC analysis (Chiralpak AD-H, ^{i}PrOH/hexane = 0.5/99.5, 1.0 mL/min, 230 nm; t_r (minor) = 6.07 min, t_r (major) = 7.04 min) gave the isomeric composition of the product: 99% ee; $[\alpha]_D^{20}$ = -20.78 (*c* = 0.30, $CHCl_3$); ^{1}H NMR (400 MHz, $CDCl_3$): δ 7.51 (d, *J* = 7.2 Hz, 2H), 7.27-7.18 (m, 3H), 5.25 (d, *J* = 6.0 Hz, 1H), 4.42-4.36 (m, 1H), 1.91-1.71 (m, 3H), 1.70-1.62 (m, 2H), 1.42 (s, 9H), 1.37-1.30 (m, 1H), 1.23-1.14 (m, 1H), 1.05 (d, *J* = 7.2 Hz, 3H), 0.87 (t, *J* = 7.6 Hz, 3H); ^{13}C NMR (100 MHz, $CDCl_3$): δ 155.52, 141.78, 129.67, 127.67, 126.61, 79.47, 57.08, 46.17, 42.20, 31.07, 28.60, 26.61, 21.23, 21.09, 12.21; IR (ATR): 2939, 1680, 1387, 1360, 1313, 1175, 1124, 702 cm^{-1}; HRMS (ESI): Exact mass calcd for $C_{19}H_{29}NNaO_2$ $[M+Na]^+$: 326.2090, Found: 326.2085.

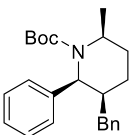

12i

Product **12i** was obtained in 75% yield as colorless oil; 10:1 dr; HPLC analysis (Chiralpak AS-H, ^{i}PrOH/hexane = 0.5/99.5, 1.0 mL/min, 205 nm; t_r (minor) = 10.59 min, t_r (major) = 7.65 min) gave the isomeric composition of the product: >99% ee; $[\alpha]_D^{20}$ = -16.7 (*c* = 0.26, $CHCl_3$); ^{1}H NMR (400 MHz, $CDCl_3$): δ 7.53 (d, *J* = 7.2 Hz, 2H), 7.31-7.16 (m, 6H), 7.03 (d, *J* = 7.2

Hz, 2H), 5.25 (d, *J* = 6.0 Hz, 1H), 4.42-4.35 (m, 1H), 2.64 (dd, *J* = 13.6, 5.2 Hz, 1H), 2.31-2.26 (m, 1H), 2.21-2.13 (m, 1H), 1.96-1.85 (m, 1H), 1.75-1.61 (m, 2H), 1.57-1.50 (m, 1H), 1.38 (s, 9H), 1.10 (d, *J* = 7.2 Hz, 3H); ^{13}C NMR (100 MHz, $CDCl_3$): δ 155.39, 141.53, 140.51, 129.79, 129.08, 129.28, 127.81, 126.80, 126.00, 79.53, 57.59, 46.03, 42.28, 40.22, 30.94, 28.53, 21.20, 20.65; IR (ATR): 2978, 1732, 1695, 1348, 1178, 1092, 910, 756 cm^{-1}; HRMS (ESI): Exact mass calcd for $C_{24}H_{31}NNaO_2$ $[M+Na]^+$: 388.2247, Found: 388.2252.

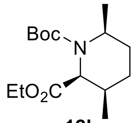

12k

Product **12k** was obtained in 98% yield as colorless oil; 10:1 dr; HPLC analysis (Chiralpak IA, ^{i}PrOH/hexane = 0.5/99.5, 1.0 mL/min, 205 nm; t_r (minor) = 19.29 min, t_r (major) = 12.32 min) gave the isomeric composition of the product: 90% ee; $[\alpha]_D^{25}$ = -4.1 (*c* = 0.5, $CHCl_3$); ^{1}H NMR (400 MHz, $CDCl_3$): δ 4.58 (br s, 1H), 4.28 (br s, 1H), 4.16-4.10 (m, 2H), 1.82-1.79 (m, 1H), 1.72-1.60 (m, 4H), 1.46 (s, 9H), 1.27 (t, *J* = 7.2 Hz, 3H), 1.23 (d, *J* = 6.8 Hz, 3H), 1.07 (d, *J* = 7.2 Hz, 3H); ^{13}C NMR (125 MHz, $CDCl_3$): δ 172.27, 155.94, 79.91, 60.30, 57.94, 46.05, 33.12, 30.75, 28.47, 23.22, 19.27, 18.59, 14.30; IR (ATR): 2978, 2359, 2342, 1734, 1697, 1174, 912, 735 cm^{-1}; GC-MS (EI): 285 (M^+, 0.2), 212 (16), 170 (5), 157 (10), 156 (100), 112 (58), 96 (8), 57 (55); HRMS (EI): Exact mass calcd for $C_{15}H_{27}NO_4$ $[M]^+$: 285.1940, Found: 285.1942.

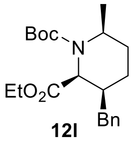

12l

Product **12l** was obtained in 92% yield as colorless oil; >20:1 dr; HPLC analysis (Chiralpak IA, ^{i}PrOH/hexane = 0.5/99.5, 1.0 mL/min, 230 nm; t_r (minor) = 10.62 min, t_r (major) = 7.63 min) gave the isomeric composition of the product: 87% ee; $[\alpha]_D^{25}$ = -6.3 (*c* = 0.5, $CHCl_3$); ^{1}H NMR (400 MHz, $CDCl_3$): δ 7.30-7.26 (m, 2H), 7.21-7.16 (m, 3H), 4.87-4.54 (m, 1H), 4.29 (s, 1H), 4.16 (q, *J* = 7.2 Hz, 2H), 2.99 (dd, *J* = 8.0, 4.0 Hz, 1H), 2.61 (t, *J* = 10.0 Hz, 1H), 1.94-1.85 (m, 1H), 1.78-1.52 (m, 4H), 1.47-1.39 (m, 9H), 1.28 (t, *J* = 7.2 Hz, 3H), 1.17 (d, *J* = 6.8 Hz, 3H); ^{13}C NMR (125 MHz, $CDCl_3$): δ 172.30, 155.08, 140.33, 129.16, 128.44, 126.17, 79.94, 60.51, 55.79, 46.34, 40.95, 39.07, 30.70, 28.42, 20.26, 18.67, 14.24; IR (ATR): 2978, 1695, 1456, 1178, 1055, 1037, 756, 739 cm^{-1}; GC-MS (EI): 361 (M^+, 0.1), 288 (17), 233 (16), 232 (100), 189 (7), 188 (51), 129 (13), 57 (64); HRMS (EI): Exact mass calcd for $C_{21}H_{31}NO_4$ $[M]^+$: 361.2253, Found: 361.2246.

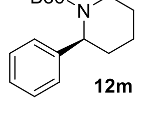

12m

Product **12m** was obtained in 70% yield as colorless oil; >20:1 dr; HPLC analysis (Chiralpak IC, ^{i}PrOH/hexane = 0.5/99.5, 0.5 mL/min, 215 nm; t_r (minor) = 13.52 min, t_r (major) = 12.56 min) gave the isomeric composition of the product: >99% ee; $[\alpha]_D^{20}$ = -99.1 (*c* = 0.31, $CHCl_3$); ^{1}H NMR (400 MHz, $CDCl_3$): δ 7.34-7.26 (m, 4H), 7.21-7.18 (m, 1H), 5.40 (d,

J = 5.6 Hz, 1H), 4.52-4.46 (m, 1H), 2.40-2.37 (m, 1H), 1.87-1.63 (m, 4H), 1.55-1.52 (m, 1H), 1.47 (s, 9H), 0.84 (d, J = 7.2 Hz, 3H); ^{13}C NMR (100 MHz, $CDCl_3$): δ 155.97, 143.39, 128.08, 126.67, 126.26, 79.52, 51.78, 46.53, 30.52, 28.58, 27.39, 20.83, 15.29; IR (ATR): 1715, 1686, 1404, 1391, 1364, 1348, 1176 cm^{-1}; HRMS (ESI): Exact mass calcd for $C_{17}H_{25}NNaO_2$ [M+Na]$^+$: 298.1777, Found: 298.1776.

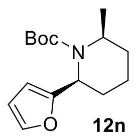

12n

Product **12n** was obtained in 95% yield as colorless oil; >20:1 dr; HPLC analysis (Chiralpak IC, ^{i}PrOH/hexane = 0.5/99.5, 1.0 mL/min, 205 nm; t_r (minor) = 6.94 min, t_r (major) = 6.21 min) gave the isomeric composition of the product: 95% ee; $[\alpha]_D^{20}$ = -35.1 (c = 0.50, $CHCl_3$); ^{1}H NMR (400 MHz, $CDCl_3$): δ 7.31 (s, 1H), 6.30-6.29 (m, 1H), 6.10 (d, J = 3.2 Hz, 1H), 5.36 (d, J = 5.6 Hz, 1H), 4.37-4.31 (m, 1H), 2.22 (d, J = 13.2 Hz, 1H), 1.92-1.65 (m, 3H), 1.55-1.53 (m, 2H), 1.50 (s, 9H), 0.82 (d, J = 7.2 Hz, 3H); ^{13}C NMR (100 MHz, $CDCl_3$): δ 156.23, 155.64, 140.96, 110.29, 105.79, 79.58, 46.71, 46.03, 30.42, 28.59, 26.77, 18.13, 15.32; IR (ATR): 2951, 1690, 1502, 1400, 1364, 1176, 864, 760 cm^{-1}; GC-MS (EI): 265 (M$^+$, 1), 210 (11), 209 (84), 192 (16), 180 (13), 164 (28), 148 (39), 57 (100); HRMS (EI): Exact mass calcd for $C_{15}H_{23}NO_3$ [M]$^+$: 265.1678, Found: 265.1674.

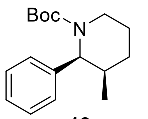

12o

Product **12o** was obtained in 91% yield as colorless oil; >20:1 dr; HPLC analysis (Chiralcel OD-H, ^{i}PrOH/hexane = 0.5/99.5, 1.0 mL/min, 230 nm; t_r (minor) = 6.80 min, t_r (major) = 7.25 min) gave the ee value: 99% ee; $[\alpha]_D^{20}$ = -26.7 (c = 0.30, $CHCl_3$); ^{1}H NMR (400 MHz, $CDCl_3$): δ 7.36-7.34 (m, 2H), 7.30-7.23 (m, 3H), 5.04 (s, 1H), 4.03-3.98 (m, 1H), 3.22-3.15 (m, 1H), 2.10-2.03 (m, 1H), 1.88-1.86 (m, 1H), 1.67-1.58 (m, 3H), 1.35 (s, 9H), 0.76 (d, J = 6.8 Hz, 3H); ^{13}C NMR (100 MHz, $CDCl_3$): δ 155.48, 140.62, 129.29, 127.89, 126.84, 79.49, 59.94, 40.19, 34.20, 28.47, 27.20, 25.38, 19.38; IR (ATR): 2974, 1690, 1391, 1362, 1269, 1176, 1152, 1115 cm^{-1}; HRMS (ESI): Exact mass calcd for $C_{17}H_{25}NNaO_2$ [M+Na]$^+$: 298.1777, Found: 298.1788.

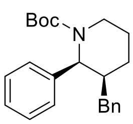

12p

Product **12p** was obtained in 83% yield as colorless oil; 20:1 dr; HPLC analysis (Chiralpak IC, ^{i}PrOH/hexane = 0.5/99.5, 1.0 mL/min, 205 nm; t_r (minor) = 6.37 min, t_r (major) = 7.80 min) gave the isomeric composition of the product: 98% ee; $[\alpha]_D^{20}$ = -22.3 (c = 0.38, $CHCl_3$); ^{1}H NMR (400 MHz, $CDCl_3$): δ 7.41-7.40 (m, 2H), 7.35-7.27 (m, 3H), 7.24-7.22 (m, 2H), 7.18-7.14 (m, 1H), 5.17 (s, 1H), 3.98 (d, J = 12.8 Hz, 1H), 3.16 (td, J = 12.8, 3.6 Hz, 1H), 2.59-2.54 (m, 1H), 2.27-2.19 (m, 1H), 2.13-2.07 (m, 1H), 1.84 (d, J = 12.8 Hz, 1H), 1.69-1.48 (m, 3H), 1.35 (s, 9H); ^{13}C NMR (100 MHz, $CDCl_3$): δ 155.26, 140.36, 140.25, 129.64, 129.14, 128.28, 128.08,

127.15, 126.03, 79.61, 58.91, 41.51, 40.18, 40.03, 28.47, 25.28, 24.50; IR (ATR): 2972, 1686, 1364, 1150, 964, 910, 735, 700 cm^{-1}; HRMS (ESI): Exact mass calcd for $C_{23}H_{29}NNaO_2$ $[M+Na]^+$: 374.2090, Found: 374.2101.

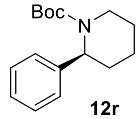
12r

Product **12r** was obtained in 92% yield as colorless oil. HPLC analysis (Chiralcel OJ-H, ^{i}PrOH/hexane = 0.5/99.5, 1.0 mL/min, 215 nm; t_r (minor) = 5.20 min, t_r (major) = 5.71 min) gave the isomeric composition of the product: 99% ee; $[\alpha]_D^{20}$ = -75.6 (*c* = 0.16, $CHCl_3$); ^1H NMR (400 MHz, $CDCl_3$): δ 7.36-7.32 (m, 2H), 7.26-7.21 (m, 3H), 5.41 (d, *J* = 3.6 Hz, 1H), 4.05 (d, *J* = 14.0 Hz, 1H), 2.80-2.73 (m, 1H), 2.33-2.28 (m, 1H), 1.93-1.84 (m, 1H), 1.56-1.40 (m, 13H); ^{13}C NMR (100 MHz, $CDCl_3$): δ 155.77, 140.55, 128.60, 126.60, 126.40, 79.62, 53.35, 40.19, 28.54, 28.20, 25.55, 19.47; IR (ATR): 1690, 1414, 1366, 1178, 1157, 910, 737, 700 cm^{-1}; HRMS (ESI): Exact mass calcd for $C_{16}H_{23}NNaO_2$ $[M+Na]^+$: 284.1621, Found:284.1624.

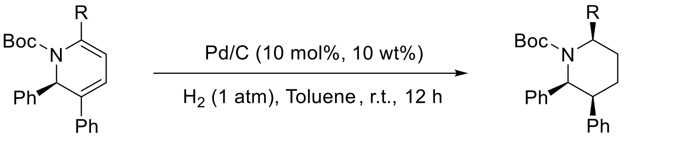

2j or **2q**
12j: R = Me; **12q**: R = H

Procedure for synthesis of 12j and 12q using Pd/C instead of $PtO_2·H_2O$. To a 10 mL flask was added chiral 1,2-dihydropyridine **2** (0.1 mmol) and Pd/C (10% w/w, 10 wt%), followed by the addition of toluene (2.0 mL). After being stirred at room temperature for 12 h under an atmosphere of H_2 balloon till full conversion of **2** by TLC analysis. The mixture was filtered and concentrated under vacuo to give the residue, which was purified by flash column chromatography (PE/Et_2O, 30/1, v/v) to afford **12j** or **12q**. The dr values was determined by ^1H NMR analysis of the crude residue.

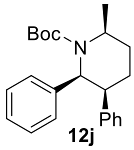
12j

The product **12j** was obtained in 92% yield as colorless oil; >20:1 dr; HPLC analysis (Chiralpak IA, ^{i}PrOH/hexane = 0.5/99.5, 1.0 mL/min, 205 nm; t_r (minor) = 6.37 min, t_r (major) = 6.80 min) gave the isomeric composition of the product: >99% ee; $[\alpha]_D^{20}$ = -39.6 (*c* = 0.04, $CHCl_3$); ^1H NMR (400 MHz, $CDCl_3$): δ 7.25-7.02 (m, 10H), 5.80 (d, *J* = 5.2 Hz, 1H), 4.55-4.49 (m, 1H), 3.31-3.26 (m, 1H), 2.40-2.30 (m, 1H), 2.01-1.90 (m, 2H), 1.80-1.77 (m, 1H), 1.44 (s, 9H), 1.04 (d, *J* = 7.2 Hz, 3H); ^{13}C NMR (100 MHz, $CDCl_3$): δ 155.62, 142.39, 140.98, 128.86, 128.22, 127.39, 127.35, 126.16, 126.11, 79.75, 57.58, 46.12, 45.01, 31.32, 28.55, 21.20, 19.44; IR (ATR): 2971, 1681, 1389, 1348, 1321, 1167, 1076, 722 cm^{-1}; HRMS (ESI): Exact mass calcd for $C_{23}H_{29}NNaO_2$ $[M+Na]^+$: 374.2090, Found: 374.2091.

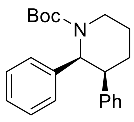
12q

The product **12q** was obtained in 80% yield as colorless oil; 12:1 dr; HPLC analysis (Chiralpak IC, ^{i}PrOH/hexane = 0.5/99.5, 1.0 mL/min, 215 nm; t_r (minor) = 12.18 min, t_r (major) = 13.36 min) gave the isomeric composition of the product: 96% ee; $[\alpha]_D^{20}$ = 64.4 (*c* = 0.38, $CHCl_3$); ^{1}H NMR (400 MHz, $CDCl_3$): δ 7.30-6.91 (m, 10H), 5.68-5.35 (m, 1H), 4.22-4.08 (m, 1H), 3.41-3.12 (m, 2H), 2.17-1.72 (m, 4H), 1.37-1.33 (m, 9H); ^{13}C NMR (100 MHz, $CDCl_3$): δ 155.83, 155.60, 144.02, 142.23, 142.13, 140.06, 128.75, 128.35, 128.34, 128.09, 128.00, 127.96, 127.49, 126.59, 126.43, 126.36, 126.32, 126.27, 79.74, 60.86, 59.20, 58.65, 46.03, 43.47, 40.82, 39.83, 28.39, 26.57, 25.54, 23.33, 20.42; IR (ATR): 2976, 1686, 1452, 1364, 1148, 976, 754, 696 cm^{-1}; HRMS (ESI): Exact mass calcd for $C_{22}H_{27}NNaO_2$ $[M+Na]^+$: 360.1934, Found: 360.1926. (The distinct rotameric isomers were observed in the NMR spectra, due to the presence of sterically bulky Boc group in the product).

Determination of the relative configuration of chiral piperidines

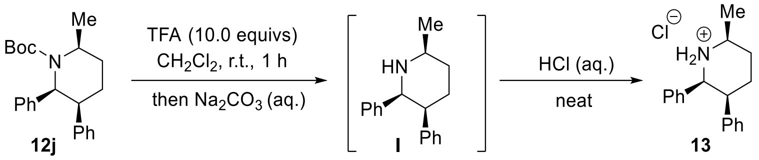

To a 25 mL flask was added chiral piperidine **12j** (35.1 mg, 0.1 mmol) and CH_2Cl_2 (5.0 mL), followed by the addition of TFA (94.0 μL, 1.0 mmol). The resulting mixture was stirred at room temperature for 1 h and quenched by saturated Na_2CO_3 (aq., 5 mL). The aqueous phase was extracted with CH_2Cl_2 (5 mL × 2). Then the combined organic layers were dried over Na_2SO_4 and concentrated under vacuo to afford the intermediate **I**. After treating the crude piperidine **I** with 2 M HCl aqueous solution, the mixture was evaporated to afford analytically pure chiral piperidine hydrochloride **13** (26.1 mg) in 91% yield as a white solid. The structure and relative configuration of **13** was confirmed by its X-ray diffraction analysis (see section 8), thus determining the relative configuration of *N*-Boc piperidine **12** as *cis*-form. Mp: 122-125 °C; ^{1}H NMR (400 MHz, methanol-d_4): δ 7.30-7.25 (m, 3H), 7.19-7.13 (m, 3H), 7.05-7.03 (m, 2H), 6.95-6.92 (m, 2H), 4.85 (d, *J* = 4.8 Hz, 1H), 3.64-3.56 (m, 1H), 3.53-3.51 (m, 1H), 2.48-2.38 (m, 1H), 2.34-2.28 (m, 1H), 2.18-2.12 (m, 2H), 1.51 (d, *J* = 6.4 Hz, 3H); ^{13}C NMR (125 MHz, methanol-d_4): δ 138.56, 136.23, 129.78, 128.68, 128.28, 127.28, 127.10, 64.34, 55.49, 42.90, 28.09, 28.06, 18.39; IR (ATR): 3420, 2251, 1738, 1717, 1456, 1416, 910, 737 cm^{-1}; HRMS (ESI): Exact mass calcd for $C_{18}H_{22}N$ $[M-Cl^-]$: 252.1747, Found: 252.1752.

Synthesis of chiral tetrahydropyridine 14

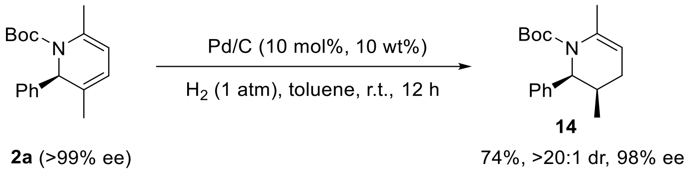

To a 10 mL flask was added chiral 1,2-dihydropyridine **2a** (71.8 mg, 0.24 mmol) and Pd/C (7.2 mg, 10% w/w, 10 wt%), followed by the addition of toluene (2.0 mL). After being stirred at room temperature for 12 h under an atmosphere of H_2 balloon till full conversion of **2a** by TLC analysis. The mixture was filtered and concentrated under vacuo to give the residue, which was purified by flash column chromatography (PE/Et_2O, 30/1, v/v) to afford **14** (51.1 mg) in 74% yield as colorless oil. The ^{1}H NMR analysis of the crude residue revealed that the dr value was >20:1; HPLC analysis (Chiralpak AD-H, ^{i}PrOH/hexane = 0.5/99.5, 1.0 mL/min, 230 nm; t_r (minor) = 6.69 min, t_r (major) = 7.07 min) gave the isomeric composition of the product: 98% ee; $[\alpha]_D^{20}$ = -31.9 (*c* = 0.20, $CHCl_3$); ^{1}H NMR (500 MHz, $CDCl_3$): δ 7.27-7.22 (m, 5H), 5.14 (d, *J* = 5.0 Hz, 1H), 4.86-4.85 (m, 1H), 2.24-2.20 (m, 4H), 2.00-1.94 (m, 1H), 1.68-1.62 (m, 1H), 1.37 (s, 9H), 0.83 (d, *J* = 7.0 Hz, 3H); ^{13}C NMR (125 MHz, $CDCl_3$): δ 153.71, 139.85, 134.97, 128.16, 127.91, 126.97, 109.34, 80.62, 62.24, 31.73, 28.30, 27.74, 23.56, 18.68; IR (ATR): 2972, 1692, 1607, 1454, 1329, 1124, 766, 698 cm^{-1}; GC-MS: 287 (M^+, 18), 231 (100), 216 (10), 187 (26), 172 (51), 170 (35), 118 (46), 57 (92); HRMS (EI): Exact mass calcd for $C_{18}H_{25}NO_2$ $[M]^+$: 287.1885, Found: 287.1887.

Synthesis of chiral bridged rings 15 via Diels-Alder reaction.

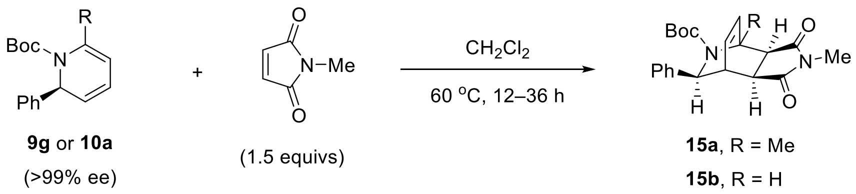

To a 10 mL oven-dried Schlenk tube (with high vacuum valve) were added chiral 1,2-dihydropyridine **9g** or **10a** (0.2 mmol), *N*-Methylmaleimide (33.3 mg, 0.3 mmol) and CH_2Cl_2 (2.0 mL). The reaction mixture was stirred at 60 °C until full conversion of **9g** or **10a** by TLC analysis. The mixture was concentrated under reduced pressure to give the residue, which was purified by flash column chromatography (PE/EtOAc, 4/1, v/v) to afford the desired enantioenriched bridged rings **15a** and **15b**. The dr values was determined by ^{1}H NMR analysis of the crude residue.

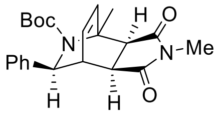

15a

Product **15a** was obtained in 83% yield as yellowish oil. Mp: 176-179 °C; >20:1 dr; HPLC analysis (Chiralpak AD-H, ^{i}PrOH/hexane = 20/80, 1.0 mL/min, 210 nm; t_r (major) = 5.61 min, t_r (minor) = 7.64 min) gave the isomeric composition of the product: >99% ee; $[\alpha]_D^{20}$ = -154.4 (*c* = 0.25, $CHCl_3$); ^{1}H NMR (400 MHz, $CDCl_3$): δ 7.27-7.24 (m, 2H), 7.20-7.17 (m, 1H), 7.11-7.09 (m, 2H), 6.05-6.03 (m, 1H), 5.81-5.78 (m, 1H), 4.85 (d, *J* = 2.8 Hz, 1H), 3.47-3.48 (m, 1H), 3.41 (d, *J* = 8.0 Hz, 1H), 3.25-3.22 (m, 1H), 2.89 (s, 3H), 2.24 (s, 3H), 1.21 (s, 9H); ^{13}C NMR (100 MHz, $CDCl_3$): δ 177.44, 175.56, 155.06, 142.01, 137.86, 130.39, 127.91, 126.78, 126.14, 80.49, 62.71, 56.46, 49.38, 43.22, 39.82, 28.17, 24.82, 23.90; IR (ATR): 2361, 1698, 1331, 1152, 1070, 914, 772, 729, 698 cm^{-1}; HRMS (ESI): Exact mass calcd for $C_{22}H_{26}N_2NaO_4$ $[M+Na]^+$: 405.1785, Found: 405.1779.

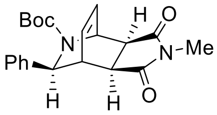

15b

Product **15b** was obtained in 82% yield as white solid. Mp: 183-186 °C; >20:1 dr; HPLC analysis (Chiralpak AD-H, ^{i}PrOH/hexane = 20/80, 1.0 mL/min, 254 nm; t_r (major) = 6.49 min, t_r (minor) = 9.12 min) gave the isomeric composition of the product: >99% ee; $[\alpha]_D^{20}$ = -123.3 (*c* = 0.30, $CHCl_3$); ^{1}H NMR (400 MHz, $CDCl_3$): δ 7.28-7.20 (m, 3H), 7.09-7.07 (m, 2H), 6.54-6.46 (m, 1H), 5.93-5.89 (m, 1H), 5.47-5.26 (m, 1H), 4.82-4.65 (m, 1H), 3.50-3.32 (m, 2H), 3.22-3.20 (m, 1H), 2.89 (s, 3H), 1.49-1.11 (m, 9H); ^{13}C NMR (100 MHz, $CDCl_3$): δ 177.39, 175.58, 154.08, 153.66, 141.66, 140.54, 132.08, 131.63, 131.30, 130.99, 128.18, 127.91, 127.10, 126.16, 125.93, 81.04, 80.52, 59.57, 59.23, 47.68, 46.23, 45.45, 45.13, 42.03, 41.72, 40.73, 28.44, 27.96, 24.79; IR (ATR): 1693, 1674, 1119, 897, 775, 754, 723, 700, 607 cm^{-1}; HRMS (ESI): Exact mass calcd for $C_{21}H_{24}N_2NaO_4$ $[M+Na]^+$: 391.1628, Found: 391.1621. (The distinct rotameric isomers were observed in the NMR spectra, due to the presence of sterically bulky Boc group in the product).

NMR analysis for mechanistic studies

The purpose of NMR experiments was to investigate whether there exists interaction between bulky base 2,6-di-tert-butylpyridine **I** with $SiCl_4$, as well as the acid-base interaction between HCl and Ph_3PO. The ^{19}Si and ^{31}P NMR studies (99 MHz for ^{19}Si; 203 MHz for ^{31}P) were recorded at room temperature in anhydrous $CDCl_3$ from Cambridge Isotope Laboratories, Inc., which was prepared by distillation from CaH_2. The general procedure for ^{19}Si NMR studies was as follows: To four 5 mm NMR tubes containing 0.5 mL $CDCl_3$ were respectively added $SiCl_4$ (36 μL, 0.3 mmol), and the mixture of $SiCl_4$ (36 μL, 0.3 mmol) and 2,6-di-*tert*-butylpyridine **I** with molar ratio of 2:1, 1:1, and 1:2, respectively. After balancing at room temperature for 30 min, the ^{19}Si NMR was recorded at 298K. The general procedure for ^{31}P NMR studies was as follows: In a 5 mm NMR tube containing Ph_3PO (56.0 mg, 0.2 mmol) in 0.5 mL $CDCl_3$ was sequentially added the corresponding amount of HCl (2.0 mol/L EtOAc solution) or different ratio of TMSCl and MeOH for *in-situ* generation of HCl. After balancing at room temperature for 10 min, the ^{31}P NMR study was conducted at 298 K using H_3PO_4 (δ = 0.00 ppm) as the external standard.

^{19}Si NMR analysis

As mentioned in Figure 2B of main text, two possible roles of $SiCl_4$ could be rationalized for the cycloisomerization. One is that it serves as a Lewis acid catalyst, and the other is that it acts as a hidden Brønsted acid, releasing HCl as the true catalyst via hydrolysis.

Supplementary Figure 1. ^{19}Si NMR study of the coordination of 2,6-di-*tert*-butylpyridine **I** to $SiCl_4$.

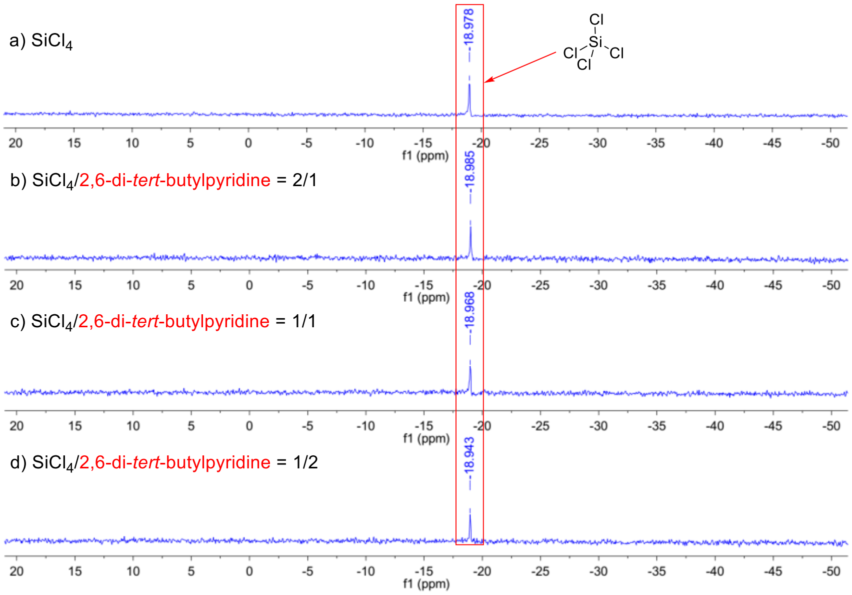

To probe the role of $SiCl_4$, 0.2 or 1.0 equiv noncoordinating bulky base 2,6-di-*tert*-butylpyridine **I** was added to the cycloisomerization of enone **1a**, and found that the reaction was completely inhibited in both cases (Figure 2A). Meanwhile, the coordination of $SiCl_4$ and 2,6-di-*tert*-butylpyridine **I** by the ^{19}Si NMR studies was conducted (Figure S1). At 298 K, the signal of $SiCl_4$ located at 18.978 ppm (a), and the spectra of the 2:1, 1:1, and 1:2 mixture of $SiCl_4$ and **I** showed almost unchanged signal peak, at 18.985, 18.968, and 18.943 ppm, respectively (b-d), which suggested that almost no coordination between $SiCl_4$ and 2,6-di-*tert*-butylpyridine **I**. These results together implied that the HCl generated via the hydrolysis of $SiCl_4$ was the true catalyst.

^{31}P NMR analysis

As illustrated in Figure 2A and 2B, the presence of 1.0 equiv Ph_3PO obviously benefitted the yield of the cycloisomerization. It was postulated that an acid-base interaction between HCl and Ph_3PO modulated the acidity of HCl, and thereby effectively suppressing side reactions.

Supplementary Figure 2. ^{31}P NMR study of the interaction between Ph_3PO and HCl.

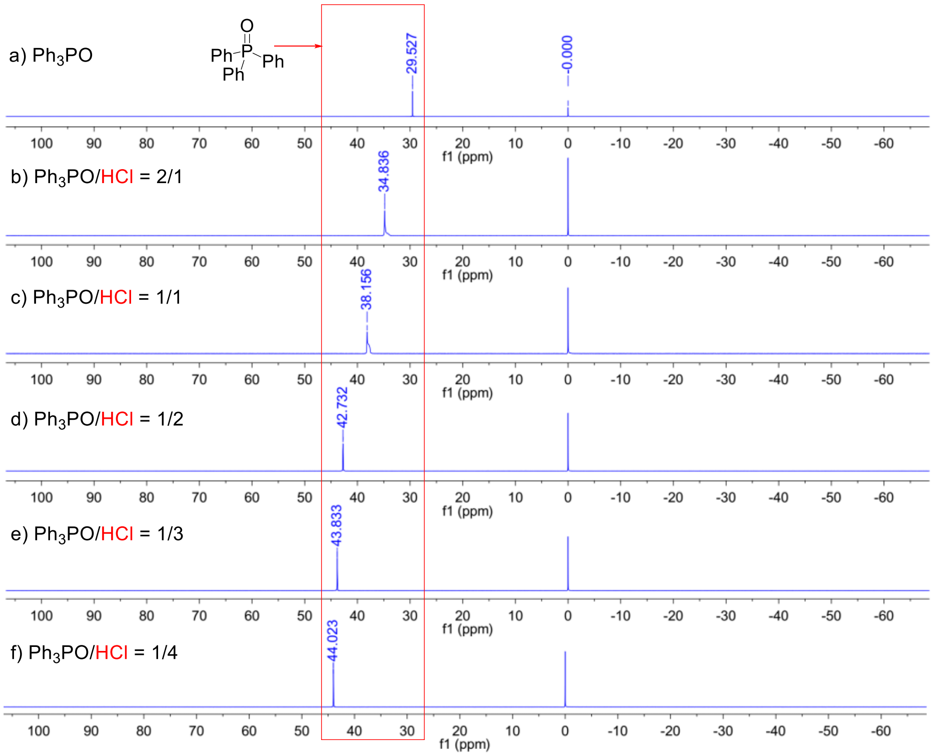

To confirm this speculation, the titration experiments between Ph_3PO and HCl (2.0 mol/L in EtOAc, purchased from Energy Chemical) was performed by using ^{31}P NMR analysis (Figure S2). At 298 K, the signal of free Ph_3PO (55.6 mg, 0.2 mmol) located at 29.527 ppm (a). After adding 0.5 equiv HCl (100 μL), the signal of ^{31}P of Ph_3PO shifted from 29.527 to 34.836 ppm (b). When

increasing the ratio of HCl gradually, the signal of ^{31}P of Ph_3PO further shifted to low field in the spectra of 1:1, 1:2, 1:3, and 1:4 mixture of Ph_3PO and HCl, at 38.156 ppm (c), 42.732 ppm (d), 43.833 ppm (e), and 44.023 ppm (f), respectively.

To further confirm the acid-base interaction between Ph_3PO and in situ generated HCl, freshly distilled TMSCl and anhydrous MeOH were used to *in-situ* generate HCl, instead of using commercial HCl (2.0 mol/L in EtOAc). The ^{31}P NMR analysis (Figure S3) showed the similar tendency. At 298 K, the signal of free Ph_3PO (14 mg, 0.05 mmol) located at 29.645 ppm (a). After adding 2.0 equiv TMSCl (13 μL), the signal of ^{31}P of Ph_3PO shifted from 29.645 to 31.337 ppm (b), due to the Lewis acid of TMSCl. As increasing the ratio of MeOH gradually, the *in-situ* generated HCl was increased. And the signal of ^{31}P of Ph_3PO shifted to low field in the spectra of 1:0.3, 1/0.5, and 1/1 mixture of Ph_3PO and HCl, at 32.907 ppm (c), 34.914 ppm (d), and 36.055 ppm (e), respectively.

Supplementary Figure 3. ^{31}P NMR study of the interaction between Ph_3PO and HCl formed in situ.

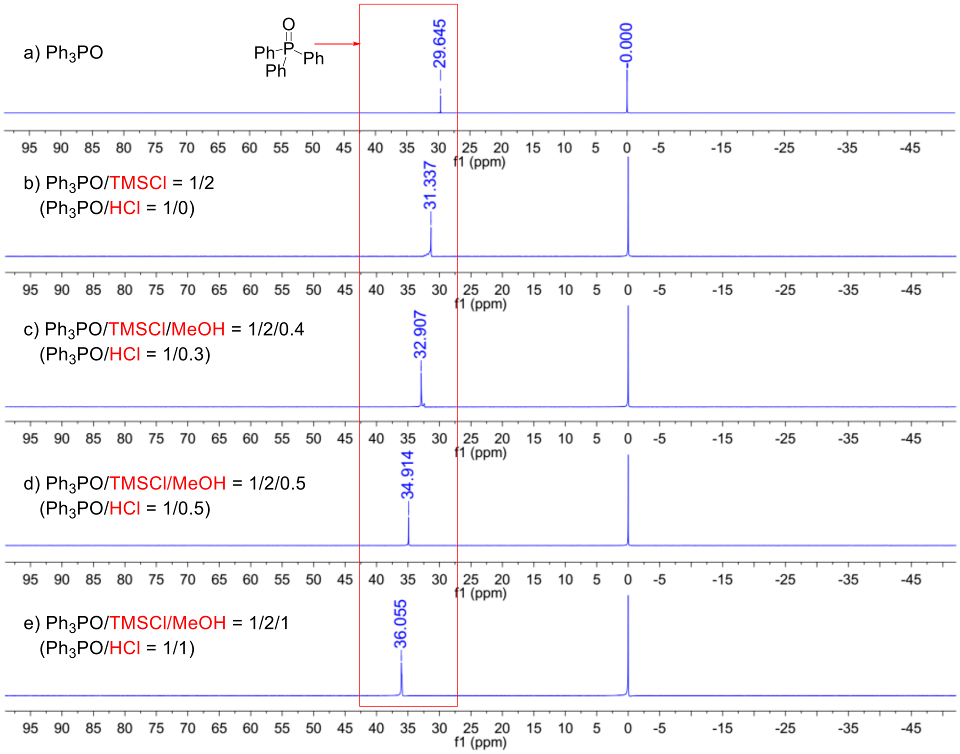

The aforementioned ^{31}P NMR analysis clearly indicated the acid-base interaction between Ph_3PO and HCl, no matter in situ formed or commercial one. This observation, together with the beneficial effect of Ph_3PO in improving the yield, suggested that the presence of Ph_3PO modulated the acidity of HCl to suppress side deprotection to improve the yield.

X-ray crystallographic data of 3e, 13, and 15b

Data intensity of **3e** was collected using a 'Bruker APEX-II CCD' diffractometer at 100.00(10) K. Data collection and reduction were done by using Olex2 and the structure was solved with the ShelXS structure solution program using direct methods and refined by full-matrix least-squares on F^2 with anisotropic displacement parameters for non-H atoms using SHELX-97. Hydrogen atoms were added at their geometrically idea positions and refined isotropically. Crystal data for **3e**: $C_{13}H_{13}NO$, T = 100.00(10) K, triclinic, P-1, a = 10.0210(2) Å, b = 11.6959(2) Å, c = 20.0879(4) Å, α = 84.448(2)°, β = 75.634(2)°, γ = 67.161(2)°, V = 2101.96(8) Å^3. Z = 8, ρ_{calc} = 1.259 g/cm^3. 46291 reflections collected, 7444 [R_{int} = 0.0416, R_{sigma} = 0.0275] independent reflections, R_1 = 0.0540, wR_2 = 0.1570 ($I > 2\sigma(I)$, final), R_1 = 0.0572, wR_2 = 0.1600 (all data), GOF = 1.170, and 553 parameters.

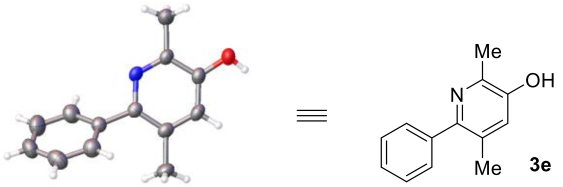

3e

Supplementary Table 3. Crystal data and structure refinement for **3e.**

Identification code	**3e**
Empirical formula	$C_{13}H_{13}NO$
Formula weight	199.24
Temperature/K	100.00(10)
Crystal system	triclinic
Space group	P-1
a/Å	10.0210(2)
b/Å	11.6959(2)
c/Å	20.0879(4)
α/°	84.448(2)
β/°	75.634(2)
γ/°	67.161(2)
Volume/Å^3	2101.96(8)
Z	8

ρ_{calc} (g/cm^3)	1.259
μ/mm^{-1}	0.630
F(000)	848.0
Crystal size/mm^3	0.48 × 0.26 × 0.12
Radiation	CuKα (λ = 1.54184)
2Θ range for data collection/°	8.202 to 134.16
Index ranges	$-11 \leq h \leq 11, -13 \leq k \leq 13, -23 \leq l \leq 23$
Reflections collected	46291
Independent reflections	7444 [R_{int} = 0.0416, R_{sigma} = 0.0275]
Data/restraints/parameters	7444/0/553
Goodness-of-fit on F^2	1.170
Final R indexes [I>=2σ (I)]	R_1 = 0.0540, wR_2 = 0.1570
Final R indexes [all data]	R_1 = 0.0572, wR_2 = 0.1600
Largest diff. peak/hole / e Å^{-3}	0.33/-0.37

Supplementary Table 4. Atomic coordinates (×10^4) and equivalent isotropic displacement parameters (Å^2×10^3) for **3e**. U(eq) is defined as one third of the trace of the orthogonalized Uij tensor.

**Atom**	***x***	***y***	***z***	**U(eq)**
O1	7029(2)	1045(2)	8035.2(12)	20.1(5)
N1	8046(3)	-1567(2)	6946.8(14)	15.4(6)
C1	7651(3)	-3219(2)	6083.2(13)	18.6(6)
C2	8034(3)	-4051(2)	5559.6(14)	20.7(6)
C3	8201(4)	-3654(3)	4878.2(17)	21.8(7)
C4	7980(3)	-2426(3)	4732.0(13)	20.2(6)
C5	7609(3)	-1591(2)	5254.9(13)	18.2(6)
C6	7458(3)	-1991(3)	5943.5(17)	14.1(6)
C7	7156(4)	-1103(3)	6504.3(18)	15.9(6)
C8	6100(3)	76(3)	6570.4(16)	14.2(6)
C9	6034(3)	826(3)	7084.0(17)	16.2(7)
C10	6999(3)	371(3)	7526.0(16)	15.5(6)

**Atom**	***x***	***y***	***z***	**U(eq)**
C11	7992(4)	-855(3)	7454.8(16)	15.4(6)
C12	9042(4)	-1384(3)	7907.5(19)	20.4(7)
C13	4995(4)	605(3)	6115.5(18)	19.1(7)
O2	4957(2)	5973(2)	6894.6(12)	21.5(5)
N2	5025(3)	3437(2)	8041.7(14)	13.7(5)
C14	2864(3)	3414(2)	9742.5(13)	19.8(6)
C15	2716(3)	2571(3)	10262.8(14)	23.2(7)
C16	3095(3)	1335(3)	10113.7(17)	20.0(7)
C17	3589(3)	963(3)	9426.5(14)	22.8(7)
C18	3726(3)	1803(2)	8904.7(13)	18.9(6)
C19	3380(3)	3051(3)	9057.9(18)	16.4(6)
C20	3674(3)	3888(3)	8494.6(17)	13.8(6)
C21	2632(3)	5117(3)	8418.8(18)	17.4(7)
C22	3062(4)	5820(3)	7869.7(17)	16.1(7)
C23	4457(4)	5355(3)	7420.0(16)	14.9(6)
C24	5423(4)	4130(3)	7533.5(17)	14.1(6)
C25	6973(3)	3569(3)	7064.6(17)	17.0(6)
C26	1106(3)	5626(3)	8881.1(18)	19.2(7)
O3	1868(3)	10923(2)	8094.9(12)	21.6(5)
N3	3127(3)	8442(2)	6881.4(14)	15.9(6)
C27	1452(3)	7743(2)	5703.2(13)	20.3(6)
C28	1817(3)	6924(3)	5172.5(14)	23.6(6)
C29	3270(4)	6397(3)	4802.1(18)	23.5(7)
C30	4375(3)	6692(2)	4960.4(13)	20.7(6)
C31	4012(3)	7511(2)	5492.3(13)	18.6(6)
C32	2549(3)	8033(3)	5875.3(17)	15.2(6)
C33	2197(4)	8896(3)	6456.5(17)	15.9(7)
C34	1073(3)	10046(3)	6559.0(16)	13.8(6)
C35	947(4)	10751(3)	7108.8(18)	17.5(7)

Atom	*x*	*y*	*z*	U(eq)
C36	1926(4)	10291(3)	7543.2(17)	17.6(7)
C37	3023(4)	9113(3)	7418.7(16)	16.3(7)
C38	4103(4)	8565(3)	7856.9(18)	19.3(7)
C39	-12(3)	10590(3)	6095.5(18)	18.4(7)
O4	54(2)	15977(2)	6996.9(11)	20.0(5)
N4	64(3)	13395(2)	8101.0(14)	14.6(6)
C40	-485(3)	12470(2)	9507.9(13)	20.1(6)
C41	-668(3)	11675(3)	10045.8(14)	23.1(6)
C42	-1929(4)	11382(3)	10210.1(17)	20.7(7)
C43	-3022(3)	11917(3)	9839.4(14)	24.9(7)
C44	-2849(3)	12727(3)	9303.8(14)	22.9(7)
C45	-1584(3)	13022(3)	9127.8(18)	17.4(7)
C46	-1313(3)	13838(3)	8543.9(17)	14.1(6)
C47	-2366(3)	15051(3)	8461.3(18)	17.6(7)
C48	-1907(4)	15776(3)	7926.5(17)	16.6(7)
C49	-496(4)	15328(3)	7497.0(17)	17.4(7)
C50	456(4)	14097(3)	7604.5(16)	15.1(7)
C51	1998(3)	13539(3)	7121.5(18)	18.5(7)
C52	-3916(3)	15563(3)	8906.0(18)	18.8(7)

Supplementary Table 5. Anisotropic Displacement Parameters ($Å^2 \times 10^3$) for **3e**. The Anisotropic displacement factor exponent takes the form: $-2\pi^2[h^2a^{*2}U_{11}+2hka^*b^*U_{12}+\ldots]$.

Atom	U_{11}	U_{22}	U_{33}	U_{23}	U_{13}	U_{12}
O1	20.4(10)	11.3(10)	26.7(11)	-5.4(9)	-8.9(9)	-0.5(8)
N1	16.5(13)	9.1(12)	18.9(13)	-1.3(10)	-0.3(11)	-5.1(10)
C1	18.1(14)	18.1(14)	19.5(13)	0.9(10)	-4.4(10)	-7.1(11)
C2	19.0(14)	14.7(14)	29.3(15)	-0.7(11)	-6.6(11)	-6.3(11)
C3	25.1(16)	22.5(17)	20.9(16)	-6.7(13)	-4.5(13)	-11.2(14)
C4	17.2(13)	25.6(15)	17.9(13)	-0.2(10)	-3.0(10)	-8.9(12)

Atom	U_{11}	U_{22}	U_{33}	U_{23}	U_{13}	U_{12}
C5	15.6(13)	16.4(14)	22.4(14)	4.0(10)	-6.2(10)	-5.5(11)
C6	12.5(14)	13.4(15)	18.1(15)	1.3(12)	-3.3(12)	-7.0(12)
C7	15.2(14)	16.0(17)	18.2(15)	-0.9(13)	-0.1(13)	-9.6(13)
C8	12.8(14)	17.8(16)	11.8(14)	2.8(13)	-1.9(13)	-6.8(13)
C9	13.9(14)	12.3(15)	20.8(15)	4.1(13)	-4.6(13)	-3.7(12)
C10	17.8(15)	19.5(16)	11.8(13)	0.9(12)	-2.5(12)	-10.6(12)
C11	19.9(16)	13.0(15)	13.0(14)	0.1(12)	-0.9(12)	-7.6(12)
C12	25.9(16)	9.6(15)	24.5(16)	3.5(13)	-6.0(14)	-6.1(13)
C13	18.9(16)	14.2(16)	21.3(16)	1.0(14)	-9.4(14)	-0.4(13)
O2	20.1(10)	16.4(11)	21.7(11)	4.9(9)	-0.3(9)	-4.3(9)
N2	11.3(12)	15.8(13)	13.1(12)	-4.4(10)	-1.4(10)	-4.0(10)
C14	18.5(14)	19.6(15)	21.8(14)	-1.4(10)	-5.8(10)	-6.5(12)
C15	19.5(14)	29.2(16)	20.3(14)	1.8(11)	-2.4(10)	-10.4(12)
C16	13.5(14)	21.5(16)	23.2(16)	6.0(13)	-5.5(12)	-5.3(12)
C17	21.9(14)	17.4(14)	30.1(16)	1.3(11)	-6.5(11)	-8.6(12)
C18	16.8(13)	19.4(15)	21.7(13)	-0.1(10)	-4.3(10)	-8.0(11)
C19	11.0(14)	16.9(15)	19.7(15)	1.6(13)	-5.6(13)	-2.6(12)
C20	12.0(14)	15.2(16)	13.2(13)	-5.6(12)	-2.5(13)	-3.0(12)
C21	16.7(16)	14.2(16)	23.3(16)	-1.4(14)	-7.9(14)	-5.7(13)
C22	17.7(15)	11.7(14)	19.3(15)	2.6(13)	-8.7(13)	-3.9(13)
C23	16.7(14)	9.6(14)	19.7(15)	1.4(12)	-9.9(12)	-3.1(12)
C24	9.2(14)	12.6(15)	20.5(15)	-4.8(12)	-5.4(12)	-1.7(11)
C25	11.1(14)	20.6(16)	13.3(14)	0.1(12)	1.1(12)	-1.9(12)
C26	17.0(16)	20.3(17)	19.3(15)	1.6(14)	-5.0(14)	-5.8(14)
O3	23.1(11)	13.3(10)	25.7(12)	-5.3(9)	-9.6(10)	-0.2(8)
N3	19.8(13)	10.0(12)	16.9(13)	-0.5(10)	-1.7(11)	-5.9(10)
C27	16.6(13)	19.2(14)	23.4(14)	-0.9(10)	-3.6(10)	-5.4(11)
C28	22.9(14)	21.4(15)	29.6(15)	-2.9(11)	-9.8(11)	-8.6(12)
C29	33.7(18)	16.9(16)	21.9(16)	-3.9(12)	-8.0(14)	-9.8(14)

Atom	U_{11}	U_{22}	U_{33}	U_{23}	U_{13}	U_{12}
C30	17.4(13)	17.7(14)	23.9(14)	-1.4(10)	-2.9(10)	-4.1(12)
C31	16.3(14)	16.9(14)	22.2(14)	1.2(10)	-4.6(10)	-6.0(11)
C32	18.3(15)	12.1(15)	16.6(14)	2.7(12)	-4.4(12)	-7.7(12)
C33	15.8(15)	15.7(16)	18.6(15)	0.9(13)	-2.2(13)	-9.9(13)
C34	14.9(14)	14.9(15)	11.3(14)	2.9(13)	-1.2(13)	-7.2(13)
C35	16.8(15)	11.6(15)	23.1(16)	2.2(13)	-5.7(13)	-4.0(12)
C36	20.6(16)	19.8(17)	14.6(14)	-0.6(13)	-4.6(13)	-9.4(13)
C37	23.7(17)	13.9(15)	12.7(14)	0.1(12)	-2.0(13)	-10.0(13)
C38	26.7(17)	10.1(15)	21.0(15)	2.4(12)	-6.1(14)	-7.0(13)
C39	17.4(15)	13.4(16)	22.9(16)	3.1(13)	-8.7(14)	-2.6(13)
O4	20.5(10)	15.1(10)	18.5(11)	3.4(9)	-0.8(9)	-3.4(8)
N4	9.1(12)	17.1(14)	17.3(13)	-3.8(10)	-3.7(11)	-3.4(10)
C40	19.4(14)	19.2(15)	22.1(14)	-0.8(11)	-3.5(10)	-8.3(12)
C41	24.5(15)	22.7(15)	23.5(14)	2.6(11)	-7.7(11)	-9.6(13)
C42	16.6(15)	19.8(16)	21.9(16)	4.1(13)	-4.3(13)	-3.7(12)
C43	22.1(14)	24.3(16)	30.7(15)	3.1(11)	-5.7(11)	-12.2(12)
C44	22.0(14)	20.1(15)	29.0(15)	2.1(11)	-8.6(11)	-9.5(12)
C45	16.0(15)	14.5(15)	19.3(15)	-2.0(13)	-3.5(13)	-3.0(12)
C46	12.6(14)	12.5(16)	15.3(14)	-5.8(12)	-4.5(13)	-0.7(12)
C47	16.0(16)	15.0(16)	22.8(16)	-3.7(14)	-7.5(14)	-4.4(13)
C48	18.8(16)	13.0(15)	18.1(15)	1.6(13)	-8.7(13)	-3.8(13)
C49	19.4(15)	10.9(15)	23.2(15)	0.4(13)	-9.5(14)	-4.5(12)
C50	11.6(14)	13.2(15)	21.2(15)	-4.3(13)	-6.7(13)	-2.7(12)
C51	10.7(14)	19.8(16)	18.9(15)	0.7(13)	-1.1(13)	-0.8(12)
C52	18.8(16)	19.8(17)	19.1(15)	-0.1(14)	-7.4(14)	-7.0(14)

Supplementary Table 6. Bond Lengths for **3e**.

Atom	Atom	Length/Å	Atom	Atom	Length/Å
O1	C10	1.364(4)	O3	C36	1.367(4)

Atom	Atom	Length/Å	Atom	Atom	Length/Å
N1	C7	1.343(4)	N3	C33	1.342(4)
N1	C11	1.356(4)	N3	C37	1.355(4)
C1	C2	1.382(4)	C27	C28	1.386(4)
C1	C6	1.383(4)	C27	C32	1.394(4)
C2	C3	1.393(4)	C28	C29	1.381(5)
C3	C4	1.379(4)	C29	C30	1.393(4)
C4	C5	1.385(4)	C30	C31	1.388(4)
C5	C6	1.407(4)	C31	C32	1.397(4)
C6	C7	1.500(4)	C32	C33	1.501(4)
C7	C8	1.368(4)	C33	C34	1.372(4)
C8	C9	1.391(4)	C34	C35	1.393(4)
C8	C13	1.518(4)	C34	C39	1.518(4)
C9	C10	1.394(4)	C35	C36	1.392(4)
C10	C11	1.389(4)	C36	C37	1.387(5)
C11	C12	1.475(5)	C37	C38	1.477(5)
O2	C23	1.326(4)	O4	C49	1.338(4)
N2	C20	1.366(4)	N4	C46	1.374(4)
N2	C24	1.322(4)	N4	C50	1.312(4)
C14	C15	1.388(4)	C40	C41	1.380(4)
C14	C19	1.389(4)	C40	C45	1.409(4)
C15	C16	1.387(4)	C41	C42	1.391(4)
C16	C17	1.396(4)	C42	C43	1.389(4)
C17	C18	1.384(4)	C43	C44	1.387(4)
C18	C19	1.410(4)	C44	C45	1.394(4)
C19	C20	1.473(4)	C45	C46	1.479(5)
C20	C21	1.432(4)	C46	C47	1.426(4)
C21	C22	1.394(5)	C47	C48	1.402(4)
C21	C26	1.500(4)	C47	C52	1.504(4)
C22	C23	1.394(5)	C48	C49	1.387(5)

Atom	Atom	Length/Å	Atom	Atom	Length/Å
C23	C24	1.418(4)	C49	C50	1.417(4)
C24	C25	1.528(4)	C50	C51	1.533(4)

Supplementary Table 7. Bond Angles for **3e**.

Atom	Atom	Atom	Angle/º	Atom	Atom	Atom	Angle/º
C7	N1	C11	121.1(3)	C33	N3	C37	121.5(3)
C2	C1	C6	121.1(3)	C28	C27	C32	120.4(3)
C1	C2	C3	120.1(3)	C29	C28	C27	120.4(3)
C4	C3	C2	119.5(3)	C28	C29	C30	119.9(3)
C3	C4	C5	120.6(2)	C31	C30	C29	119.8(3)
C4	C5	C6	120.2(2)	C30	C31	C32	120.5(2)
C1	C6	C5	118.6(3)	C27	C32	C31	118.9(3)
C1	C6	C7	121.4(3)	C27	C32	C33	121.8(3)
C5	C6	C7	120.0(3)	C31	C32	C33	119.3(3)
N1	C7	C6	113.5(3)	N3	C33	C32	113.1(3)
N1	C7	C8	121.7(3)	N3	C33	C34	121.4(3)
C8	C7	C6	124.8(3)	C34	C33	C32	125.5(3)
C7	C8	C9	118.1(3)	C33	C34	C35	118.0(3)
C7	C8	C13	123.5(3)	C33	C34	C39	123.1(3)
C9	C8	C13	118.4(3)	C35	C34	C39	118.9(3)
C8	C9	C10	120.4(3)	C36	C35	C34	120.7(3)
O1	C10	C9	124.4(3)	O3	C36	C35	124.1(3)
O1	C10	C11	117.0(3)	O3	C36	C37	117.4(3)
C11	C10	C9	118.6(3)	C37	C36	C35	118.4(3)
N1	C11	C10	119.9(3)	N3	C37	C36	119.9(3)
N1	C11	C12	119.2(3)	N3	C37	C38	118.6(3)
C10	C11	C12	120.8(3)	C36	C37	C38	121.5(3)
C24	N2	C20	121.1(3)	C50	N4	C46	120.6(3)
C15	C14	C19	121.1(3)	C41	C40	C45	120.9(3)

Atom	Atom	Atom	Angle/º	Atom	Atom	Atom	Angle/º
C14	C15	C16	120.8(3)	C40	C41	C42	120.3(3)
C15	C16	C17	118.6(3)	C43	C42	C41	119.5(3)
C18	C17	C16	120.9(3)	C44	C43	C42	120.3(3)
C17	C18	C19	120.5(3)	C43	C44	C45	120.9(3)
C14	C19	C18	118.1(3)	C40	C45	C46	118.6(3)
C14	C19	C20	122.9(3)	C44	C45	C40	118.1(3)
C18	C19	C20	118.9(3)	C44	C45	C46	123.2(3)
N2	C20	C19	116.3(3)	N4	C46	C45	116.2(3)
N2	C20	C21	120.7(3)	N4	C46	C47	121.0(3)
C21	C20	C19	123.0(3)	C47	C46	C45	122.7(3)
C20	C21	C26	122.5(3)	C46	C47	C52	123.5(3)
C22	C21	C20	117.1(3)	C48	C47	C46	116.8(3)
C22	C21	C26	120.3(3)	C48	C47	C52	119.7(3)
C21	C22	C23	121.5(3)	C49	C48	C47	121.4(3)
O2	C23	C22	125.1(3)	O4	C49	C48	125.2(3)
O2	C23	C24	117.2(3)	O4	C49	C50	117.0(3)
C22	C23	C24	117.7(3)	C48	C49	C50	117.7(3)
N2	C24	C23	121.8(3)	N4	C50	C49	122.4(3)
N2	C24	C25	117.8(3)	N4	C50	C51	117.9(3)
C23	C24	C25	120.4(3)	C49	C50	C51	119.7(3)

Supplementary Table 8. Torsion Angles for **3e**.

A	B	C	D	Angle/º
O1	C10	C11	N1	-178.3(3)
O1	C10	C11	C12	-0.9(5)
N1	C7	C8	C9	4.4(5)
N1	C7	C8	C13	-175.3(3)
C1	C2	C3	C4	0.0(4)
C1	C6	C7	N1	45.7(4)

A	B	C	D	Angle/º
C1	C6	C7	C8	-135.1(3)
C2	C1	C6	C5	2.3(4)
C2	C1	C6	C7	-175.3(3)
C2	C3	C4	C5	0.5(4)
C3	C4	C5	C6	0.3(4)
C4	C5	C6	C1	-1.7(4)
C4	C5	C6	C7	176.0(2)
C5	C6	C7	N1	-131.8(3)
C5	C6	C7	C8	47.3(4)
C6	C1	C2	C3	-1.5(4)
C6	C7	C8	C9	-174.7(3)
C6	C7	C8	C13	5.6(5)
C7	N1	C11	C10	0.4(5)
C7	N1	C11	C12	-177.0(3)
C7	C8	C9	C10	-1.5(5)
C8	C9	C10	O1	178.9(3)
C8	C9	C10	C11	-1.9(5)
C9	C10	C11	N1	2.4(5)
C9	C10	C11	C12	179.8(3)
C11	N1	C7	C6	175.2(3)
C11	N1	C7	C8	-3.9(5)
C13	C8	C9	C10	178.3(3)
O2	C23	C24	N2	-179.7(3)
O2	C23	C24	C25	0.3(4)
N2	C20	C21	C22	-0.3(5)
N2	C20	C21	C26	176.7(3)
C14	C15	C16	C17	-1.8(4)
C14	C19	C20	N2	130.0(3)
C14	C19	C20	C21	-49.1(4)

A	B	C	D	Angle/º
C15	C14	C19	C18	1.1(4)
C15	C14	C19	C20	-174.7(3)
C15	C16	C17	C18	1.2(4)
C16	C17	C18	C19	0.6(4)
C17	C18	C19	C14	-1.7(4)
C17	C18	C19	C20	174.3(2)
C18	C19	C20	N2	-45.7(4)
C18	C19	C20	C21	135.2(3)
C19	C14	C15	C16	0.7(4)
C19	C20	C21	C22	178.8(3)
C19	C20	C21	C26	-4.3(5)
C20	N2	C24	C23	-2.0(5)
C20	N2	C24	C25	178.0(2)
C20	C21	C22	C23	-1.0(5)
C21	C22	C23	O2	-178.7(3)
C21	C22	C23	C24	0.9(5)
C22	C23	C24	N2	0.7(5)
C22	C23	C24	C25	-179.3(3)
C24	N2	C20	C19	-177.3(3)
C24	N2	C20	C21	1.8(5)
C26	C21	C22	C23	-178.0(3)
O3	C36	C37	N3	179.6(3)
O3	C36	C37	C38	-1.0(5)
N3	C33	C34	C35	1.8(5)
N3	C33	C34	C39	-179.4(3)
C27	C28	C29	C30	-0.2(5)
C27	C32	C33	N3	131.7(3)
C27	C32	C33	C34	-49.5(5)
C28	C27	C32	C31	1.7(4)

A	B	C	D	Angle/º
C28	C27	C32	C33	-178.7(3)
C28	C29	C30	C31	0.3(4)
C29	C30	C31	C32	0.6(4)
C30	C31	C32	C27	-1.6(4)
C30	C31	C32	C33	178.8(3)
C31	C32	C33	N3	-48.8(4)
C31	C32	C33	C34	130.1(3)
C32	C27	C28	C29	-0.8(4)
C32	C33	C34	C35	-177.0(3)
C32	C33	C34	C39	1.8(5)
C33	N3	C37	C36	0.7(5)
C33	N3	C37	C38	-178.8(3)
C33	C34	C35	C36	-0.7(5)
C34	C35	C36	O3	-179.5(3)
C34	C35	C36	C37	-0.5(5)
C35	C36	C37	N3	0.5(5)
C35	C36	C37	C38	179.9(3)
C37	N3	C33	C32	177.1(3)
C37	N3	C33	C34	-1.8(5)
C39	C34	C35	C36	-179.5(3)
O4	C49	C50	N4	176.6(3)
O4	C49	C50	C51	-4.4(4)
N4	C46	C47	C48	-2.3(4)
N4	C46	C47	C52	176.8(3)
C40	C41	C42	C43	-1.5(4)
C40	C45	C46	N4	46.3(4)
C40	C45	C46	C47	-129.6(3)
C41	C40	C45	C44	-1.0(4)
C41	C40	C45	C46	-178.3(3)

A	B	C	D	Angle/º
C41	C42	C43	C44	0.8(5)
C42	C43	C44	C45	-0.2(4)
C43	C44	C45	C40	0.3(4)
C43	C44	C45	C46	177.4(3)
C44	C45	C46	N4	-130.8(3)
C44	C45	C46	C47	53.3(5)
C45	C40	C41	C42	1.7(4)
C45	C46	C47	C48	173.4(3)
C45	C46	C47	C52	-7.5(5)
C46	N4	C50	C49	0.0(5)
C46	N4	C50	C51	-179.0(2)
C46	C47	C48	C49	0.3(5)
C47	C48	C49	O4	-176.7(3)
C47	C48	C49	C50	1.7(5)
C48	C49	C50	N4	-1.9(5)
C48	C49	C50	C51	177.0(3)
C50	N4	C46	C45	-173.8(3)
C50	N4	C46	C47	2.2(5)
C52	C47	C48	C49	-178.8(3)

Supplementary Table 9. Hydrogen Atom Coordinates (Å×104) and Isotropic Displacement Parameters ($Å^2×10^3$) for **3e**.

Atom	*x*	*y*	*z*	U(eq)
H1	6332.83	1719.31	8068.92	30
H1A	7520.05	-3488.5	6536.99	22
H2	8181.35	-4878.1	5662.21	25
H3	8458.19	-4212.3	4524.78	26
H4	8082.16	-2156.02	4277.99	24
H5	7458.57	-764.44	5150.8	22

H9	5340.72	1637.51	7132.64	19
H12A	8626.85	-1804.36	8285.75	31
H12B	9218.25	-730	8079.03	31
H12C	9964.58	-1962.79	7652.12	31
H13A	5443.35	927.22	5701.36	29
H13B	4126.63	1259.34	6355.13	29
H13C	4716.79	-37.96	6002.38	29
H2A	4437.84	6714.69	6937.29	32
H14	2613.97	4236.77	9854.01	24
H15	2358.04	2837.03	10717.29	28
H16	3021.74	765.96	10463.93	24
H17	3829.79	141.12	9318.08	27
H18	4047.5	1541.22	8449.52	23
H22	2404.51	6616.53	7801.79	19
H25A	7470.64	2738.02	7210.93	25
H25B	7536.05	4061.36	7088.12	25
H25C	6887.61	3556.57	6600.13	25
H26A	810.65	4953.52	9074.93	29
H26B	412.91	6152.46	8620.47	29
H26C	1117.47	6097.23	9243.7	29
H3A	1193.25	11607.85	8118.03	32
H27	469.2	8101.27	5946.13	24
H28	1080.11	6727.51	5065.22	28
H29	3509.7	5845.81	4447.35	28
H30	5353.45	6342.01	4710.42	25
H31	4749.12	7714.31	5594.63	22
H35	200.8	11536.66	7186.36	21
H38A	3687.08	8163.39	8245.09	29
H38B	4322.1	9207.23	8014.28	29
H38C	5001.27	7967.27	7596.19	29

H39A	505.06	10371.48	5626.87	28
H39B	-442.04	11476.47	6141.06	28
H39C	-785.31	10265.03	6225.38	28
H4A	-515.76	16706.25	7022.13	30
H40	375.45	12642.32	9394.88	24
H41	56.46	11334.6	10299.66	28
H42	-2040.47	10830.79	10565.6	25
H43	-3873.99	11731.96	9950.92	30
H44	-3587.62	13078.77	9058.37	27
H48	-2562.24	16573.54	7858.34	20
H51A	2478.3	12688.98	7245.07	28
H51B	2587.68	13998.55	7160.6	28
H51C	1894.15	13579.81	6656.52	28
H52A	-4578.85	15372.71	8703.64	28
H52B	-4237.2	16446.07	8943.37	28
H52C	-3921.88	15196.21	9354.97	28

Data intensity of **13** was collected using a 'Bruker APEX-II CCD' diffractometer at 296(2) K. Data collection and reduction were done by using Olex2 and the structure was solved with the ShelXS structure solution program using direct methods and refined by full-matrix least-squares on F^2 with anisotropic displacement parameters for non-H atoms using SHELX-97. Hydrogen atoms were added at their geometrically idea positions and refined isotropically. Crystal data for **13**: $C_{18}H_{22}ClN$, T = 293.4 K, monoclinic, $P2_1/c$, a = 13.7064(11) Å, b = 7.2752(6) Å, c = 16.9676(15) Å, α = 90°, β = 106.735(3)°, γ = 90°, V = 1620.3(2) Å^3. Z = 4, ρ_{calc} = 1.180 g/cm^3. 14903 reflections collected, 3178 [R_{int} = 0.0496, R_{sigma} = 0.0435] independent reflections, R_1 = 0.0495, wR_2 = 0.1082 ($I > 2\sigma(I)$, final), R_1 = 0.0821, wR_2 = 0.1248 (all data), GOF = 1.062, and 183 parameters.

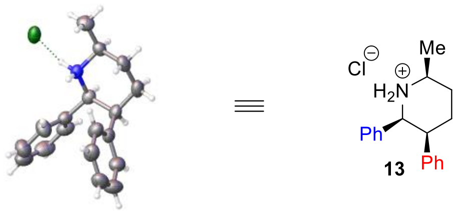

Supplementary Table 10. Crystal data and structure refinement for **13**.

Identification code	**13**
Empirical formula	$C_{18}H_{22}ClN$
Formula weight	287.81
Temperature/K	293.4
Crystal system	monoclinic
Space group	$P2_1/c$
a/Å	13.7064(11)
b/Å	7.2752(6)
c/Å	16.9676(15)
α/°	90
β/°	106.735(3)
γ/°	90
Volume/Å^3	1620.3(2)
Z	4
ρ_{calc}g/cm^3	1.180
μ/mm^{-1}	0.227
F(000)	616.0
Crystal size/mm^3	0.18 × 0.15 × 0.11
Radiation	MoKα (λ = 0.71073)
2Θ range for data collection/°	5.014 to 51.992
Index ranges	$-16 \leq h \leq 16, -8 \leq k \leq 8, -17 \leq l \leq 20$
Reflections collected	14903
Independent reflections	3178 [R_{int} = 0.0496, R_{sigma} = 0.0435]
Data/restraints/parameters	3178/0/183
Goodness-of-fit on F^2	1.062
Final R indexes [I>=2σ (I)]	R_1 = 0.0495, wR_2 = 0.1082
Final R indexes [all data]	R_1 = 0.0821, wR_2 = 0.1248
Largest diff. peak/hole / e Å^{-3}	0.16/-0.21

Supplementary Table 11. Atomic coordinates (×10^4) and equivalent isotropic displacement parameters (Å^2×10^3) for **13**. U(eq) is defined as one third of the trace of the orthogonalized Uij tensor.

**Atom**	***x***	***y***	***z***	**U(eq)**
Cl1	5529.5(5)	2432.7(8)	6816.5(4)	51.5(2)
N1	4245.5(13)	3098(2)	8035.5(11)	39.4(5)
C1	3255.7(17)	2063(3)	7745.0(14)	43.4(6)
C2	2679.7(18)	1940(3)	8406.2(15)	48.3(6)
C3	3419(2)	1346(4)	9237.7(15)	59.1(7)
C4	4409(2)	2426(3)	9481.3(15)	53.8(7)
C5	4946.1(17)	2351(3)	8817.5(14)	43.8(6)
C6	5946.9(19)	3345(4)	9033.1(16)	59.9(7)
C7	2609.0(17)	2741(3)	6921.0(14)	44.5(6)
C8	2135.3(19)	1453(4)	6333.0(17)	58.2(7)
C9	1502(2)	1986(5)	5573.2(18)	72.6(9)
C10	1337(2)	3806(5)	5396.9(18)	73.6(9)
C11	1804(2)	5092(5)	5962.1(18)	74.2(9)
C12	2436(2)	4572(4)	6721.2(16)	63.5(7)
C13	2014.4(17)	3555(3)	8470.4(14)	46.3(6)
C14	974.9(19)	3273(4)	8291.3(16)	60.7(7)
C15	318(2)	4663(5)	8368.3(19)	76.5(9)
C16	687(2)	6367(5)	8623.6(18)	71.8(8)
C17	1713(2)	6691(4)	8806.2(18)	66.2(8)
C18	2369(2)	5304(3)	8731.3(17)	57.4(7)

Supplementary Table 12. Anisotropic Displacement Parameters (Å^2×10^3) for **13**. The Anisotropic displacement factor exponent takes the form: $-2\pi^2[h^2a^{*2}U_{11}+2hka^*b^*U_{12}+\ldots]$.

**Atom**	**U_{11}**	**U_{22}**	**U_{33}**	**U_{23}**	**U_{13}**	**U_{12}**
Cl1	70.8(4)	39.0(3)	53.8(4)	2.7(3)	32.2(3)	4.7(3)
N1	47.1(11)	35.8(9)	39.2(11)	-2.3(8)	18.6(9)	0.4(8)
C1	48.3(14)	37.4(12)	47.4(14)	-6.1(10)	18.1(11)	-3.5(10)
C2	53.9(15)	41.7(13)	55.1(16)	2.9(11)	25.1(12)	-5.1(11)

Atom	U_{11}	U_{22}	U_{33}	U_{23}	U_{13}	U_{12}
C3	73.8(18)	56.2(16)	58.2(16)	16.7(13)	36.3(14)	13.9(14)
C4	67.7(17)	57.2(15)	38.5(14)	6.4(12)	18.7(12)	13.9(14)
C5	49.6(13)	40.4(12)	40.7(13)	0.9(10)	12.1(11)	8.2(11)
C6	54.9(16)	64.2(17)	55.0(16)	-9.0(14)	6.8(13)	-0.2(14)
C7	41.2(13)	53.1(14)	41.3(13)	-6.2(11)	15.2(11)	-1.6(11)
C8	48.2(14)	64.9(17)	63.7(18)	-14.8(14)	19.6(14)	-1.8(13)
C9	53.2(17)	107(3)	54.1(19)	-26.6(17)	9.5(14)	-8.4(18)
C10	49.4(16)	119(3)	51.2(18)	1.7(19)	12.2(14)	5.6(19)
C11	77(2)	83(2)	57.4(19)	12.4(17)	11.3(16)	5.5(17)
C12	75.5(19)	60.4(17)	48.4(16)	-0.2(13)	8.0(14)	-2.5(15)
C13	48.6(14)	53.0(15)	41.7(13)	6.9(11)	19.8(11)	2.6(12)
C14	50.3(16)	76.1(18)	53.8(17)	0.0(14)	11.7(13)	-1.1(15)
C15	46.9(17)	100(3)	78(2)	-0.6(18)	11.7(15)	12.5(17)
C16	68(2)	80(2)	71(2)	11.4(17)	26.2(16)	27.2(18)
C17	75(2)	55.6(16)	77(2)	5.7(15)	36.0(16)	9.6(15)
C18	54.0(15)	51.4(15)	73.6(19)	2.3(13)	29.2(14)	2.8(13)

Supplementary Table 13. Bond Lengths for **13**.

Atom	Atom	Length/Å	Atom	Atom	Length/Å
N1	C1	1.505(3)	C8	C9	1.386(4)
N1	C5	1.498(3)	C9	C10	1.362(4)
C1	C2	1.549(3)	C10	C11	1.361(4)
C1	C7	1.507(3)	C11	C12	1.382(4)
C2	C3	1.543(3)	C13	C14	1.384(3)
C2	C13	1.511(3)	C13	C18	1.388(3)
C3	C4	1.519(4)	C14	C15	1.385(4)
C4	C5	1.513(3)	C15	C16	1.362(4)
C5	C6	1.500(3)	C16	C17	1.371(4)
C7	C8	1.386(3)	C17	C18	1.382(3)

Atom	Atom	Length/Å	Atom	Atom	Length/Å
C7	C12	1.378(3)			

Supplementary Table 14. Bond Angles for **13**.

Atom	Atom	Atom	Angle/º	Atom	Atom	Atom	Angle/º
C5	N1	C1	113.31(17)	C12	C7	C8	117.7(2)
N1	C1	C2	113.02(18)	C9	C8	C7	121.2(3)
N1	C1	C7	111.96(18)	C10	C9	C8	119.7(3)
C7	C1	C2	113.86(19)	C11	C10	C9	119.9(3)
C3	C2	C1	109.93(19)	C10	C11	C12	120.7(3)
C13	C2	C1	116.83(19)	C7	C12	C11	120.7(3)
C13	C2	C3	113.77(19)	C14	C13	C2	118.2(2)
C4	C3	C2	113.8(2)	C14	C13	C18	116.8(2)
C5	C4	C3	111.5(2)	C18	C13	C2	124.9(2)
N1	C5	C4	108.72(18)	C13	C14	C15	121.8(3)
N1	C5	C6	110.42(19)	C16	C15	C14	120.2(3)
C6	C5	C4	114.0(2)	C15	C16	C17	119.4(3)
C8	C7	C1	118.4(2)	C16	C17	C18	120.4(3)
C12	C7	C1	123.9(2)	C17	C18	C13	121.4(3)

Supplementary Table 15. Torsion Angles for **13**.

A	B	C	D	Angle/º
N1	C1	C2	C3	-47.4(3)
N1	C1	C2	C13	84.1(2)
N1	C1	C7	C8	136.0(2)
N1	C1	C7	C12	-45.7(3)
C1	N1	C5	C4	-58.8(2)
C1	N1	C5	C6	175.38(18)
C1	C2	C3	C4	48.3(3)
C1	C2	C13	C14	115.9(2)
C1	C2	C13	C18	-67.0(3)

A	B	C	D	Angle/º
C1	C7	C8	C9	177.7(2)
C1	C7	C12	C11	-177.7(2)
C2	C1	C7	C8	-94.2(2)
C2	C1	C7	C12	84.0(3)
C2	C3	C4	C5	-55.1(3)
C2	C13	C14	C15	177.3(2)
C2	C13	C18	C17	-177.2(2)
C3	C2	C13	C14	-114.3(2)
C3	C2	C13	C18	62.8(3)
C3	C4	C5	N1	58.1(3)
C3	C4	C5	C6	-178.2(2)
C5	N1	C1	C2	54.8(2)
C5	N1	C1	C7	-174.97(17)
C7	C1	C2	C3	-176.66(19)
C7	C1	C2	C13	-45.1(3)
C7	C8	C9	C10	-0.1(4)
C8	C7	C12	C11	0.5(4)
C8	C9	C10	C11	0.9(4)
C9	C10	C11	C12	-1.0(5)
C10	C11	C12	C7	0.2(4)
C12	C7	C8	C9	-0.6(4)
C13	C2	C3	C4	-84.9(2)
C13	C14	C15	C16	0.2(4)
C14	C13	C18	C17	-0.1(4)
C14	C15	C16	C17	-0.2(5)
C15	C16	C17	C18	0.1(4)
C16	C17	C18	C13	0.1(4)
C18	C13	C14	C15	0.0(4)

Supplementary Table 16. Hydrogen Atom Coordinates (Å×10^4) and Isotropic Displacement Parameters (Å^2×10^3) for **13**.

**Atom**	***x***	***y***	***z***	**U(eq)**
H1A	4588	3055	7609	47
H1B	4100	4377	8120	47
H1	3442	797	7653	52
H2	2209	905	8235	58
H3A	3081	1490	9664	71
H3B	3576	52	9209	71
H4A	4266	3697	9581	65
H4B	4854	1927	9988	65
H5	5077	1057	8724	53
H6A	6248	3223	8590	90
H6B	6395	2826	9525	90
H6C	5839	4623	9122	90
H8	2245	209	6450	70
H9	1191	1104	5185	87
H10	905	4170	4891	88
H11	1698	6333	5836	89
H12	2748	5468	7102	76
H14	710	2118	8114	73
H15	-377	4431	8245	92
H16	246	7303	8674	86
H17	1970	7852	8982	79
H18	3063	5547	8858	69

Data intensity of **15b** was collected using a 'Bruker APEX-II CCD' diffractometer at 100.00(10) K. Data collection and reduction were done by using Olex2 and the structure was solved with the ShelXS structure solution program using direct methods and refined by full-matrix least-squares on F2 with anisotropic displacement parameters for non-H atoms using SHELX-97. Hydrogen atoms were added at their geometrically idea positions and refined isotropically. Crystal data for **15b**:

$C_{18}H_{22}ClN$, T = 100.00(10) K, orthorhombic, $P2_12_12_1$, a = 6.31030(10) Å, b = 11.60320(10) Å, c = 25.2401(2) Å, α = 90°, β = 90°, γ = 90°, V = 1848.07(4) Å^3. Z = 4, ρcalc = 1.324 g/cm^3. 17057 reflections collected, 3301 [Rint = 0.0233, Rsigma = 0.0154] independent reflections, R_1 = 0.0232, wR_2 = 0.0587 (I > 2σ(I), final), R_1 = 0.0234, wR_2 = 0.0588 (all data), GOF = 1.062, and 183 parameters.

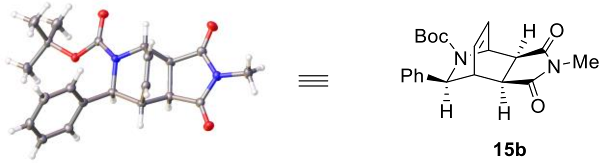

15b

Supplementary Table 17. Crystal data and structure refinement for **15b**.

Identification code	**15b**
Empirical formula	$C_{21}H_{24}N_2O_4$
Formula weight	368.42
Temperature/K	100.00(10)
Crystal system	orthorhombic
Space group	$P2_12_12_1$
a/Å	6.31030(10)
b/Å	11.60320(10)
c/Å	25.2401(2)
α/°	90
β/°	90
γ/°	90
Volume/Å^3	1848.07(4)
Z	4
ρ_{calc}g/cm^3	1.324
μ/mm^{-1}	0.750
F(000)	784.0
Crystal size/mm^3	0.36 × 0.34 × 0.28
Radiation	CuKα (λ = 1.54184)
2Θ range for data collection/°	7.004 to 134.13

Index ranges	-7≤h≤6, -13≤k≤13, -30≤l≤28
Reflections collected	17057
Independent reflections	3301 [Rint = 0.0233, Rsigma = 0.0154]
Data/restraints/parameters	3301/0/249
Goodness-of-fit on F^2	1.058
Final R indexes [I>=2σ (I)]	R_1 = 0.0232, wR_2 = 0.0587
Final R indexes [all data]	R_1 = 0.0234, wR_2 = 0.0588
Largest diff. peak/hole / e $Å^{-3}$	0.18/-0.12

Supplementary Table 18. Fractional atomic coordinates ($\times10^4$) and equivalent isotropic displacement parameters ($Å^2\times10^3$) for **15b**. U(eq) is defined as one third of the trace of the orthogonalized Uij tensor.

**Atom**	***x***	***y***	***z***	**U(eq)**
C1	6295(3)	7027.6(15)	6028.2(6)	21.1(3)
C2	6859(3)	7973.3(15)	6334.2(6)	23.9(4)
C3	8875(3)	8047.0(14)	6550.8(6)	23.6(4)
C4	10332(3)	7176.5(15)	6456.6(6)	23.2(3)
C5	9775(2)	6231.7(14)	6149.5(6)	19.9(3)
C6	7747(2)	6149.6(13)	5931.4(6)	16.8(3)
C7	7230(2)	5110.8(13)	5591.0(6)	16.7(3)
C8	7446(3)	5382.2(13)	4986.6(6)	16.5(3)
C9	7256(2)	4217.7(13)	4688.0(6)	17.3(3)
C10	5136(3)	3647.3(13)	4834.9(6)	18.8(3)
C11	3839(2)	4504.9(15)	5173.0(6)	19.1(3)
C12	5617(3)	6119.0(14)	4806.1(6)	19.6(3)
C13	3736(3)	5651.7(15)	4892.2(6)	21.2(3)
C14	4397(2)	4123.5(13)	6112.3(6)	17.0(3)
C15	5284(3)	4040.6(14)	7054.6(6)	20.6(3)
C16	4678(3)	2789.3(15)	7149.7(7)	27.0(4)
C17	3519(3)	4866.4(16)	7206.9(7)	30.0(4)

Atom	*x*	*y*	*z*	U(eq)
C18	7318(3)	4343.4(17)	7342.2(6)	30.3(4)
C19	7153(3)	4357.3(13)	4090.6(6)	19.0(3)
C20	4073(3)	3392.2(13)	4313.4(6)	19.8(3)
C21	4706(3)	3732.2(15)	3360.0(6)	24.6(4)
N1	5051(2)	4674.8(11)	5665.1(5)	17.7(3)
N2	5319(2)	3830.3(11)	3912.4(5)	18.8(3)
O1	5863.8(17)	4234.3(9)	6495.9(4)	18.7(2)
O2	2693.5(17)	3636.9(9)	6154.1(4)	21.7(2)
O3	8417.6(19)	4842.7(11)	3806.2(5)	27.2(3)
O4	2389.2(19)	2910.7(11)	4244.3(4)	27.3(3)

Supplementary Table 19. Anisotropic Displacement Parameters ($Å^2 \times 10^3$) for **15b**. The Anisotropic displacement factor exponent takes the form: $-2\pi^2[h^2a^{*2}U_{11}+2hka^*b^*U_{12}+\ldots]$.

Atom	U_{11}	U_{22}	U_{33}	U_{23}	U_{13}	U_{12}
C1	18.3(8)	25.5(8)	19.4(7)	2.2(7)	-0.8(6)	2.4(7)
C2	28.9(9)	22.1(8)	20.6(8)	0.4(7)	3.3(7)	6.7(7)
C3	32.1(9)	19.3(8)	19.4(8)	-2.4(6)	0.4(7)	-1.7(7)
C4	20.5(8)	26.1(8)	22.9(8)	-0.4(7)	-3.1(7)	-1.4(7)
C5	18.8(8)	21.2(8)	19.7(7)	0.7(6)	-0.4(6)	2.2(6)
C6	18.5(7)	18.6(7)	13.3(7)	4.6(6)	1.1(6)	-0.7(6)
C7	13.2(7)	18.7(7)	18.3(8)	2.1(6)	-0.7(6)	0.2(6)
C8	14.6(7)	18.0(7)	16.8(7)	0.8(6)	0.8(6)	-2.2(6)
C9	16.3(7)	17.8(7)	17.9(7)	1.1(6)	-1.1(6)	1.1(7)
C10	19.6(8)	18.8(7)	18.2(7)	4.3(6)	-2.8(6)	-3.9(7)
C11	13.8(7)	27.4(8)	16.0(7)	3.3(6)	-1.7(6)	-3.2(7)
C12	24.1(8)	19.1(8)	15.5(7)	2.1(6)	0.1(6)	1.4(7)
C13	20.2(8)	25.8(8)	17.5(7)	2.1(7)	-0.9(6)	5.5(7)
C14	16.9(7)	18.3(7)	15.8(7)	-1.3(6)	0.9(6)	2.7(6)
C15	25.8(8)	23.6(8)	12.5(7)	1.0(6)	2.4(6)	0.2(7)

Atom	U_{11}	U_{22}	U_{33}	U_{23}	U_{13}	U_{12}
C16	34.3(10)	25.4(9)	21.2(8)	5.2(7)	3.2(7)	1.3(8)
C17	37.9(11)	29.7(9)	22.5(8)	-1.6(7)	7.2(8)	8.0(8)
C18	33.3(10)	41.3(10)	16.4(8)	-0.4(7)	-3.8(7)	-5.1(9)
C19	20.0(8)	17.6(7)	19.4(7)	-1.3(6)	1.6(6)	0.5(7)
C20	22.0(8)	17.8(7)	19.6(8)	3.6(6)	-1.3(7)	-2.5(6)
C21	32.2(9)	26.3(8)	15.4(8)	-0.3(6)	-1.7(7)	-4.8(7)
N1	15.1(6)	22.8(6)	15.1(6)	2.3(5)	-1.1(5)	-3.3(5)
N2	21.2(7)	19.0(6)	16.2(6)	1.0(5)	-1.4(5)	-2.1(5)
O1	18.7(5)	24.3(6)	13.2(5)	1.0(4)	0.1(4)	-1.5(5)
O2	18.7(6)	25.8(6)	20.7(5)	3.1(5)	1.4(5)	-4.3(5)
O3	27.5(6)	31.3(7)	22.9(6)	-2.0(5)	7.2(5)	-10.3(5)
O4	25.7(6)	33.4(6)	22.8(6)	2.2(5)	-3.7(5)	-12.9(6)

Supplementary Table 20. Bond Lengths for **15b**.

Atom	Atom	Length/Å	Atom	Atom	Length/Å
C1	C2	1.388(2)	C11	C13	1.509(2)
C1	C6	1.392(2)	C11	N1	1.4718(19)
C2	C3	1.387(2)	C12	C13	1.323(2)
C3	C4	1.387(2)	C14	N1	1.361(2)
C4	C5	1.388(2)	C14	O1	1.3457(19)
C5	C6	1.397(2)	C14	O2	1.2189(19)
C6	C7	1.516(2)	C15	C16	1.521(2)
C7	C8	1.564(2)	C15	C17	1.519(2)
C7	N1	1.4771(19)	C15	C18	1.516(2)
C8	C9	1.552(2)	C15	O1	1.4739(17)
C8	C12	1.507(2)	C19	N2	1.384(2)
C9	C10	1.538(2)	C19	O3	1.2120(19)
C9	C19	1.518(2)	C20	N2	1.379(2)
C10	C11	1.546(2)	C20	O4	1.213(2)
C10	C20	1.507(2)	C21	N2	1.451(2)

Supplementary Table 21. Bond Angles for **15b**.

Atom	Atom	Atom	Angle/°	Atom	Atom	Atom	Angle/°
C2	C1	C6	120.49(16)	C12	C13	C11	113.58(14)
C3	C2	C1	120.21(16)	O1	C14	N1	110.09(13)
C4	C3	C2	119.72(16)	O2	C14	N1	123.82(14)
C3	C4	C5	120.21(16)	O2	C14	O1	126.06(14)
C4	C5	C6	120.41(15)	C17	C15	C16	112.21(15)
C1	C6	C5	118.96(15)	C18	C15	C16	111.01(14)
C1	C6	C7	122.69(14)	C18	C15	C17	110.71(14)
C5	C6	C7	118.35(14)	O1	C15	C16	111.04(13)
C6	C7	C8	111.97(12)	O1	C15	C17	109.15(13)
N1	C7	C6	113.64(12)	O1	C15	C18	102.27(13)
N1	C7	C8	105.88(12)	N2	C19	C9	108.14(13)
C9	C8	C7	106.96(12)	O3	C19	C9	127.58(15)
C12	C8	C7	110.02(12)	O3	C19	N2	124.28(15)
C12	C8	C9	106.74(12)	N2	C20	C10	108.37(13)
C10	C9	C8	108.97(12)	O4	C20	C10	127.30(15)
C19	C9	C8	113.13(12)	O4	C20	N2	124.31(15)
C19	C9	C10	104.38(12)	C11	N1	C7	115.02(12)
C9	C10	C11	108.47(12)	C14	N1	C7	123.22(12)
C20	C10	C9	105.14(12)	C14	N1	C11	118.63(12)
C20	C10	C11	111.90(13)	C19	N2	C21	124.74(13)
C13	C11	C10	109.34(12)	C20	N2	C19	113.65(13)
N1	C11	C10	106.07(12)	C20	N2	C21	121.61(13)
N1	C11	C13	107.49(13)	C14	O1	C15	120.21(12)
C13	C12	C8	113.89(14)				

Supplementary Table 22. Hydrogen Atom Coordinates (Å×10^4) and Isotropic Displacement Parameters (Å^2×10^3) for **15b**.

Atom	*x*	*y*	*z*	U(eq)
H1	4936.53	6980.56	5886.84	25
H2	5882.66	8559.24	6394.19	29

H3	9247.2	8677.57	6758.32	28
H4	11687.6	7225.99	6599.57	28
H5	10758.76	5649.63	6088.56	24
H7	8223.82	4491.39	5680.03	20
H8	8803.75	5756.9	4909.97	20
H9	8436.71	3710.81	4784.48	21
H10	5383.14	2932.37	5032.21	23
H11	2416.65	4205.85	5247.05	23
H12	5794.8	6837.1	4648.57	23
H13	2470.93	5996.96	4789.49	25
H16A	4560.66	2651.78	7523.59	40
H16B	5747.74	2294.65	7003.27	40
H16C	3343.32	2631.42	6982.24	40
H17A	2243.3	4657.41	7023.23	45
H17B	3915.91	5637.72	7111.6	45
H17C	3281.65	4826.05	7582.13	45
H18A	7099.1	4282.01	7717.55	46
H18B	7724.1	5118.03	7255.33	46
H18C	8420.1	3821.75	7235.96	46
H21A	5731.97	4117.75	3142.29	37
H21B	3340.63	4080.78	3309.76	37
H21C	4638.33	2933.24	3262.7	37

NMR spectra

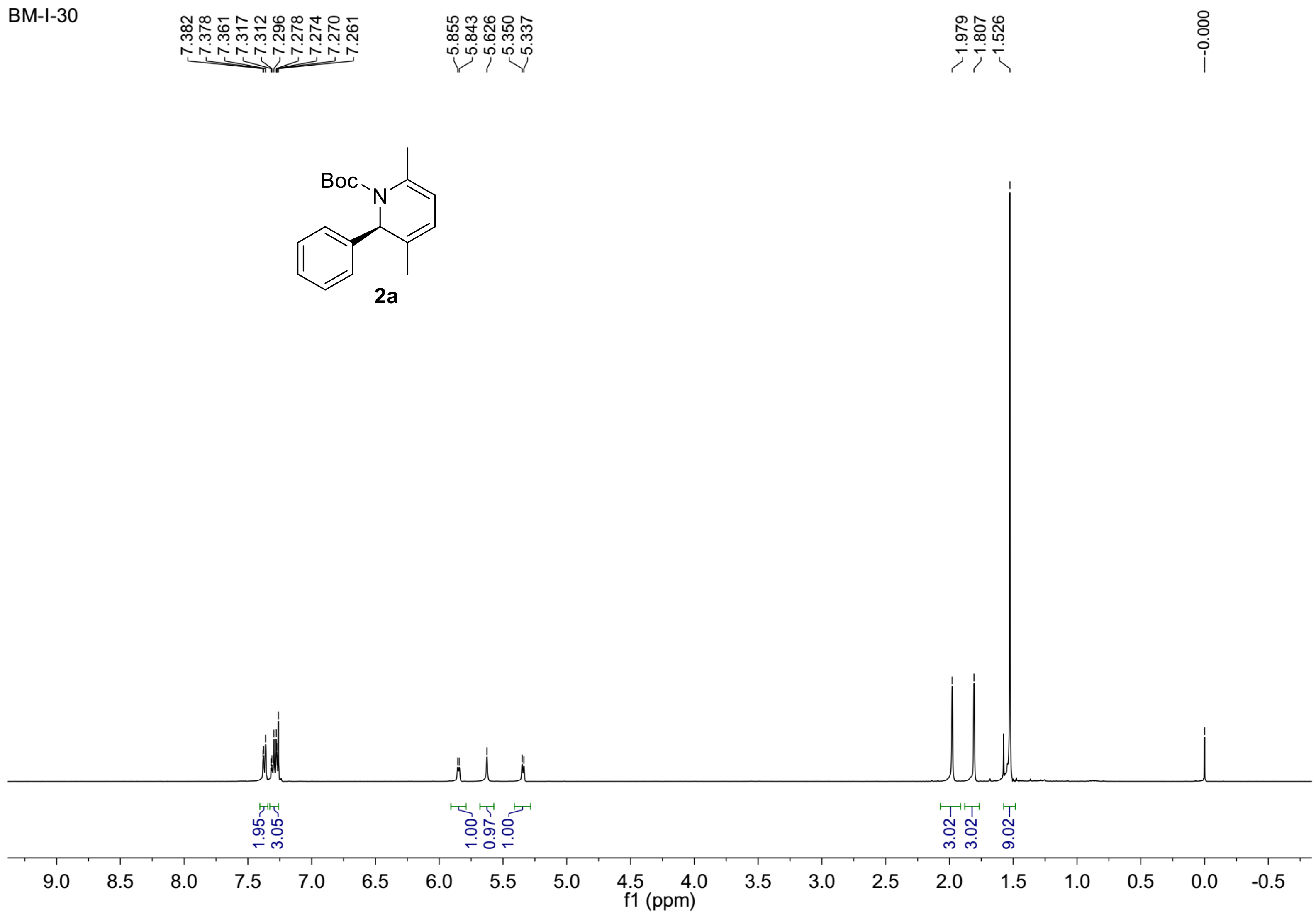

Supplementary Figure 4. ^{1}H NMR (400 MHz, $CDCl_3$) spectra for compound **2a**

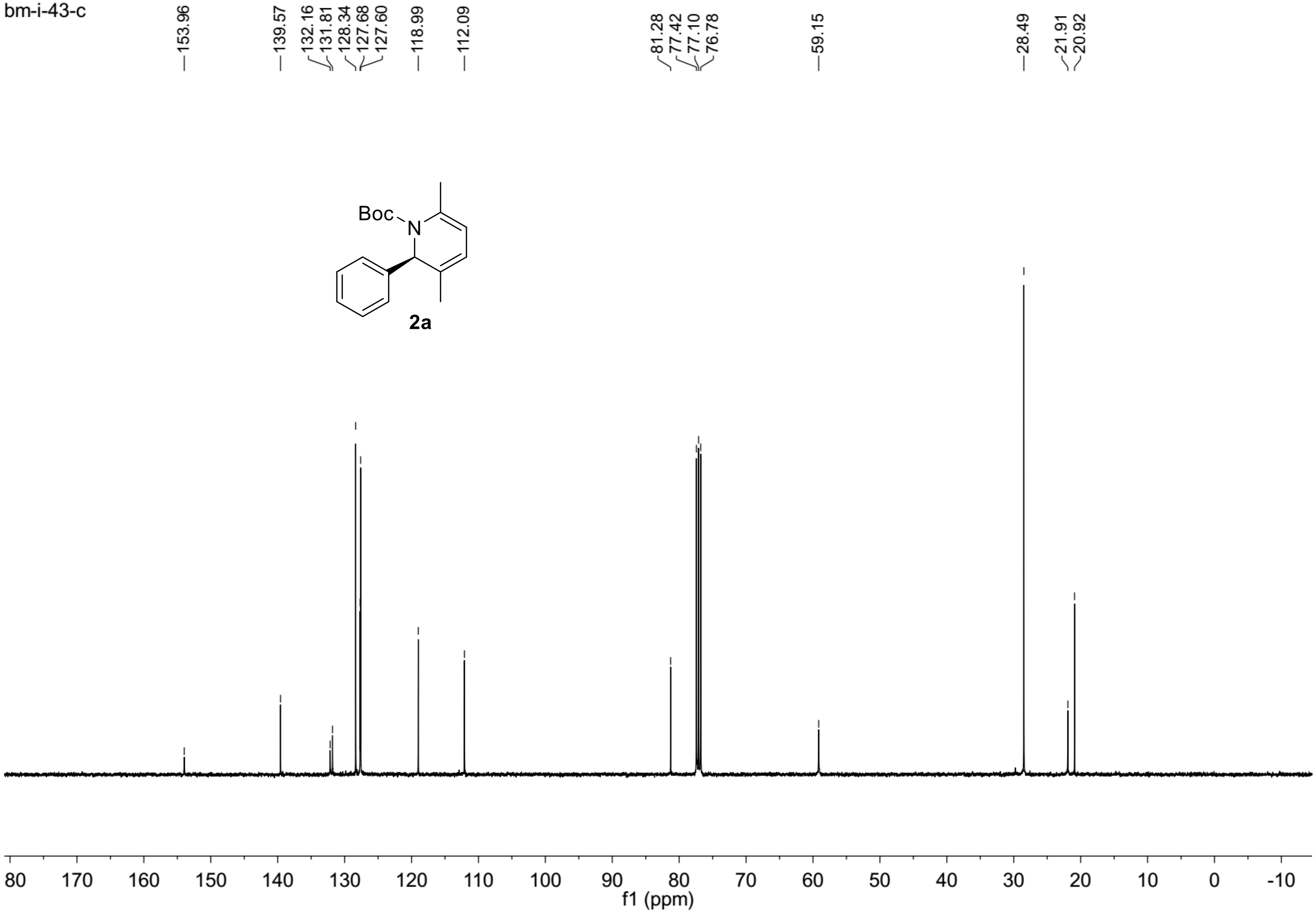

Supplementary Figure 5. ^{13}C NMR (100 MHz, $CDCl_3$) spectra for compound **2a**

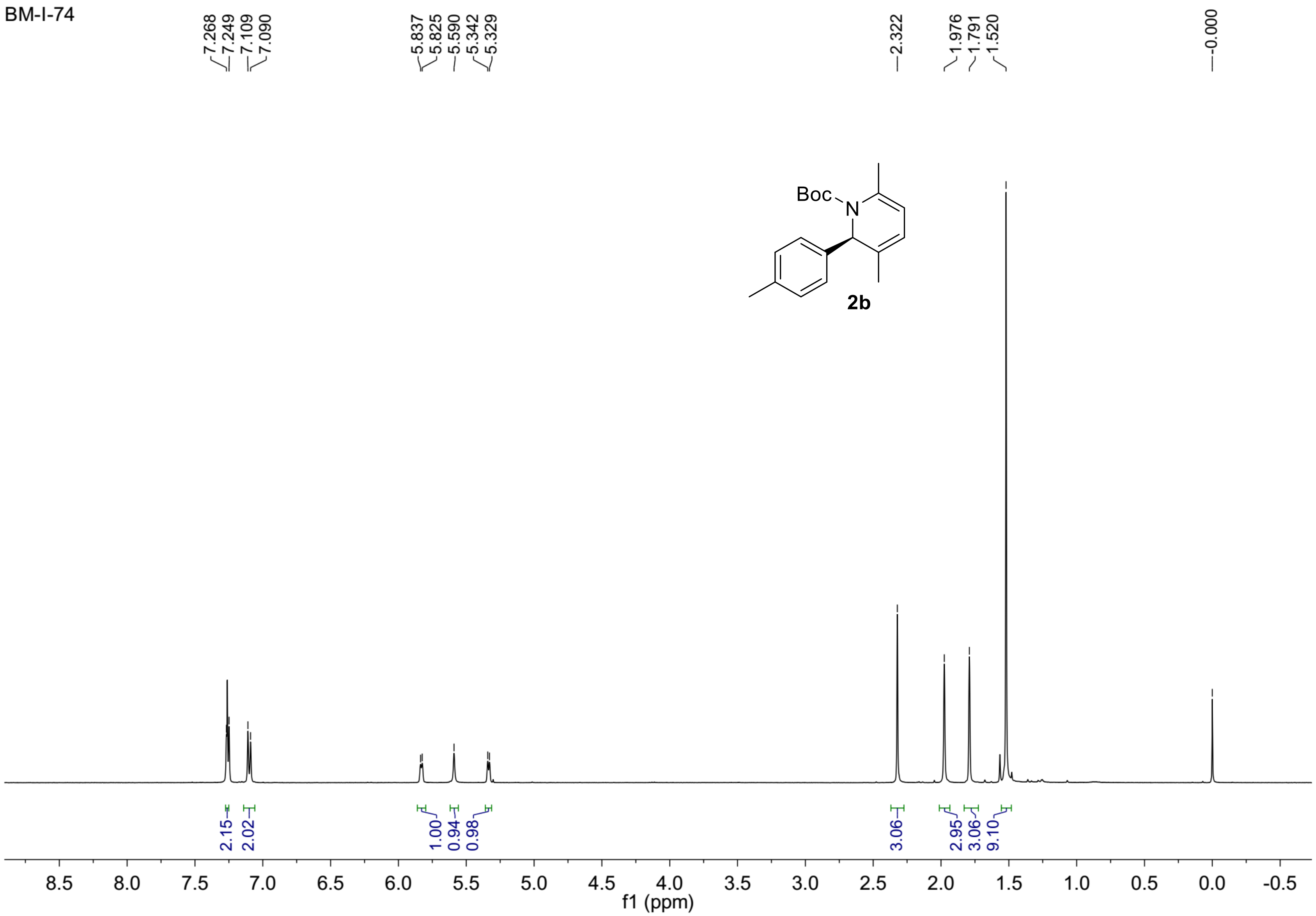

Supplementary Figure 6. ^{1}H NMR (400 MHz, $CDCl_3$) spectra for compound **2b**

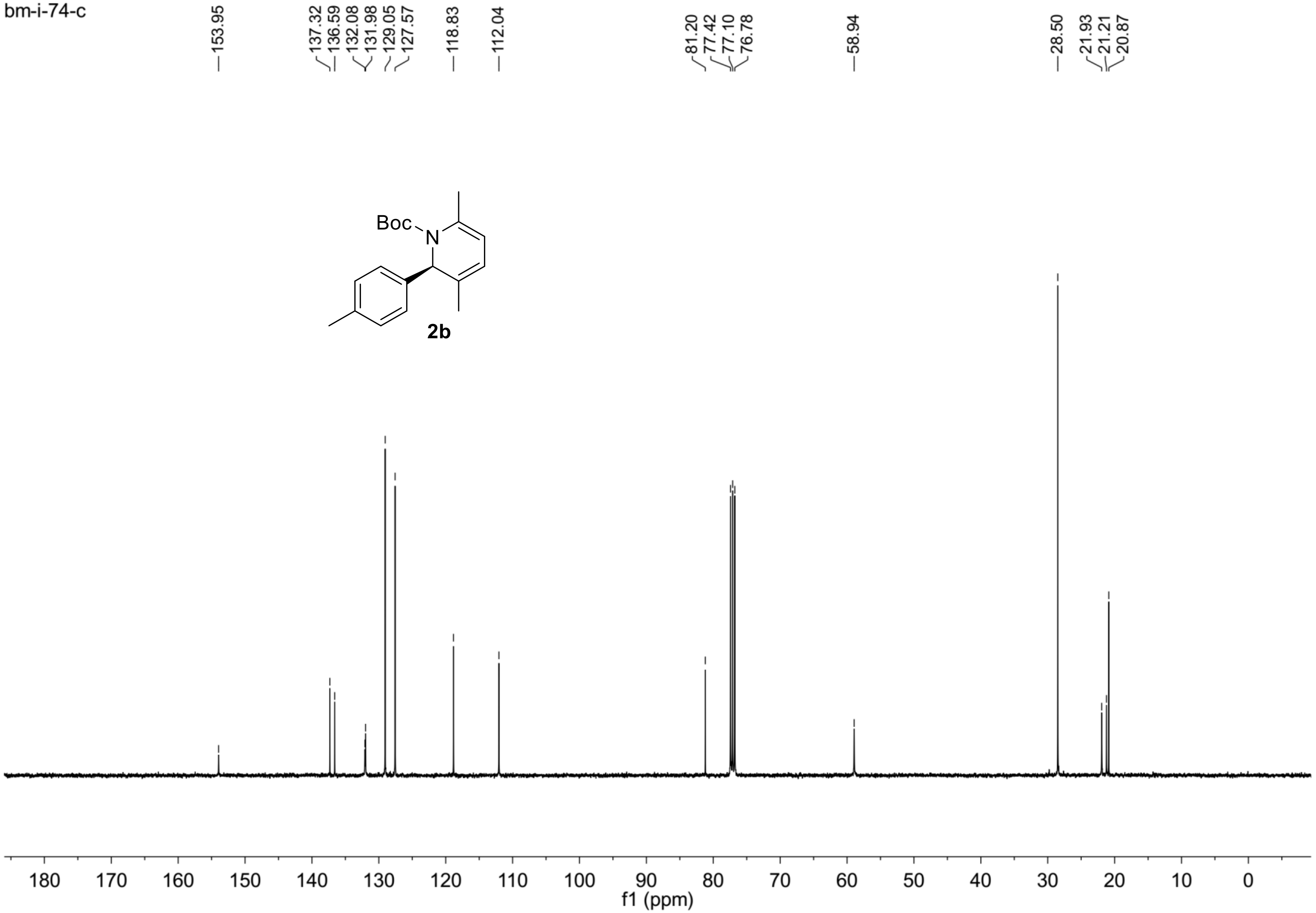

Supplementary Figure 7. ^{13}C NMR (100 MHz, $CDCl_3$) spectra for compound **2b**

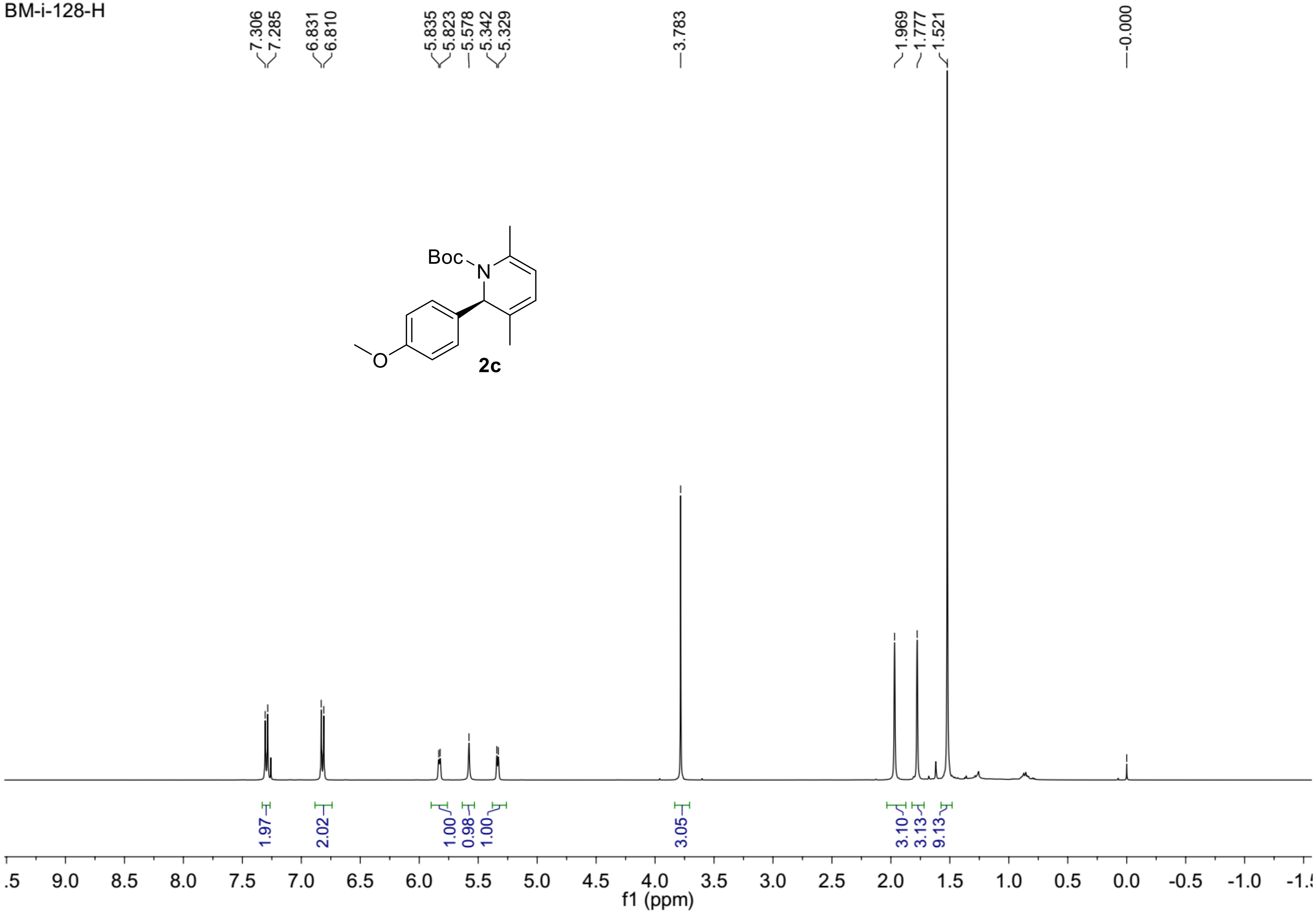

Supplementary Figure 8. ^{1}H NMR (400 MHz, $CDCl_3$) spectra for compound **2c**

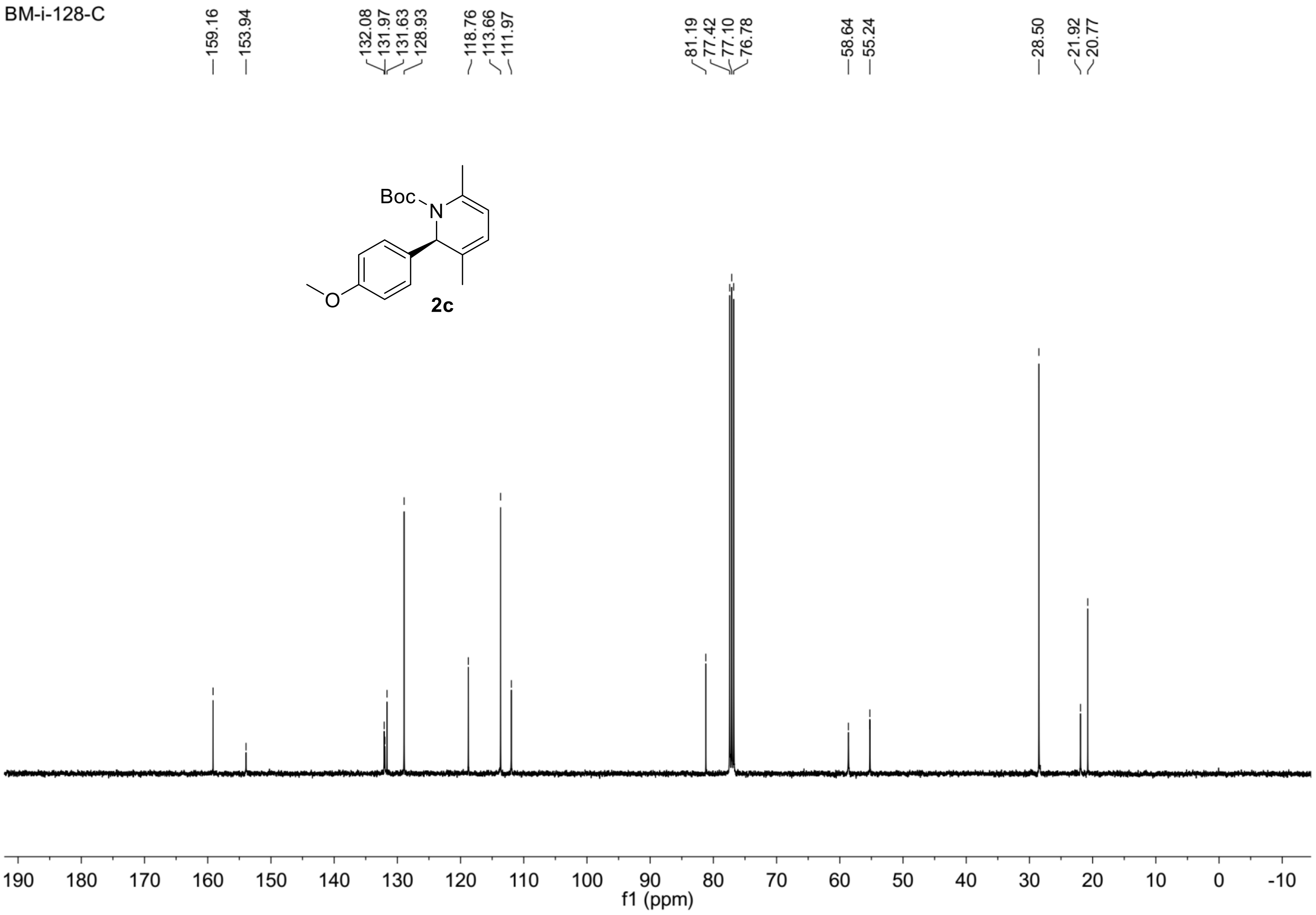

Supplementary Figure 9. ^{13}C NMR (100 MHz, $CDCl_3$) spectra for compound **2c**

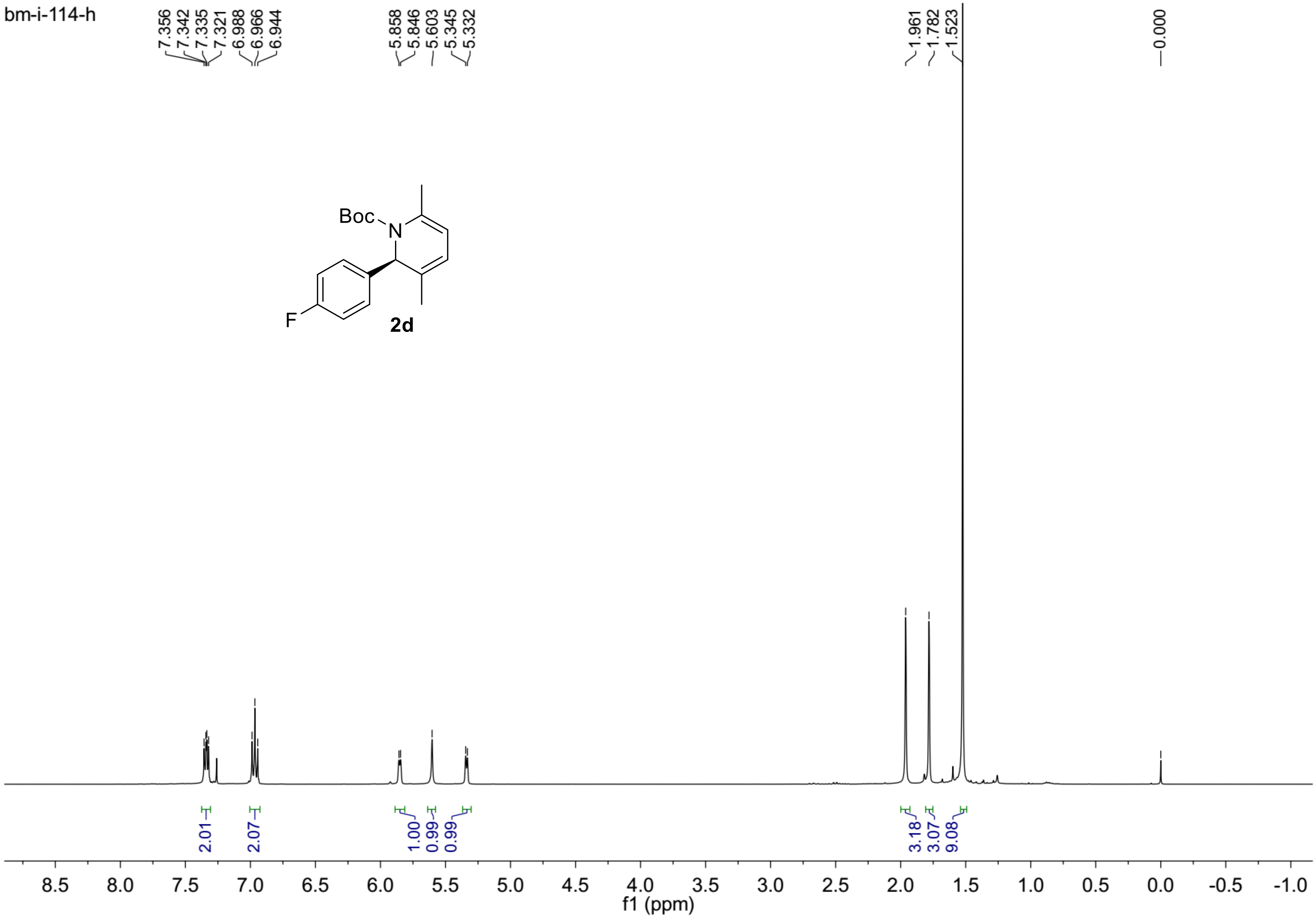

Supplementary Figure 10. ^{1}H NMR (400 MHz, $CDCl_3$) spectra for compound **2d**

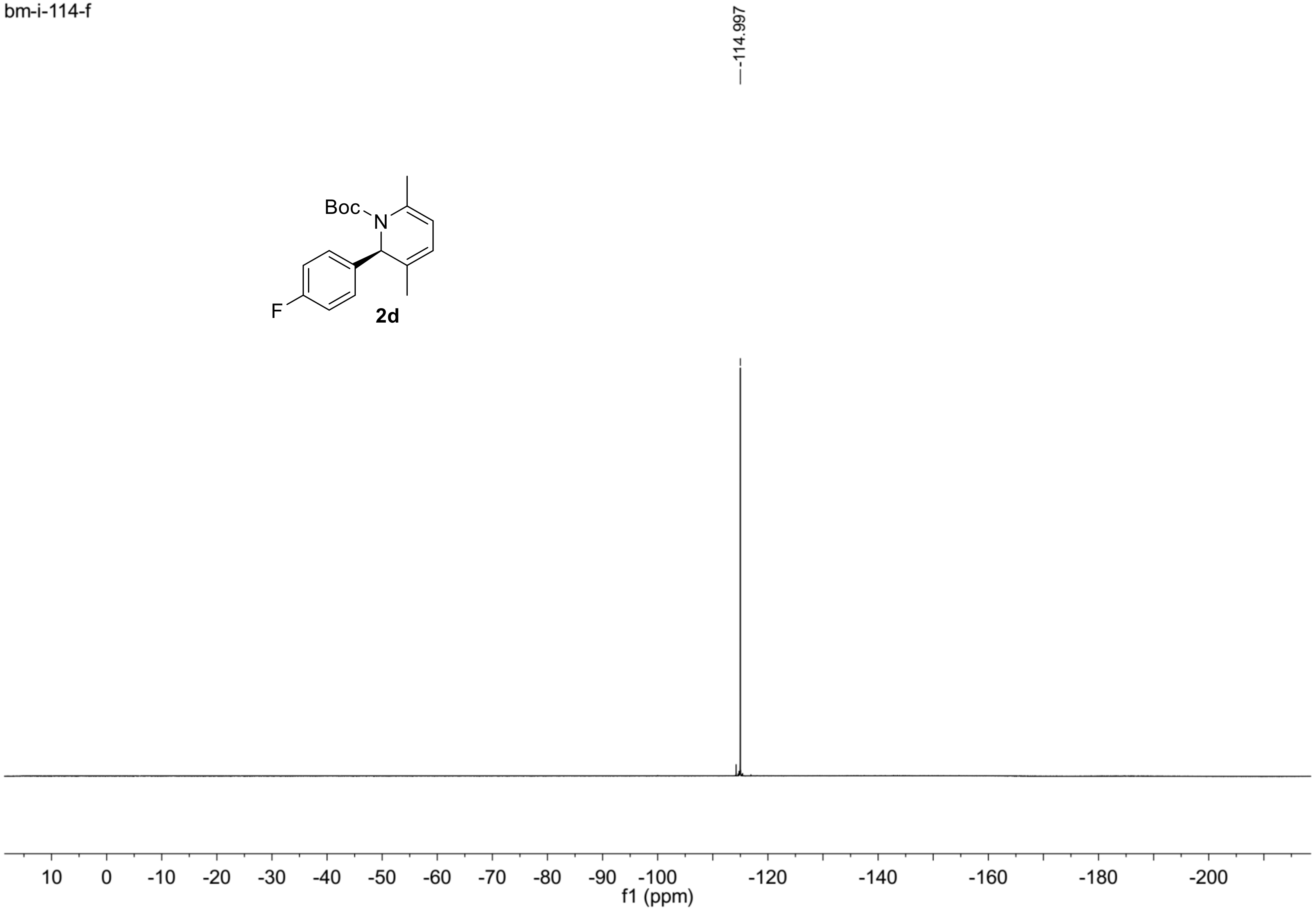

Supplementary Figure 11. ^{19}F NMR (376 MHz, $CDCl_3$) spectra for compound **2d**

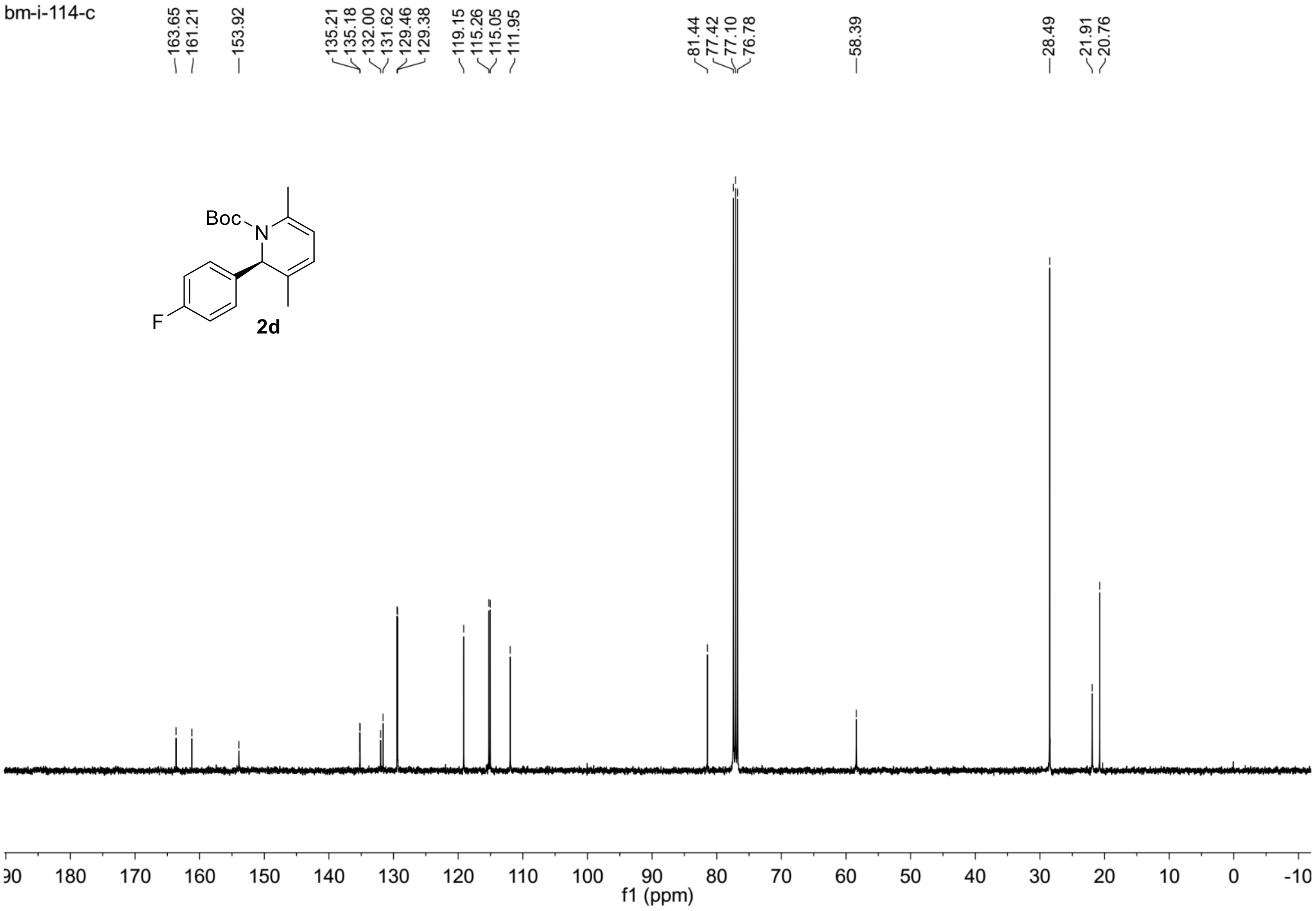

Supplementary Figure 12. ^{13}C NMR (100 MHz, $CDCl_3$) spectra for compound **2d**

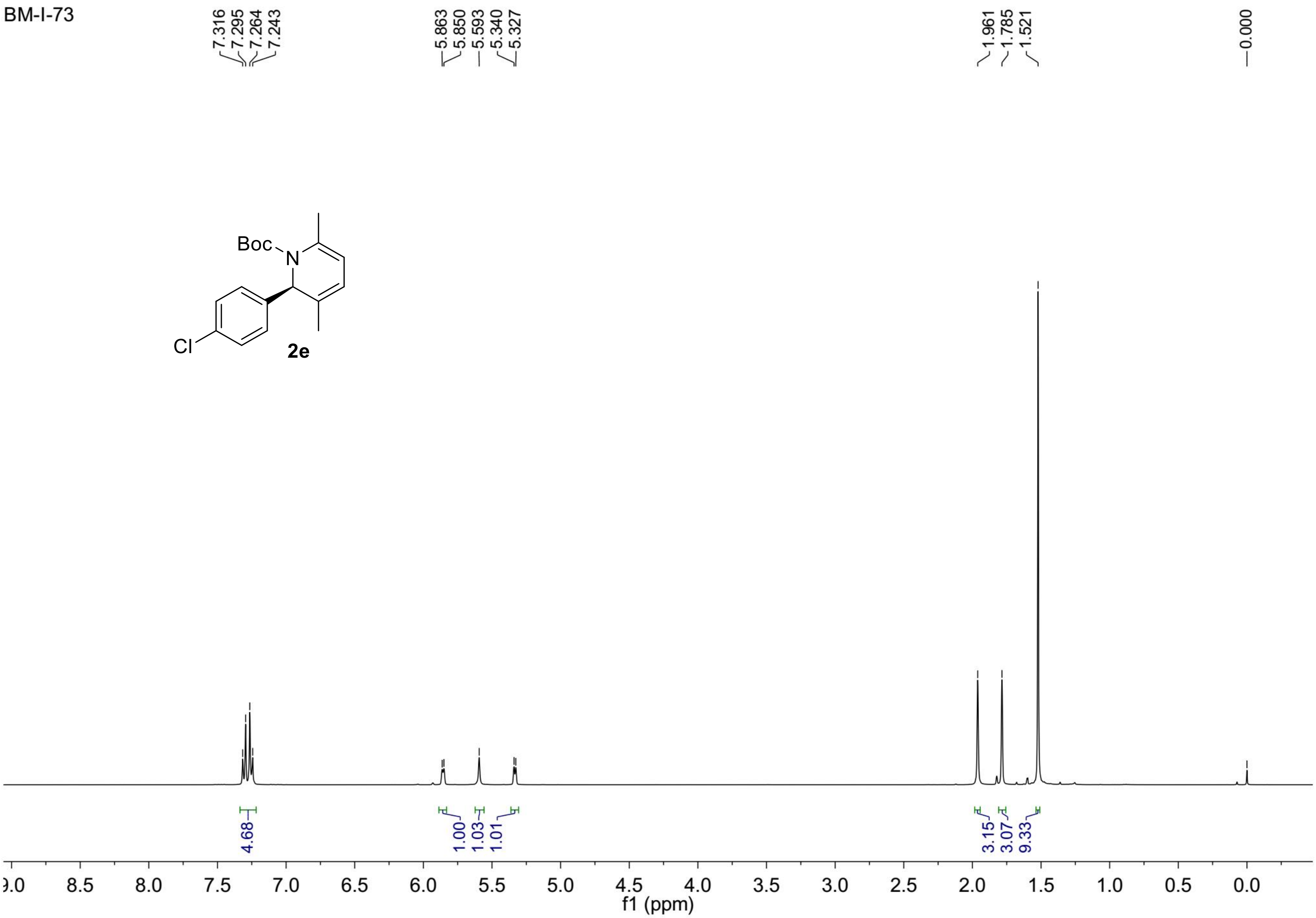

Supplementary Figure 13. ^{1}H NMR (400 MHz, $CDCl_3$) spectra for compound **2e**

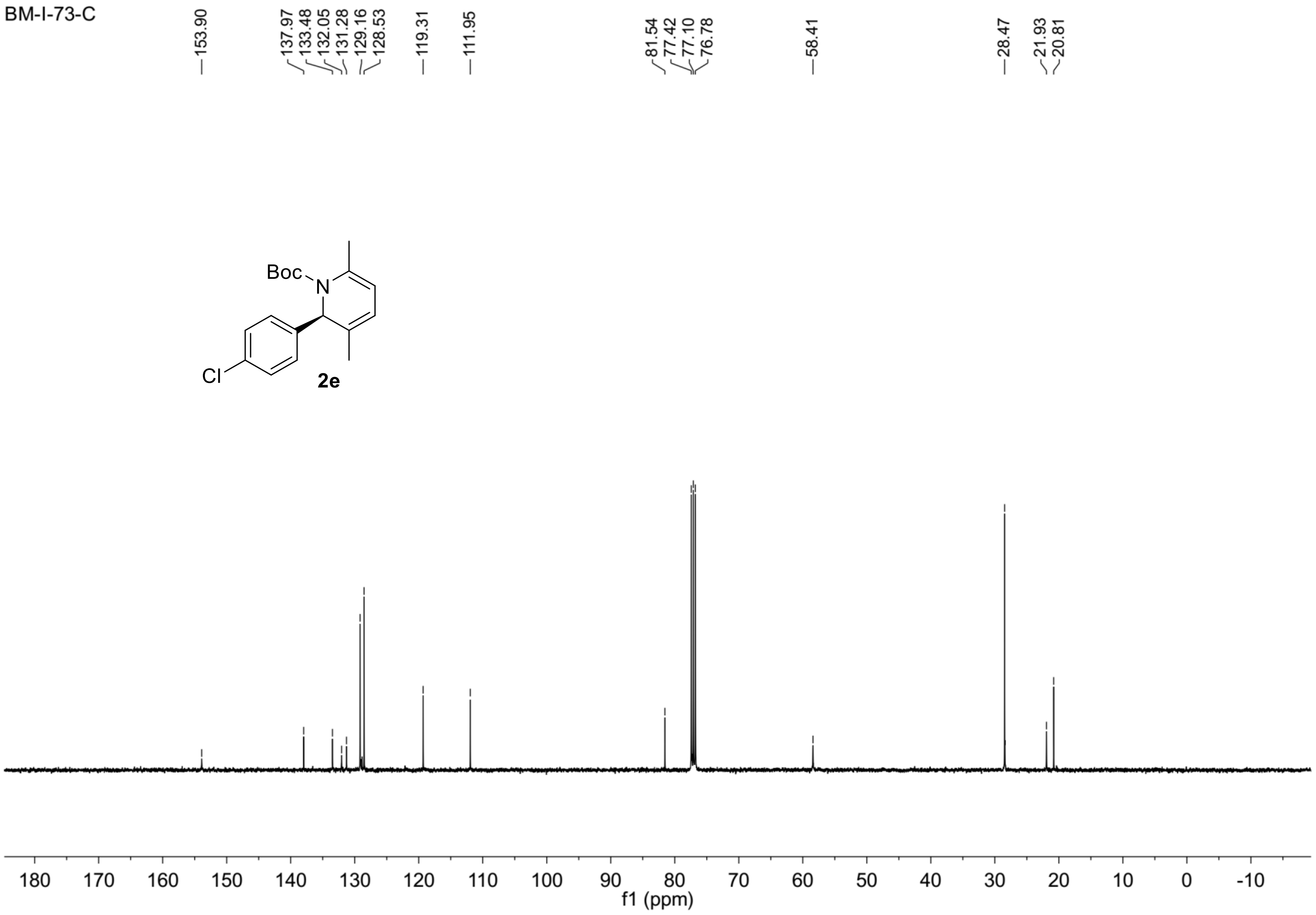

Supplementary Figure 14. ^{13}C NMR (100 MHz, $CDCl_3$) spectra for compound **2e**

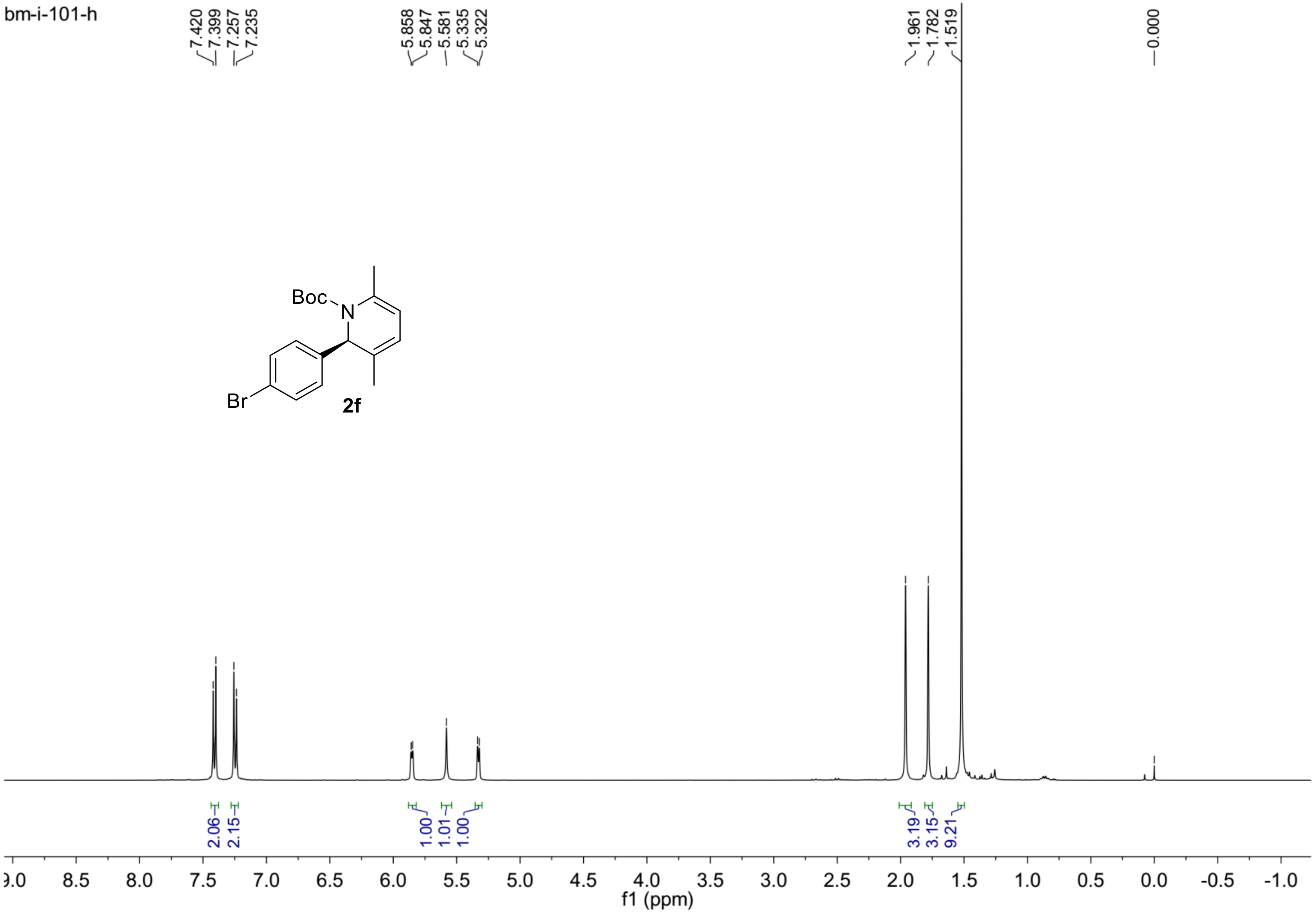

Supplementary Figure 15. ^{1}H NMR (400 MHz, $CDCl_3$) spectra for compound **2f**

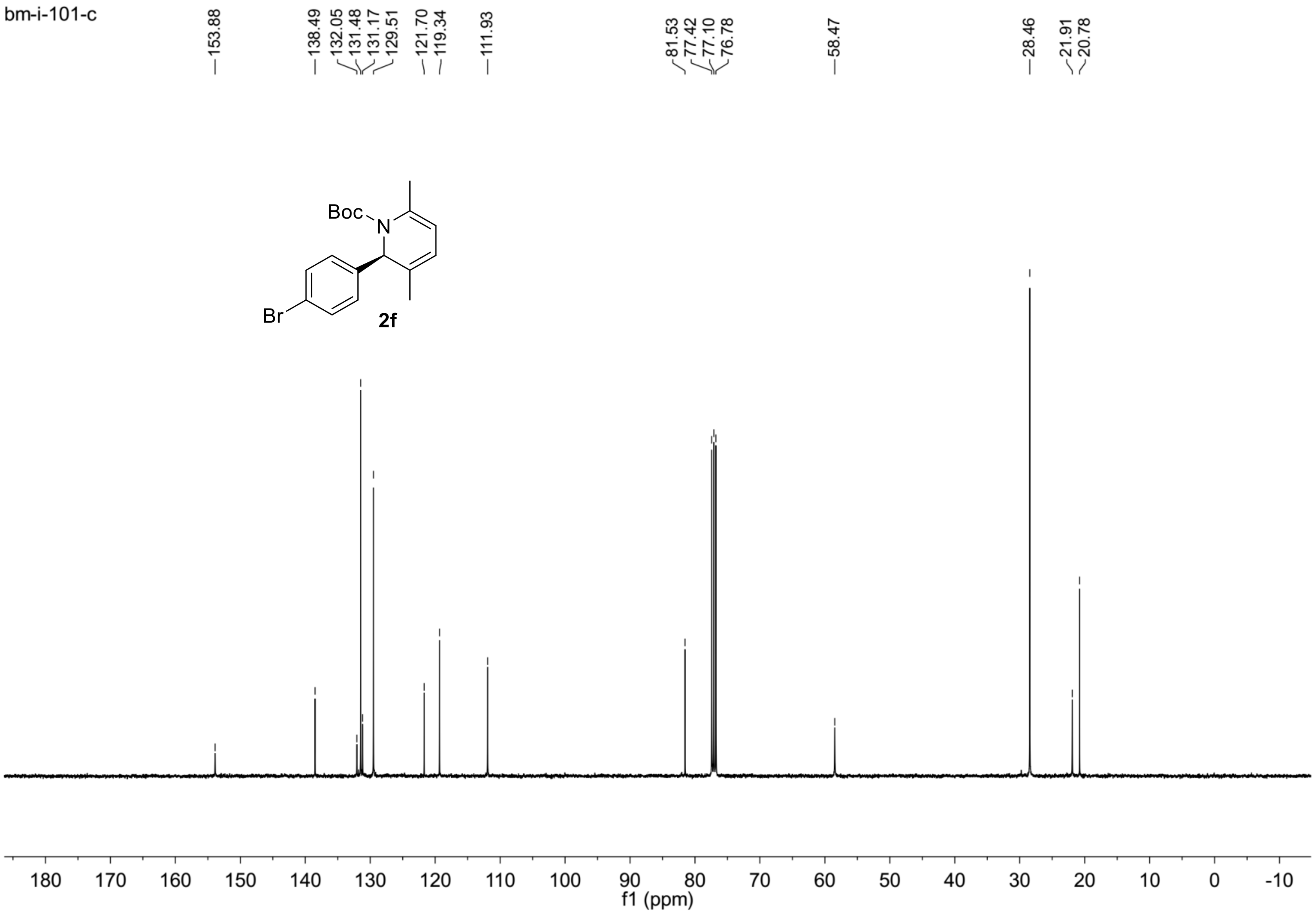

Supplementary Figure 16. ^{13}C NMR (100 MHz, $CDCl_3$) spectra for compound **2f**

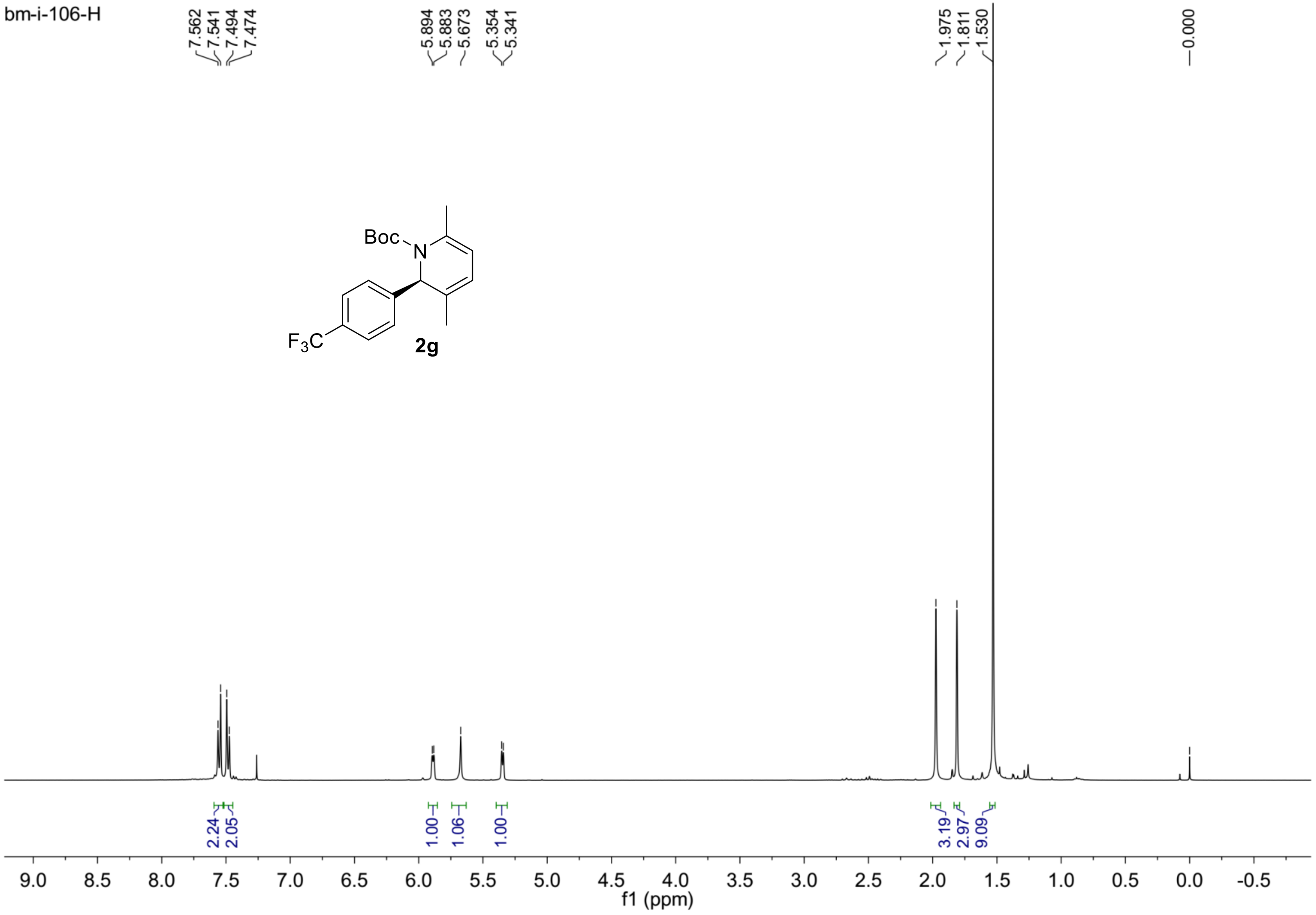

Supplementary Figure 17. ^{1}H NMR (400 MHz, $CDCl_3$) spectra for compound **2g**

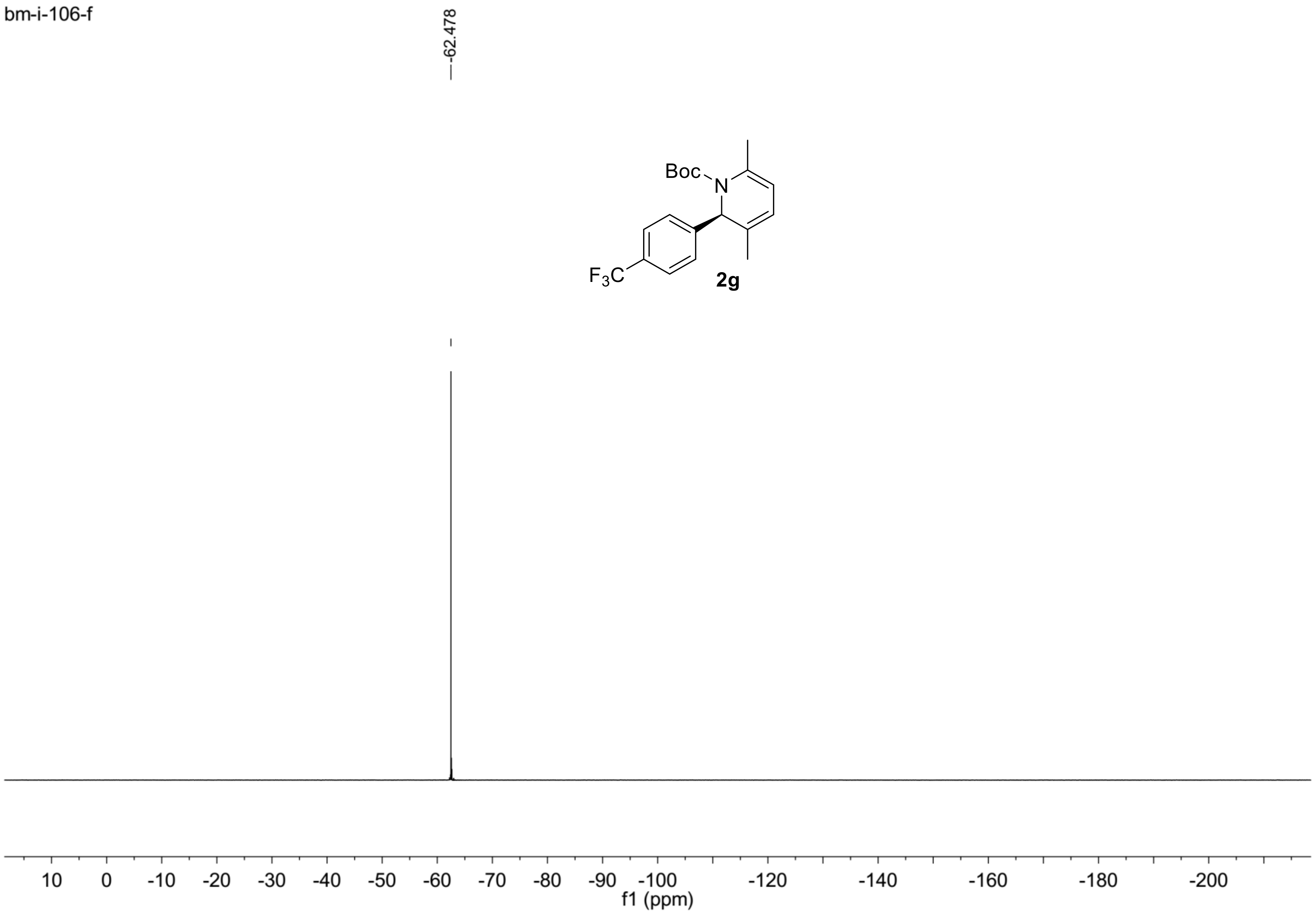

Supplementary Figure 18. ^{19}F NMR (376 MHz, $CDCl_3$) spectra for compound **2g**

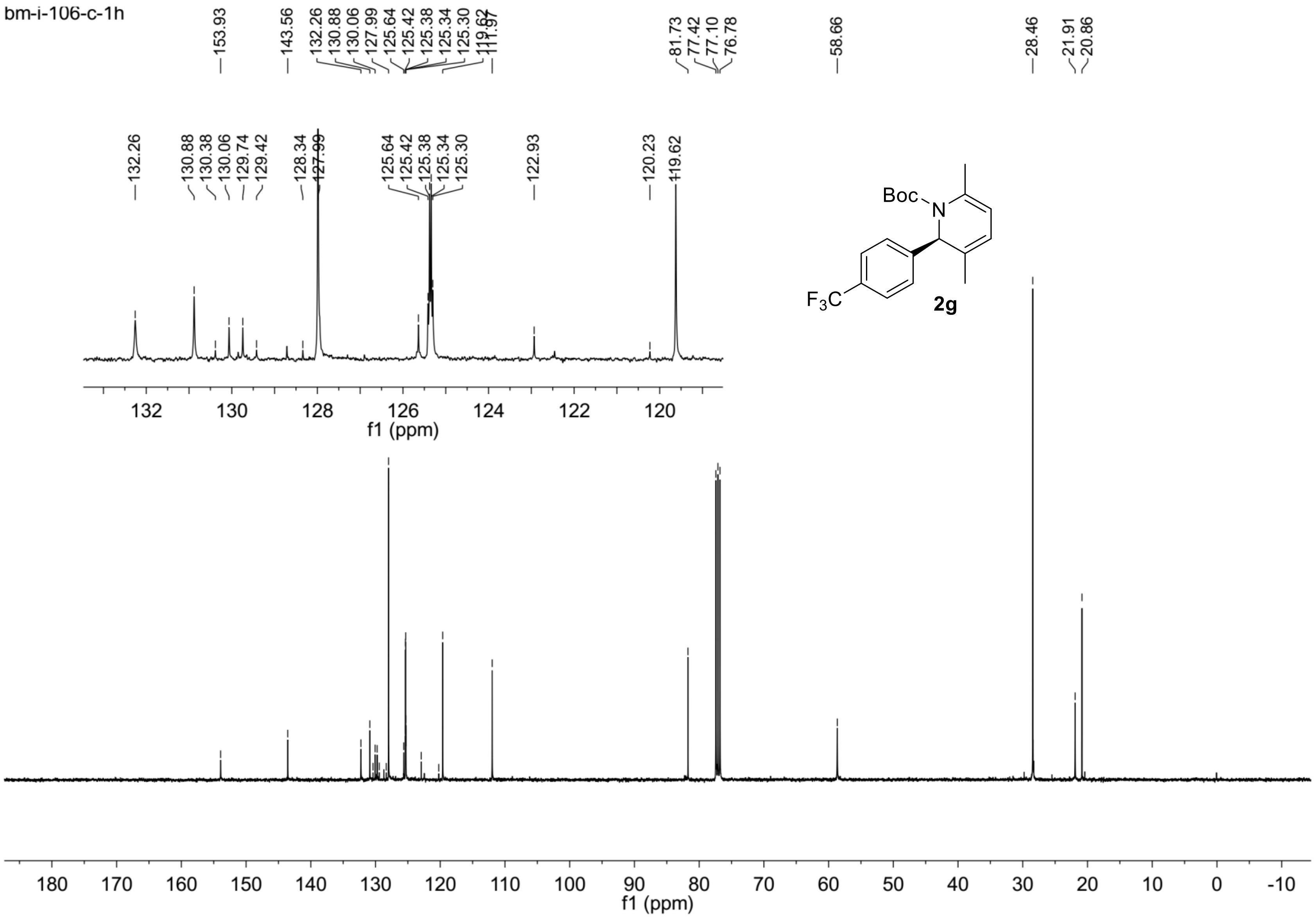

Supplementary Figure 19. ^{13}C NMR (100 MHz, $CDCl_3$) spectra for compound **2g**

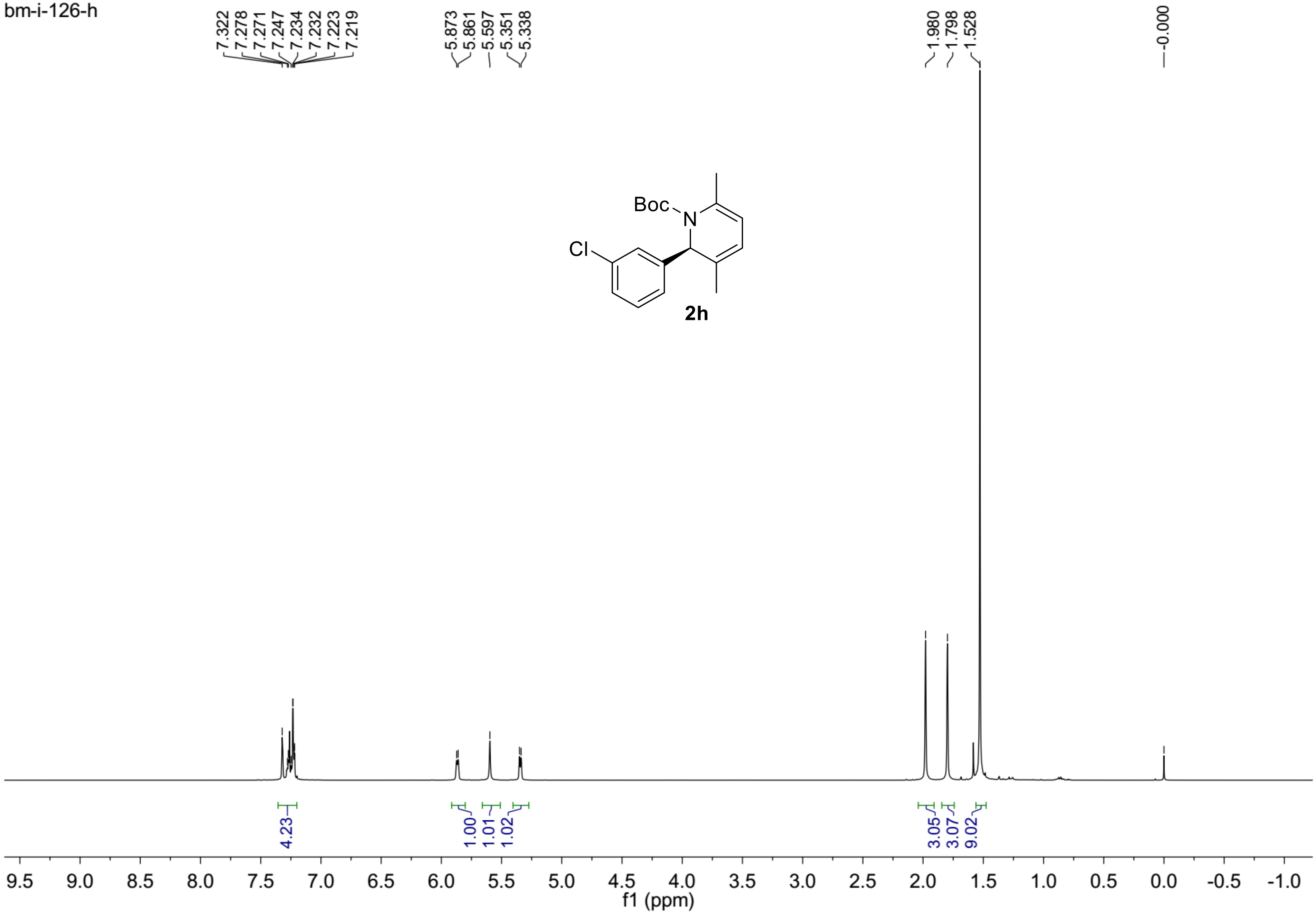

Supplementary Figure 20. ^{1}H NMR (400 MHz, $CDCl_3$) spectra for compound **2h**

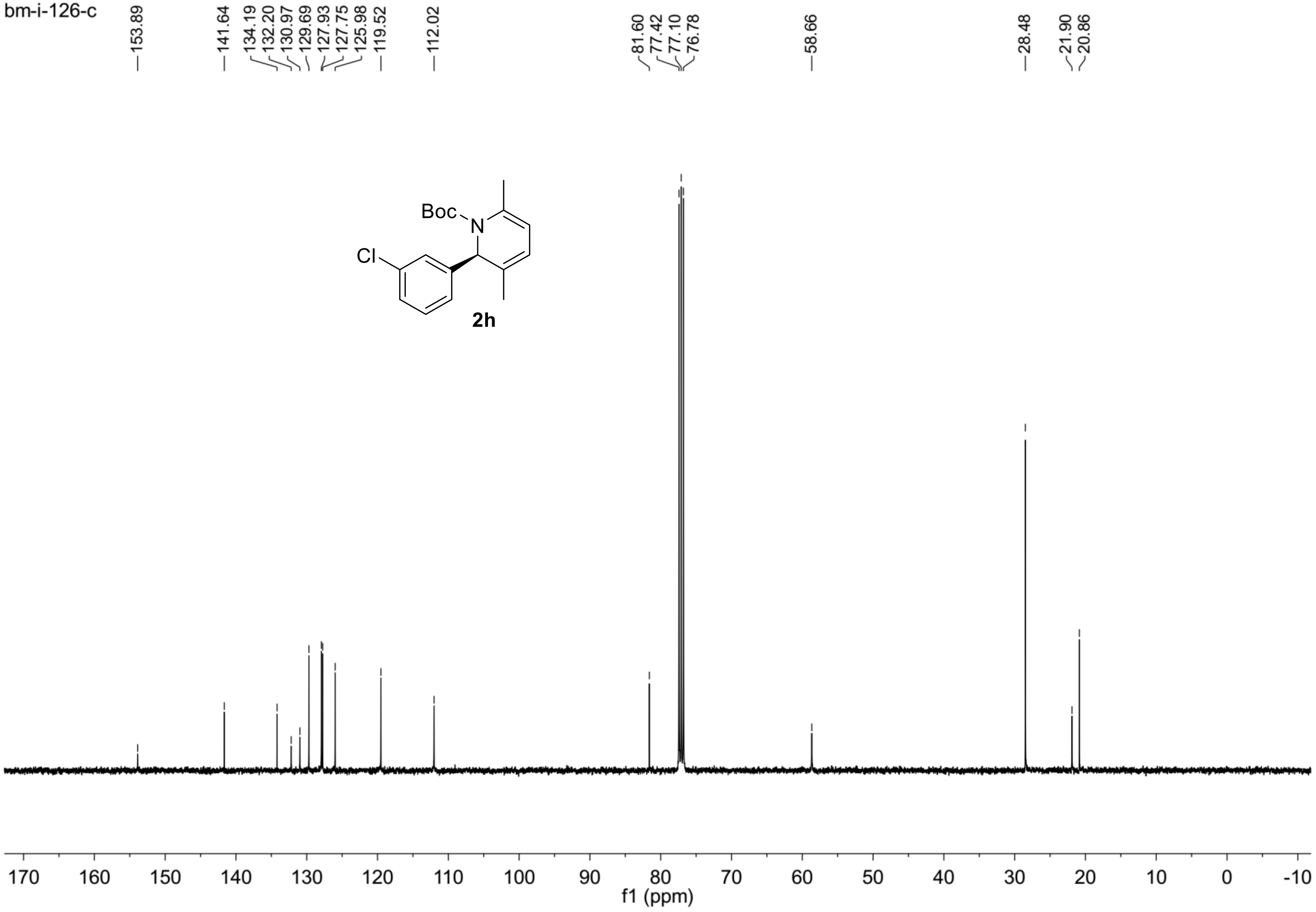

Supplementary Figure 21. ^{13}C NMR (100 MHz, $CDCl_3$) spectra for compound **2h**

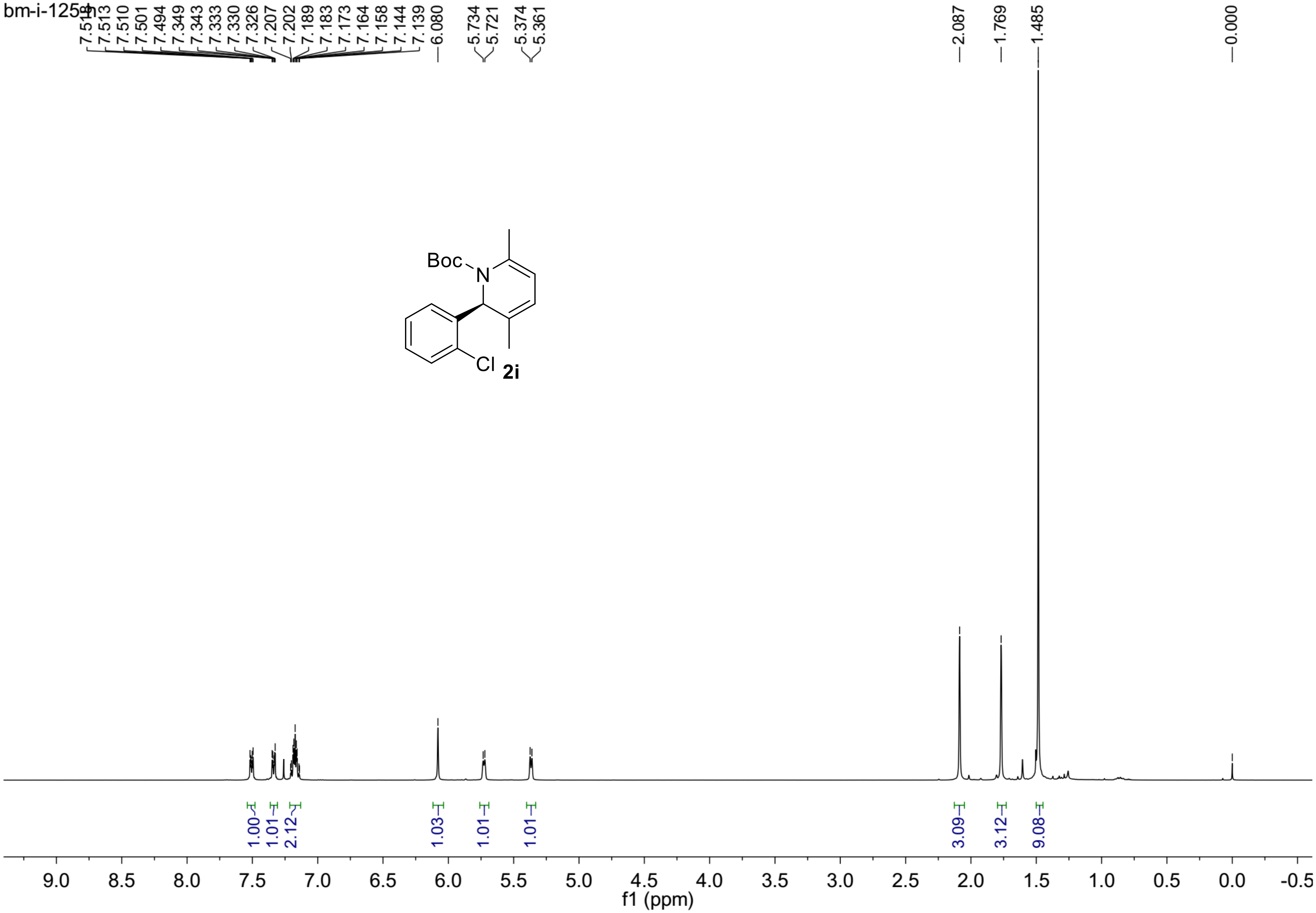

Supplementary Figure 22. ^{1}H NMR (400 MHz, $CDCl_3$) spectra for compound **2i**

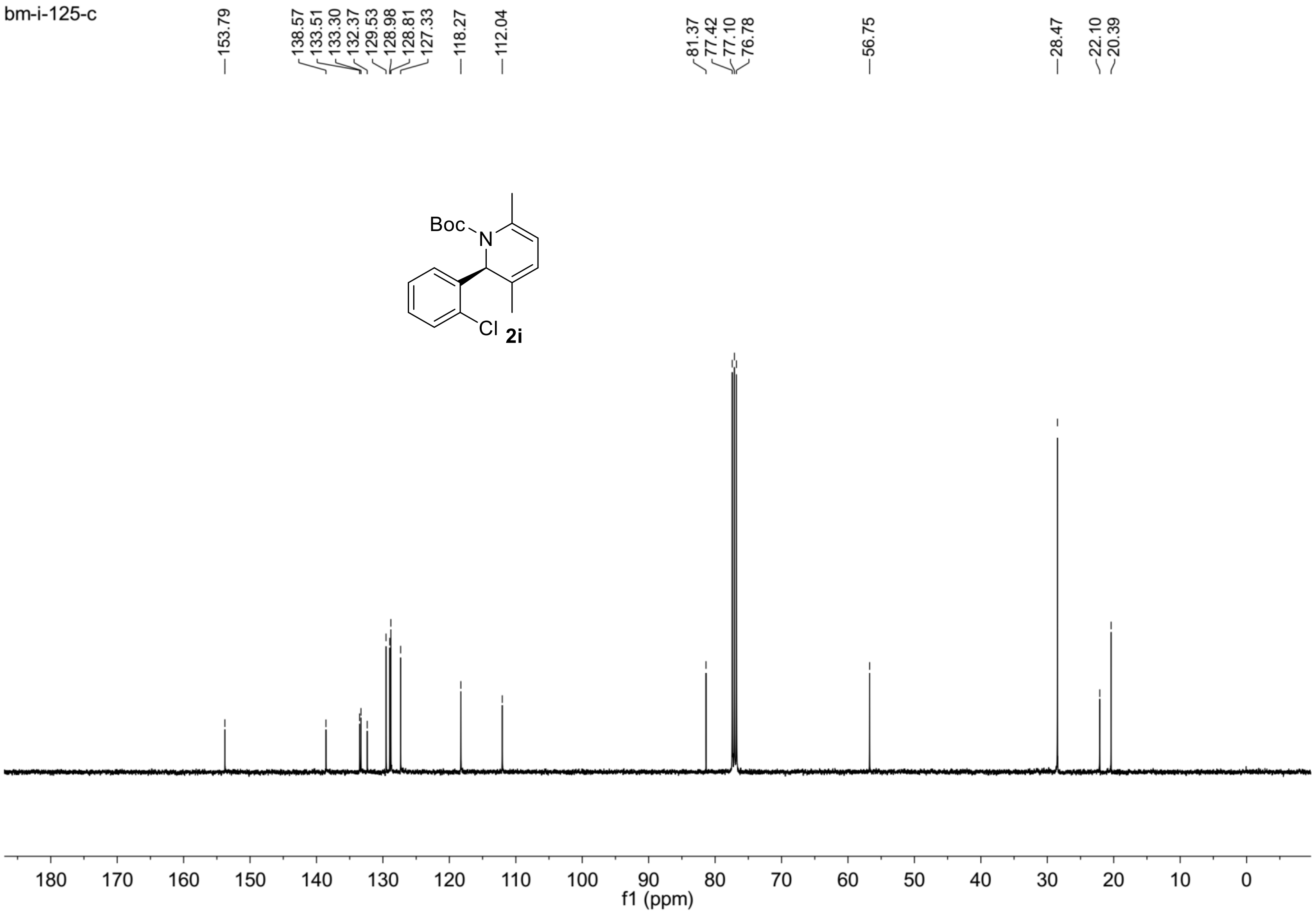

Supplementary Figure 23. ^{13}C NMR (100 MHz, $CDCl_3$) spectra for compound **2i**

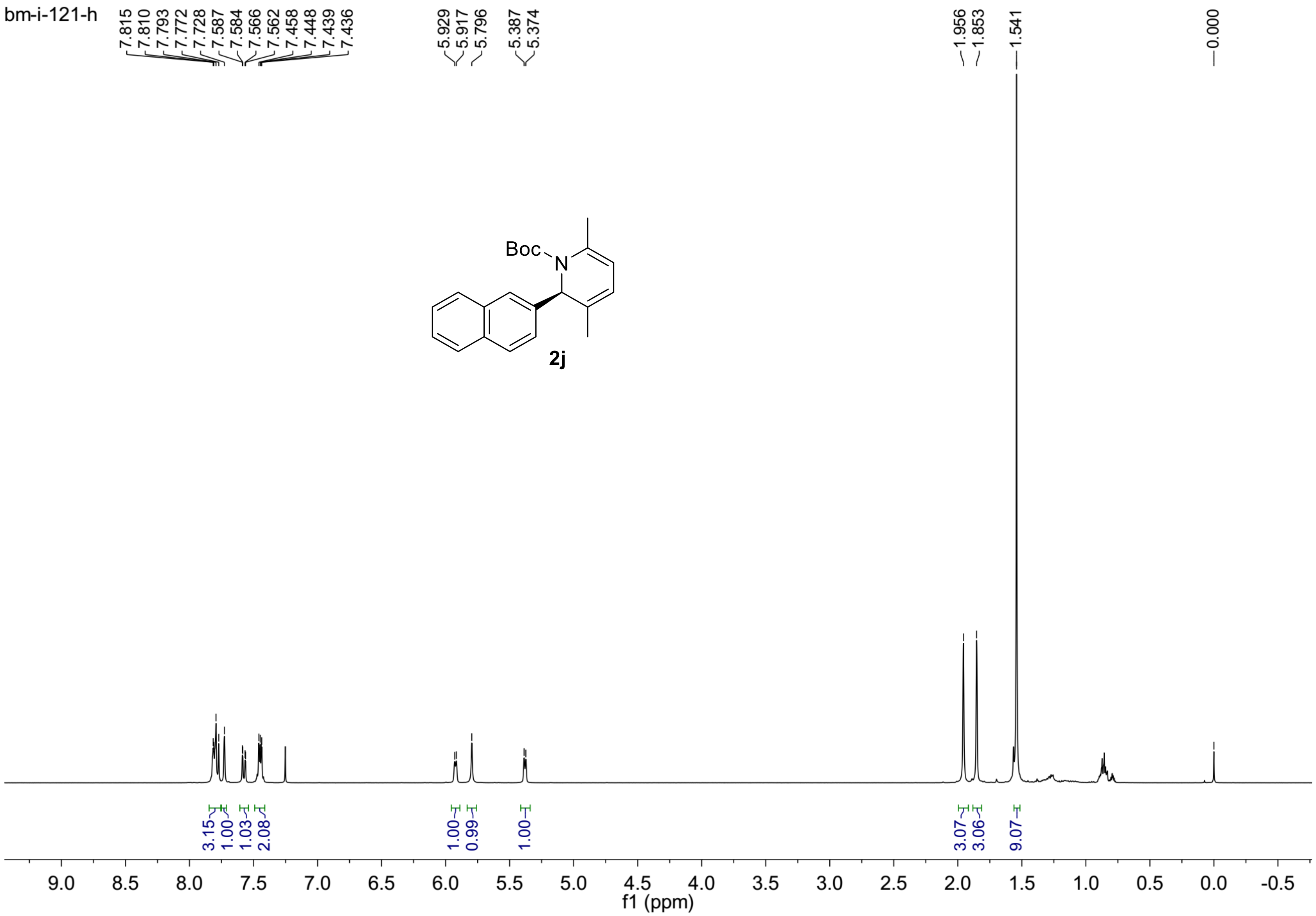

Supplementary Figure 24. ^{1}H NMR (400 MHz, $CDCl_3$) spectra for compound **2j**

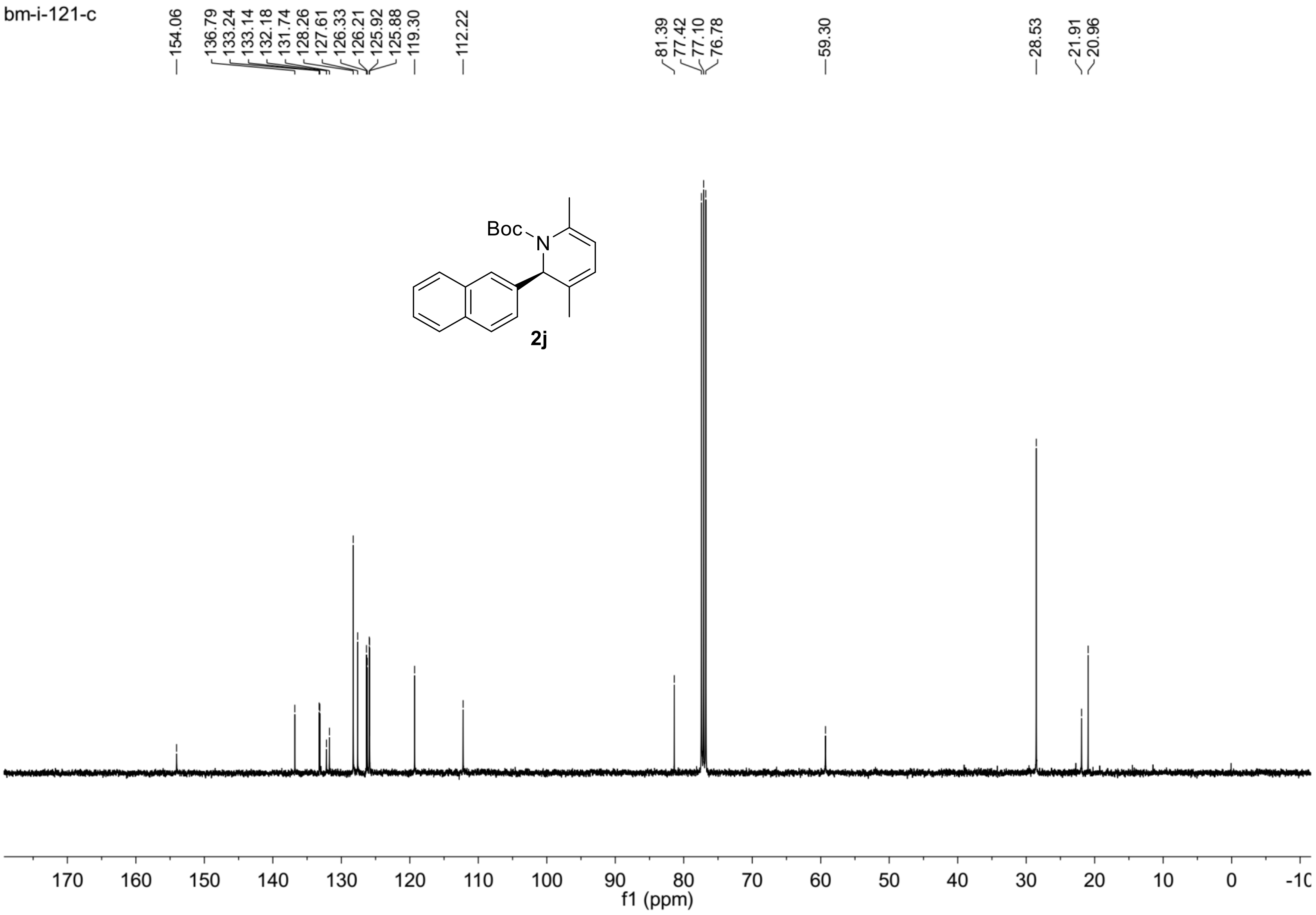

Supplementary Figure 25. ^{13}C NMR (100 MHz, $CDCl_3$) spectra for compound **2j**

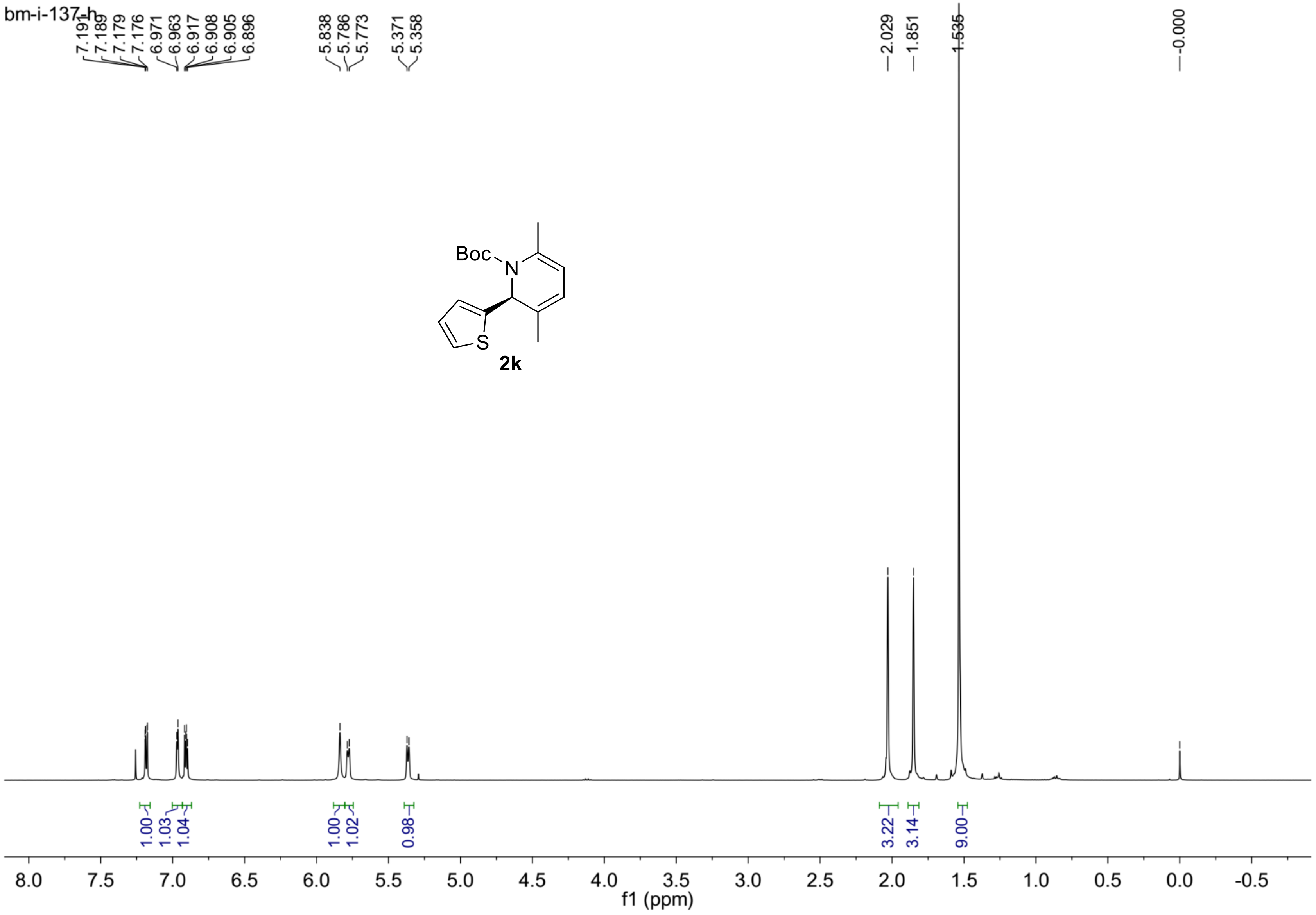

Supplementary Figure 26. ^{1}H NMR (400 MHz, $CDCl_3$) spectra for compound **2k**

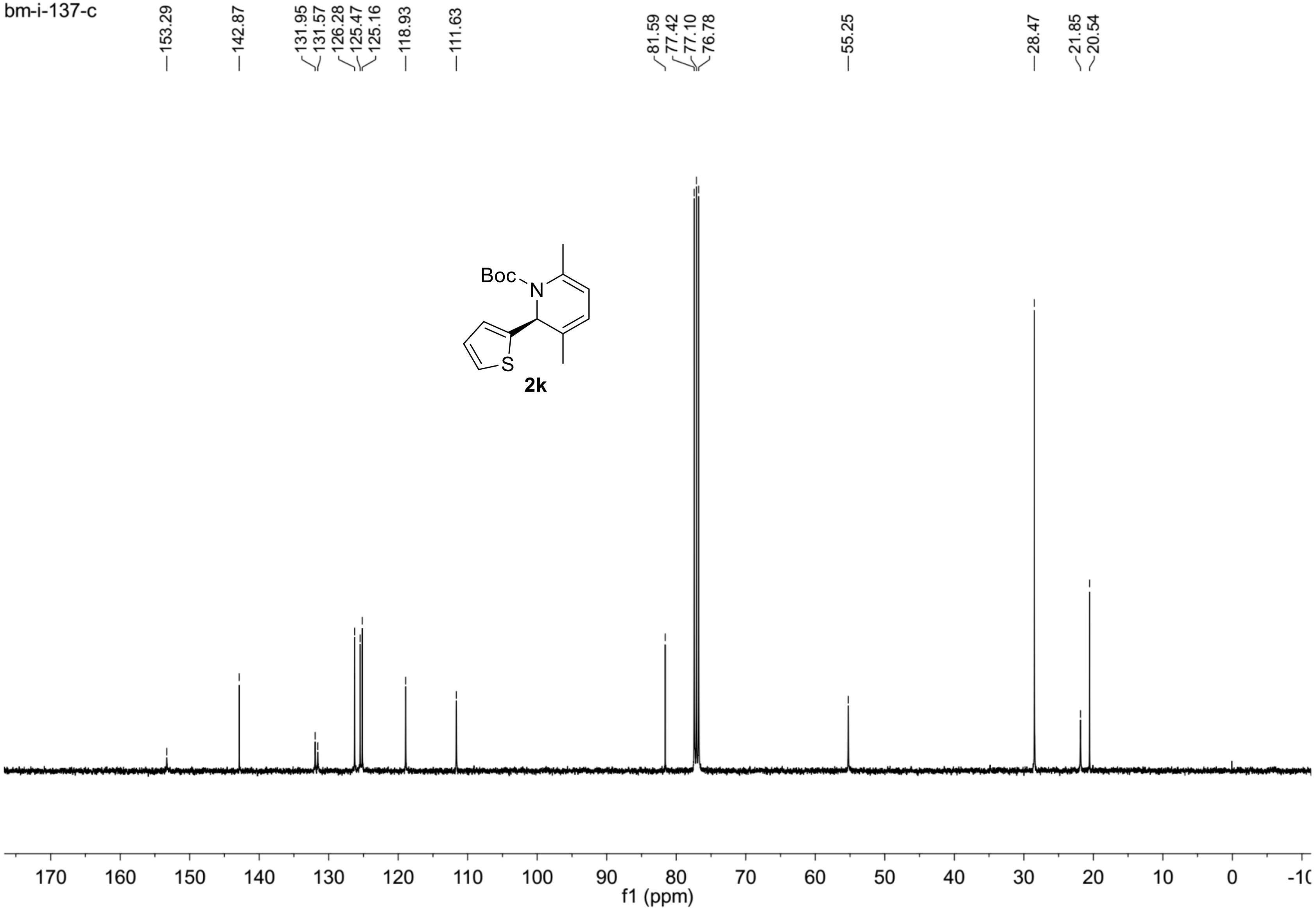

Supplementary Figure 27. ^{13}C NMR (100 MHz, $CDCl_3$) spectra for compound **2k**

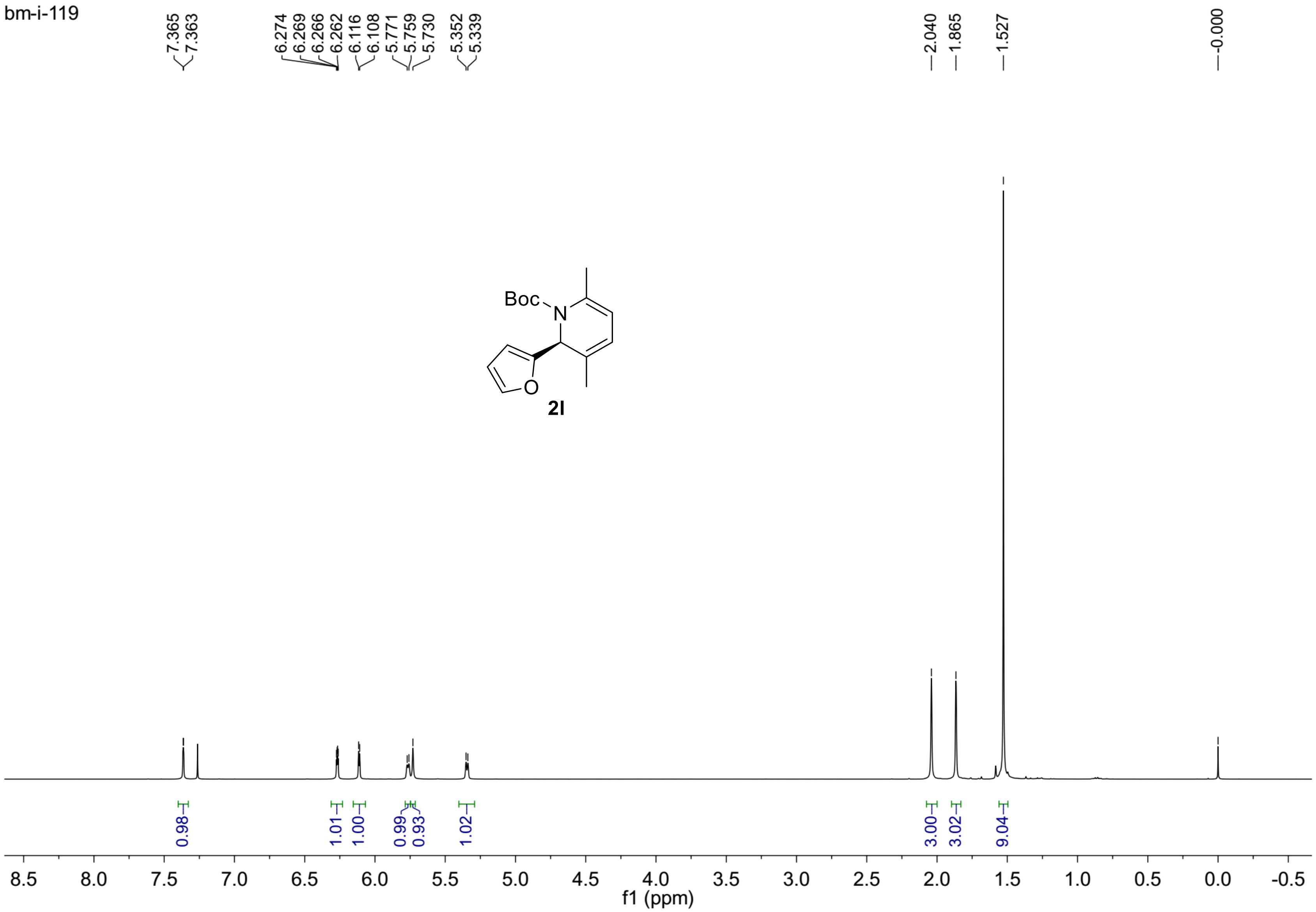

Supplementary Figure 28. ^{1}H NMR (400 MHz, $CDCl_3$) spectra for compound **2l**

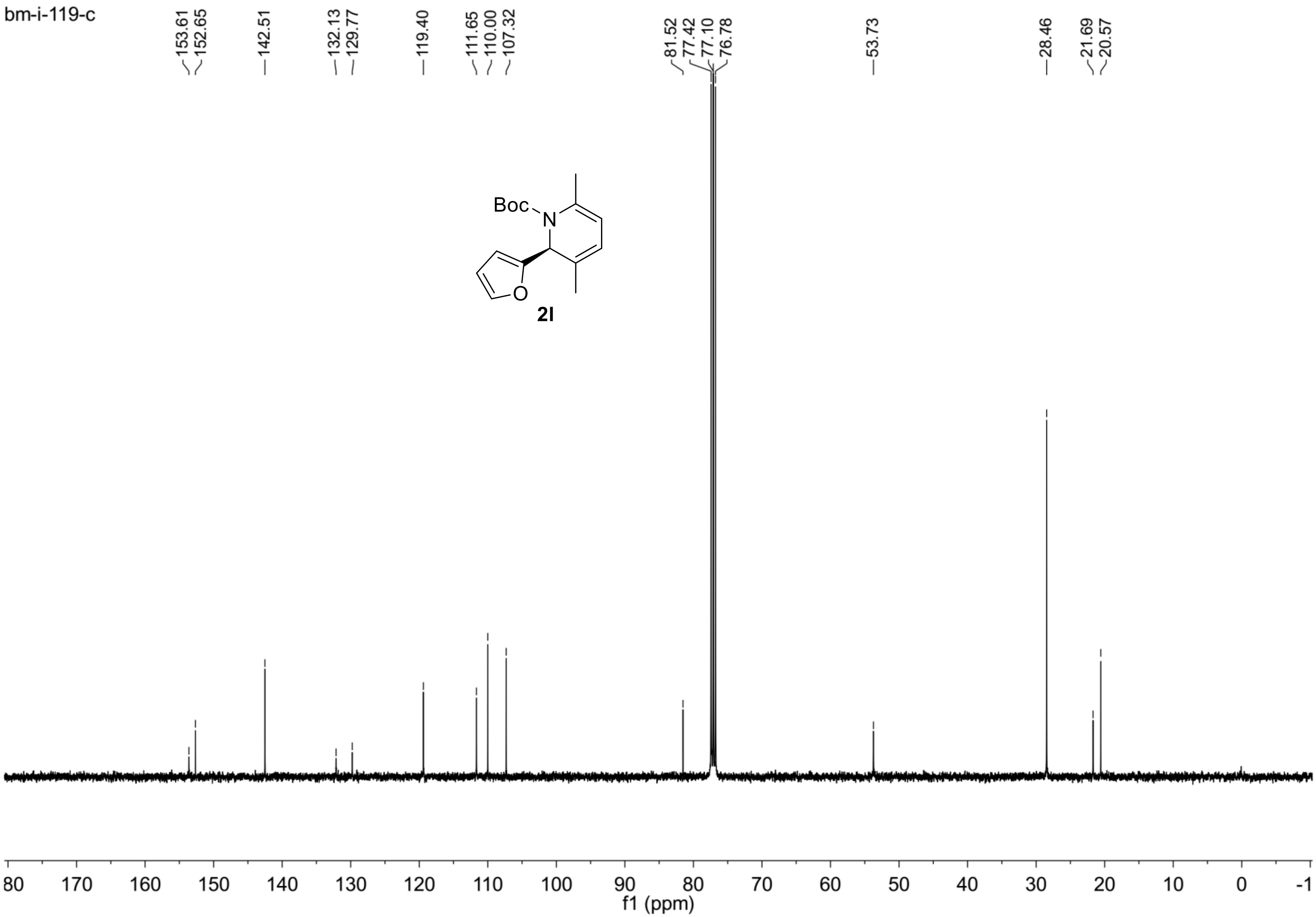

Supplementary Figure 29. ^{13}C NMR (100 MHz, $CDCl_3$) spectra for compound **2l**

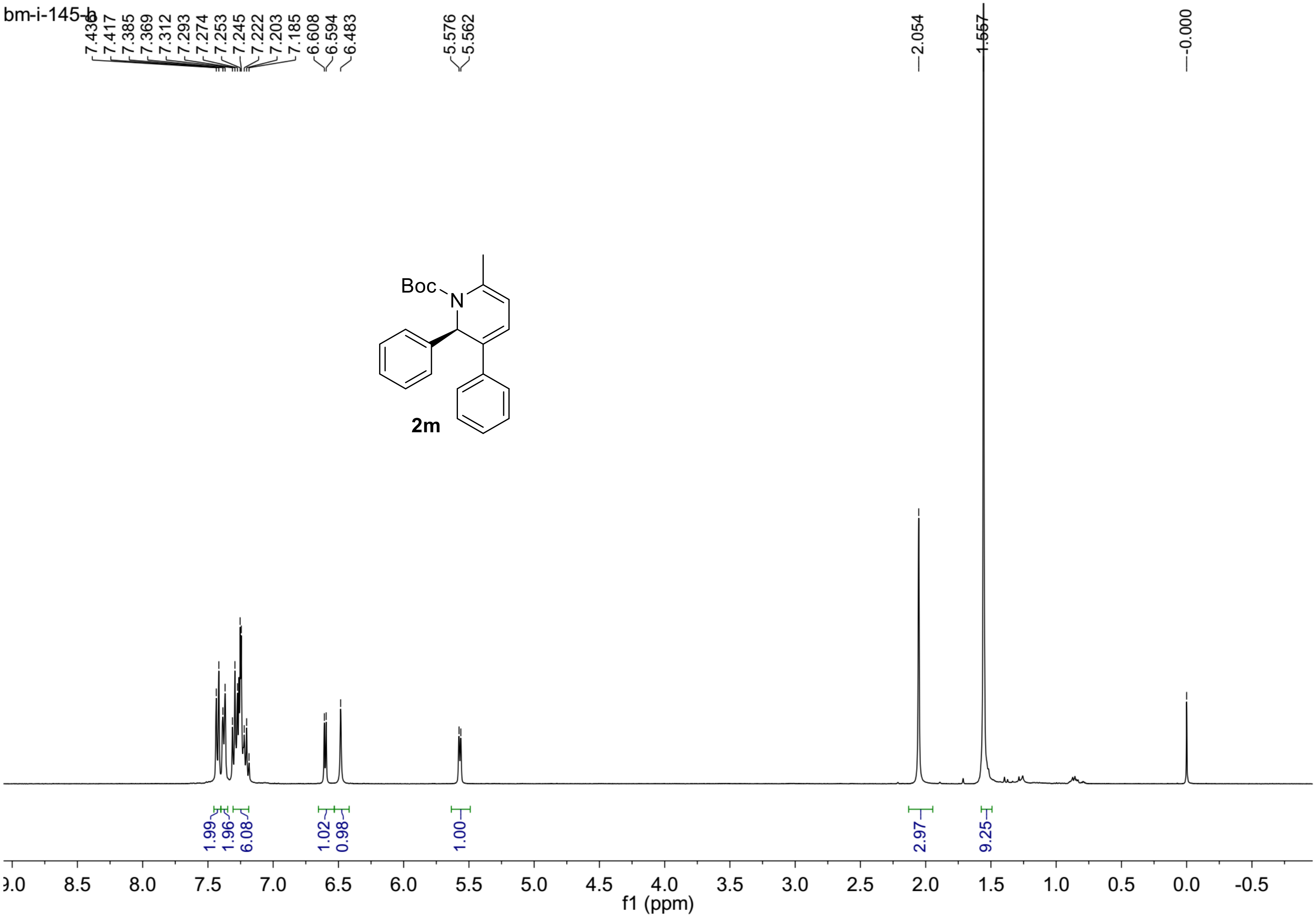

Supplementary Figure 30. ^{1}H NMR (400 MHz, $CDCl_3$) spectra for compound **2m**

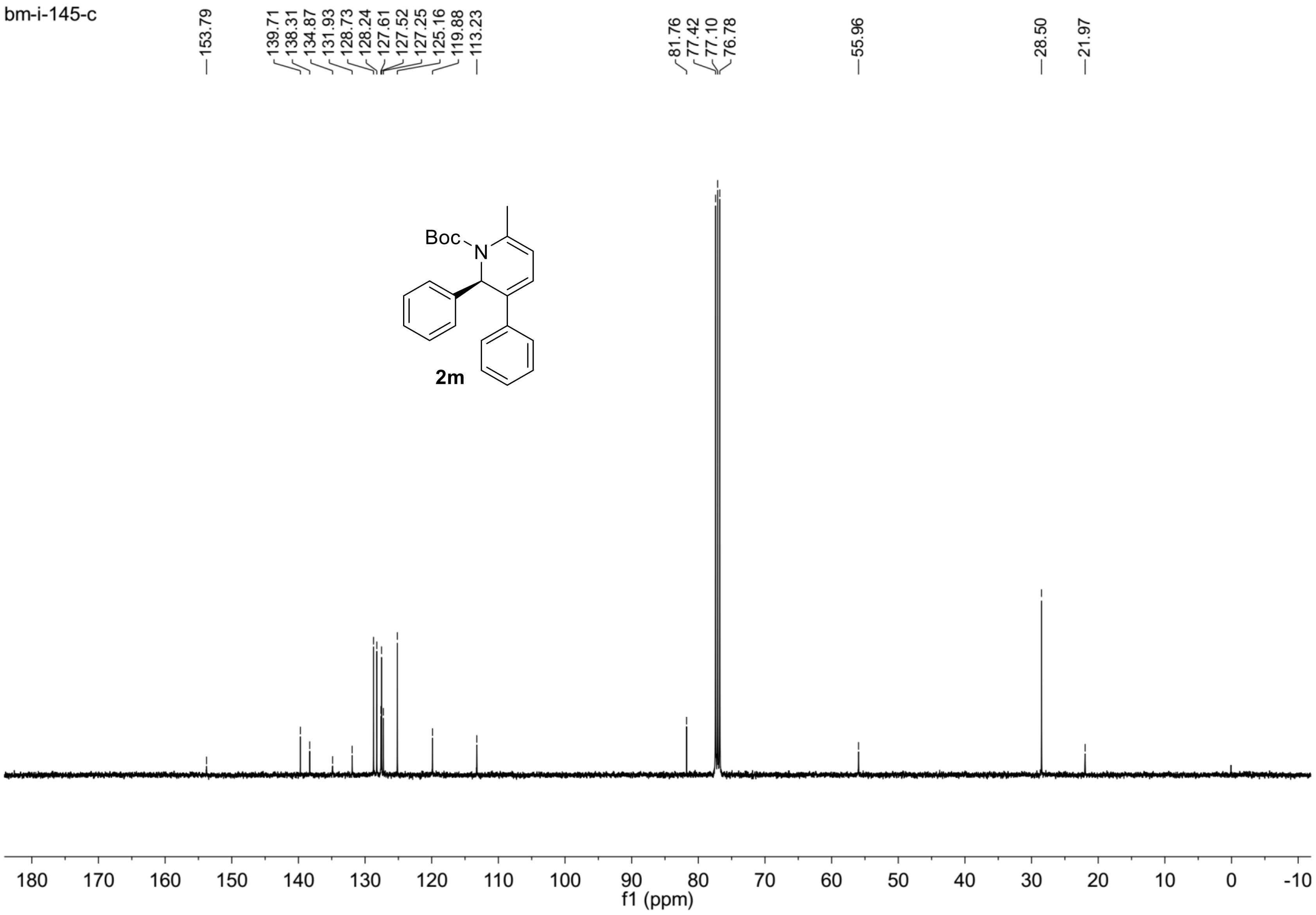

Supplementary Figure 31. ^{13}C NMR (100 MHz, $CDCl_3$) spectra for compound **2m**

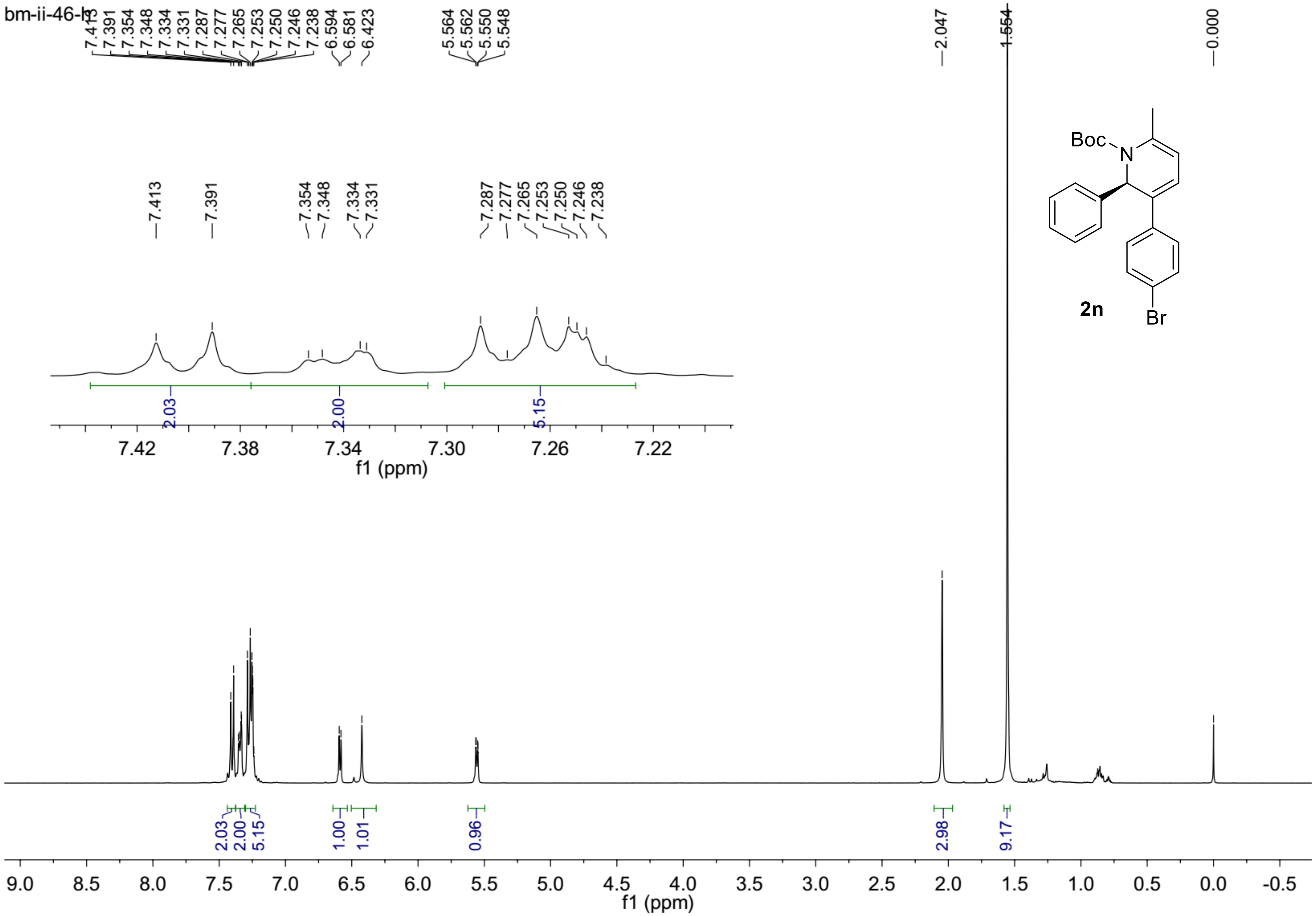

Supplementary Figure 32. ^{1}H NMR (400 MHz, $CDCl_3$) spectra for compound **2n**

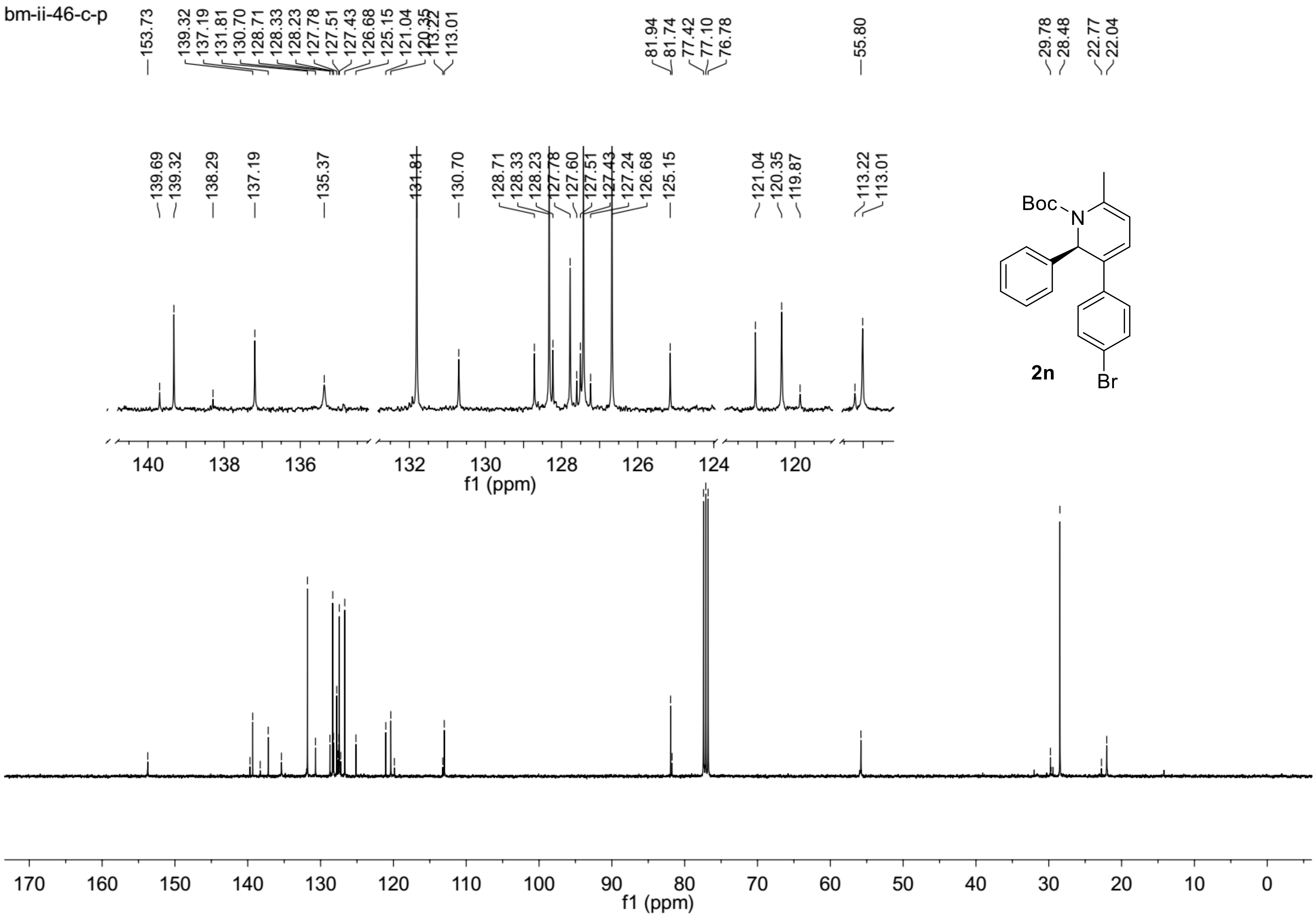

Supplementary Figure 33. ^{13}C NMR (100 MHz, $CDCl_3$) spectra for compound **2n**

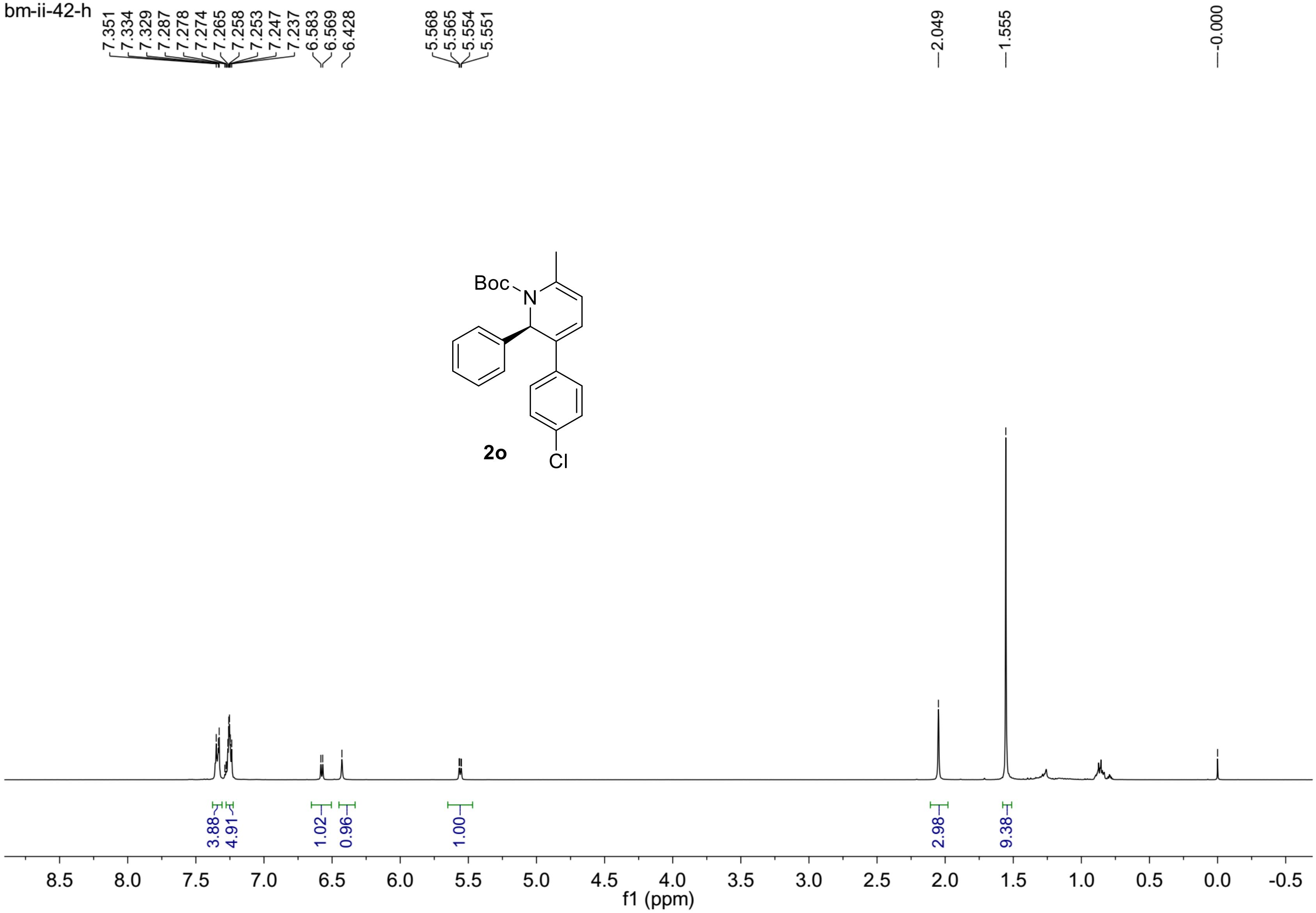

Supplementary Figure 34. ^{1}H NMR (400 MHz, $CDCl_3$) spectra for compound **2o**

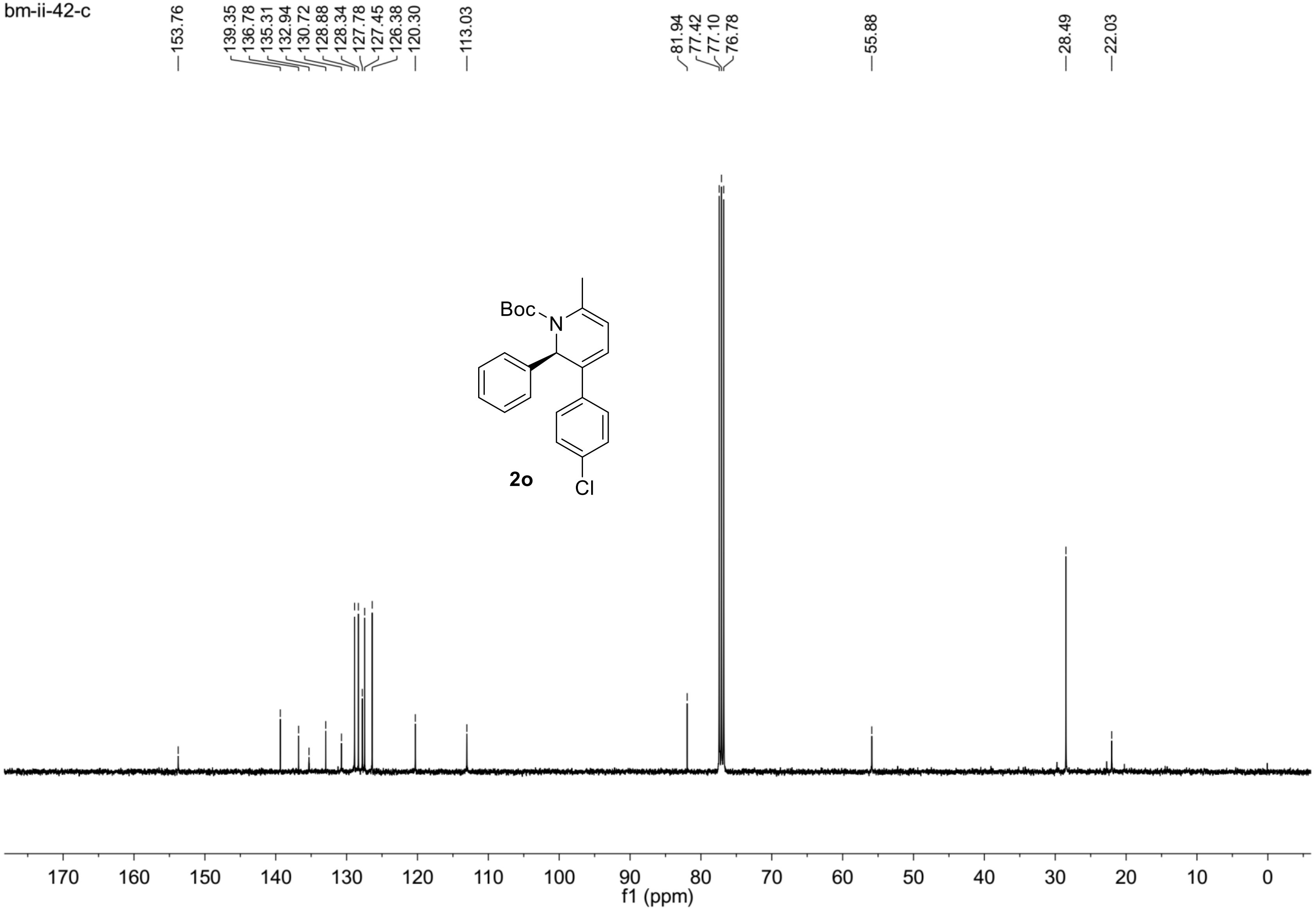

Supplementary Figure 35. ^{13}C NMR (100 MHz, $CDCl_3$) spectra for compound **2o**

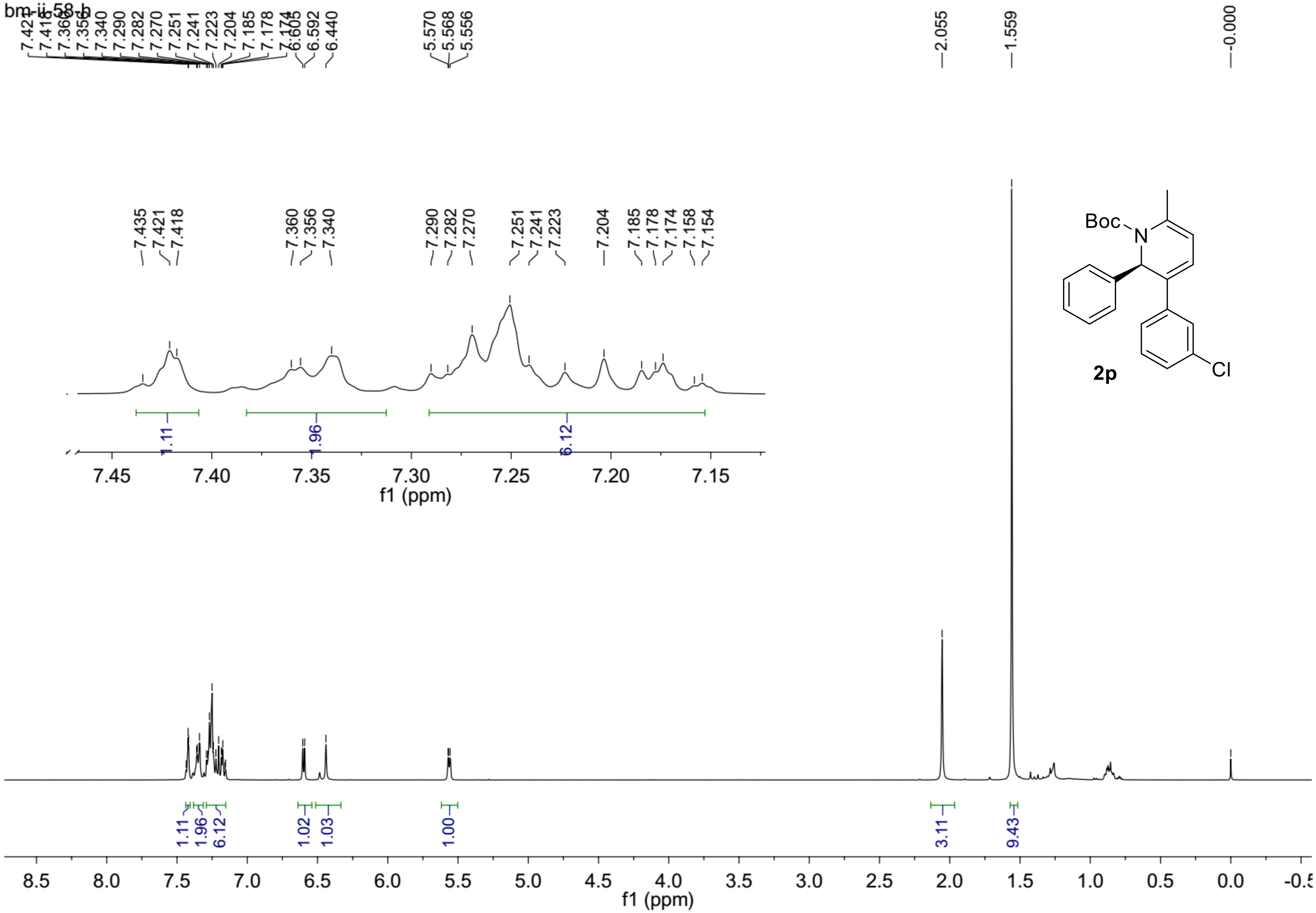

Supplementary Figure 36. ^{1}H NMR (400 MHz, $CDCl_3$) spectra for compound **2p**

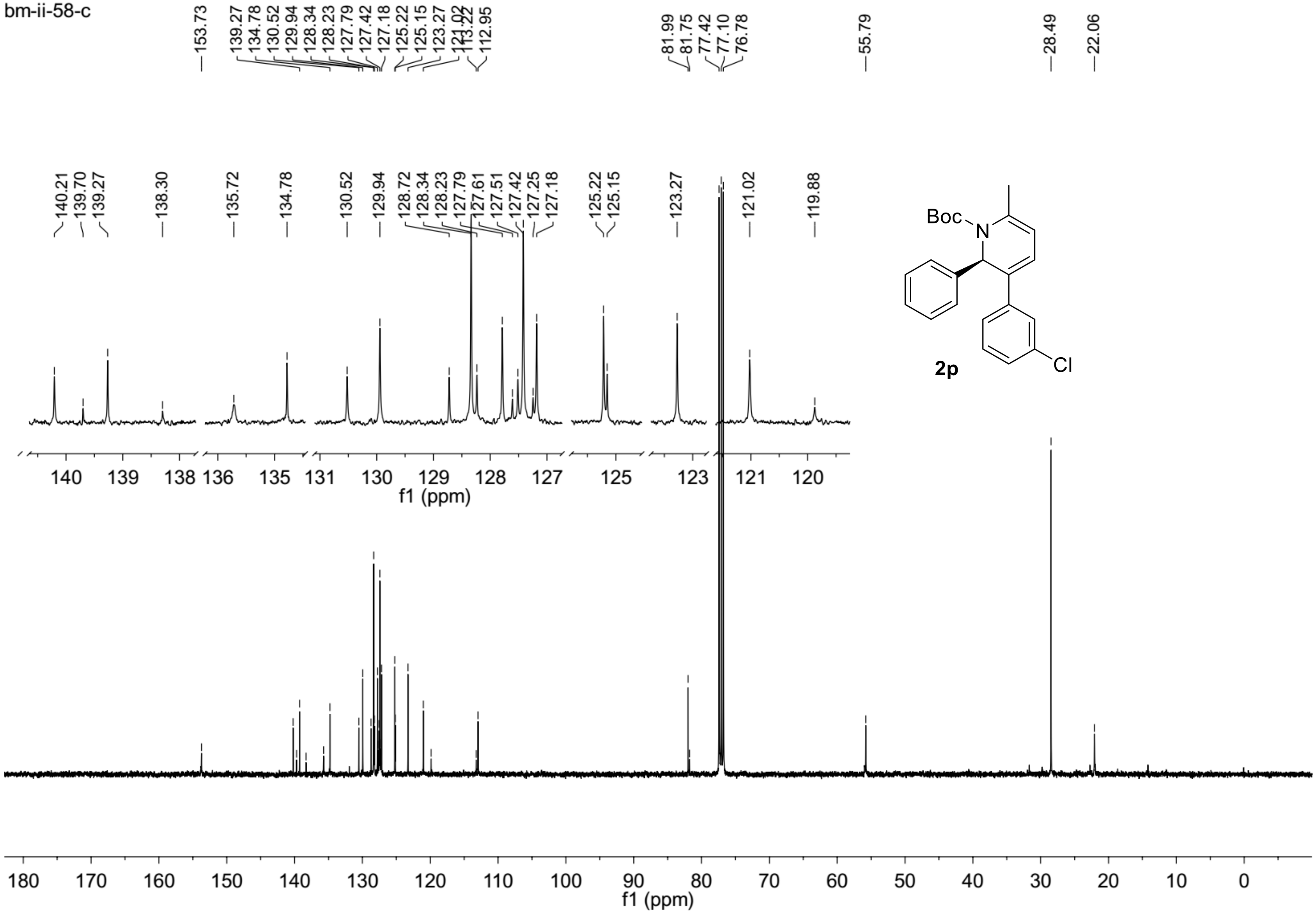

Supplementary Figure 37. ^{13}C NMR (100 MHz, $CDCl_3$) spectra for compound **2p**

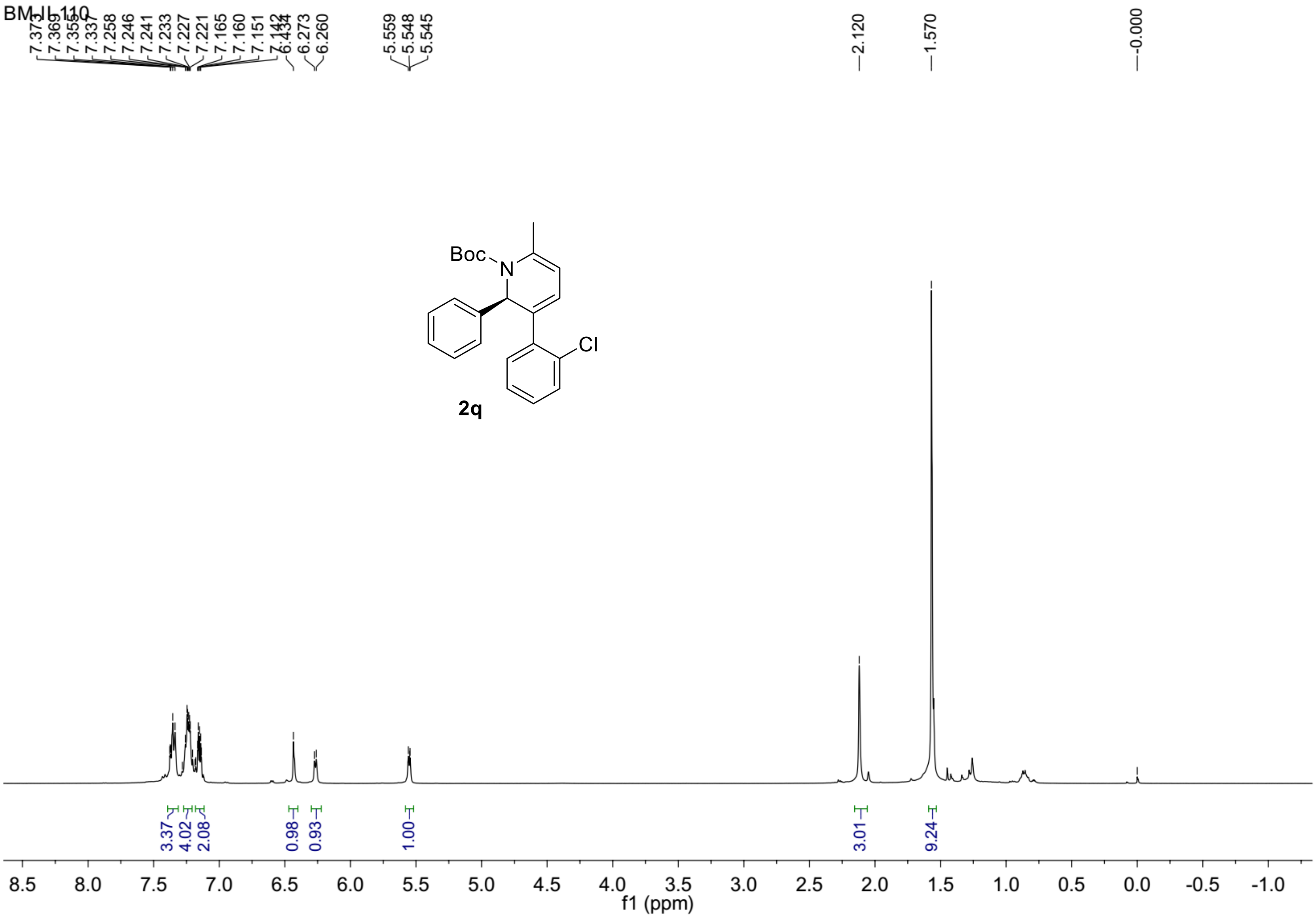

Supplementary Figure 38. ^{1}H NMR (400 MHz, $CDCl_3$) spectra for compound **2q**

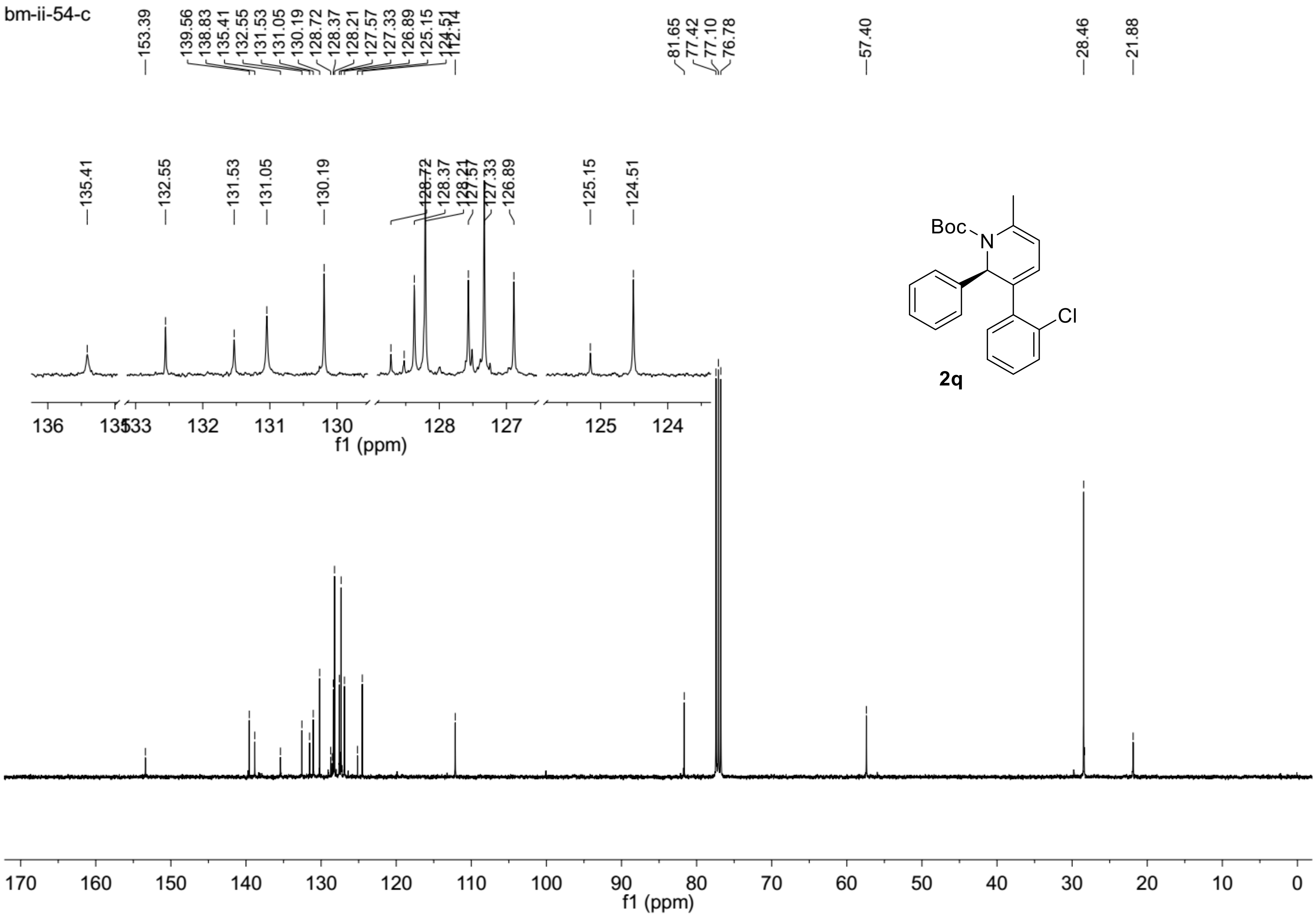

Supplementary Figure 39. ^{13}C NMR (100 MHz, $CDCl_3$) spectra for compound **2q**

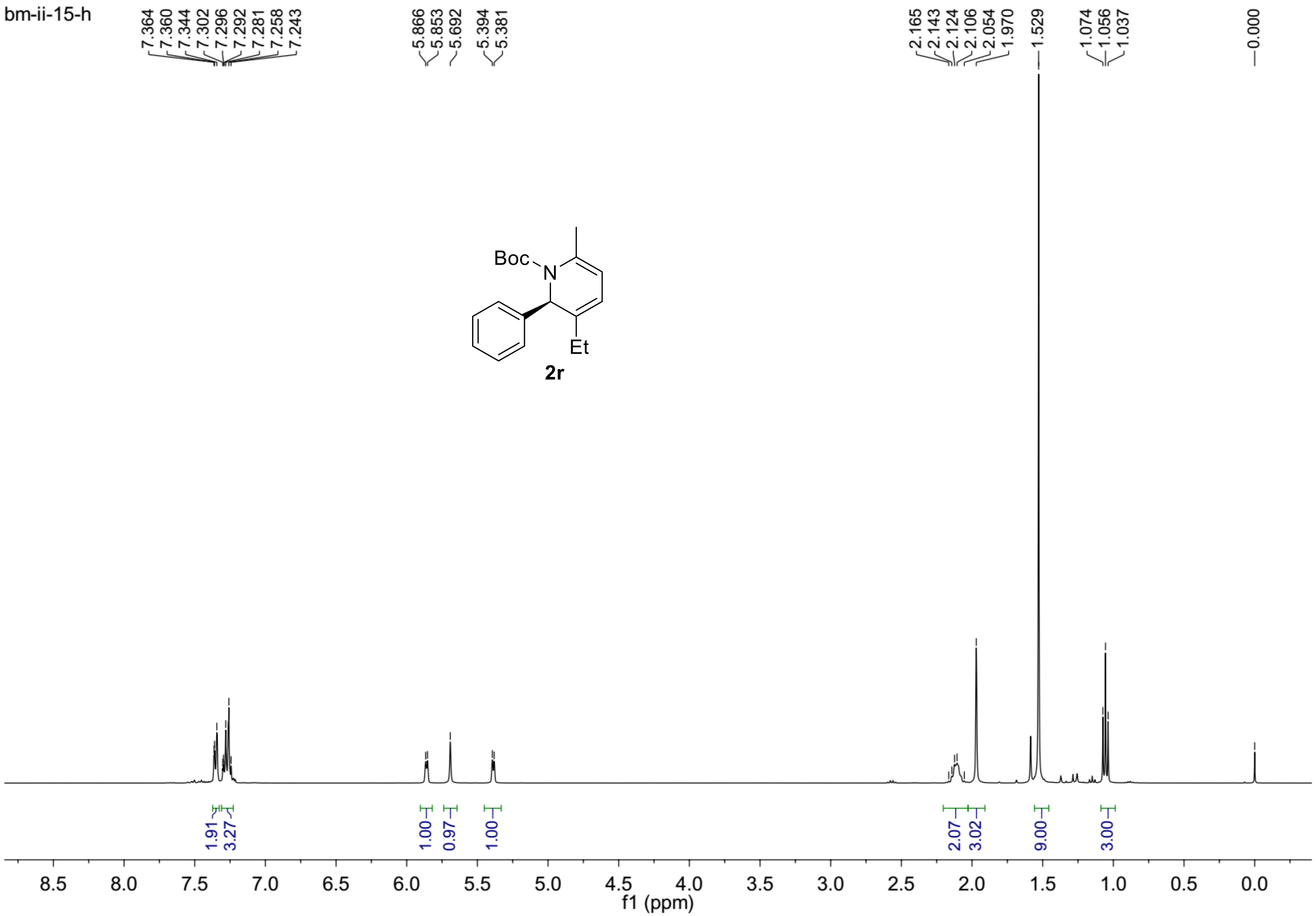

Supplementary Figure 40. ^{1}H NMR (400 MHz, $CDCl_3$) spectra for compound **2r**

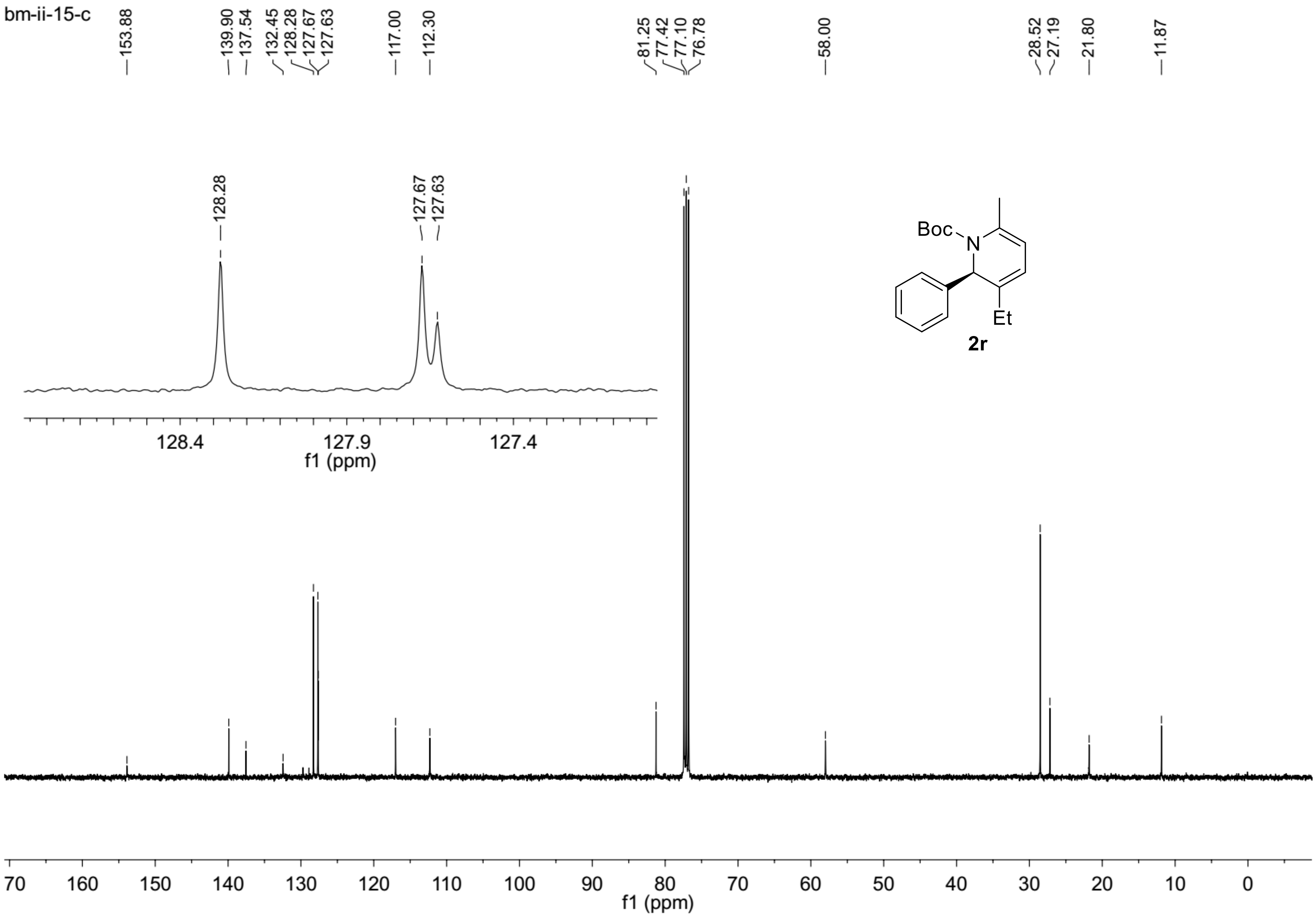

Supplementary Figure 41. ^{13}C NMR (100 MHz, $CDCl_3$) spectra for compound **2r**

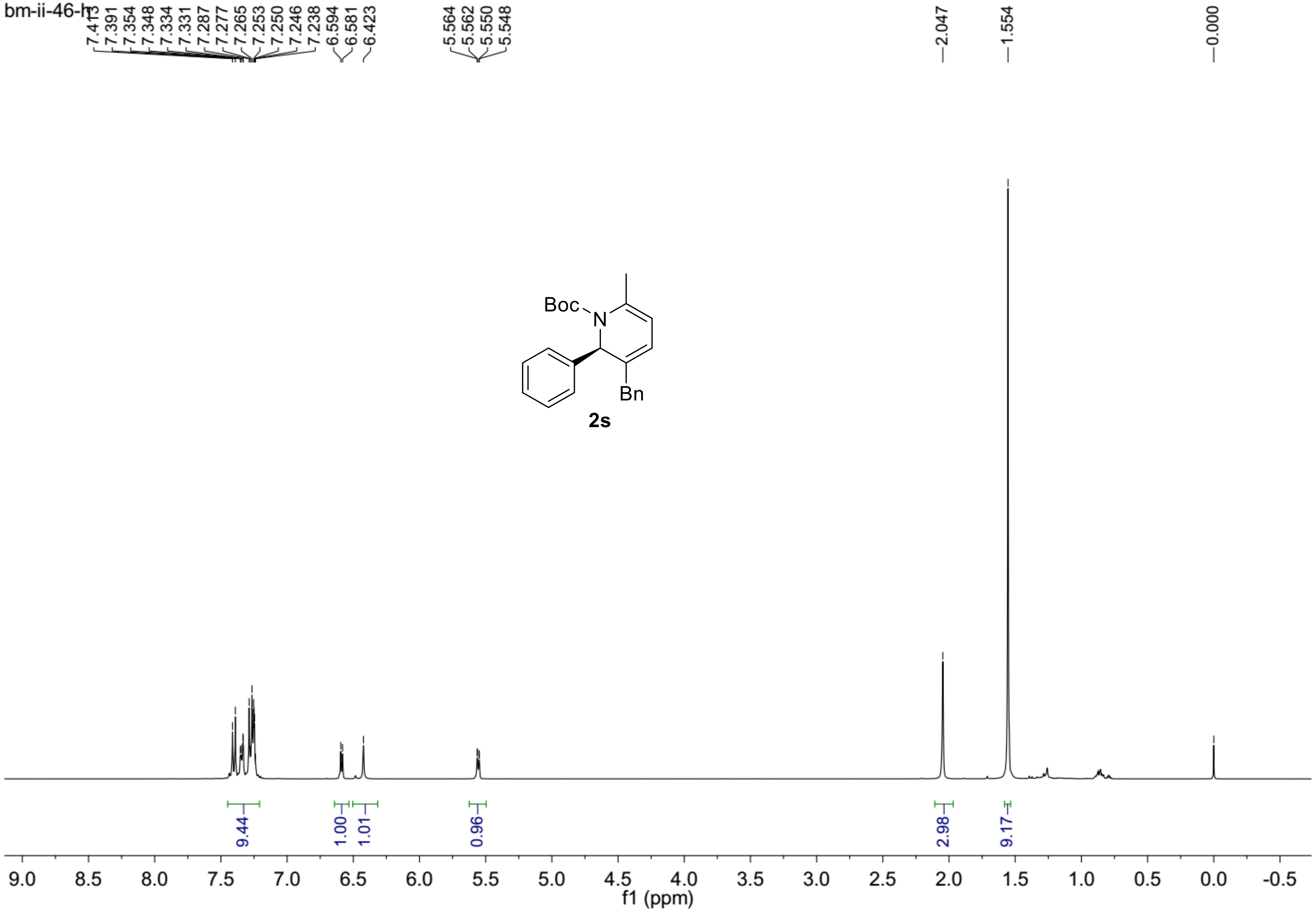

Supplementary Figure 42. ^{1}H NMR (400 MHz, $CDCl_3$) spectra for compound **2s**

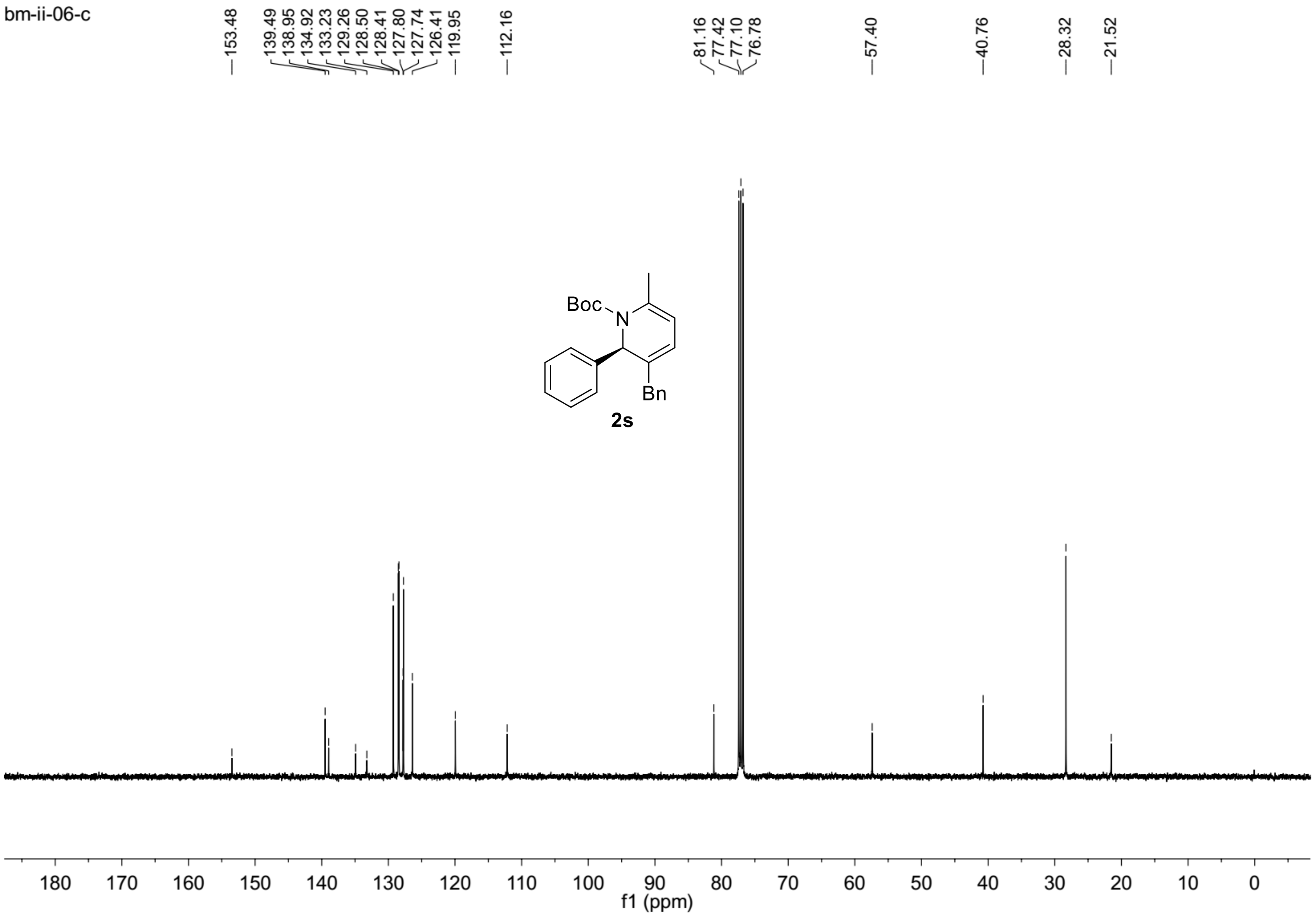

Supplementary Figure 43. ^{13}C NMR (100 MHz, $CDCl_3$) spectra for compound **2s**

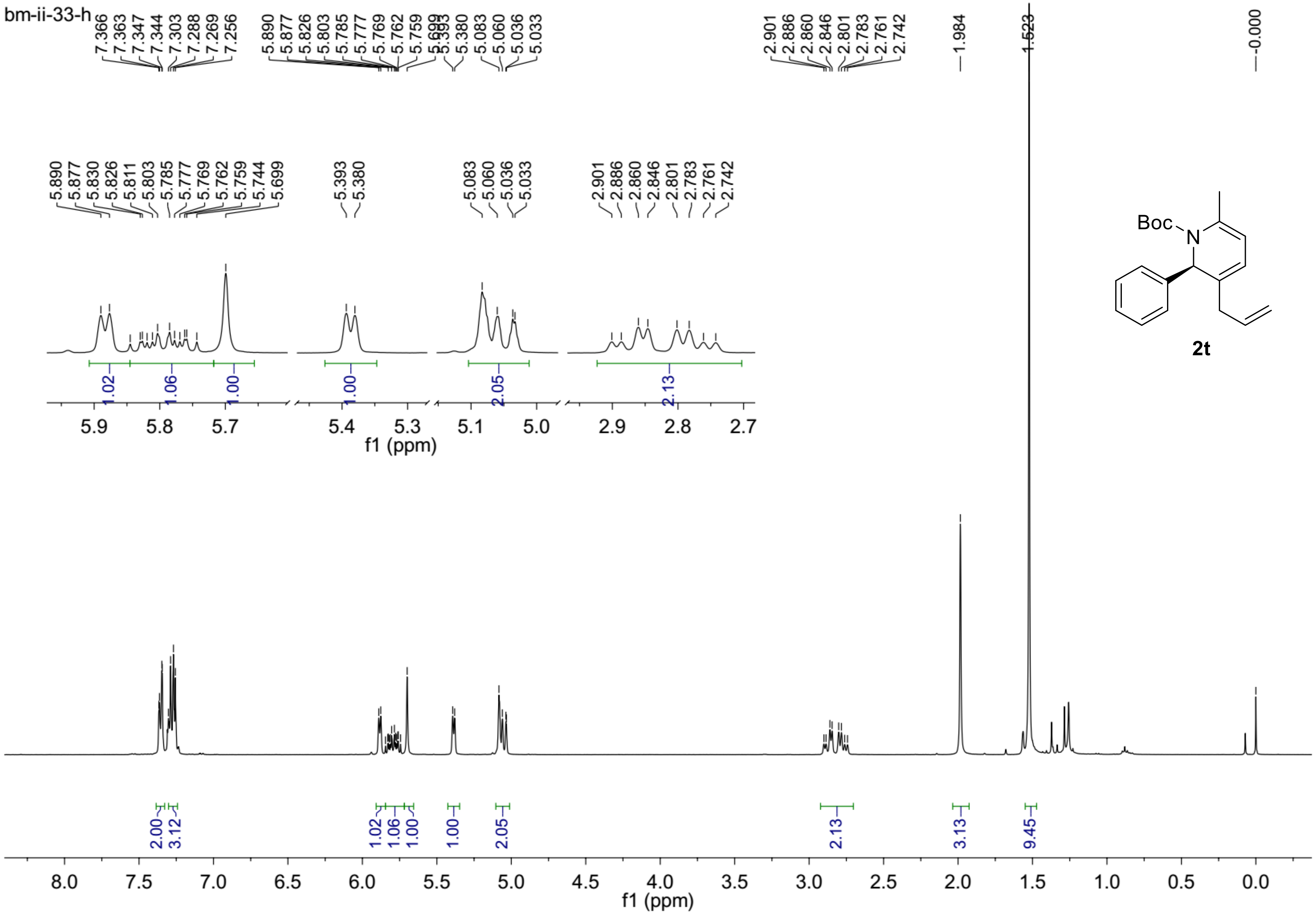

Supplementary Figure 44. ^{1}H NMR (400 MHz, $CDCl_3$) spectra for compound **2t**

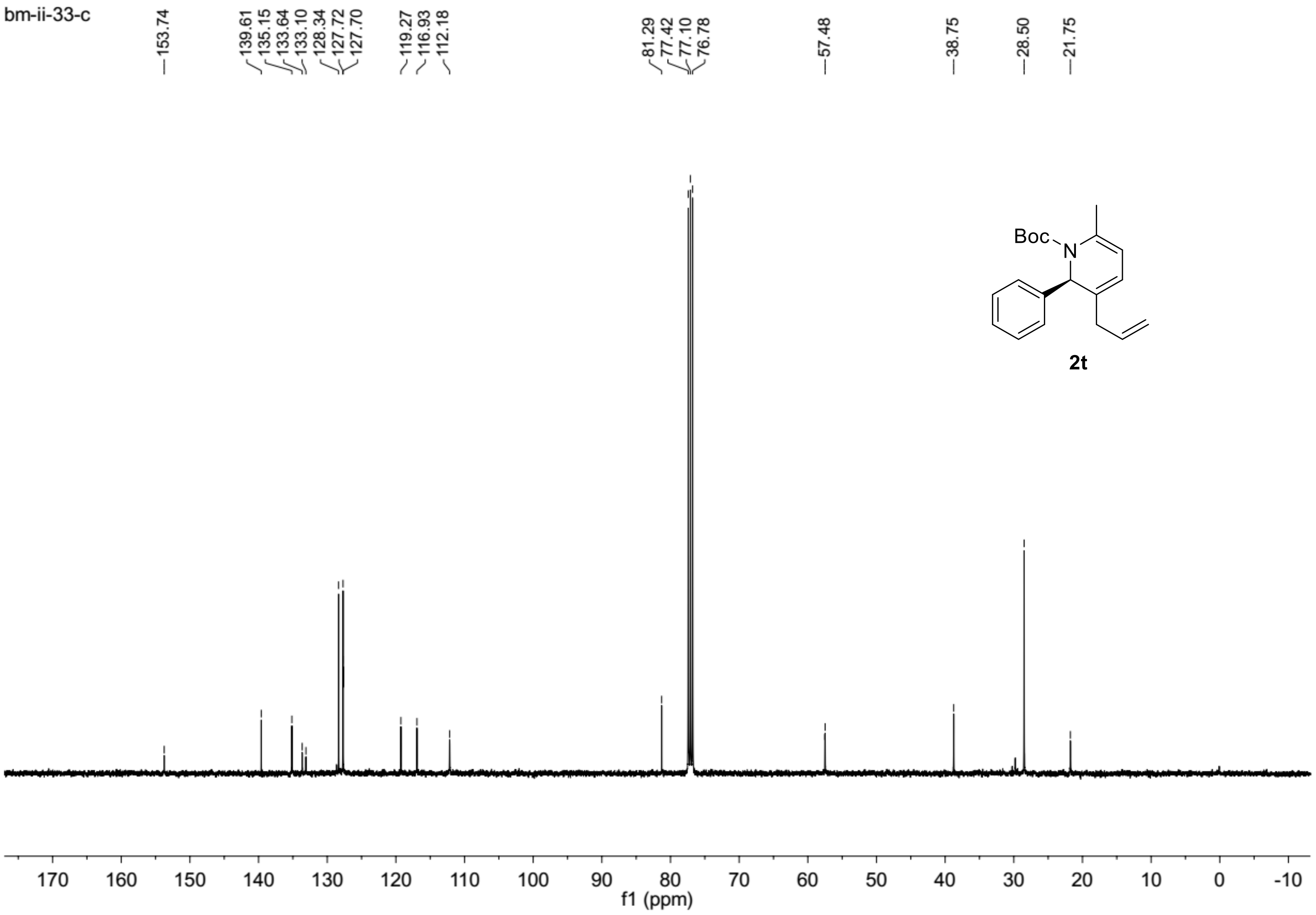

Supplementary Figure 45. ^{13}C NMR (100 MHz, $CDCl_3$) spectra for compound **2t**

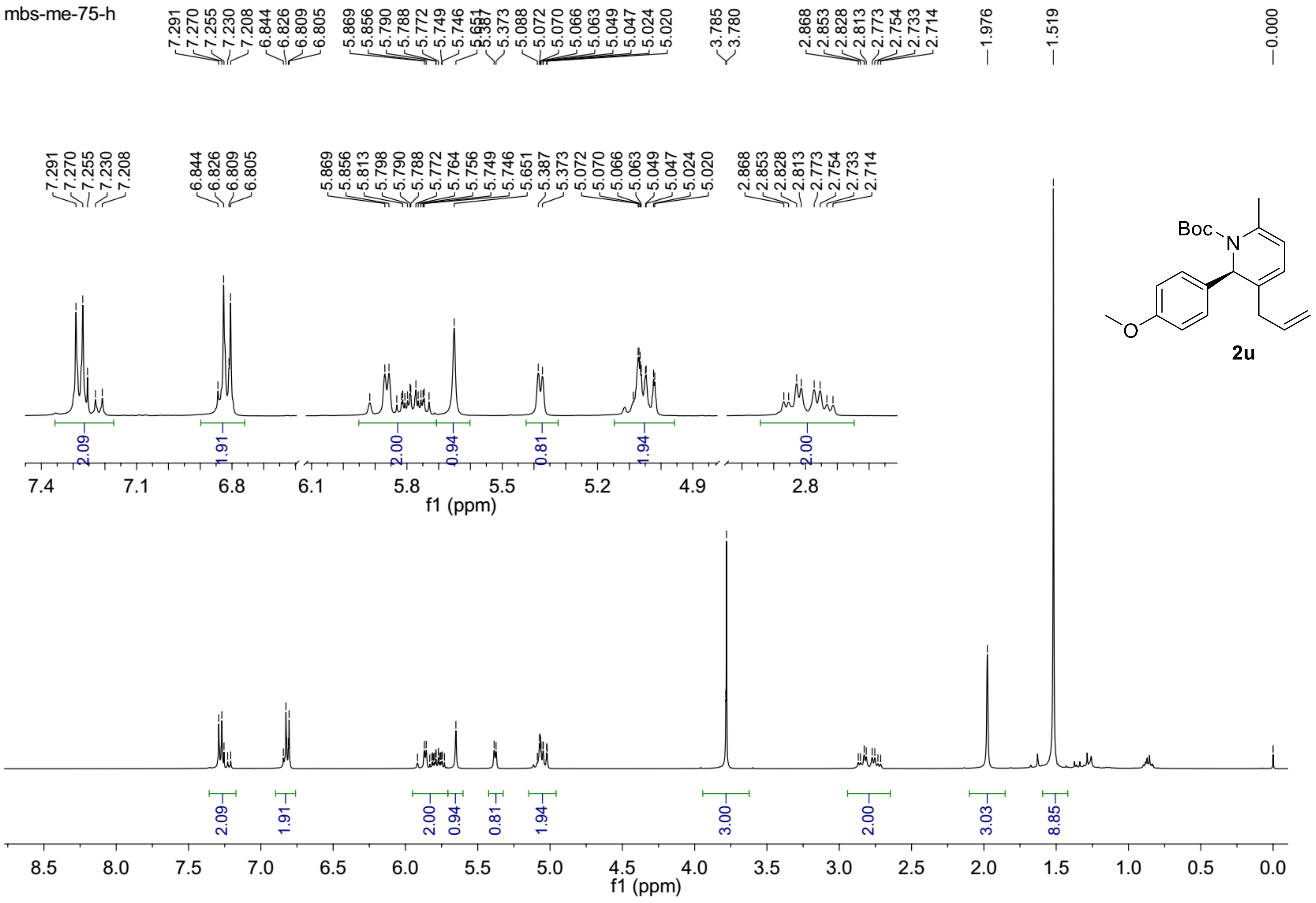

Supplementary Figure 46. ^{1}H NMR (400 MHz, $CDCl_3$) spectra for compound **2u**

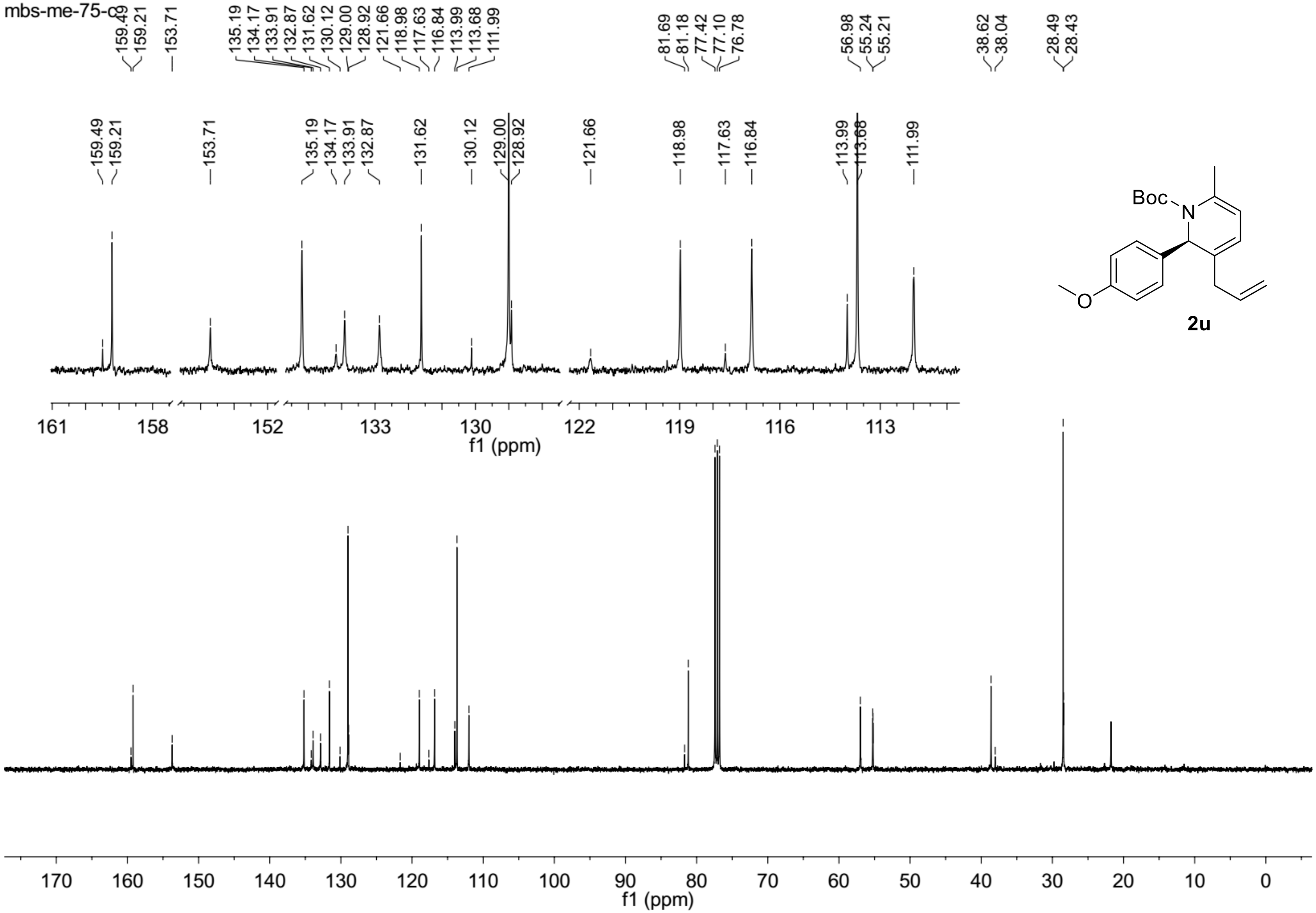

Supplementary Figure 47. ^{13}C NMR (100 MHz, $CDCl_3$) spectra for compound **2u**

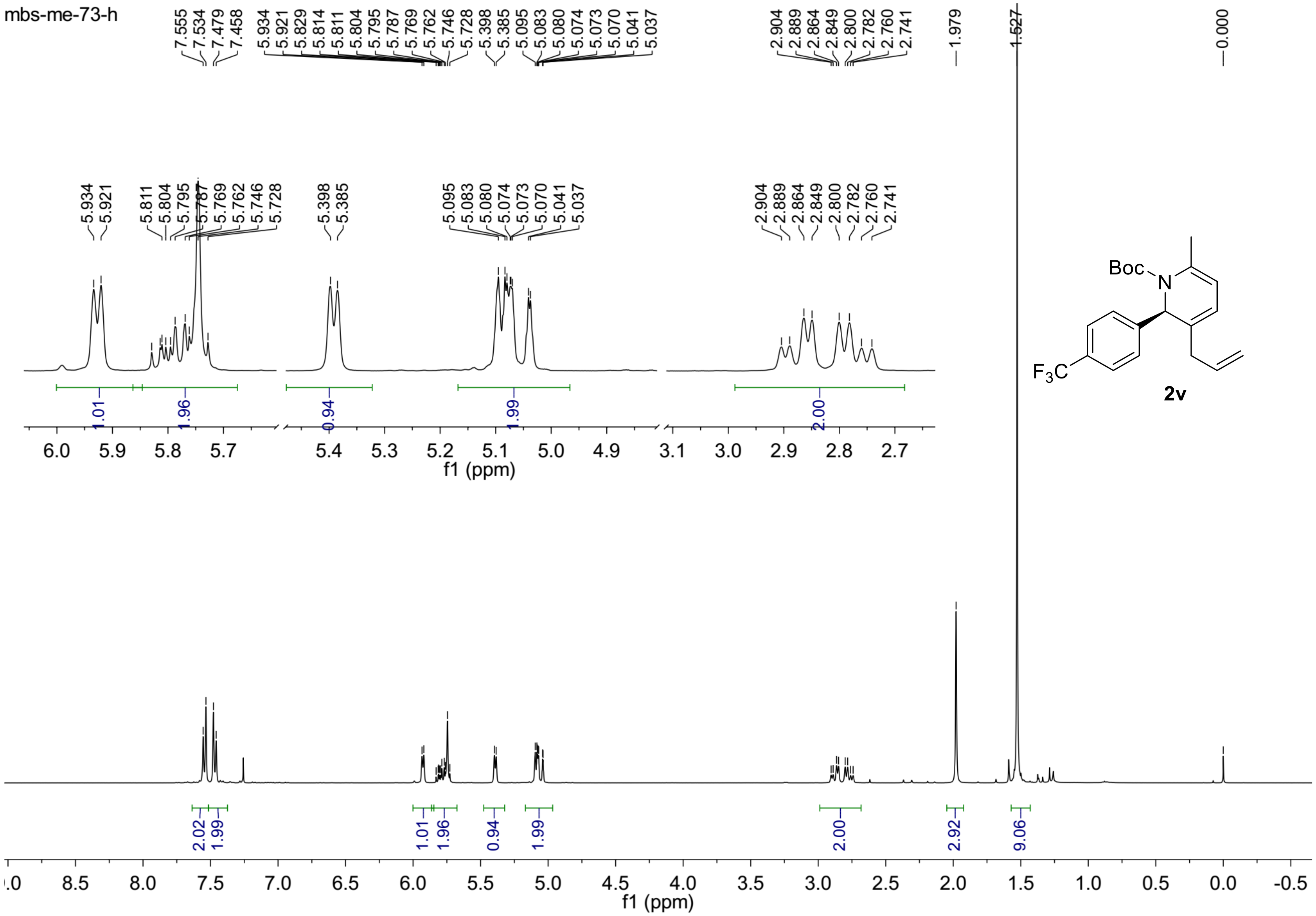

Supplementary Figure 48. ^{1}H NMR (400 MHz, $CDCl_3$) spectra for compound **2v**

mbs-me-73-f

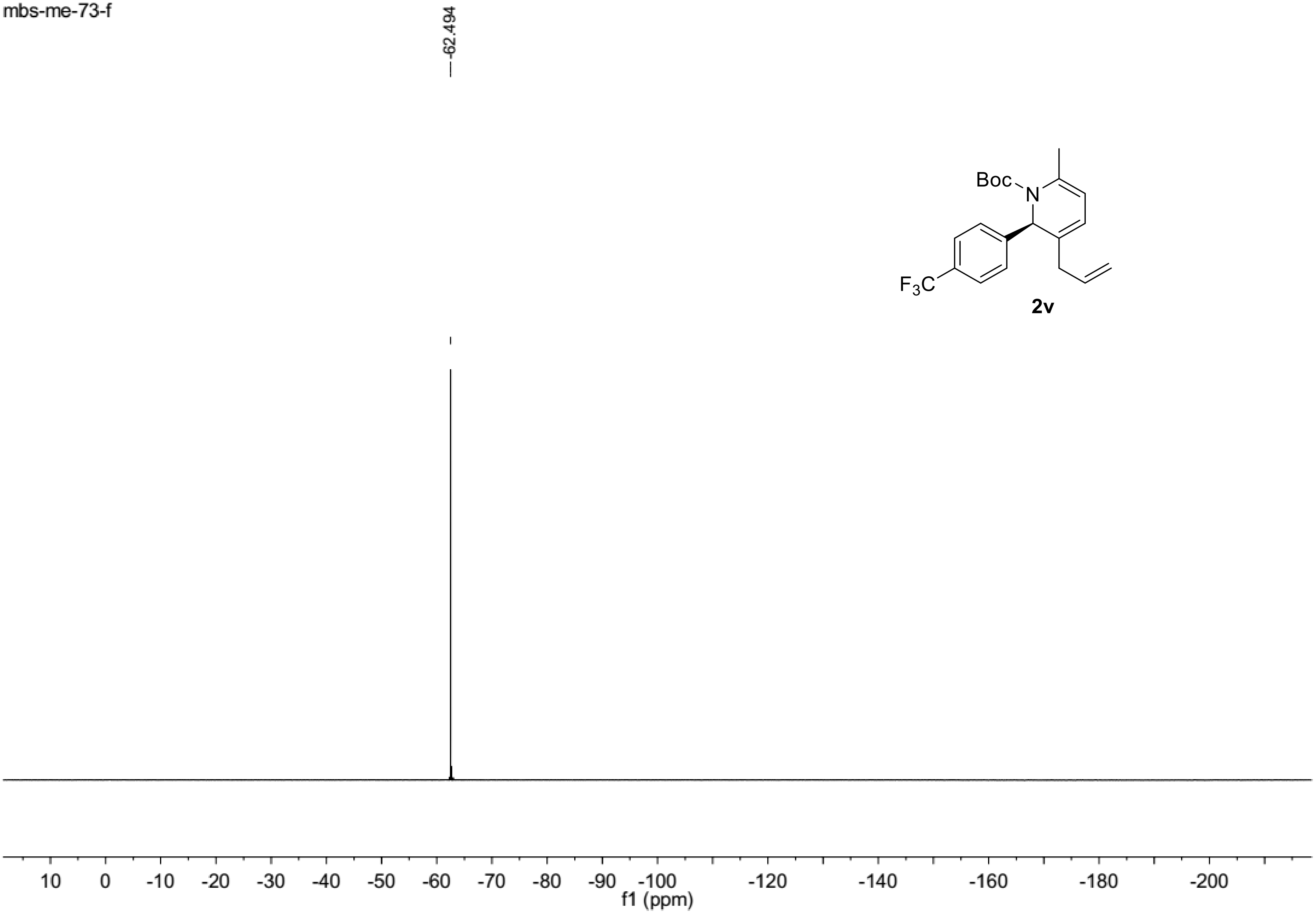

2v

Supplementary Figure 49. ^{19}F NMR (376 MHz, $CDCl_3$) spectra for compound **2v**

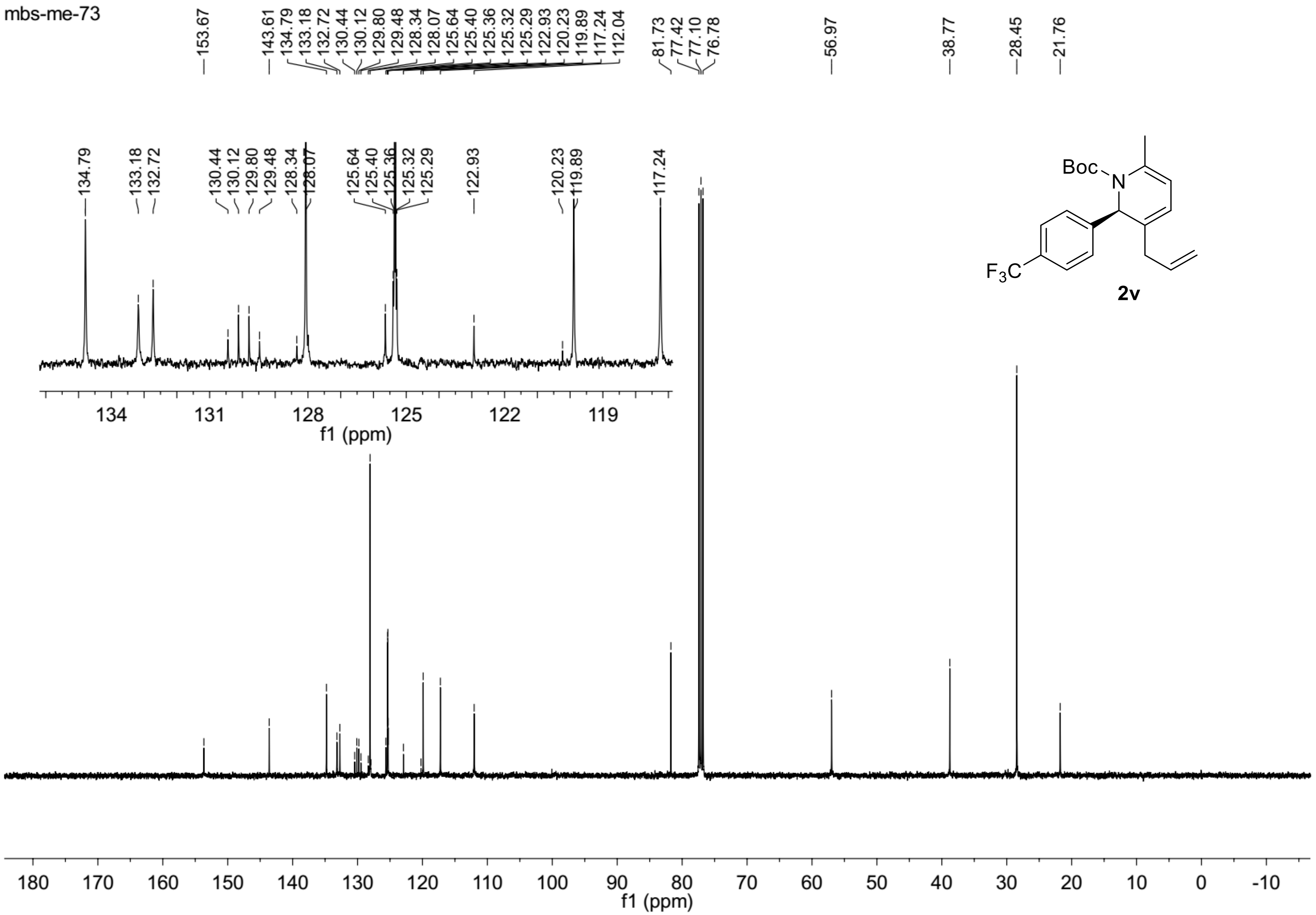

Supplementary Figure 50. ^{13}C NMR (100 MHz, $CDCl_3$) spectra for compound **2v**

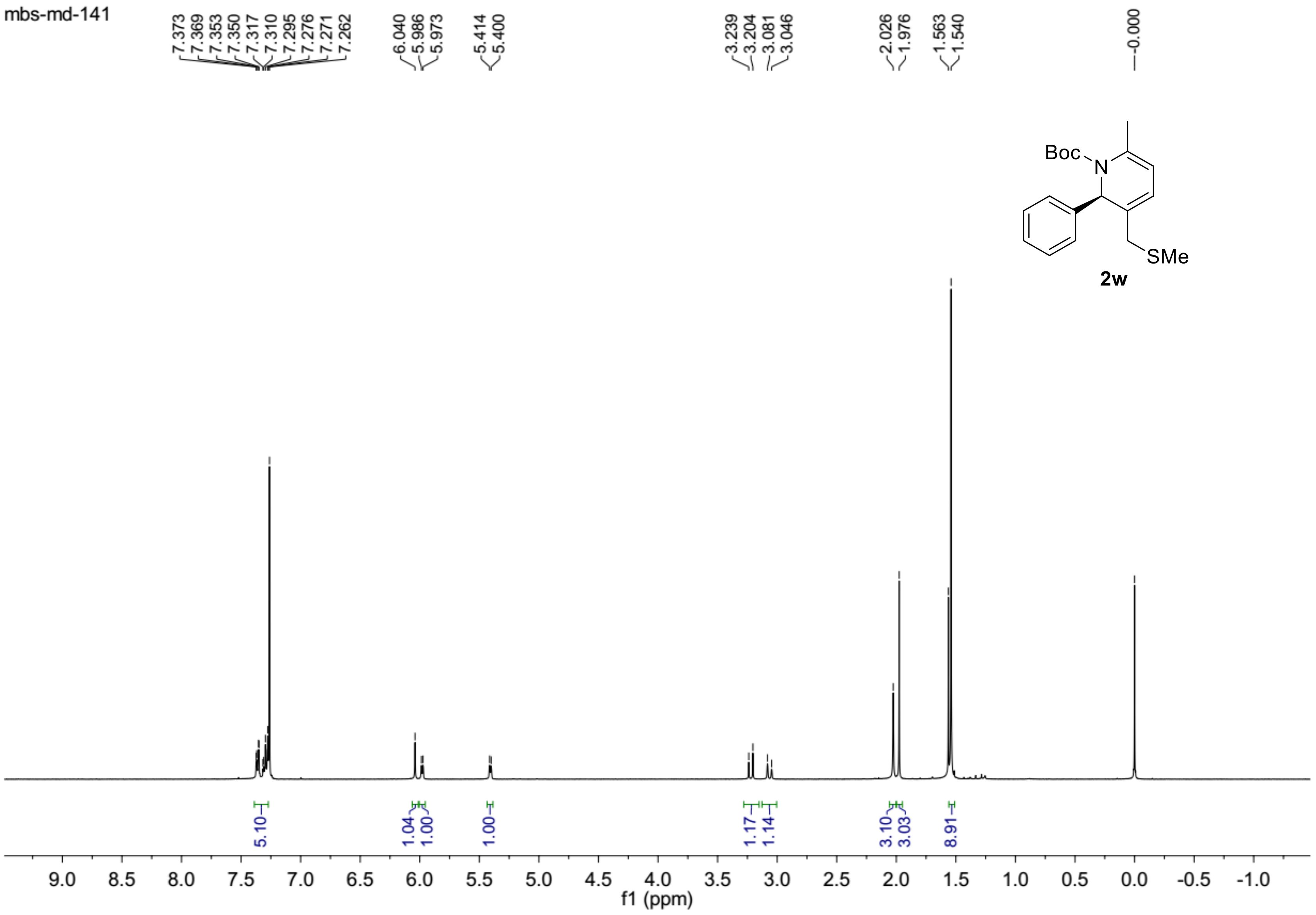

Supplementary Figure 51. ^{1}H NMR (400 MHz, $CDCl_3$) spectra for compound **2w**

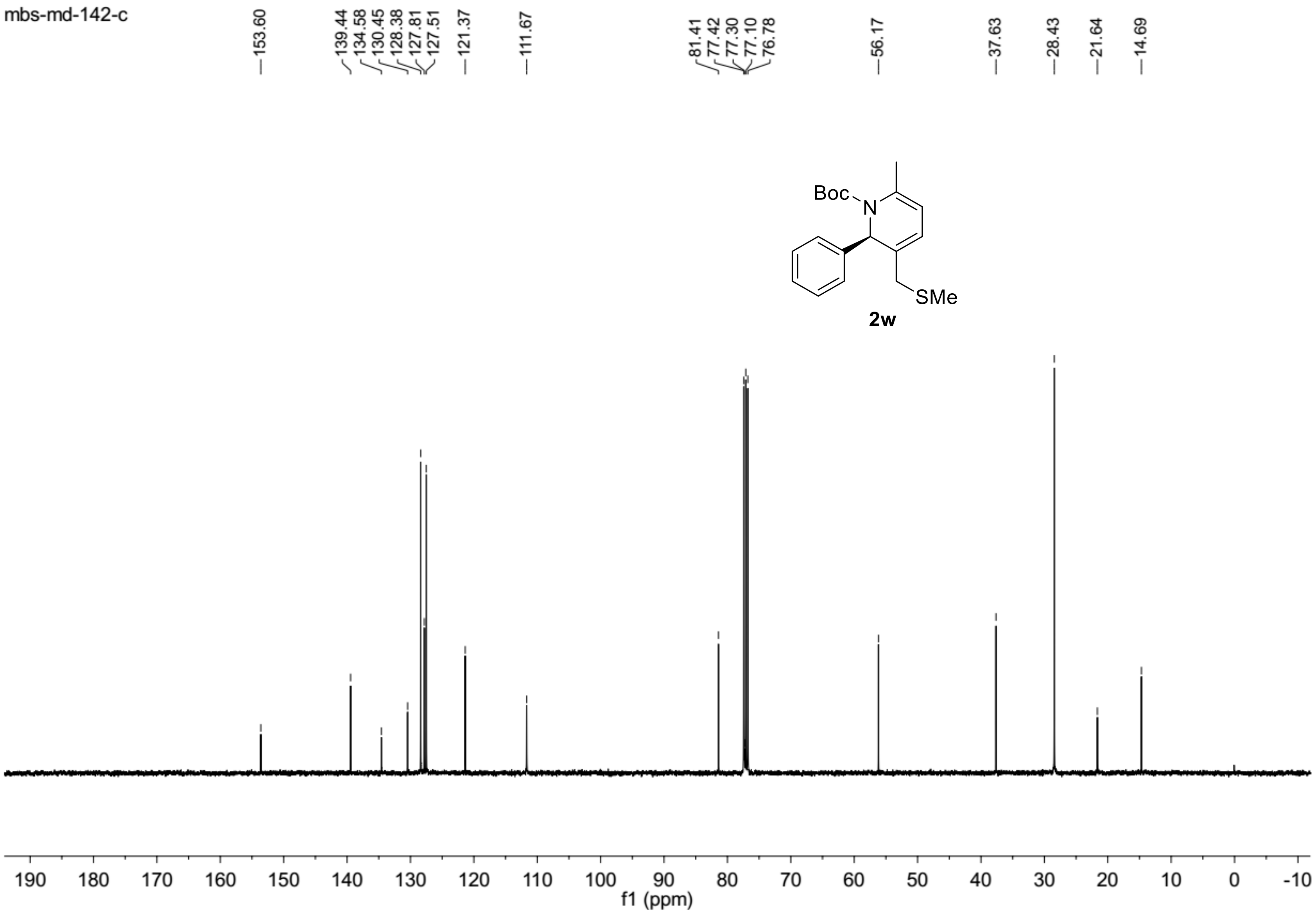

Supplementary Figure 52. ^{13}C NMR (100 MHz, $CDCl_3$) spectra for compound **2w**

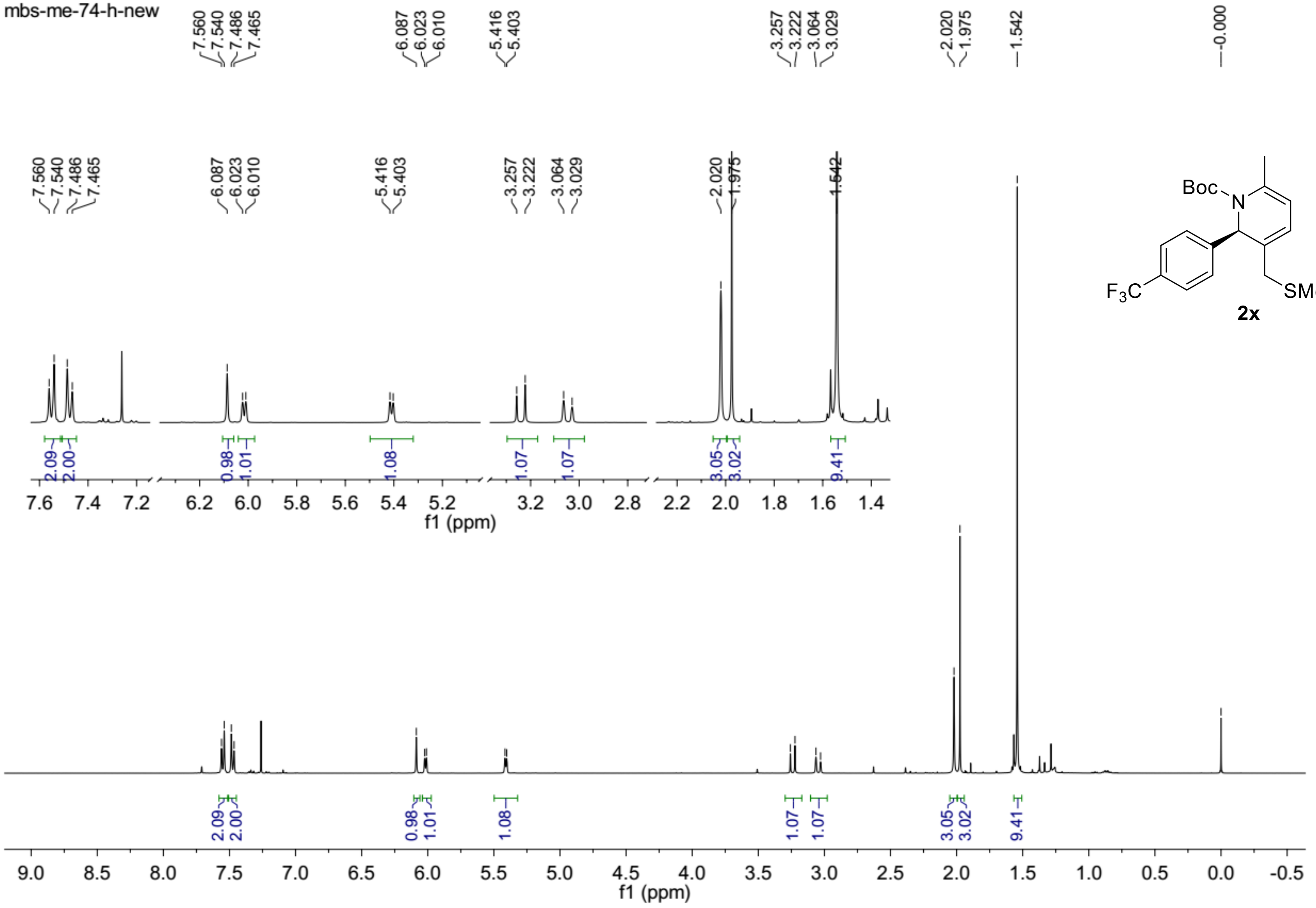

Supplementary Figure 53. ^{1}H NMR (400 MHz, $CDCl_3$) spectra for compound **2x**

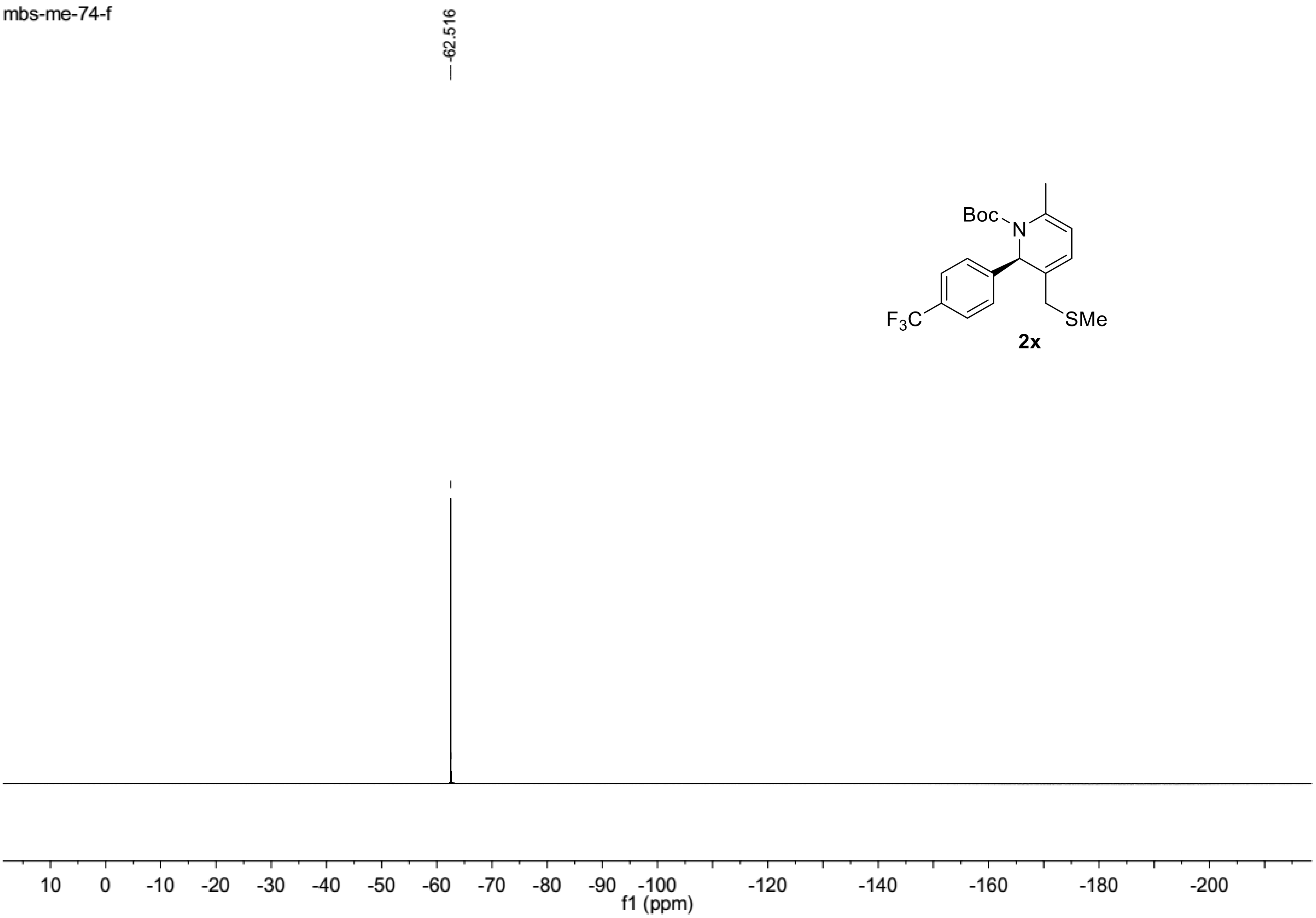

Supplementary Figure 54. ^{19}F NMR (376 MHz, $CDCl_3$) spectra for compound **2x**

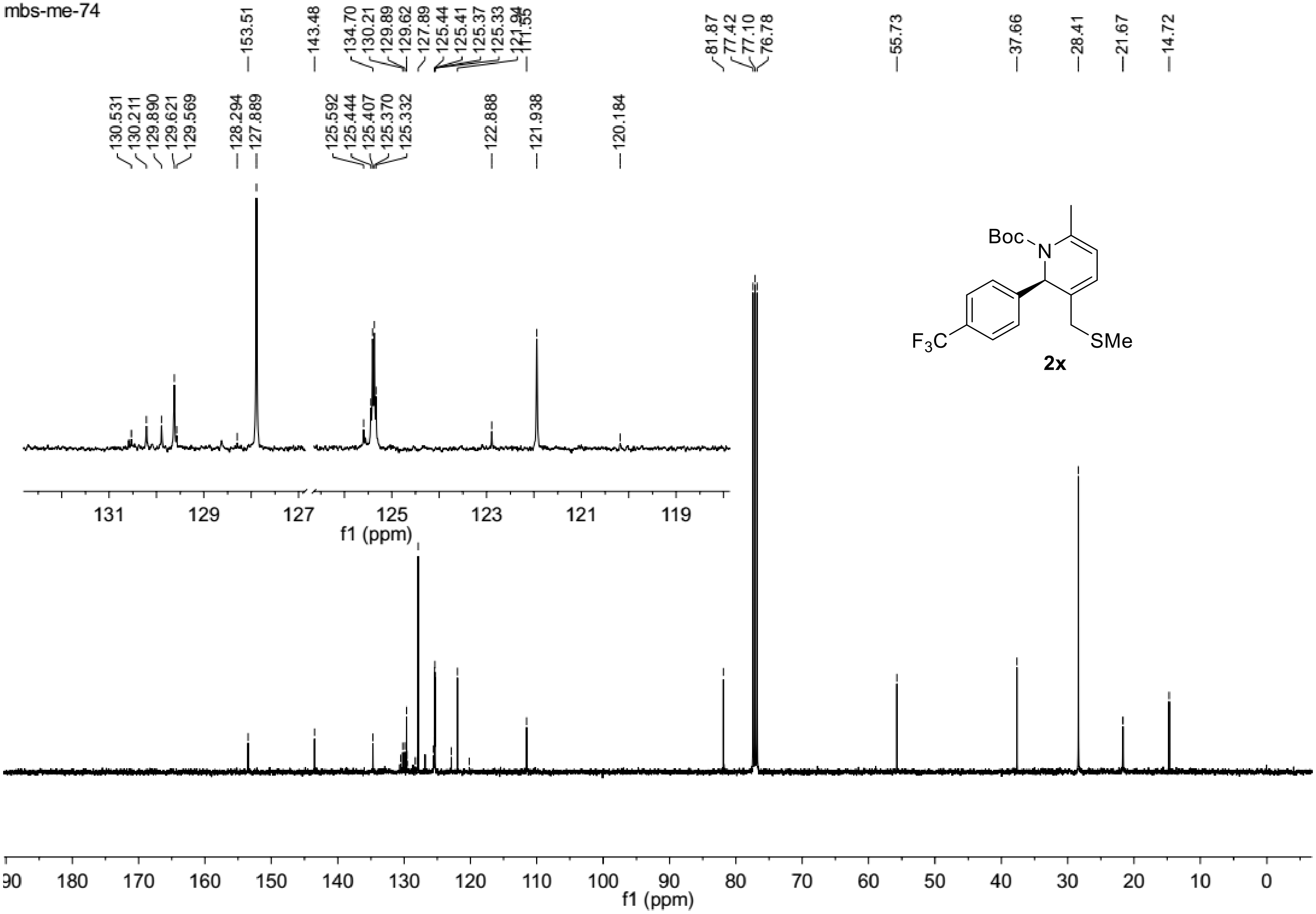

Supplementary Figure 55. ^{13}C NMR (100 MHz, $CDCl_3$) spectra for compound **2x**

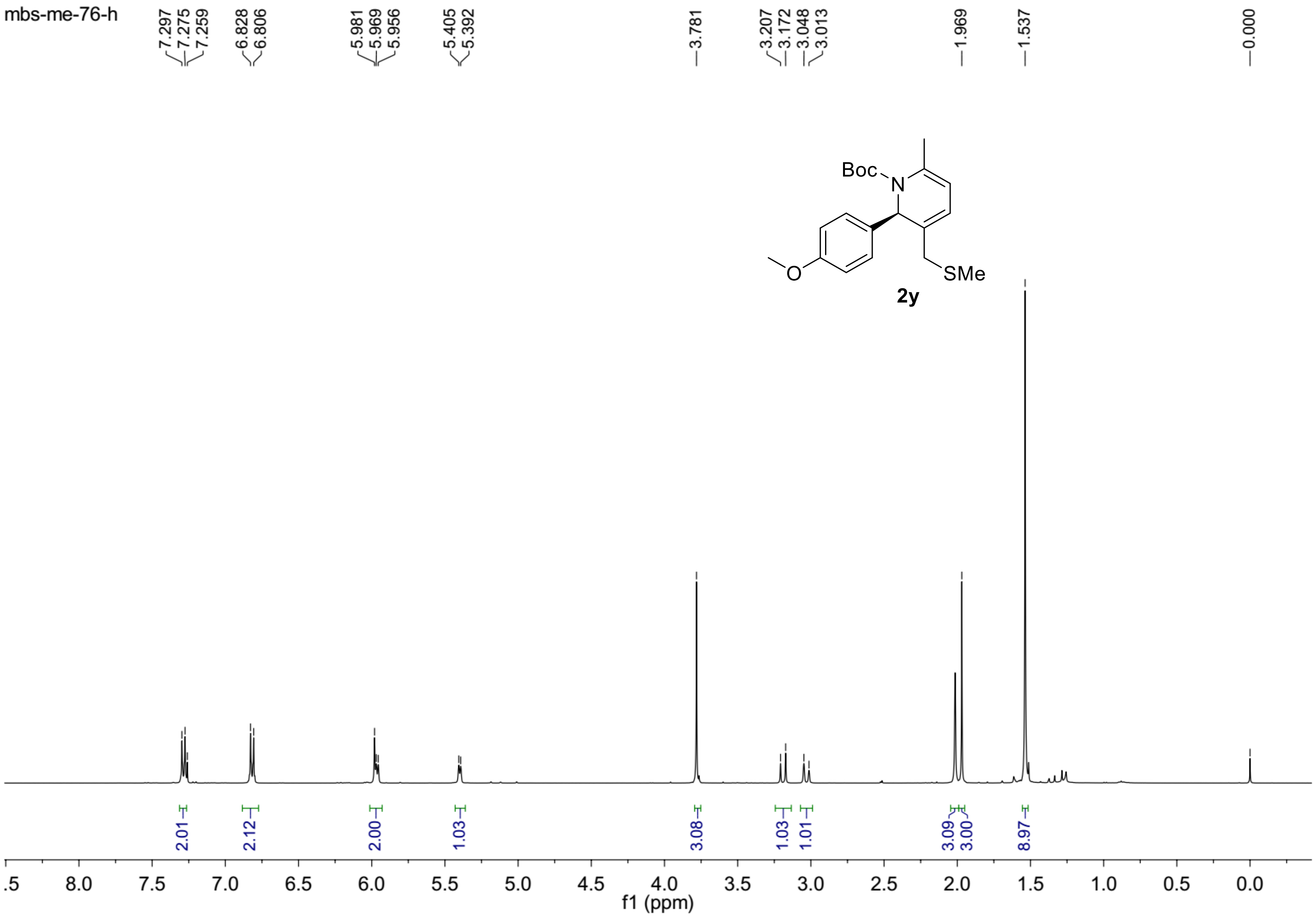

Supplementary Figure 56. ^{1}H NMR (400 MHz, $CDCl_3$) spectra for compound **2y**

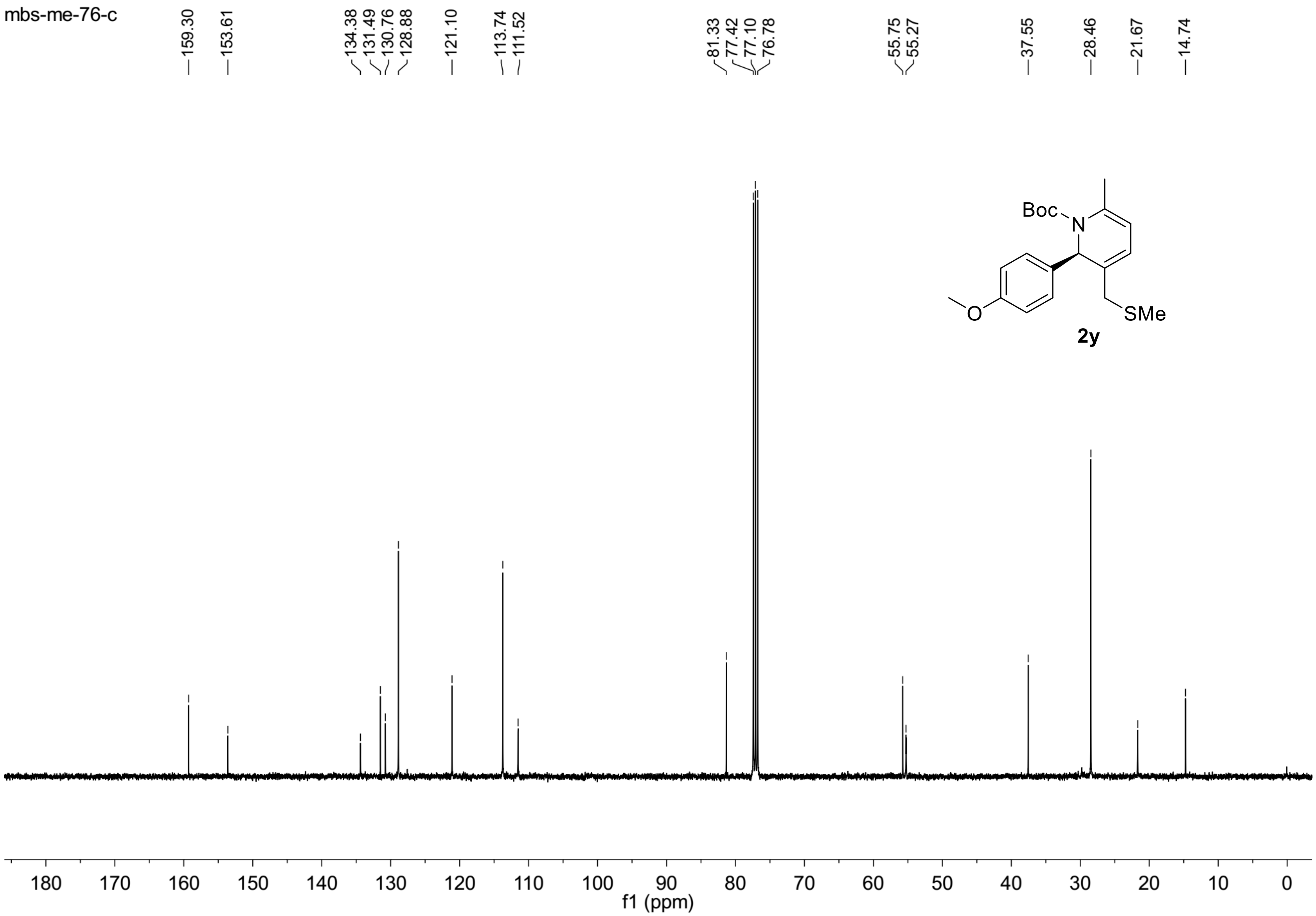

Supplementary Figure 57. ^{13}C NMR (100 MHz, $CDCl_3$) spectra for compound **2y**

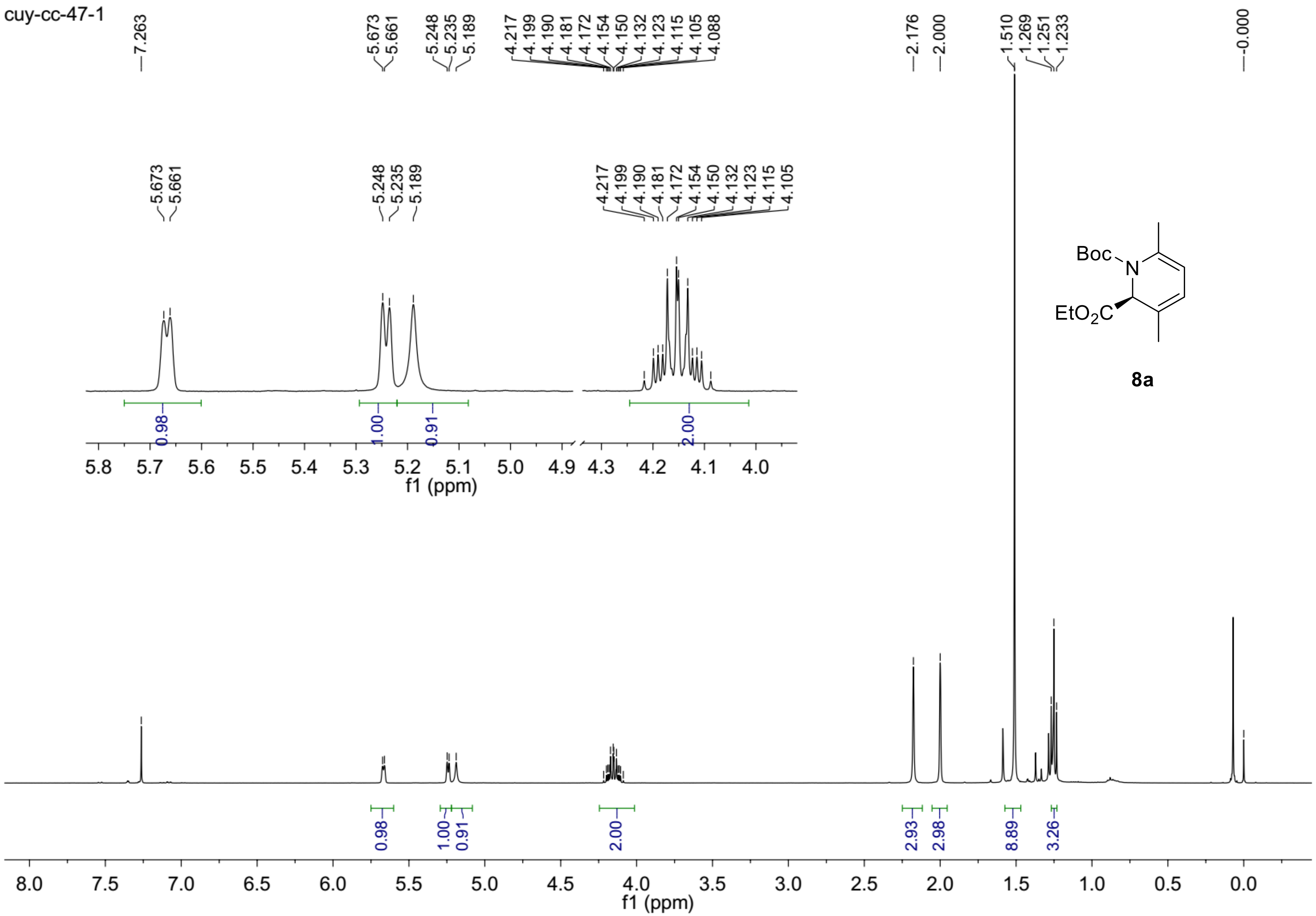

Supplementary Figure 58. ^{1}H NMR (400 MHz, $CDCl_3$) spectra for compound **8a**

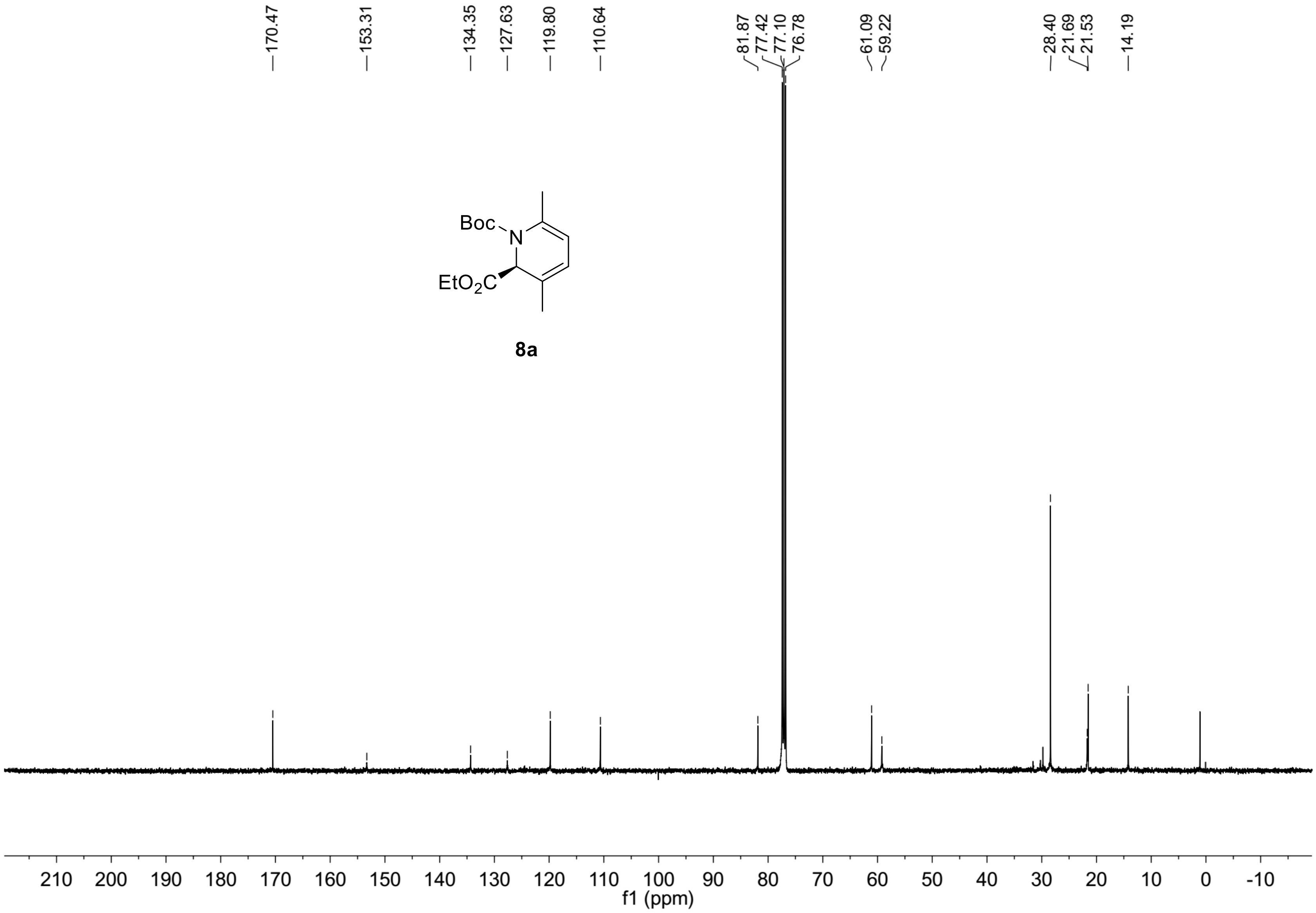

Supplementary Figure 59. ^{13}C NMR (100 MHz, $CDCl_3$) spectra for compound **8a**

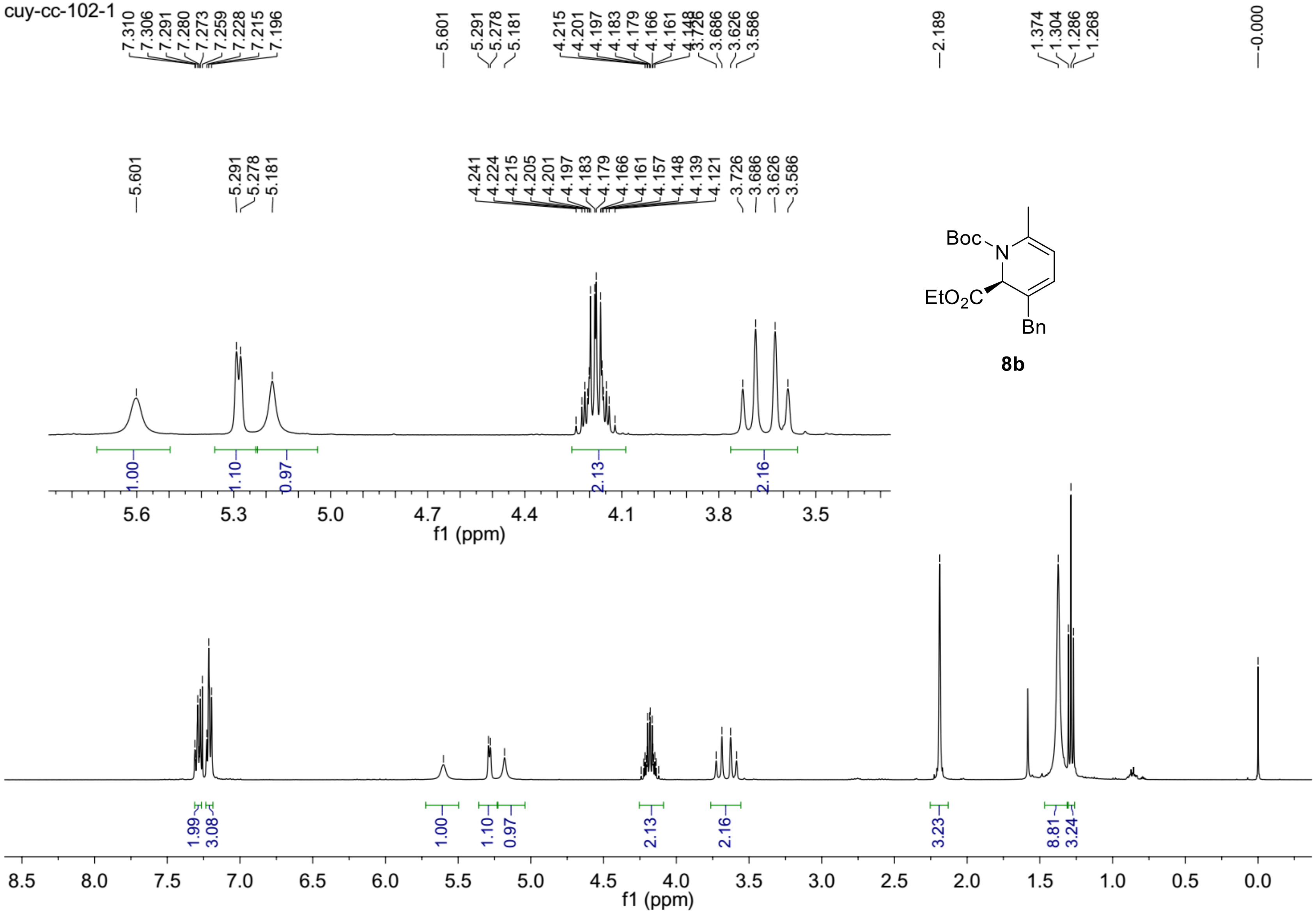

Supplementary Figure 60. ^{1}H NMR (400 MHz, $CDCl_3$) spectra for compound **8b**

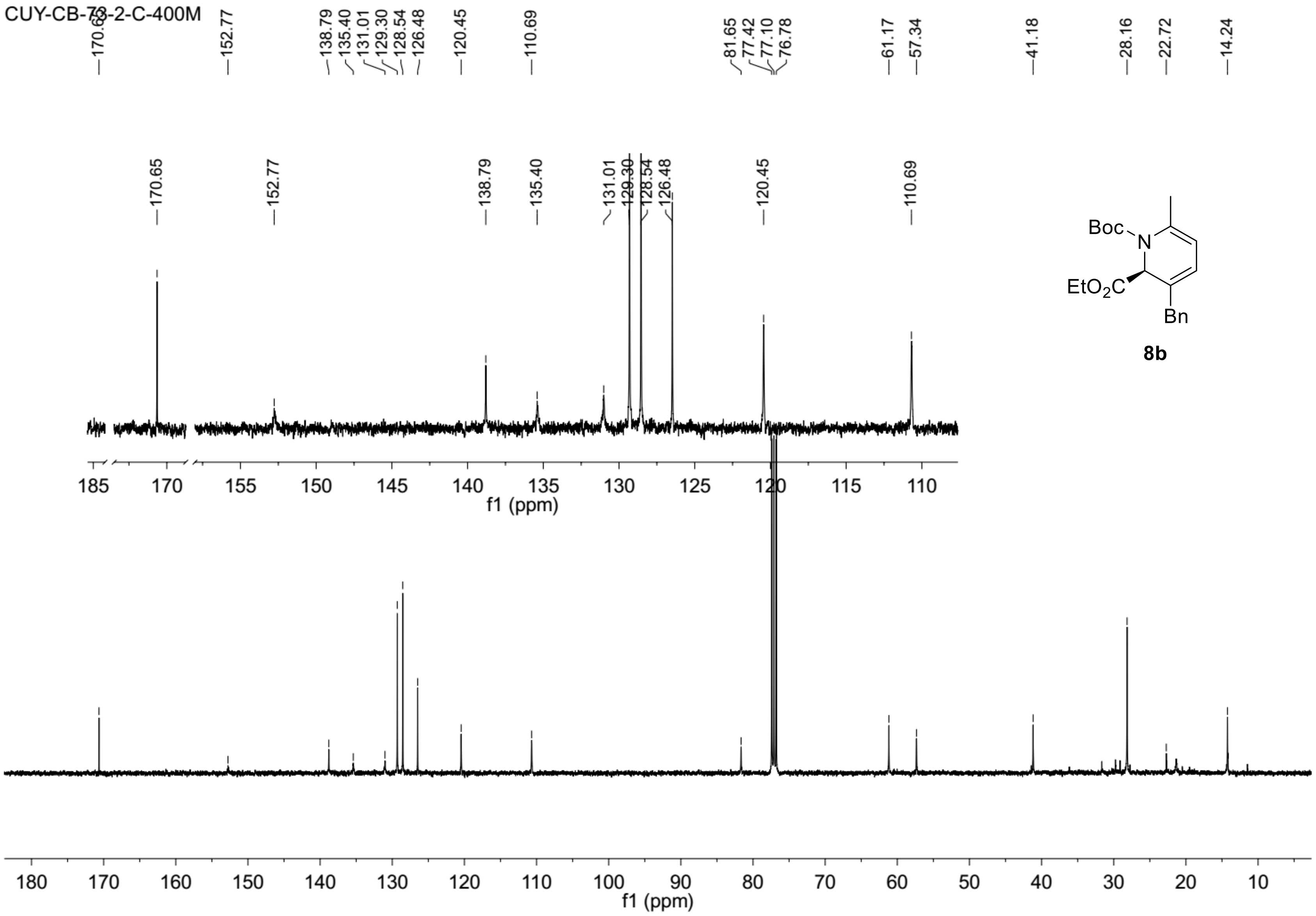

8b

Supplementary Figure 61. ^{13}C NMR (100 MHz, $CDCl_3$) spectra for compound **8b**

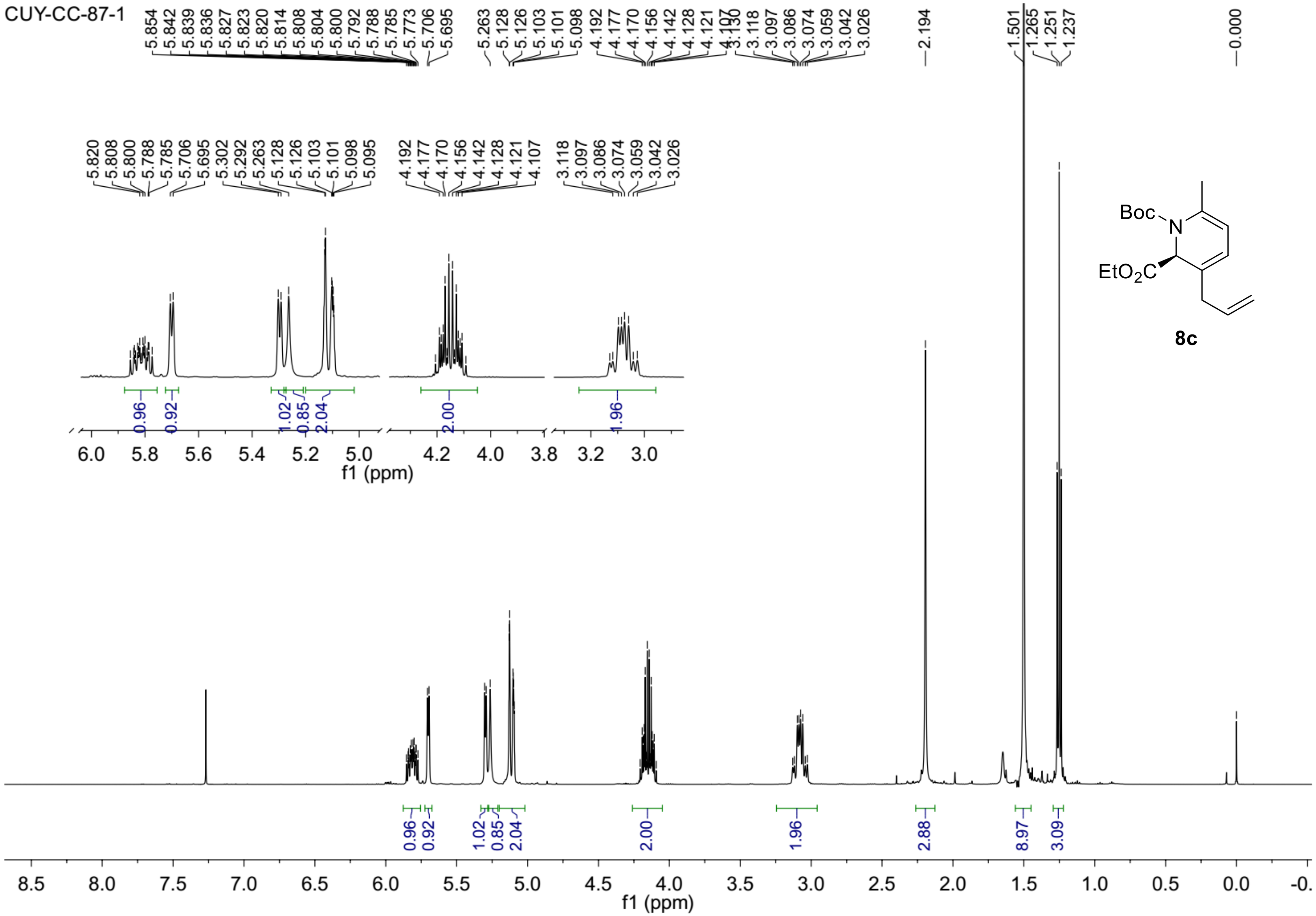

Supplementary Figure 62. ^{1}H NMR (500 MHz, $CDCl_3$) spectra for compound **8c**

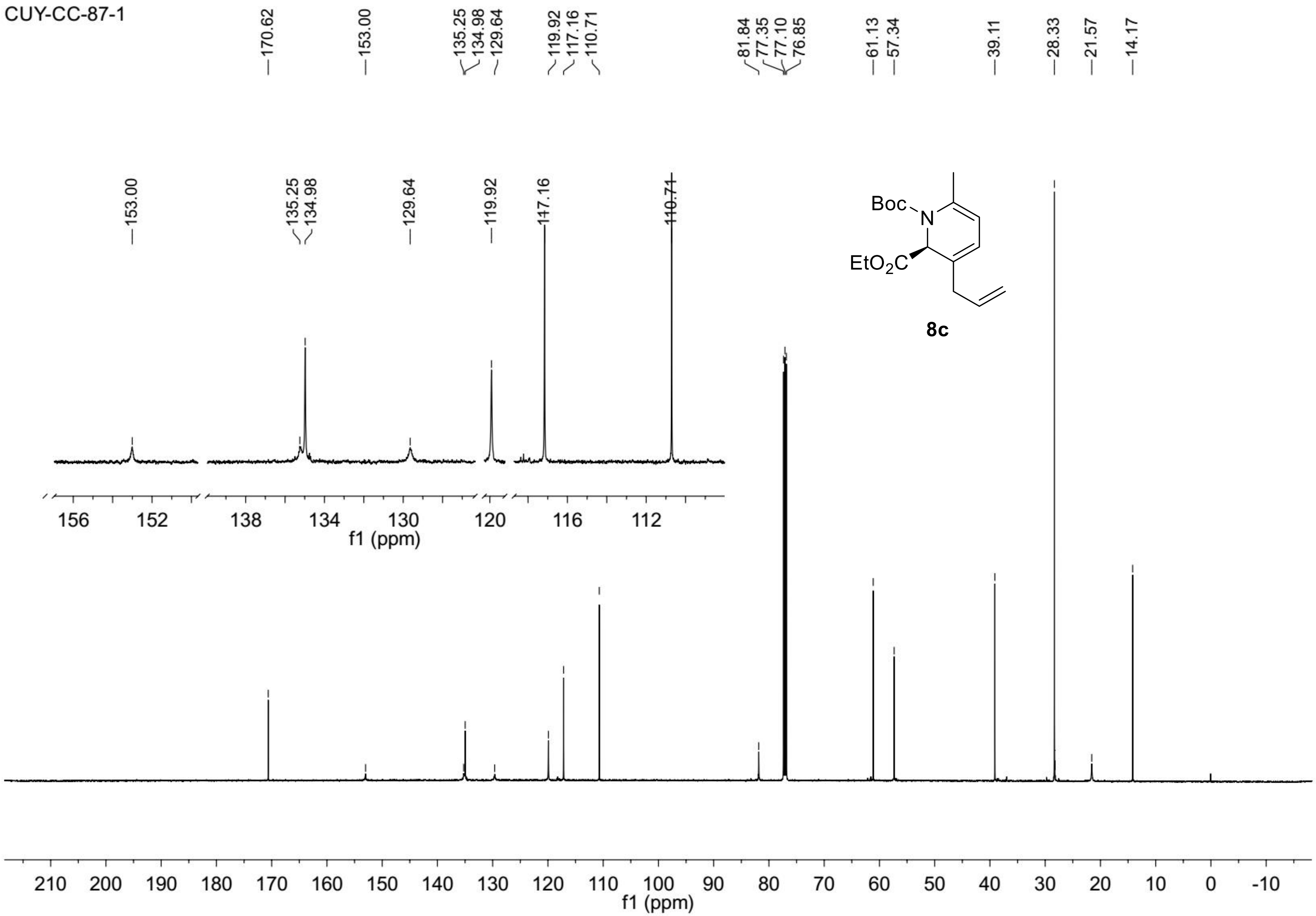

Supplementary Figure 63. ^{13}C NMR (125 MHz, $CDCl_3$) spectra for compound **8c**

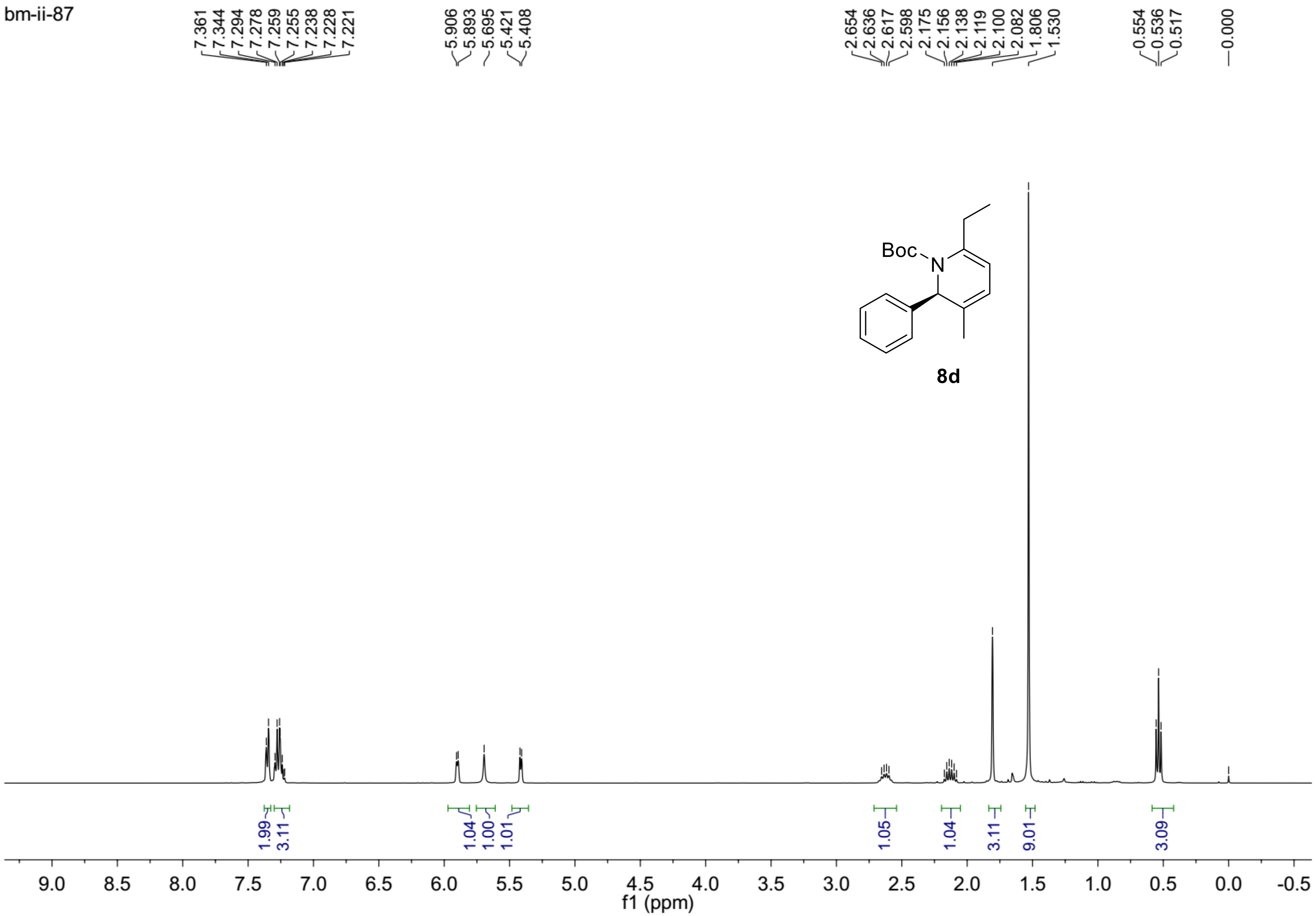

Supplementary Figure 64. ^{1}H NMR (400 MHz, $CDCl_3$) spectra for compound **8d**

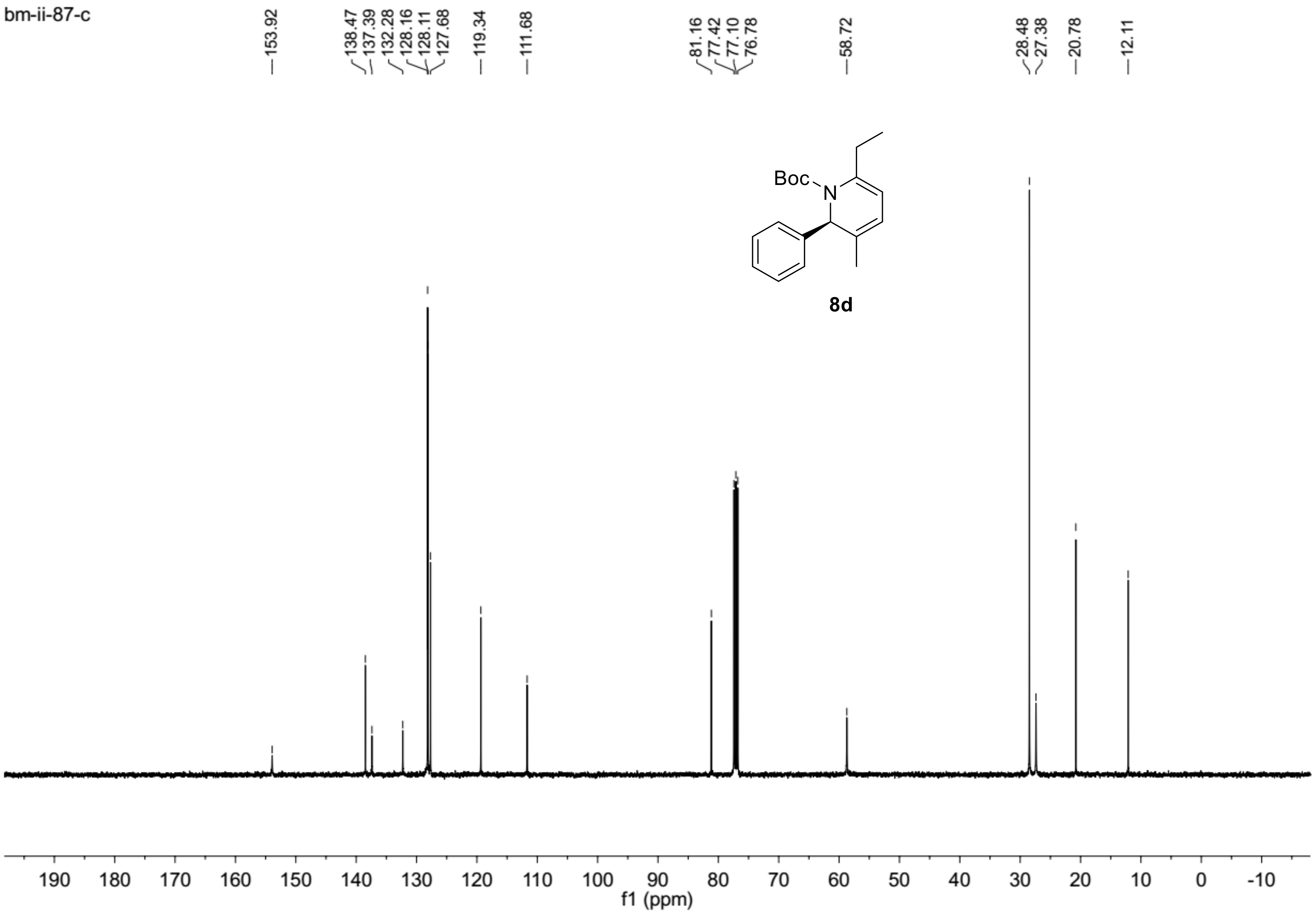

Supplementary Figure 65. ^{13}C NMR (100 MHz, $CDCl_3$) spectra for compound **8d**

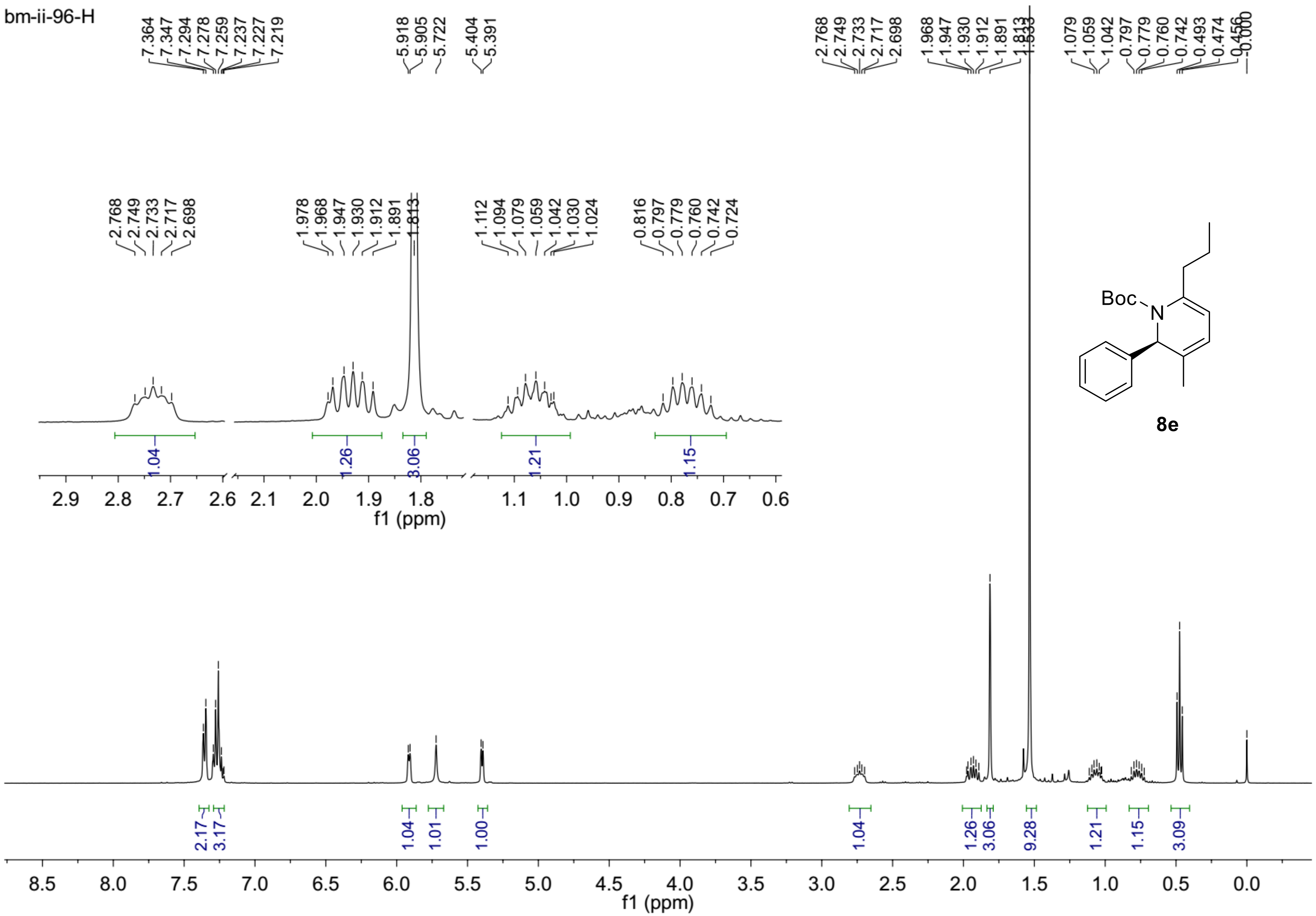

Supplementary Figure 66. ^{1}H NMR (400 MHz, $CDCl_3$) spectra for compound **8e**

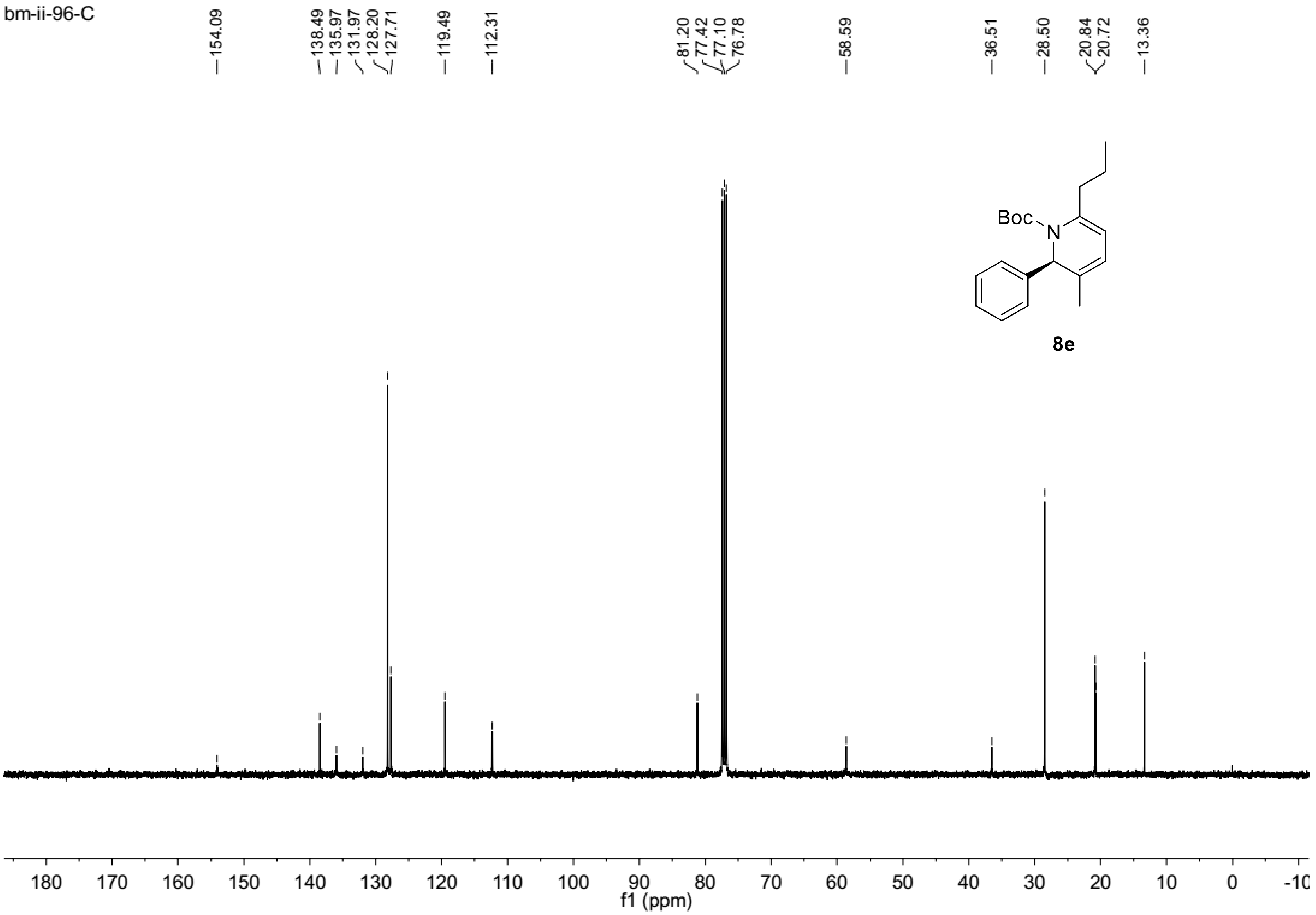

Supplementary Figure 67. ^{13}C NMR (100 MHz, $CDCl_3$) spectra for compound **8e**

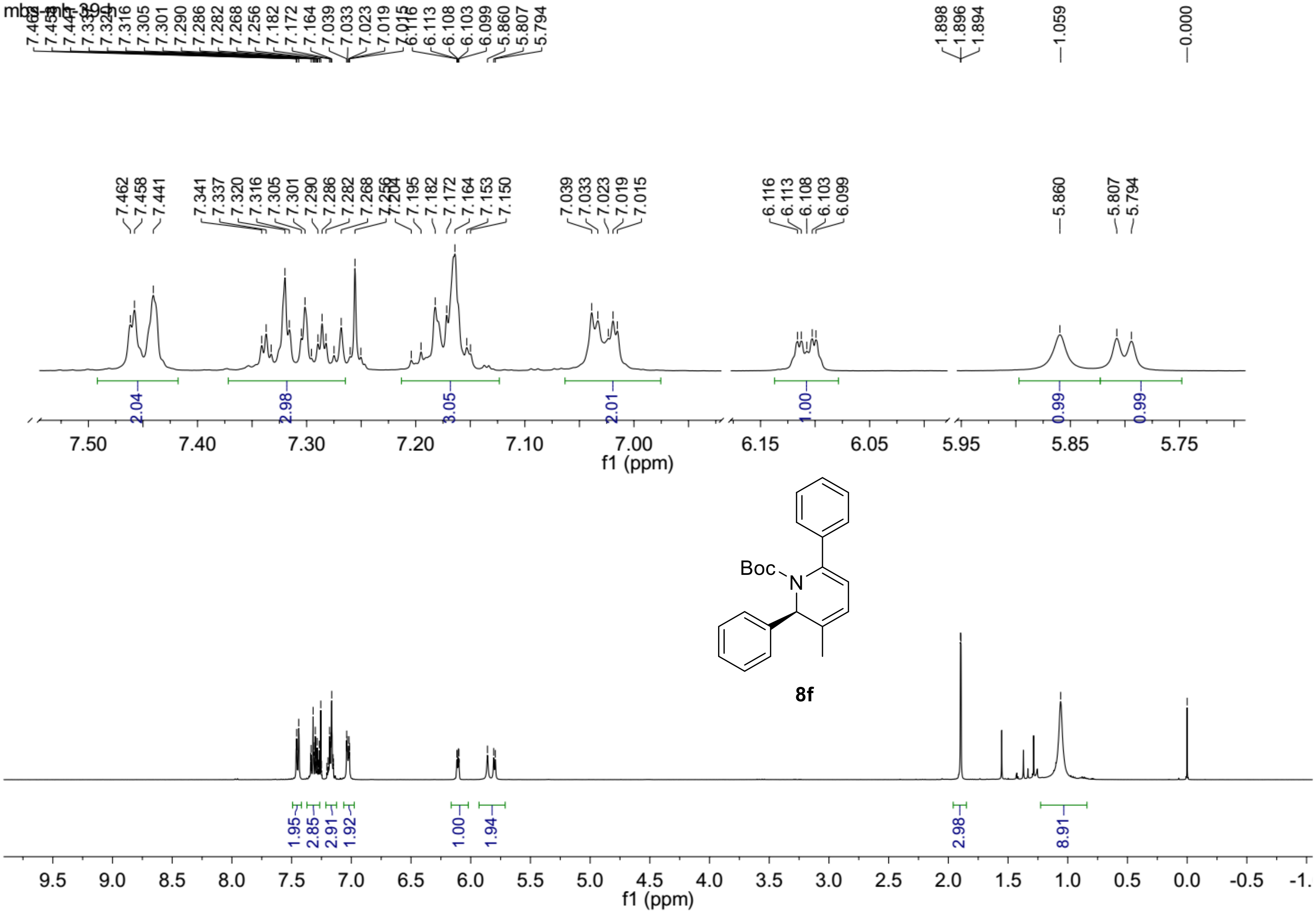

Supplementary Figure 68. ^{1}H NMR (400 MHz, $CDCl_3$) spectra for compound **8f**

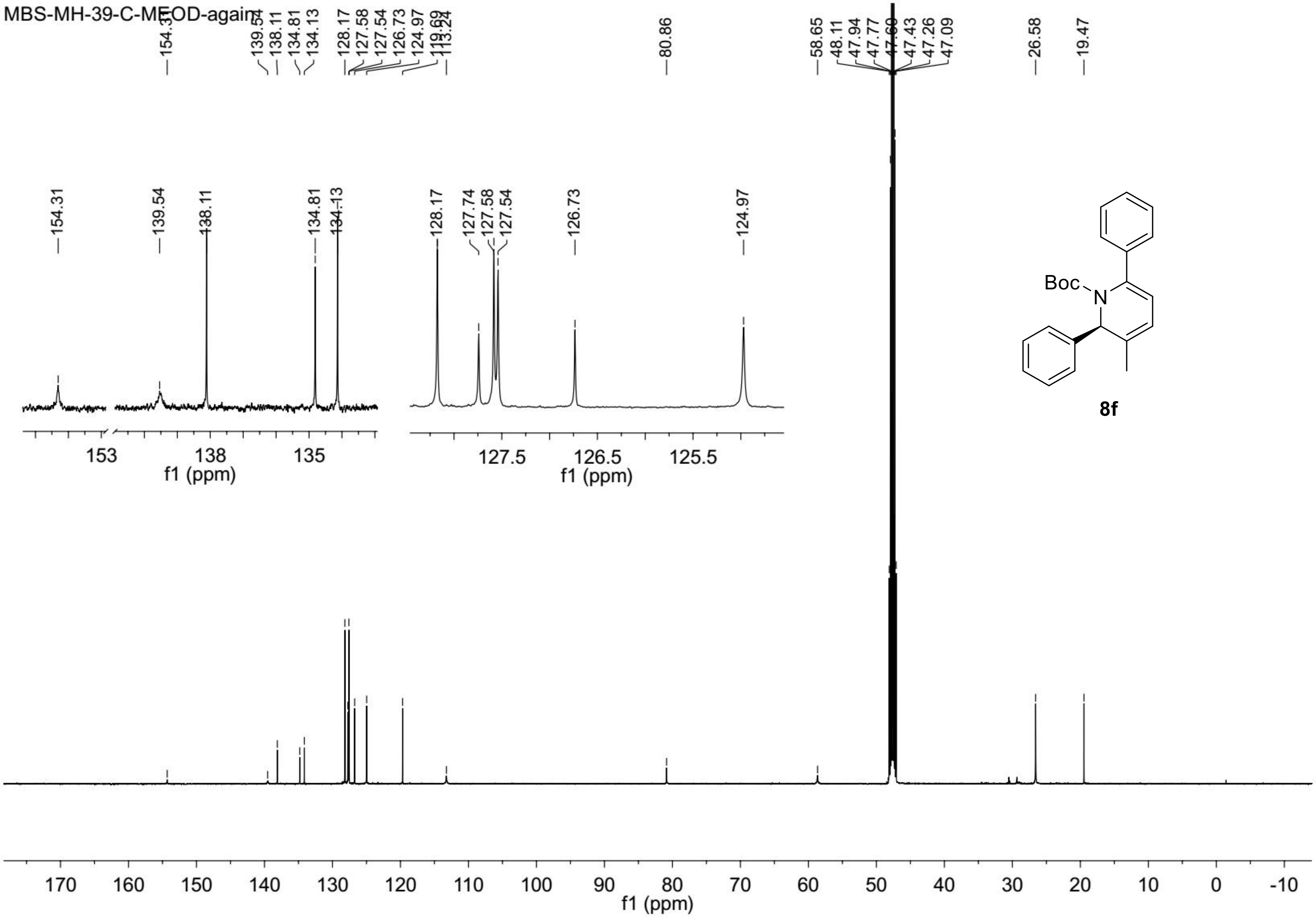

Supplementary Figure 69. ^{13}C NMR (100 MHz, $CDCl_3$) spectra for compound **8f**

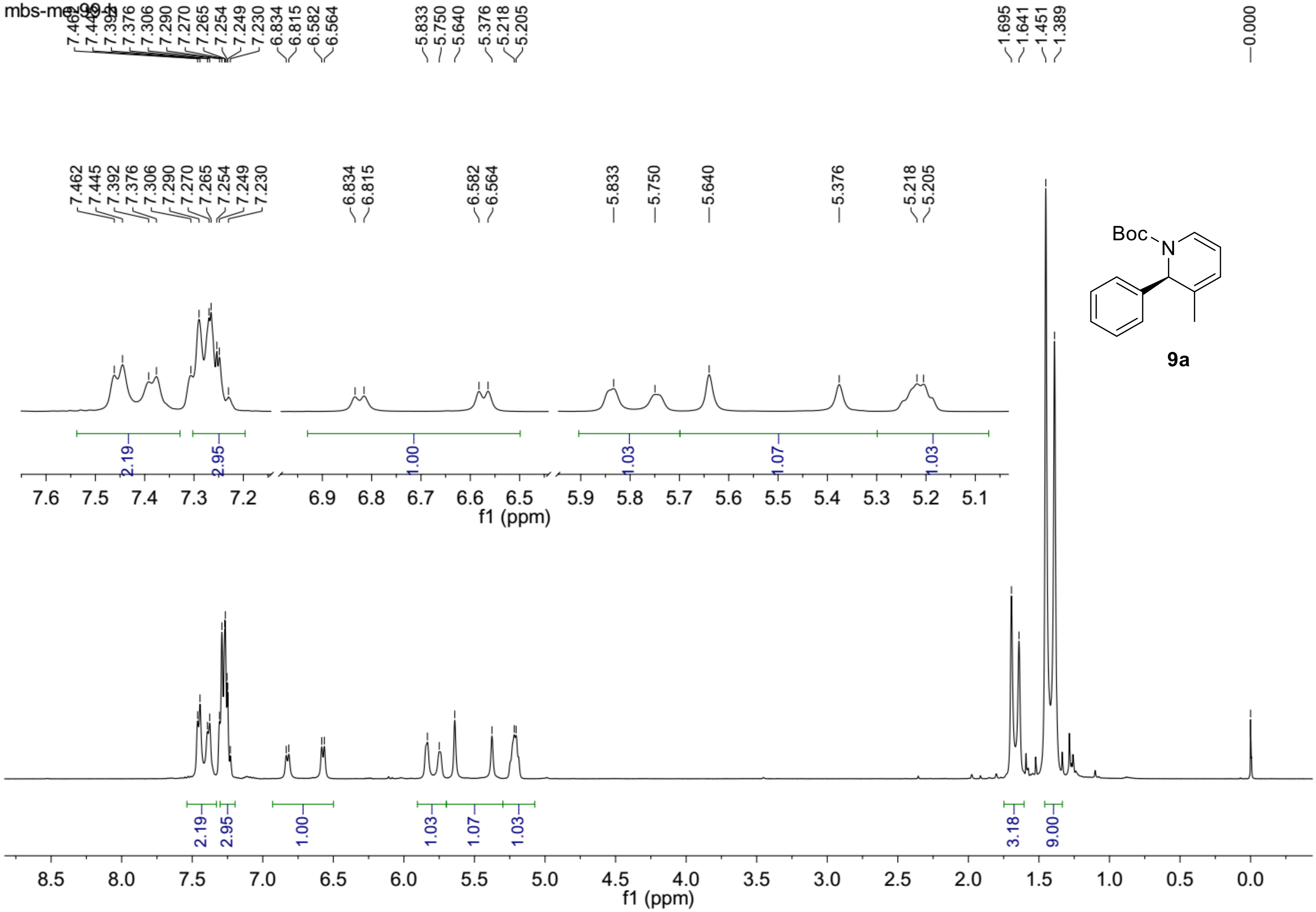

Supplementary Figure 70. ^{1}H NMR (400 MHz, $CDCl_3$) spectra for compound **9a**

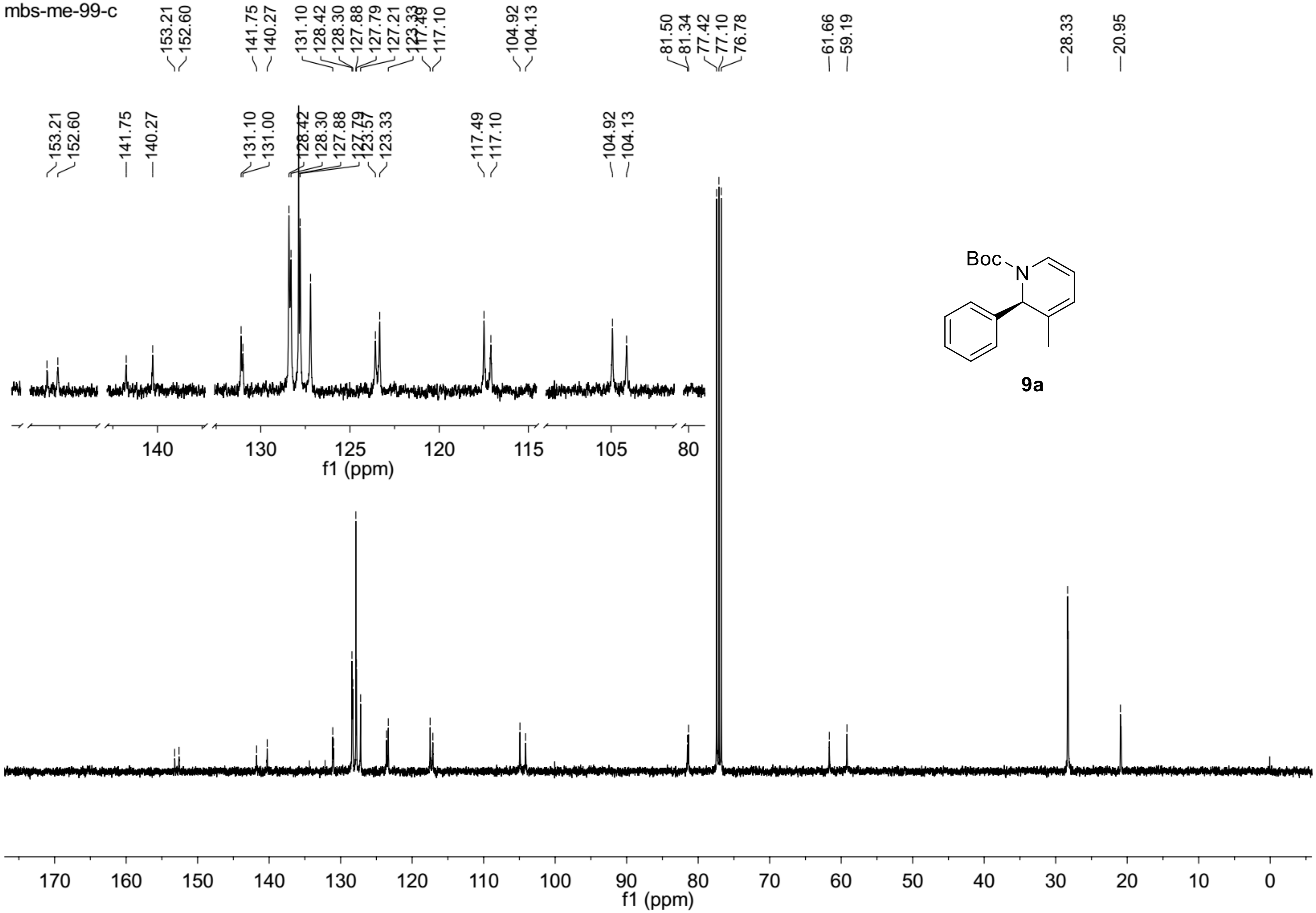

Supplementary Figure 71. ^{13}C NMR (100 MHz, $CDCl_3$) spectra for compound **9a**

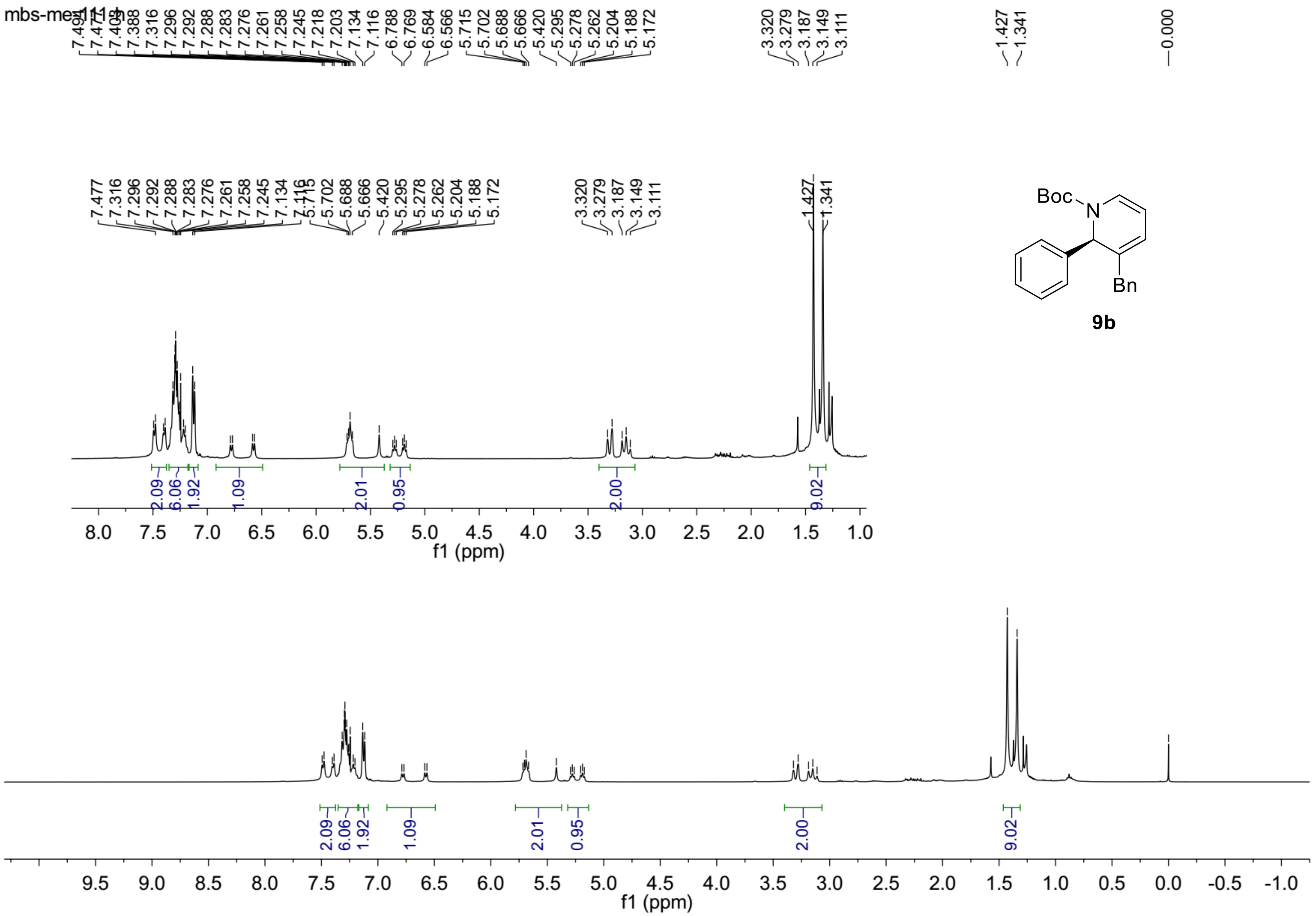

Supplementary Figure 72. ^{1}H NMR (400 MHz, $CDCl_3$) spectra for compound **9b**

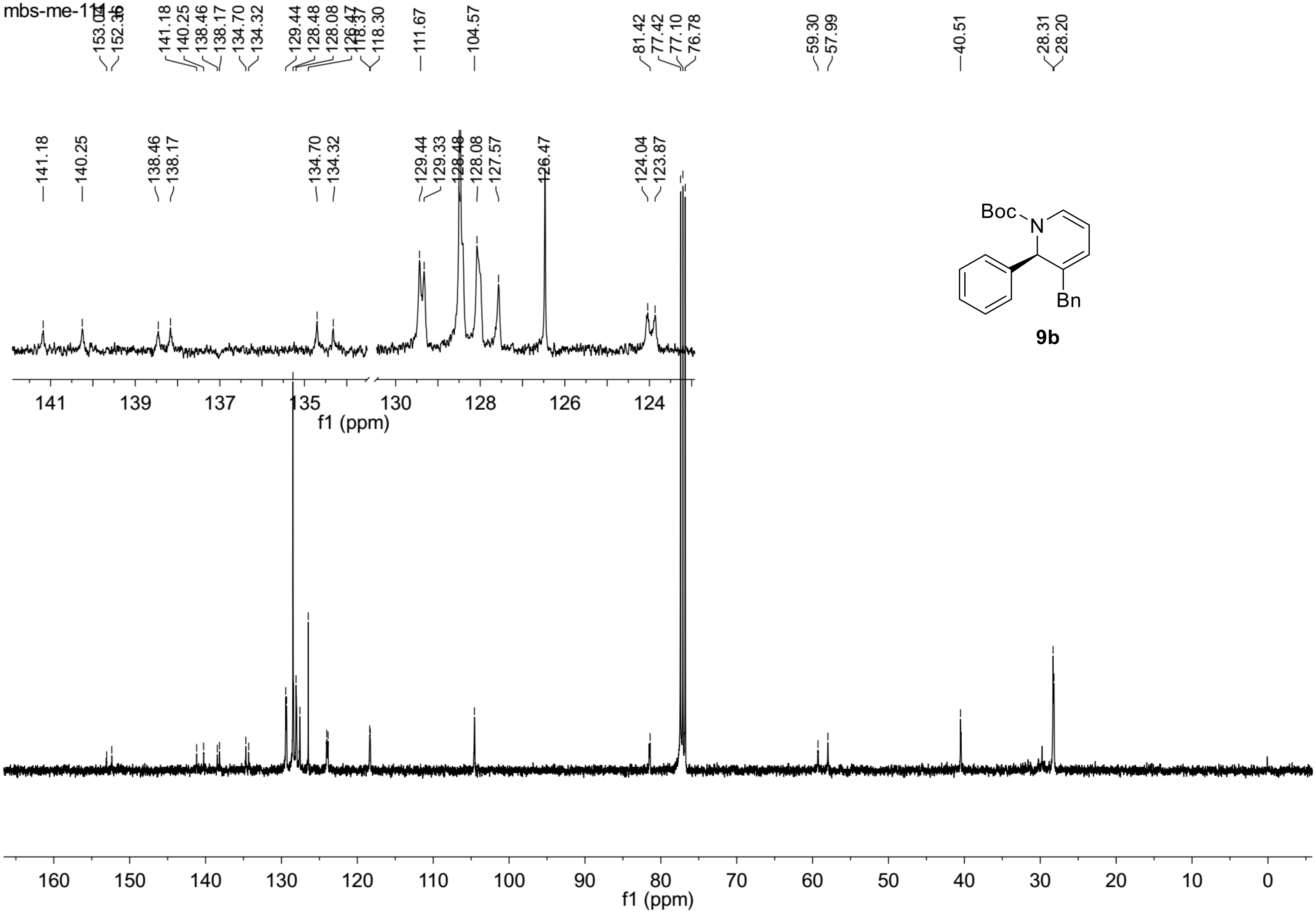

Supplementary Figure 73. ^{13}C NMR (100 MHz, $CDCl_3$) spectra for compound **9b**

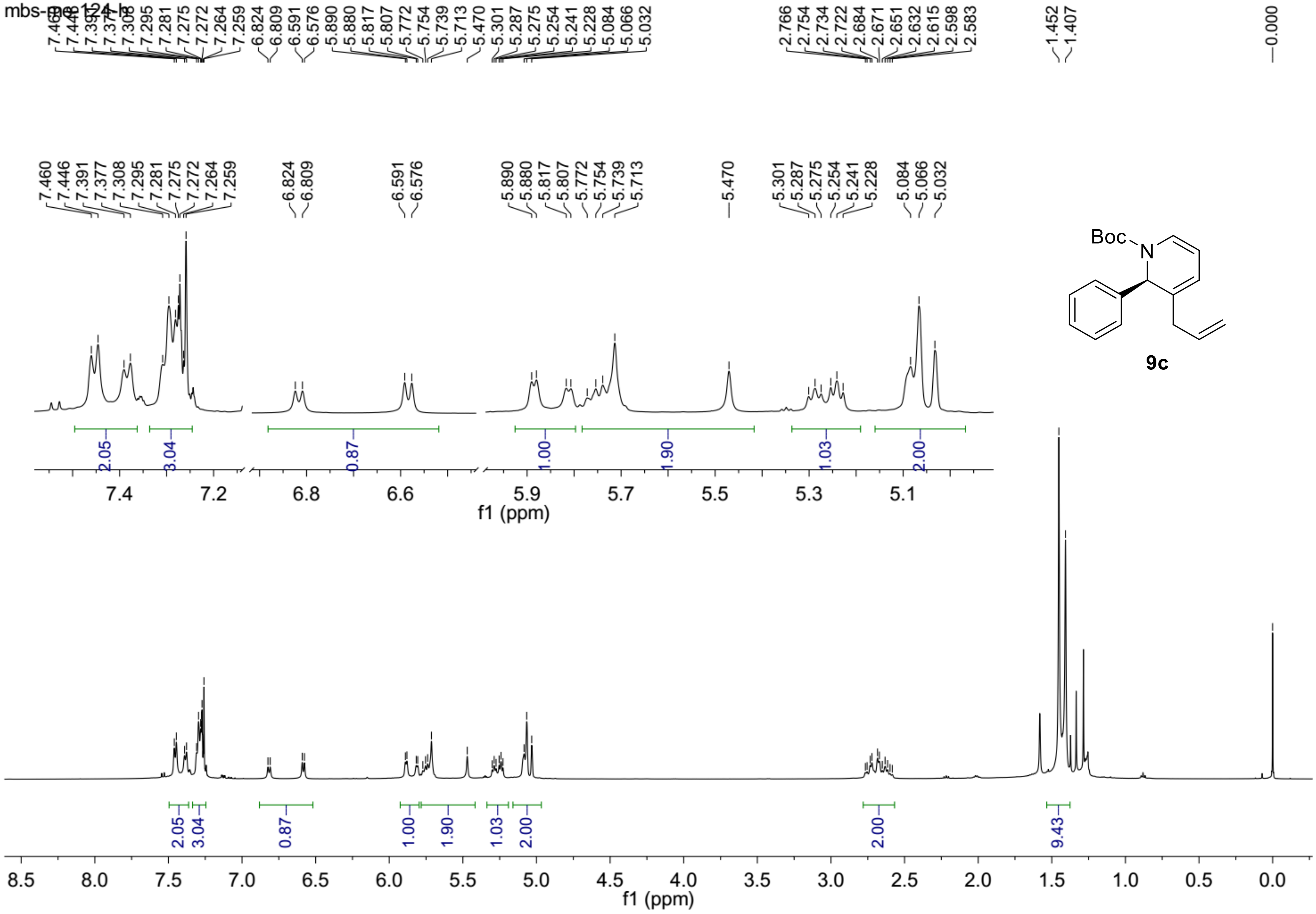

Supplementary Figure 74. ^{1}H NMR (500 MHz, $CDCl_3$) spectra for compound **9c**

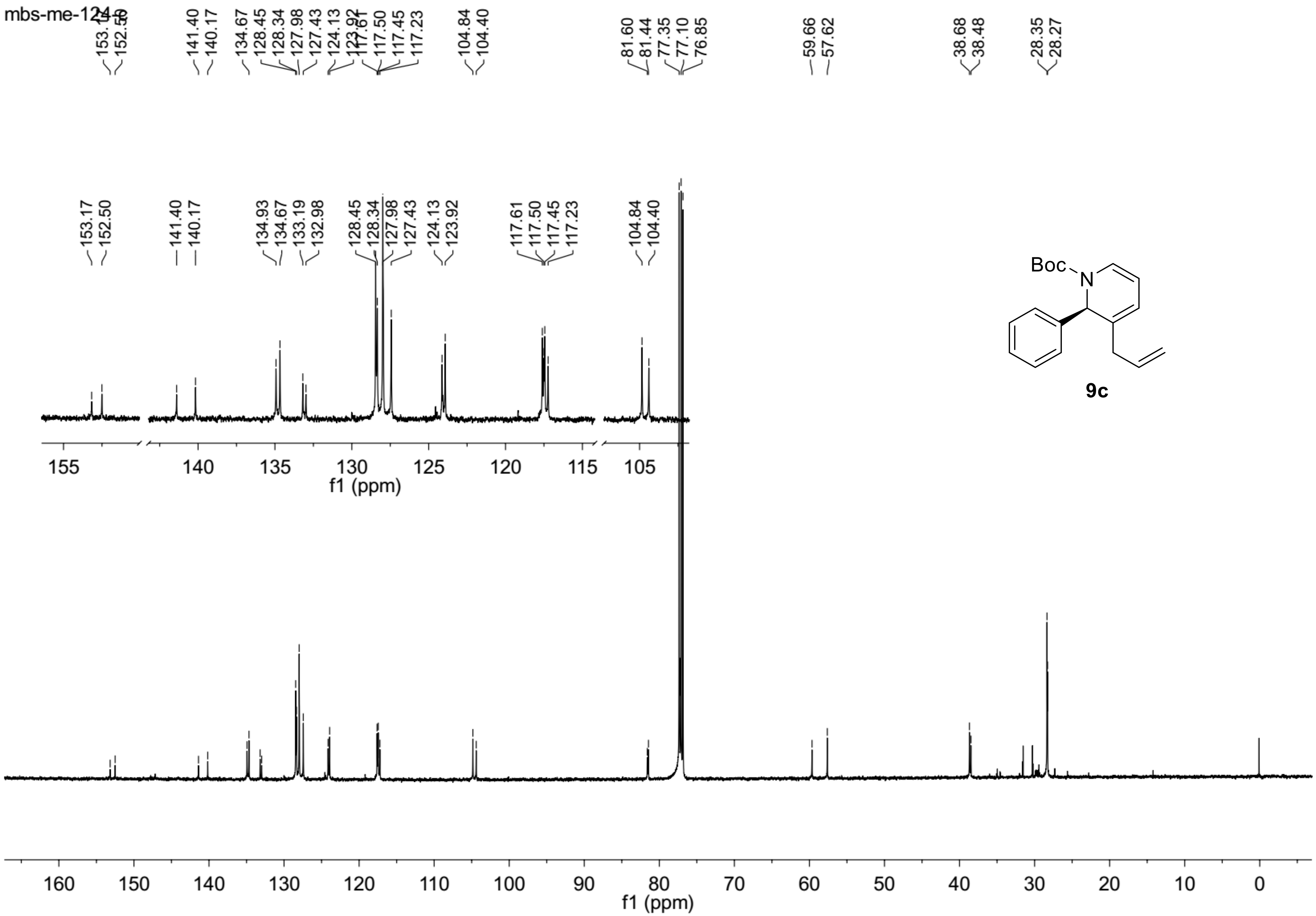

Supplementary Figure 75. ^{13}C NMR (125 MHz, $CDCl_3$) spectra for compound **9c**

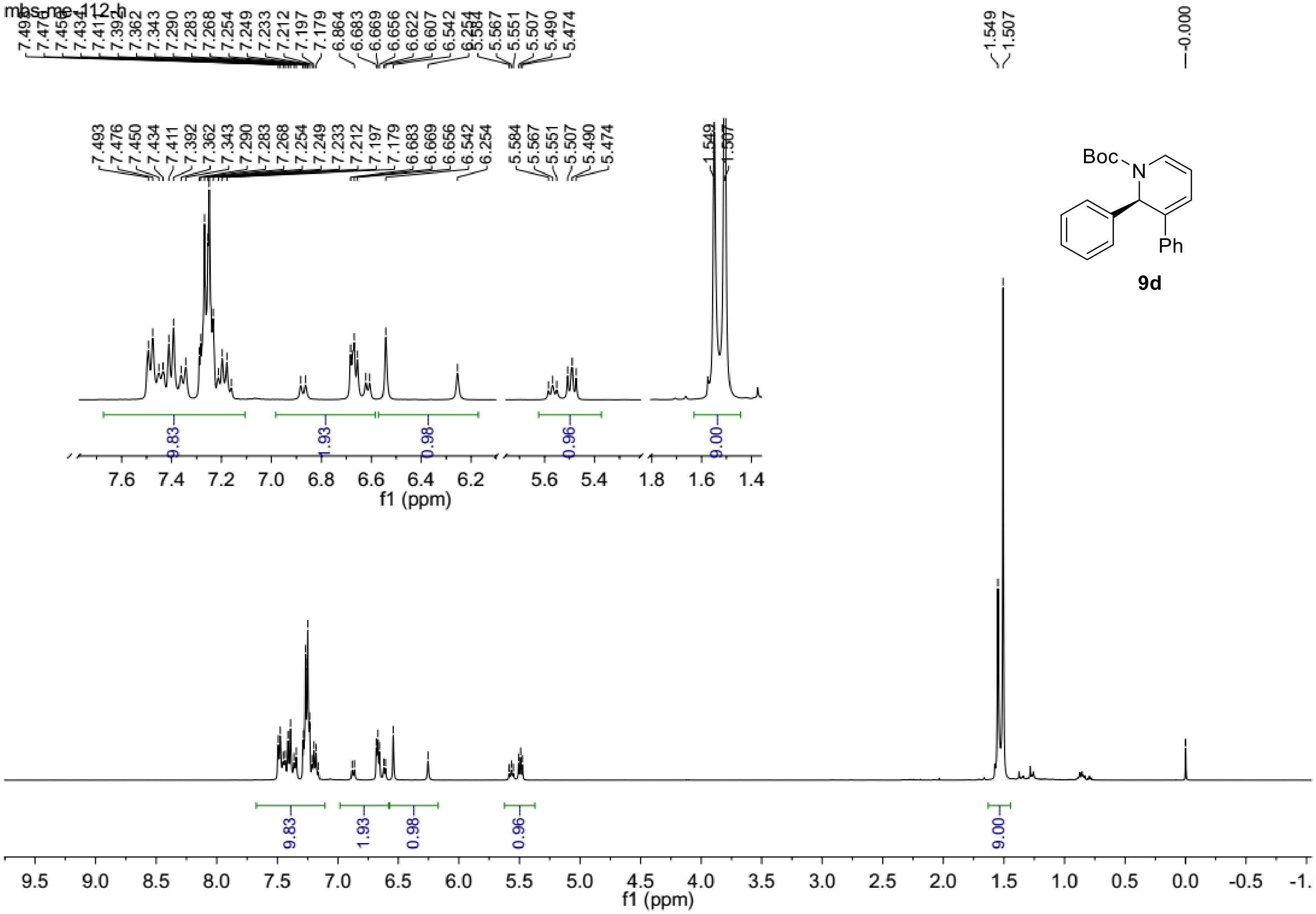

Supplementary Figure 76. ^{1}H NMR (400 MHz, $CDCl_3$) spectra for compound **9d**

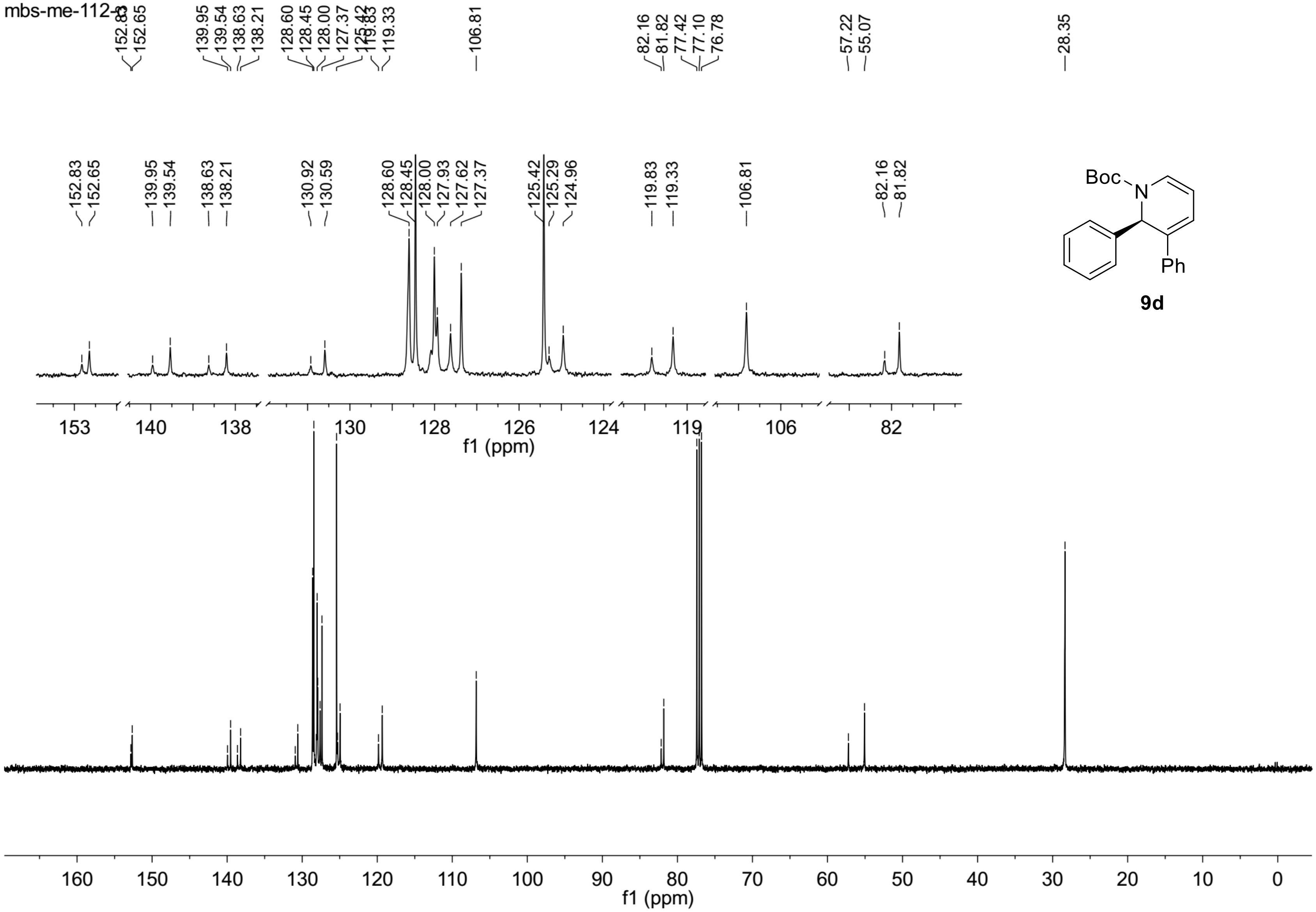

Supplementary Figure 77. ^{13}C NMR (100 MHz, $CDCl_3$) spectra for compound **9d**

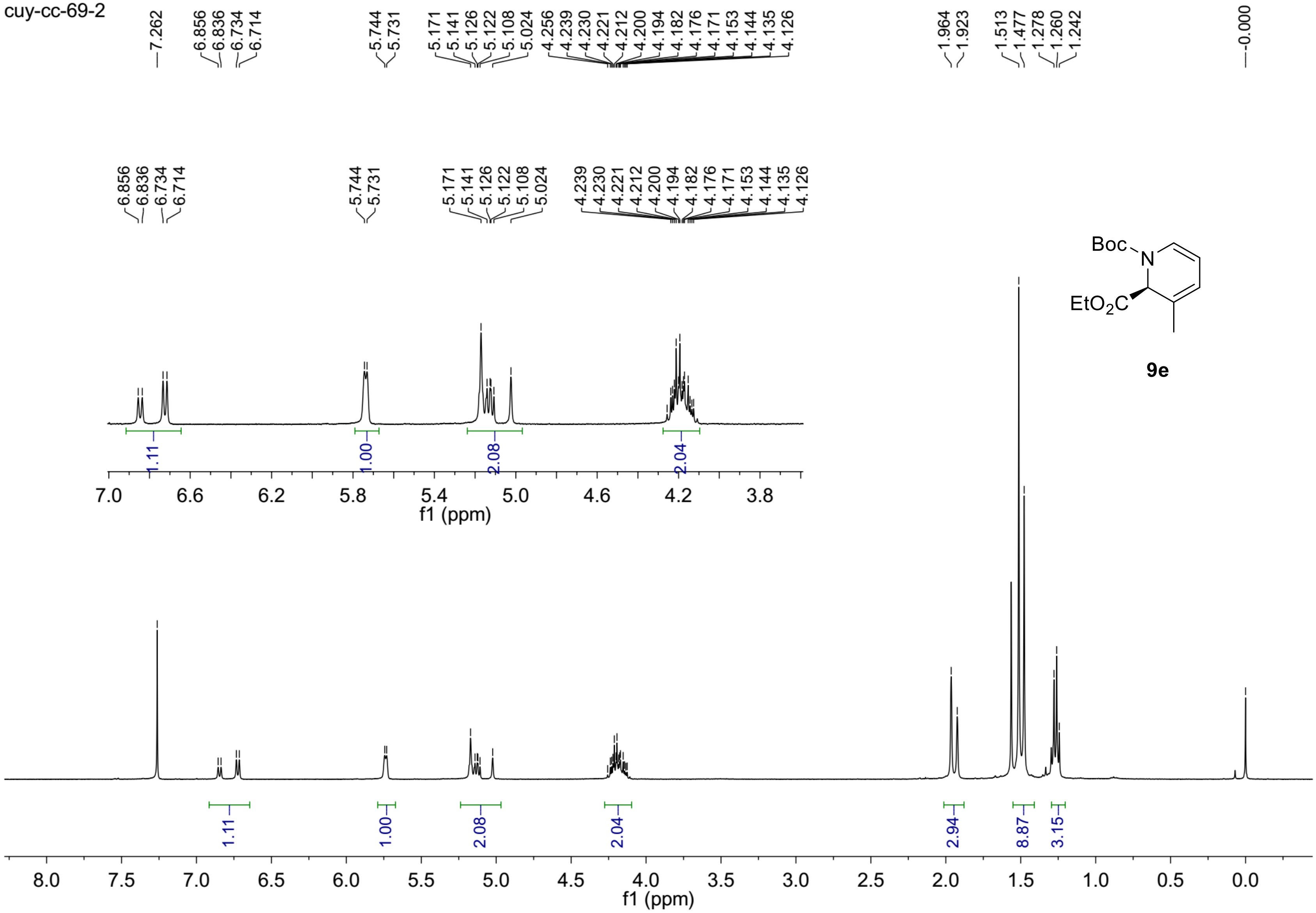

Supplementary Figure 78. ^{1}H NMR (400 MHz, $CDCl_3$) spectra for compound **9e**

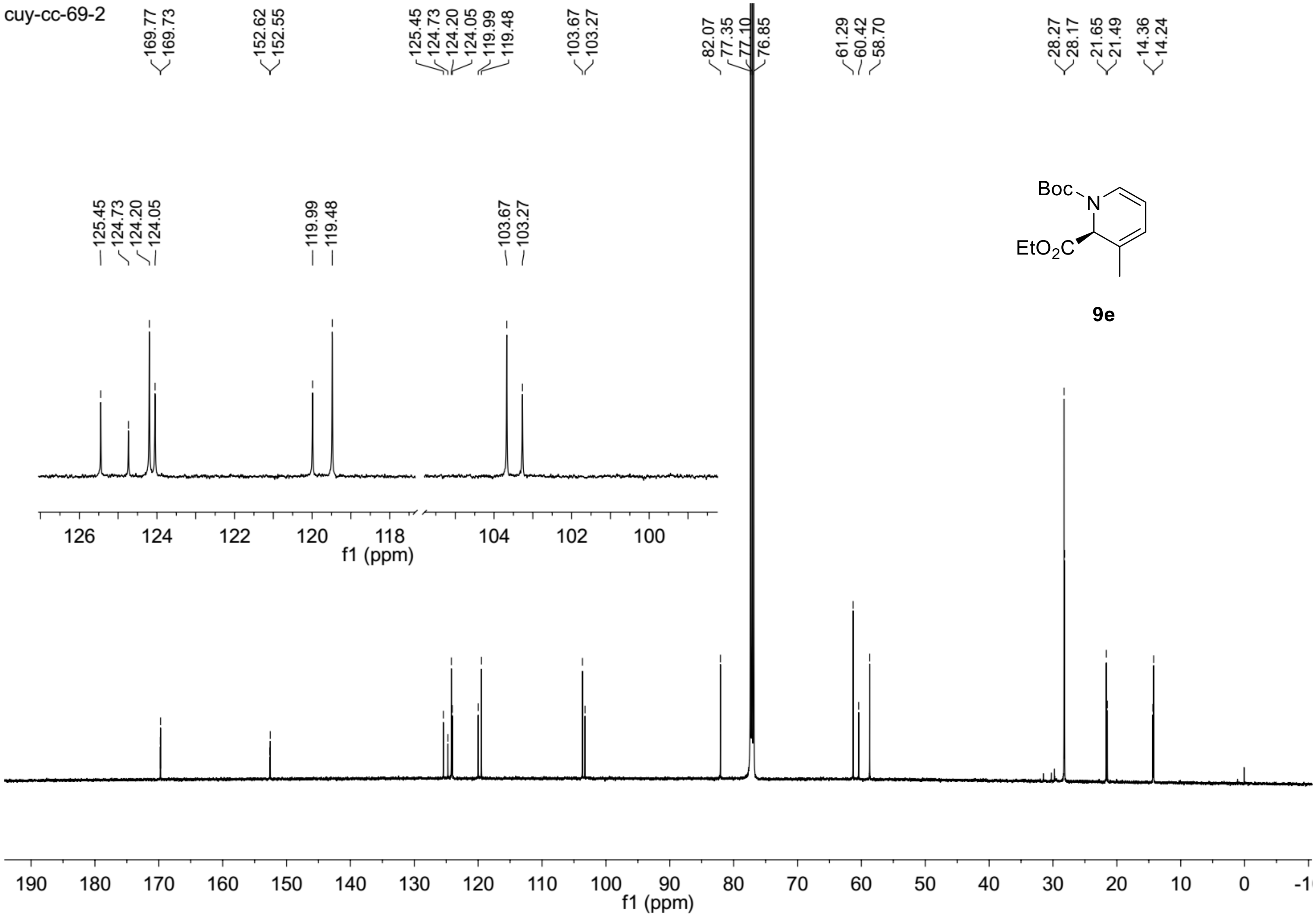

Supplementary Figure 79. ^{13}C NMR (125 MHz, $CDCl_3$) spectra for compound **9e**

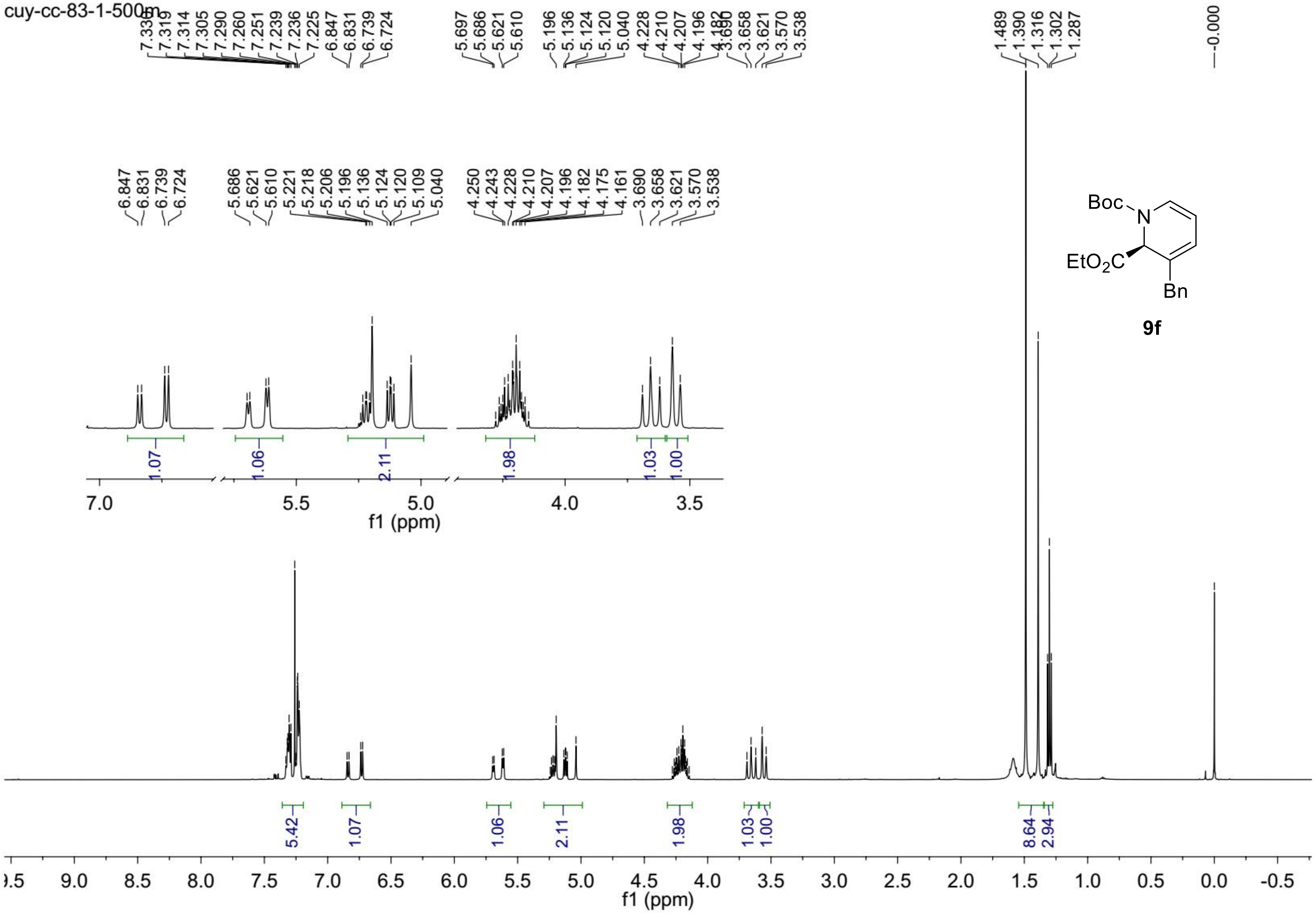

Supplementary Figure 80. ^{1}H NMR (500 MHz, $CDCl_3$) spectra for compound **9f**

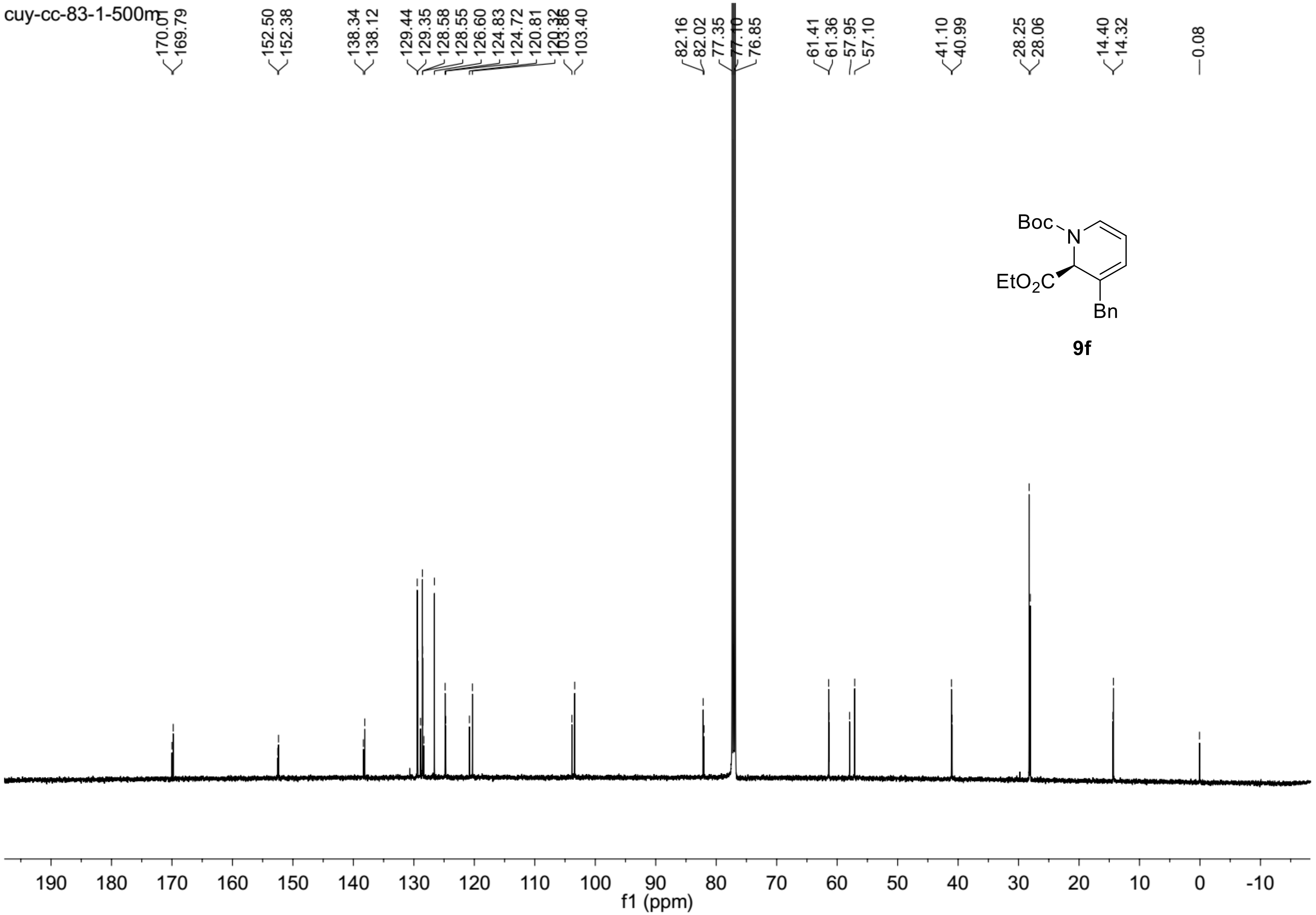

Supplementary Figure 81. ^{13}C NMR (125 MHz, $CDCl_3$) spectra for compound **9f**

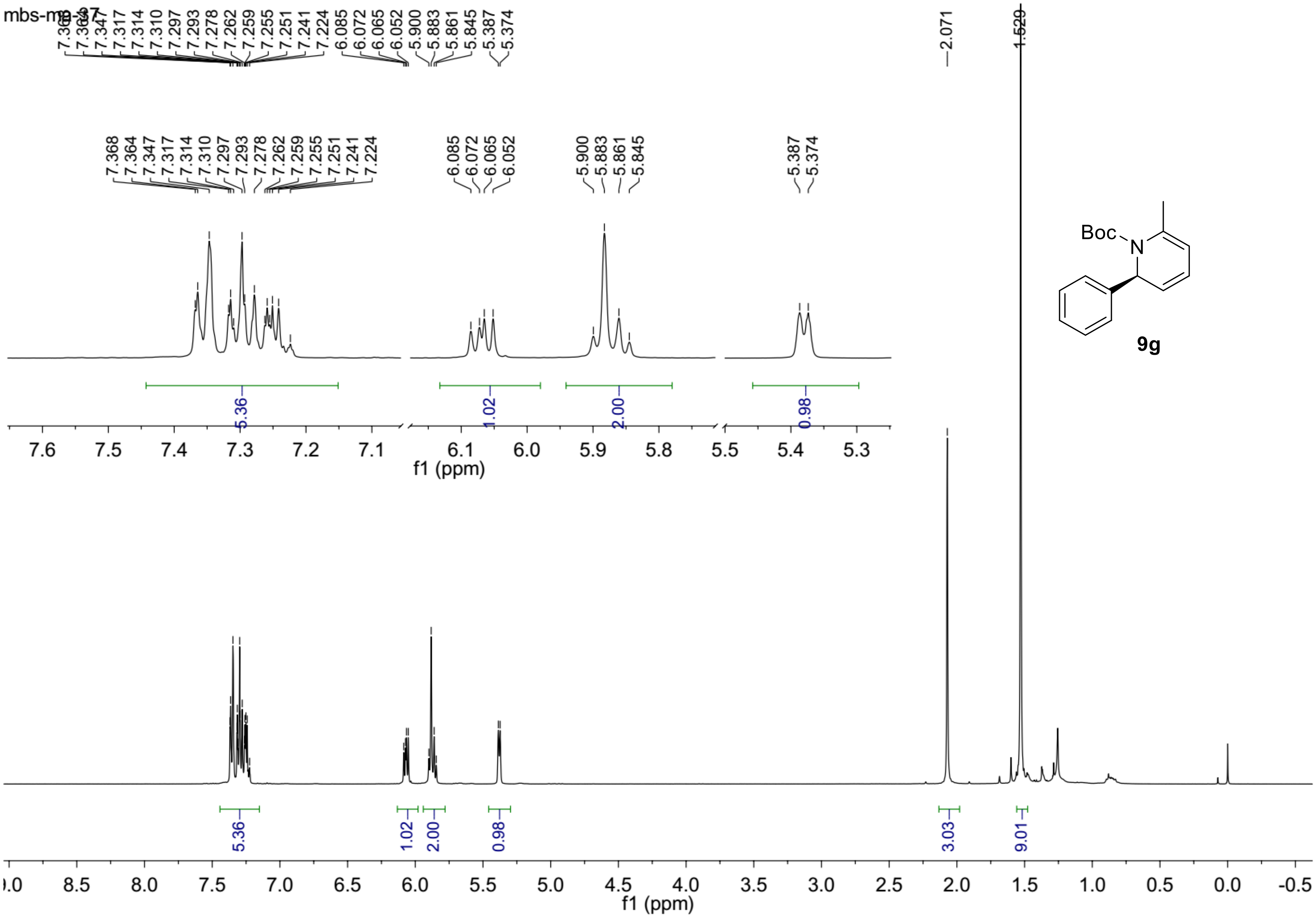

Supplementary Figure 82. ^{1}H NMR (400 MHz, $CDCl_3$) spectra for compound **9g**

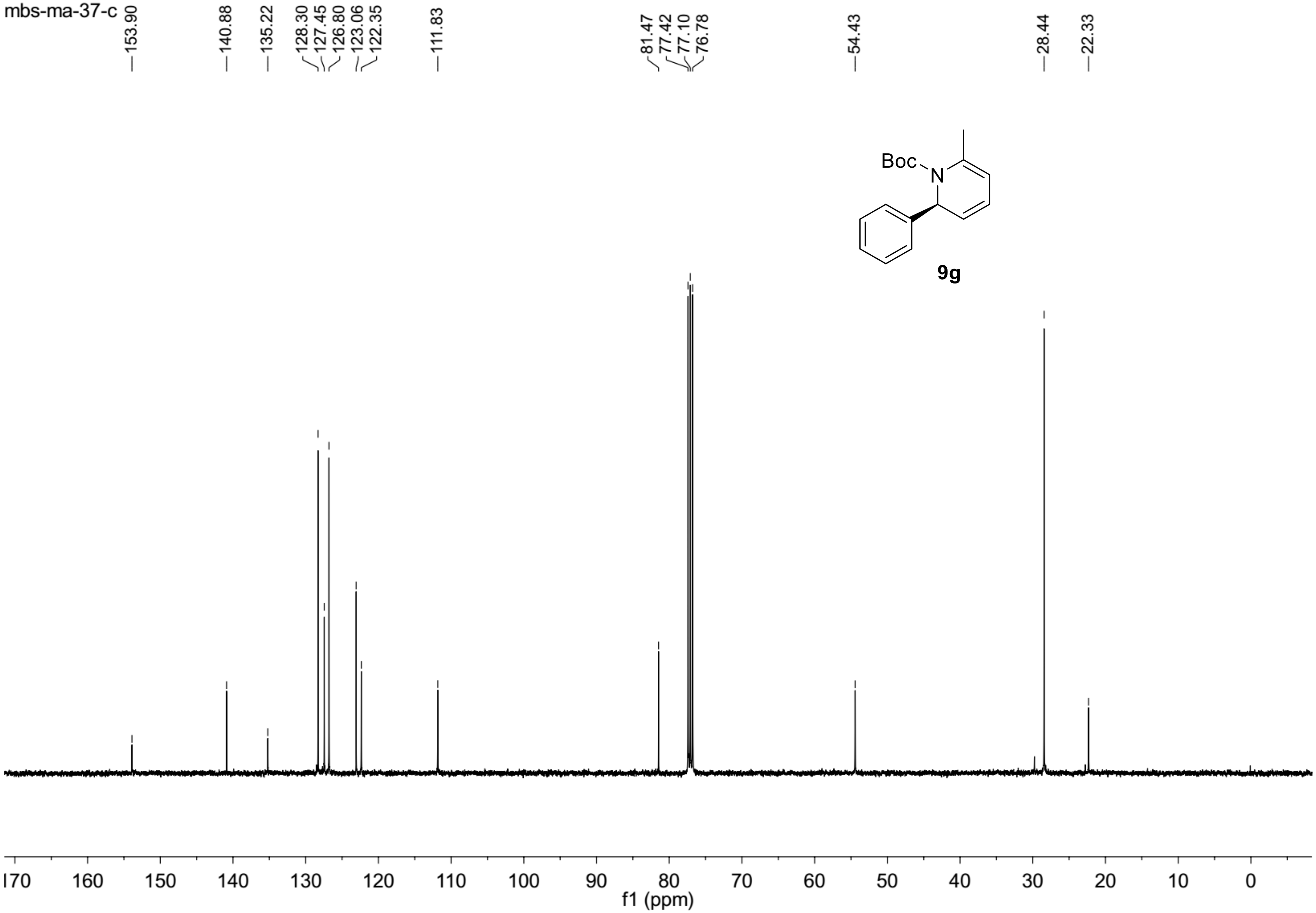

Supplementary Figure 83. ^{13}C NMR (100 MHz, $CDCl_3$) spectra for compound **9g**

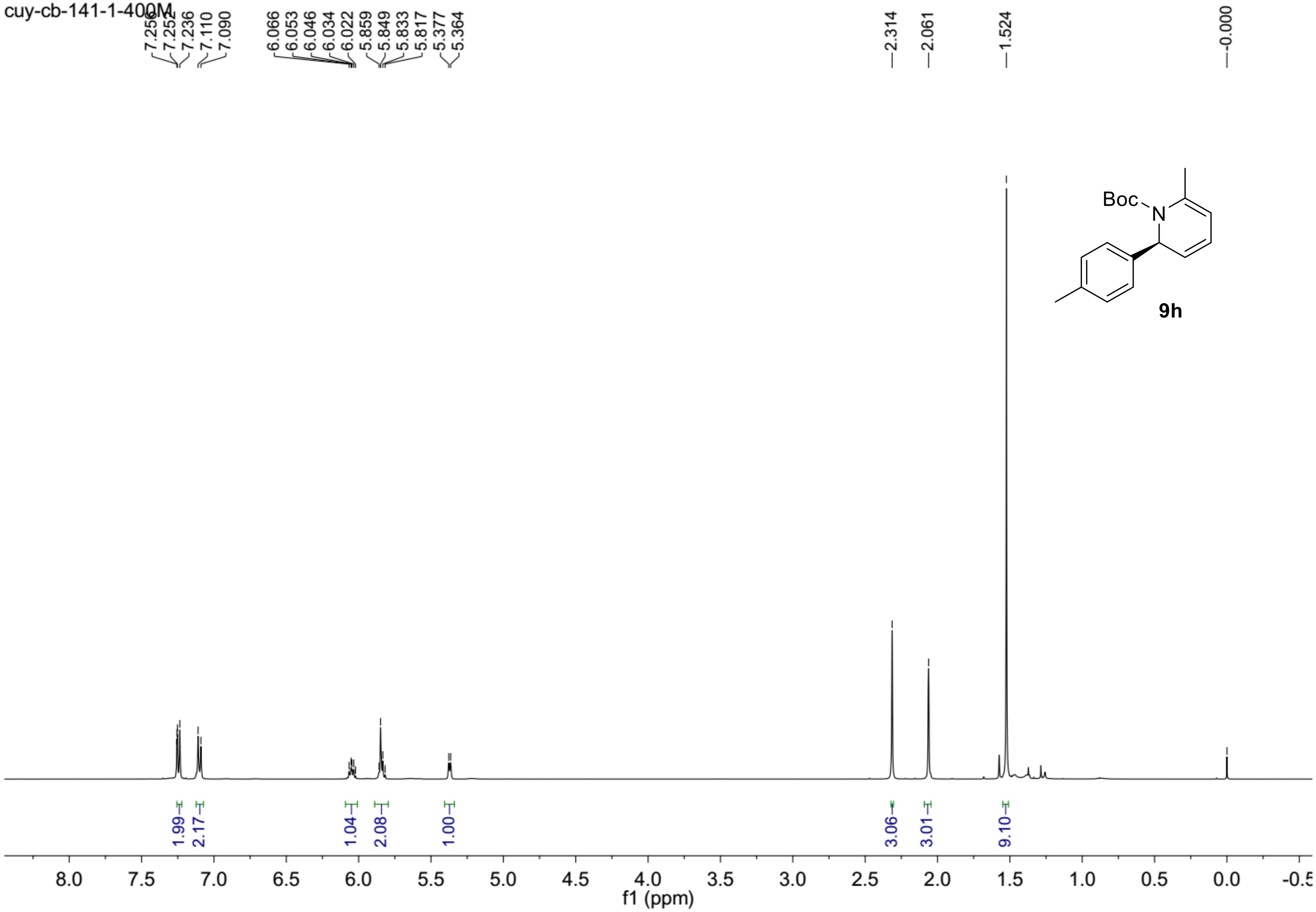

Supplementary Figure 84. ^{1}H NMR (400 MHz, $CDCl_3$) spectra for compound **9h**

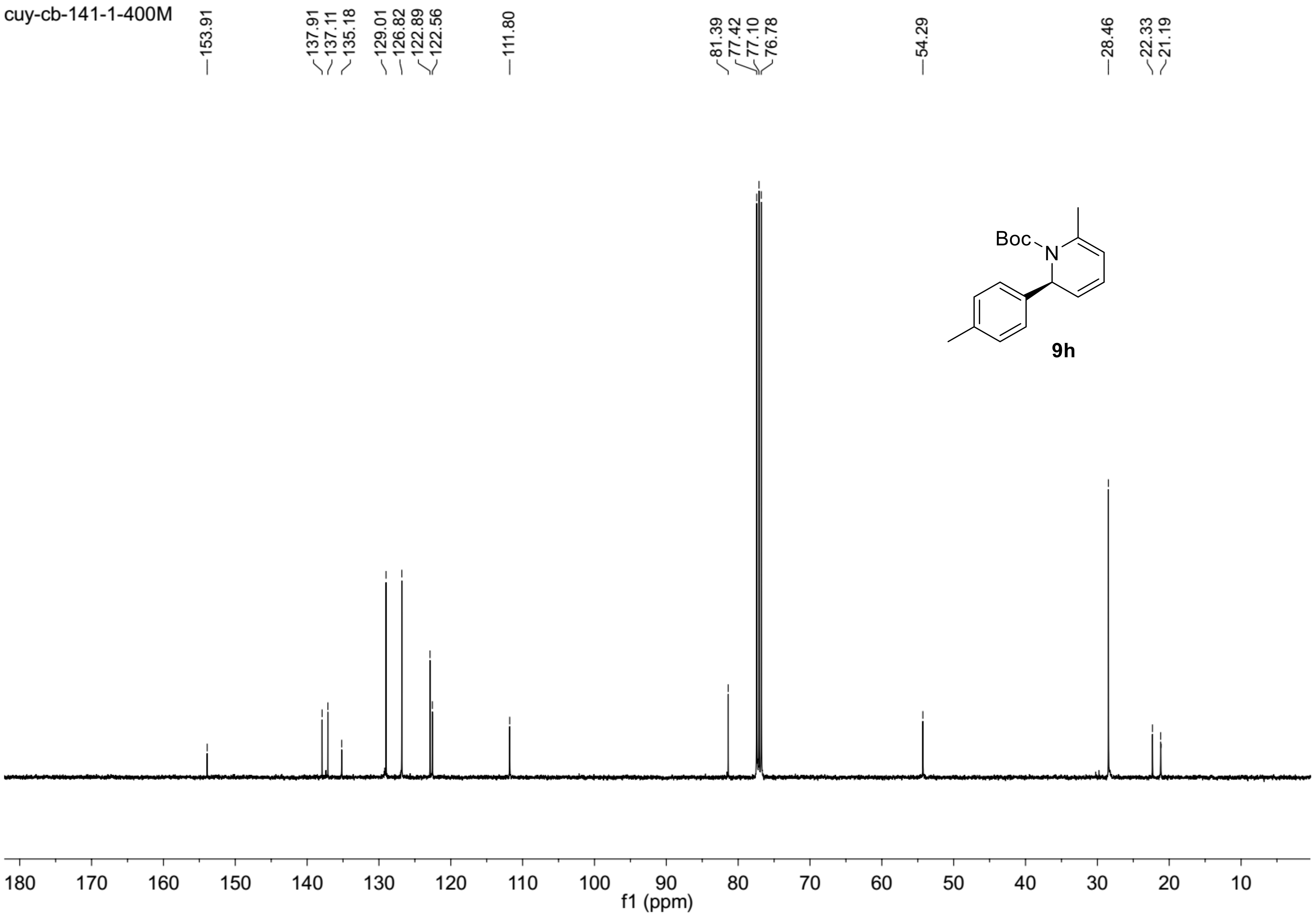

Supplementary Figure 85. ^{13}C NMR (100 MHz, $CDCl_3$) spectra for compound **9h**

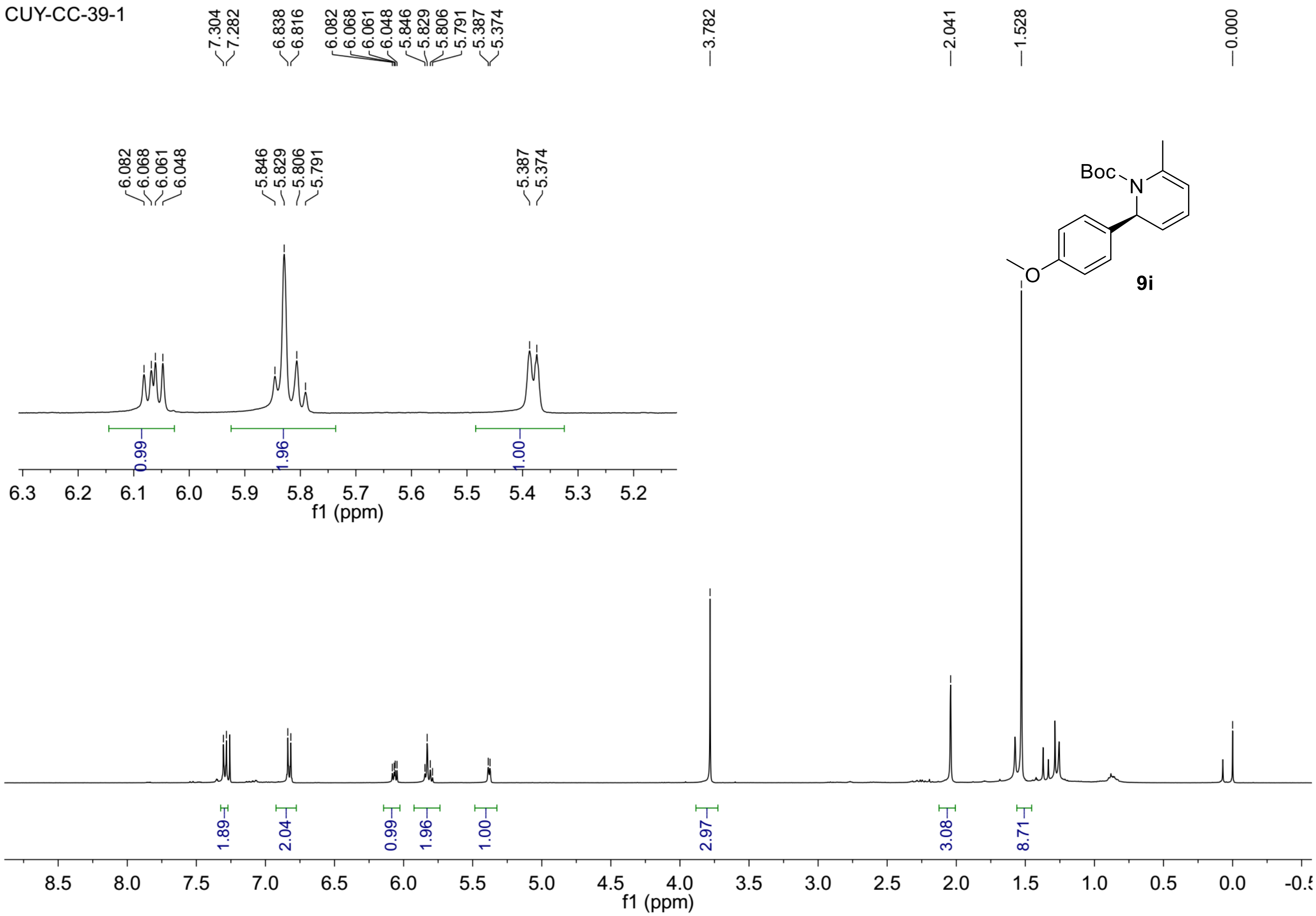

Supplementary Figure 86. ^{1}H NMR (400 MHz, $CDCl_3$) spectra for compound **9i**

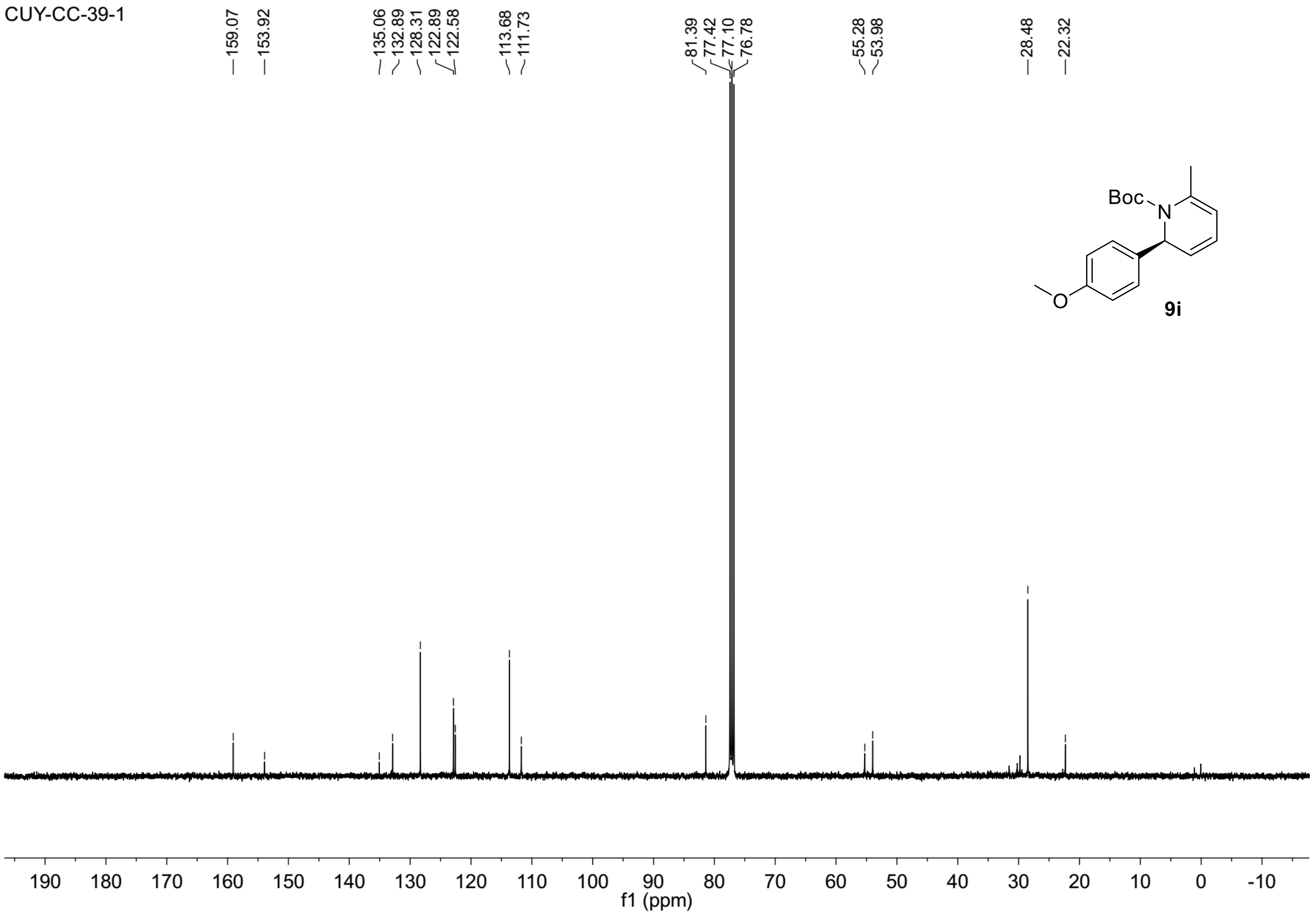

Supplementary Figure 87. ^{13}C NMR (100 MHz, $CDCl_3$) spectra for compound **9i**

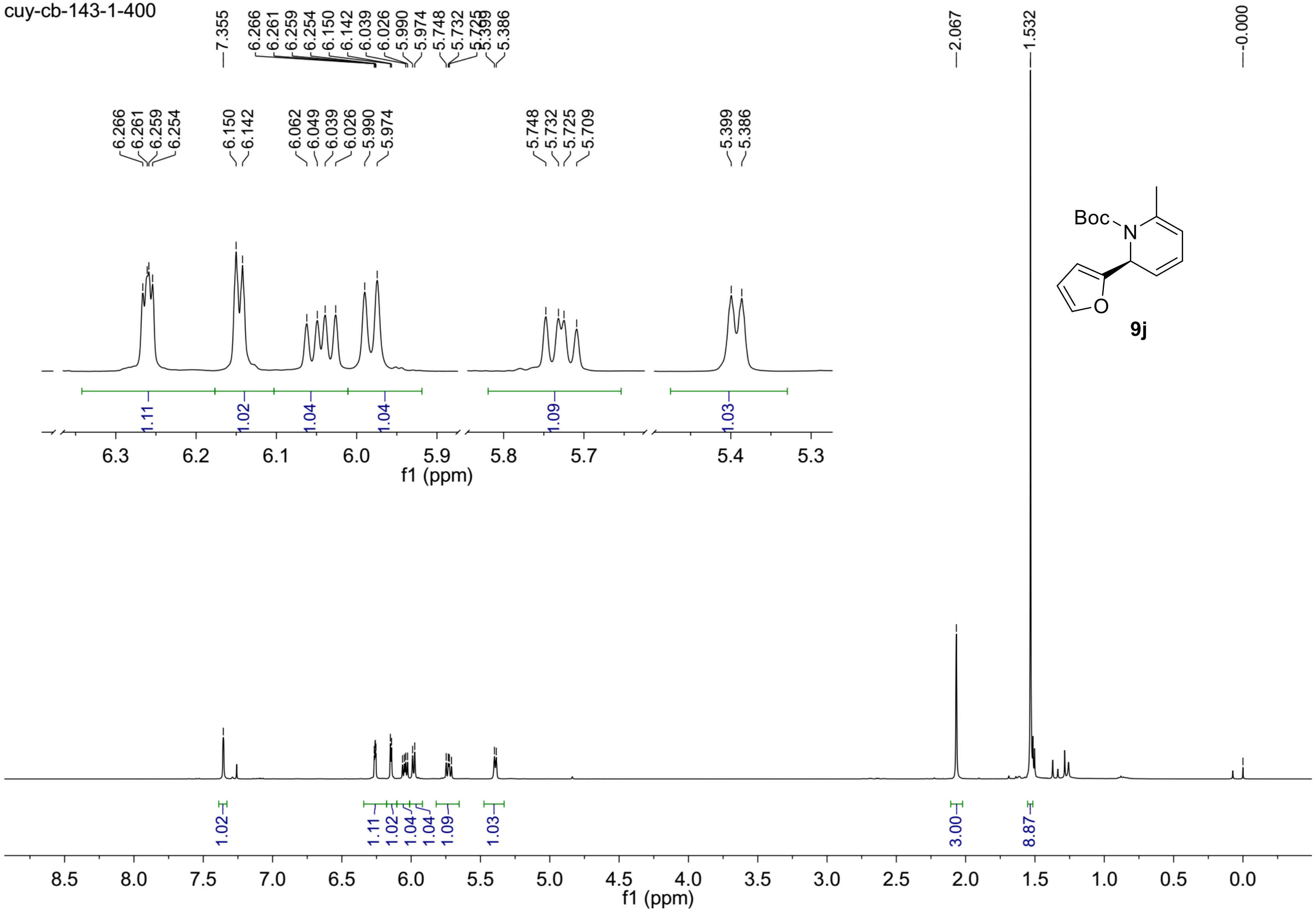

Supplementary Figure 88. ^{1}H NMR (400 MHz, $CDCl_3$) spectra for compound **9j**

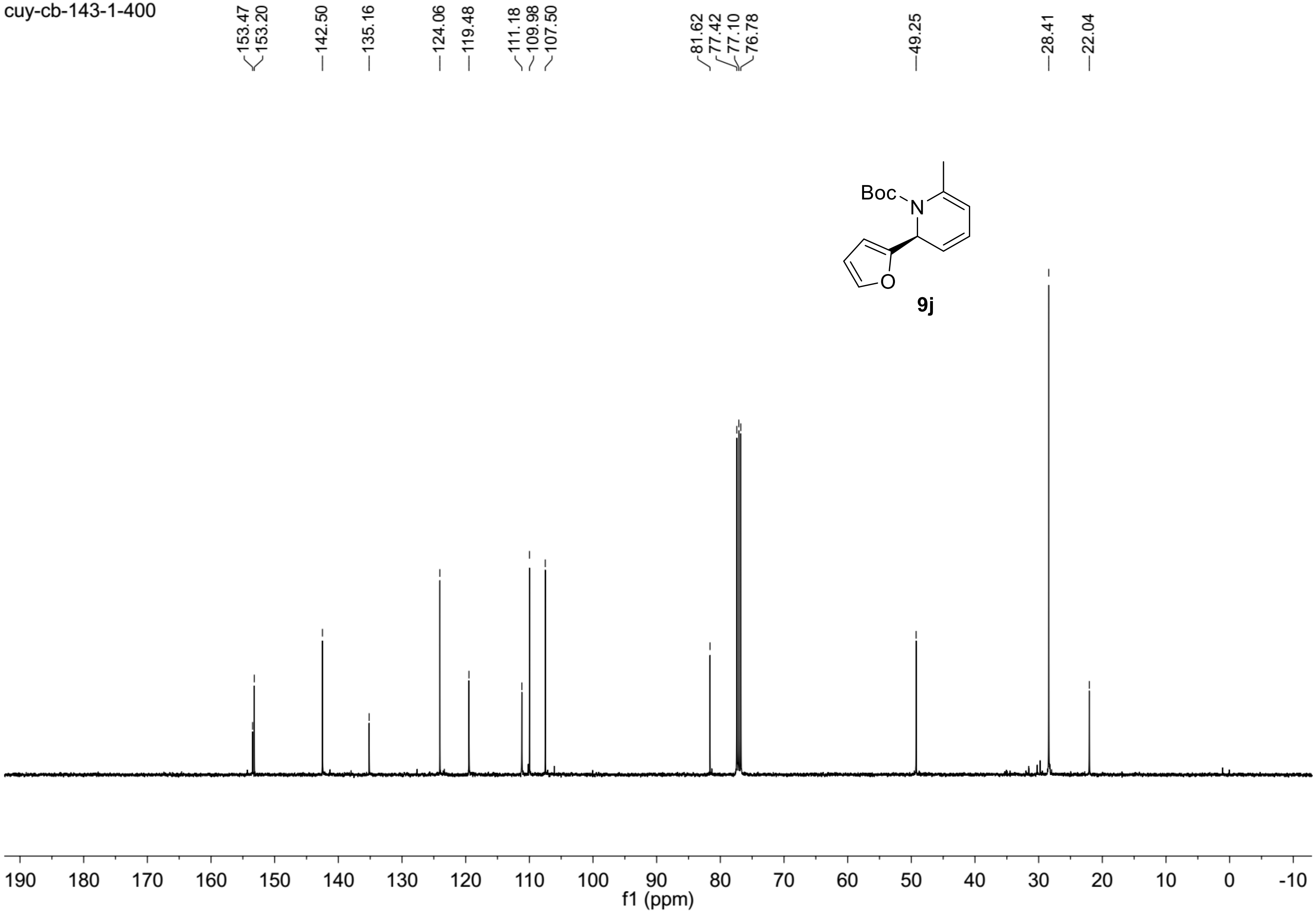

Supplementary Figure 89. ^{13}C NMR (100 MHz, $CDCl_3$) spectra for compound **9j**

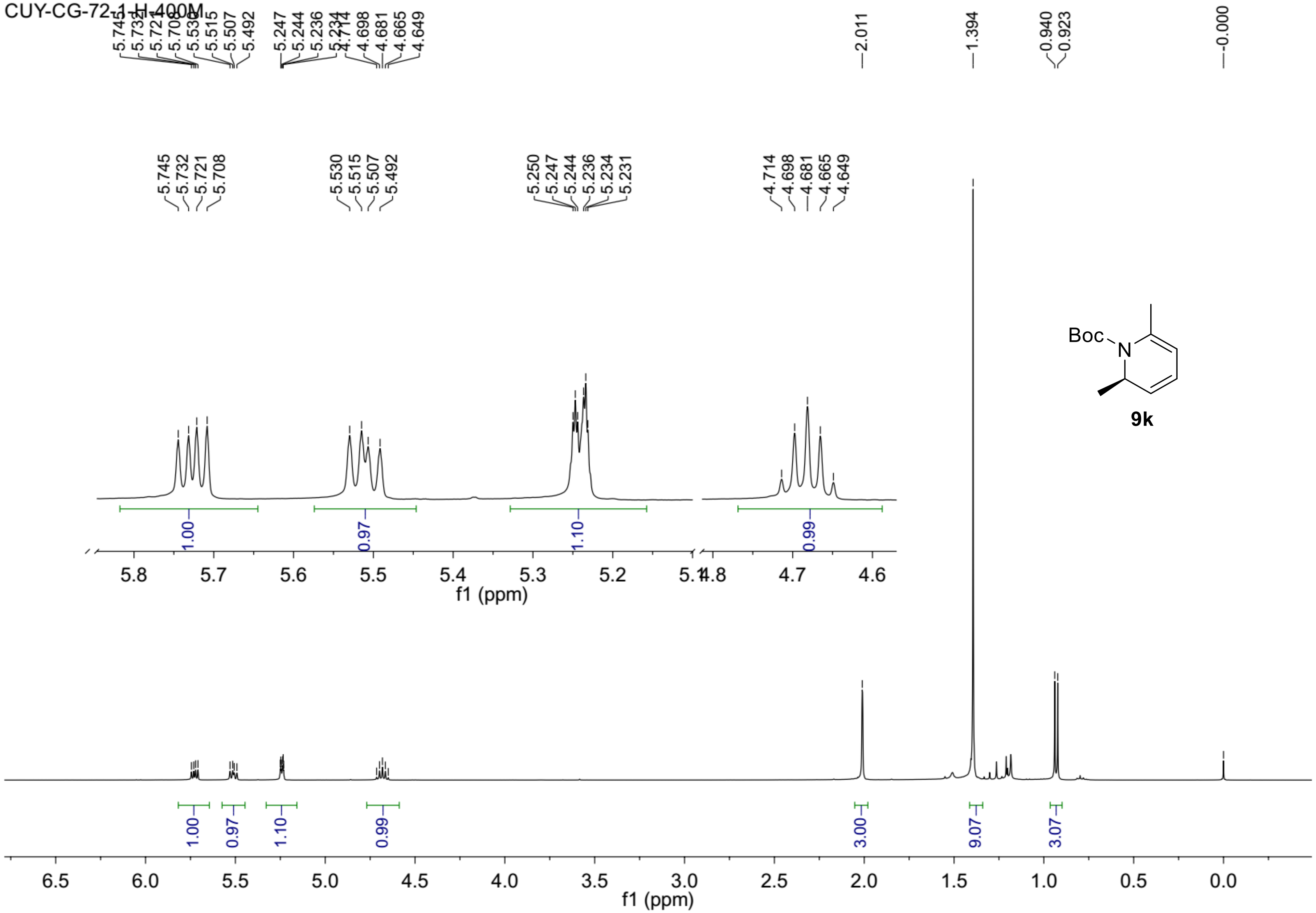

Supplementary Figure 90. ^{1}H NMR (400 MHz, $CDCl_3$) spectra for compound **9k**

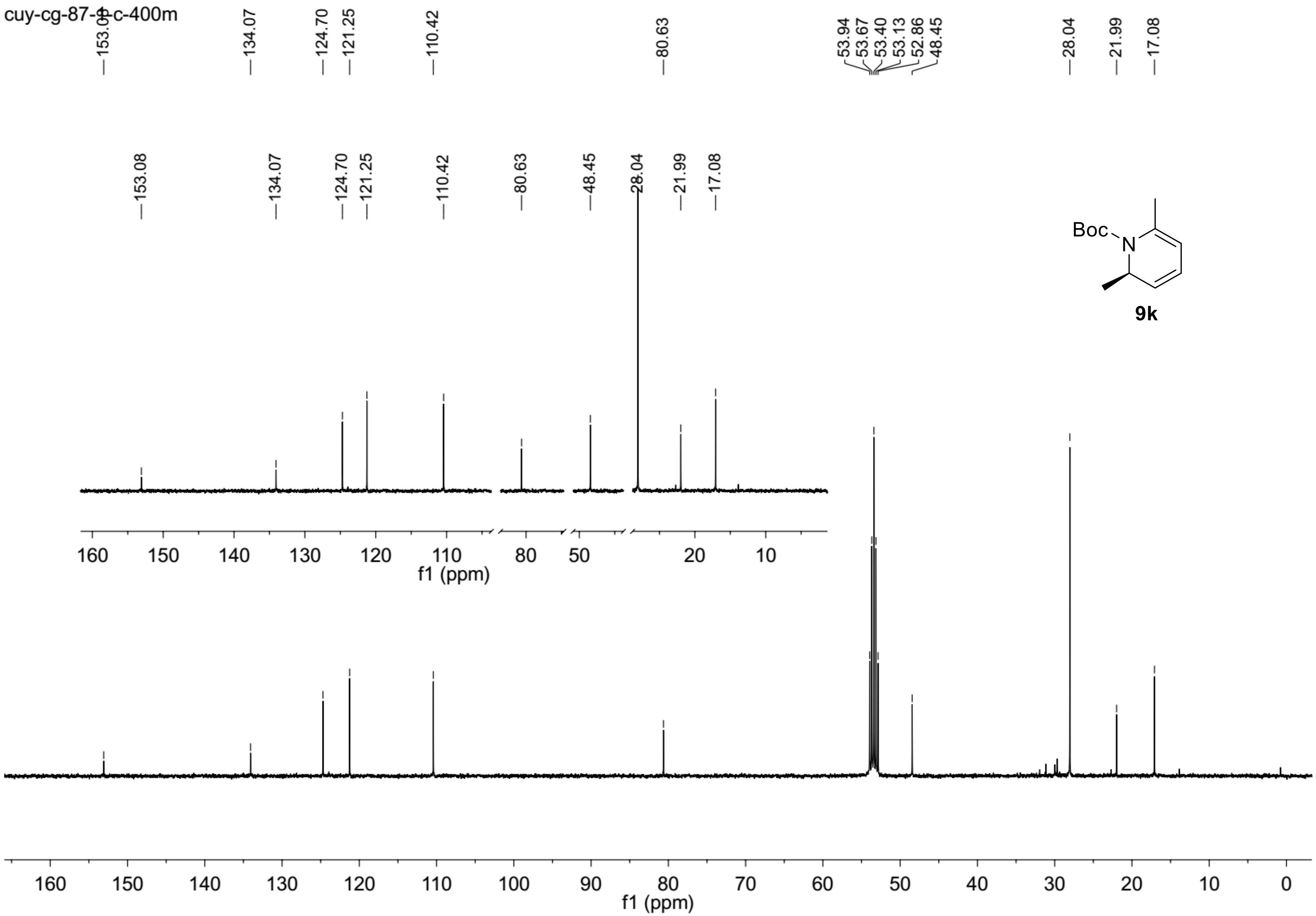

Supplementary Figure 91. ^{13}C NMR (100 MHz, $CDCl_3$) spectra for compound **9k**

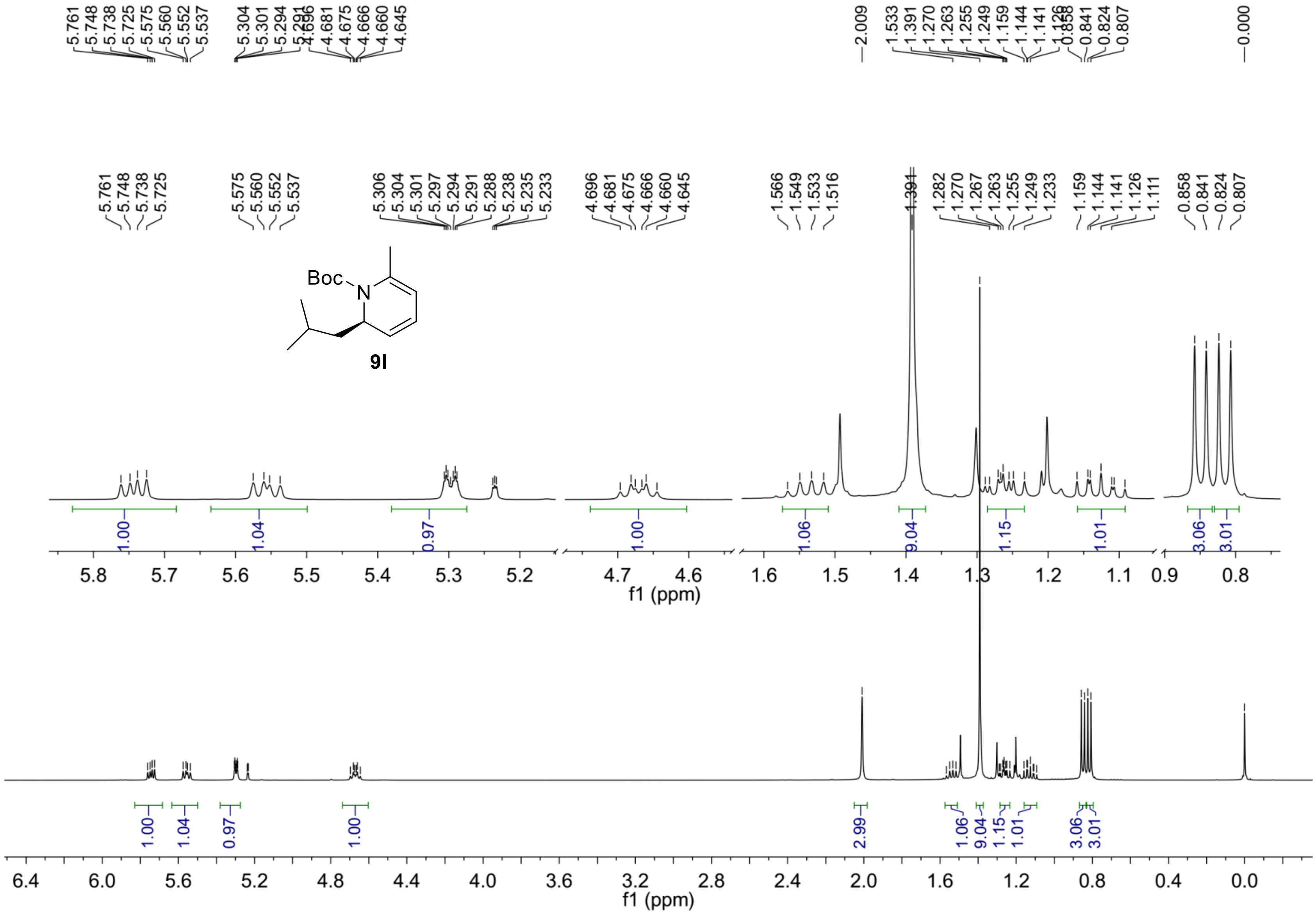

Supplementary Figure 92. ^{1}H NMR (400 MHz, $CDCl_3$) spectra for compound **9l**

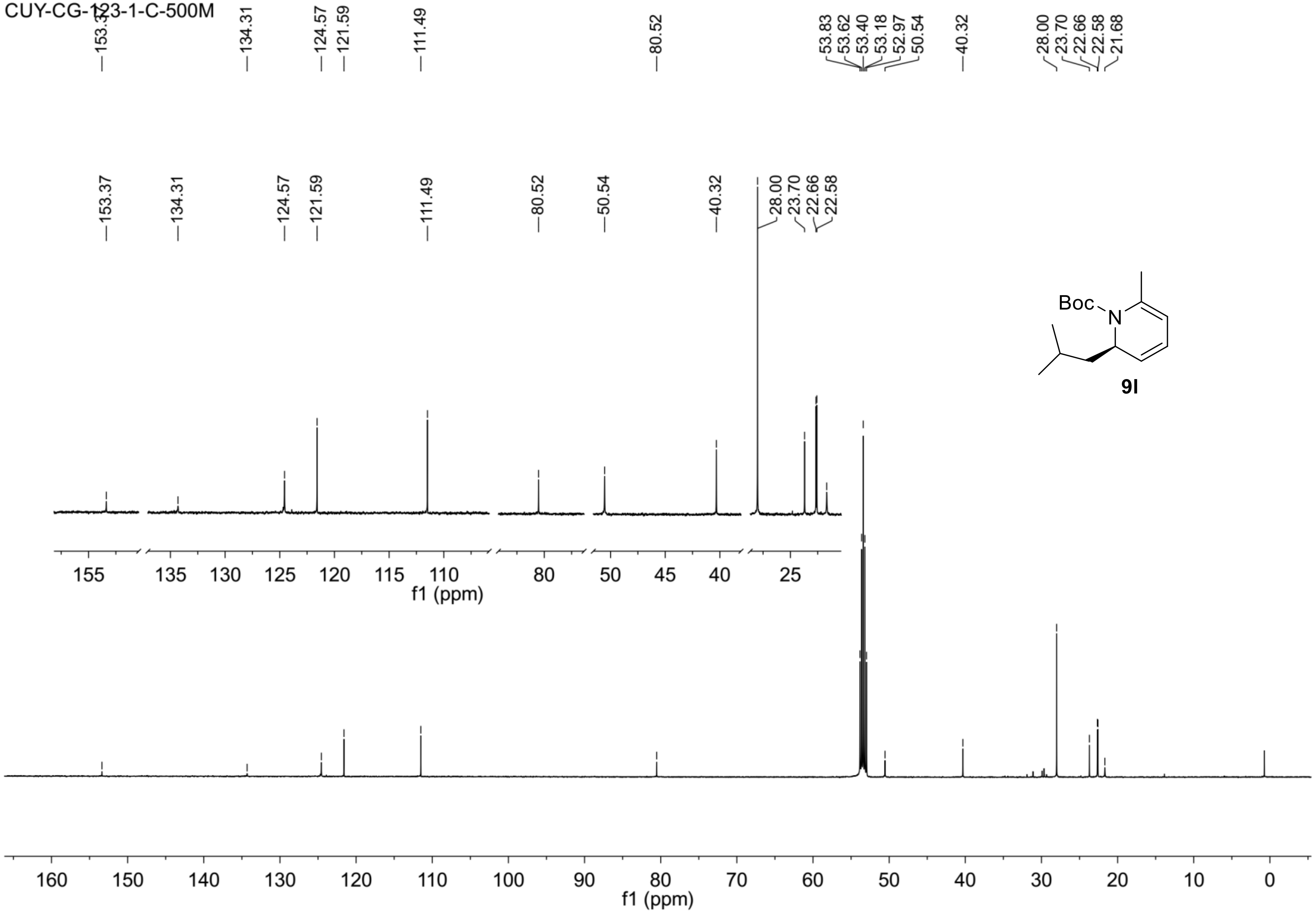

Supplementary Figure 93. ^{13}C NMR (125 MHz, $CDCl_3$) spectra for compound **9l**

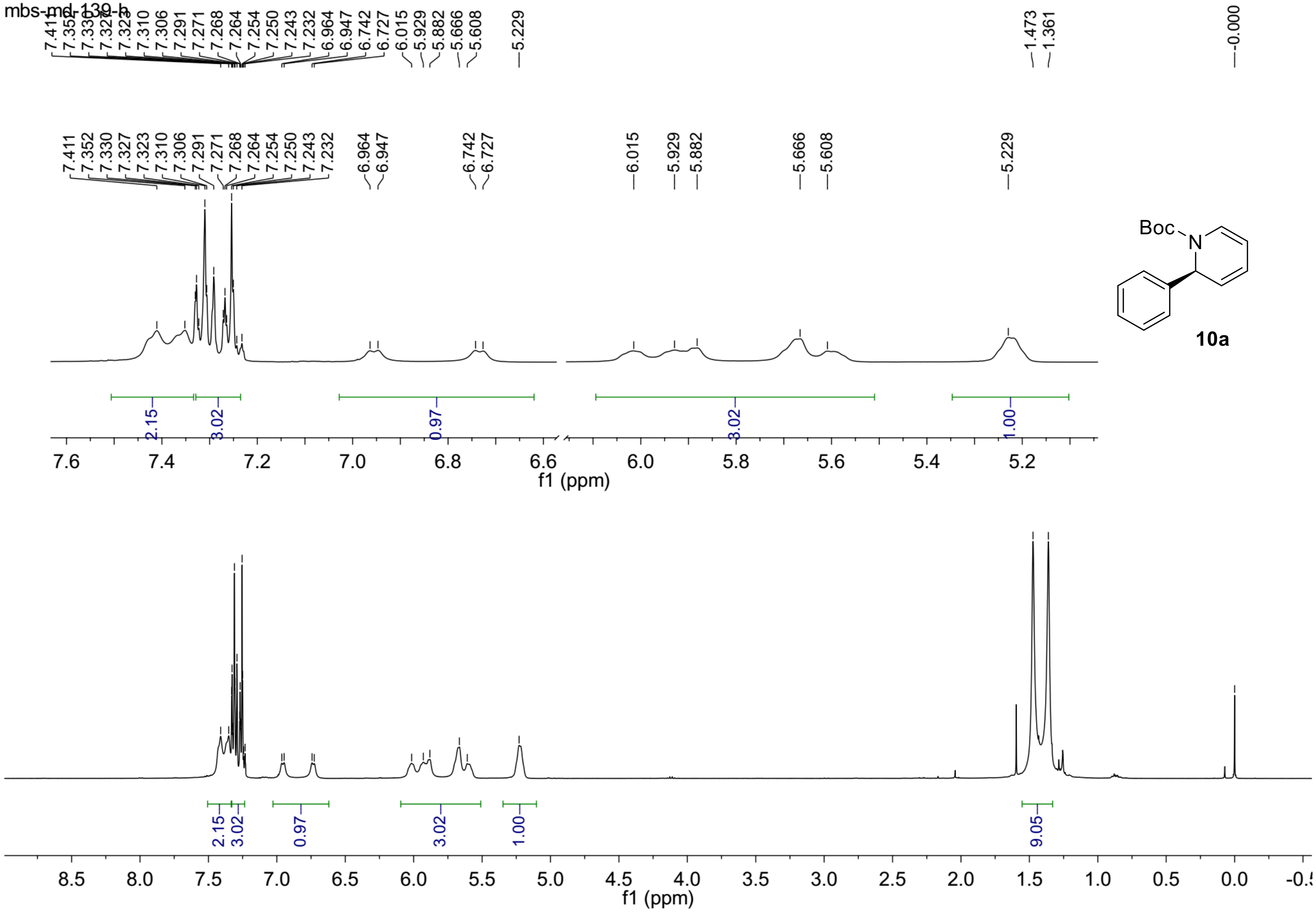

Supplementary Figure 94. ^{1}H NMR (400 MHz, $CDCl_3$) spectra for compound **10a**

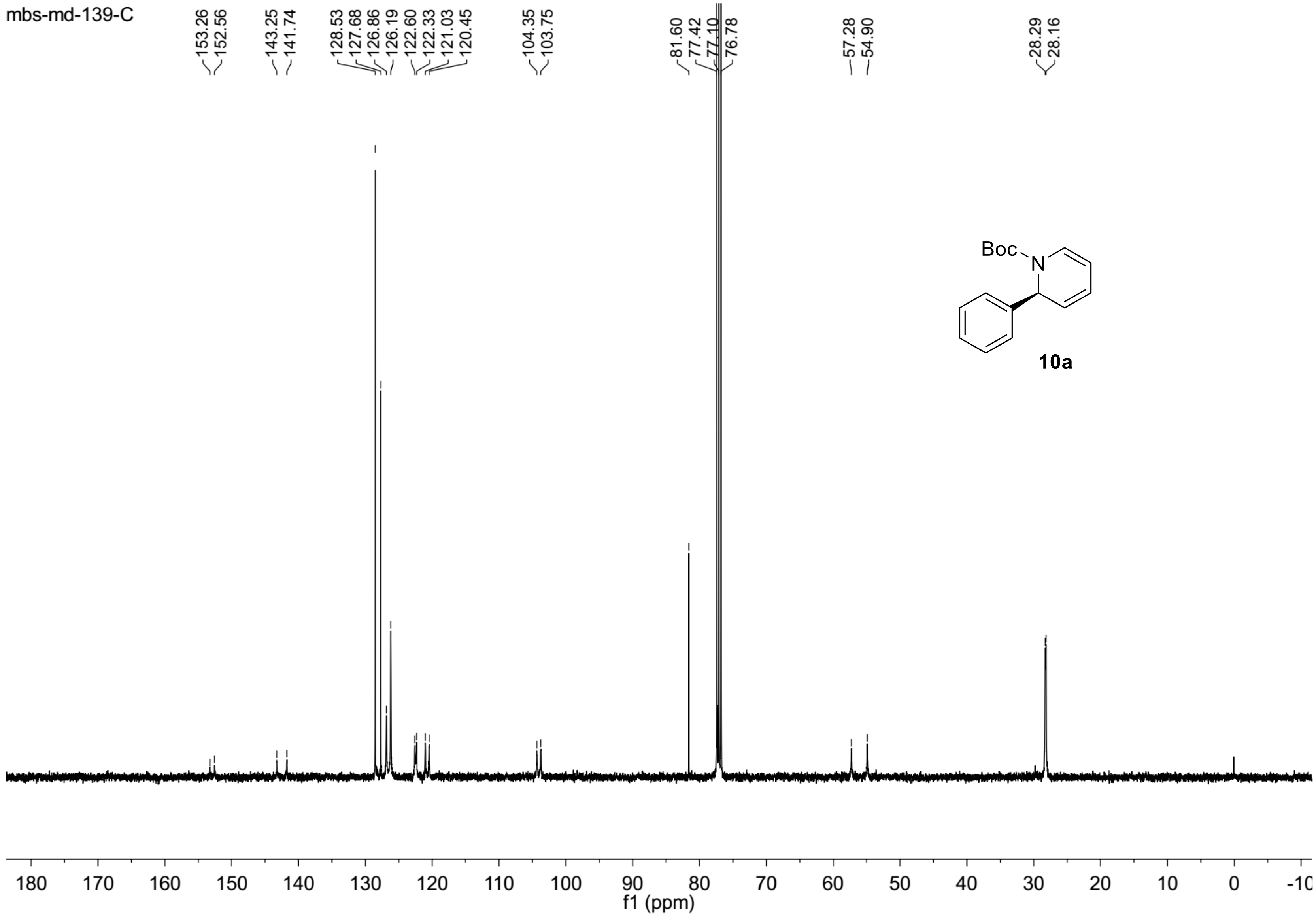

Supplementary Figure 95. ^{13}C NMR (100 MHz, $CDCl_3$) spectra for compound **10a**

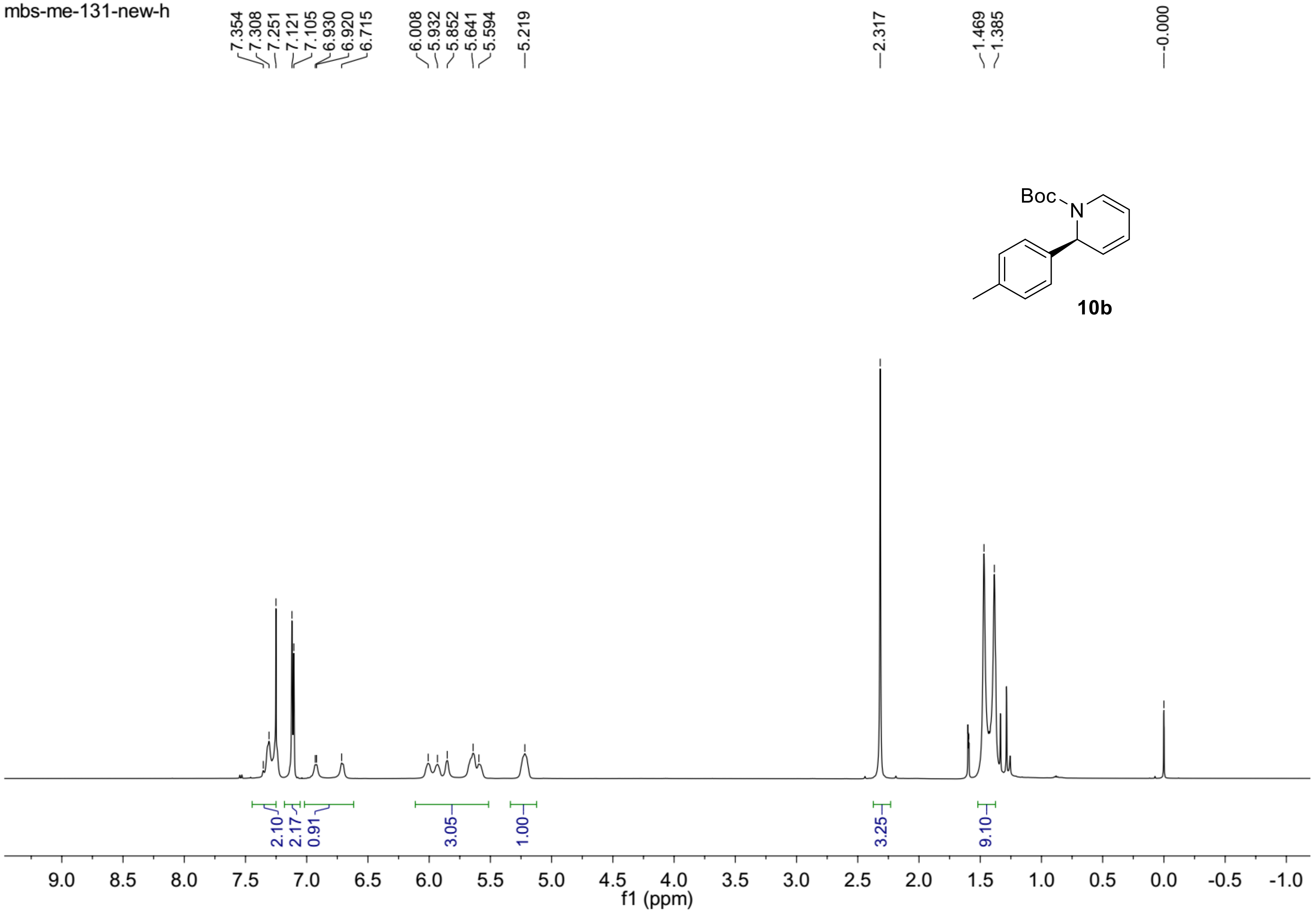

Supplementary Figure 96. ^{1}H NMR (500 MHz, $CDCl_3$) spectra for compound **10b**

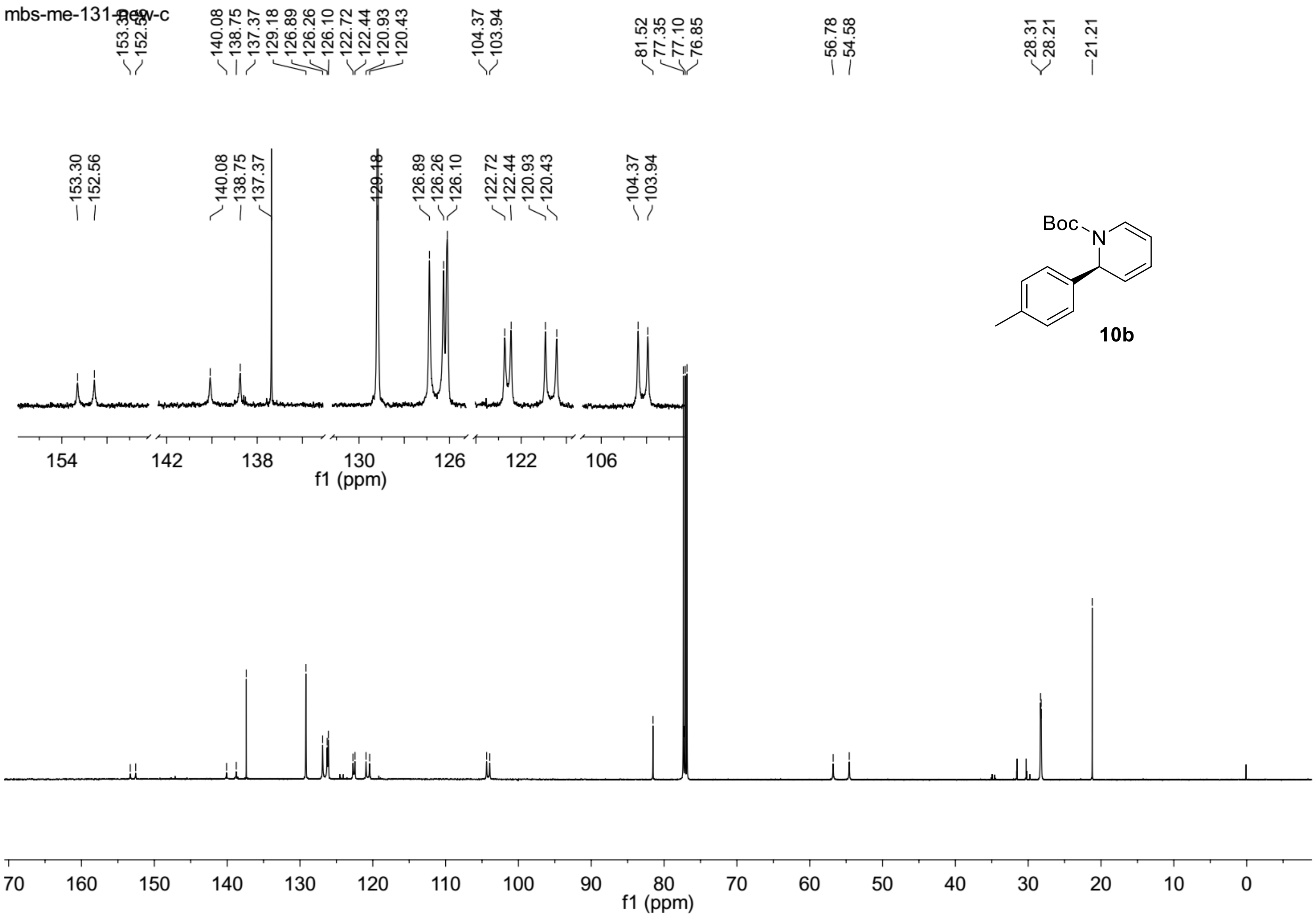

Supplementary Figure 97. ^{13}C NMR (125 MHz, $CDCl_3$) spectra for compound **10b**

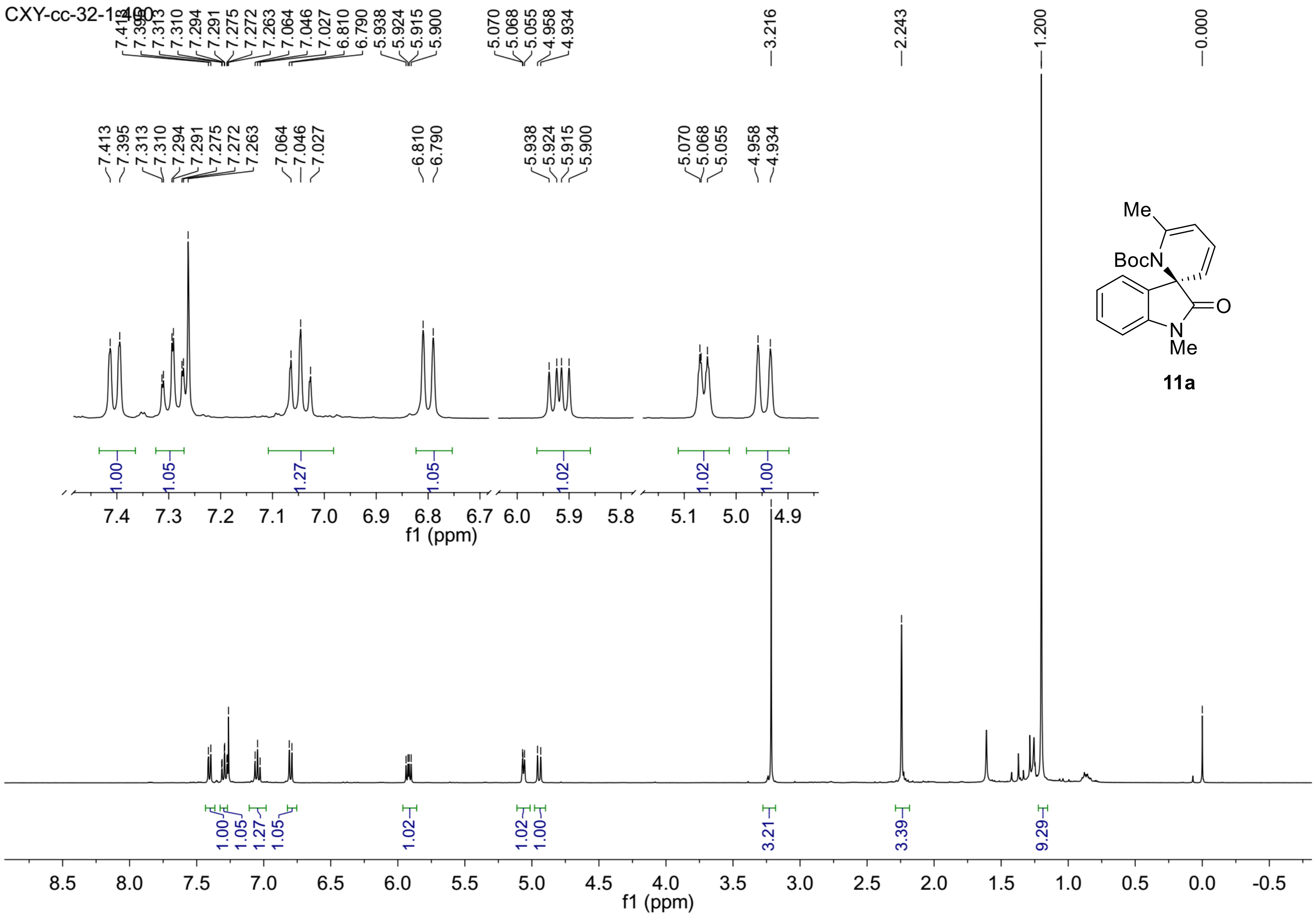

Supplementary Figure 98. ^{1}H NMR (400 MHz, $CDCl_3$) spectra for compound **11a**

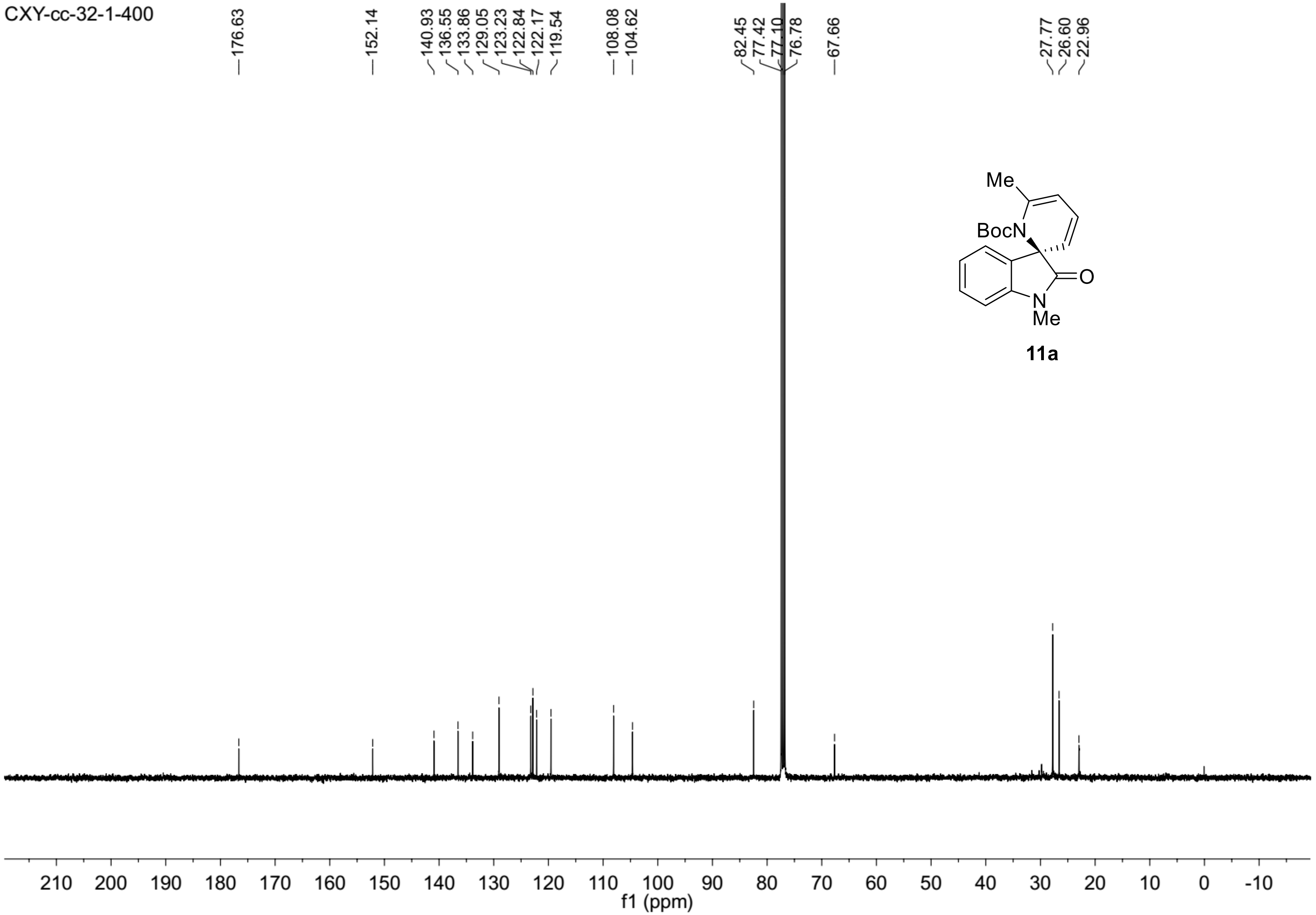

Supplementary Figure 99. ^{13}C NMR (100 MHz, $CDCl_3$) spectra for compound **11a**

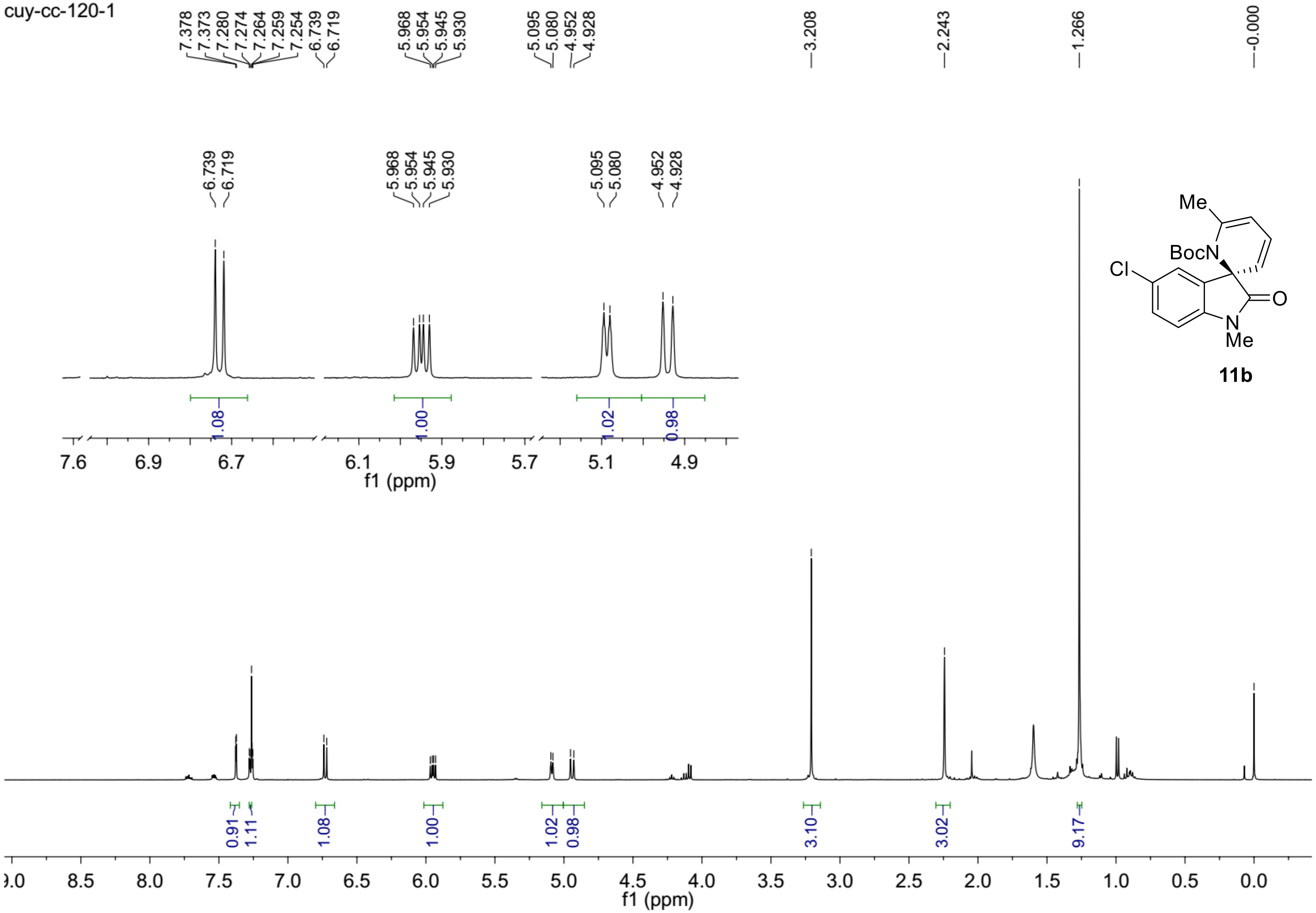

Supplementary Figure 100. ^{1}H NMR (400 MHz, $CDCl_3$) spectra for compound **11b**

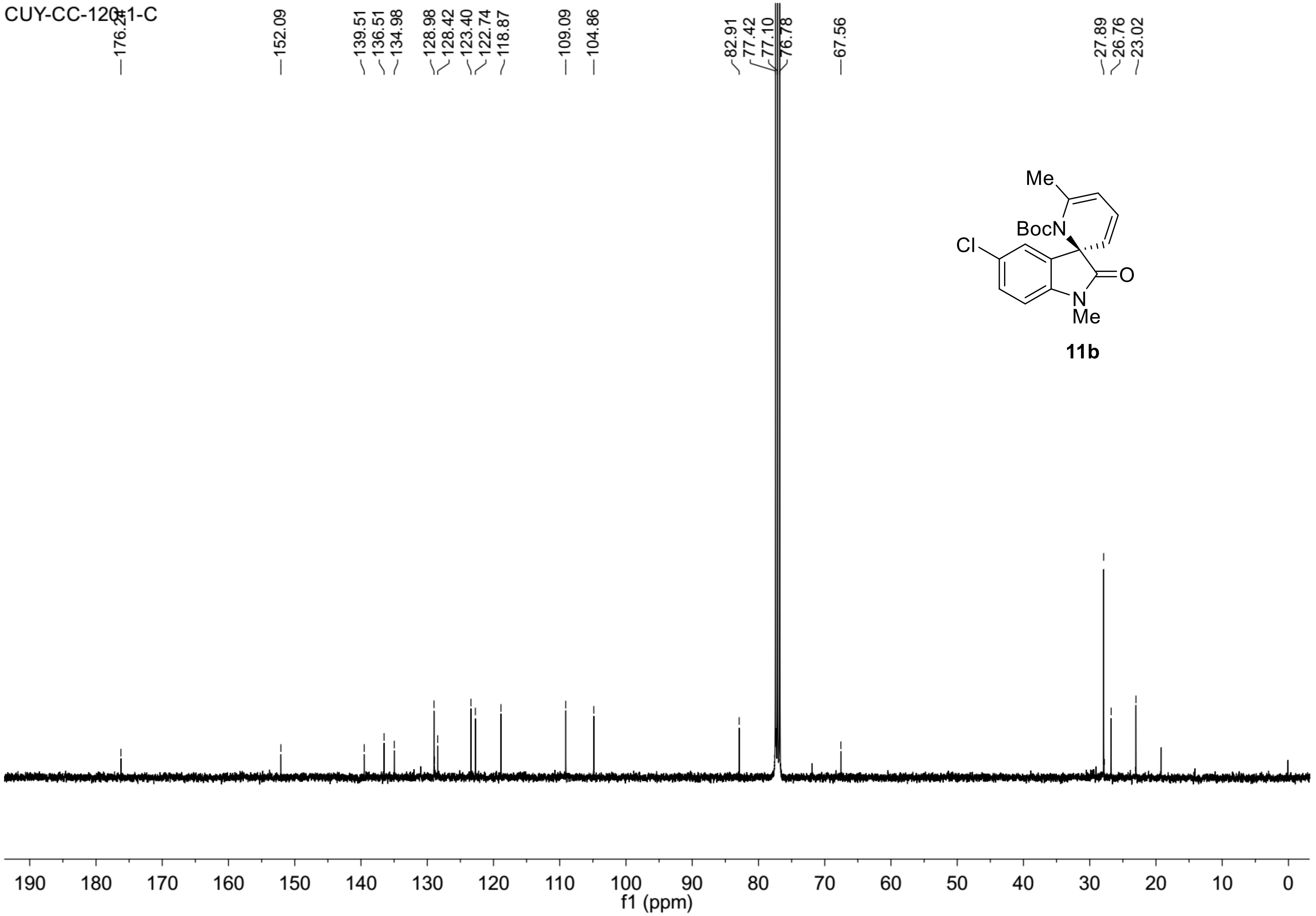

Supplementary Figure 101. ^{13}C NMR (100 MHz, $CDCl_3$) spectra for compound **11b**

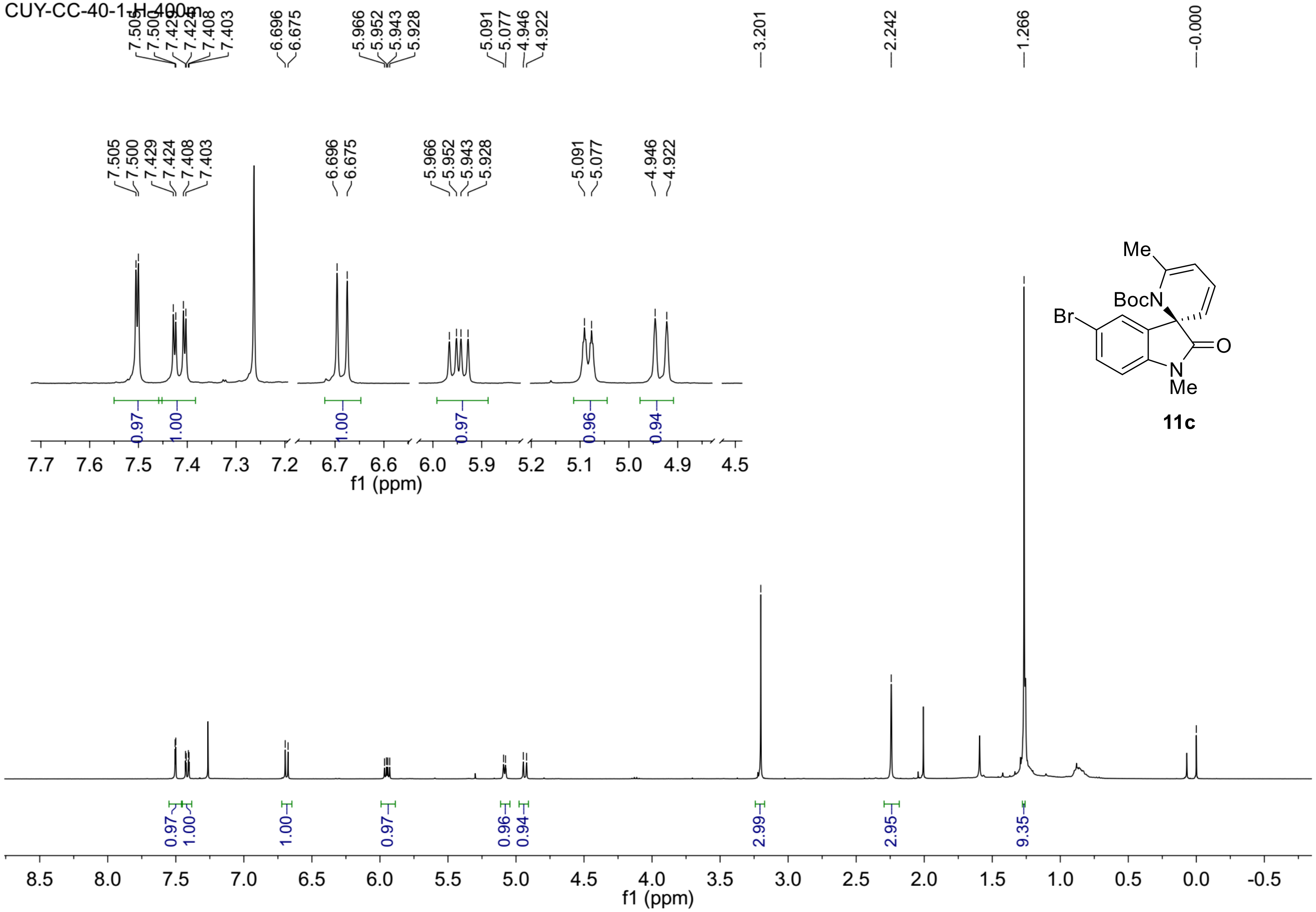

Supplementary Figure 102. ^{1}H NMR (400 MHz, $CDCl_3$) spectra for compound **11c**

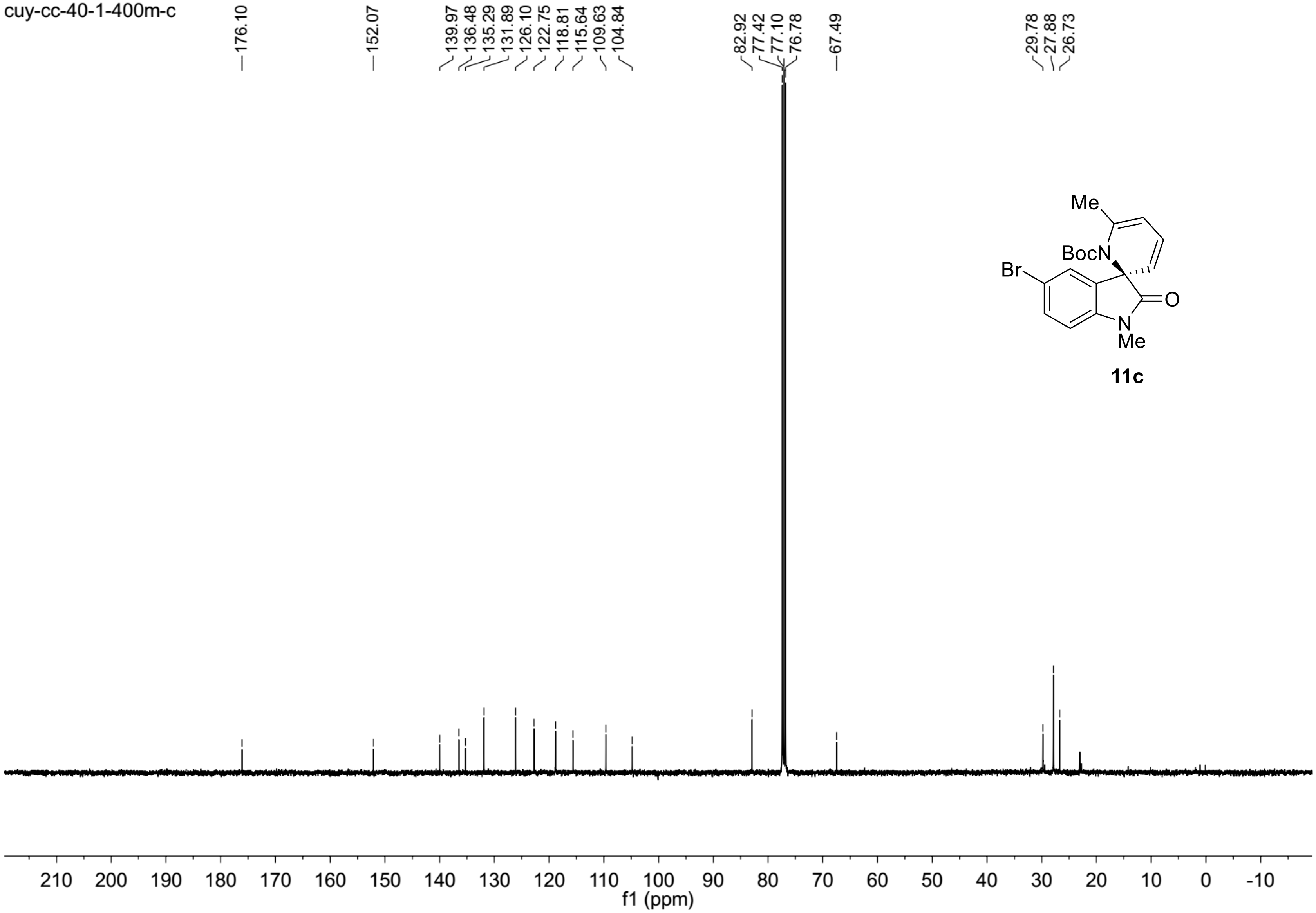

Supplementary Figure 103. ^{13}C NMR (100 MHz, $CDCl_3$) spectra for compound **11c**

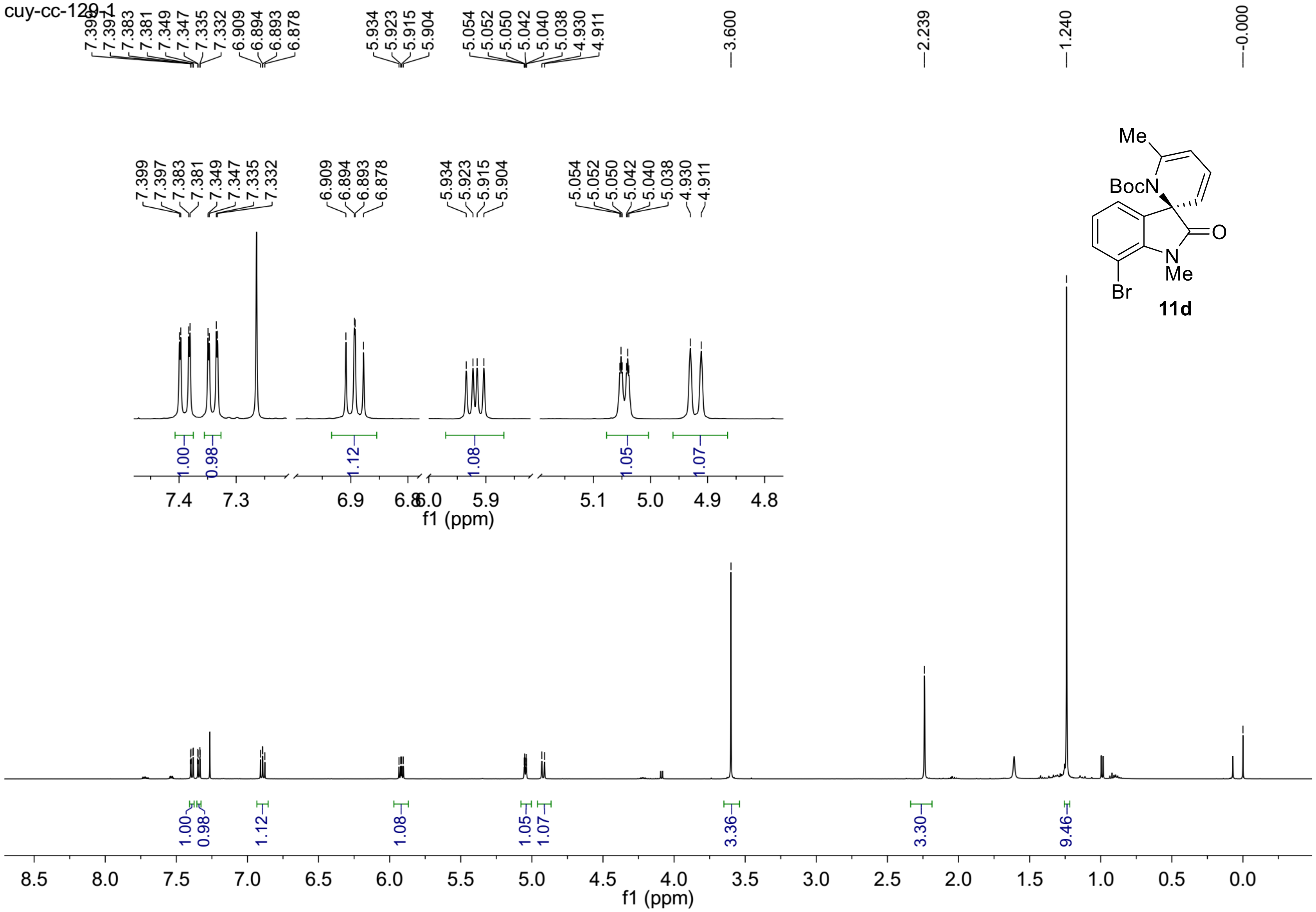

Supplementary Figure 104. ^{1}H NMR (500 MHz, $CDCl_3$) spectra for compound **11d**

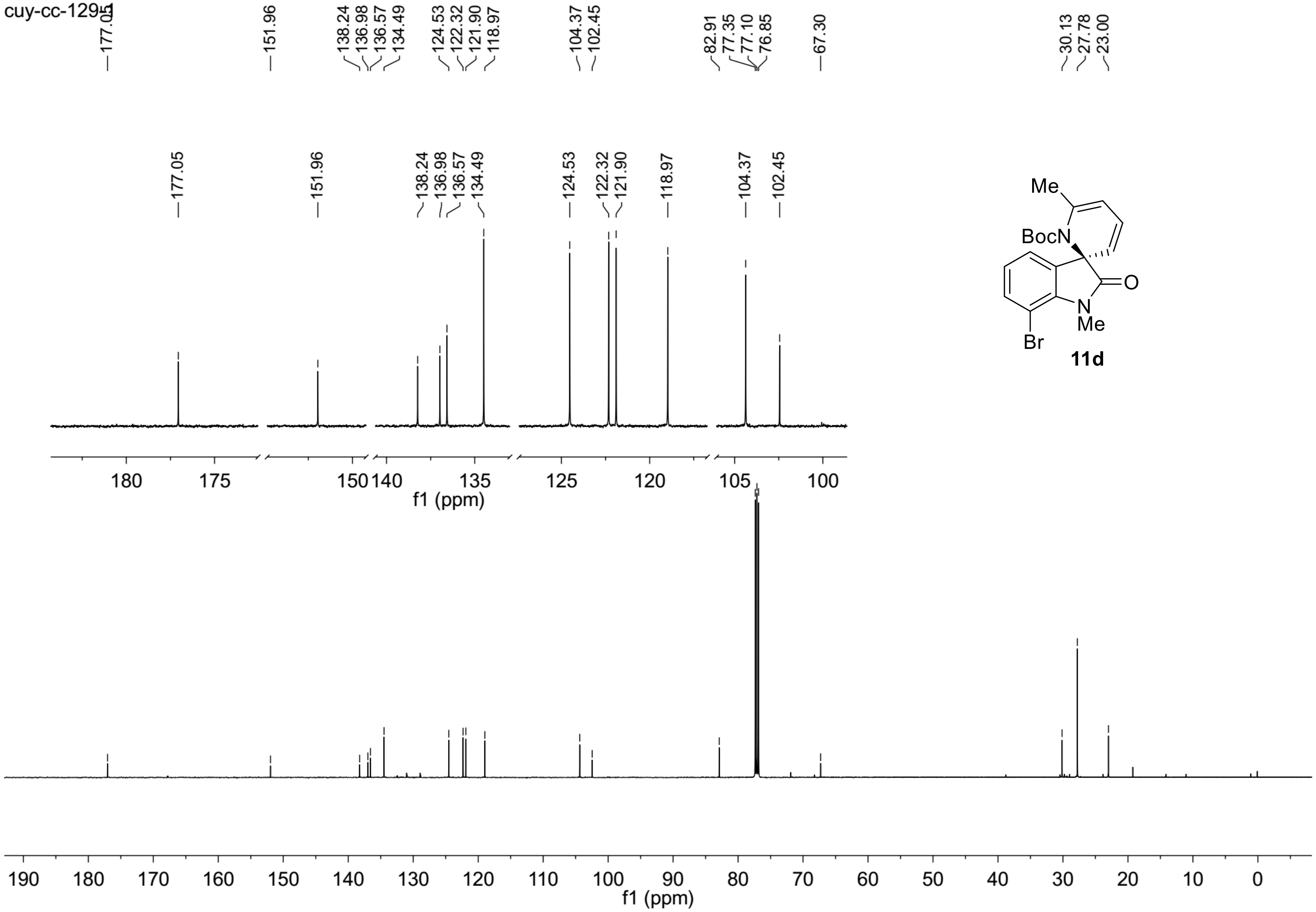

Supplementary Figure 105. ^{13}C NMR (125 MHz, $CDCl_3$) spectra for compound **11d**

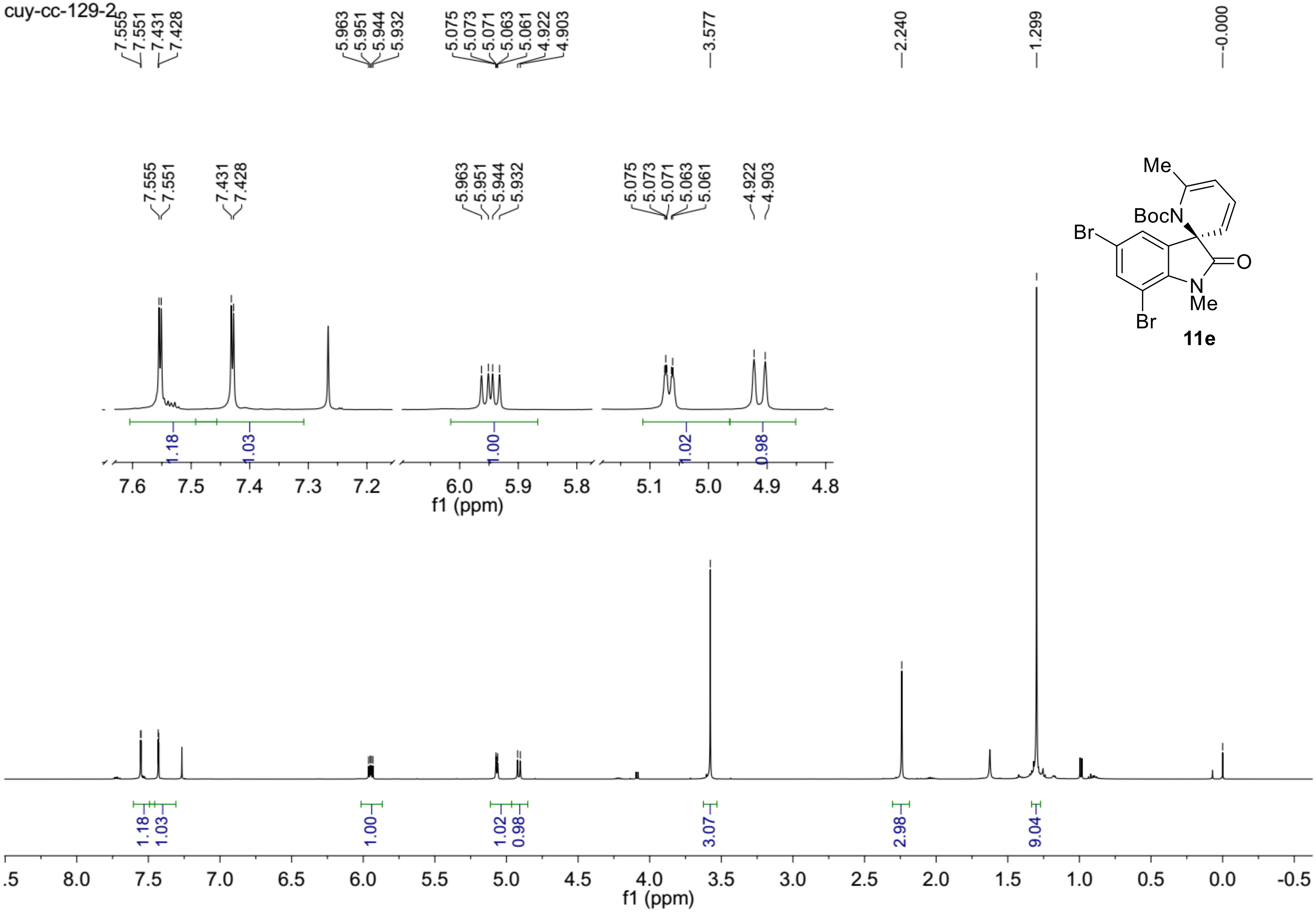

Supplementary Figure 106. ^{1}H NMR (500 MHz, $CDCl_3$) spectra for compound **11e**

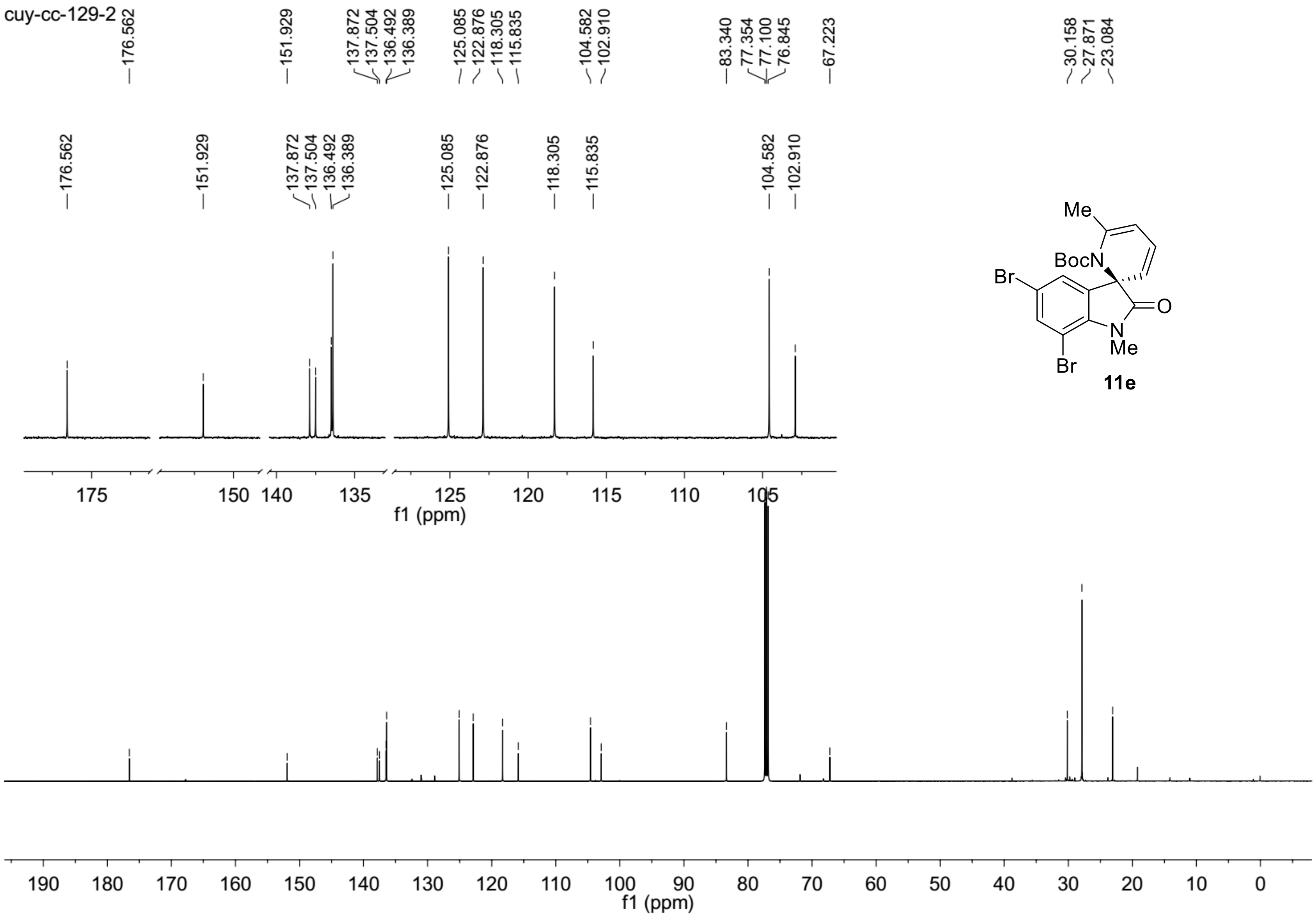

Supplementary Figure 107. ^{13}C NMR (125 MHz, $CDCl_3$) spectra for compound **11e**

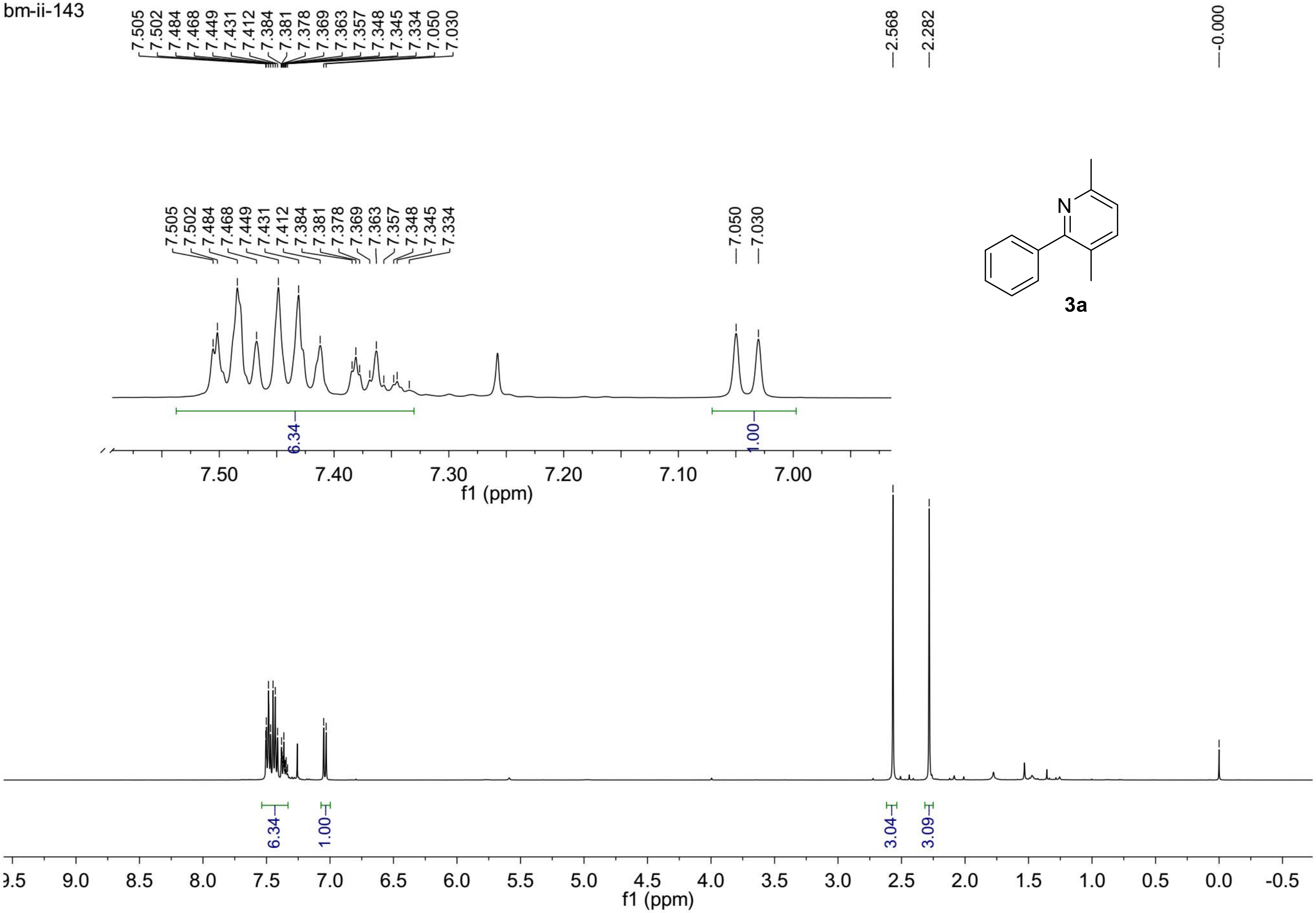

Supplementary Figure 108. ^{1}H NMR (400 MHz, $CDCl_3$) spectra for compound **3a**

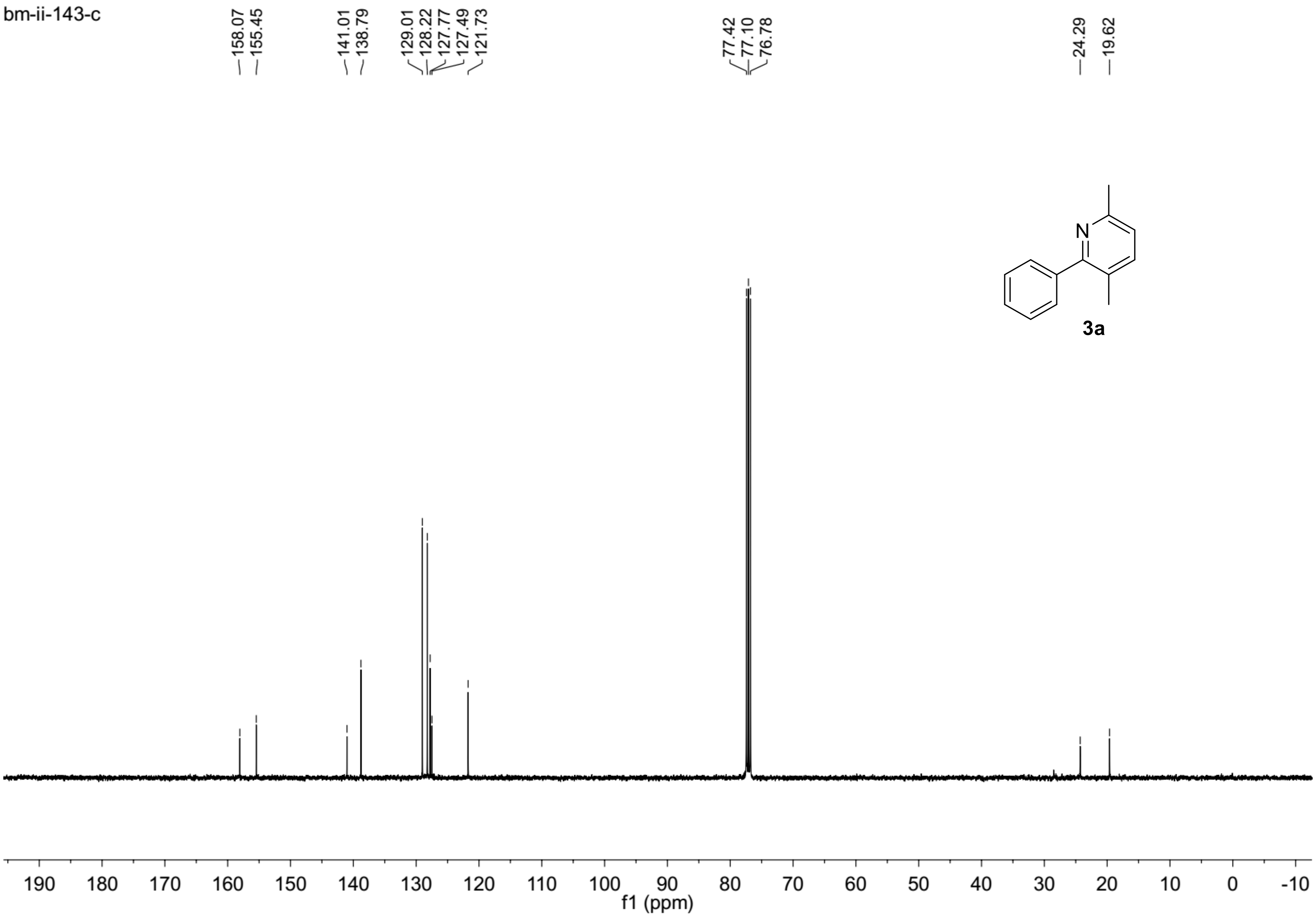

Supplementary Figure 109. ^{13}C NMR (100 MHz, $CDCl_3$) spectra for compound **3a**

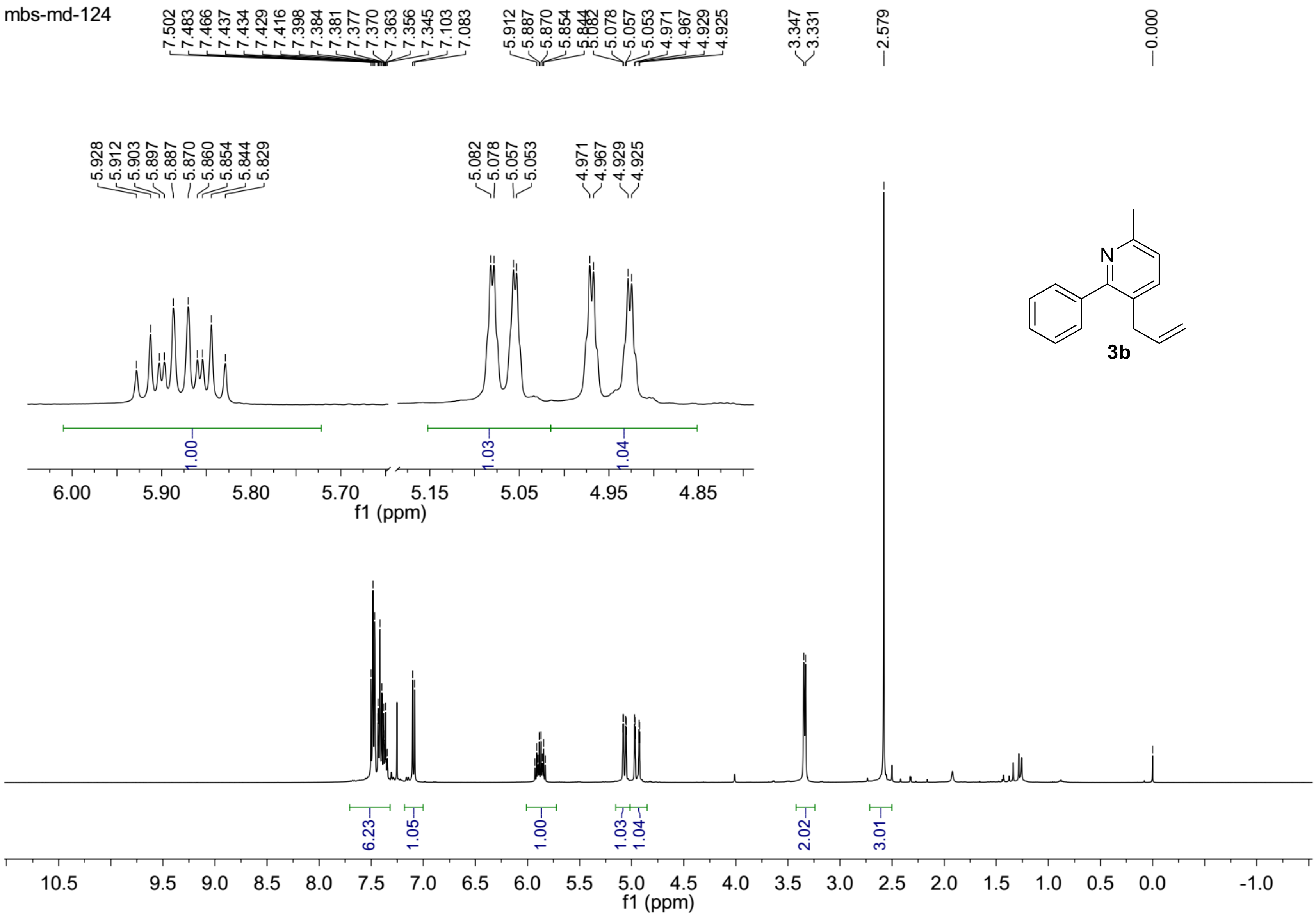

Supplementary Figure 110. ^{1}H NMR (400 MHz, $CDCl_3$) spectra for compound **3b**

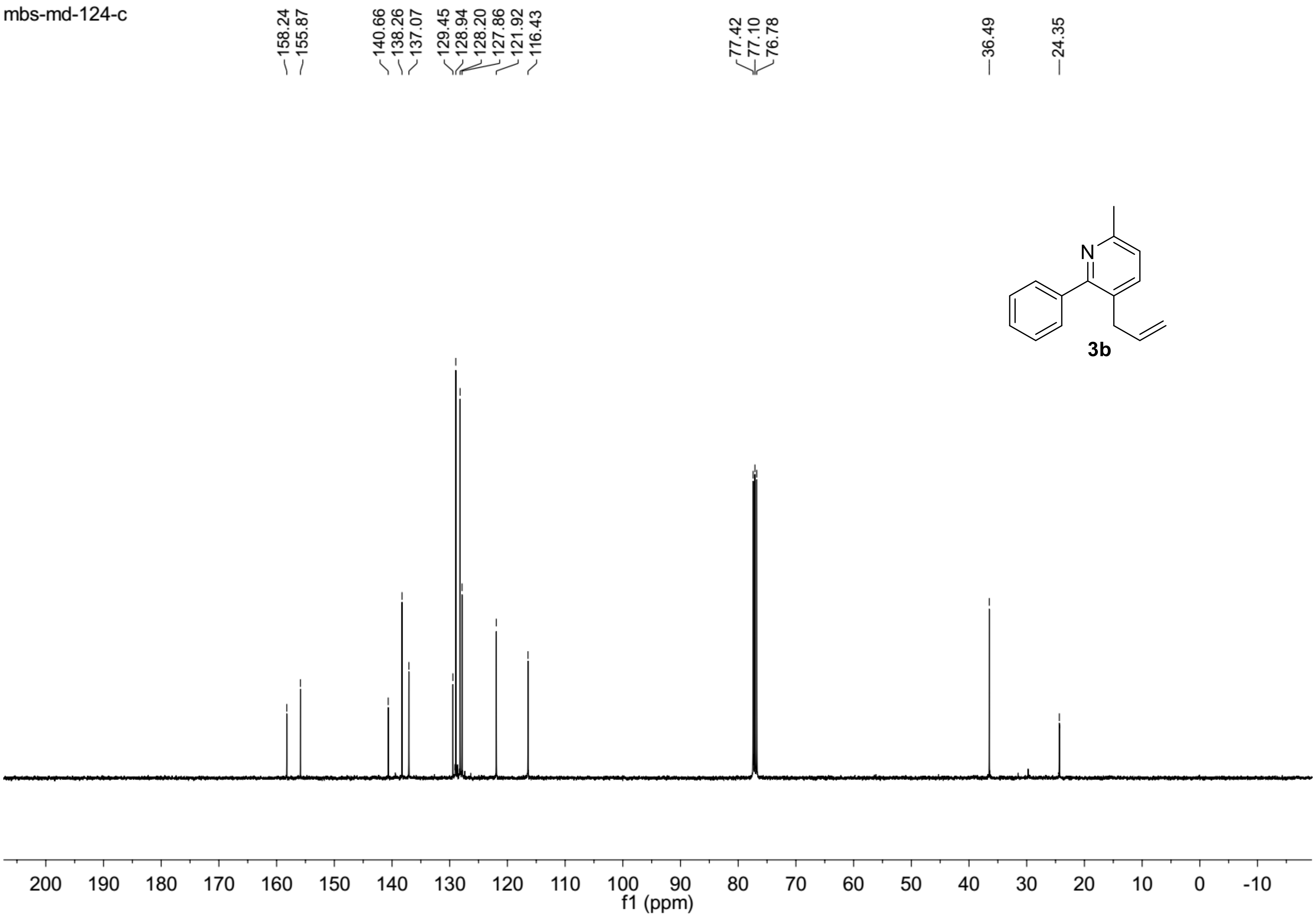

Supplementary Figure 111. ^{13}C NMR (100 MHz, $CDCl_3$) spectra for compound **3b**

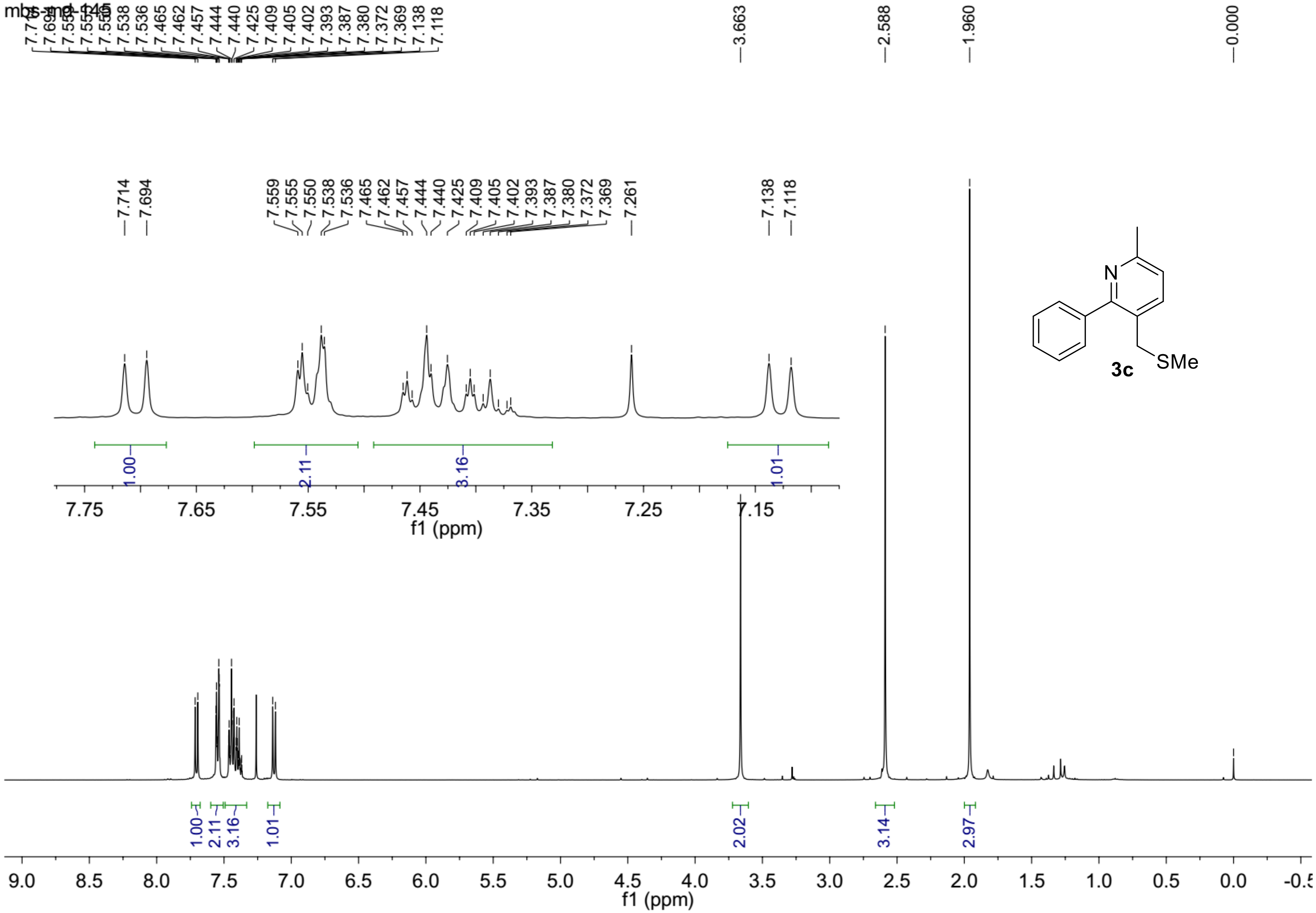

Supplementary Figure 112. ^{1}H NMR (400 MHz, $CDCl_3$) spectra for compound **3c**

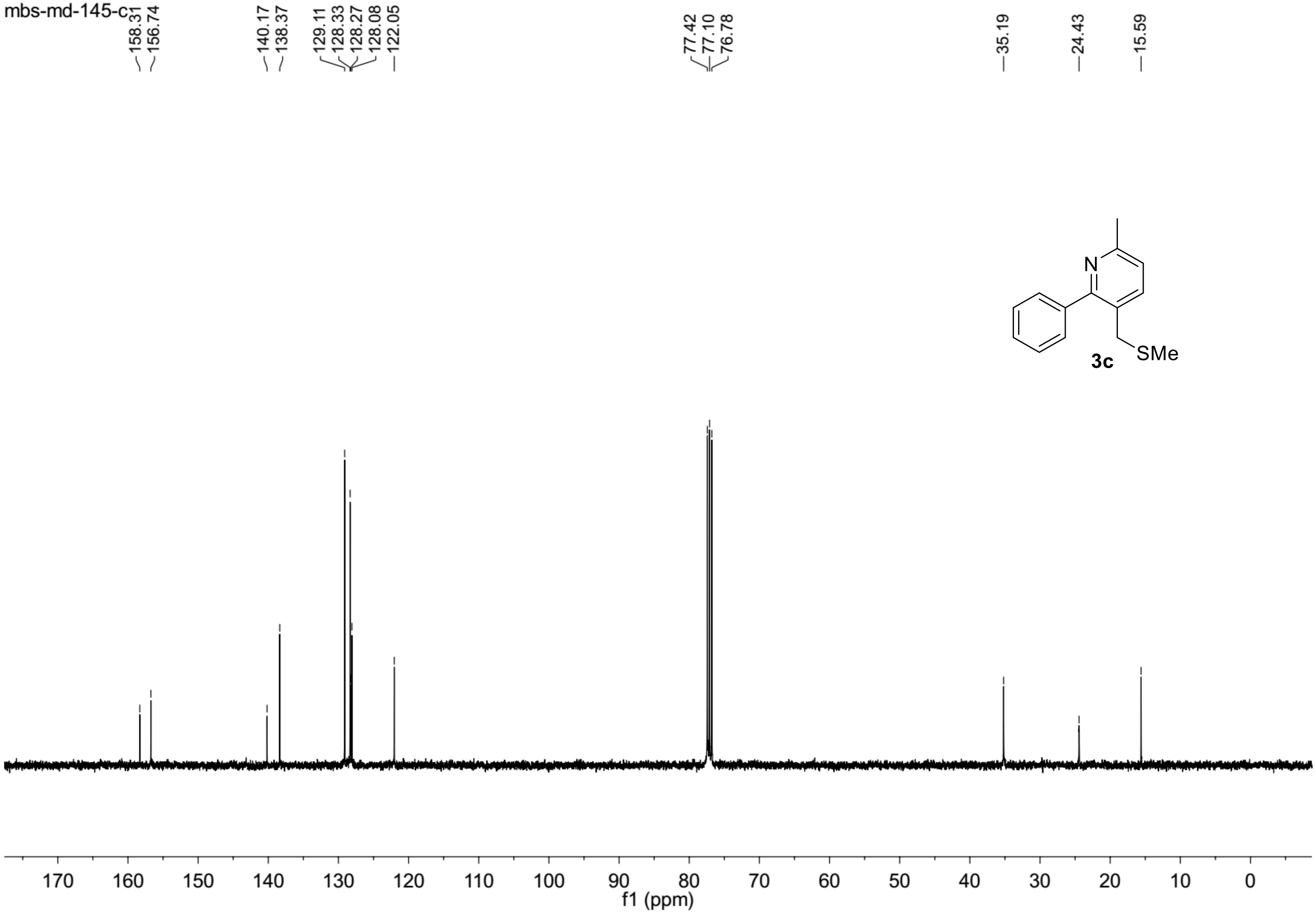

Supplementary Figure 113. ^{13}C NMR (100 MHz, $CDCl_3$) spectra for compound **3c**

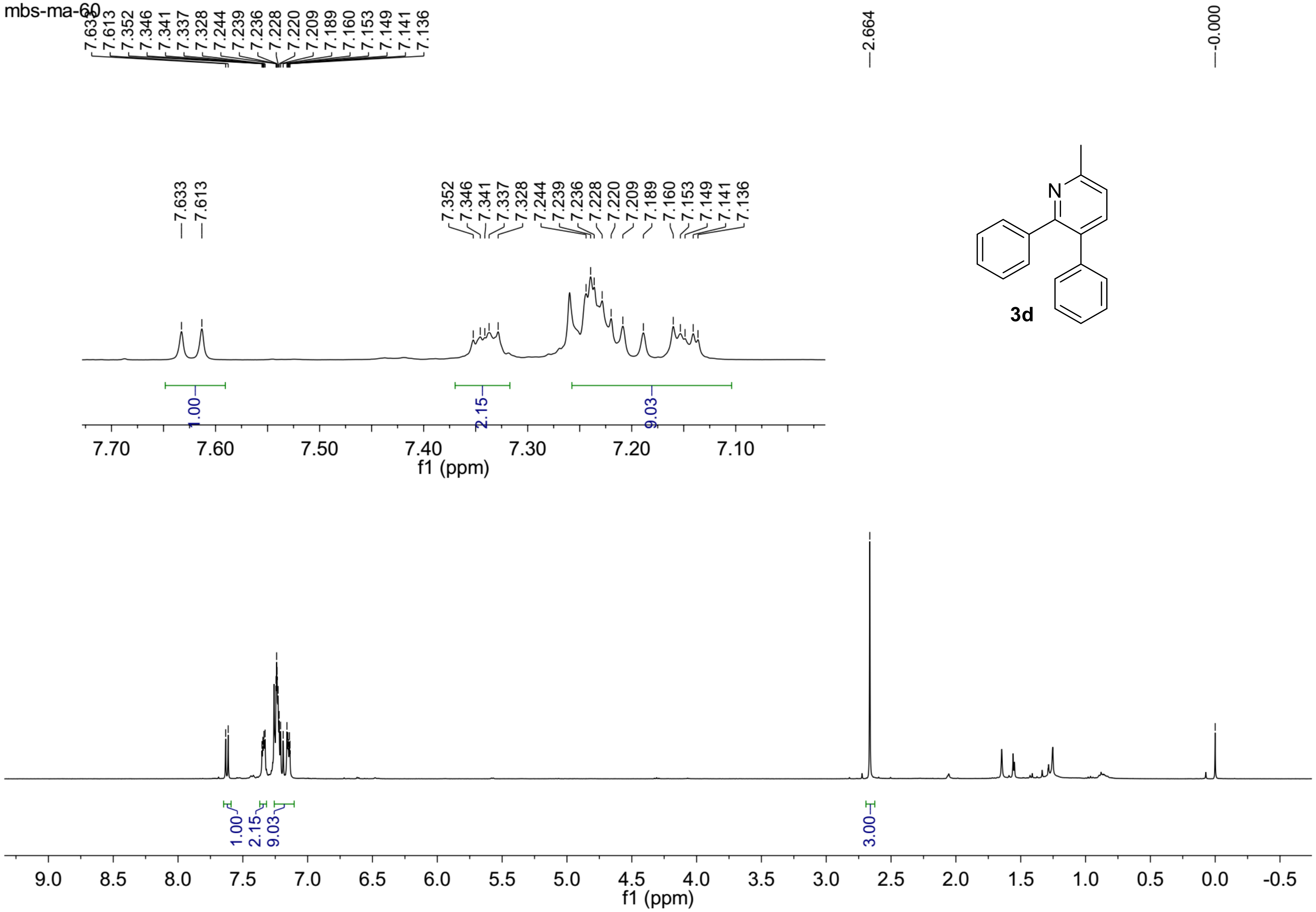

Supplementary Figure 114. ^{1}H NMR (400 MHz, $CDCl_3$) spectra for compound **3d**

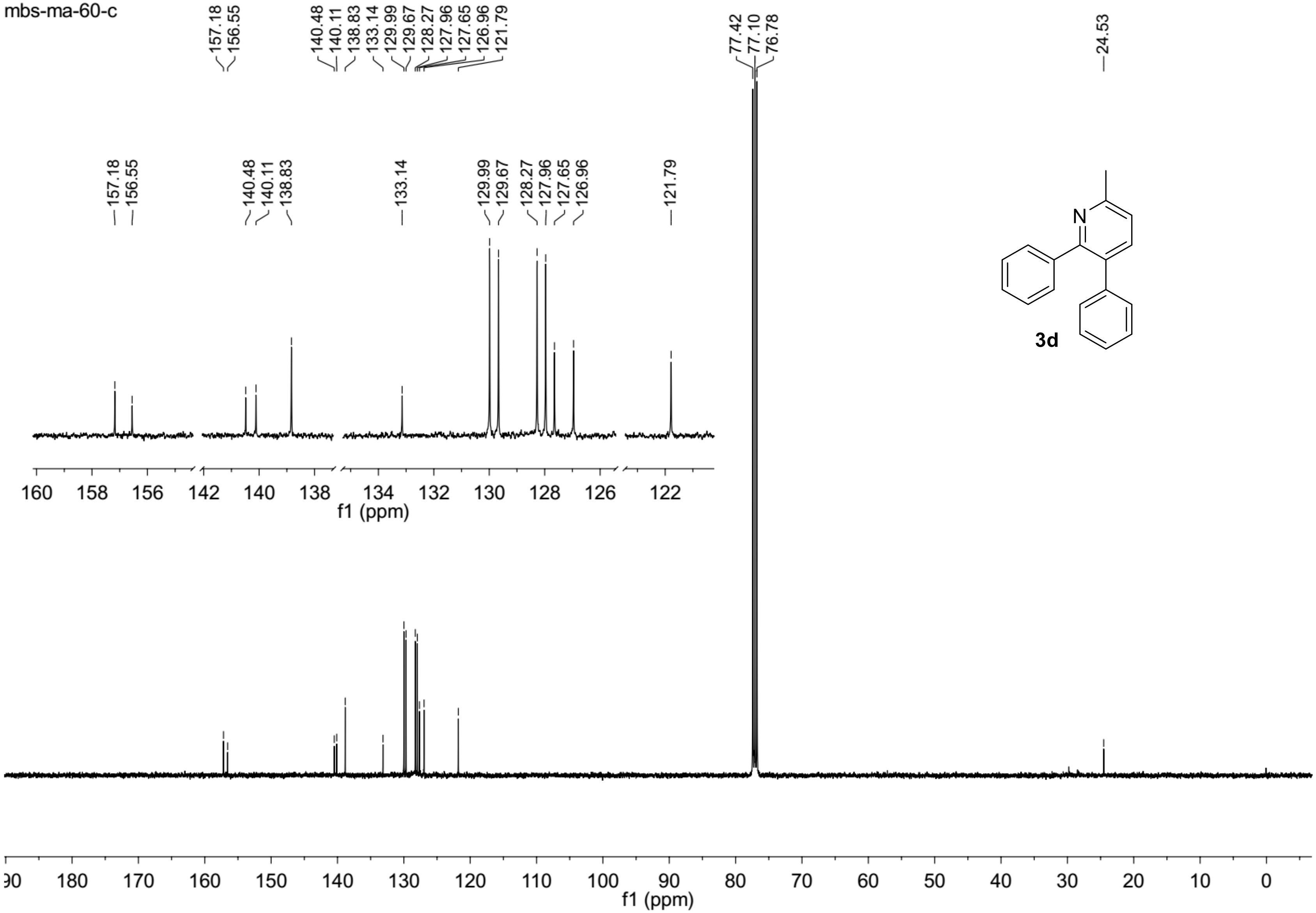

Supplementary Figure 115. ^{13}C NMR (100 MHz, $CDCl_3$) spectra for compound **3d**

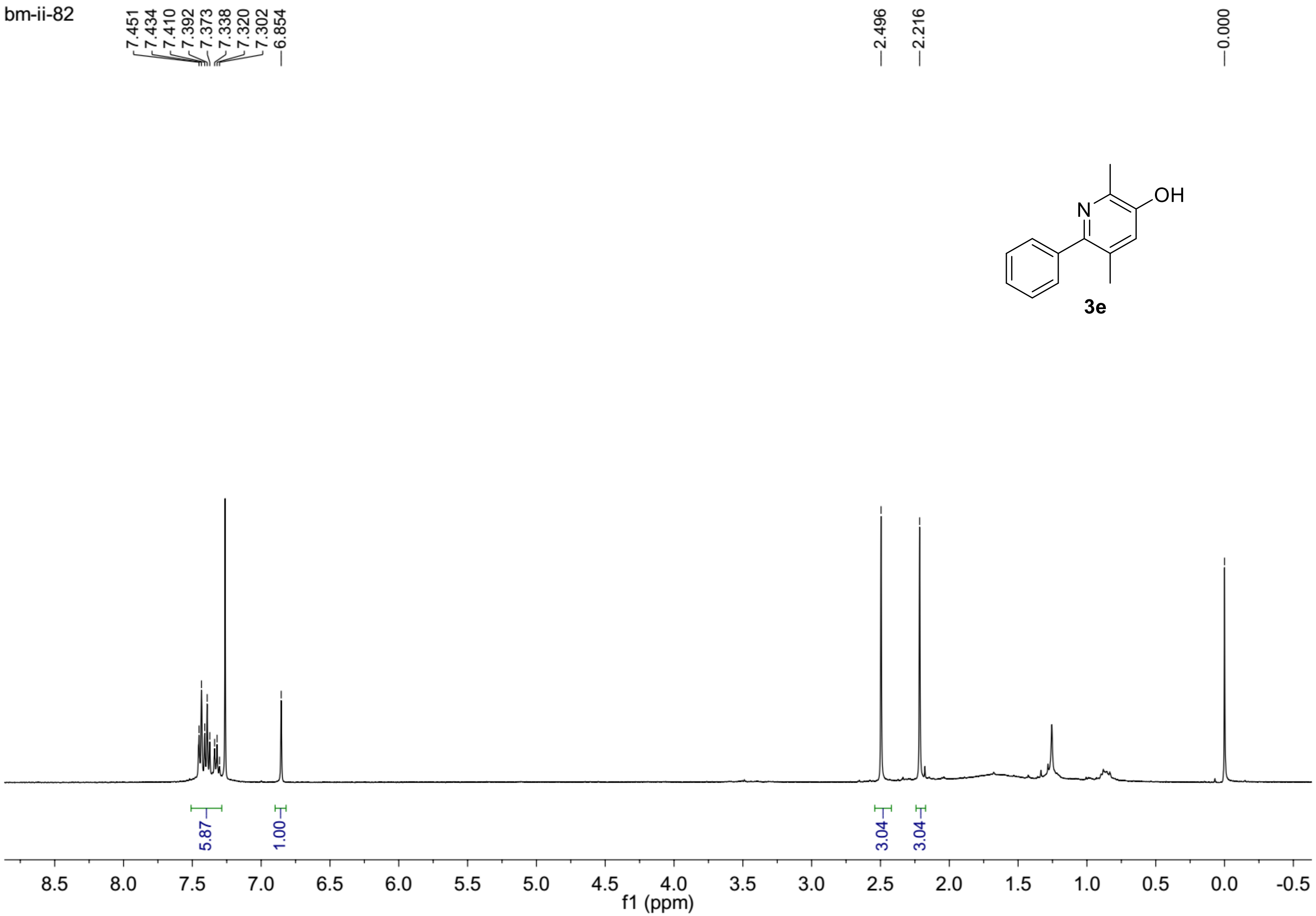

Supplementary Figure 116. ^{1}H NMR (400 MHz, $CDCl_3$) spectra for compound **3e**

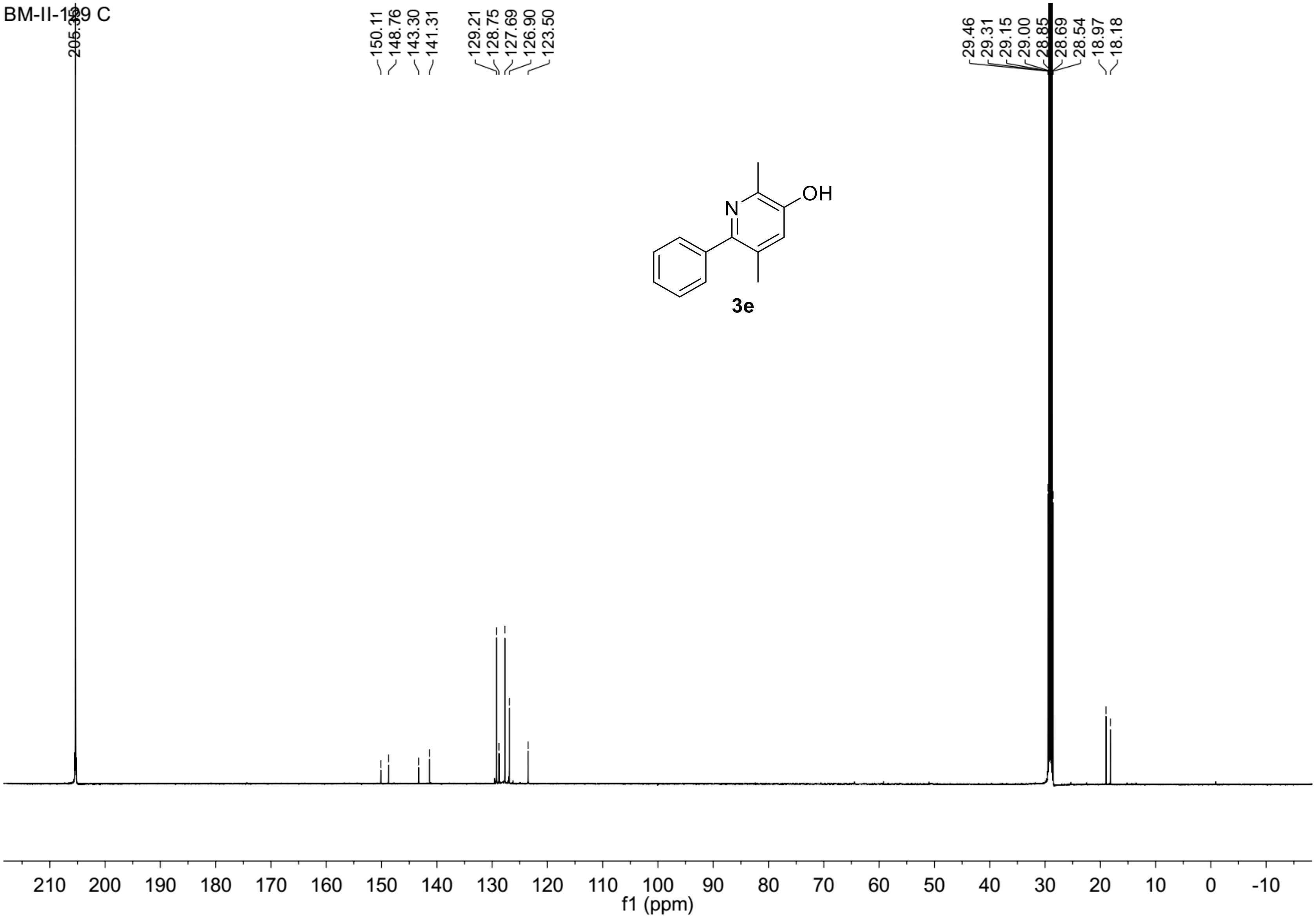

Supplementary Figure 117. ^{13}C NMR (125 MHz, $CDCl_3$) spectra for compound **3e**

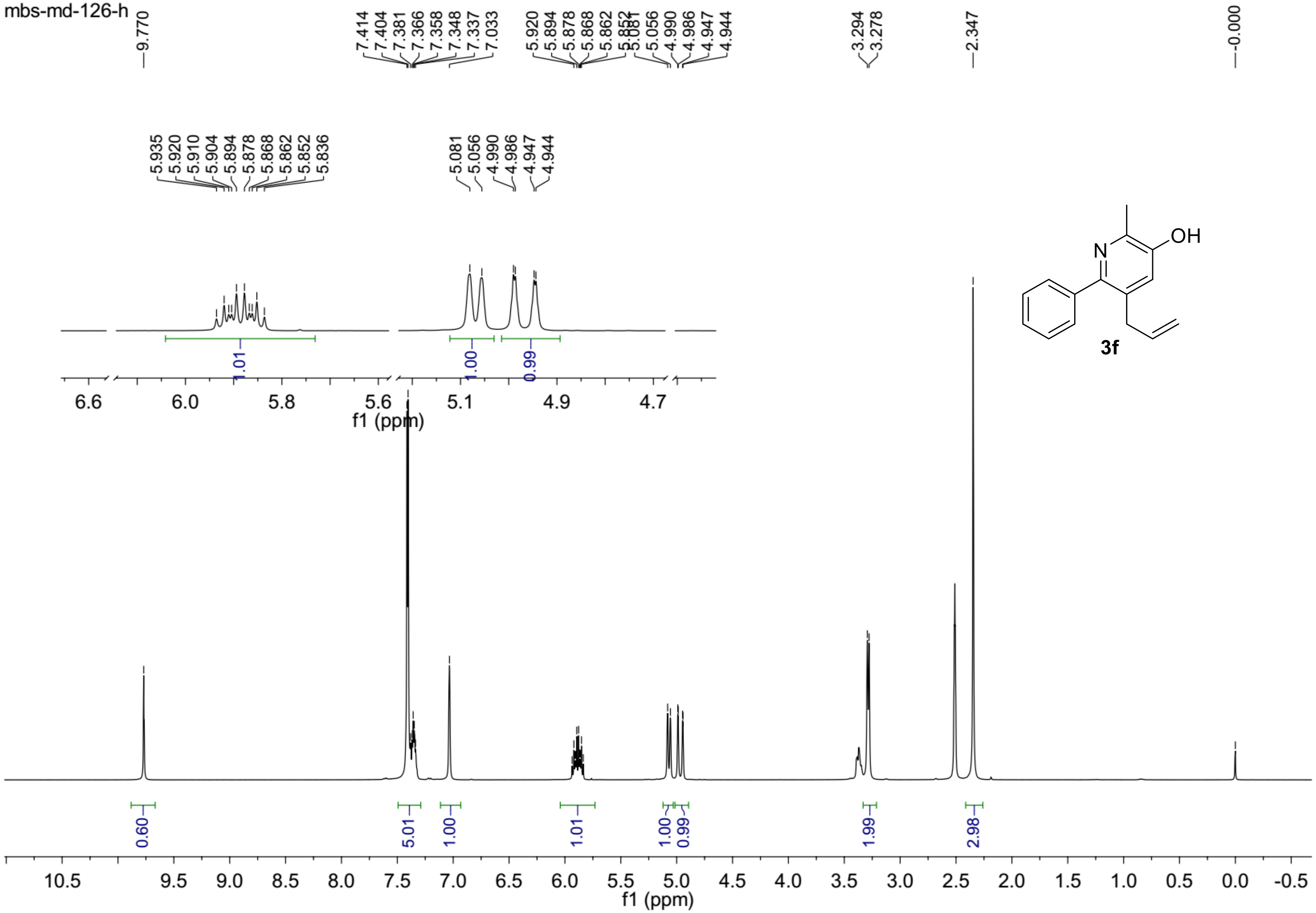

Supplementary Figure 118. ^{1}H NMR (400 MHz, $CDCl_3$) spectra for compound **3f**

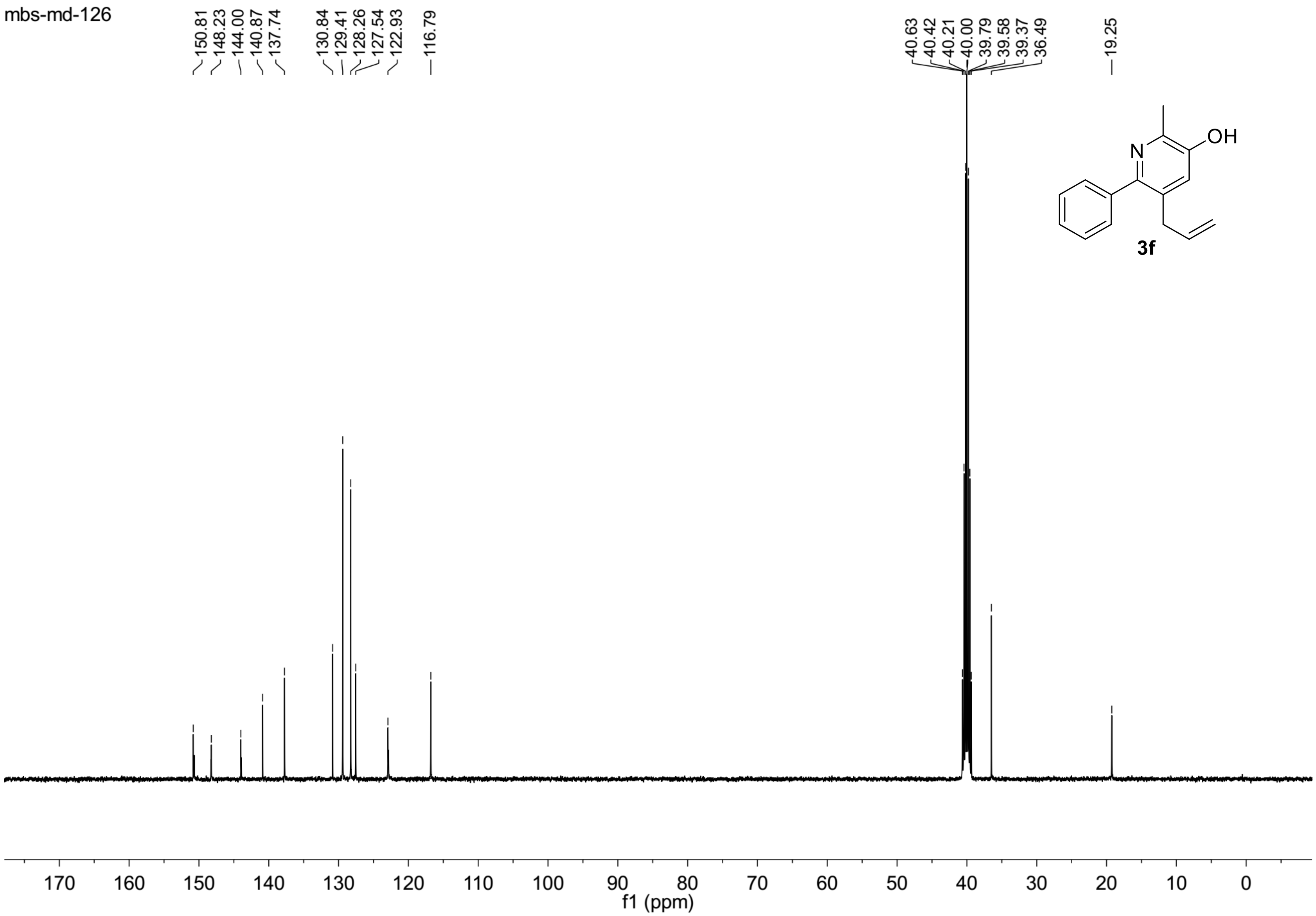

Supplementary Figure 119. ^{13}C NMR (100 MHz, $CDCl_3$) spectra for compound **3f**

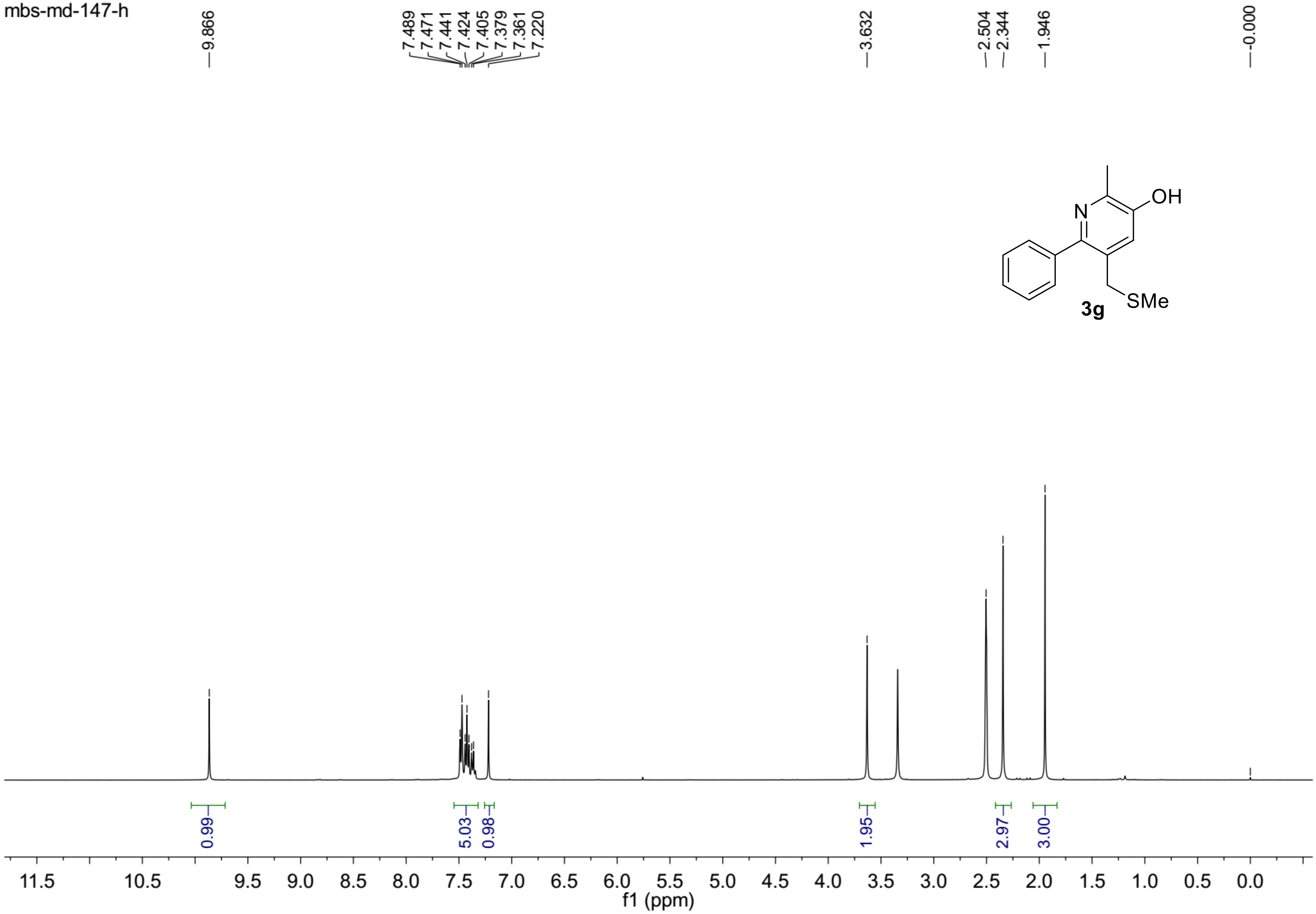

Supplementary Figure 120. ^{1}H NMR (400 MHz, $CDCl_3$) spectra for compound **3g**

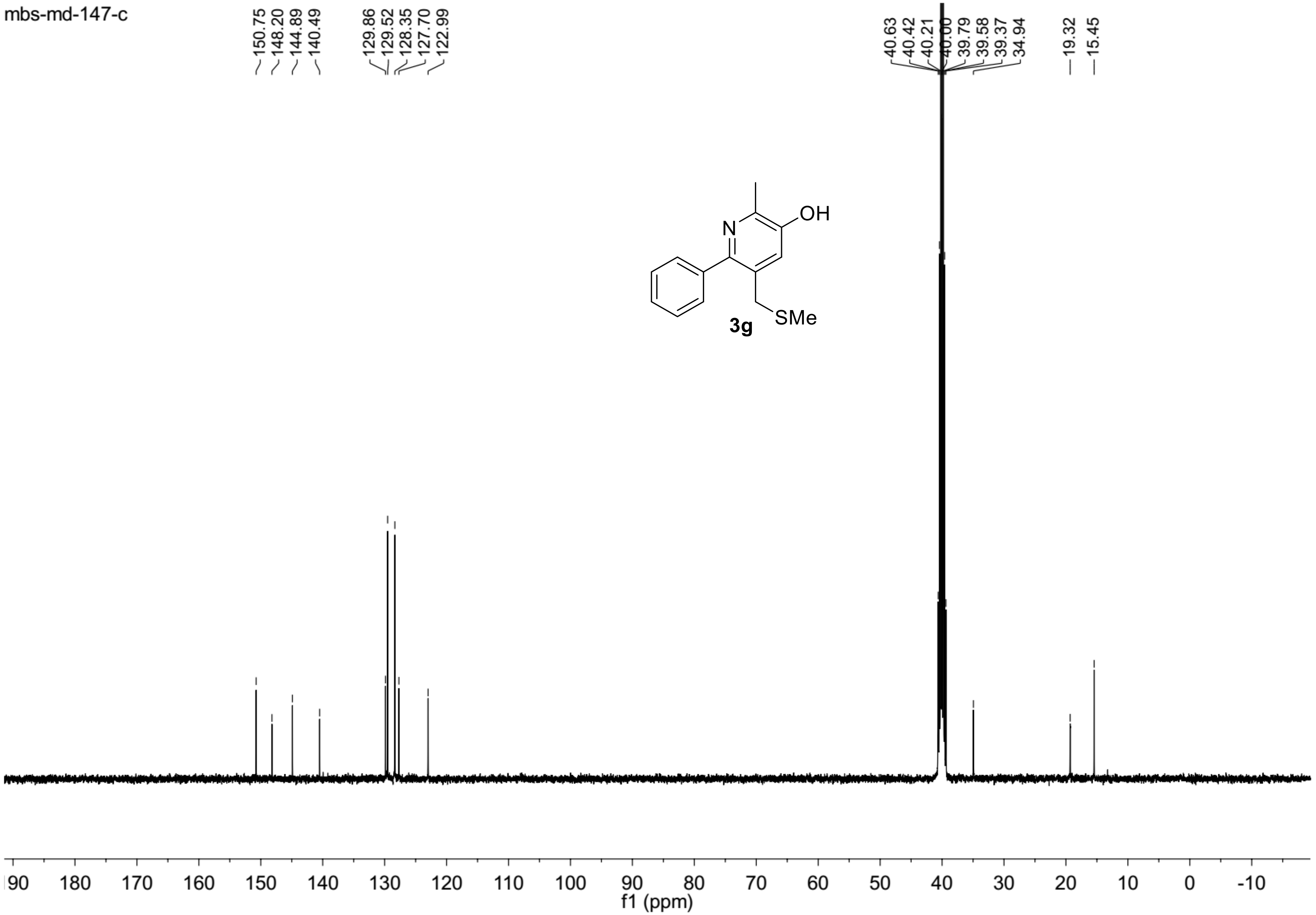

Supplementary Figure 121. ^{13}C NMR (100 MHz, $CDCl_3$) spectra for compound **3g**

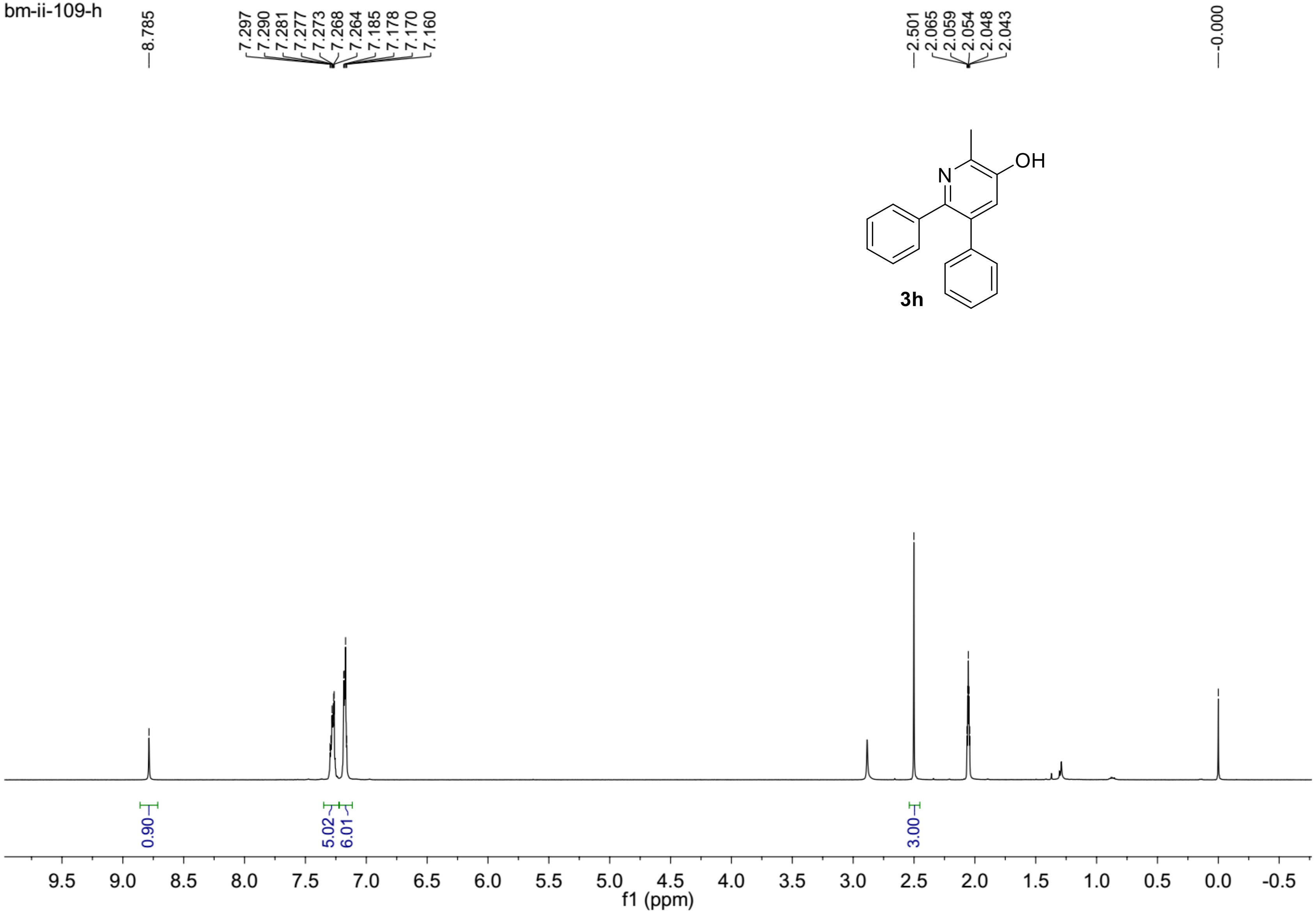

Supplementary Figure 122. ^{1}H NMR (400 MHz, $CDCl_3$) spectra for compound **3h**

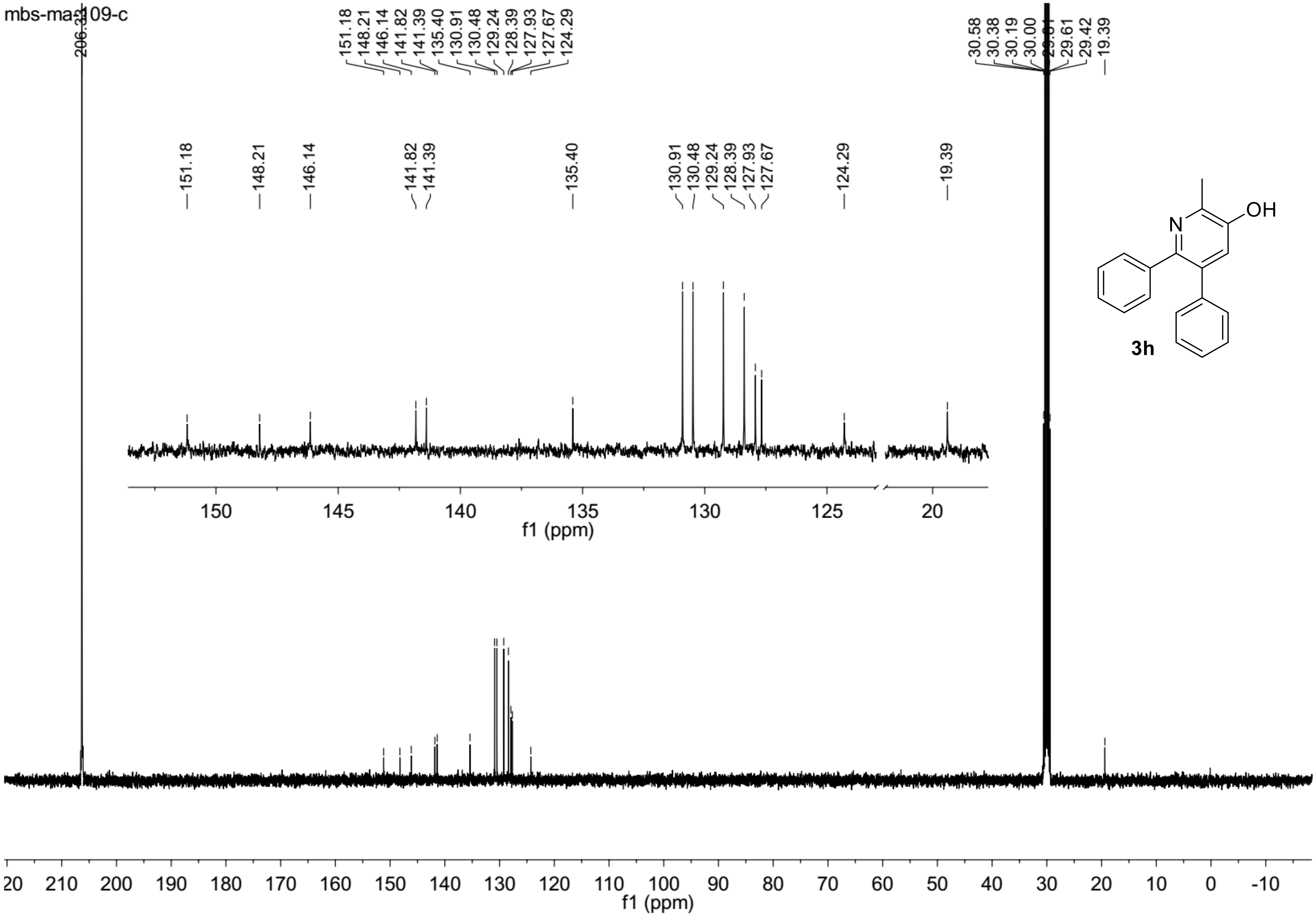

Supplementary Figure 123. ^{13}C NMR (100 MHz, $CDCl_3$) spectra for compound **3h**

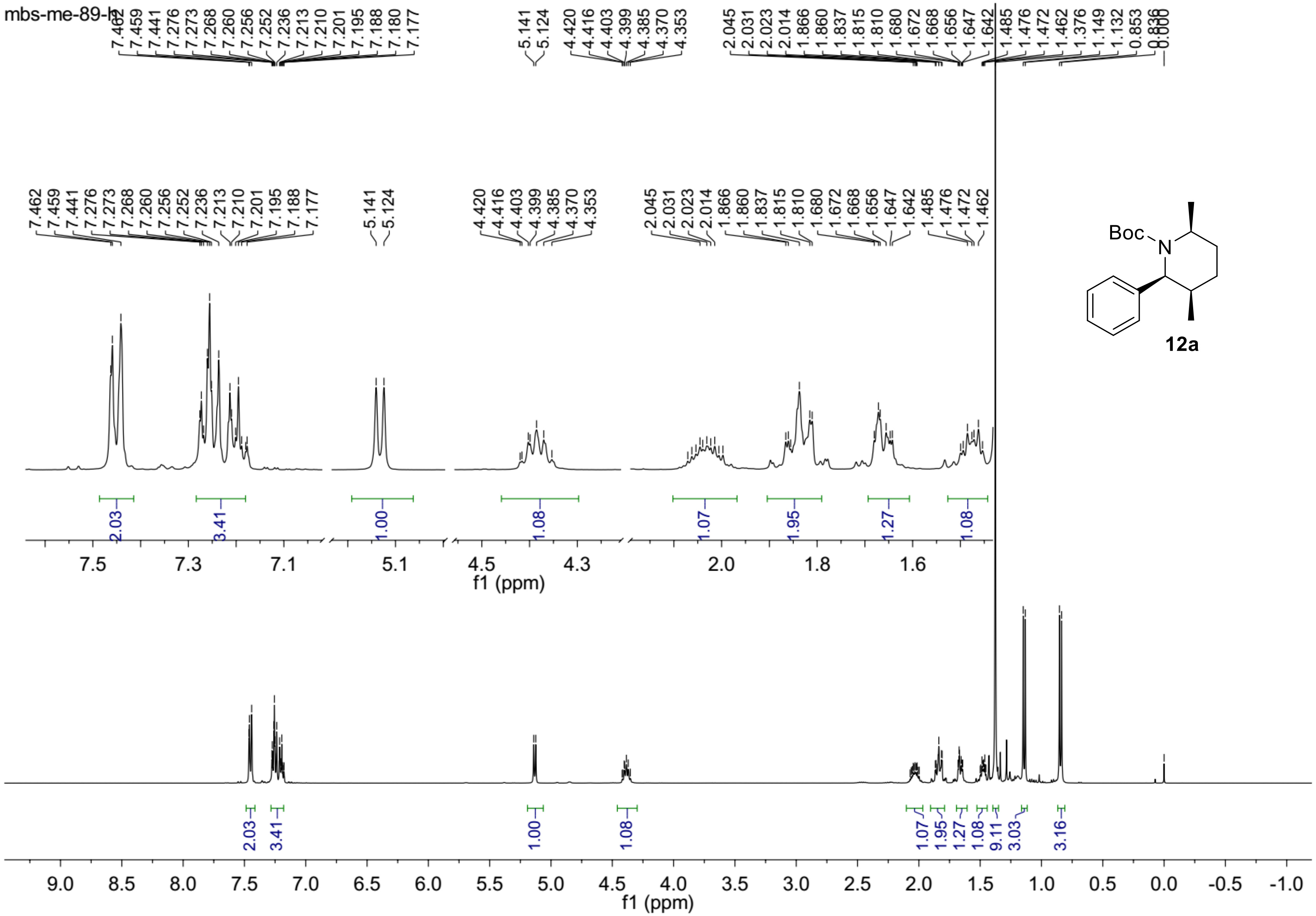

Supplementary Figure 124. ^{1}H NMR (400 MHz, $CDCl_3$) spectra for compound **12a**

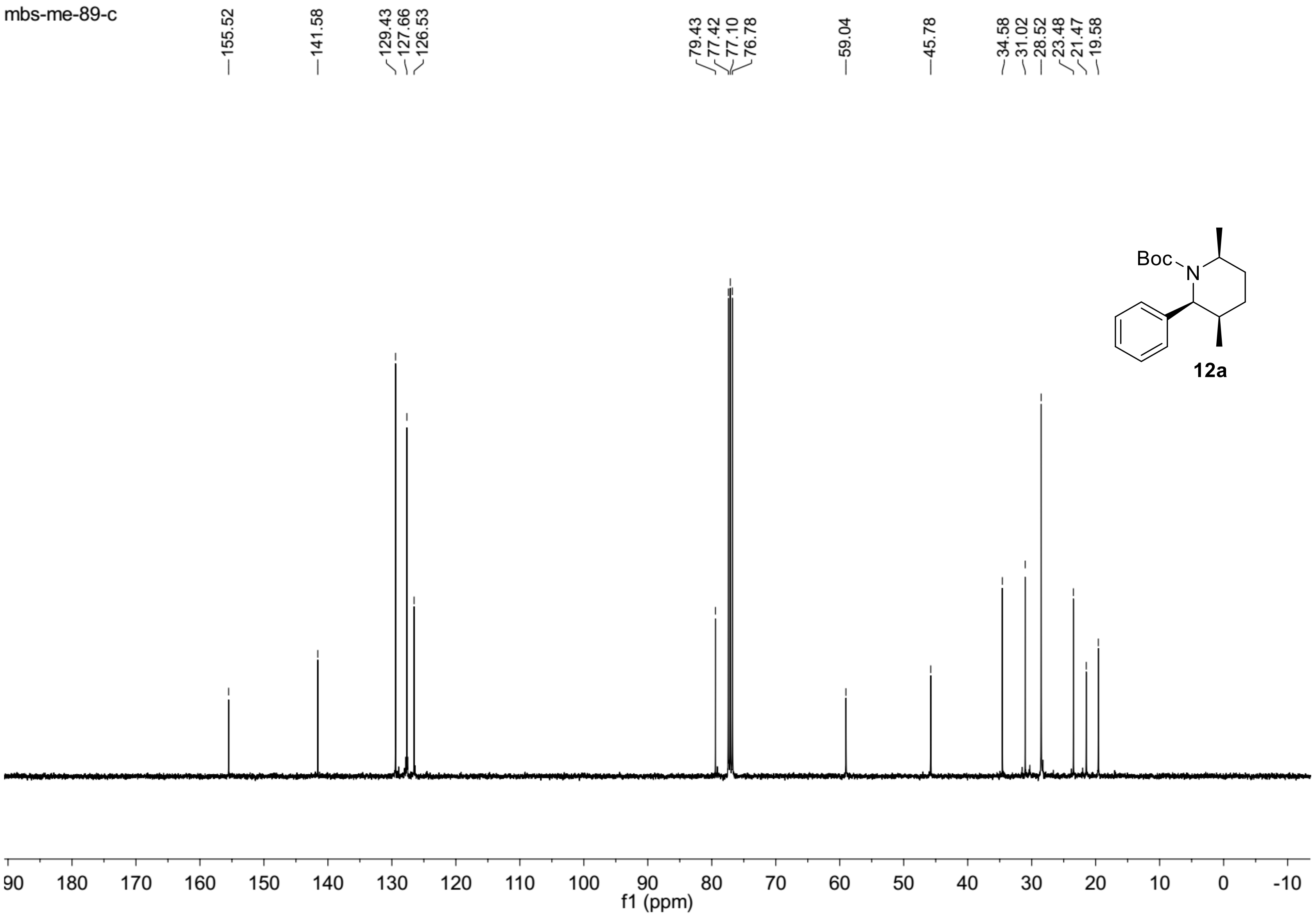

Supplementary Figure 125. ^{13}C NMR (100 MHz, $CDCl_3$) spectra for compound **12a**

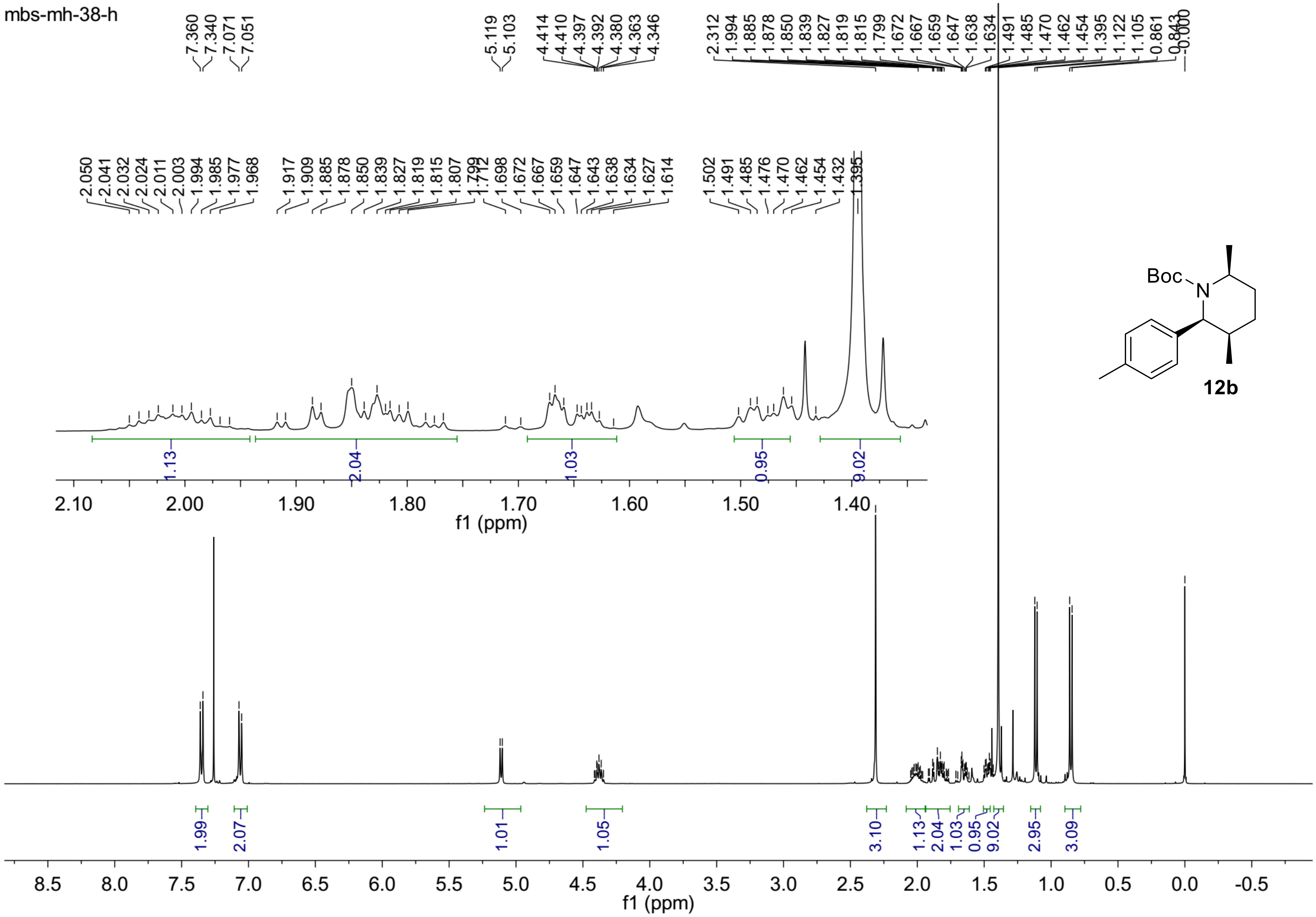

Supplementary Figure 126. ^{1}H NMR (400 MHz, $CDCl_3$) spectra for compound **12b**

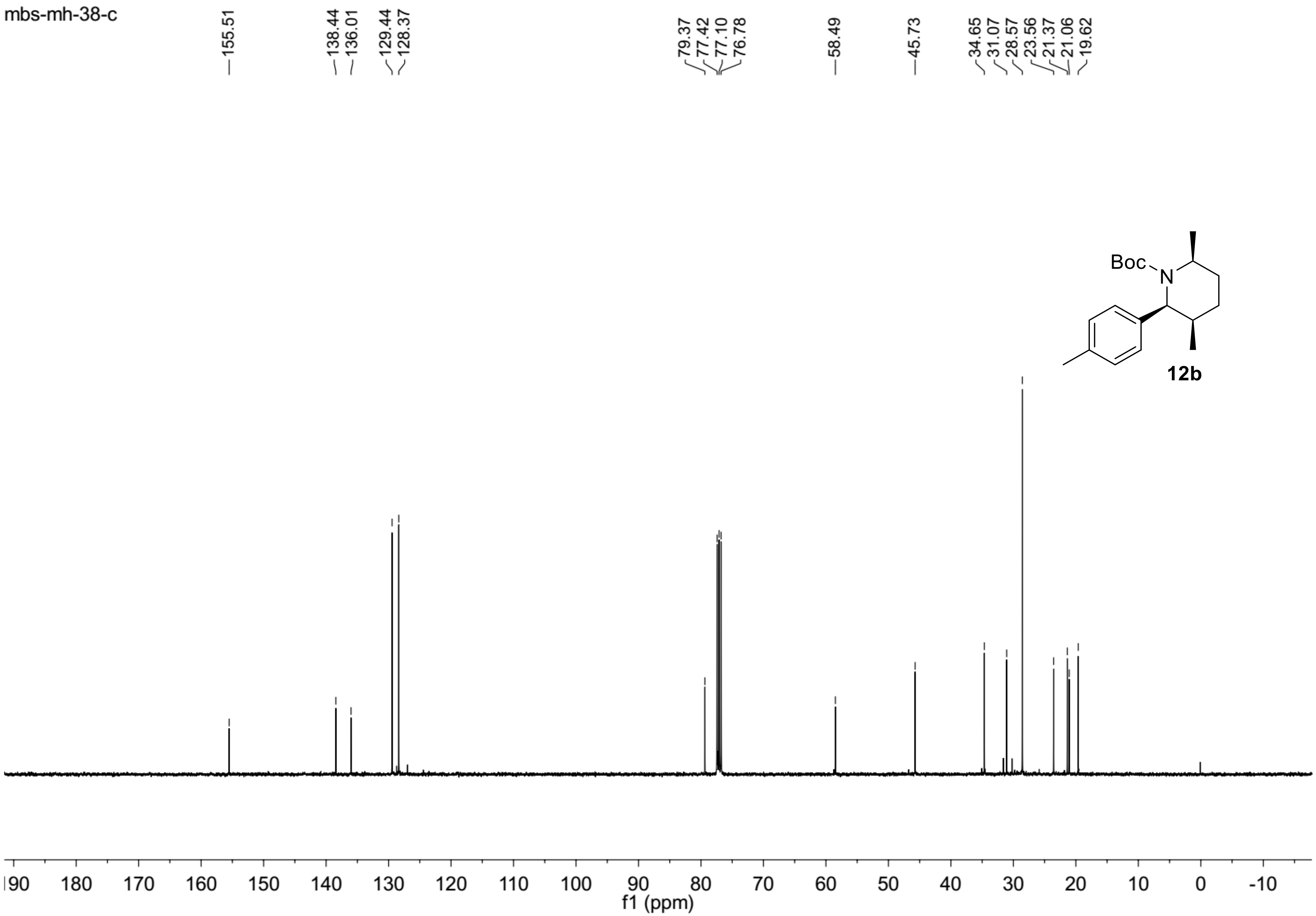

Supplementary Figure 127. ^{13}C NMR (100 MHz, $CDCl_3$) spectra for compound **12b**

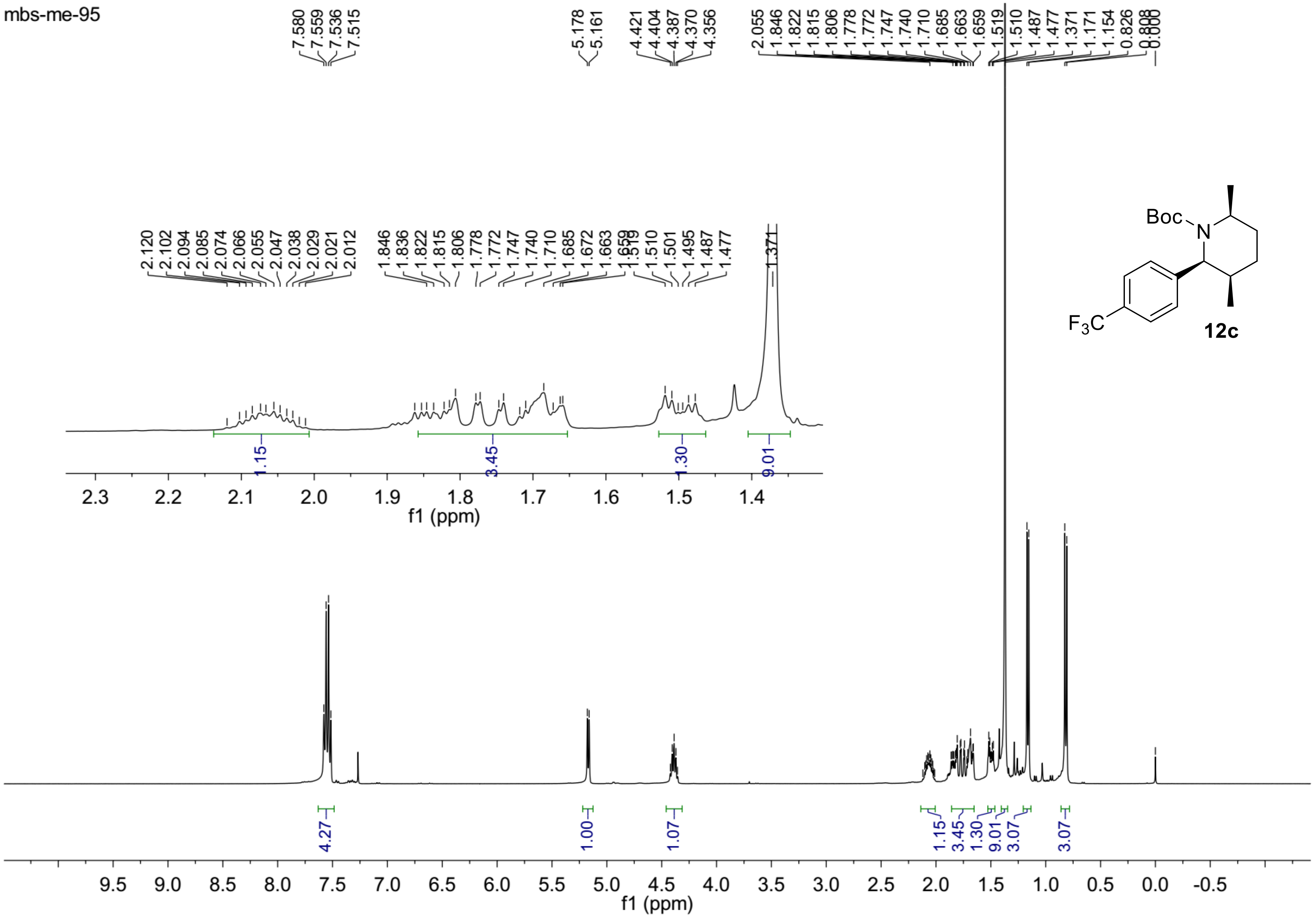

Supplementary Figure 128. ^{1}H NMR (400 MHz, $CDCl_3$) spectra for compound **12c**

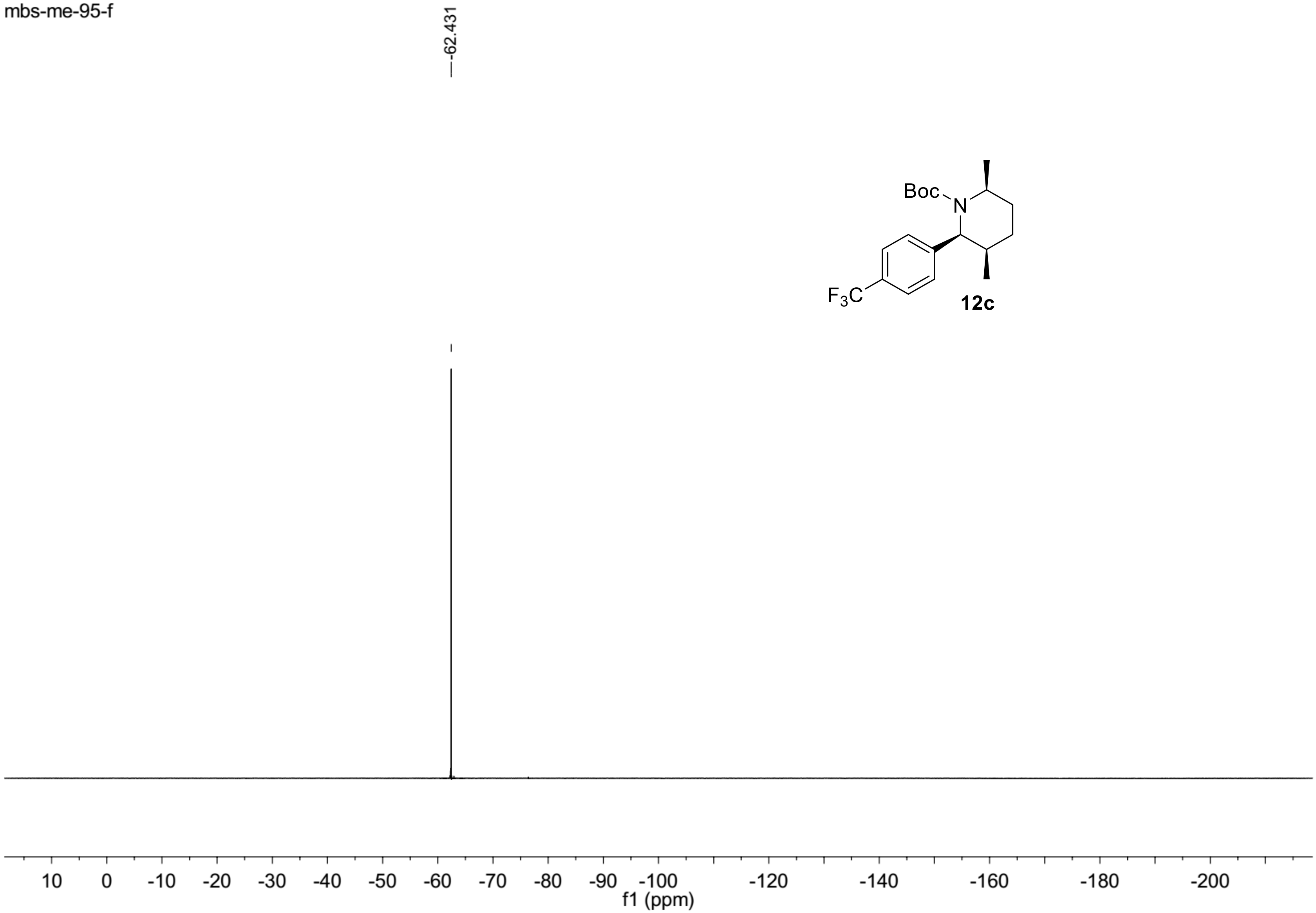

Supplementary Figure 129. ^{19}F NMR (376 MHz, $CDCl_3$) spectra for compound **12c**

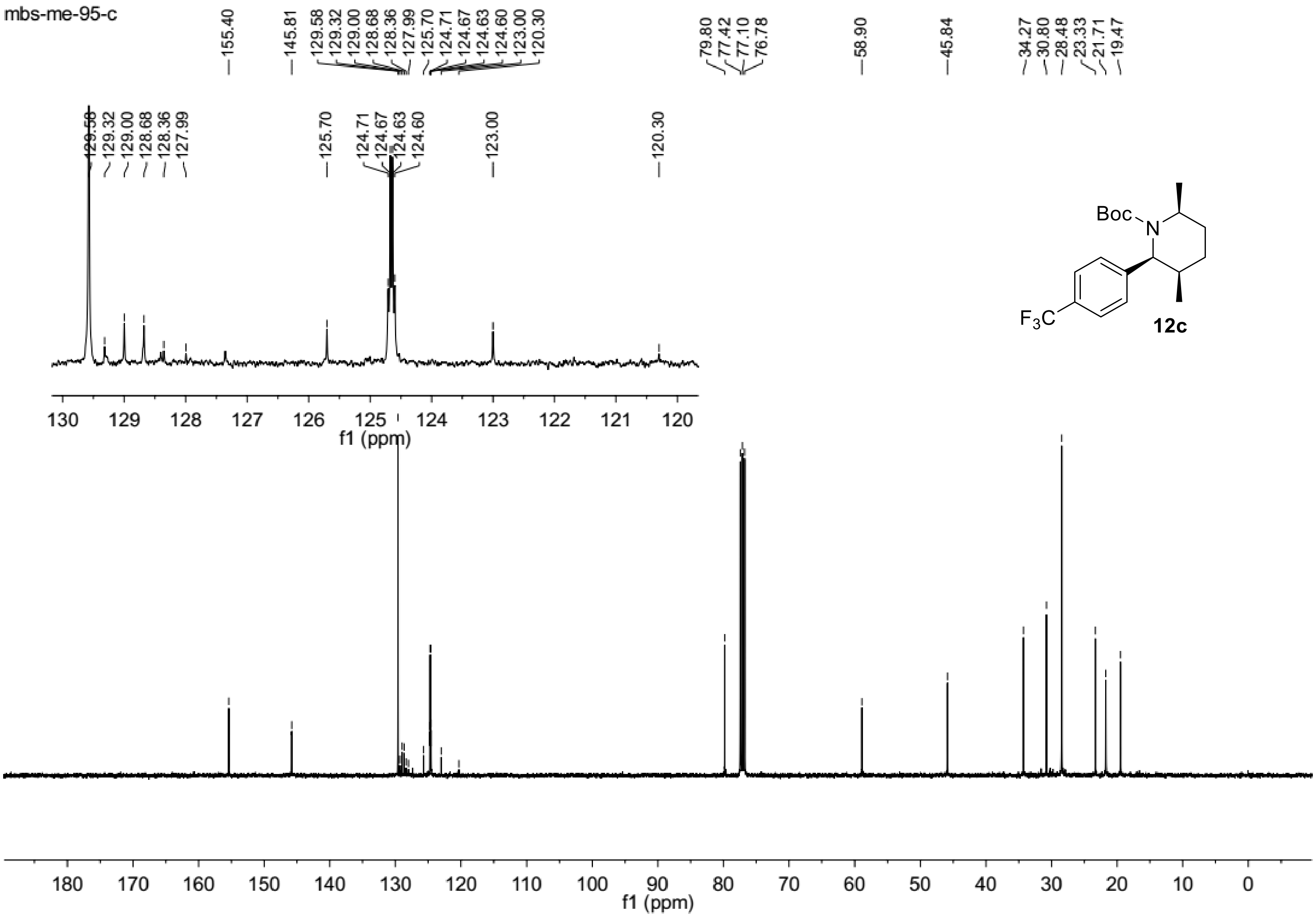

Supplementary Figure 130. ^{13}C NMR (100 MHz, $CDCl_3$) spectra for compound **12c**

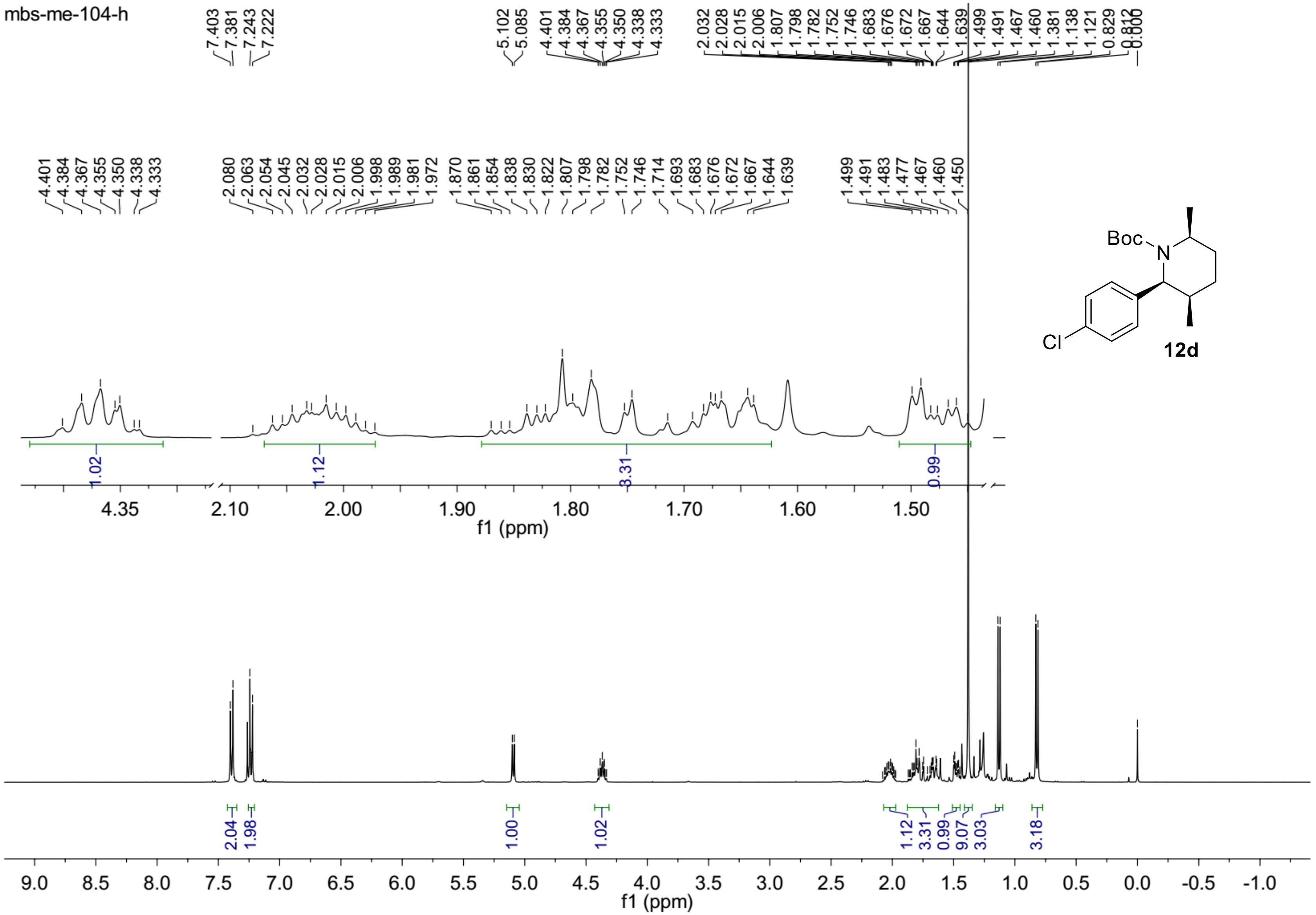

Supplementary Figure 131. ^{1}H NMR (400 MHz, $CDCl_3$) spectra for compound **12d**

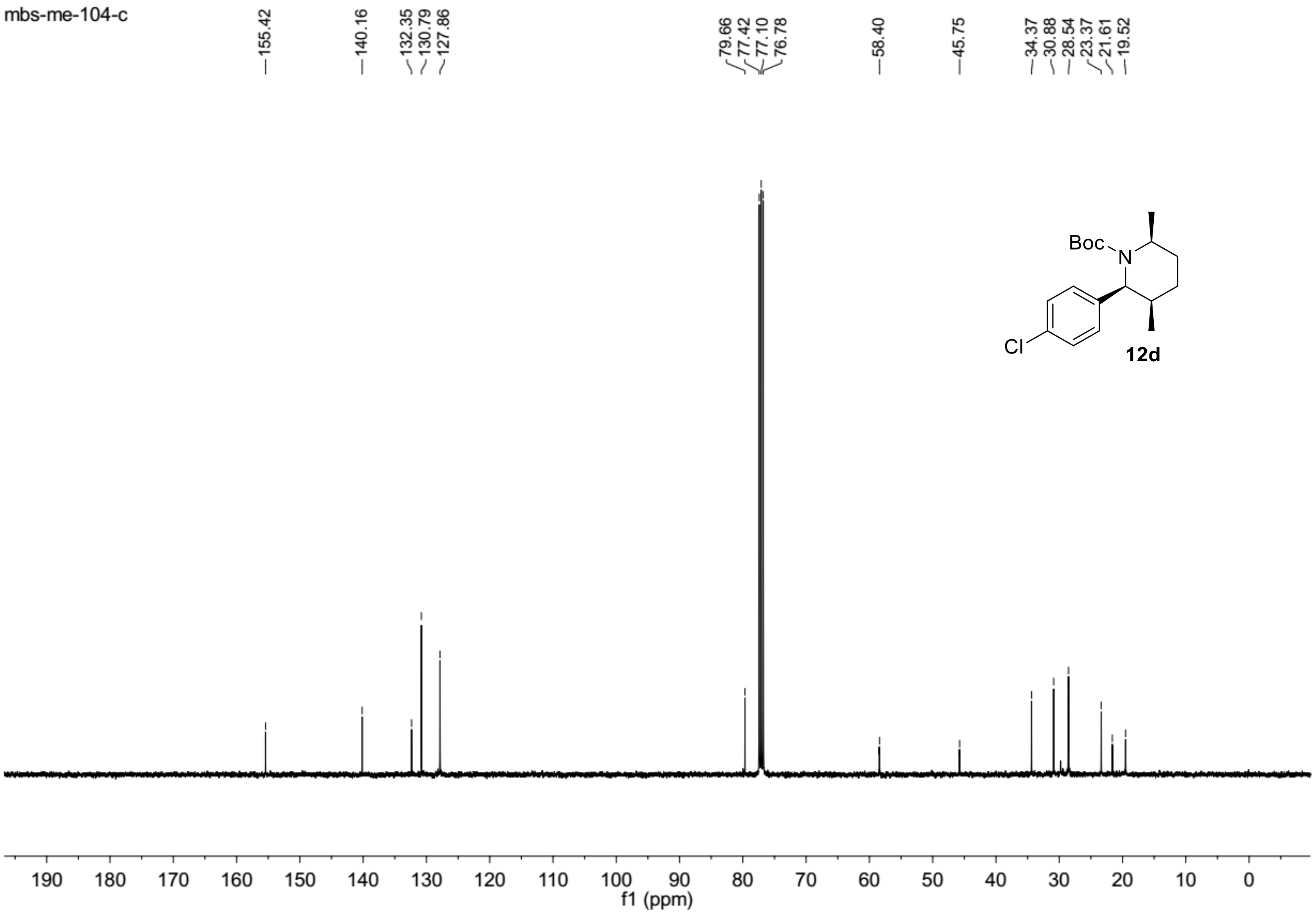

Supplementary Figure 132. ^{13}C NMR (100 MHz, $CDCl_3$) spectra for compound **12d**

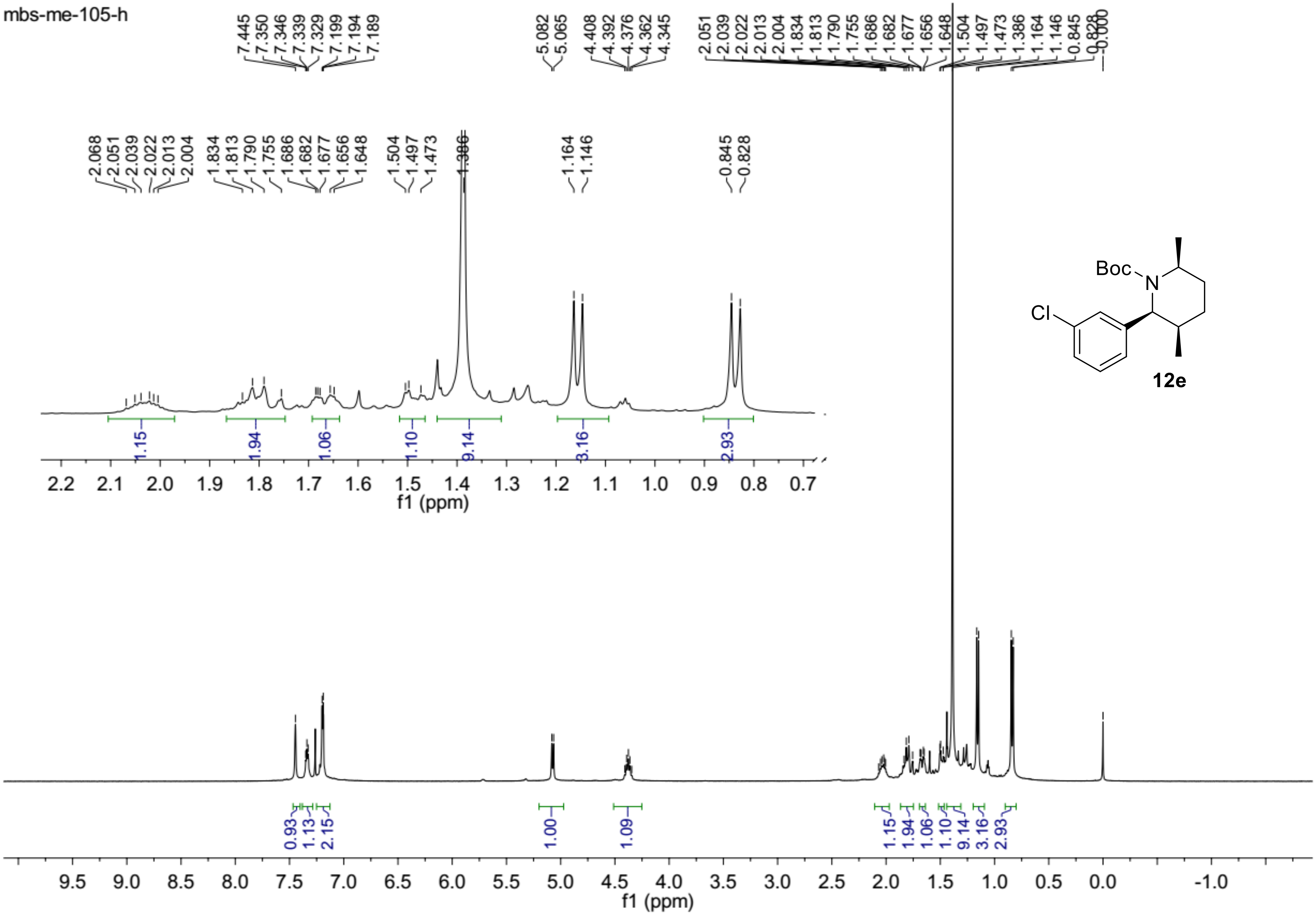

Supplementary Figure 133. ^{1}H NMR (400 MHz, $CDCl_3$) spectra for compound **12e**

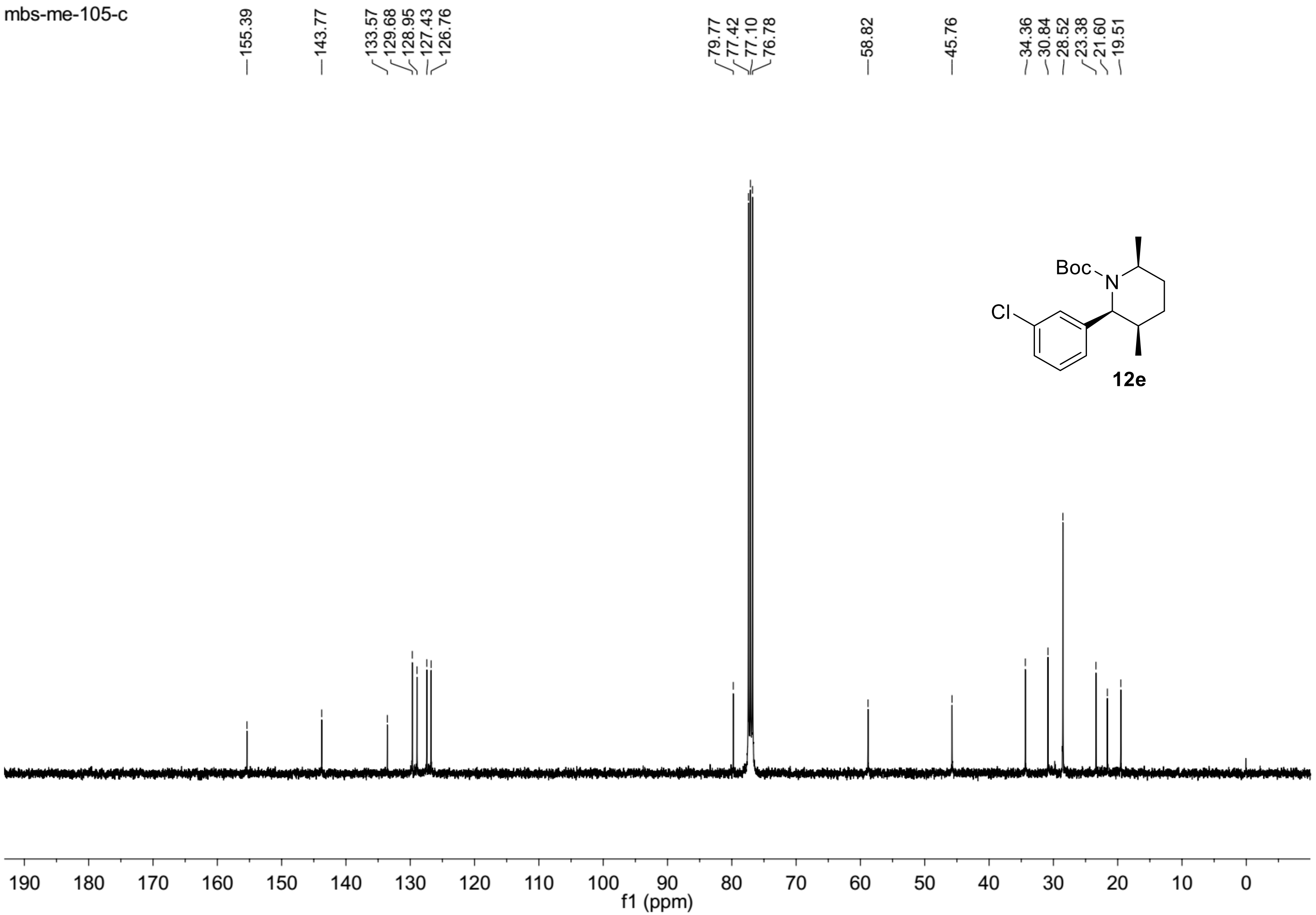

Supplementary Figure 134. ^{13}C NMR (100 MHz, $CDCl_3$) spectra for compound **12e**

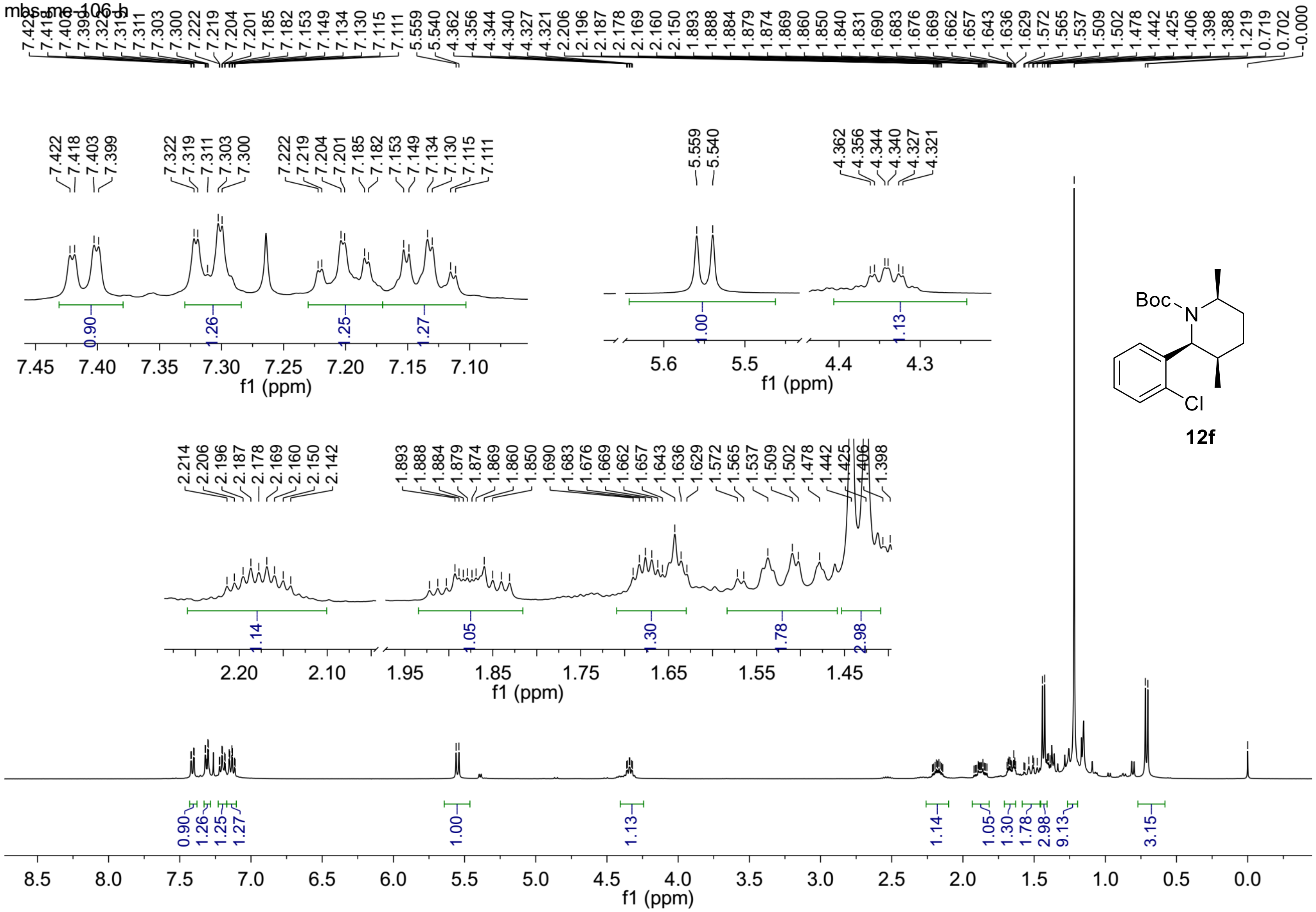

Supplementary Figure 135. ^{1}H NMR (400 MHz, $CDCl_3$) spectra for compound **12f**

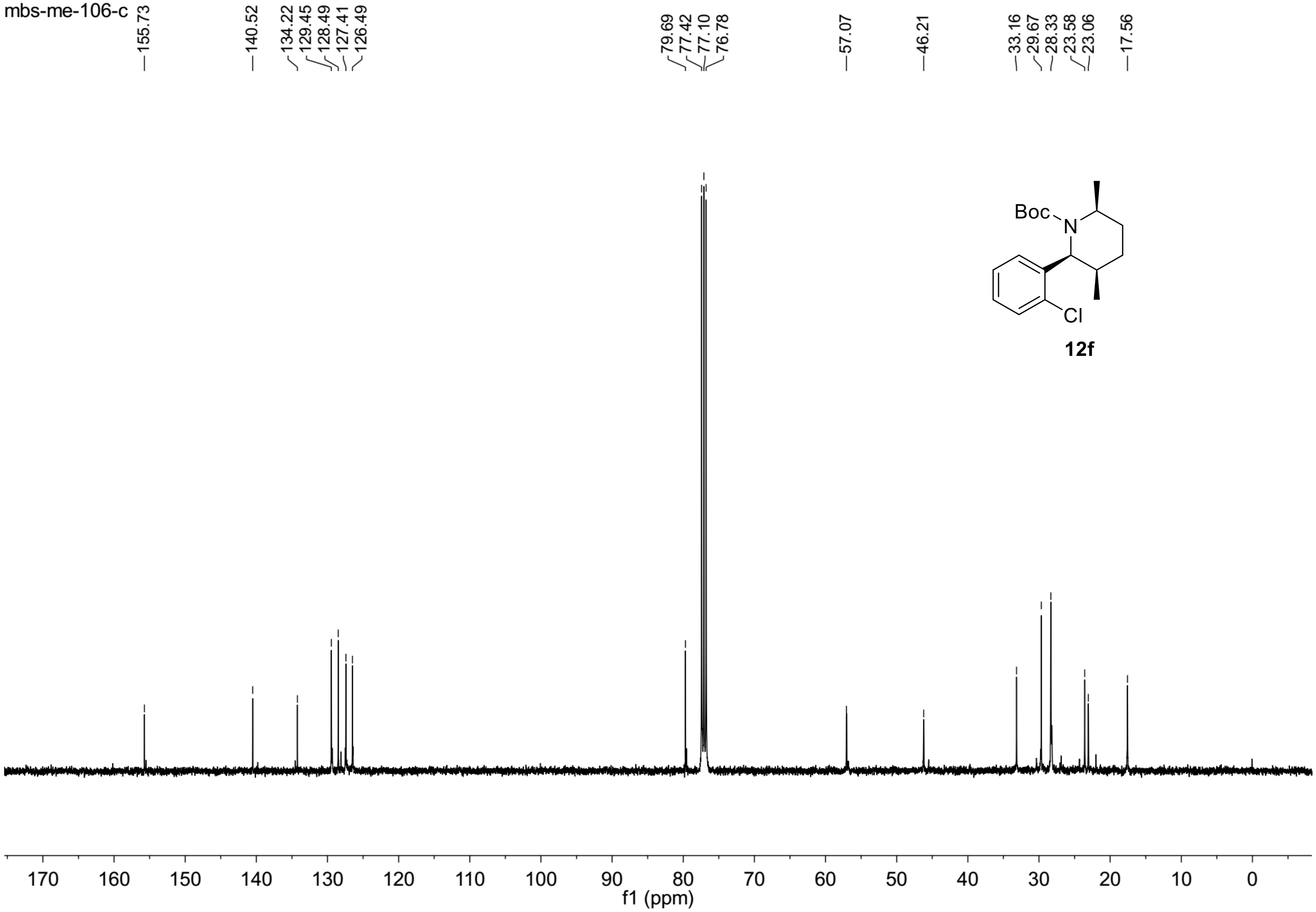

Supplementary Figure 136. ^{13}C NMR (100 MHz, $CDCl_3$) spectra for compound **12f**

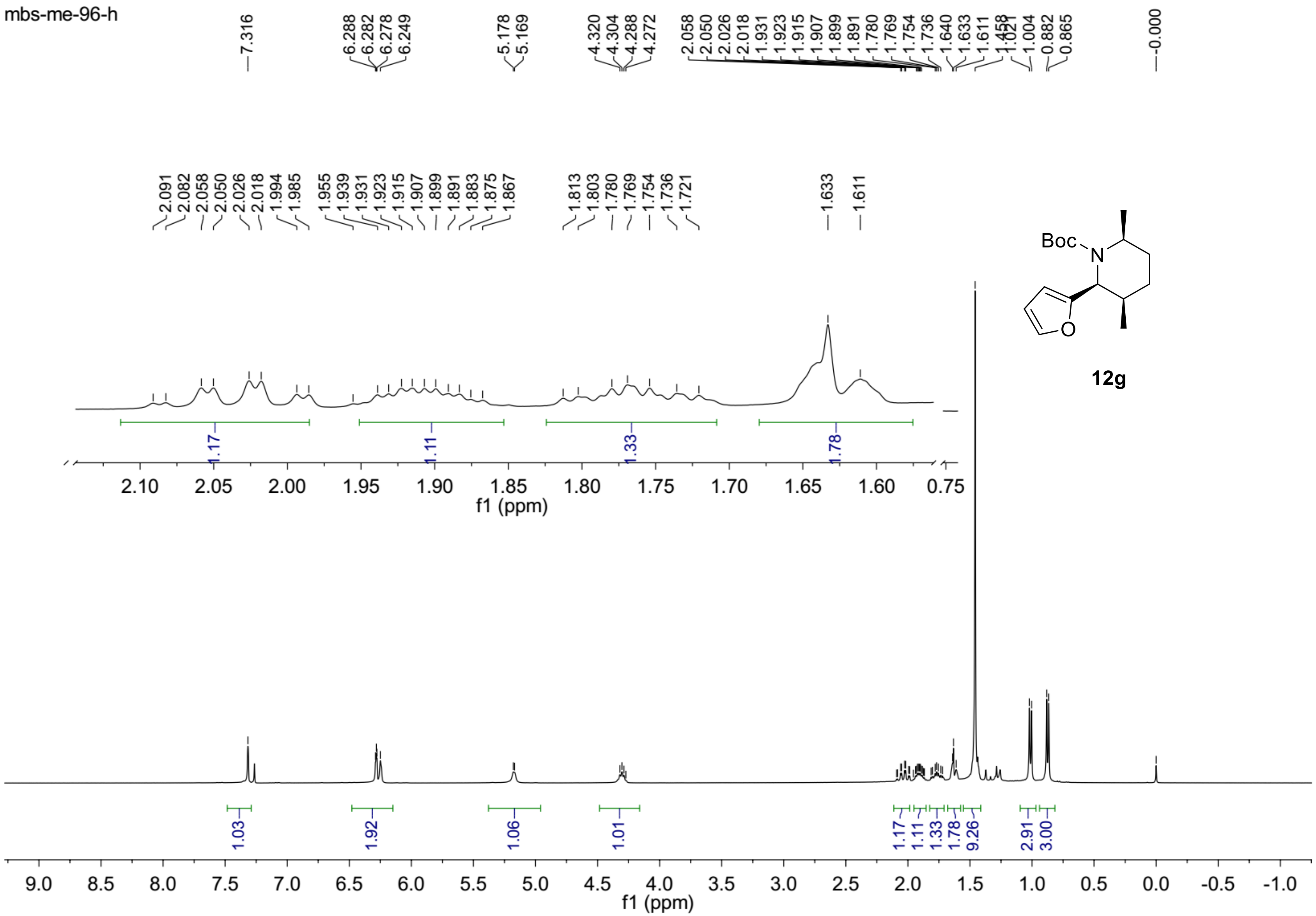

Supplementary Figure 137. ^{1}H NMR (400 MHz, $CDCl_3$) spectra for compound **12g**

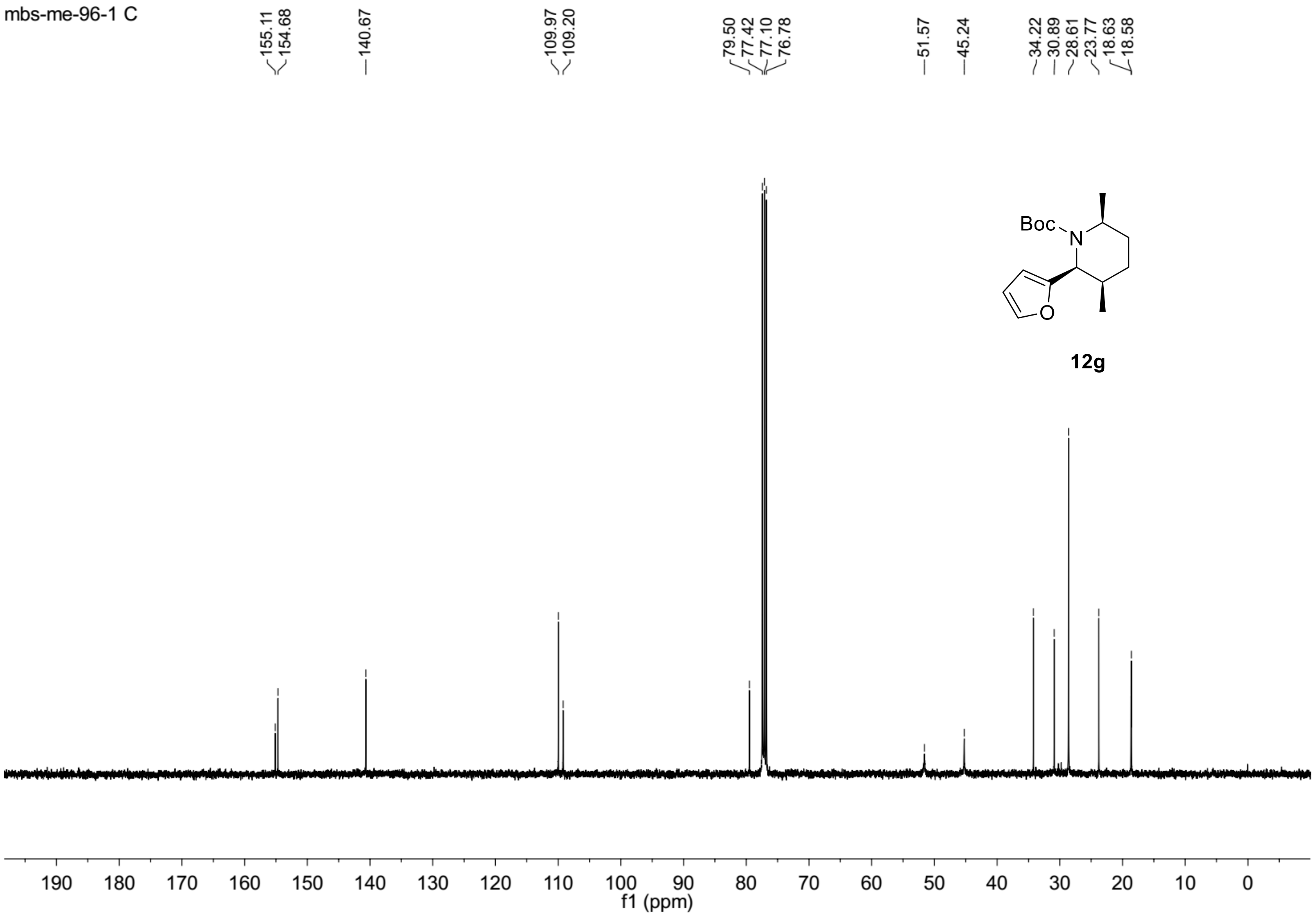

12g

Supplementary Figure 138. ^{13}C NMR (100 MHz, $CDCl_3$) spectra for compound **12g**

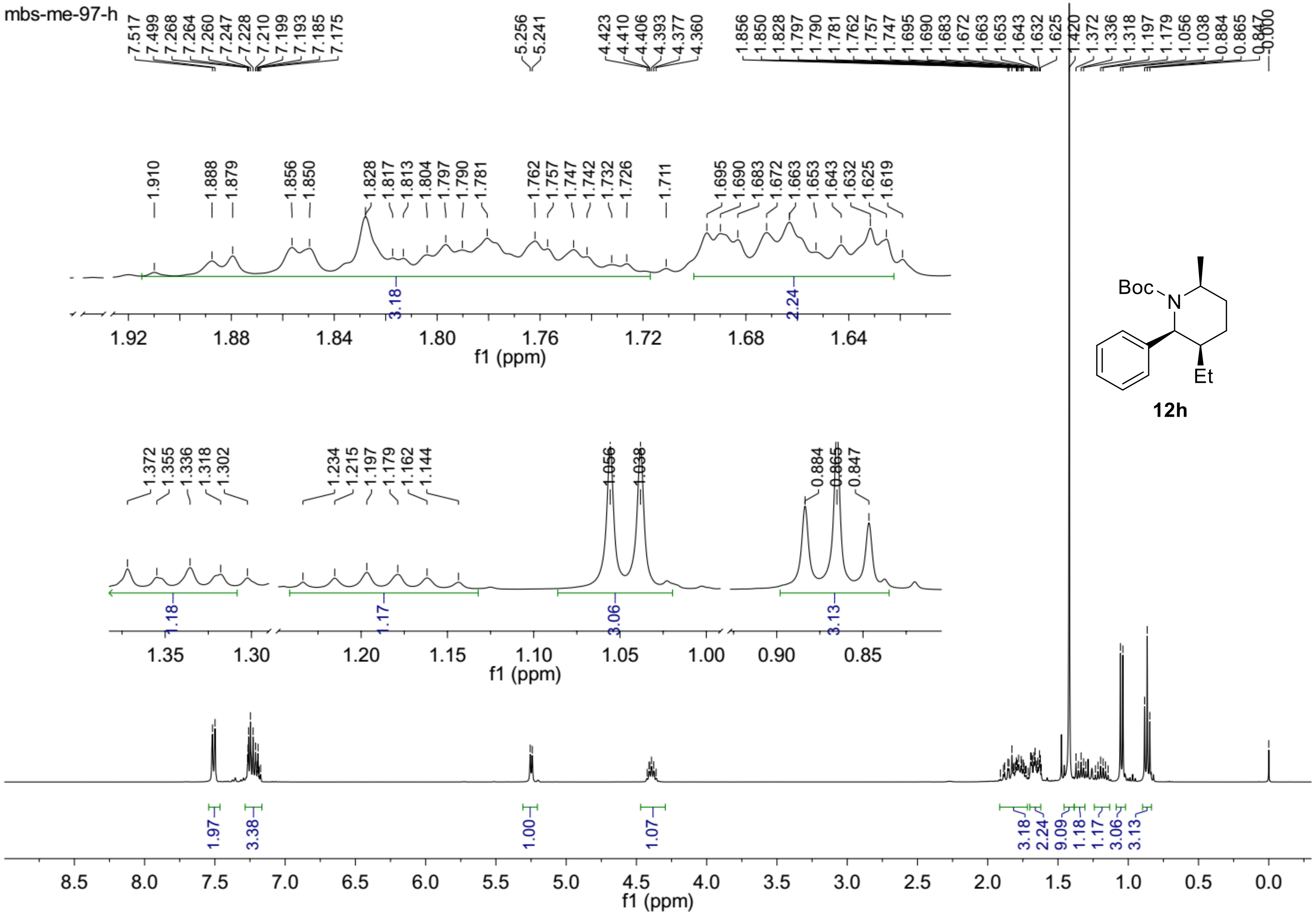

Supplementary Figure 139. ^{1}H NMR (400 MHz, $CDCl_3$) spectra for compound **12h**

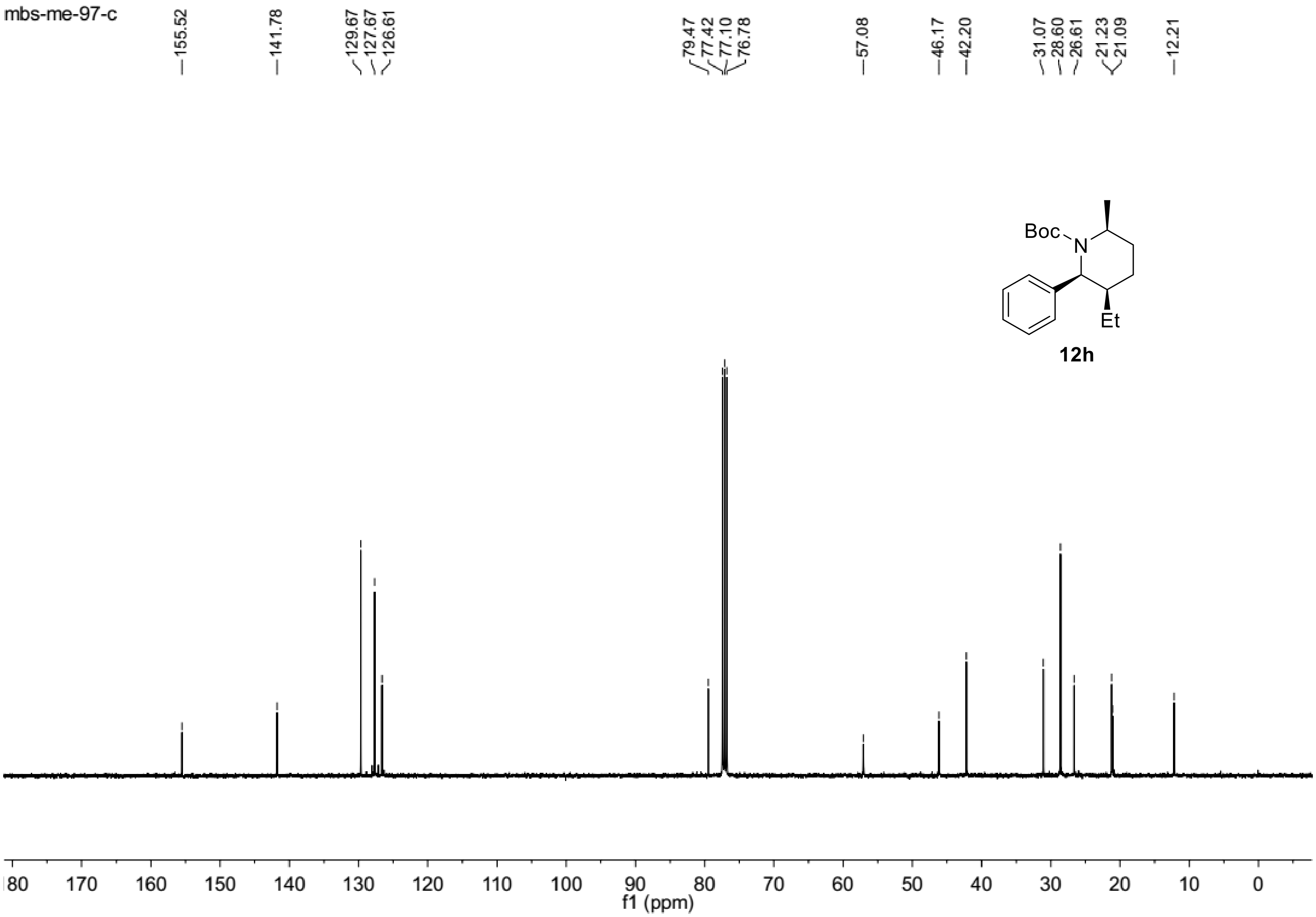

Supplementary Figure 140. ^{13}C NMR (100 MHz, $CDCl_3$) spectra for compound **12h**

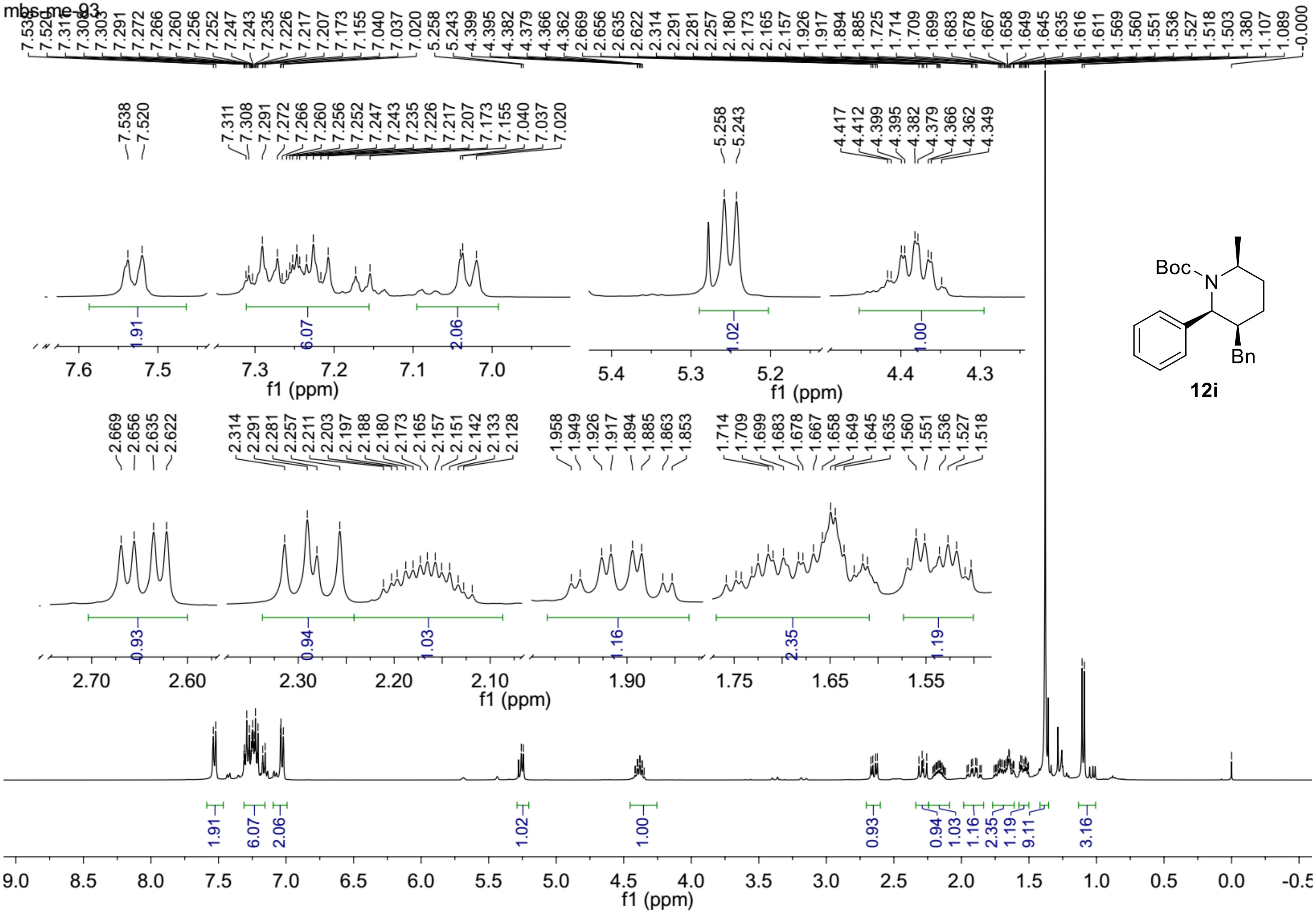

Supplementary Figure 141. ^{1}H NMR (400 MHz, $CDCl_3$) spectra for compound **12i**

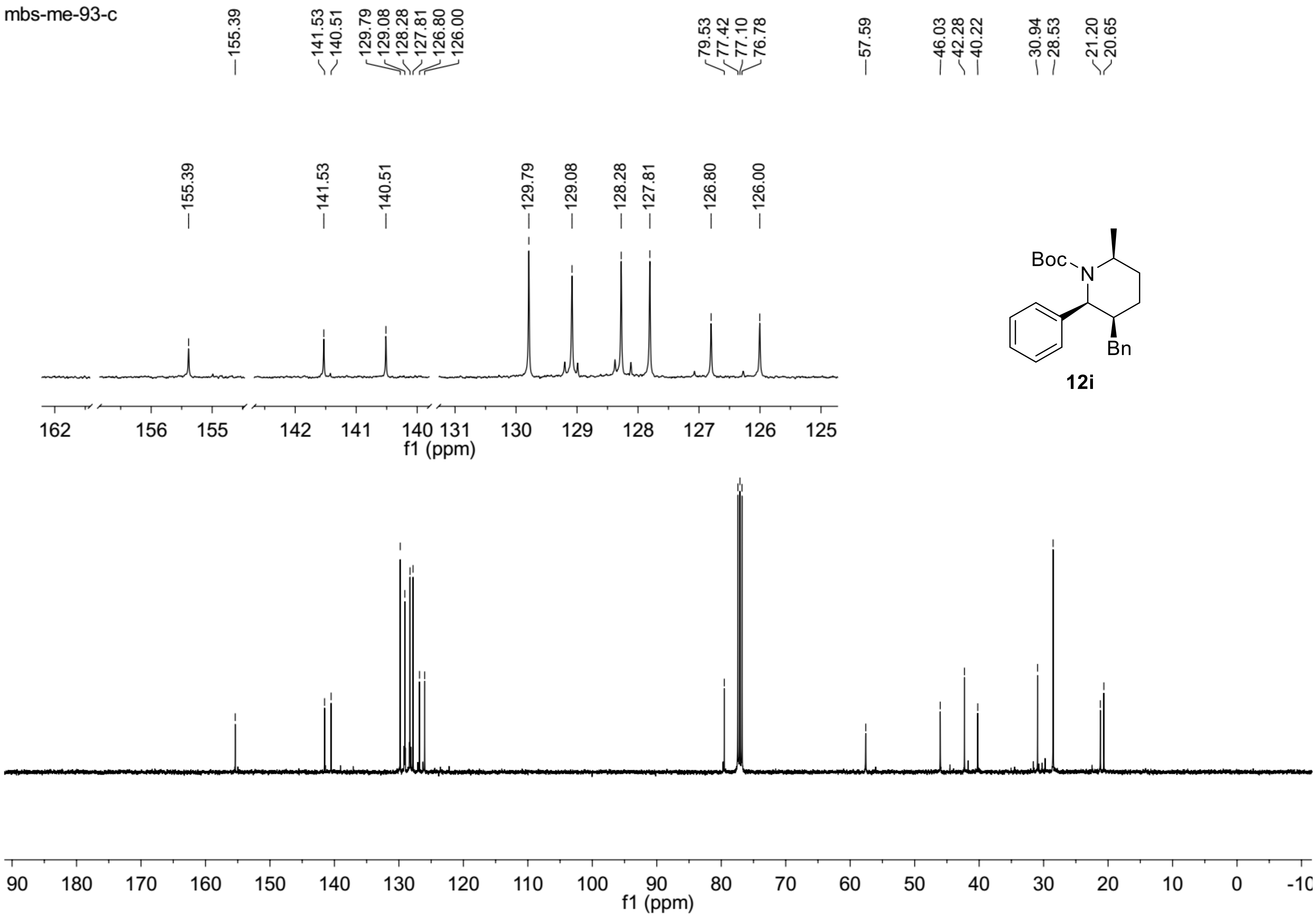

Supplementary Figure 142. ^{13}C NMR (100 MHz, $CDCl_3$) spectra for compound **12i**

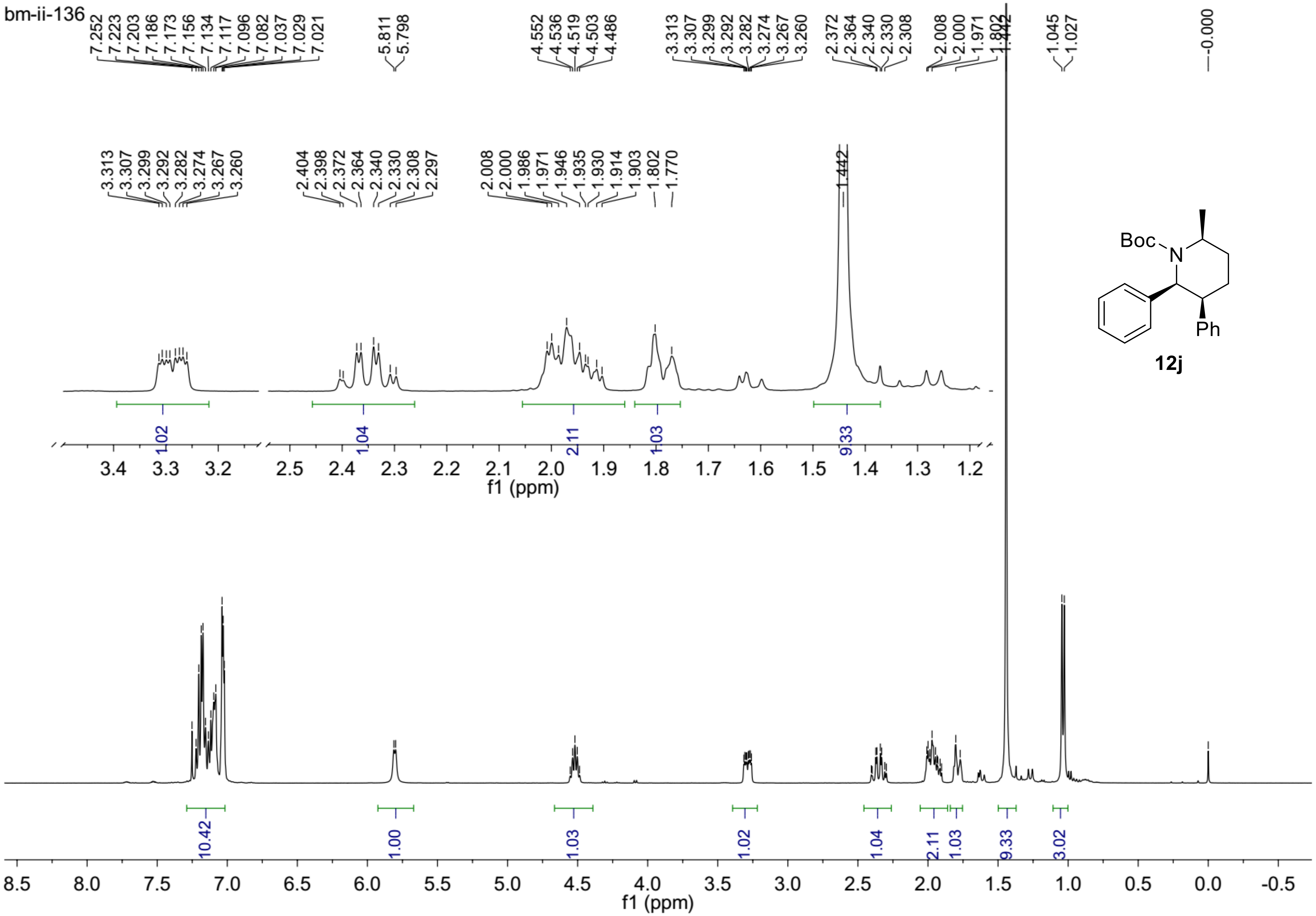

Supplementary Figure 143. ^{1}H NMR (400 MHz, $CDCl_3$) spectra for compound **12j**

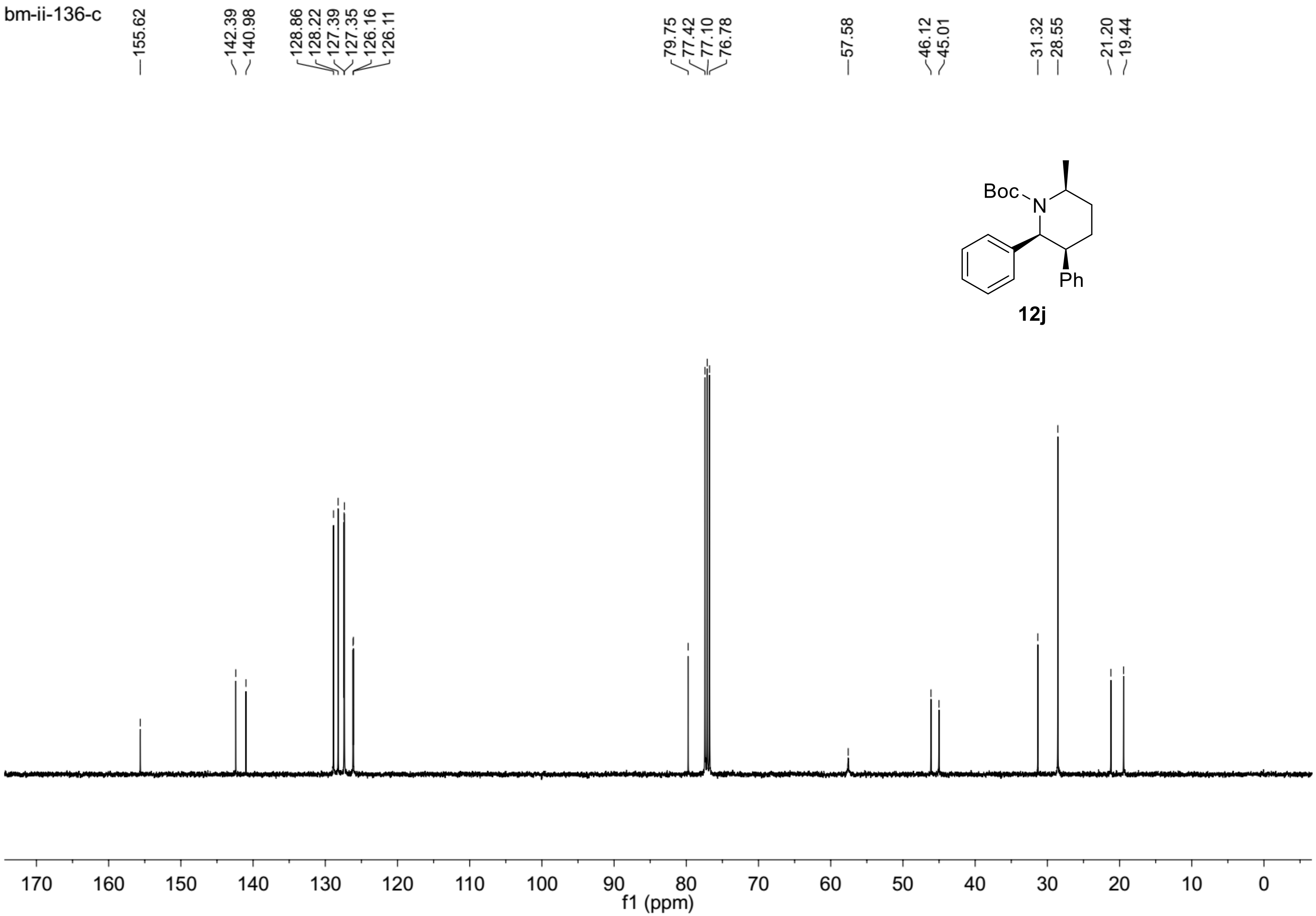

Supplementary Figure 144. ^{13}C NMR (100 MHz, $CDCl_3$) spectra for compound **12j**

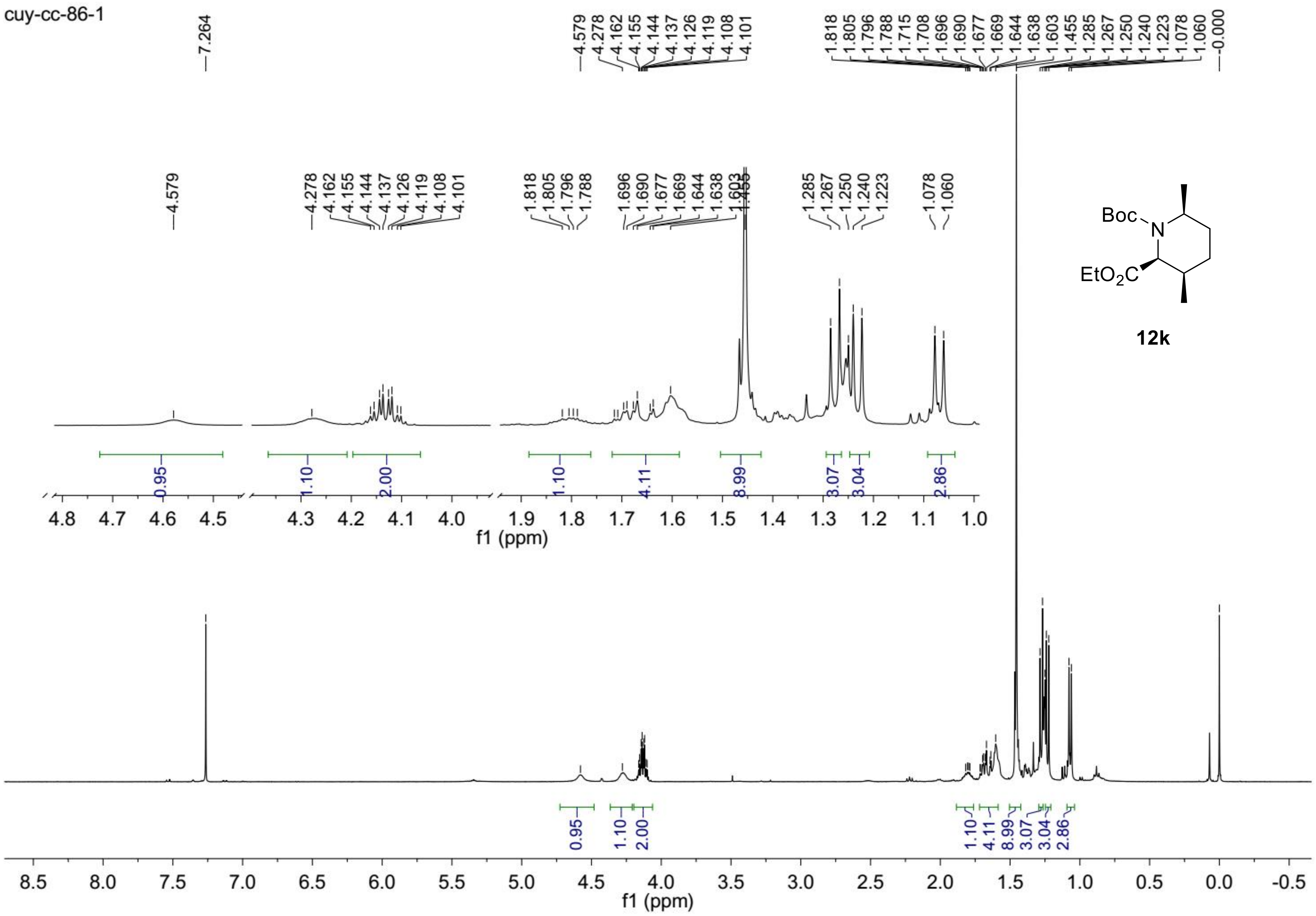

Supplementary Figure 145. ^{1}H NMR (400 MHz, $CDCl_3$) spectra for compound **12k**

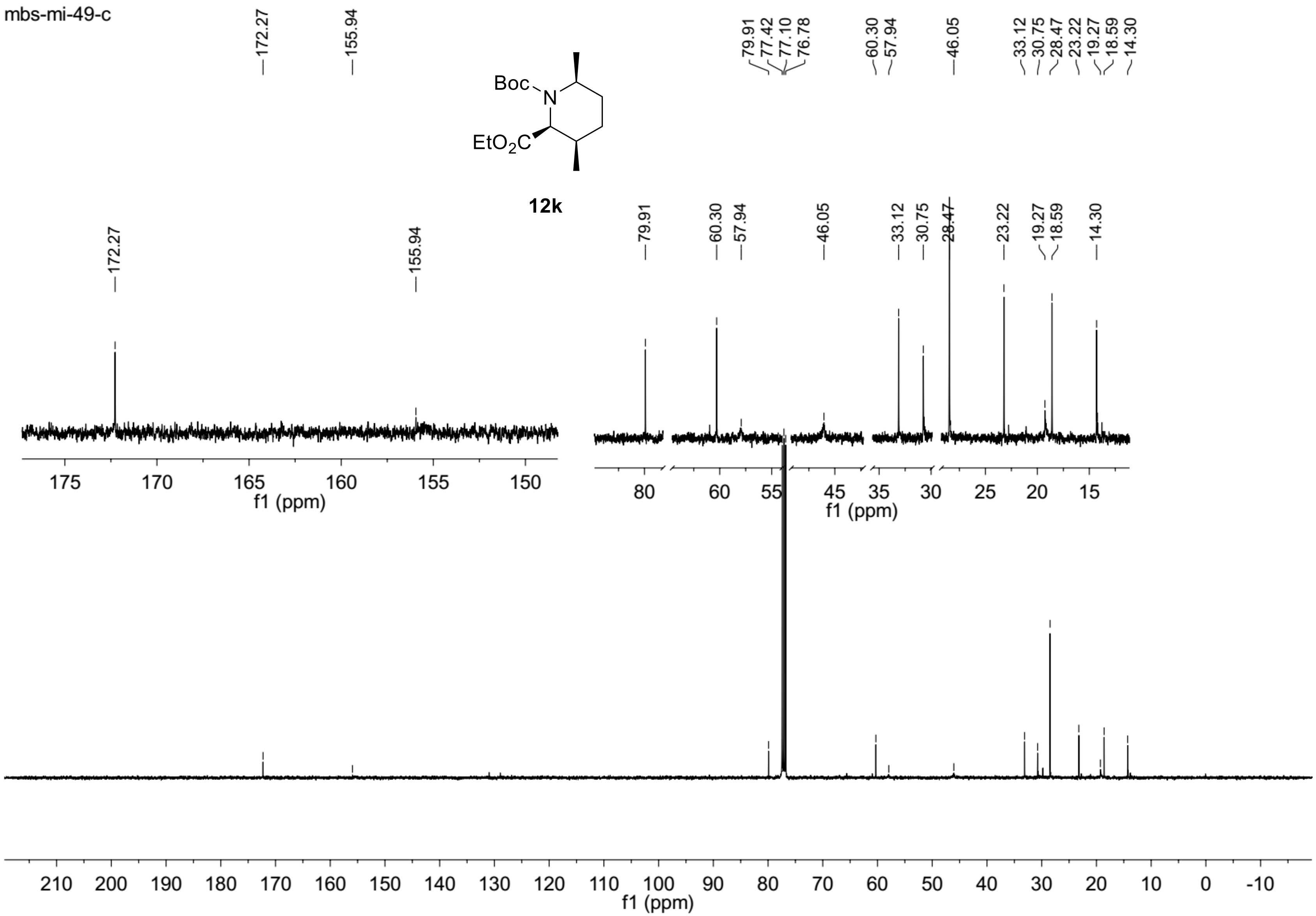

Supplementary Figure 146. ^{13}C NMR (125 MHz, $CDCl_3$) spectra for compound **12k**

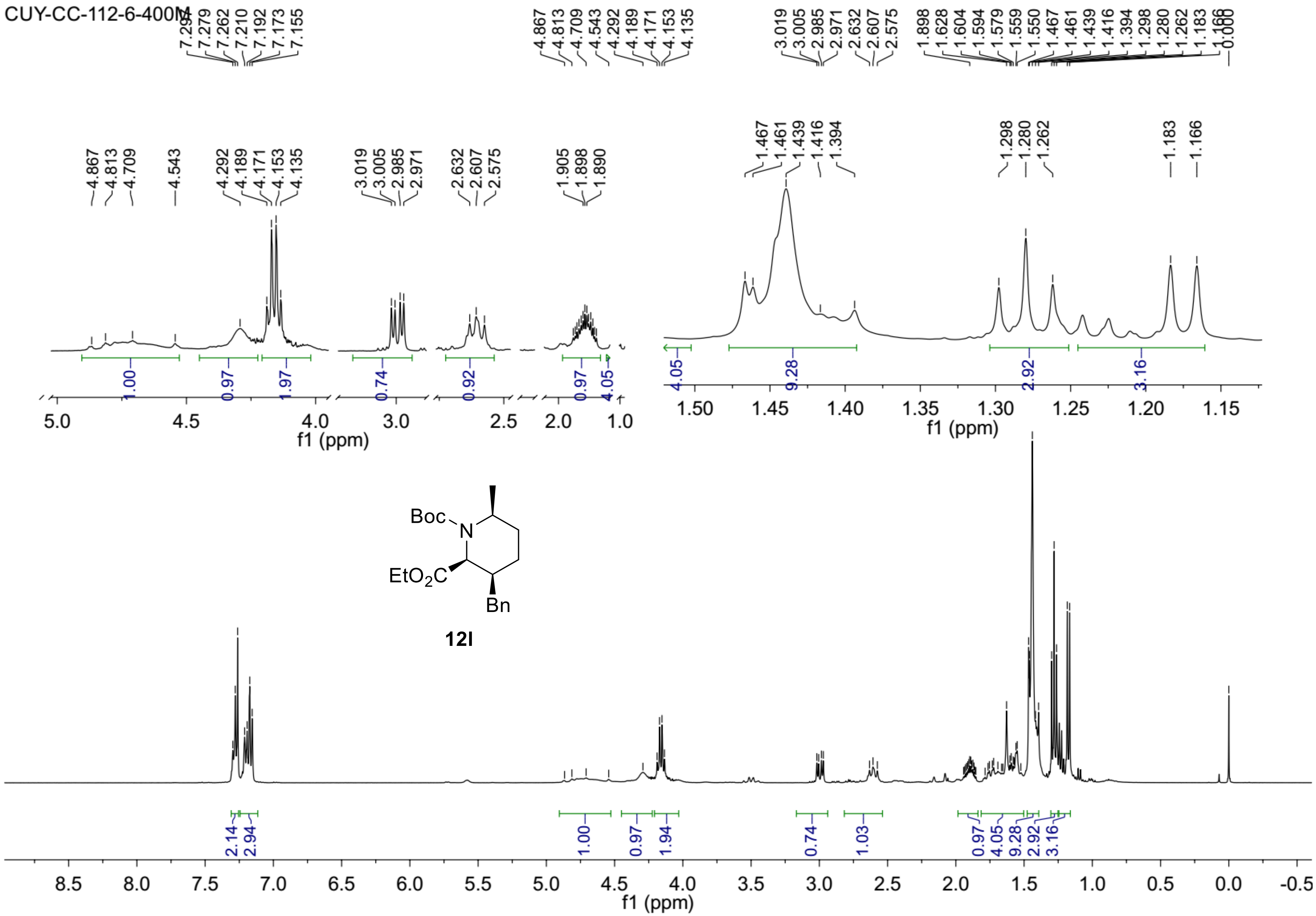

Supplementary Figure 147. ^{1}H NMR (125 MHz, $CDCl_3$) spectra for compound **12l**

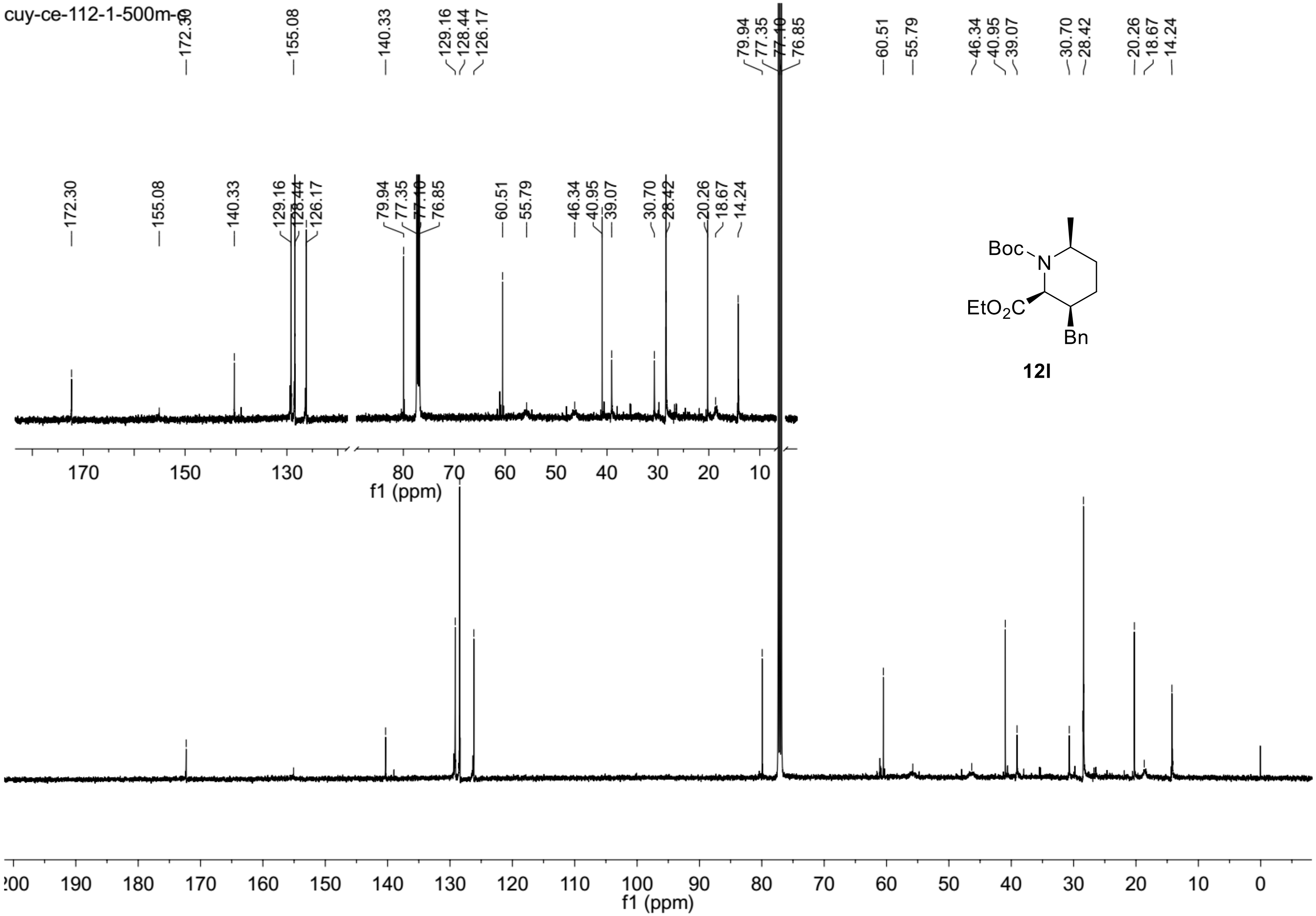

Supplementary Figure 148. ^{13}C NMR (100 MHz, $CDCl_3$) spectra for compound **12l**

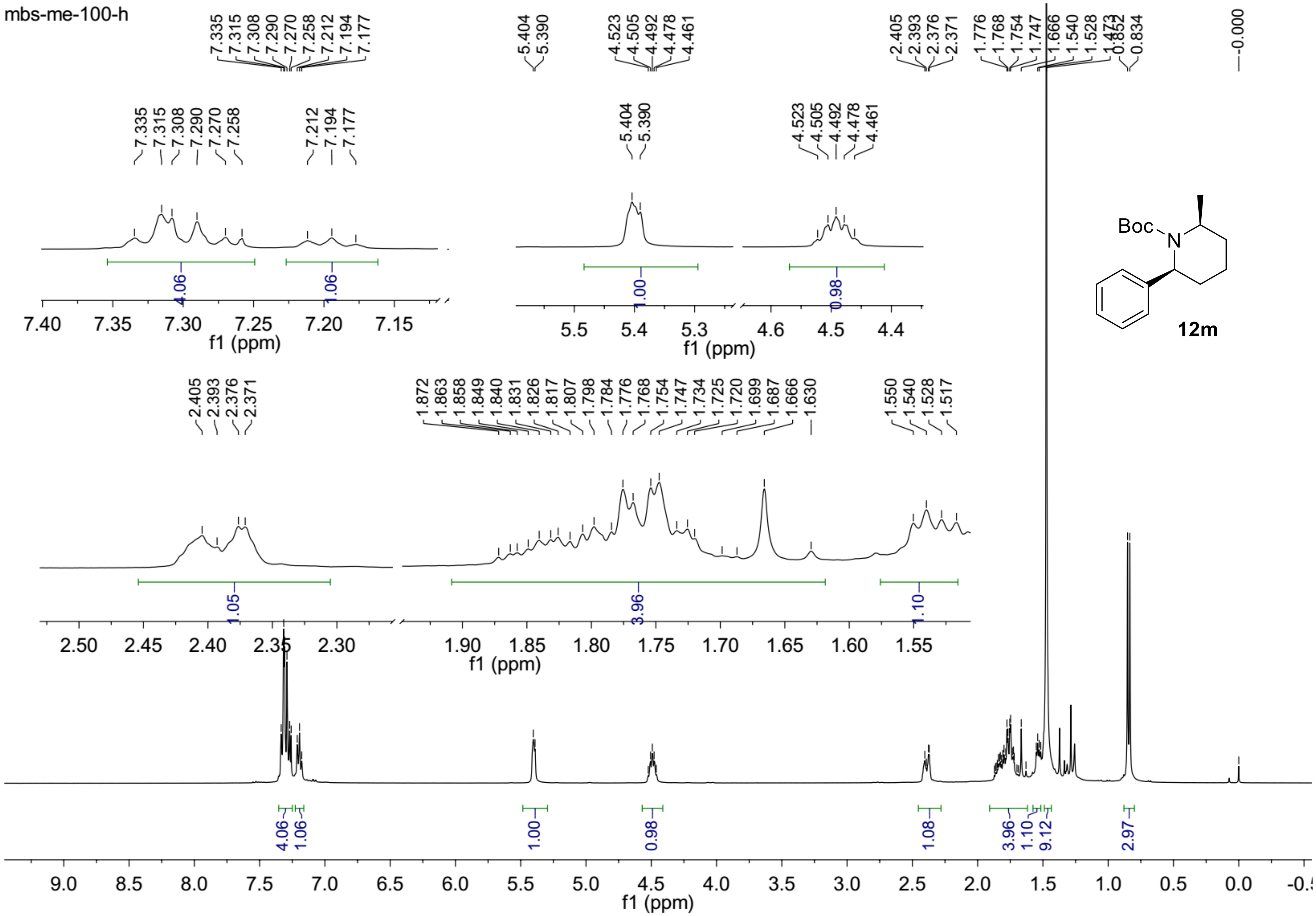

Supplementary Figure 149. ^{1}H NMR (400 MHz, $CDCl_3$) spectra for compound **12m**

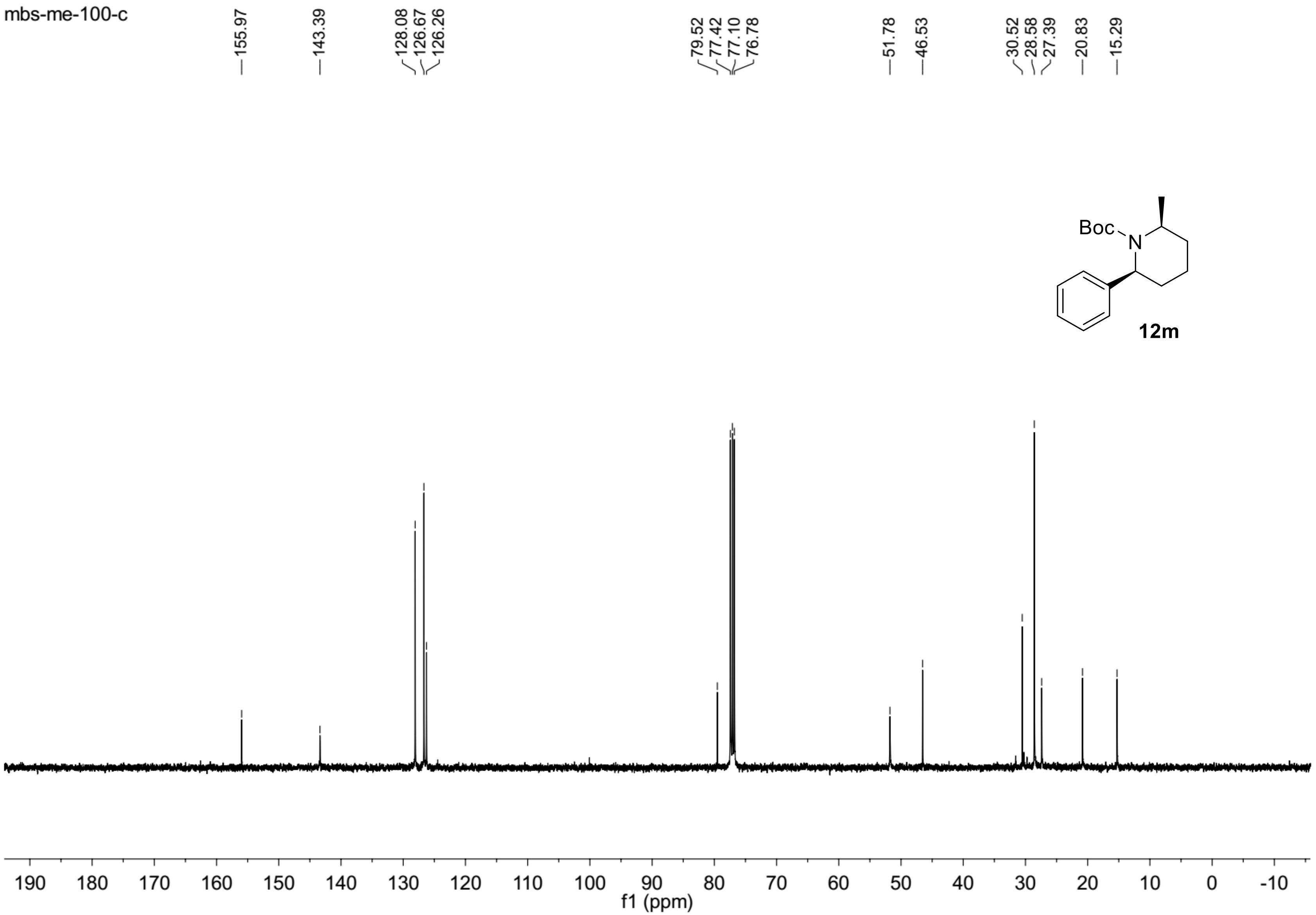

Supplementary Figure 150. ^{13}C NMR (100 MHz, $CDCl_3$) spectra for compound **12m**

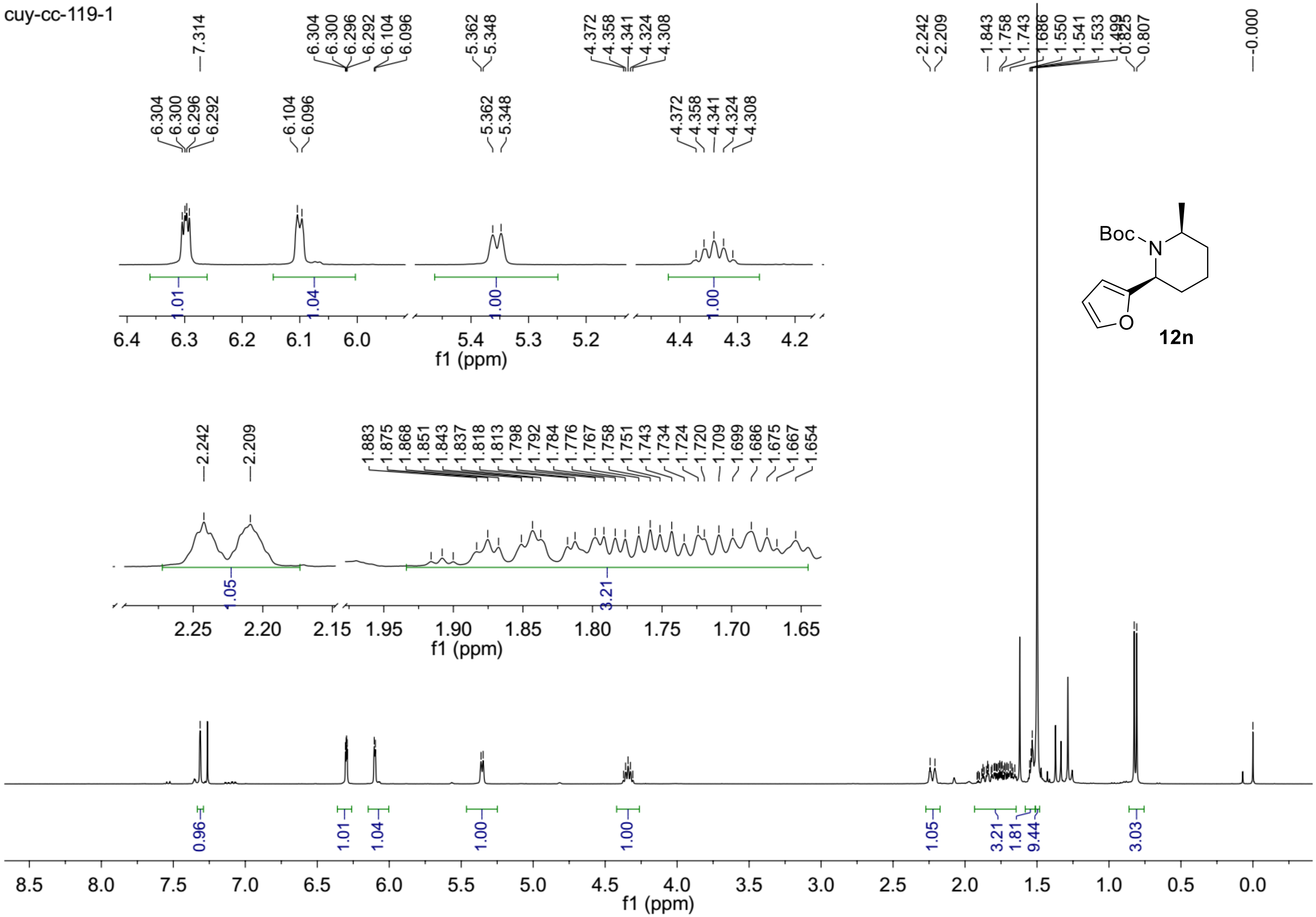

Supplementary Figure 151. ^{1}H NMR (400 MHz, $CDCl_3$) spectra for compound **12n**

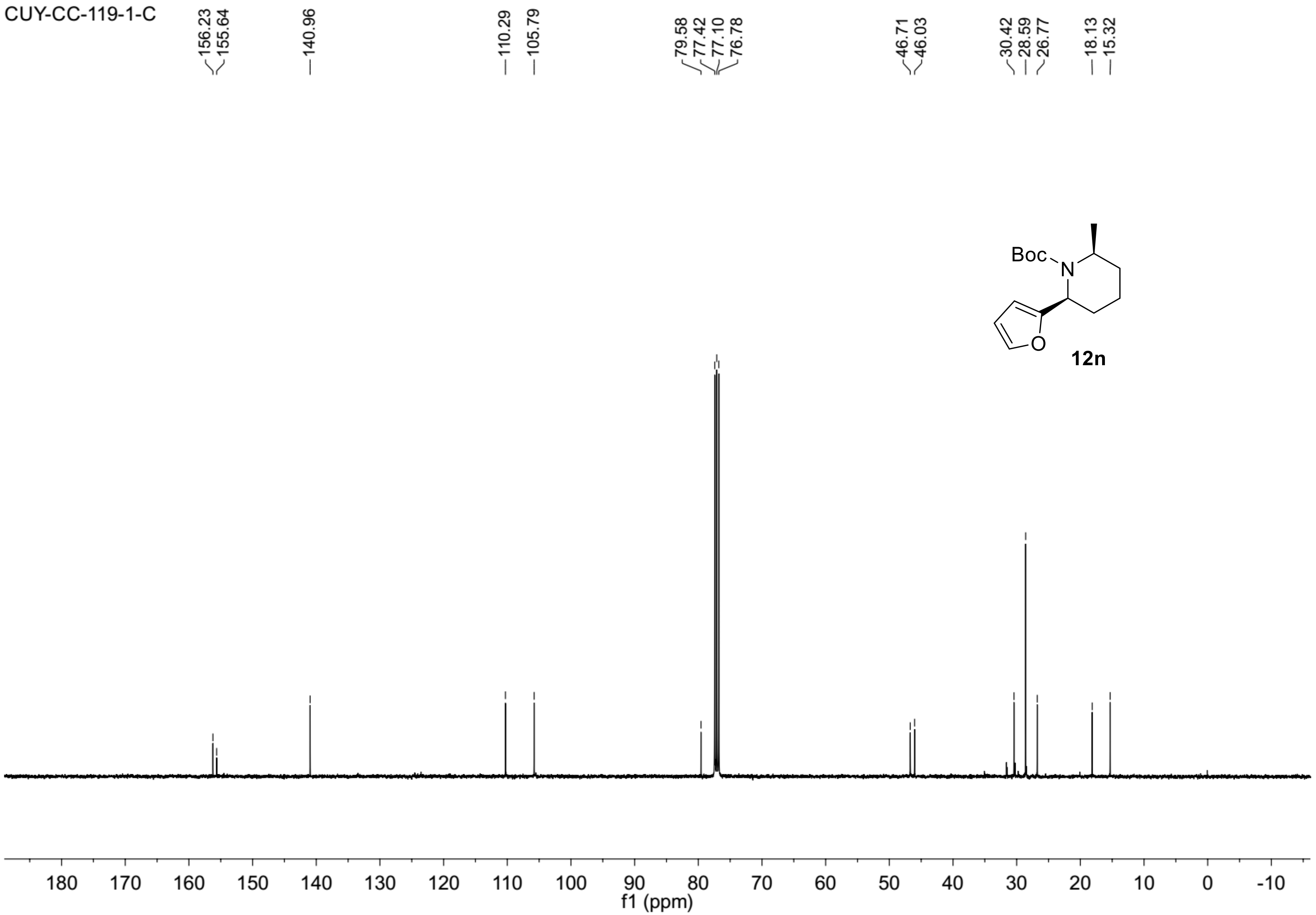

Supplementary Figure 152. ^{13}C NMR (100 MHz, $CDCl_3$) spectra for compound **12n**

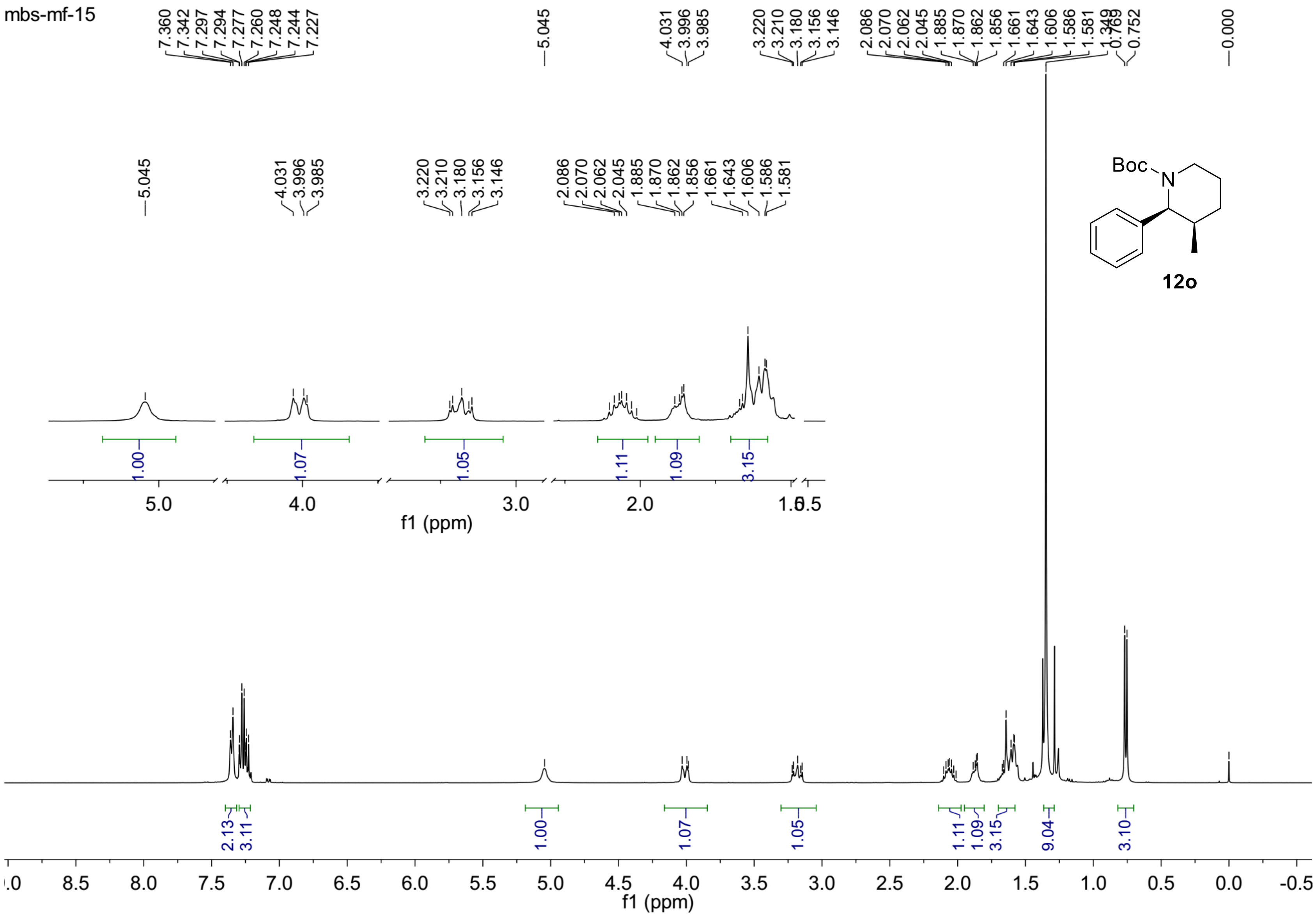

Supplementary Figure 153. ^{1}H NMR (400 MHz, $CDCl_3$) spectra for compound **12o**

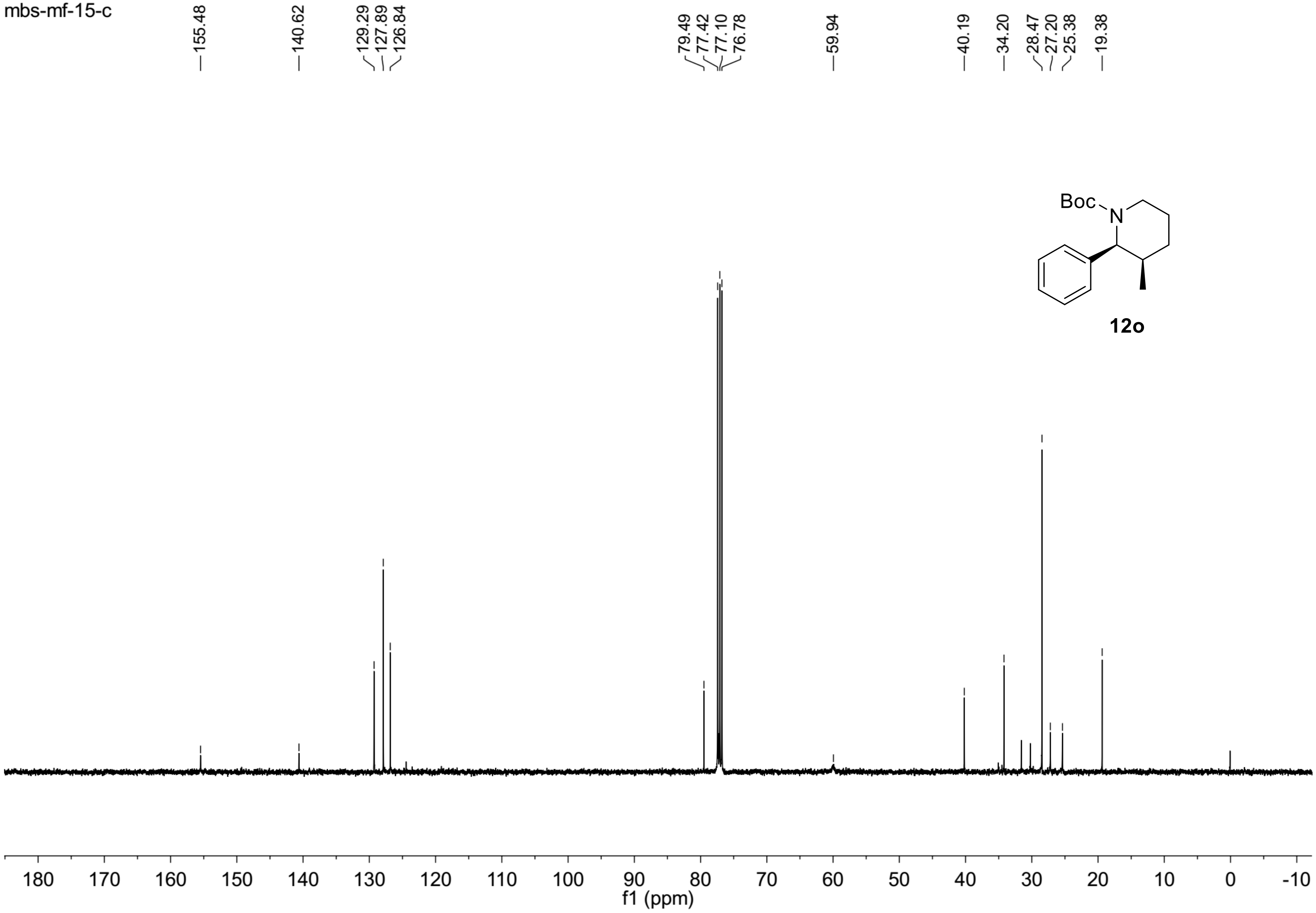

Supplementary Figure 154. ^{13}C NMR (100 MHz, $CDCl_3$) spectra for compound **12o**

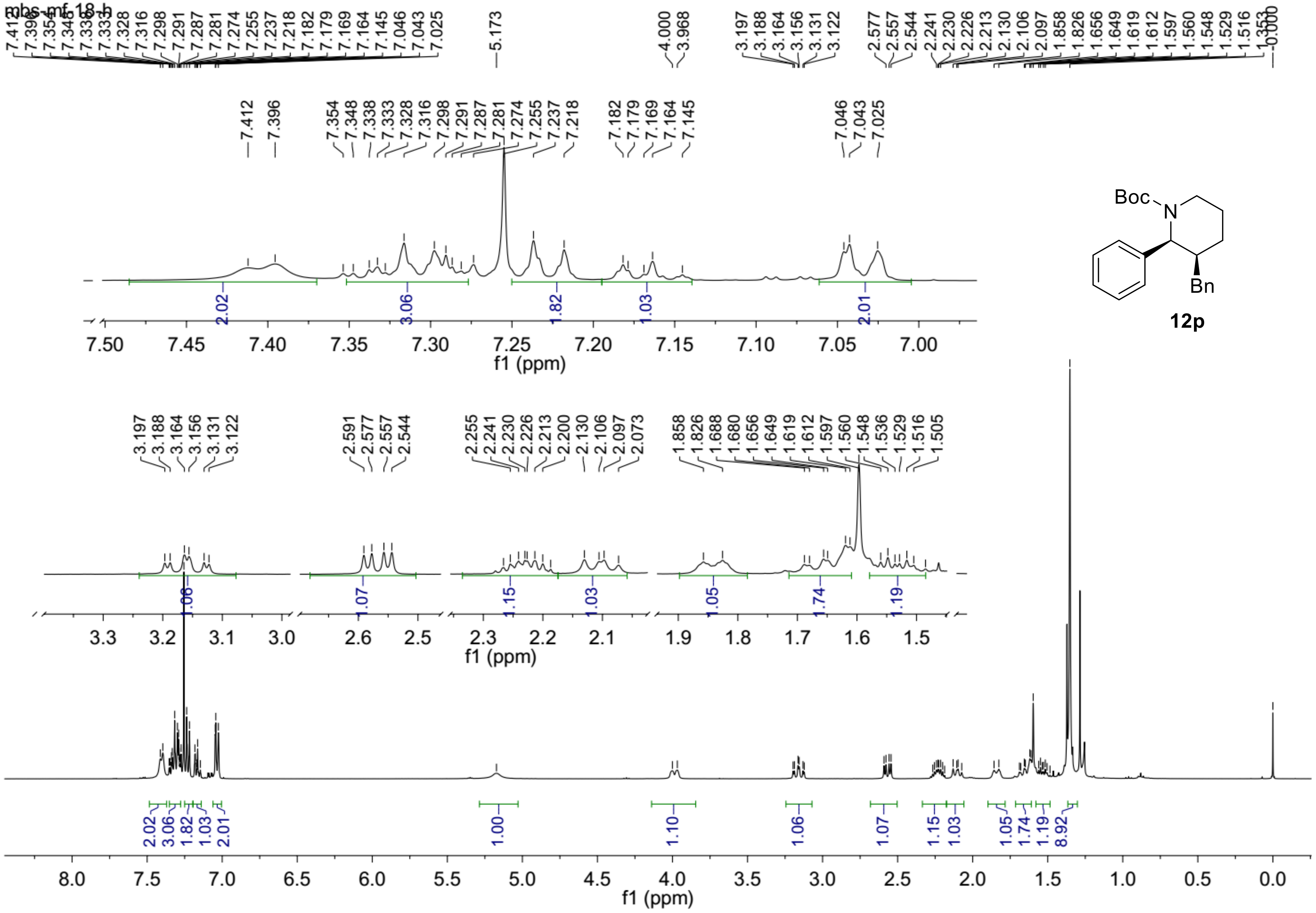

Supplementary Figure 155. ^{1}H NMR (400 MHz, $CDCl_3$) spectra for compound **12p**

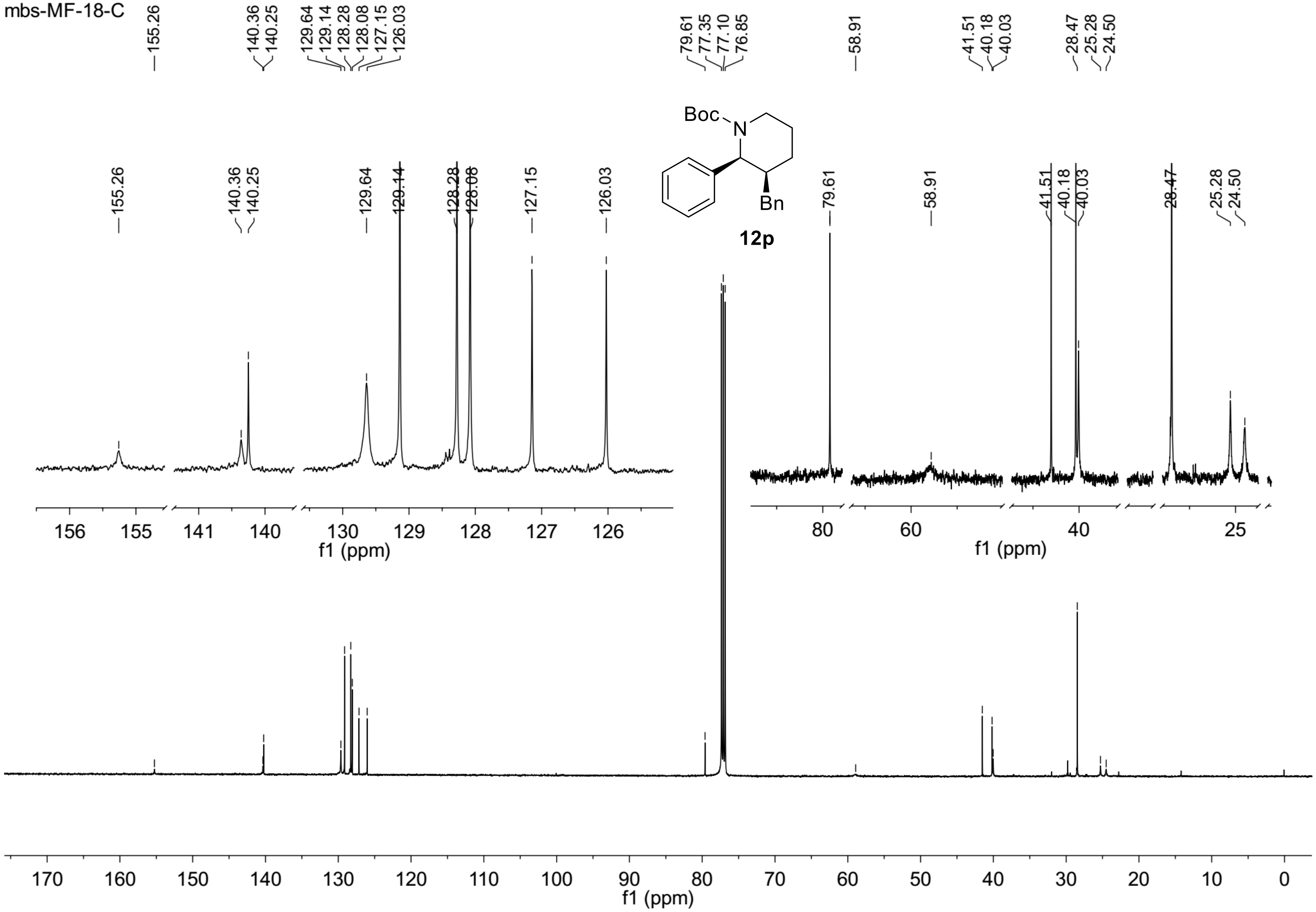

Supplementary Figure 156. ^{13}C NMR (100 MHz, $CDCl_3$) spectra for compound **12p**

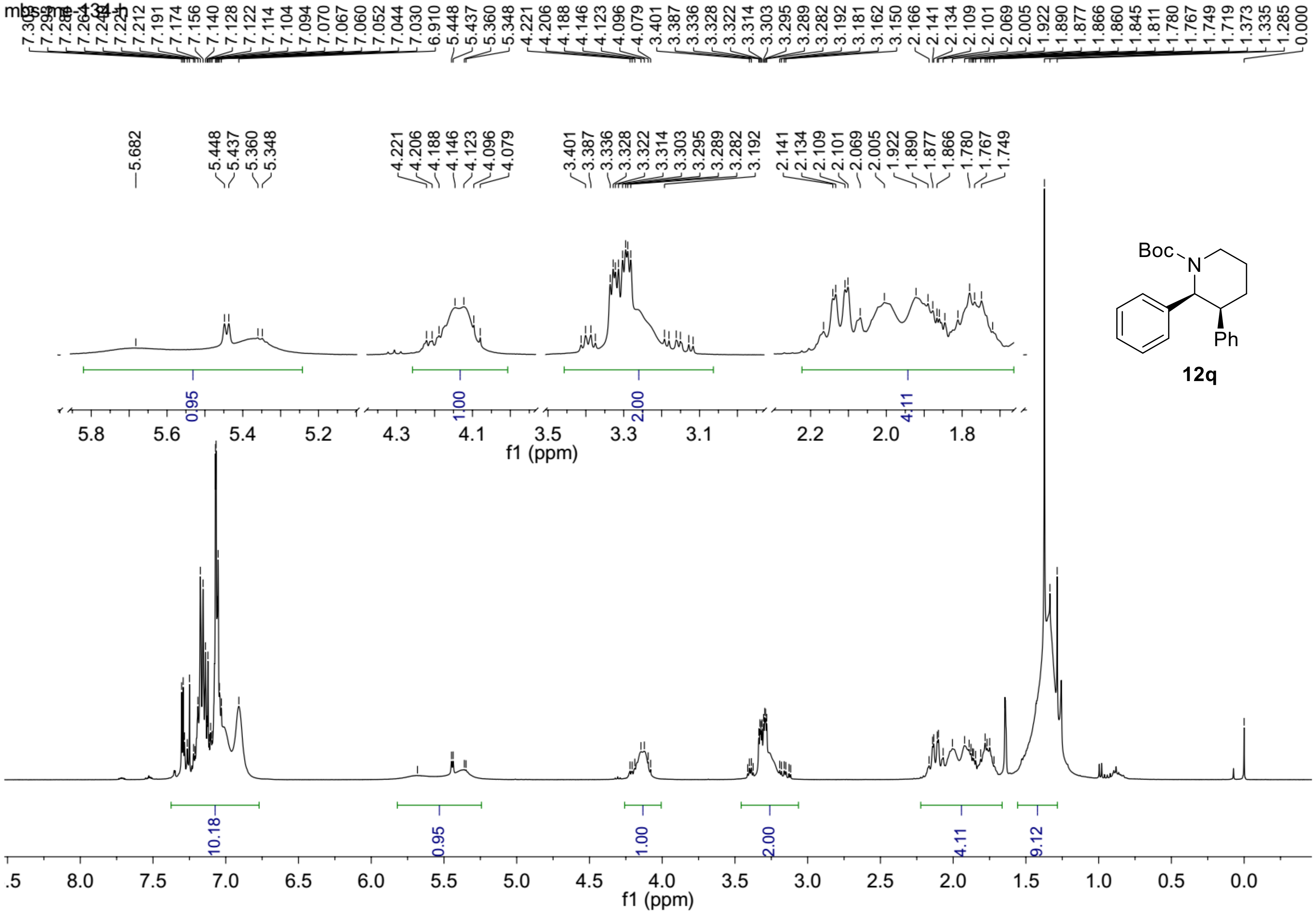

Supplementary Figure 157. ^{1}H NMR (400 MHz, $CDCl_3$) spectra for compound **12q**

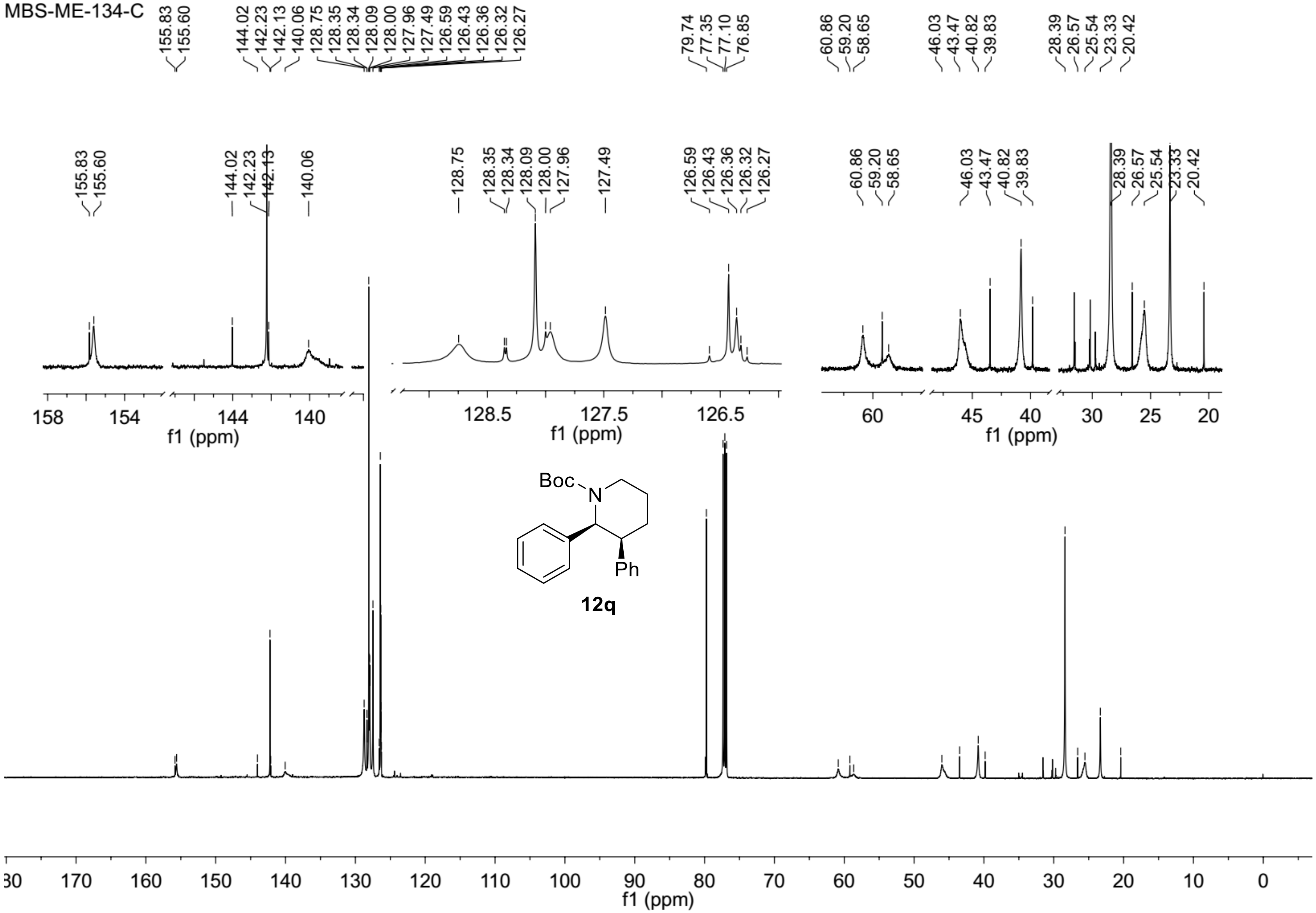

Supplementary Figure 158. ^{13}C NMR (100 MHz, $CDCl_3$) spectra for compound **12q**

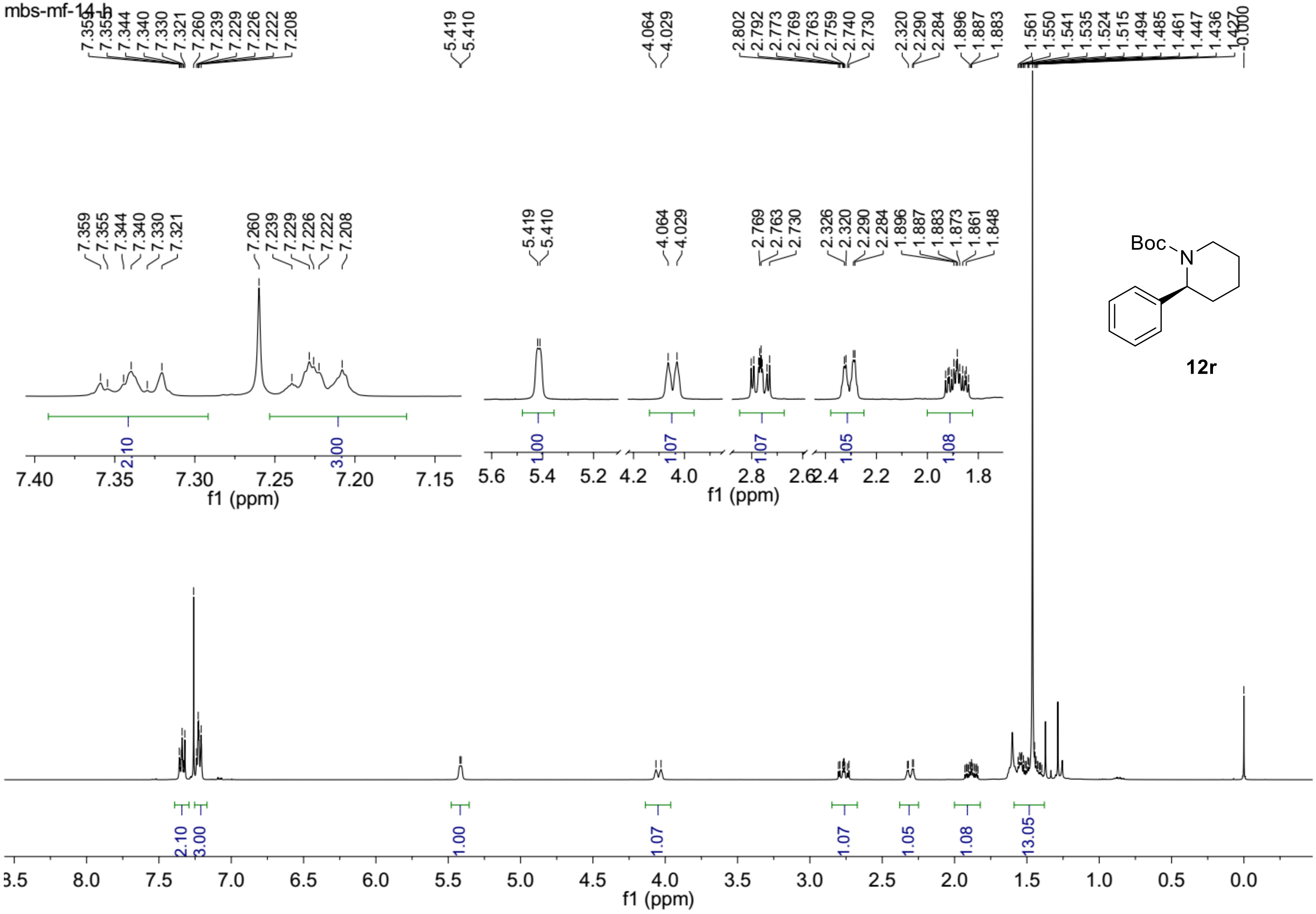

Supplementary Figure 159. ^{1}H NMR (400 MHz, $CDCl_3$) spectra for compound **12r**

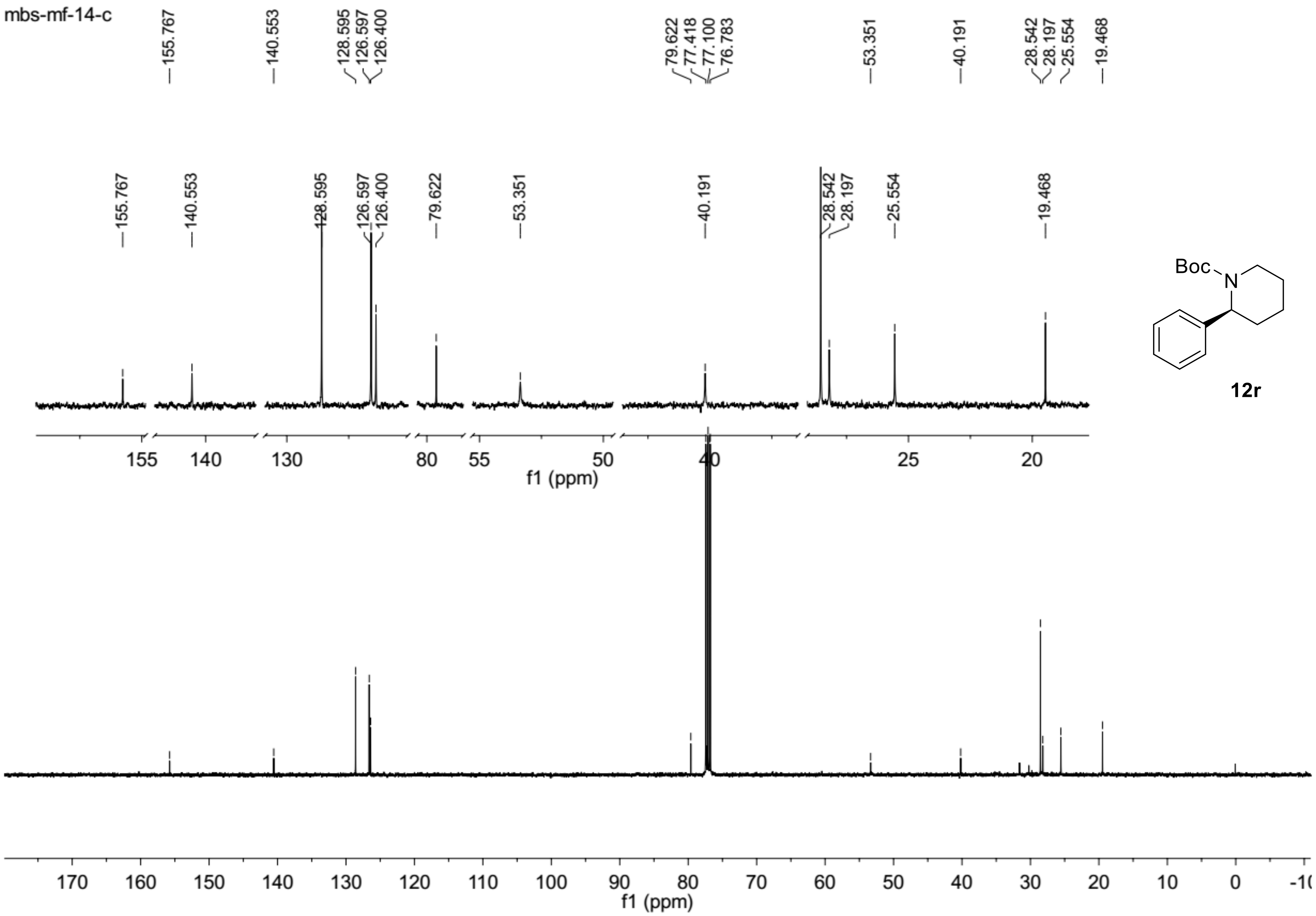

Supplementary Figure 160. ^{13}C NMR (100 MHz, $CDCl_3$) spectra for compound **12r**

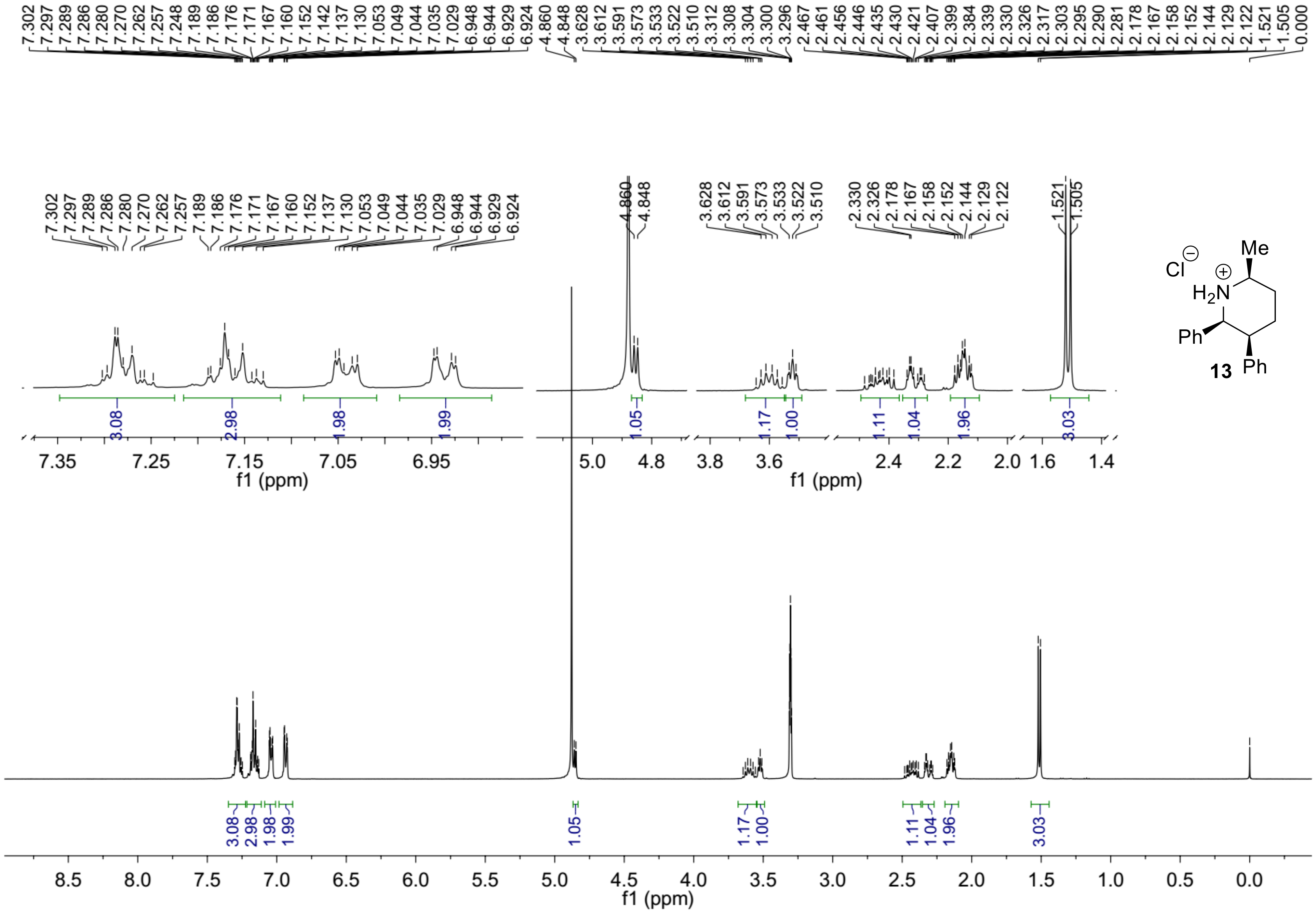

Supplementary Figure 161. ^{1}H NMR (400 MHz, $CDCl_3$) spectra for compound **13**

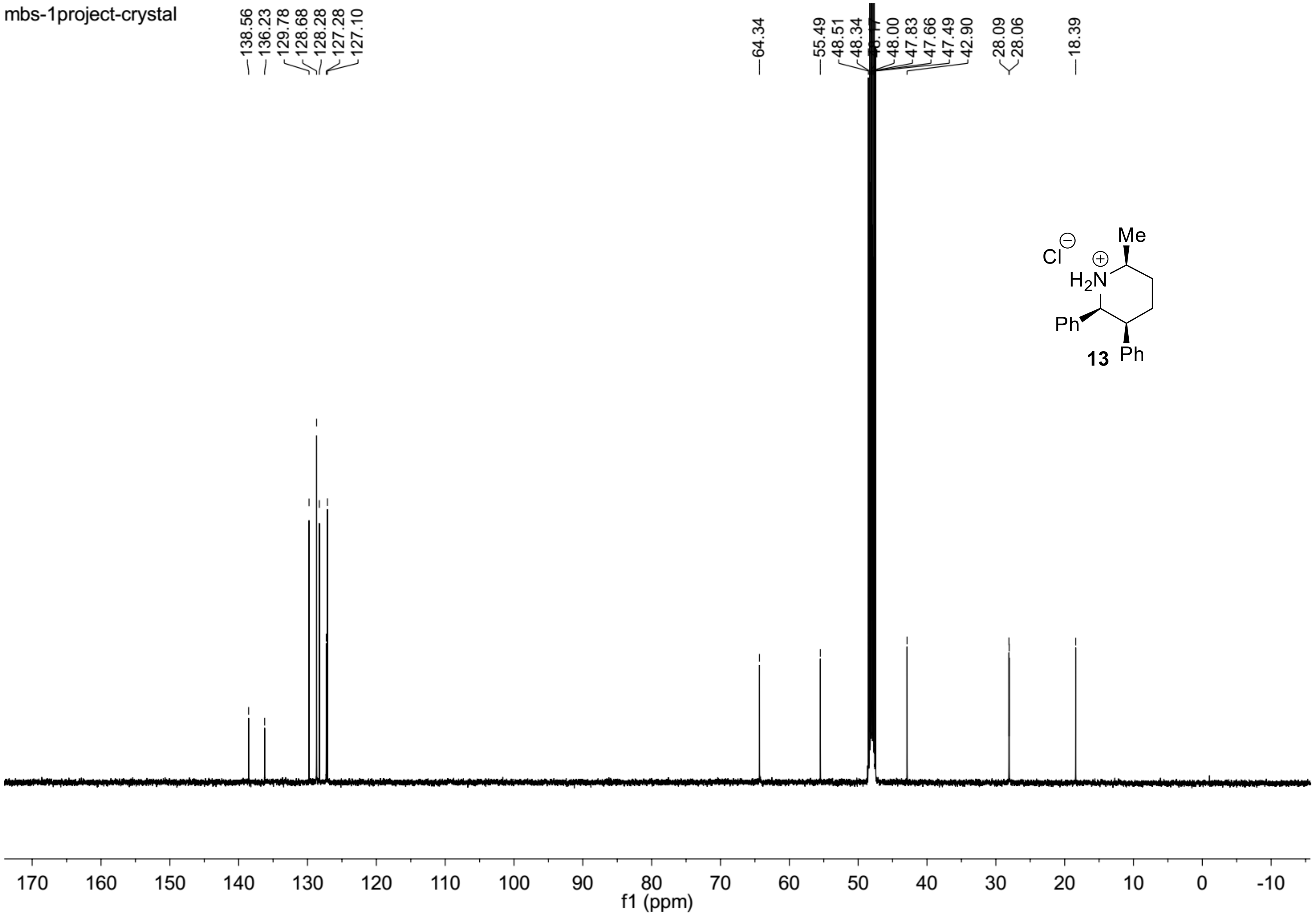

Supplementary Figure 162. ^{13}C NMR (125 MHz, $CDCl_3$) spectra for compound **13**

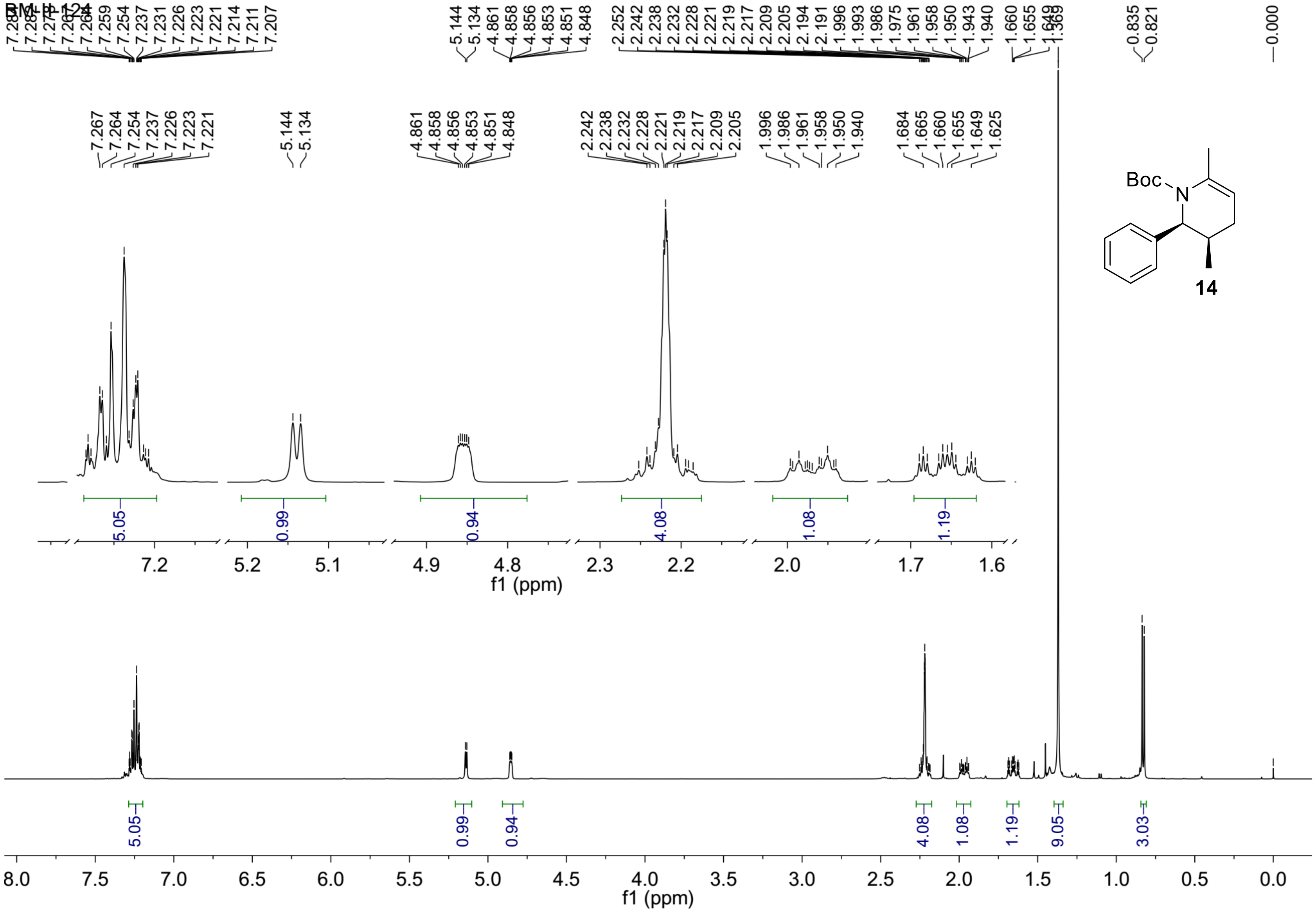

Supplementary Figure 163. ^{1}H NMR (500 MHz, $CDCl_3$) spectra for compound **14**

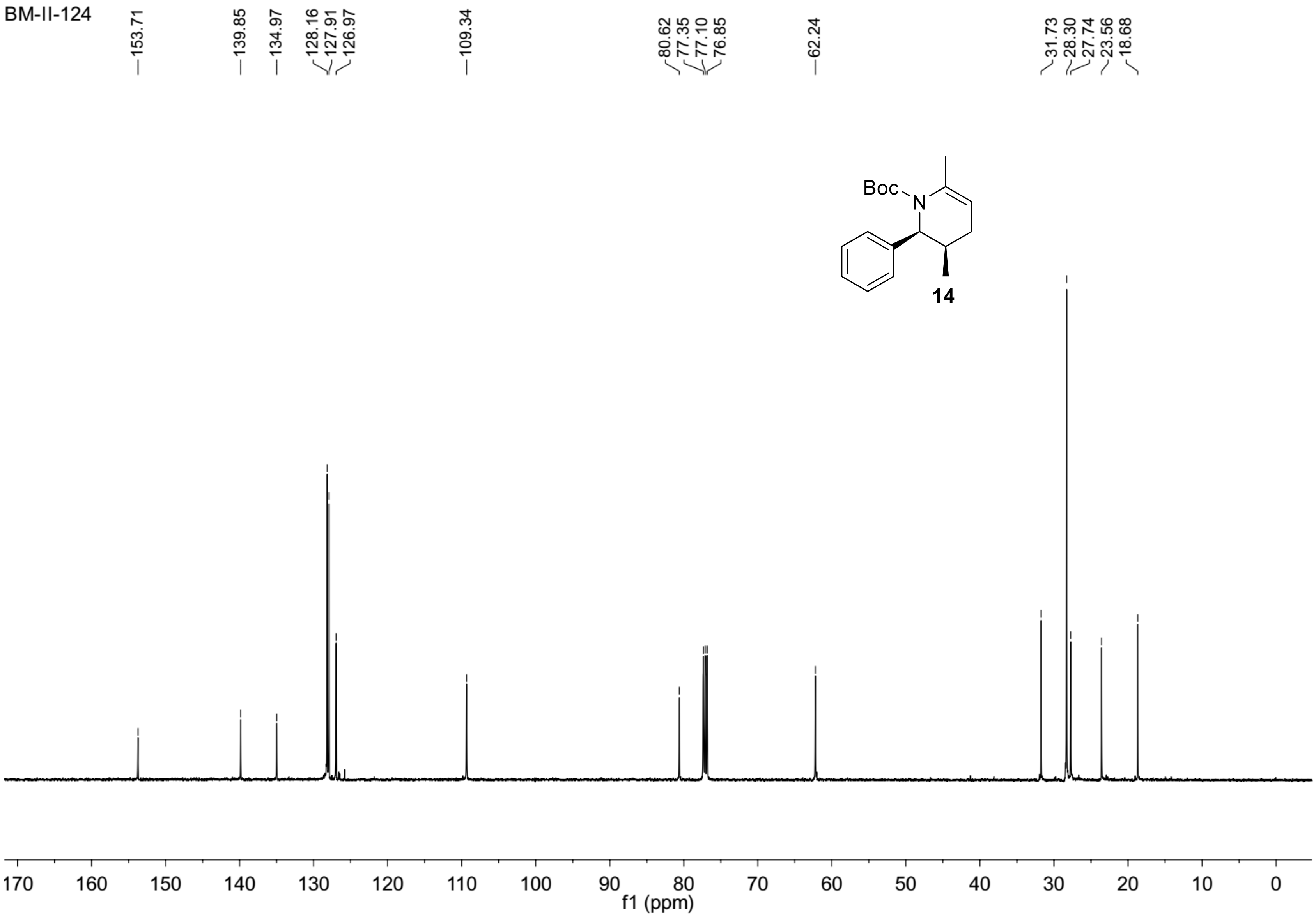

Supplementary Figure 164. ^{13}C NMR (125 MHz, $CDCl_3$) spectra for compound **14**

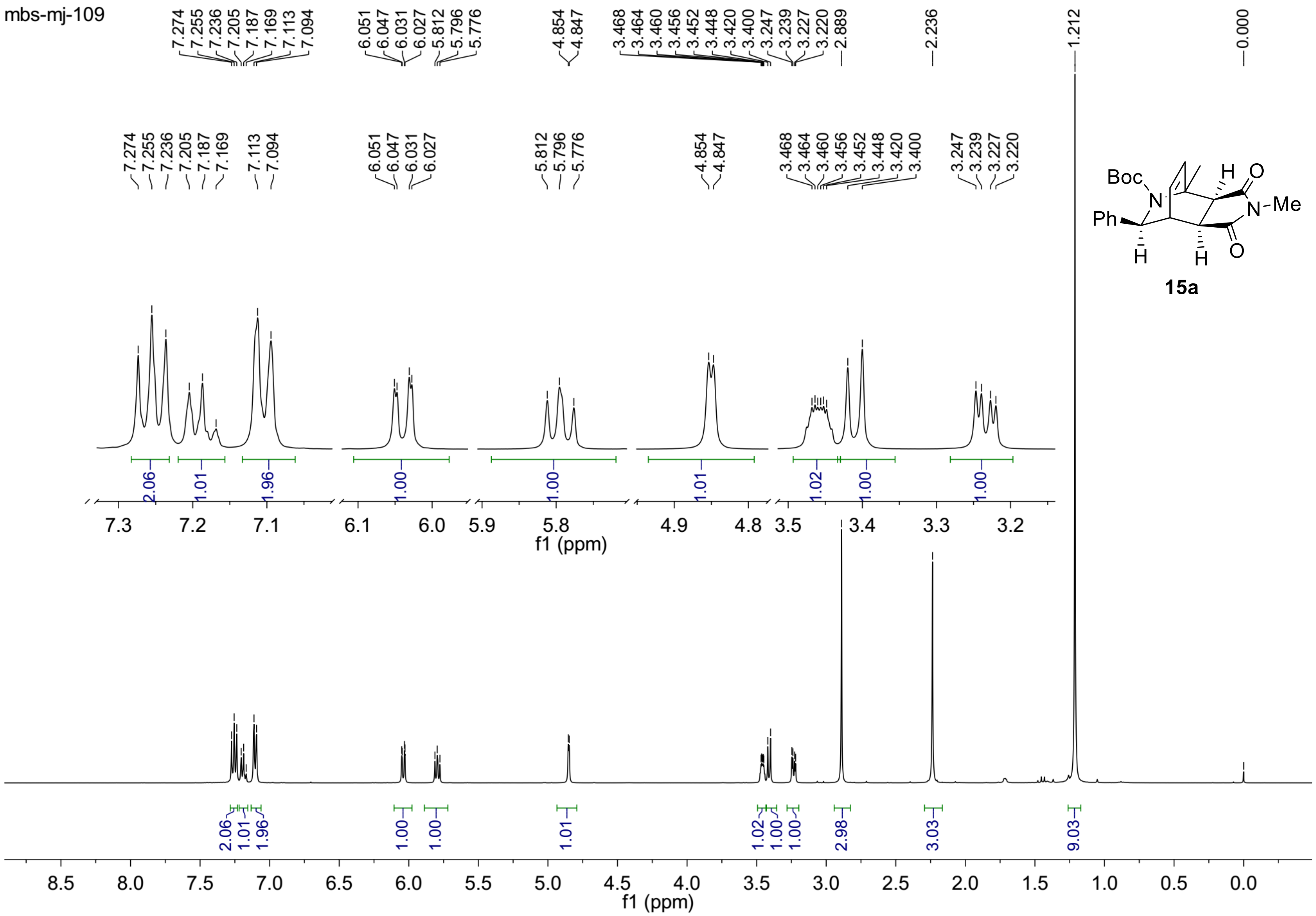

Supplementary Figure 165. ^{1}H NMR (400 MHz, $CDCl_3$) spectra for compound **15a**

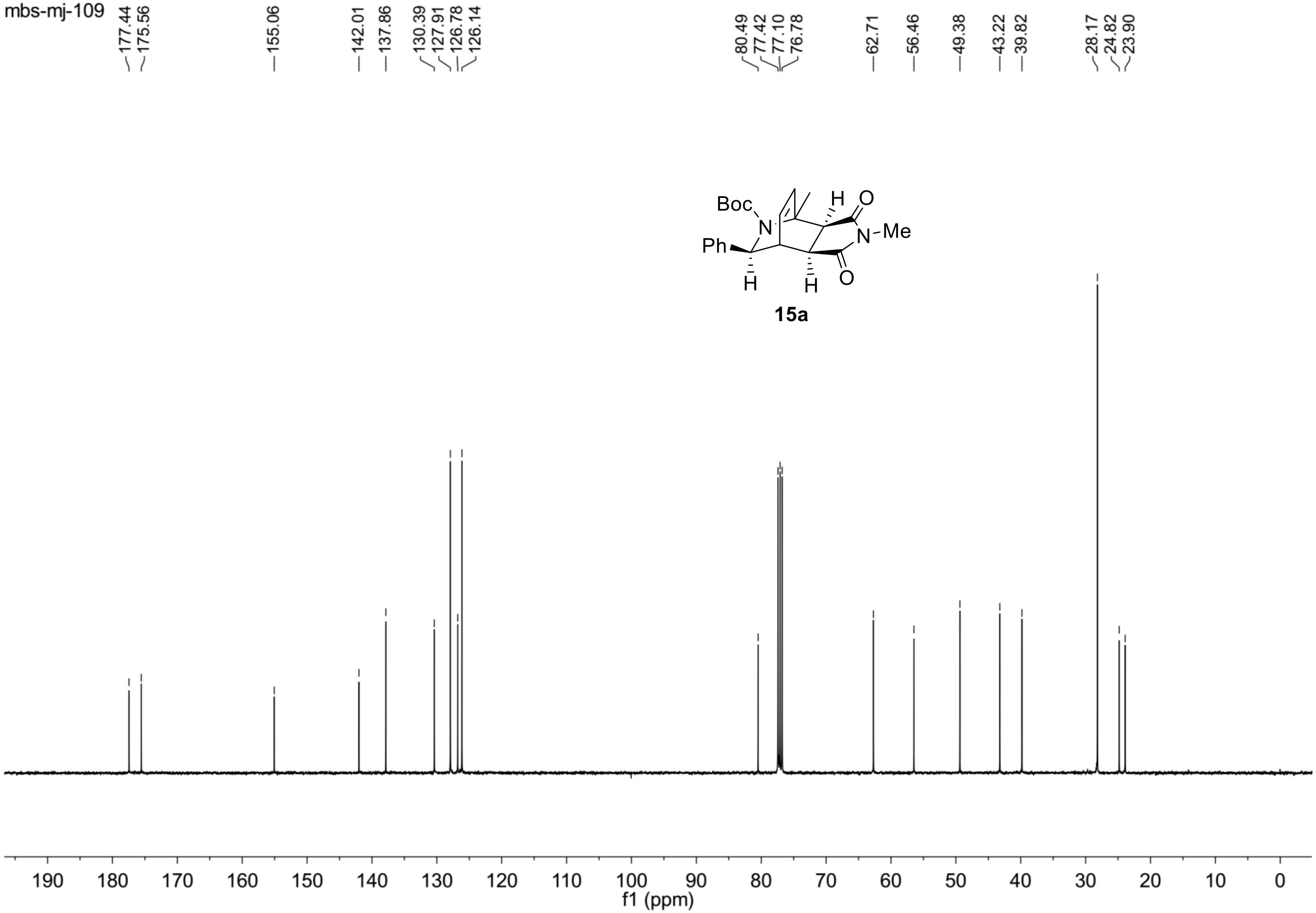

Supplementary Figure 166. ^{13}C NMR (100 MHz, $CDCl_3$) spectra for compound **15a**

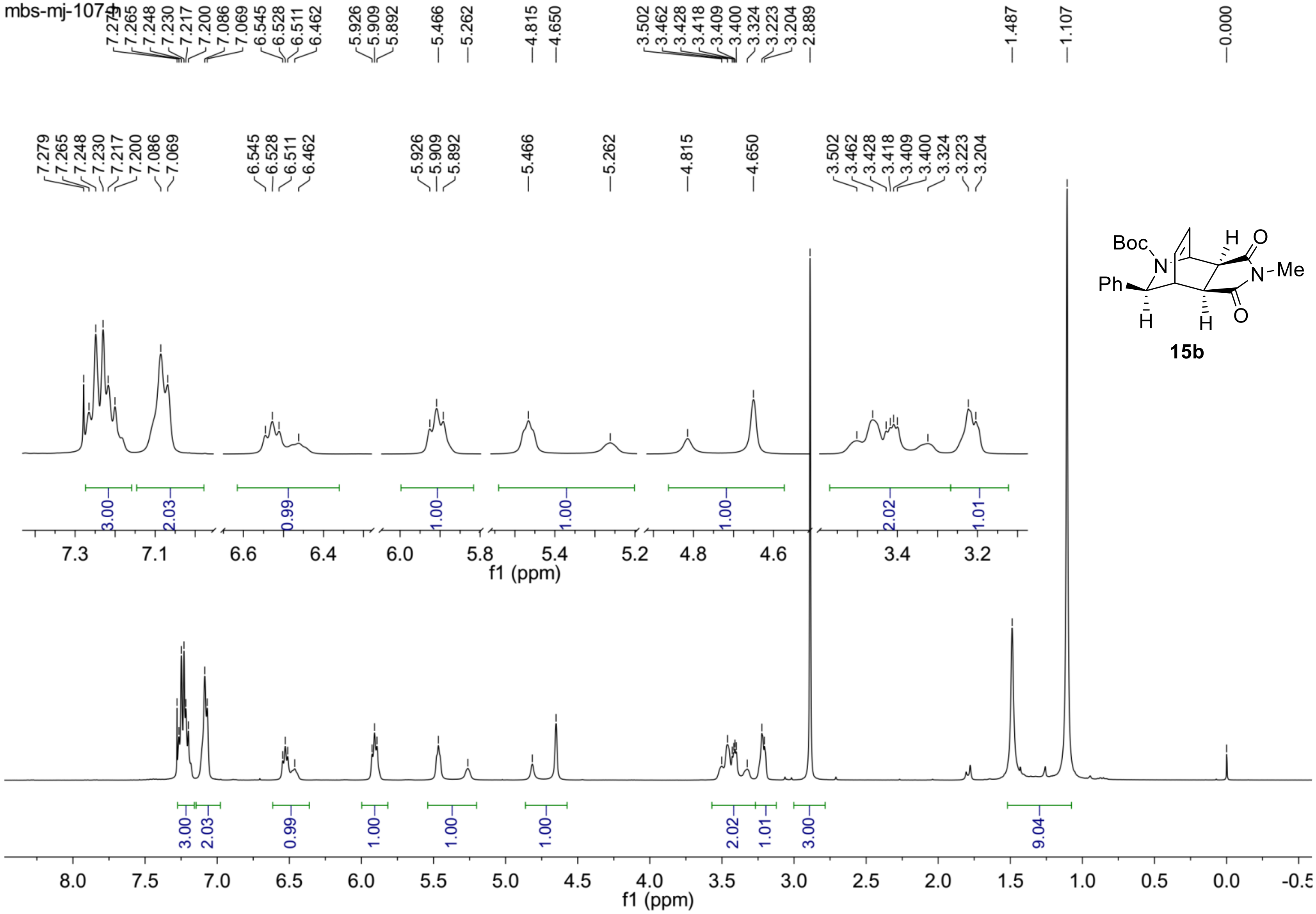

Supplementary Figure 167. ^{1}H NMR (400 MHz, $CDCl_3$) spectra for compound **15b**

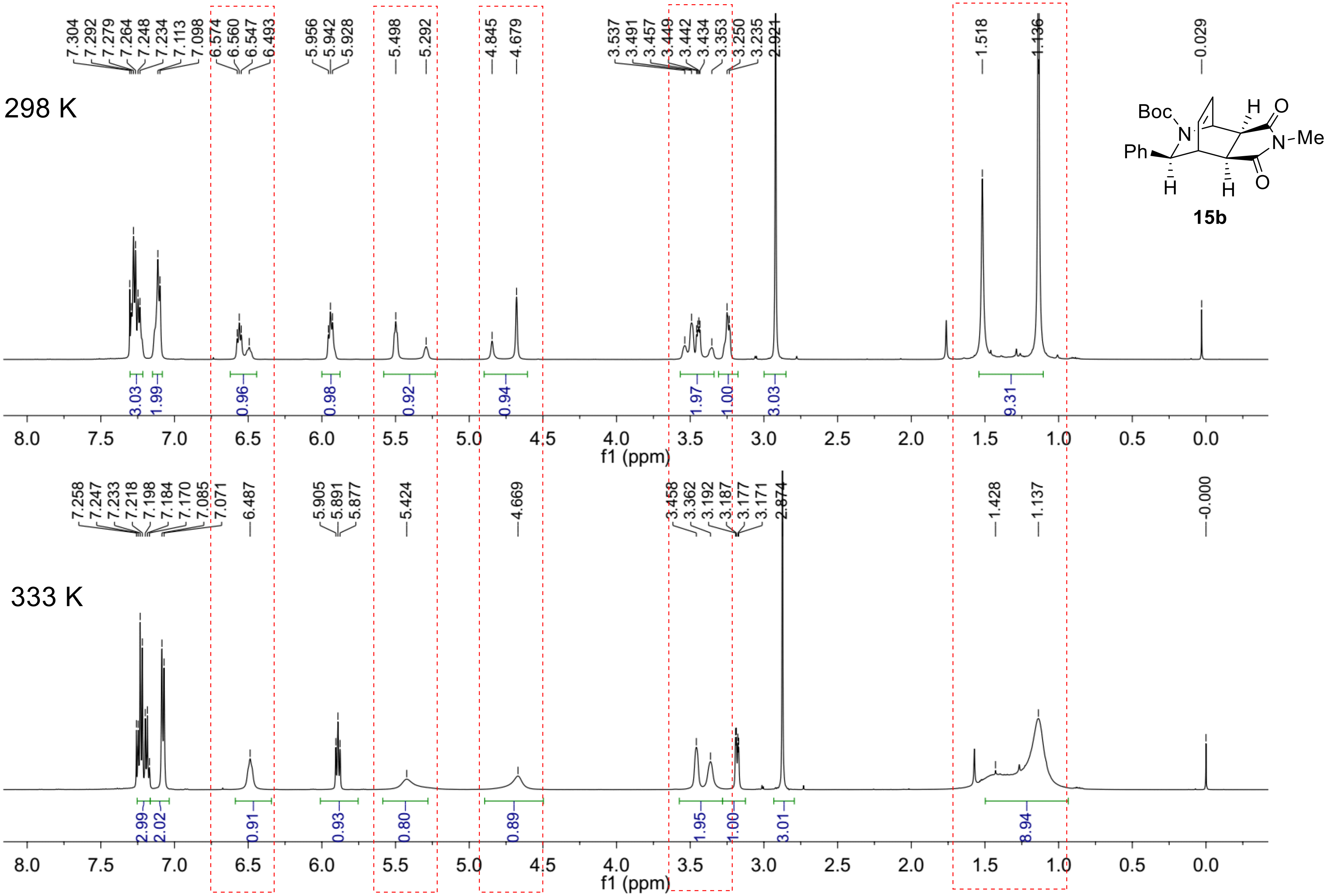

Supplementary Figure 168. VT-NMR for compound **15b**

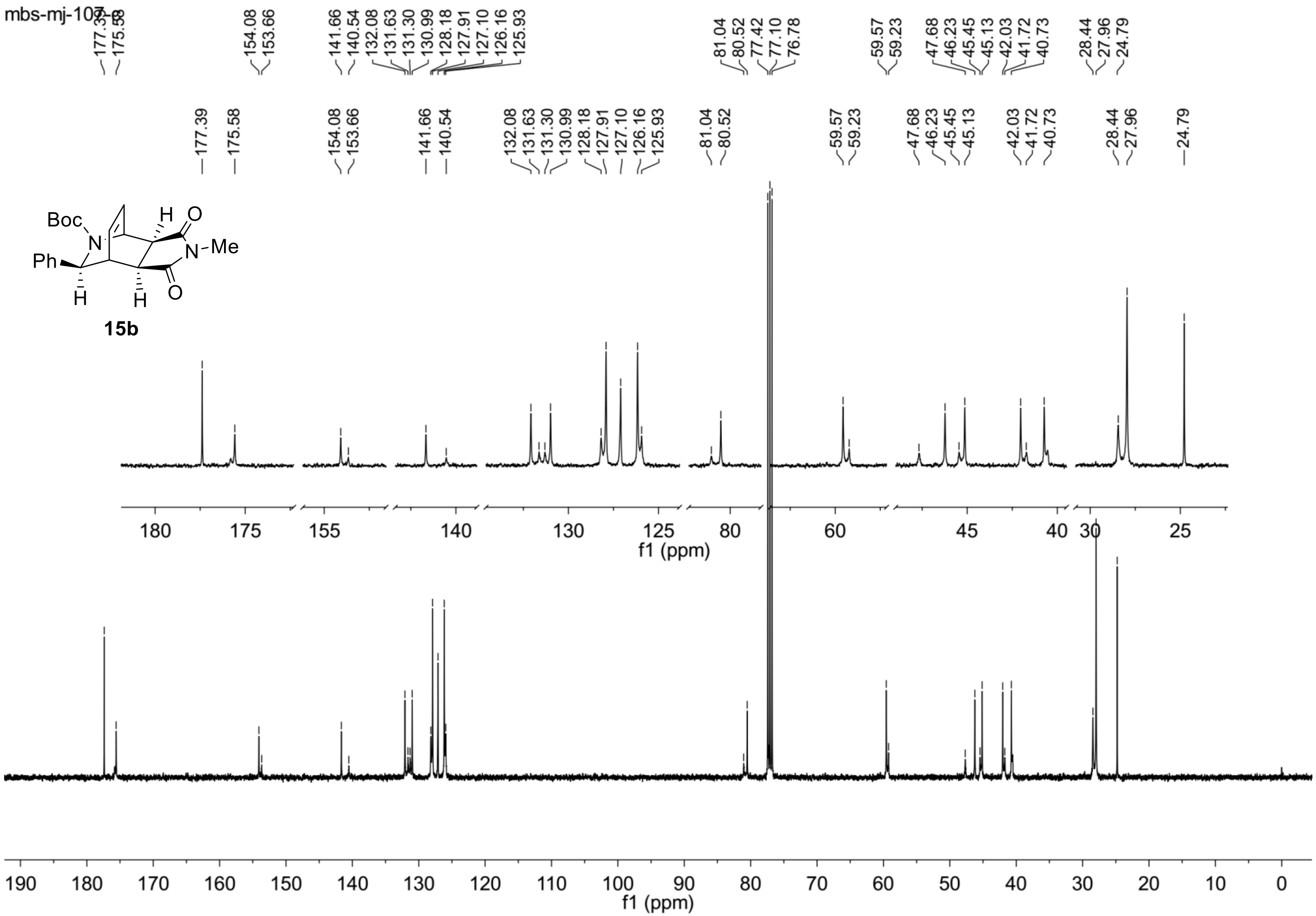

Supplementary Figure 169. ^{13}C NMR (100 MHz, $CDCl_3$) spectra for compound **15b**

HPLC spectra

2017/5/29 星期一 11:01:52 Page 1 / 1

SHIMADZU LabSolutions **Analysis Report**

<Sample Information>

Sample Name : mbs-i-43-s-adh-99.5-0.5-1.0-2nd
Sample ID :
Data Filename : mbs-i-43-s-adh-99.5-0.5-1.0-2nd1.lcd
Method Filename : zry-cl.lcm
Batch Filename :
Vial # : 1-1 Sample Type : Unknown
Injection Volume : 20 uL
Date Acquired : 2017/3/15 星期三 19:48:15 Acquired by : System Administrator
Date Processed : 2017/3/16 星期四 20:44:24 Processed by : System Administrator

<Chromatogram>

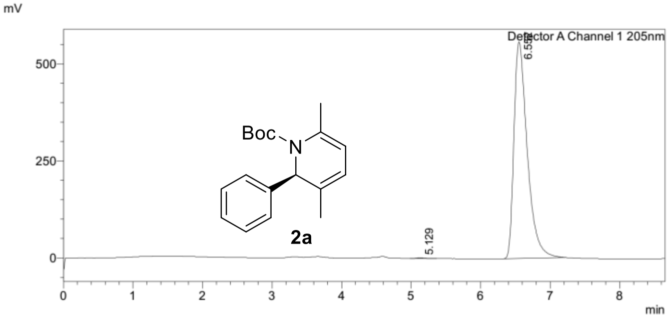

<Peak Table>

Detector A Channel 1 205nm

Peak#	Ret. Time	Area	Height	Conc.
1	5.129	13851	1872	0.187
2	6.552	7410934	557457	99.813
Total		7424784	559329	

D:\Data\mbs\mbs-i-43-s-adh-99.5-0.5-1.0-2nd1.lcd

2020/7/22 22:04:38 Page 1 / 1

Analysis Report

<Sample Information>

Data Filename : mbs-i-47-rac-adh-99.5-0.5-1.0-2nd1.lcd
Method Filename : zry-cl.lcm
Batch Filename :
Vial # : 1-1 Sample Type : Unknown
Injection Volume : 20 uL
Date Acquired : 2017/3/15 19:20:14 Acquired by : System Administrator
Date Processed : 2017/3/16 20:43:50 Processed by : System Administrator

<Chromatogram>

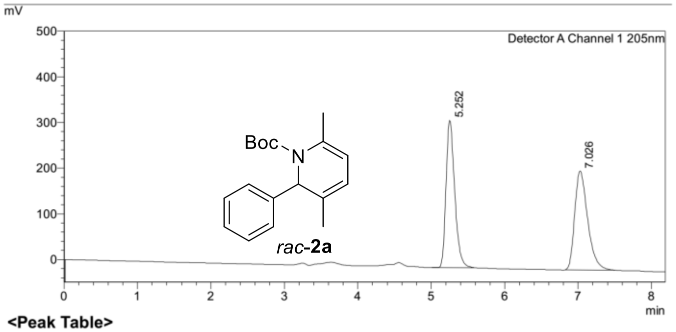

<Peak Table>

Detector A Channel 1 205nm

Peak#	Ret. Time	Area	Height	Conc.
1	5.252	2646610	322507	49.966
2	7.026	2650192	216433	50.034
Total		5296802	538940	

D:\Data\mbs\mbs-i-47-rac-adh-99.5-0.5-1.0-2nd1.lcd

Supplementary Figure 170. HPLC analysis for compound **2a**

Analysis Report

SHIMADZU LabSolutions

<Sample Information>

Sample Name : mbs-i-74-s-adh-99.5-0.5-1.0
Sample ID :
Data Filename : mbs-i-74-s-adh-99.5-0.5-1.0.lcd
Method Filename : wyh-wb-17-rac-ADH-1.0-230-254.lcm
Batch Filename :
Vial # : 1-1 Sample Type : Unknown
Injection Volume : 20 uL
Date Acquired : 2017/3/14 星期二 16:48:47 Acquired by : System Administrator
Date Processed : 2017/3/16 星期四 20:39:02 Processed by : System Administrator

<Chromatogram>

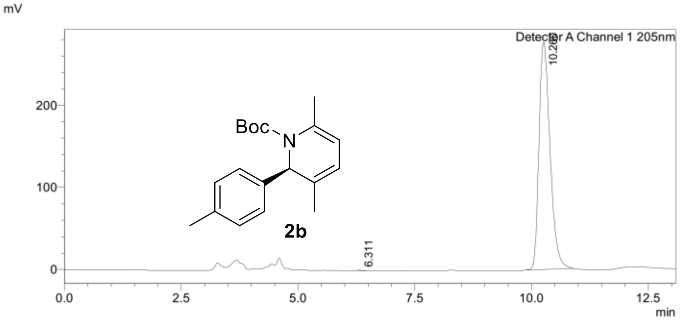

<Peak Table>

Detector A Channel 1 205nm

Peak#	Ret. Time	Area	Height	Conc.
1	6.311	3069	490	0.067
2	10.260	4544762	276513	99.933
Total		4547831	277003	

Analysis Report

SHIMADZU LabSolutions

<Sample Information>

Sample Name : mbs-i-88-rac-adh-99.5-0.5-1.0
Sample ID :
Data Filename : mbs-i-88-rac-adh-99.5-0.5-1.0.lcd
Method Filename : wyh-wb-17-rac-ADH-1.0-230-254.lcm
Batch Filename :
Vial # : 1-1 Sample Type : Unknown
Injection Volume : 20 uL
Date Acquired : 2017/3/14 星期二 16:33:43 Acquired by : System Administrator
Date Processed : 2017/3/16 星期四 20:37:41 Processed by : System Administrator

<Chromatogram>

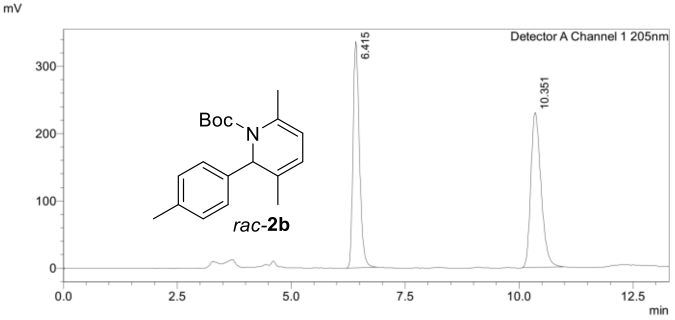

<Peak Table>

Detector A Channel 1 205nm

Peak#	Ret. Time	Area	Height	Conc.
1	6.415	3273660	336014	47.627
2	10.351	3599870	230043	52.373
Total		6873529	566056	

Supplementary Figure 171. HPLC analysis for compound **2b**

Analysis Report

SHIMADZU LabSolutions

<Sample Information>

Sample Name	: mbs-i-94-s-adh-99.5-0.5-1.0		
Sample ID	:		
Data Filename	: mbs-i-94-s-adh-99.5-0.5-1.0.lcd		
Method Filename	: zry-cl.lcm		
Batch Filename	:		
Vial #	: 1-1	Sample Type	: Unknown
Injection Volume	: 20 uL		
Date Acquired	: 2017/3/15 星期三 22:22:17	Acquired by	: System Administrator
Date Processed	: 2017/3/16 星期四 20:40:09	Processed by	: System Administrator

<Chromatogram>

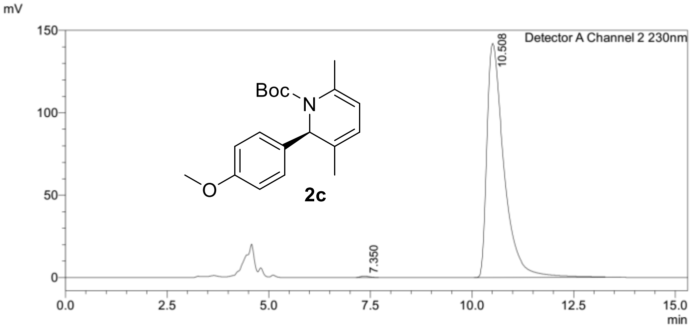

<Peak Table>

Detector A Channel 2 230nm

Peak#	Ret. Time	Area	Height	Conc.
1	7.350	13562	940	0.326
2	10.508	4150403	142037	99.674
Total		4163965	142977	

Analysis Report

SHIMADZU LabSolutions

<Sample Information>

Sample Name	: mbs-i-95-s-adh-99.5-0.5-1.0		
Sample ID	:		
Data Filename	: mbs-i-95-rac-adh-99.5-0.5-1.0.lcd		
Method Filename	: zry-cl.lcm		
Batch Filename	:		
Vial #	: 1-1	Sample Type	: Unknown
Injection Volume	: 20 uL		
Date Acquired	: 2017/3/15 星期三 21:47:56	Acquired by	: System Administrator
Date Processed	: 2017/5/29 星期一 11:29:08	Processed by	: System Administrator

<Chromatogram>

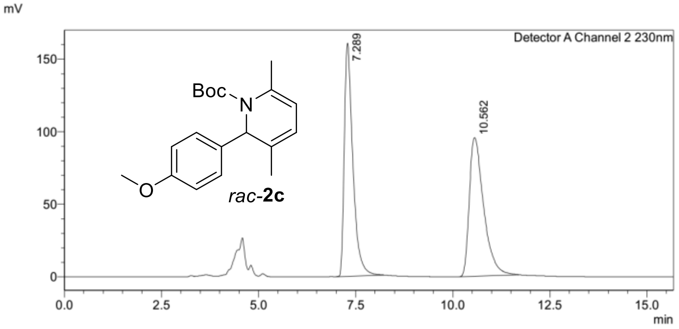

<Peak Table>

Detector A Channel 2 230nm

Peak#	Ret. Time	Area	Height	Conc.
1	7.289	2458595	160545	50.083
2	10.562	2450438	95567	49.917
Total		4909033	256111	

Supplementary Figure 172. HPLC analysis for compound **2c**

SHIMADZU LabSolutions

Analysis Report

<Sample Information>

Sample Name : mbs-i-114-s-adh-99.5-0.5-1.0
Sample ID :
Data Filename : mbs-i-114-s-adh-99.5-0.5-1.0.lcd
Method Filename : zry-cl.lcm
Batch Filename :
Vial # : 1-1 Sample Type : Unknown
Injection Volume : 20 uL
Date Acquired : 2017/4/12 星期三 22:00:49 Acquired by : System Administrator
Date Processed : 2017/4/12 星期三 22:12:53 Processed by : System Administrator

<Chromatogram>

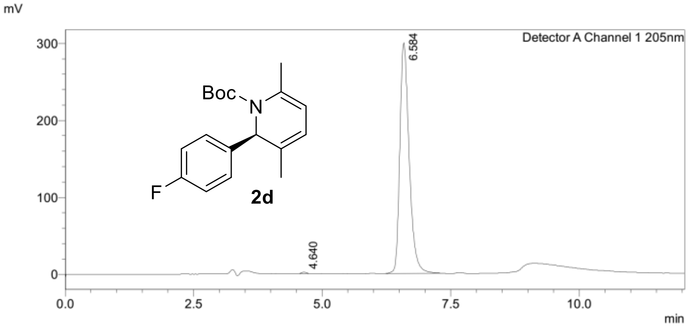

<Peak Table>

Detector A Channel 1 205nm

Peak#	Ret. Time	Area	Height	Conc.
1	4.640	12556	1991	0.330
2	6.584	3794112	299642	99.670
Total		3806667	301633	

SHIMADZU LabSolutions

Analysis Report

<Sample Information>

Sample Name : mbs-i-115-rac-adh-99.5-0.5-1.0
Sample ID :
Data Filename : mbs-i-115-rac-adh-99.5-0.5-1.0.lcd
Method Filename : zry-cl.lcm
Batch Filename :
Vial # : 1-1 Sample Type : Unknown
Injection Volume : 20 uL
Date Acquired : 2017/4/12 星期三 21:33:38 Acquired by : System Administrator
Date Processed : 2017/4/12 星期三 21:41:52 Processed by : System Administrator

<Chromatogram>

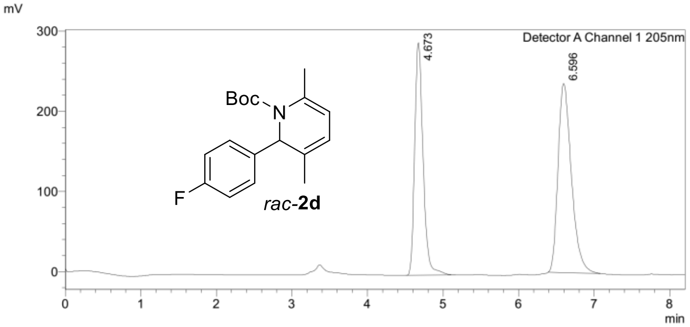

<Peak Table>

Detector A Channel 1 205nm

Peak#	Ret. Time	Area	Height	Conc.
1	4.673	2247829	289538	43.690
2	6.596	2897176	235277	56.310
Total		5145005	524816	

Supplementary Figure 173. HPLC analysis for compound **2d**

SHIMADZU LabSolutions

Analysis Report

<Sample Information>

Sample Name	: mbs-i-73-s-adh-99.5-0.5-1.0		
Sample ID	:		
Data Filename	: mbs-i-73-s-adh-99.5-0.5-1.0.lcd		
Method Filename	: wyh-wb-17-rac-ADH-1.0-230-254.lcm		
Batch Filename	:		
Vial #	: 1-1	Sample Type	: Unknown
Injection Volume	: 20 uL		
Date Acquired	: 2017/3/14 星期二 16:13:37	Acquired by	: System Administrator
Date Processed	: 2017/3/14 星期二 16:31:01	Processed by	: System Administrator

<Chromatogram>

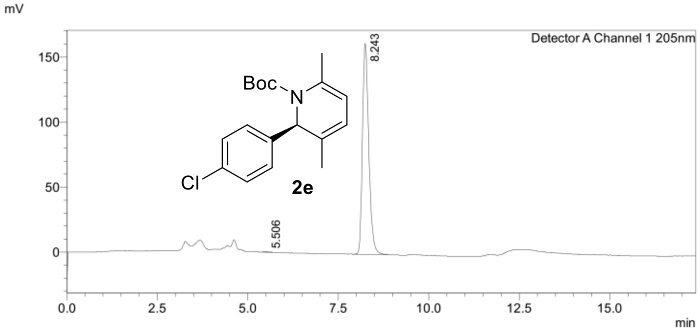

<Peak Table>

Detector A Channel 1 205nm

Peak#	Ret. Time	Area	Height	Conc.
1	5.506	3746	510	0.190
2	8.243	1970284	161810	99.810
Total		1974030	162320	

Analysis Report

<Sample Information>

Data Filename	: mbs-i-89-rac-adh-99.5-0.5-1.0.lcd		
Method Filename	: wyh-wb-17-rac-ADH-1.0-230-254.lcm		
Batch Filename	:		
Vial #	: 1-1	Sample Type	: Unknown
Injection Volume	: 20 uL		
Date Acquired	: 2017/3/14 15:42:17	Acquired by	: System Administrator
Date Processed	: 2017/3/16 20:34:53	Processed by	: System Administrator

<Chromatogram>

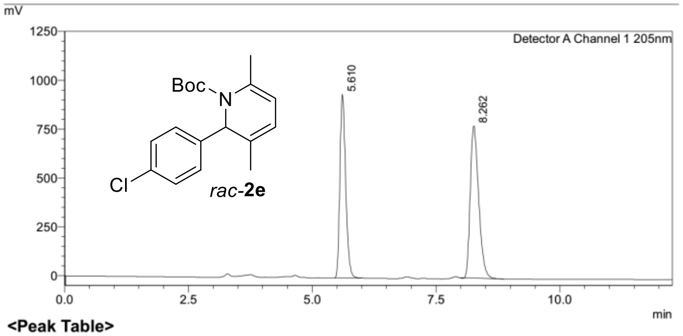

<Peak Table>

Detector A Channel 1 205nm

Peak#	Ret. Time	Area	Height	Conc.
1	5.610	7615724	935459	45.278
2	8.262	9204286	777515	54.722
Total		16820010	1712975	

Supplementary Figure 174. HPLC analysis for compound **2e**

SHIMADZU LabSolutions

Analysis Report

<Sample Information>

Sample Name	: mbs-i-101-s-adh-99.5-0.5-1.0		
Sample ID	:		
Data Filename	: mbs-i-101-s-adh-99.5-0.5-1.1.lcd		
Method Filename	: 20170113.lcm		
Batch Filename	:		
Vial #	: 1-1	Sample Type	: Unknown
Injection Volume	: 20 uL		
Date Acquired	: 2017/3/24 星期五 11:55:03	Acquired by	: System Administrator
Date Processed	: 2017/3/24 星期五 12:04:16	Processed by	: System Administrator

<Chromatogram>

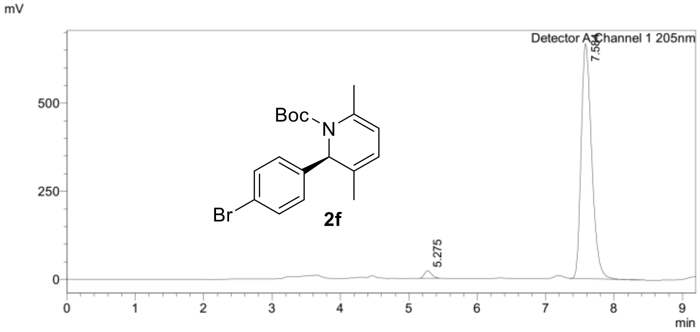

<Peak Table>

Detector A Channel 1 205nm

Peak#	Ret. Time	Area	Height	Conc.
1	5.275	155580	21304	2.138
2	7.584	7120190	666676	97.862
Total		7275770	687980	

Analysis Report

<Sample Information>

Data Filename	: mbs-i-102-rac-adh-99.5-0.5-1.1.lcd		
Method Filename	: 20170113.lcm		
Batch Filename	:		
Vial #	: 1-1	Sample Type	: Unknown
Injection Volume	: 20 uL		
Date Acquired	: 2017/3/24 11:28:08	Acquired by	: System Administrator
Date Processed	: 2017/5/29 11:37:55	Processed by	: System Administrator

<Chromatogram>

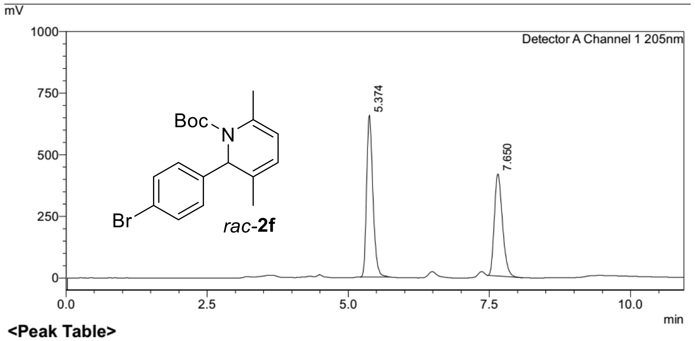

<Peak Table>

Detector A Channel 1 205nm

Peak#	Ret. Time	Area	Height	Conc.
1	5.374	5100057	656723	55.031
2	7.650	4167478	415008	44.969
Total		9267535	1071731	

Supplementary Figure 175. HPLC analysis for compound **2f**

SHIMADZU LabSolutions Analysis Report

<Sample Information>

Sample Name	: mbs-i-106-s-adh-99.5-0.5-0.5-column		
Sample ID	:		
Data Filename	: mbs-i-106-s-adh-99.5-0.5-0.5-column.lcd		
Method Filename	: zry-cl.lcm		
Batch Filename	:		
Vial #	: 1-1	Sample Type	: Unknown
Injection Volume	: 20 uL		
Date Acquired	: 2017/4/12 星期三 22:21:55	Acquired by	: System Administrator
Date Processed	: 2017/4/12 星期三 22:35:39	Processed by	: System Administrator

<Chromatogram>

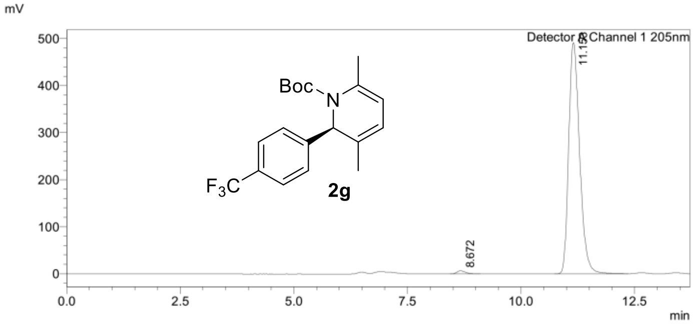

<Peak Table>

Detector A Channel 1 205nm

Peak#	Ret. Time	Area	Height	Conc.
1	8.672	88597	6677	1.065
2	11.153	8227099	491571	98.935
Total		8315696	498249	

SHIMADZU LabSolutions Analysis Report

<Sample Information>

Sample Name	: mbs-i-107-rac-adh-99.5-0.5-0.5-2nd		
Sample ID	:		
Data Filename	: mbs-i-107-rac-adh-99.5-0.5-0.5-2nd1.lcd		
Method Filename	: 20170113.lcm		
Batch Filename	:		
Vial #	: 1-1	Sample Type	: Unknown
Injection Volume	: 20 uL		
Date Acquired	: 2017/3/24 星期五 14:56:58	Acquired by	: System Administrator
Date Processed	: 2017/3/24 星期五 15:10:11	Processed by	: System Administrator

<Chromatogram>

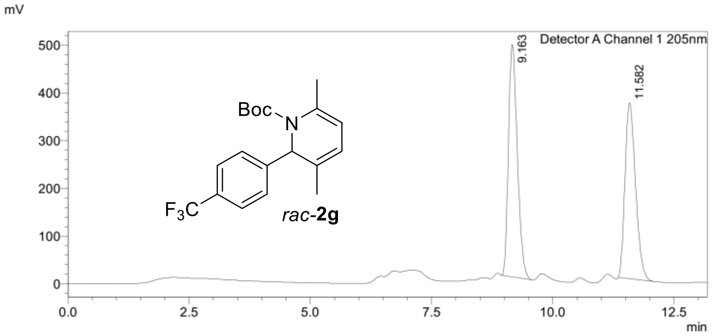

<Peak Table>

Detector A Channel 1 205nm

Peak#	Ret. Time	Area	Height	Conc.
1	9.163	6180519	486616	52.553
2	11.582	5579972	368346	47.447
Total		11760492	854961	

Supplementary Figure 176. HPLC analysis for compound **2g**

SHIMADZU LabSolutions

Analysis Report

<Sample Information>

Sample Name	: mbs-i-84-s-adh-99.5-0.5-1.0		
Sample ID	:		
Data Filename	: mbs-i-84-s-adh-99.5-0.5-1.0.lcd		
Method Filename	: wyh-wb-17-rac-ADH-1.0-230-254.lcm		
Batch Filename	:		
Vial #	: 1-1	Sample Type	: Unknown
Injection Volume	: 20 uL		
Date Acquired	: 2017/3/14 星期二 21:11:34	Acquired by	: System Administrator
Date Processed	: 2017/3/16 星期四 20:45:37	Processed by	: System Administrator

<Chromatogram>

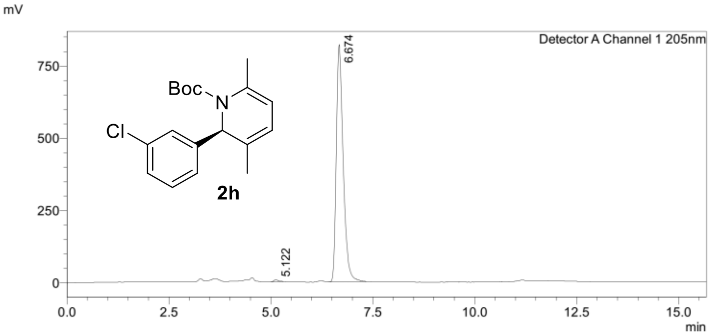

<Peak Table>

Detector A Channel 1 205nm

Peak#	Ret. Time	Area	Height	Conc.
1	5.122	48006	6562	0.494
2	6.674	9674489	819170	99.506
Total		9722494	825732	

SHIMADZU LabSolutions

Analysis Report

<Sample Information>

Sample Name	: mbs-i-85-rac-adh-99.5-0.5-1.0		
Sample ID	:		
Data Filename	: mbs-i-85-rac-adh-99.5-0.5-1.0.lcd		
Method Filename	: wyh-wb-17-rac-ADH-1.0-230-254.lcm		
Batch Filename	:		
Vial #	: 1-1	Sample Type	: Unknown
Injection Volume	: 20 uL		
Date Acquired	: 2017/3/14 星期二 17:21:51	Acquired by	: System Administrator
Date Processed	: 2017/3/16 星期四 20:46:13	Processed by	: System Administrator

<Chromatogram>

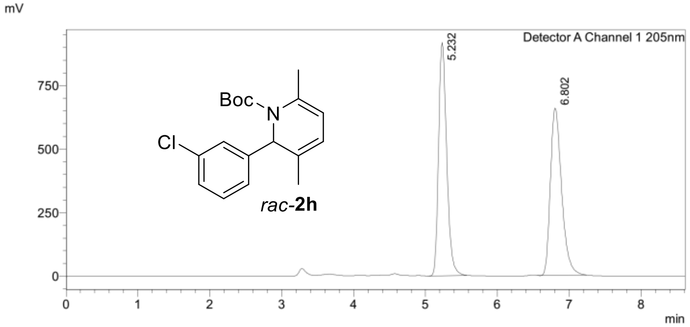

<Peak Table>

Detector A Channel 1 205nm

Peak#	Ret. Time	Area	Height	Conc.
1	5.232	7191310	916694	50.193
2	6.802	7135971	657542	49.807
Total		14327281	1574236	

Supplementary Figure 177. HPLC analysis for compound **2h**

SHIMADZU LabSolutions Analysis Report

<Sample Information>

Sample Name	: mbs-i-86-s-adh-99.5-0.5-0.5		
Sample ID	:		
Data Filename	: mbs-i-86-s-adh-99.5-0.5-0.5.lcd		
Method Filename	: zry-cl.lcm		
Batch Filename	:		
Vial #	: 1-1	Sample Type	: Unknown
Injection Volume	: 20 uL		
Date Acquired	: 2017/3/15 星期三 20:58:32	Acquired by	: System Administrator
Date Processed	: 2017/3/16 星期四 20:47:11	Processed by	: System Administrator

<Chromatogram>

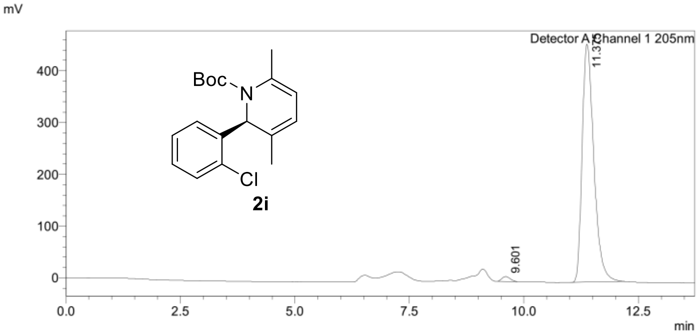

<Peak Table>

Detector A Channel 1 205nm

Peak#	Ret. Time	Area	Height	Conc.
1	9.601	115587	9337	1.397
2	11.375	8157719	459509	98.603
Total		8273306	468846	

SHIMADZU LabSolutions Analysis Report

<Sample Information>

Sample Name	: mbs-i-87-rac-adh-99.5-0.5-0.5		
Sample ID	:		
Data Filename	: mbs-i-87-rac-adh-99.5-0.5-0.5.lcd		
Method Filename	: zry-cl.lcm		
Batch Filename	:		
Vial #	: 1-1	Sample Type	: Unknown
Injection Volume	: 20 uL		
Date Acquired	: 2017/3/15 星期三 20:35:24	Acquired by	: System Administrator
Date Processed	: 2017/3/16 星期四 20:48:10	Processed by	: System Administrator

<Chromatogram>

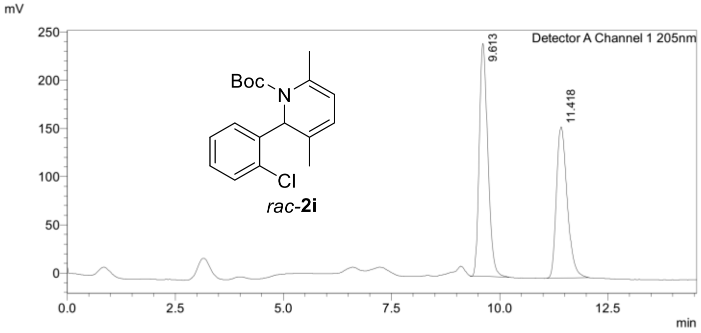

<Peak Table>

Detector A Channel 1 205nm

Peak#	Ret. Time	Area	Height	Conc.
1	9.613	3351125	241114	55.145
2	11.418	2725782	156729	44.855
Total		6076907	397843	

Supplementary Figure 178. HPLC analysis for compound **2i**

SHIMADZU LabSolutions

Analysis Report

<Sample Information>

Sample Name	: mbs-i-121-s-adh-99.5-0.5-1.0		
Sample ID	:		
Data Filename	: mbs-i-121-s-adh-99.5-0.5-1.0.lcd		
Method Filename	: zry-cl.lcm		
Batch Filename	:		
Vial #	: 1-1	Sample Type	: Unknown
Injection Volume	: 20 uL		
Date Acquired	: 2017/4/12 星期三 20:21:01	Acquired by	: System Administrator
Date Processed	: 2017/4/12 星期三 20:33:19	Processed by	: System Administrator

<Chromatogram>

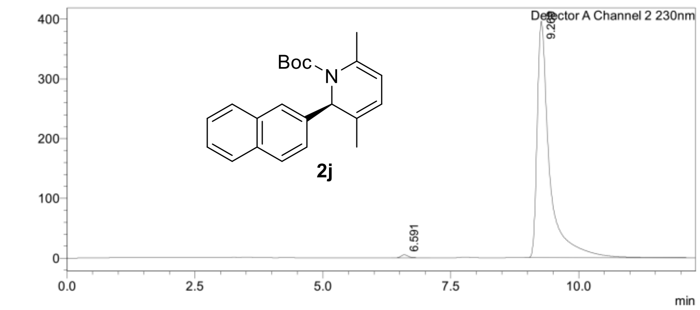

<Peak Table>

Detector A Channel 2 230nm

Peak#	Ret. Time	Area	Height	Conc.
1	6.591	51308	5293	0.755
2	9.269	6742312	395749	99.245
Total		6793619	401042	

SHIMADZU LabSolutions

Analysis Report

<Sample Information>

Sample Name	: mbs-i-122-rac-adh-99.5-0.5-1.0		
Sample ID	:		
Data Filename	: mbs-i-122-rac-adh-99.5-0.5-1.0.lcd		
Method Filename	: zry-cl.lcm		
Batch Filename	:		
Vial #	: 1-1	Sample Type	: Unknown
Injection Volume	: 20 uL		
Date Acquired	: 2017/4/12 星期三 17:17:30	Acquired by	: System Administrator
Date Processed	: 2017/4/12 星期三 17:28:29	Processed by	: System Administrator

<Chromatogram>

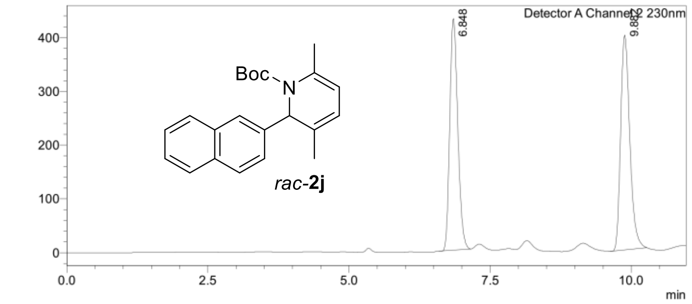

<Peak Table>

Detector A Channel 2 230nm

Peak#	Ret. Time	Area	Height	Conc.
1	6.848	4237602	429975	48.587
2	9.882	4483997	399314	51.413
Total		8721599	829290	

Supplementary Figure 179. HPLC analysis for compound **2j**

SHIMADZU LabSolutions Analysis Report

<Sample Information>

Sample Name : mbs-i-137-s-99.5-0.5-1.0-2nd
Sample ID :
Data Filename : mbs-i-137-s-adh-99.5-0.5-1.0-2nd1.lcd
Method Filename : wyh-wb-17-rac-ADH-1.0-230-254.lcm
Batch Filename :
Vial # : 1-1 Sample Type : Unknown
Injection Volume : 20 uL
Date Acquired : 2017/5/29 星期一 21:16:20 Acquired by : System Administrator
Date Processed : 2017/5/29 星期一 21:25:24 Processed by : System Administrator

<Chromatogram>

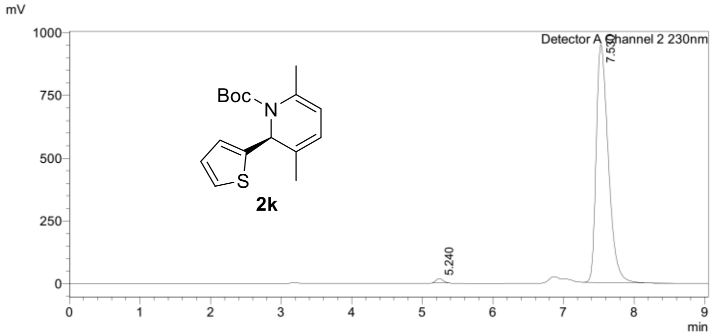

<Peak Table>

Detector A Channel 2 230nm

Peak#	Ret. Time	Area	Height	Conc.
1	5.240	122674	17127	1.067
2	7.530	11378104	948096	98.933
Total		11500778	965223	

SHIMADZU LabSolutions Analysis Report

<Sample Information>

Sample Name : mbs-i-138-rac-99.5-0.5-1.0
Sample ID :
Data Filename : mbs-i-138-rac-adh-99.5-0.5-1.1.lcd
Method Filename : wyh-wb-17-rac-ADH-1.0-230-254.lcm
Batch Filename :
Vial # : 1-1 Sample Type : Unknown
Injection Volume : 20 uL
Date Acquired : 2017/5/29 星期一 20:16:26 Acquired by : System Administrator
Date Processed : 2017/5/29 星期一 20:25:45 Processed by : System Administrator

<Chromatogram>

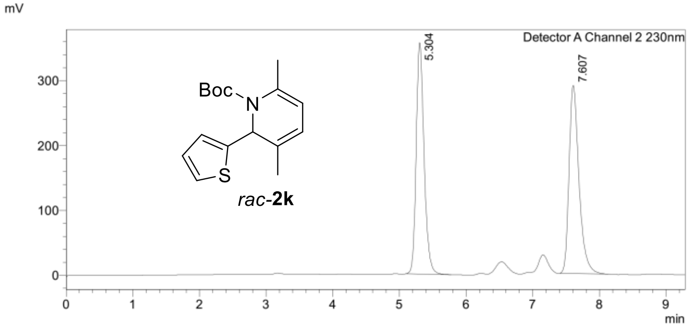

<Peak Table>

Detector A Channel 2 230nm

Peak#	Ret. Time	Area	Height	Conc.
1	5.304	2926500	356498	49.363
2	7.607	3002051	289913	50.637
Total		5928551	646411	

Supplementary Figure 180. HPLC analysis for compound **2k**

SHIMADZU LabSolutions Analysis Report

<Sample Information>

Sample Name	: mbs-i-119-s-adh-99.5-0.5-1.0-2nd		
Sample ID	:		
Data Filename	: mbs-i-119-s-adh-99.5-0.5-1.0-2nd.lcd		
Method Filename	: zry-cl.lcm		
Batch Filename	:		
Vial #	: 1-1	Sample Type	: Unknown
Injection Volume	: 20 uL		
Date Acquired	: 2017/4/12 星期三 16:09:37	Acquired by	: System Administrator
Date Processed	: 2017/4/12 星期三 16:19:32	Processed by	: System Administrator

<Chromatogram>

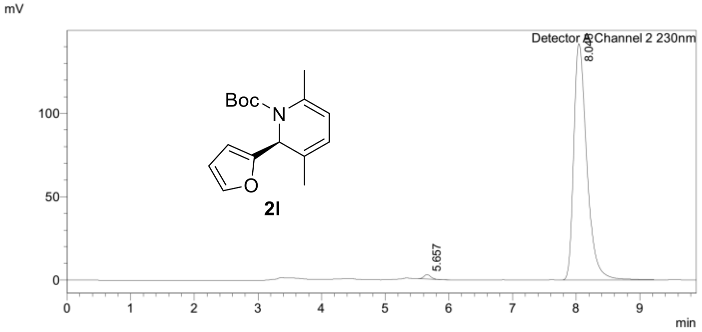

<Peak Table>

Detector A Channel 2 230nm

Peak#	Ret. Time	Area	Height	Conc.
1	5.657	19042	2593	0.930
2	8.045	2028641	141760	99.070
Total		2047683	144353	

SHIMADZU LabSolutions Analysis Report

<Sample Information>

Sample Name	: mbs-i-120-rac-adh-99.5-0.5-1.0-10th		
Sample ID	:		
Data Filename	: mbs-i-120-rac-adh-99.5-0.5-1.0-10th.lcd		
Method Filename	: zry-cl.lcm		
Batch Filename	:		
Vial #	: 1-1	Sample Type	: Unknown
Injection Volume	: 20 uL		
Date Acquired	: 2017/4/12 星期三 15:55:06	Acquired by	: System Administrator
Date Processed	: 2017/4/12 星期三 16:04:10	Processed by	: System Administrator

<Chromatogram>

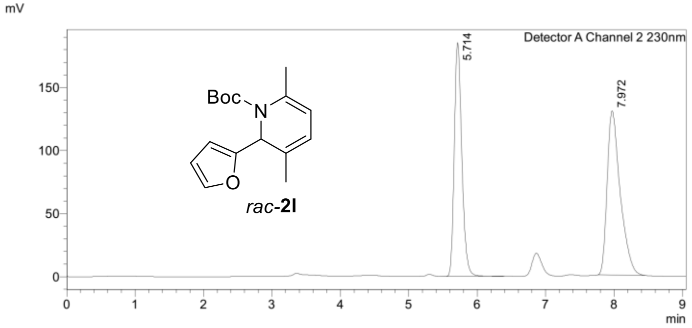

<Peak Table>

Detector A Channel 2 230nm

Peak#	Ret. Time	Area	Height	Conc.
1	5.714	1419479	185234	45.206
2	7.972	1720510	130068	54.794
Total		3139989	315302	

Supplementary Figure 181. HPLC analysis for compound **2l**

Analysis Report

<Sample Information>

Data Filename	: mbs-i-145-s-ayh-99.5-0.5-1.1.lcd		
Method Filename	: wa-1.0.lcm		
Batch Filename	:		
Vial #	: 1-1	Sample Type	: Unknown
Injection Volume	: 20 uL		
Date Acquired	: 2017/6/5 星期一 21:48:03	Acquired by	: System Administrator
Date Processed	: 2017/6/5 星期一 22:01:43	Processed by	: System Administrator

<Chromatogram>

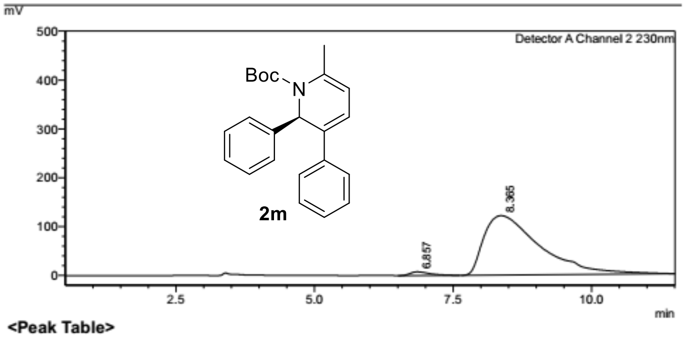

<Peak Table>

Detector A Channel 2 230nm

Peak#	Ret. Time	Area	Height	Conc.
1	6.857	185784	7549	2.105
2	8.365	8641226	121557	97.895
Total		8827010	129106	

Analysis Report

<Sample Information>

Data Filename	: mbs-i-146-rac-ayh-99.5-0.5-1.1.lcd		
Method Filename	: wa-1.0.lcm		
Batch Filename	:		
Vial #	: 1-1	Sample Type	: Unknown
Injection Volume	: 20 uL		
Date Acquired	: 2017/6/5 星期一 20:32:34	Acquired by	: System Administrator
Date Processed	: 2017/6/5 星期一 20:47:38	Processed by	: System Administrator

<Chromatogram>

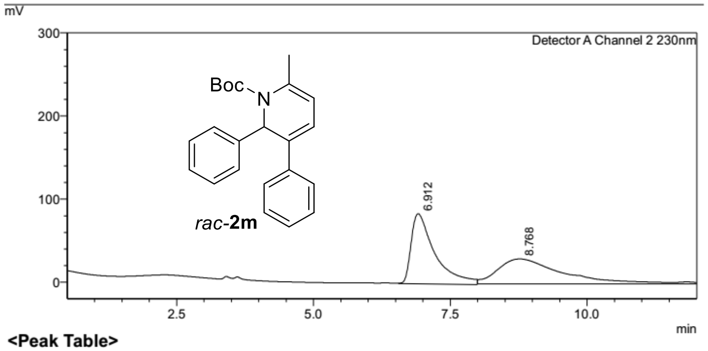

<Peak Table>

Detector A Channel 2 230nm

Peak#	Ret. Time	Area	Height	Conc.
1	6.912	2549215	84426	49.866
2	8.768	2562918	29958	50.134
Total		5112133	114384	

Supplementary Figure 182. HPLC analysis for compound **2m**

Analysis Report

<Sample Information>

Data Filename	: mbs-ii-46-s-odh-99.5-0.5-1.1.lcd		
Method Filename	: wa-1.0.lcm		
Batch Filename	:		
Vial #	: 1-1	Sample Type	: Unknown
Injection Volume	: 20 uL		
Date Acquired	: 2017/6/3 星期六 11:09:35	Acquired by	: System Administrator
Date Processed	: 2017/6/3 星期六 11:20:40	Processed by	: System Administrator

<Chromatogram>

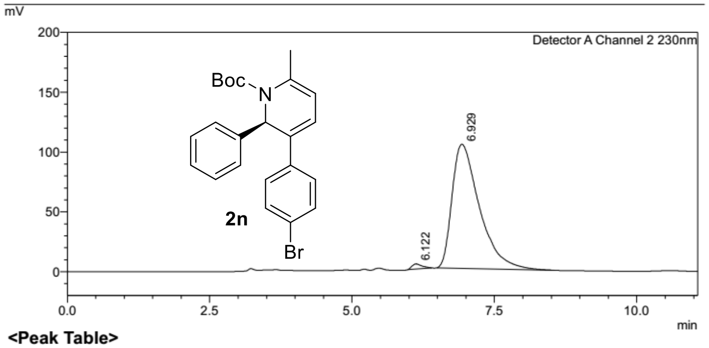

<Peak Table>

Detector A Channel 2 230nm

Peak#	Ret. Time	Area	Height	Conc.
1	6.122	53648	4351	1.518
2	6.929	3480509	103779	98.482
Total		3534157	108130	

Analysis Report

<Sample Information>

Data Filename	: mbs-ii-47-rac-odh-99.5-0.5-1.0-2nd1.lcd		
Method Filename	: wa-1.0.lcm		
Batch Filename	:		
Vial #	: 1-1	Sample Type	: Unknown
Injection Volume	: 20 uL		
Date Acquired	: 2017/6/3 星期六 10:18:55	Acquired by	: System Administrator
Date Processed	: 2017/6/3 星期六 10:29:16	Processed by	: System Administrator

<Chromatogram>

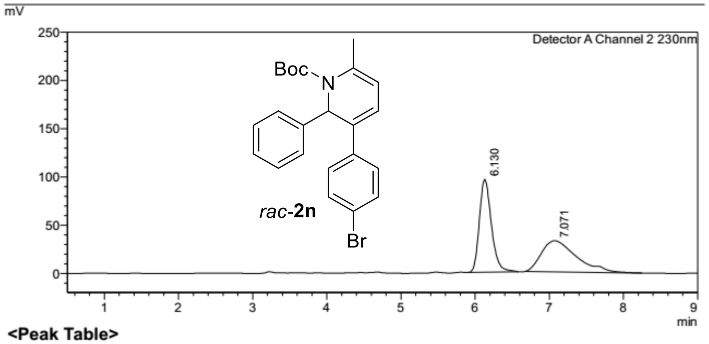

<Peak Table>

Detector A Channel 2 230nm

Peak#	Ret. Time	Area	Height	Conc.
1	6.130	1085506	96073	50.352
2	7.071	1070309	32311	49.648
Total		2155814	128384	

Supplementary Figure 183. HPLC analysis for compound **2n**

Analysis Report

<Sample Information>

Data Filename	: mbs-ii-42-s-ash-99.5-0.5-1.0-2nd1.lcd		
Method Filename	: wa-1.0.lcm		
Batch Filename	:		
Vial #	: 1-1	Sample Type	: Unknown
Injection Volume	: 20 uL		
Date Acquired	: 2017/6/5 星期一 15:58:13	Acquired by	: System Administrator
Date Processed	: 2017/6/5 星期一 16:08:10	Processed by	: System Administrator

<Chromatogram>

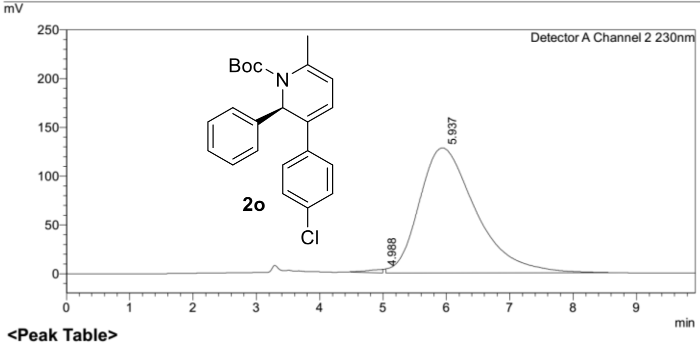

<Peak Table>

Detector A Channel 2 230nm

Peak#	Ret. Time	Area	Height	Conc.
1	4.988	55680	3620	0.693
2	5.937	7982704	128282	99.307
Total		8038383	131902	

Analysis Report

<Sample Information>

Data Filename	: mbs-ii-43-rac-ash-99.5-0.5-1.1.lcd		
Method Filename	: wa-1.0.lcm		
Batch Filename	:		
Vial #	: 1-1	Sample Type	: Unknown
Injection Volume	: 20 uL		
Date Acquired	: 2017/6/5 星期一 11:58:36	Acquired by	: System Administrator
Date Processed	: 2017/6/5 星期一 12:32:45	Processed by	: System Administrator

<Chromatogram>

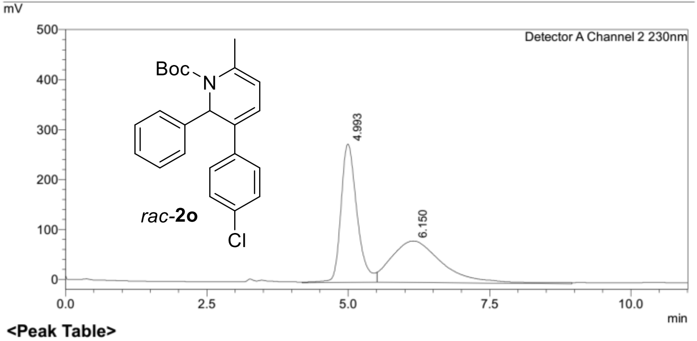

<Peak Table>

Detector A Channel 2 230nm

Peak#	Ret. Time	Area	Height	Conc.
1	4.993	5500021	276827	51.476
2	6.150	5184508	82806	48.524
Total		10684529	359633	

Supplementary Figure 184. HPLC analysis for compound **2o**

Analysis Report

SHIMADZU LabSolutions

<Sample Information>

Sample Name	: mbs-ii-58-s-ayh-99.5-0.5-1.0		
Sample ID	:		
Data Filename	: mbs-ii-58-s-ayh-99.5-0.5-1.1.lcd		
Method Filename	: dpg-OZH-95-5-1.0-230.lcm		
Batch Filename	:		
Vial #	: 1-1	Sample Type	: Unknown
Injection Volume	: 20 uL		
Date Acquired	: 2017/11/2 20:40:54	Acquired by	: System Administrator
Date Processed	: 2017/11/2 20:52:25	Processed by	: System Administrator

<Chromatogram>

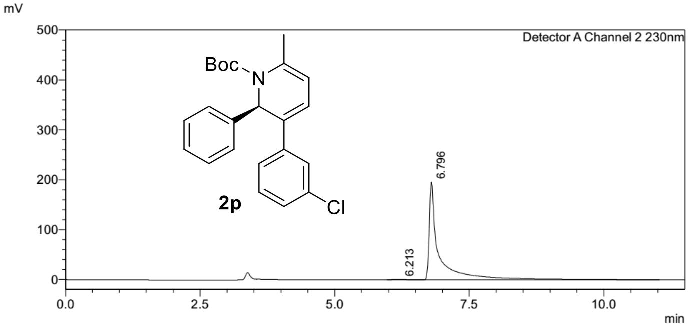

<Peak Table>

Detector A Channel 2 230nm

Peak#	Ret. Time	Area	Height	Conc.	Unit	Mark	Name
1	6.213	11875	643	0.464		M	
2	6.796	2546629	195437	99.536		M	
Total		2558504	196080				

Analysis Report

SHIMADZU LabSolutions

<Sample Information>

Sample Name	: mbs-ii-59-rac-ayh-99.5-0.5-1.0-2nd		
Sample ID	:		
Data Filename	: mbs-ii-59-rac-ayh-99.5-0.5-1.0-2nd1.lcd		
Method Filename	: dpg-OZH-95-5-1.0-230.lcm		
Batch Filename	:		
Vial #	: 1-1	Sample Type	: Unknown
Injection Volume	: 20 uL		
Date Acquired	: 2017/11/2 20:04:01	Acquired by	: System Administrator
Date Processed	: 2017/11/2 20:13:33	Processed by	: System Administrator

<Chromatogram>

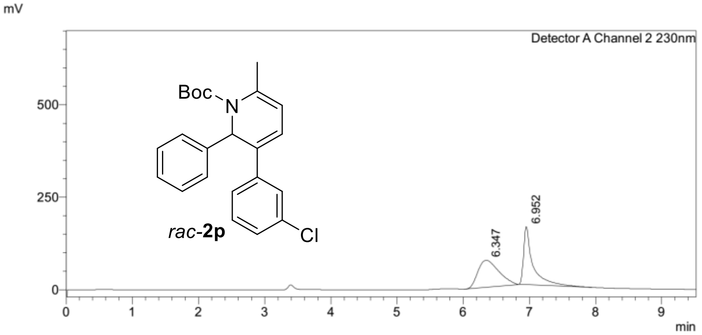

<Peak Table>

Detector A Channel 2 230nm

Peak#	Ret. Time	Area	Height	Conc.	Unit	Mark	Name
1	6.347	1600873	72820	51.129		M	
2	6.952	1530204	156923	48.871		M	
Total		3131077	229742				

Supplementary Figure 185. HPLC analysis for compound **2p**

Analysis Report

<Sample Information>

Data Filename	: mbs-ii-54-s-ayh-97-3-0.5-3rd1.lcd		
Method Filename	: wa-1.0.lcm		
Batch Filename	:		
Vial #	: 1-1	Sample Type	: Unknown
Injection Volume	: 20 uL		
Date Acquired	: 2017/6/8 19:13:03	Acquired by	: System Administrator
Date Processed	: 2018/1/17 16:14:09	Processed by	: System Administrator

<Chromatogram>

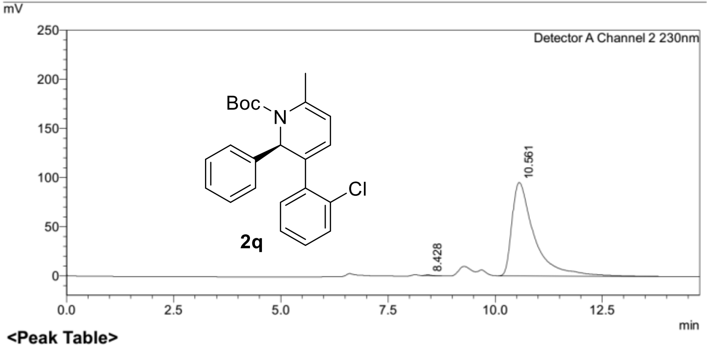

<Peak Table>

Detector A Channel 2 230nm

Peak#	Ret. Time	Area	Height	Conc.
1	8.428	5685	685	0.164
2	10.561	3461889	95103	99.836
Total		3467574	95788	

Analysis Report

<Sample Information>

Data Filename	: mbs-ii-55-rac-ayh-97-3-0.6.lcd		
Method Filename	: wa-1.0.lcm		
Batch Filename	:		
Vial #	: 1-1	Sample Type	: Unknown
Injection Volume	: 20 uL		
Date Acquired	: 2017/6/8 16:43:50	Acquired by	: System Administrator
Date Processed	: 2017/11/6 22:07:19	Processed by	: System Administrator

<Chromatogram>

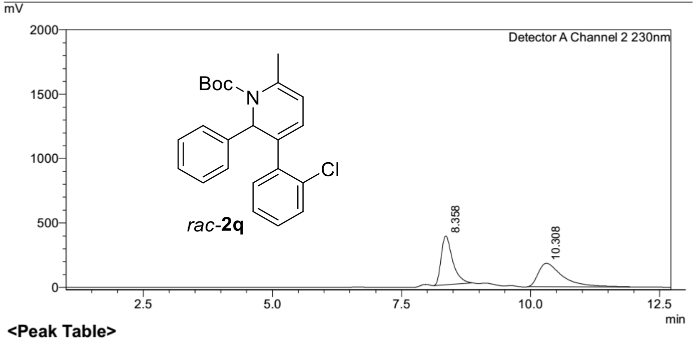

<Peak Table>

Detector A Channel 2 230nm

Peak#	Ret. Time	Area	Height	Conc.
1	8.358	5883548	380305	49.758
2	10.308	5940792	181532	50.242
Total		11824340	561837	

Supplementary Figure 186. HPLC analysis for compound **2q**

Analysis Report

<Sample Information>

Data Filename	: mbs-ii-15-s-adh-99.5-0.5-1.1.lcd		
Method Filename	: wa-1.0.lcm		
Batch Filename	:		
Vial #	: 1-1	Sample Type	: Unknown
Injection Volume	: 20 uL		
Date Acquired	: 2017/6/3 星期六 21:44:05	Acquired by	: System Administrator
Date Processed	: 2017/9/14 星期四 17:26:26	Processed by	: System Administrator

<Chromatogram>

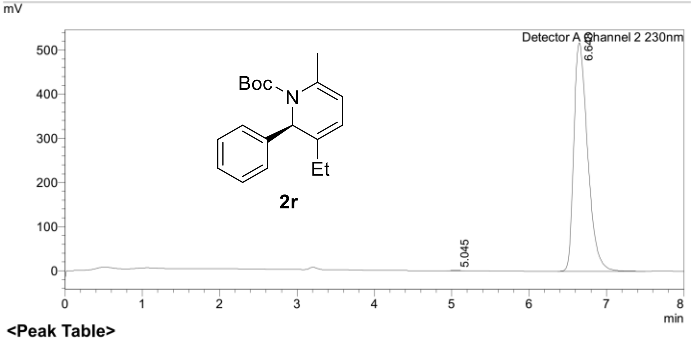

<Peak Table>

Detector A Channel 2 230nm

Peak#	Ret. Time	Area	Height	Conc.
1	5.045	9257	1296	0.143
2	6.648	6442136	517735	99.857
Total		6451393	519031	

Analysis Report

<Sample Information>

Data Filename	: mbs-ii-16-rac-adh-99.5-0.5-1.1.lcd		
Method Filename	: wa-1.0.lcm		
Batch Filename	:		
Vial #	: 1-1	Sample Type	: Unknown
Injection Volume	: 20 uL		
Date Acquired	: 2017/6/3 星期六 21:11:53	Acquired by	: System Administrator
Date Processed	: 2017/6/3 星期六 21:19:28	Processed by	: System Administrator

<Chromatogram>

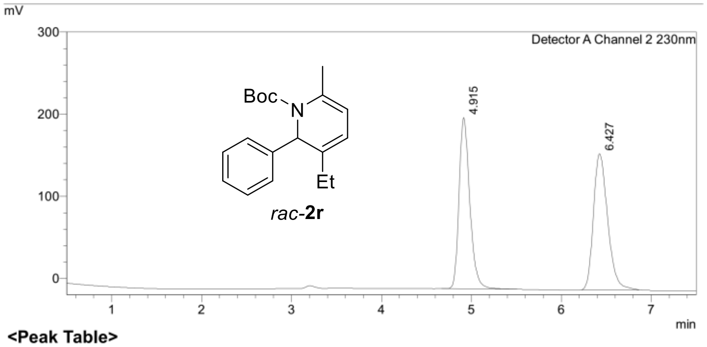

<Peak Table>

Detector A Channel 2 230nm

Peak#	Ret. Time	Area	Height	Conc.
1	4.915	1735974	208532	48.877
2	6.427	1815781	165764	51.123
Total		3551755	374297	

Supplementary Figure 187. HPLC analysis for compound **2r**

Analysis Report

<Sample Information>

Data Filename	: mbs-ii-06-s-adh-99.5-0.5-1.0-3rd1.lcd		
Method Filename	: wa-1.0.lcm		
Batch Filename	:		
Vial #	: 1-1	Sample Type	: Unknown
Injection Volume	: 20 uL		
Date Acquired	: 2017/6/5 星期一 9:25:40	Acquired by	: System Administrator
Date Processed	: 2017/6/5 星期一 9:41:26	Processed by	: System Administrator

<Chromatogram>

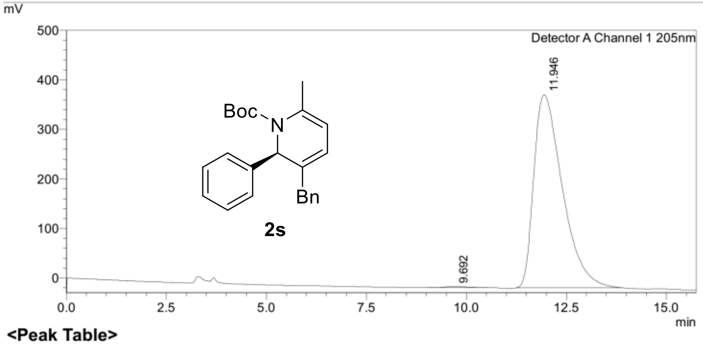

<Peak Table>

Detector A Channel 1 205nm

Peak#	Ret. Time	Area	Height	Conc.
1	9.692	75755	2289	0.388
2	11.946	19452609	390293	99.612
Total		19528364	392582	

Analysis Report

<Sample Information>

Data Filename	: mbs-ii-07-rac-adh-99.5-0.5-1.0-2nd1.lcd		
Method Filename	: wa-1.0.lcm		
Batch Filename	:		
Vial #	: 1-1	Sample Type	: Unknown
Injection Volume	: 20 uL		
Date Acquired	: 2017/6/5 星期一 9:50:25	Acquired by	: System Administrator
Date Processed	: 2017/6/5 星期一 10:05:44	Processed by	: System Administrator

<Chromatogram>

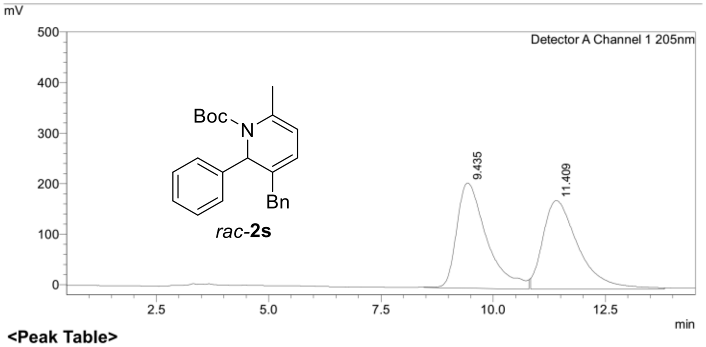

<Peak Table>

Detector A Channel 1 205nm

Peak#	Ret. Time	Area	Height	Conc.
1	9.435	9688093	208311	50.026
2	11.409	9678132	175204	49.974
Total		19366225	383515	

Supplementary Figure 188. HPLC analysis for compound **2s**

Analysis Report

<Sample Information>

Data Filename	: mbs-ii-33-s-adh-99.5-0.5-1.1.lcd		
Method Filename	: wa-1.0.lcm		
Batch Filename	:		
Vial #	: 1-1	Sample Type	: Unknown
Injection Volume	: 20 uL		
Date Acquired	: 2017/6/3 星期六 18:02:26	Acquired by	: System Administrator
Date Processed	: 2017/6/3 星期六 18:13:05	Processed by	: System Administrator

<Chromatogram>

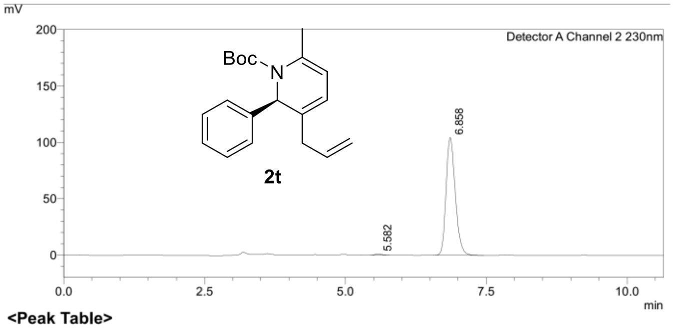

<Peak Table>

Detector A Channel 2 230nm

Peak#	Ret. Time	Area	Height	Conc.
1	5.582	7664	856	0.638
2	6.858	1194401	104503	99.362
Total		1202066	105359	

Analysis Report

<Sample Information>

Data Filename	: mbs-ii-34-rac-adh-99.5-0.5-1.1.lcd		
Method Filename	: wa-1.0.lcm		
Batch Filename	:		
Vial #	: 1-1	Sample Type	: Unknown
Injection Volume	: 20 uL		
Date Acquired	: 2017/6/3 星期六 17:34:47	Acquired by	: System Administrator
Date Processed	: 2017/6/3 星期六 17:44:36	Processed by	: System Administrator

<Chromatogram>

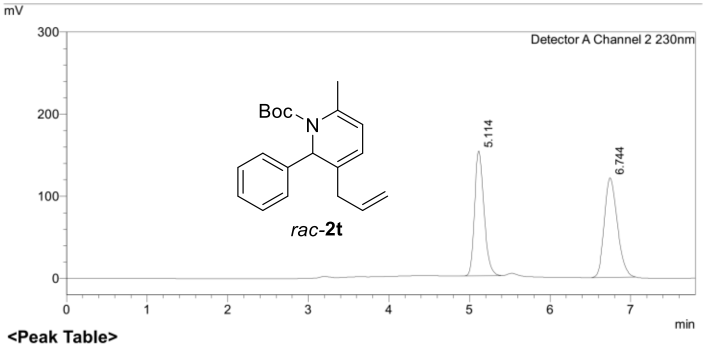

<Peak Table>

Detector A Channel 2 230nm

Peak#	Ret. Time	Area	Height	Conc.
1	5.114	1230293	151978	47.327
2	6.744	1369263	121199	52.673
Total		2599556	273177	

Supplementary Figure 189. HPLC analysis for compound **2t**

Analysis Report

<Sample Information>

Data Filename	: mbs-me-75-asy-adh-99.5-0.5-1.0ml4.lcd		
Method Filename	: dpg-OZH-95-5-1.0-230.lcm		
Batch Filename	:		
Vial #	: 1-1	Sample Type	: Unknown
Injection Volume	: 20 uL		
Date Acquired	: 2019/4/25 21:25:14	Acquired by	: System Administrator
Date Processed	: 2019/4/25 21:47:04	Processed by	: System Administrator

<Chromatogram>

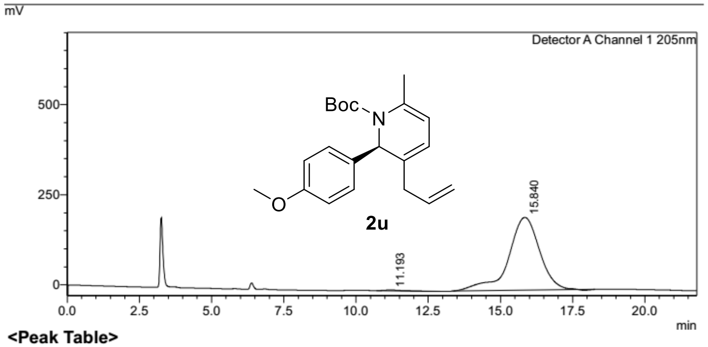

<Peak Table>

Detector A Channel 1 205nm

Peak#	Ret. Time	Area	Height	Conc.
1	11.193	107392	2344	0.702
2	15.840	15187304	201991	99.298
Total		15294696	204336	

Analysis Report

<Sample Information>

Data Filename	: mbs-me-77-rac-adh-99.5-0.5-1.0mllast1.lcd		
Method Filename	: dpg-OZH-95-5-1.0-230.lcm		
Batch Filename	:		
Vial #	: 1-1	Sample Type	: Unknown
Injection Volume	: 20 uL		
Date Acquired	: 2019/4/25 21:49:13	Acquired by	: System Administrator
Date Processed	: 2019/4/25 22:09:03	Processed by	: System Administrator

<Chromatogram>

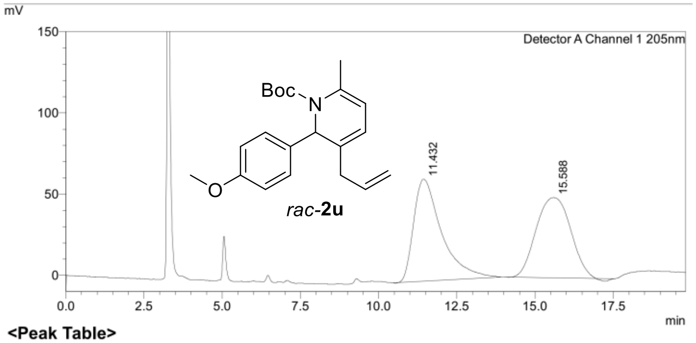

<Peak Table>

Detector A Channel 1 205nm

Peak#	Ret. Time	Area	Height	Conc.
1	11.432	3899179	62819	50.735
2	15.588	3786161	49374	49.265
Total		7685340	112193	

Supplementary Figure 190. HPLC analysis for compound **2u**

Analysis Report

<Sample Information>

Data Filename	: mbs-me-73-asy-adh-99.5-0.5-1.0 ml1.lcd		
Method Filename	: 20170113.lcm		
Batch Filename	:		
Vial #	: 1-1	Sample Type	: Unknown
Injection Volume	: 20 uL		
Date Acquired	: 2019/4/23 17:52:04	Acquired by	: System Administrator
Date Processed	: 2019/4/23 18:13:35	Processed by	: System Administrator

<Chromatogram>

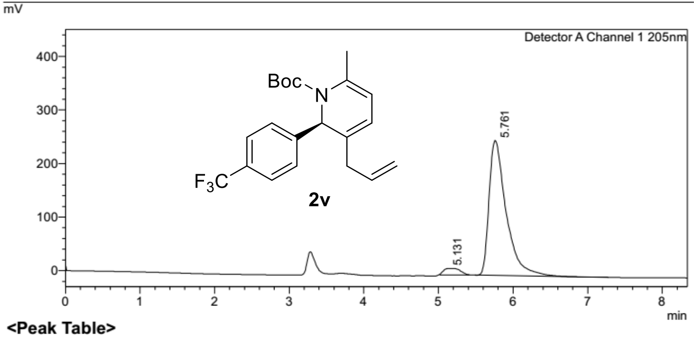

<Peak Table>

Detector A Channel 1 205nm

Peak#	Ret. Time	Area	Height	Conc.
1	5.131	187642	12165	4.455
2	5.761	4024386	251841	95.545
Total		4212028	264006	

Analysis Report

<Sample Information>

Data Filename	: mbs-me-71-rac-adh-99.5-0.5-1.0 ml5.lcd		
Method Filename	: 20170113.lcm		
Batch Filename	:		
Vial #	: 1-1	Sample Type	: Unknown
Injection Volume	: 20 uL		
Date Acquired	: 2019/4/23 17:20:14	Acquired by	: System Administrator
Date Processed	: 2019/4/23 17:28:44	Processed by	: System Administrator

<Chromatogram>

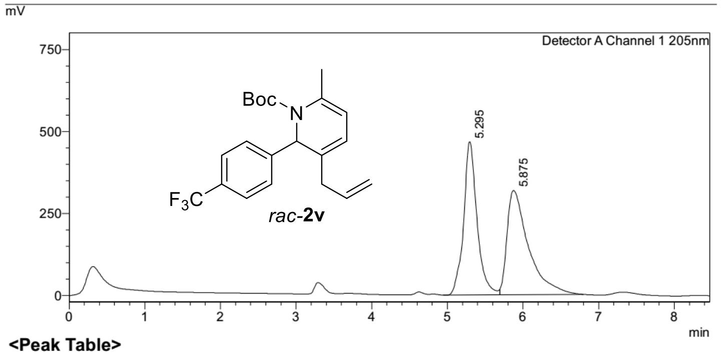

<Peak Table>

Detector A Channel 1 205nm

Peak#	Ret. Time	Area	Height	Conc.
1	5.295	5772305	466946	47.909
2	5.875	6276146	318393	52.091
Total		12048451	785340	

Supplementary Figure 191. HPLC analysis for compound **2v**

Analysis Report

<Sample Information>

Data Filename	: mbs-md-142-asy-adh-99.5-0.5-1.0ml1.lcd		
Method Filename	: WAC-93-FANFA.lcm		
Batch Filename	:		
Vial #	: 1-1	Sample Type	: Unknown
Injection Volume	: 20 uL		
Date Acquired	: 2019/3/9 15:06:29	Acquired by	: System Administrator
Date Processed	: 2019/3/9 16:22:59	Processed by	: System Administrator

<Chromatogram>

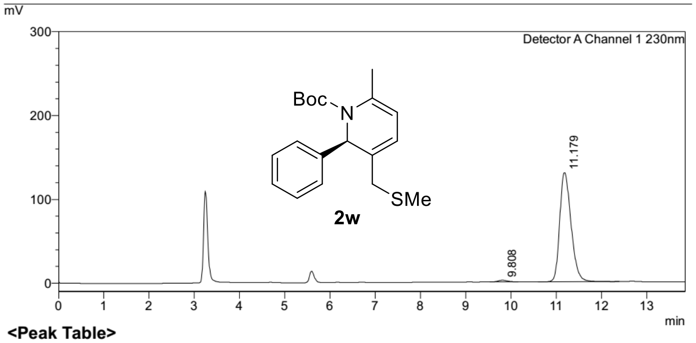

<Peak Table>

Detector A Channel 1 230nm

Peak#	Ret. Time	Area	Height	Conc.
1	9.808	28850	1994	1.253
2	11.179	2273578	130228	98.747
Total		2302429	132222	

Analysis Report

<Sample Information>

Data Filename	: mbs-md-144-rac-adh-99.5-0.5-1.0ml1.lcd		
Method Filename	: WAC-93-FANFA.lcm		
Batch Filename	:		
Vial #	: 1-1	Sample Type	: Unknown
Injection Volume	: 20 uL		
Date Acquired	: 2019/3/9 14:43:16	Acquired by	: System Administrator
Date Processed	: 2019/3/9 14:56:22	Processed by	: System Administrator

<Chromatogram>

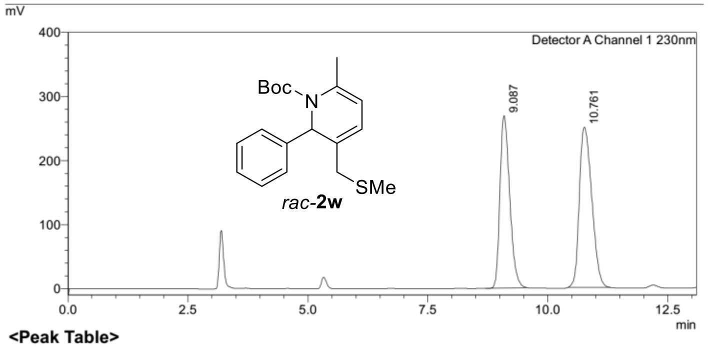

<Peak Table>

Detector A Channel 1 230nm

Peak#	Ret. Time	Area	Height	Conc.
1	9.087	3806094	268699	45.687
2	10.761	4524698	250245	54.313
Total		8330792	518945	

Supplementary Figure 192. HPLC analysis for compound **2w**

Analysis Report

<Sample Information>

Data Filename	: mbs-me-74-asy-adh-99.5-0.5-1.0 ml1.lcd		
Method Filename	: 20170113.lcm		
Batch Filename	:		
Vial #	: 1-1	Sample Type	: Unknown
Injection Volume	: 20 uL		
Date Acquired	: 2019/4/23 18:07:53	Acquired by	: System Administrator
Date Processed	: 2019/4/23 18:21:08	Processed by	: System Administrator

<Chromatogram>

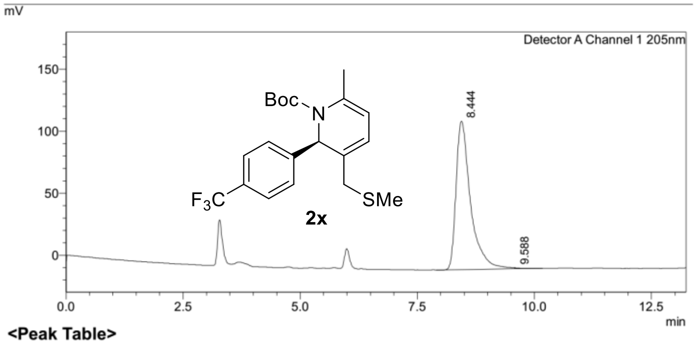

<Peak Table>

Detector A Channel 1 205nm

Peak#	Ret. Time	Area	Height	Conc.
1	8.444	2587183	119737	99.654
2	9.588	8978	850	0.346
Total		2596161	120586	

Analysis Report

<Sample Information>

Data Filename	: mbs-me-72-rac-adh-99.5-0.5-1.0 ml2.lcd		
Method Filename	: 20170113.lcm		
Batch Filename	:		
Vial #	: 1-1	Sample Type	: Unknown
Injection Volume	: 20 uL		
Date Acquired	: 2019/4/23 17:06:11	Acquired by	: System Administrator
Date Processed	: 2020/5/6 11:28:47	Processed by	: System Administrator

<Chromatogram>

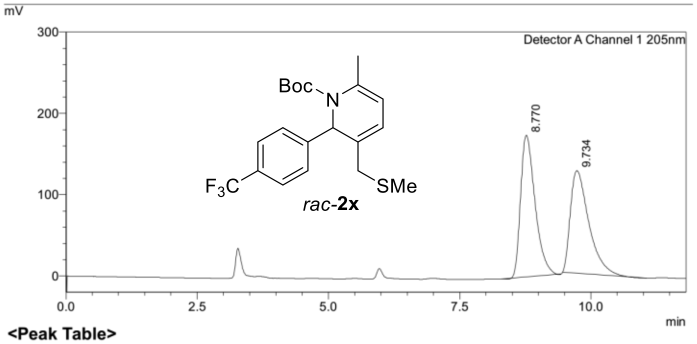

<Peak Table>

Detector A Channel 1 205nm

Peak#	Ret. Time	Area	Height	Conc.
1	8.770	3336237	174166	51.765
2	9.734	3108696	126274	48.235
Total		6444933	300440	

Supplementary Figure 193. HPLC analysis for compound **2x**

Analysis Report

<Sample Information>

Data Filename	: mbs-me-76-asy-ADH-9.5-0.5-1ml2.lcd		
Method Filename	: 205 and 254nm.lcm		
Batch Filename	:		
Vial #	: 1-1	Sample Type	: Unknown
Injection Volume	: 20 uL		
Date Acquired	: 2020/5/8 16:42:25	Acquired by	: System Administrator
Date Processed	: 2020/5/8 16:52:38	Processed by	: System Administrator

<Chromatogram>

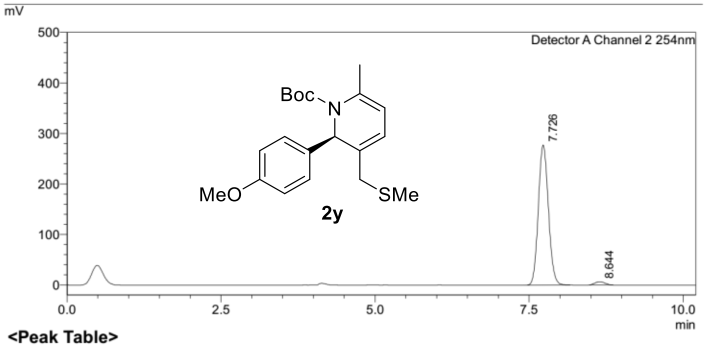

<Peak Table>

Detector A Channel 2 254nm

Peak#	Ret. Time	Area	Height	Conc.
1	7.726	3155397	277133	97.718
2	8.644	73691	6617	2.282
Total		3229088	283749	

Analysis Report

<Sample Information>

Data Filename	: mbs-me-76-rac-ADH-9.5-0.5-1ml1.lcd		
Method Filename	: 205 and 254nm.lcm		
Batch Filename	:		
Vial #	: 1-1	Sample Type	: Unknown
Injection Volume	: 20 uL		
Date Acquired	: 2020/5/8 16:31:35	Acquired by	: System Administrator
Date Processed	: 2020/5/8 16:41:17	Processed by	: System Administrator

<Chromatogram>

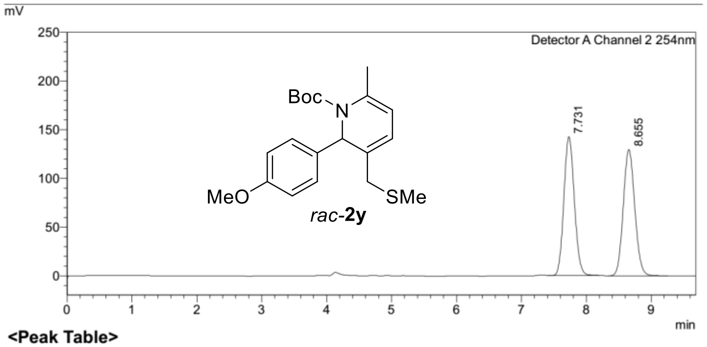

<Peak Table>

Detector A Channel 2 254nm

Peak#	Ret. Time	Area	Height	Conc.
1	7.731	1597656	142748	49.977
2	8.655	1599130	129895	50.023
Total		3196786	272644	

Supplementary Figure 194. HPLC analysis for compound **2y**

2019/5/21 9:12:46 Page 1 / 1

Analysis Report

<Sample Information>

Sample Name : cuy-cc-47-3-asy-adh-99.5-0.5-254-1.0
Sample ID :
Data Filename : cuy-cc-47-3-asy-adh-99.5-0.5-1.0-230.lcd
Method Filename : xsl-230-254-1.0.lcm
Batch Filename :
Vial # : 1-1 Sample Type : Unknown
Injection Volume : 20 uL
Date Acquired : 2019/5/17 9:47:36 Acquired by : System Administrator
Date Processed : 2019/5/17 12:33:20 Processed by : System Administrator

<Chromatogram>

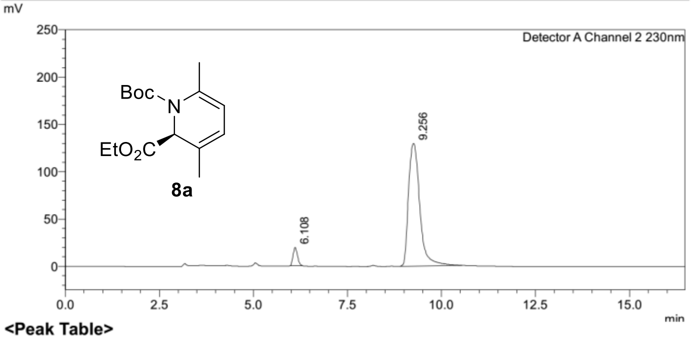

<Peak Table>

Detector A Channel 2 230nm

Peak#	Ret. Time	Area	Height	Conc.
1	6.108	156505	19301	5.485
2	9.256	2696775	129622	94.515
Total		2853280	148922	

D:\Data\Cui Xiaoyuan\cuy-cc-47-3-asy-adh-99.5-0.5-1.0-230.lcd

2019/5/21 13:09:38 Page 1 / 1

Analysis Report

<Sample Information>

Sample Name : cuy-cc-61-3-rac-adh-99.5-0.5-254-1.0
Sample ID :
Data Filename : cuy-cc-61-3-rac-adh-99.5-0.5-1.0-230.lcd
Method Filename : xsl-230-254-1.0.lcm
Batch Filename :
Vial # : 1-1 Sample Type : Unknown
Injection Volume : 20 uL
Date Acquired : 2019/5/17 10:12:36 Acquired by : System Administrator
Date Processed : 2019/5/21 13:08:41 Processed by : System Administrator

<Chromatogram>

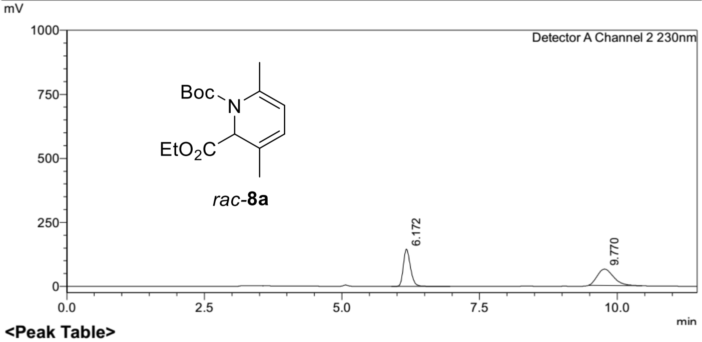

<Peak Table>

Detector A Channel 2 230nm

Peak#	Ret. Time	Area	Height	Conc.
1	6.172	1300275	144887	49.918
2	9.770	1304565	63379	50.082
Total		2604840	208266	

D:\Data\Cui Xiaoyuan\cuy-cc-61-3-rac-adh-99.5-0.5-1.0-230.lcd

Supplementary Figure 195. HPLC analysis for compound **8a**

Analysis Report

<Sample Information>

Sample Name	: cuy-cc-69-1-asy-ia-99.5-0.5-0.5-2		
Sample ID	:		
Data Filename	: cuy-cc-73-1-asy-ia-99.5-0.5-0.5-3.lcd		
Method Filename	: 20190312.lcm		
Batch Filename	:		
Vial #	: 1-1	Sample Type	: Unknown
Injection Volume	: 20 uL		
Date Acquired	: 2019/5/27 14:29:04	Acquired by	: System Administrator
Date Processed	: 2019/5/27 14:54:55	Processed by	: System Administrator

<Chromatogram>

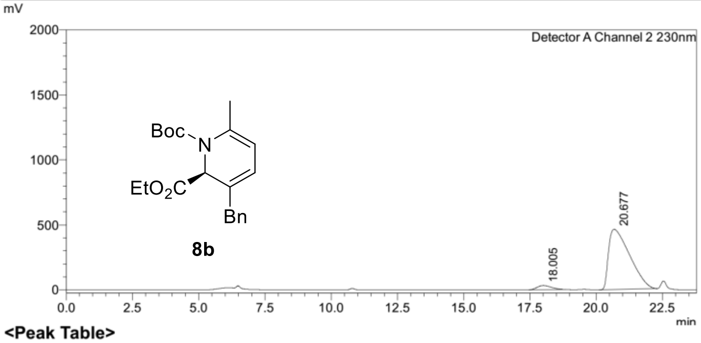

<Peak Table>

Detector A Channel 2 230nm

Peak#	Ret. Time	Area	Height	Conc.
1	18.005	1223311	32361	4.547
2	20.677	25679298	463353	95.453
Total		26902608	495713	

Analysis Report

<Sample Information>

Sample Name	: cuy-cc-76-1-asy-ia-99.5-0.5-0.5-2		
Sample ID	:		
Data Filename	: cuy-cc-76-1-rac-ia-99.5-0.5-0.5-3.lcd		
Method Filename	: 20190312.lcm		
Batch Filename	:		
Vial #	: 1-1	Sample Type	: Unknown
Injection Volume	: 20 uL		
Date Acquired	: 2019/5/27 14:04:24	Acquired by	: System Administrator
Date Processed	: 2019/5/27 14:54:58	Processed by	: System Administrator

<Chromatogram>

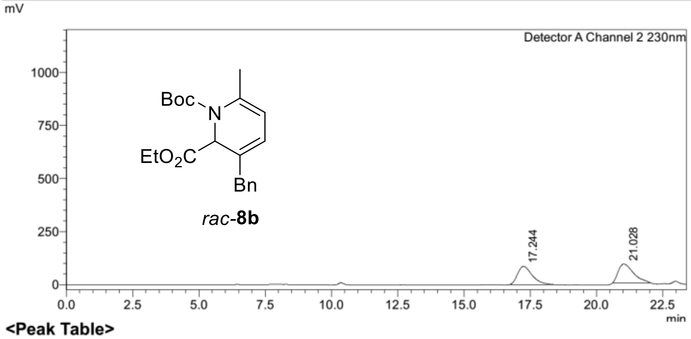

<Peak Table>

Detector A Channel 2 230nm

Peak#	Ret. Time	Area	Height	Conc.
1	17.244	3381993	86433	48.872
2	21.028	3538127	88582	51.128
Total		6920121	175015	

Supplementary Figure 196. HPLC analysis for compound **8b**

Analysis Report

<Sample Information>

Sample Name	: cuy-cc-87-2-asy-adh-99.5-0.5-0.8-2		
Sample ID	:		
Data Filename	: cuy-cc-87-2-asy-adh-99.5-0.5-0.8-3.lcd		
Method Filename	: 20190312.lcm		
Batch Filename	:		
Vial #	: 1-1	Sample Type	: Unknown
Injection Volume	: 20 uL		
Date Acquired	: 2019/6/3 19:40:03	Acquired by	: System Administrator
Date Processed	: 2019/6/3 19:57:36	Processed by	: System Administrator

<Chromatogram>

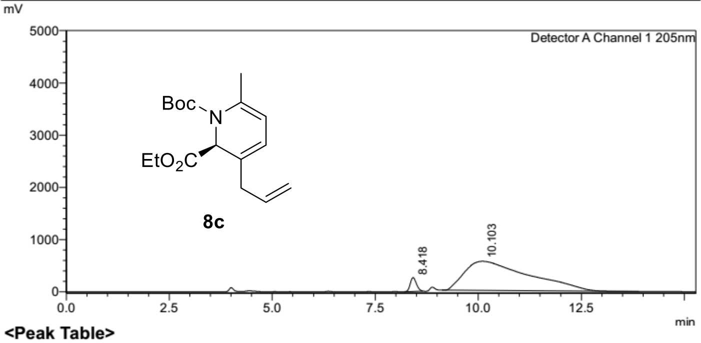

<Peak Table>

Detector A Channel 1 205nm

Peak#	Ret. Time	Area	Height	Conc.
1	8.418	2684818	263780	4.192
2	10.103	61358947	558823	95.808
Total		64043765	822602	

Analysis Report

<Sample Information>

Sample Name	: cuy-cc-87-1-rac-adh-99.5-0.5-0.8-2		
Sample ID	:		
Data Filename	: cuy-cc-87-1-rac-adh-99.5-0.5-0.8-3.lcd		
Method Filename	: 20190312.lcm		
Batch Filename	:		
Vial #	: 1-1	Sample Type	: Unknown
Injection Volume	: 20 uL		
Date Acquired	: 2019/6/3 19:23:38	Acquired by	: System Administrator
Date Processed	: 2019/6/3 19:57:33	Processed by	: System Administrator

<Chromatogram>

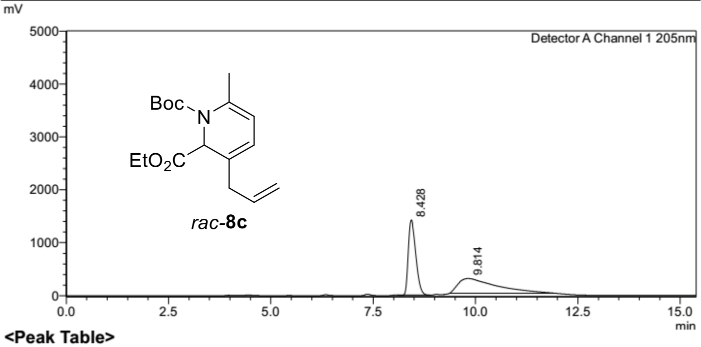

<Peak Table>

Detector A Channel 1 205nm

Peak#	Ret. Time	Area	Height	Conc.
1	8.428	18214410	1420660	49.213
2	9.814	18796910	280530	50.787
Total		37011320	1701190	

Supplementary Figure 197. HPLC analysis for compound **8c**

SHIMADZU LabSolutions Analysis Report

<Sample Information>

Sample Name : mbs-ii-87-s-adh-99.5-0.5-1.0-2nd
Sample ID :
Data Filename : mbs-ii-87-s-adh-99.5-0.5-1.0-2nd1.lcd
Method Filename : dpg-OZH-95-5-1.0-230.lcm
Batch Filename :
Vial # : 1-1 Sample Type : Unknown
Injection Volume : 20 uL
Date Acquired : 2017/11/2 14:25:09 Acquired by : System Administrator
Date Processed : 2017/11/2 14:43:22 Processed by : System Administrator

<Chromatogram>

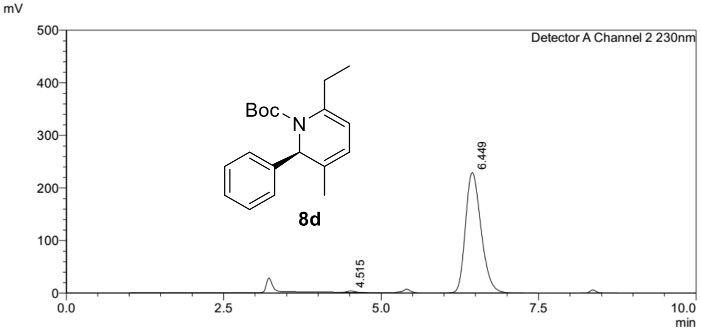

<Peak Table>

Detector A Channel 2 230nm

Peak#	Ret. Time	Area	Height	Conc.	Unit	Mark	Name
1	4.515	22495	2727	0.560		M	
2	6.449	3992067	229578	99.440		M	
Total		4014563	232305				

SHIMADZU LabSolutions Analysis Report

<Sample Information>

Sample Name : mbs-ii-103-rac-adh-99.5-0.5-1.0
Sample ID :
Data Filename : mbs-ii-103-rac-adh-99.5-0.5-1.2.lcd
Method Filename : dpg-OZH-95-5-1.0-230.lcm
Batch Filename :
Vial # : 1-1 Sample Type : Unknown
Injection Volume : 20 uL
Date Acquired : 2017/11/2 11:53:07 Acquired by : System Administrator
Date Processed : 2017/11/2 14:04:06 Processed by : System Administrator

<Chromatogram>

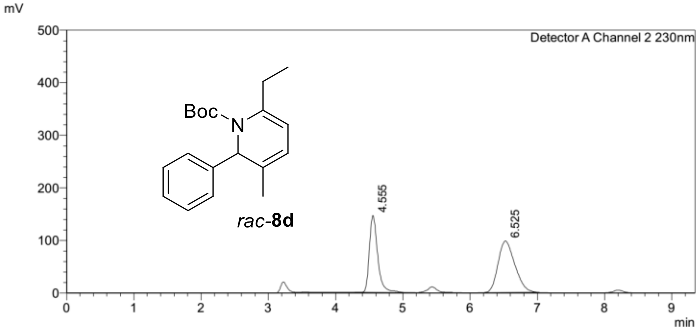

<Peak Table>

Detector A Channel 2 230nm

Peak#	Ret. Time	Area	Height	Conc.	Unit	Mark	Name
1	4.555	1269520	146020	42.799		M	
2	6.525	1696745	97722	57.201		M	
Total		2966266	243742				

Supplementary Figure 198. HPLC analysis for compound **8d**

SHIMADZU LabSolutions

Analysis Report

<Sample Information>

Sample Name	: mbs-ii-96-s-adh-99.5-0.5-1.0		
Sample ID	:		
Data Filename	: mbs-ii-96-s-adh-99.5-0.5-1.1.lcd		
Method Filename	: dpg-OZH-95-5-1.0-230.lcm		
Batch Filename	:		
Vial #	: 1-1	Sample Type	: Unknown
Injection Volume	: 20 uL		
Date Acquired	: 2017/11/2 15:56:11	Acquired by	: System Administrator
Date Processed	: 2017/11/2 16:09:00	Processed by	: System Administrator

<Chromatogram>

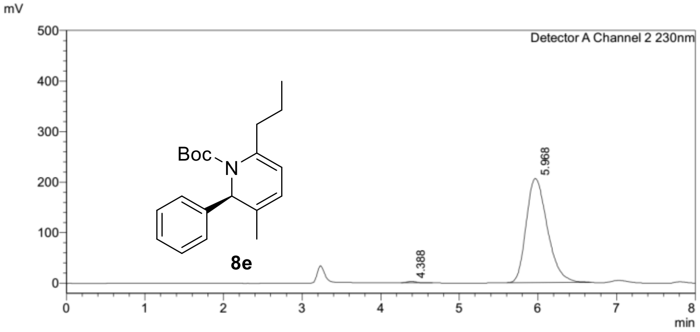

<Peak Table>

Detector A Channel 2 230nm

Peak#	Ret. Time	Area	Height	Conc.	Unit	Mark	Name
1	4.388	22073	2916	0.568		M	
2	5.968	3862344	206003	99.432		M	
Total		3884416	208919				

SHIMADZU LabSolutions

Analysis Report

<Sample Information>

Sample Name	: mbs-ii-104-rac-adh-99.5-0.5-1.0-2nd		
Sample ID	:		
Data Filename	: mbs-ii-104-rac-adh-99.5-0.5-1.0-2nd1.lcd		
Method Filename	: dpg-OZH-95-5-1.0-230.lcm		
Batch Filename	:		
Vial #	: 1-1	Sample Type	: Unknown
Injection Volume	: 20 uL		
Date Acquired	: 2017/11/2 15:19:56	Acquired by	: System Administrator
Date Processed	: 2017/11/2 15:29:25	Processed by	: System Administrator

<Chromatogram>

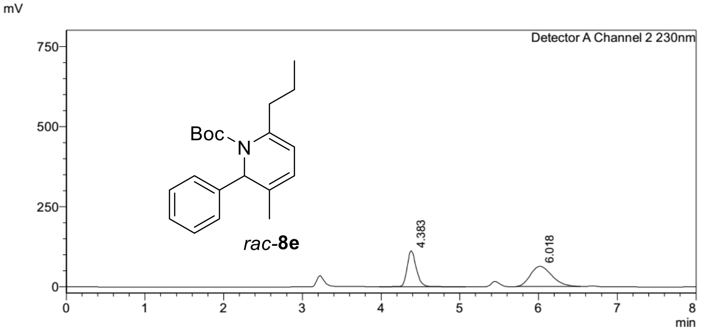

<Peak Table>

Detector A Channel 2 230nm

Peak#	Ret. Time	Area	Height	Conc.	Unit	Mark	Name
1	4.383	915168	111528	43.273		M	
2	6.018	1199725	63000	56.727		M	
Total		2114893	174529				

Supplementary Figure 199. HPLC analysis for compound **8e**

Analysis Report

<Sample Information>

Data Filename	: mbs-mh-39-asy-adh-99.5-0.5-1.0ml.lcd		
Method Filename	: 205 and 254nm.lcm		
Batch Filename	:		
Vial #	: 1-1	Sample Type	: Unknown
Injection Volume	: 20 uL		
Date Acquired	: 2020/5/10 10:32:34	Acquired by	: System Administrator
Date Processed	: 2020/5/10 10:43:37	Processed by	: System Administrator

<Chromatogram>

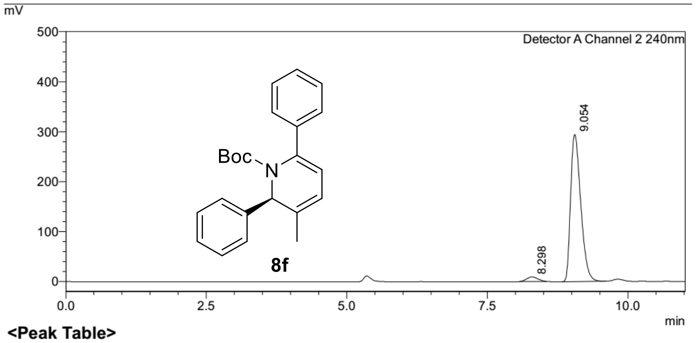

<Peak Table>

Detector A Channel 2 240nm

Peak#	Ret. Time	Area	Height	Conc.
1	8.298	126589	9192	3.310
2	9.054	3698320	294463	96.690
Total		3824909	303655	

Analysis Report

<Sample Information>

Data Filename	: mbs-mh-39-rac-adh-99.5-0.5-1.0ml.lcd		
Method Filename	: 205 and 254nm.lcm		
Batch Filename	:		
Vial #	: 1-1	Sample Type	: Unknown
Injection Volume	: 20 uL		
Date Acquired	: 2020/5/10 10:20:07	Acquired by	: System Administrator
Date Processed	: 2020/5/10 10:31:12	Processed by	: System Administrator

<Chromatogram>

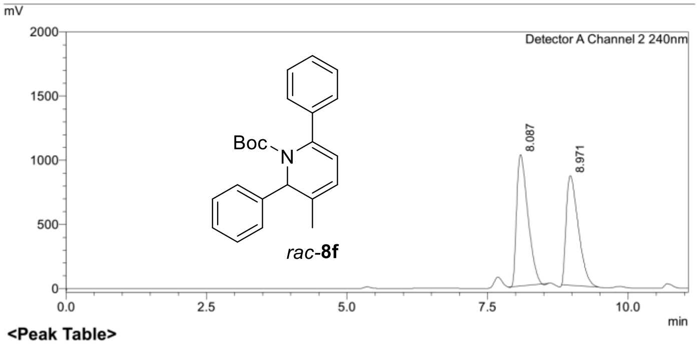

<Peak Table>

Detector A Channel 2 240nm

Peak#	Ret. Time	Area	Height	Conc.
1	8.087	14141530	1024035	52.416
2	8.971	12837780	853309	47.584
Total		26979310	1877344	

Supplementary Figure 200. HPLC analysis for compound **8f**

Analysis Report

<Sample Information>

Data Filename	: mbs-me-99-asy--adh-99.5-0.5-1.0ml1.lcd		
Method Filename	: WAC-93-FANFA.lcm		
Batch Filename	:		
Vial #	: 1-1	Sample Type	: Unknown
Injection Volume	: 20 uL		
Date Acquired	: 2019/7/2 11:41:37	Acquired by	: System Administrator
Date Processed	: 2019/7/2 11:50:43	Processed by	: System Administrator

<Chromatogram>

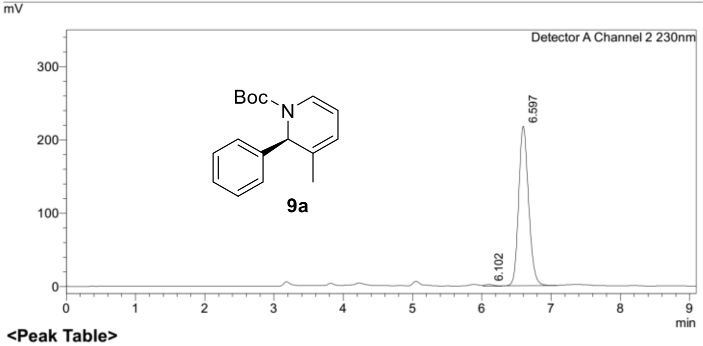

<Peak Table>

Detector A Channel 2 230nm

Peak#	Ret. Time	Area	Height	Conc.
1	6.102	20051	2199	0.914
2	6.597	2172516	217518	99.086
Total		2192567	219717	

Analysis Report

<Sample Information>

Data Filename	: mbs-me-53-rac--adh-99.5-0.5-1.0ml-4.lcd		
Method Filename	: WAC-93-FANFA.lcm		
Batch Filename	:		
Vial #	: 1-1	Sample Type	: Unknown
Injection Volume	: 20 uL		
Date Acquired	: 2019/7/2 11:21:45	Acquired by	: System Administrator
Date Processed	: 2019/7/2 11:29:56	Processed by	: System Administrator

<Chromatogram>

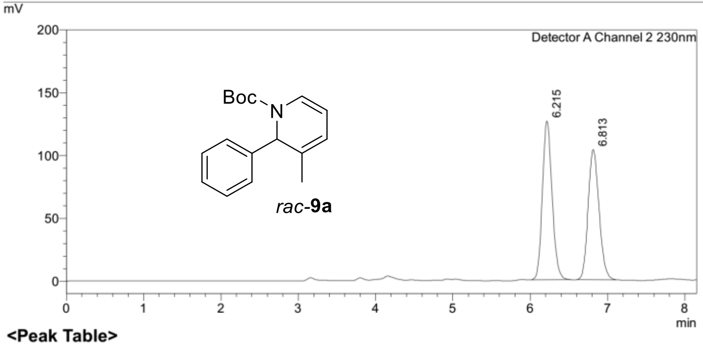

<Peak Table>

Detector A Channel 2 230nm

Peak#	Ret. Time	Area	Height	Conc.
1	6.215	1104495	126542	51.960
2	6.813	1021172	103624	48.040
Total		2125666	230166	

Supplementary Figure 201. HPLC analysis for compound **9a**

Analysis Report

<Sample Information>

Data Filename	: mbs-me-111-asy-adh-99.5-0.5-1.0ml1.lcd		
Method Filename	: WAC-93-FANFA.lcm		
Batch Filename	:		
Vial #	: 1-1	Sample Type	: Unknown
Injection Volume	: 20 uL		
Date Acquired	: 2019/7/1 23:08:03	Acquired by	: System Administrator
Date Processed	: 2019/7/1 23:18:16	Processed by	: System Administrator

<Chromatogram>

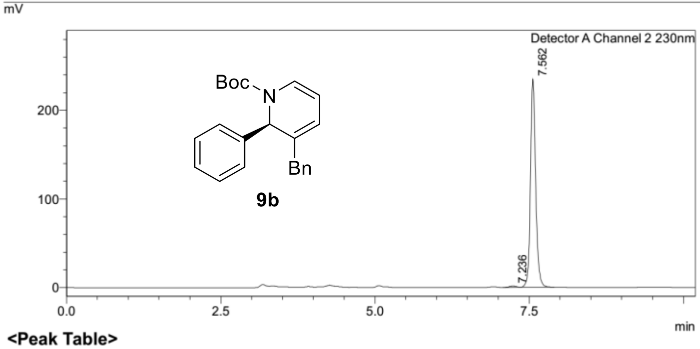

<Peak Table>

Detector A Channel 2 230nm

Peak#	Ret. Time	Area	Height	Conc.
1	7.236	8020	1149	0.584
2	7.562	1365181	234238	99.416
Total		1373202	235387	

Analysis Report

<Sample Information>

Data Filename	: mbs-me-110-rac-adh-99.5-0.5-1.0ml-3.lcd		
Method Filename	: WAC-93-FANFA.lcm		
Batch Filename	:		
Vial #	: 1-1	Sample Type	: Unknown
Injection Volume	: 20 uL		
Date Acquired	: 2019/7/1 22:54:20	Acquired by	: System Administrator
Date Processed	: 2019/7/1 23:06:50	Processed by	: System Administrator

<Chromatogram>

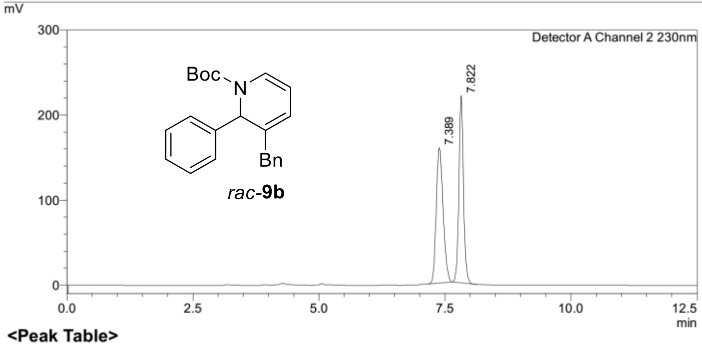

<Peak Table>

Detector A Channel 2 230nm

Peak#	Ret. Time	Area	Height	Conc.
1	7.389	1500484	158825	51.691
2	7.822	1402318	220207	48.309
Total		2902803	379032	

Supplementary Figure 202. HPLC analysis for compound **9b**

Analysis Report

<Sample Information>

Data Filename	: mbs-me-124-asy--adh-99.5-0.5-1.0ml1.lcd		
Method Filename	: WAC-93-FANFA.lcm		
Batch Filename	:		
Vial #	: 1-1	Sample Type	: Unknown
Injection Volume	: 20 uL		
Date Acquired	: 2019/7/2 12:00:25	Acquired by	: System Administrator
Date Processed	: 2019/7/2 12:07:34	Processed by	: System Administrator

<Chromatogram>

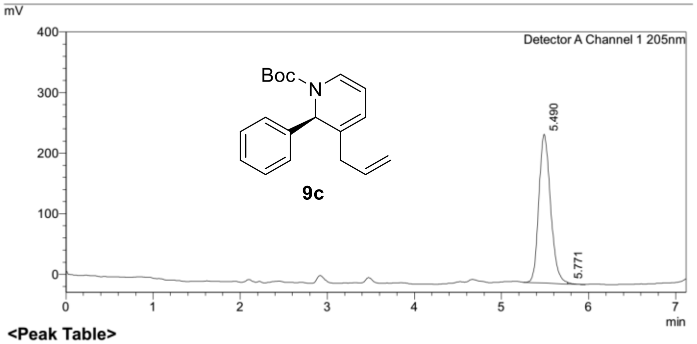

<Peak Table>

Detector A Channel 1 205nm

Peak#	Ret. Time	Area	Height	Conc.
1	5.490	2297177	245546	99.894
2	5.771	2449	1965	0.106
Total		2299625	247511	

Analysis Report

<Sample Information>

Data Filename	: mbs-me-124-rac--adh-99.5-0.5-1.0ml1.lcd		
Method Filename	: WAC-93-FANFA.lcm		
Batch Filename	:		
Vial #	: 1-1	Sample Type	: Unknown
Injection Volume	: 20 uL		
Date Acquired	: 2019/7/2 11:52:29	Acquired by	: System Administrator
Date Processed	: 2019/7/2 11:59:24	Processed by	: System Administrator

<Chromatogram>

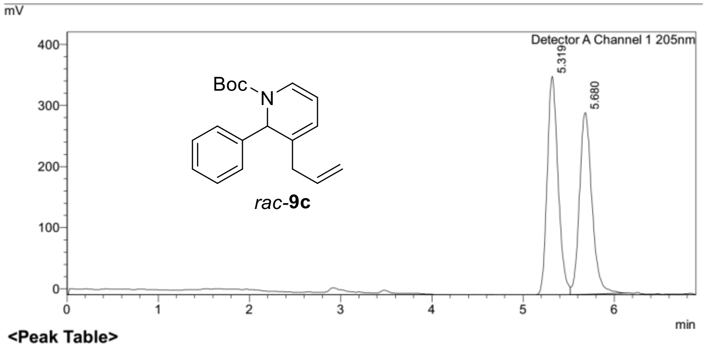

<Peak Table>

Detector A Channel 1 205nm

Peak#	Ret. Time	Area	Height	Conc.
1	5.319	2979791	358104	51.406
2	5.680	2816830	297128	48.594
Total		5796621	655232	

Supplementary Figure 203. HPLC analysis for compound **9c**

Analysis Report

<Sample Information>

Data Filename	: mbs-me-112-asy-adh-99.5-0.5-1ml.lcd		
Method Filename	: 278-254nm.lcm		
Batch Filename	:		
Vial #	: 1-1	Sample Type	: Unknown
Injection Volume	: 20 uL		
Date Acquired	: 2019/6/2 13:45:12	Acquired by	: System Administrator
Date Processed	: 2019/6/2 14:00:23	Processed by	: System Administrator

<Chromatogram>

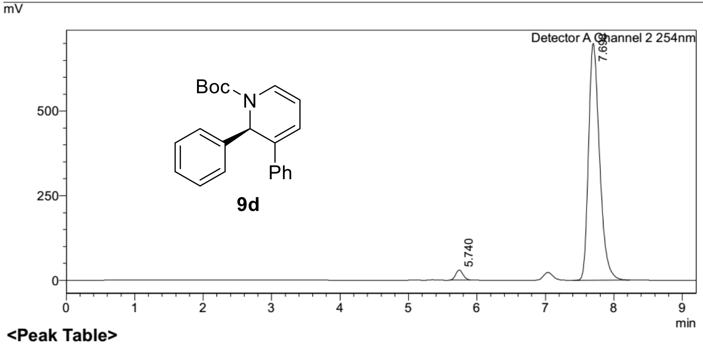

<Peak Table>

Detector A Channel 2 254nm

Peak#	Ret. Time	Area	Height	Conc.
1	5.740	217328	29275	2.685
2	7.698	7876239	699454	97.315
Total		8093567	728728	

Analysis Report

<Sample Information>

Data Filename	: mbs-me-113-rac-adh-99.5-0.5-1ml.lcd		
Method Filename	: 278-254nm.lcm		
Batch Filename	:		
Vial #	: 1-1	Sample Type	: Unknown
Injection Volume	: 20 uL		
Date Acquired	: 2019/6/2 10:12:44	Acquired by	: System Administrator
Date Processed	: 2019/6/2 11:01:21	Processed by	: System Administrator

<Chromatogram>

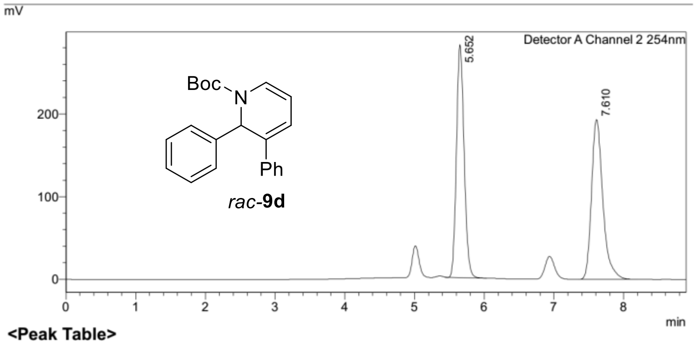

<Peak Table>

Detector A Channel 2 254nm

Peak#	Ret. Time	Area	Height	Conc.
1	5.652	2152828	281062	49.650
2	7.610	2183178	193129	50.350
Total		4336006	474191	

Supplementary Figure 204. HPLC analysis for compound **9d**

Analysis Report

<Sample Information>

Sample Name	: cuy-cc-69-1-asy-ia-99-1-0.8-2		
Sample ID	:		
Data Filename	: cuy-cc-69-1-asy-ia-99-1-0.8-3.lcd		
Method Filename	: 20190312.lcm		
Batch Filename	:		
Vial #	: 1-1	Sample Type	: Unknown
Injection Volume	: 20 uL		
Date Acquired	: 2019/5/27 10:57:50	Acquired by	: System Administrator
Date Processed	: 2019/5/27 11:30:22	Processed by	: System Administrator

<Chromatogram>

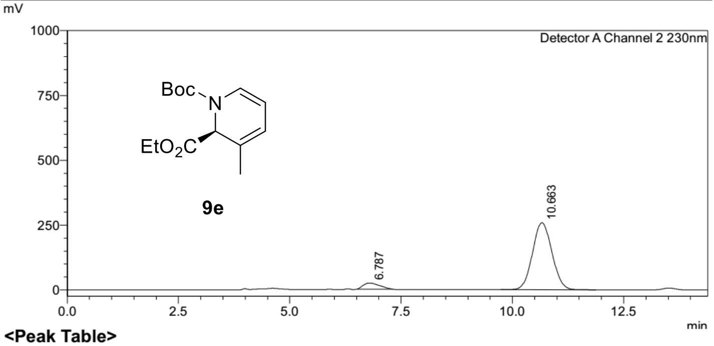

<Peak Table>

Detector A Channel 2 230nm

Peak#	Ret. Time	Area	Height	Conc.
1	6.787	599082	23302	7.075
2	10.663	7869050	258695	92.925
Total		8468131	281997	

Analysis Report

<Sample Information>

Sample Name	: cuy-cc-74-5-rac-ia-99-1-0.8-2		
Sample ID	:		
Data Filename	: cuy-cc-74-5-rac-ia-99-1-0.8-3.lcd		
Method Filename	: 20190312.lcm		
Batch Filename	:		
Vial #	: 1-1	Sample Type	: Unknown
Injection Volume	: 20 uL		
Date Acquired	: 2019/5/27 11:13:44	Acquired by	: System Administrator
Date Processed	: 2019/5/27 11:31:03	Processed by	: System Administrator

<Chromatogram>

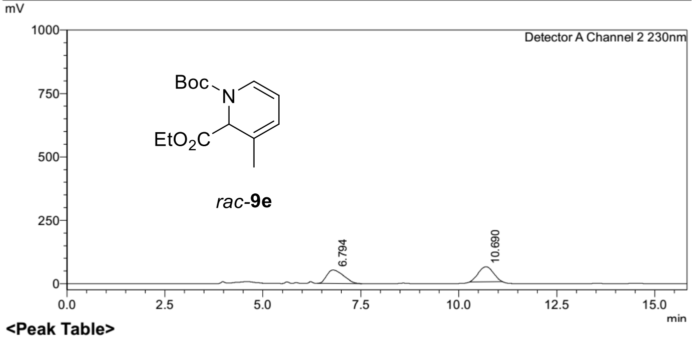

<Peak Table>

Detector A Channel 2 230nm

Peak#	Ret. Time	Area	Height	Conc.
1	6.794	1561061	52934	49.866
2	10.690	1569469	59704	50.134
Total		3130530	112638	

Supplementary Figure 205. HPLC analysis for compound **9e**

Analysis Report

<Sample Information>

Sample Name : cuy-cc-83-2-asy-ia-99-1-230-0.8-2
Sample ID :
Data Filename : cuy-cc-83-2-asy-ia-99-1-230-0.8-3.lcd
Method Filename : 20190312.lcm
Batch Filename :
Vial # : 1-1
Injection Volume : 20 uL
Date Acquired : 2019/5/30 21:16:22
Date Processed : 2019/5/30 21:30:45
Sample Type : Unknown
Acquired by : System Administrator
Processed by : System Administrator

<Chromatogram>

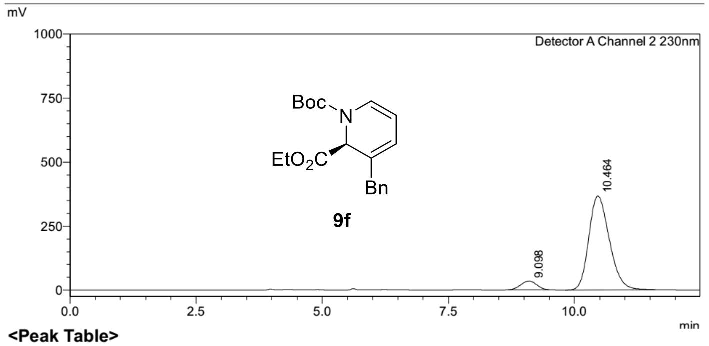

<Peak Table>

Detector A Channel 2 230nm

Peak#	Ret. Time	Area	Height	Conc.
1	9.098	748103	34315	6.716
2	10.464	10390679	366493	93.284
Total		11138783	400807	

Analysis Report

<Sample Information>

Sample Name : cuy-cc-83-1-rac-ia-99-1-230-0.8-1
Sample ID :
Data Filename : cuy-cc-83-1-rac-ia-99-1-230-0.8-2.lcd
Method Filename : 20190312.lcm
Batch Filename :
Vial # : 1-1
Injection Volume : 20 uL
Date Acquired : 2019/5/30 20:43:36
Date Processed : 2019/5/30 21:30:53
Sample Type : Unknown
Acquired by : System Administrator
Processed by : System Administrator

<Chromatogram>

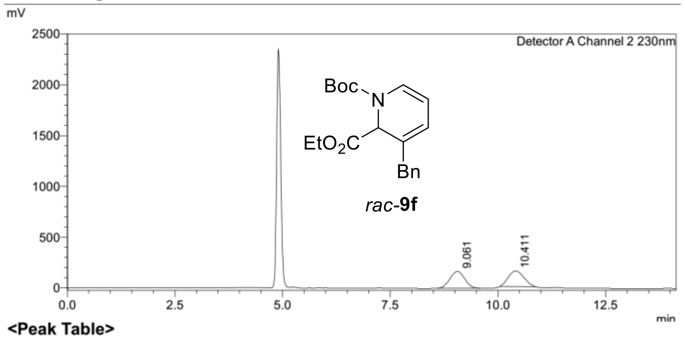

<Peak Table>

Detector A Channel 2 230nm

Peak#	Ret. Time	Area	Height	Conc.
1	9.061	3902302	164646	49.627
2	10.411	3960921	155076	50.373
Total		7863223	319722	

Supplementary Figure 206. HPLC analysis for compound **9f**

SHIMADZU LabSolutions

Analysis Report

<Sample Information>

Sample Name	: mbs-ma-37-s-adh-99.5-0.5-1.0-2nd		
Sample ID	:		
Data Filename	: mbs-ma-37-s-adh-99.5-0.5-1.0-2nd.lcd		
Method Filename	: wa-1.0.lcm		
Batch Filename	:		
Vial #	: 1-1	Sample Type	: Unknown
Injection Volume	: 20 uL		
Date Acquired	: 2018/1/3 17:27:10	Acquired by	: System Administrator
Date Processed	: 2018/1/17 16:44:04	Processed by	: System Administrator

<Chromatogram>

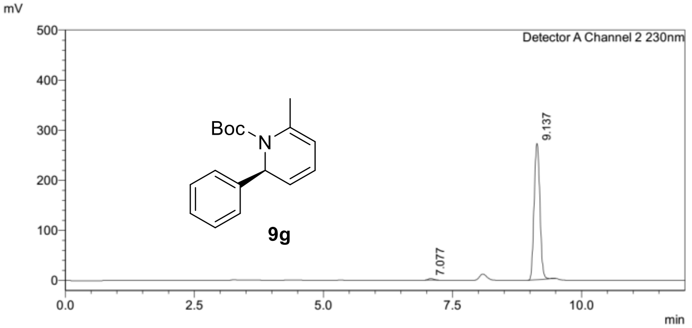

<Peak Table>

Detector A Channel 2 230nm

Peak#	Ret. Time	Area	Height	Conc.	Unit	Mark	Name
1	7.077	24549	3065	1.140		M	
2	9.137	2128639	272010	98.860		M	
Total		2153188	275075				

SHIMADZU LabSolutions

Analysis Report

<Sample Information>

Sample Name	: mbs-ma-38-rac-adh-99.5-0.5-1.0-3nd		
Sample ID	:		
Data Filename	: mbs-ma-38-rac-adh-99.5-0.5-1.0-3nd1.lcd		
Method Filename	: wa-1.0.lcm		
Batch Filename	:		
Vial #	: 1-1	Sample Type	: Unknown
Injection Volume	: 20 uL		
Date Acquired	: 2018/1/3 19:49:52	Acquired by	: System Administrator
Date Processed	: 2018/1/3 20:25:46	Processed by	: System Administrator

<Chromatogram>

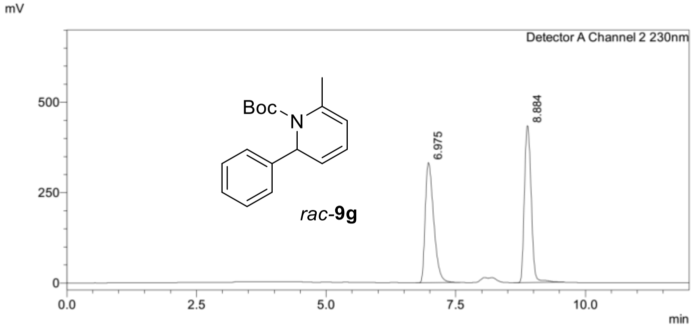

<Peak Table>

Detector A Channel 2 230nm

Peak#	Ret. Time	Area	Height	Conc.	Unit	Mark	Name
1	6.975	3778297	332553	48.675		M	
2	8.884	3984076	434605	51.325		M	
Total		7762373	767158				

Supplementary Figure 207. HPLC analysis for compound **9g**

Analysis Report

<Sample Information>

Sample Name	: cuy-cb-141-1-asy-adh-99.5-0.5-1.0-230		
Sample ID	:		
Data Filename	: cuy-cb-141-1-asy-adh-99.5-0.5-1.0-230.lcd		
Method Filename	: 20190312.lcm		
Batch Filename	:		
Vial #	: 1-1	Sample Type	: Unknown
Injection Volume	: 20 uL		
Date Acquired	: 2019/5/9 14:49:09	Acquired by	: System Administrator
Date Processed	: 2019/5/10 20:07:49	Processed by	: System Administrator

<Chromatogram>

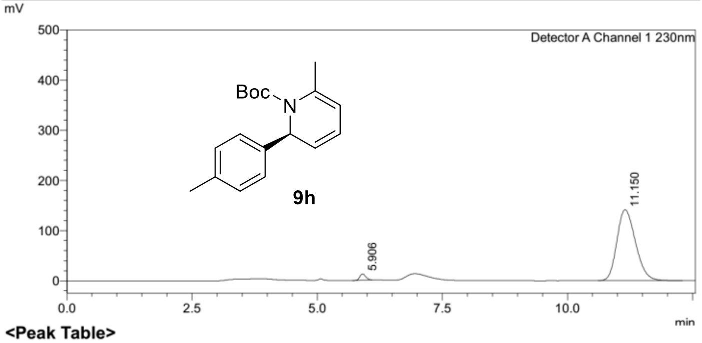

<Peak Table>

Detector A Channel 1 230nm

Peak#	Ret. Time	Area	Height	Conc.
1	5.906	102616	12445	2.737
2	11.150	3646527	141424	97.263
Total		3749143	153869	

Analysis Report

<Sample Information>

Sample Name	: cuy-cc-48-1-rac-adh-99.5-0.5-1.0-230		
Sample ID	:		
Data Filename	: cuy-cc-48-1-rac-adh-99.5-0.5-1.0-230.lcd		
Method Filename	: 20190312.lcm		
Batch Filename	:		
Vial #	: 1-1	Sample Type	: Unknown
Injection Volume	: 20 uL		
Date Acquired	: 2019/5/9 14:18:52	Acquired by	: System Administrator
Date Processed	: 2019/5/10 20:06:59	Processed by	: System Administrator

<Chromatogram>

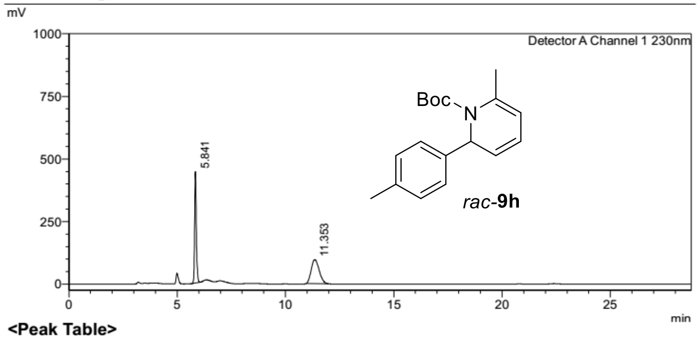

<Peak Table>

Detector A Channel 1 230nm

Peak#	Ret. Time	Area	Height	Conc.
1	5.841	2504883	445544	50.379
2	11.353	2467188	96118	49.621
Total		4972071	541662	

Supplementary Figure 208. HPLC analysis for compound **9h**

Analysis Report

<Sample Information>

Sample Name : cuy-cc-39-3-rac-adh-99.5-0.5-254-1.0
Sample ID :
Data Filename : cuy-cc-39-3-asy-adh-99.5-0.5-1.0-230.lcd
Method Filename : xsl-230-254-1.0.lcm
Batch Filename :
Vial # : 1-1 Sample Type : Unknown
Injection Volume : 20 uL
Date Acquired : 2019/5/17 12:54:37 Acquired by : System Administrator
Date Processed : 2019/5/17 14:02:55 Processed by : System Administrator

<Chromatogram>

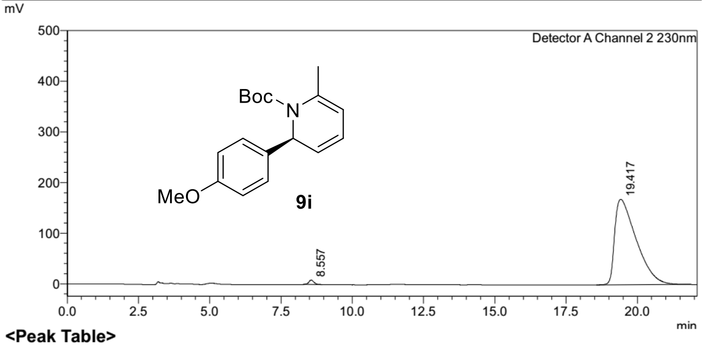

<Peak Table>

Detector A Channel 2 230nm

Peak#	Ret. Time	Area	Height	Conc.
1	8.557	110944	8624	1.231
2	19.417	8903023	168754	98.769
Total		9013968	177379	

Analysis Report

<Sample Information>

Sample Name : cuy-cc-28-3-rac-adh-99.5-0.5-254-1.0
Sample ID :
Data Filename : cuy-cc-28-3-rac-adh-99.5-0.5-254-1.1.lcd
Method Filename : xsl-230-254-1.0.lcm
Batch Filename :
Vial # : 1-1 Sample Type : Unknown
Injection Volume : 20 uL
Date Acquired : 2019/5/17 13:21:34 Acquired by : System Administrator
Date Processed : 2019/5/21 8:49:03 Processed by : System Administrator

<Chromatogram>

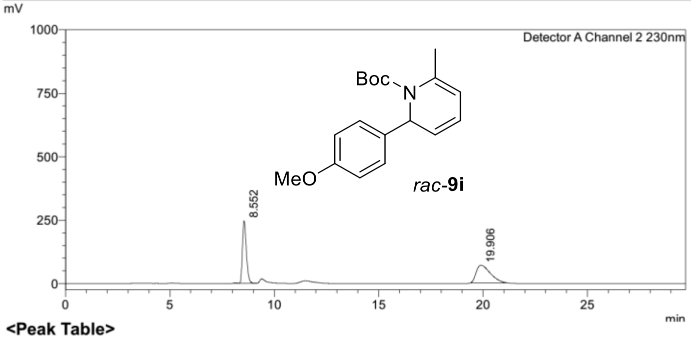

<Peak Table>

Detector A Channel 2 230nm

Peak#	Ret. Time	Area	Height	Conc.
1	8.552	3165841	245363	49.054
2	19.906	3287945	70040	50.946
Total		6453786	315403	

Supplementary Figure 209. HPLC analysis for compound **9i**

SHIMADZU LabSolutions

Analysis Report

<Sample Information>

Sample Name	: cuy-cb-143-1-asy-adh-95.5-0.5-1.0-230,254		
Sample ID	:		
Data Filename	: cuy-cb-143-1-asy-adh-95.5-0.5-1.0-230,255.lcd		
Method Filename	: lik-run-1.0.lcm		
Batch Filename	:		
Vial #	: 1-1	Sample Type	: Unknown
Injection Volume	: 20 uL		
Date Acquired	: 5/11/2019 3:02:45 PM	Acquired by	: System Administrator
Date Processed	: 5/17/2019 9:15:47 AM	Processed by	: System Administrator

<Chromatogram>

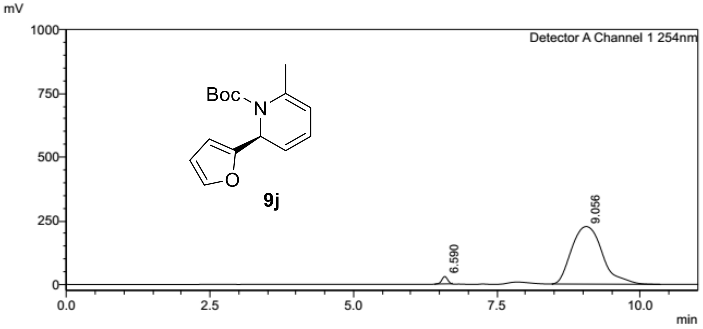

<Peak Table>

Detector A Channel 1 254nm

Peak#	Ret. Time	Height	Mark	Area%
1	6.590	29214	M	2.380
2	9.056	225944	M	97.620
Total		255158		100.000

SHIMADZU LabSolutions

Analysis Report

<Sample Information>

Sample Name	: cuy-cc-54-1-RAC-adh-95.5-0.5-1.0-230,254		
Sample ID	:		
Data Filename	: cuy-cc-54-1-RAC-adh-95.5-0.5-1.0-230,255.lcd		
Method Filename	: lik-run-1.0.lcm		
Batch Filename	:		
Vial #	: 1-1	Sample Type	: Unknown
Injection Volume	: 20 uL		
Date Acquired	: 5/11/2019 2:39:50 PM	Acquired by	: System Administrator
Date Processed	: 5/21/2019 12:34:49 PM	Processed by	: System Administrator

<Chromatogram>

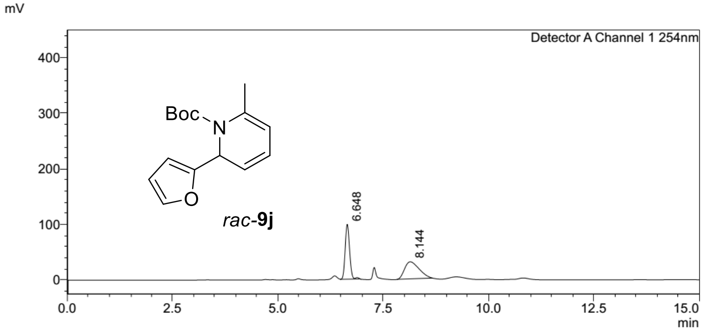

<Peak Table>

Detector A Channel 1 254nm

Peak#	Ret. Time	Height	Mark	Area%
1	6.648	98553	M	49.817
2	8.144	30730	M	50.183
Total		129283		100.000

Supplementary Figure 210. HPLC analysis for compound **9j**

Analysis Report

<Sample Information>

Sample Name : cuy-cg-72-1-asy-adh-95.5-0.5-1.0-230nm-1
Sample ID :
Data Filename : cuy-cg-72-1-asy-adh-95.5-0.5-1.0-230nm-2.lcd
Method Filename : 1.0ml-254-230.lcm
Batch Filename :
Vial # : 1-1 Sample Type : Unknown
Injection Volume : 20 uL
Date Acquired : 2020/10/1 17:07:37 Acquired by : System Administrator
Date Processed : 2020/10/1 17:15:14 Processed by : System Administrator

<Chromatogram>

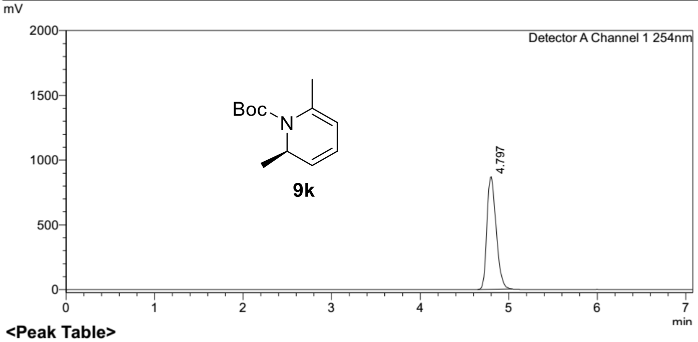

<Peak Table>

Detector A Channel 1 254nm

Peak#	Ret. Time	Area	Height	Conc.
1	4.797	6301759	869773	100.000
Total		6301759	869773	

Analysis Report

<Sample Information>

Sample Name : cuy-cg-85-1-rac-adh-95.5-0.5-1.0-230nm-1
Sample ID :
Data Filename : cuy-cg-85-1-rac-adh-95.5-0.5-1.0-230nm-2.lcd
Method Filename : 1.0ml-254-230.lcm
Batch Filename :
Vial # : 1-1 Sample Type : Unknown
Injection Volume : 20 uL
Date Acquired : 2020/10/1 16:51:37 Acquired by : System Administrator
Date Processed : 2020/10/1 17:15:24 Processed by : System Administrator

<Chromatogram>

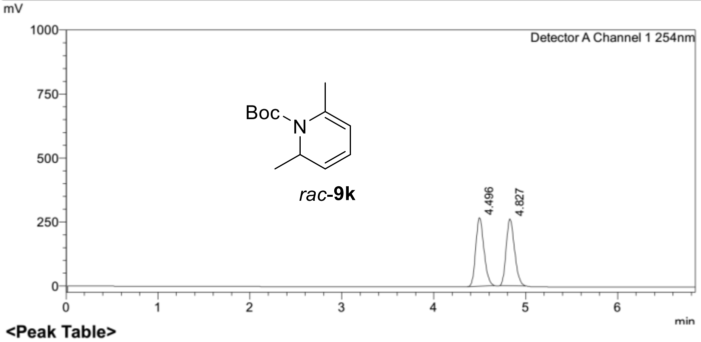

<Peak Table>

Detector A Channel 1 254nm

Peak#	Ret. Time	Area	Height	Conc.
1	4.496	1663315	266773	50.205
2	4.827	1649739	260205	49.795
Total		3313053	526978	

Supplementary Figure 211. HPLC analysis for compound **9k**

Analysis Report

<Sample Information>

Sample Name	: cuy-cg-123-1-asy--adh-99.9-0.1-0.5-254nm-01		
Sample ID	:		
Data Filename	: cuy-cg-123-1-asy--adh-99.9-0.1-0.5-254nm-2.lcd		
Method Filename	: xsl-230-254-1.0.lcm		
Batch Filename	:		
Vial #	: 1-1	Sample Type	: Unknown
Injection Volume	: 20 uL		
Date Acquired	: 2020/11/10 10:15:23	Acquired by	: System Administrator
Date Processed	: 2020/11/10 10:35:26	Processed by	: System Administrator

<Chromatogram>

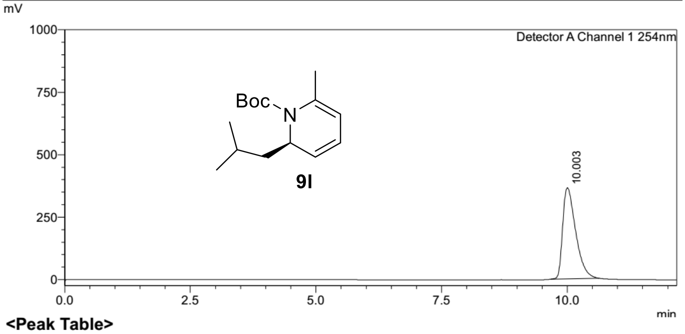

<Peak Table>

Detector A Channel 1 254nm

Peak#	Ret. Time	Area	Height	Conc.
1	10.003	6576459	364893	100.000
Total		6576459	364893	

Analysis Report

<Sample Information>

Sample Name	: cuy-cg-123-1-rac--adh-99.9-0.1-0.5-230nm-01		
Sample ID	:		
Data Filename	: cuy-cg-123-1-rac--adh-99.9-0.1-0.5-230nm-2.lcd		
Method Filename	: xsl-230-254-1.0.lcm		
Batch Filename	:		
Vial #	: 1-1	Sample Type	: Unknown
Injection Volume	: 20 uL		
Date Acquired	: 2020/11/10 9:15:19	Acquired by	: System Administrator
Date Processed	: 2020/11/10 10:35:24	Processed by	: System Administrator

<Chromatogram>

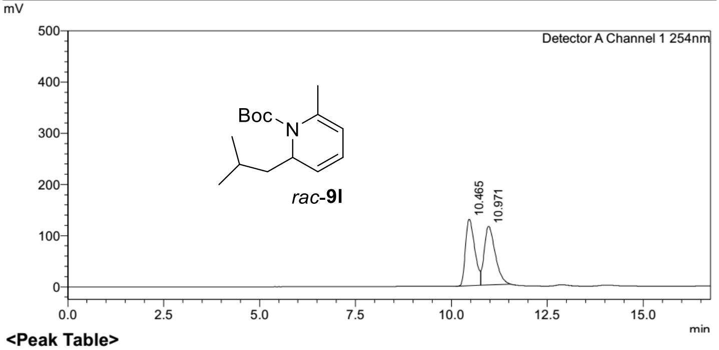

<Peak Table>

Detector A Channel 1 254nm

Peak#	Ret. Time	Area	Height	Conc.
1	10.465	2211332	130071	49.118
2	10.971	2290757	114717	50.882
Total		4502089	244788	

Supplementary Figure 212. HPLC analysis for compound **9l**

Analysis Report

<Sample Information>

Data Filename	: mbs-md-139-asy-odh-99.5-0.5-1.0ml1.lcd		
Method Filename	: 20190312-205-250.lcm		
Batch Filename	:		
Vial #	: 1-1	Sample Type	: Unknown
Injection Volume	: 20 uL		
Date Acquired	: 2019/3/13 21:31:35	Acquired by	: System Administrator
Date Processed	: 2019/7/1 21:04:56	Processed by	: System Administrator

<Chromatogram>

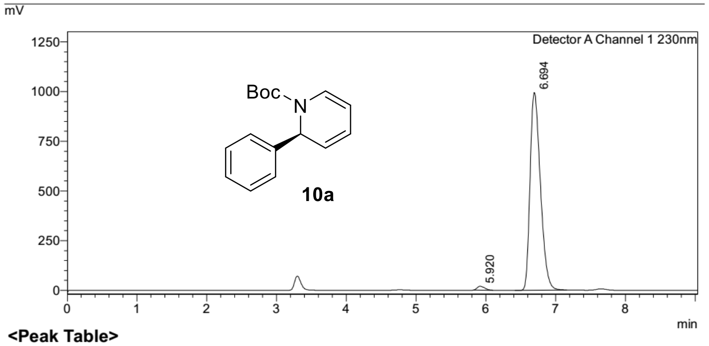

<Peak Table>

Detector A Channel 1 230nm

Peak#	Ret. Time	Area	Height	Conc.
1	5.920	143898	19325	1.386
2	6.694	10241594	994725	98.614
Total		10385493	1014050	

Analysis Report

<Sample Information>

Data Filename	: mbs-me-7-rac-odh-99.5-0.5-1.0ml1.lcd		
Method Filename	: 20190312-205-250.lcm		
Batch Filename	:		
Vial #	: 1-1	Sample Type	: Unknown
Injection Volume	: 20 uL		
Date Acquired	: 2019/3/13 20:59:13	Acquired by	: System Administrator
Date Processed	: 2019/3/13 21:17:07	Processed by	: System Administrator

<Chromatogram>

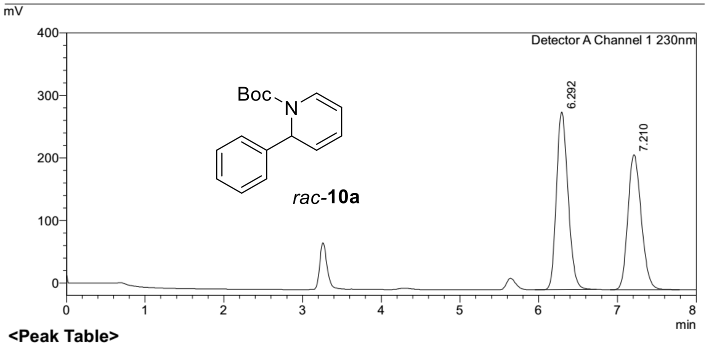

<Peak Table>

Detector A Channel 1 230nm

Peak#	Ret. Time	Area	Height	Conc.
1	6.292	2915316	284194	53.299
2	7.210	2554408	215942	46.701
Total		5469724	500137	

Supplementary Figure 213. HPLC analysis for compound **10a**

Analysis Report

<Sample Information>

Data Filename	: mbs-me-132-asy-odh-99.5-0.5-1.0ml1.lcd		
Method Filename	: WAC-93-FANFA.lcm		
Batch Filename	:		
Vial #	: 1-1	Sample Type	: Unknown
Injection Volume	: 20 uL		
Date Acquired	: 2019/7/1 22:05:42	Acquired by	: System Administrator
Date Processed	: 2019/7/1 22:12:33	Processed by	: System Administrator

<Chromatogram>

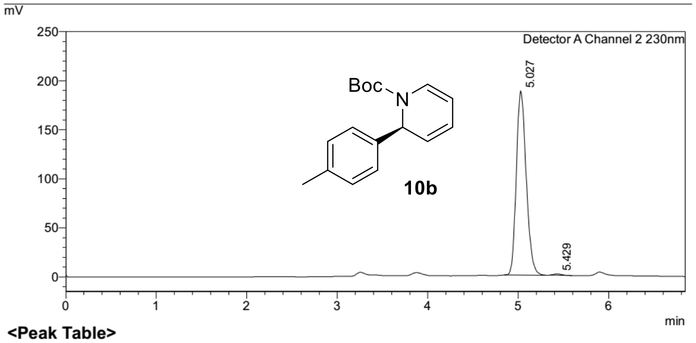

<Peak Table>

Detector A Channel 2 230nm

Peak#	Ret. Time	Area	Height	Conc.
1	5.027	1425400	187436	99.424
2	5.429	8257	1345	0.576
Total		1433657	188781	

Analysis Report

<Sample Information>

Data Filename	: mbs-me-131-rac-odh-99.5-0.5-1.0ml1.lcd		
Method Filename	: WAC-93-FANFA.lcm		
Batch Filename	:		
Vial #	: 1-1	Sample Type	: Unknown
Injection Volume	: 20 uL		
Date Acquired	: 2019/7/1 21:34:49	Acquired by	: System Administrator
Date Processed	: 2019/7/1 22:04:20	Processed by	: System Administrator

<Chromatogram>

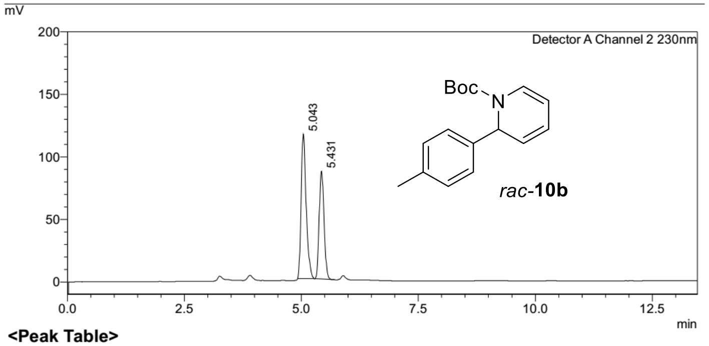

<Peak Table>

Detector A Channel 2 230nm

Peak#	Ret. Time	Area	Height	Conc.
1	5.043	863080	115456	58.000
2	5.431	624989	86327	42.000
Total		1488070	201783	

Supplementary Figure 214. HPLC analysis for compound **10b**

Analysis Report

<Sample Information>

Sample Name : cuy-cc-65-1-01-asy-4-AD-H-99-1-1.0
Sample ID :
Data Filename : cuy-cc-65-1-01-asy-4-AD-H-99-1-1.1.lcd
Method Filename : WAC-93-FANFA.lcm
Batch Filename :
Vial # : 1-1 Sample Type : Unknown
Injection Volume : 20 uL
Date Acquired : 2019/6/26 15:04:57 Acquired by : System Administrator
Date Processed : 2019/6/26 15:45:02 Processed by : System Administrator

<Chromatogram>

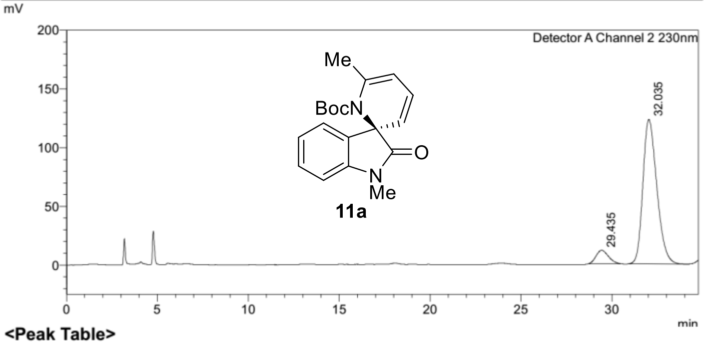

<Peak Table>

Detector A Channel 2 230nm

Peak#	Ret. Time	Area	Height	Conc.
1	29.435	543215	11475	7.636
2	32.035	6570921	123077	92.364
Total		7114135	134552	

Analysis Report

<Sample Information>

Sample Name : cuy-cc-62-1-01-rac-4-AD-H-99-1-1.0
Sample ID :
Data Filename : cuy-cc-62-1-01-rac-4-AD-H-99-1-1.1.lcd
Method Filename : WAC-93-FANFA.lcm
Batch Filename :
Vial # : 1-1 Sample Type : Unknown
Injection Volume : 20 uL
Date Acquired : 2019/6/26 14:14:29 Acquired by : System Administrator
Date Processed : 2019/6/26 15:45:15 Processed by : System Administrator

<Chromatogram>

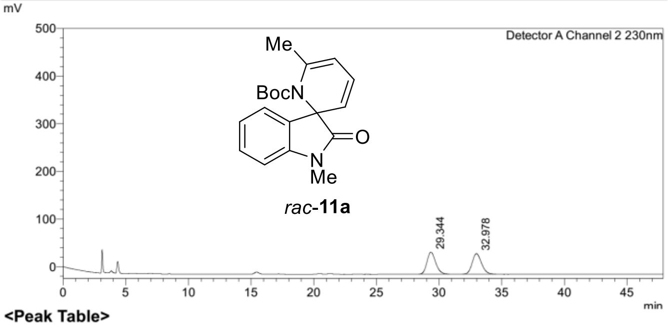

<Peak Table>

Detector A Channel 2 230nm

Peak#	Ret. Time	Area	Height	Conc.
1	29.344	2225273	46167	49.831
2	32.978	2240380	43034	50.169
Total		4465653	89201	

Supplementary Figure 215. HPLC analysis for compound **11a**

Analysis Report

<Sample Information>

Sample Name	: cuy-cc-121-2-01-asy-ojh-95-5-1.0		
Sample ID	:		
Data Filename	: cuy-cc-121-2-01-asy-ojh-95-5-1.1.lcd		
Method Filename	: WAC-93-FANFA.lcm		
Batch Filename	:		
Vial #	: 1-1	Sample Type	: Unknown
Injection Volume	: 20 uL		
Date Acquired	: 2019/6/27 14:09:30	Acquired by	: System Administrator
Date Processed	: 2019/6/27 14:42:22	Processed by	: System Administrator

<Chromatogram>

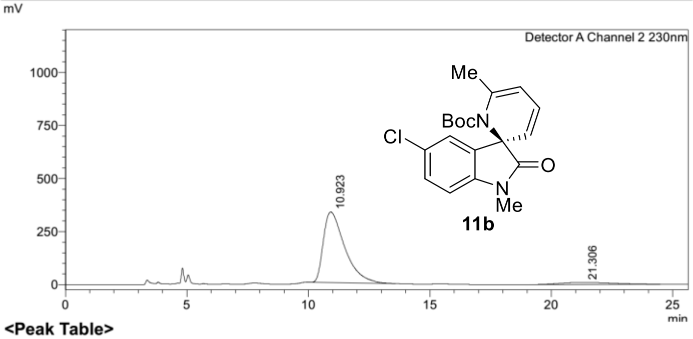

<Peak Table>

Detector A Channel 2 230nm

Peak#	Ret. Time	Area	Height	Conc.
1	10.923	20411546	332345	93.434
2	21.306	1434419	9448	6.566
Total		21845965	341794	

Analysis Report

<Sample Information>

Sample Name	: cuy-cc-120-1-rac-ojh-95-5-1.0		
Sample ID	:		
Data Filename	: cuy-cc-120-1-rac-ojh-95-5-1.1.lcd		
Method Filename	: WAC-93-FANFA.lcm		
Batch Filename	:		
Vial #	: 1-1	Sample Type	: Unknown
Injection Volume	: 20 uL		
Date Acquired	: 2019/6/27 11:20:09	Acquired by	: System Administrator
Date Processed	: 2019/6/27 14:42:25	Processed by	: System Administrator

<Chromatogram>

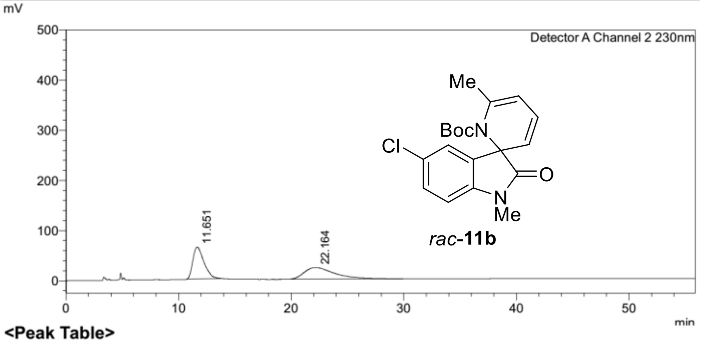

<Peak Table>

Detector A Channel 2 230nm

Peak#	Ret. Time	Area	Height	Conc.
1	11.651	4386225	63831	49.718
2	22.164	4436068	23214	50.282
Total		8822293	87045	

Supplementary Figure 216. HPLC analysis for compound **11b**

Analysis Report

<Sample Information>

Sample Name	: cuy-cc-121-1-01-asy-ojh-95-5-1.0		
Sample ID	:		
Data Filename	: cuy-cc-121-1-01-asy-ojh-95-5-1.1.lcd		
Method Filename	: WAC-93-FANFA.lcm		
Batch Filename	:		
Vial #	: 1-1	Sample Type	: Unknown
Injection Volume	: 20 uL		
Date Acquired	: 2019/6/27 15:19:29	Acquired by	: System Administrator
Date Processed	: 2019/6/27 15:59:18	Processed by	: System Administrator

<Chromatogram>

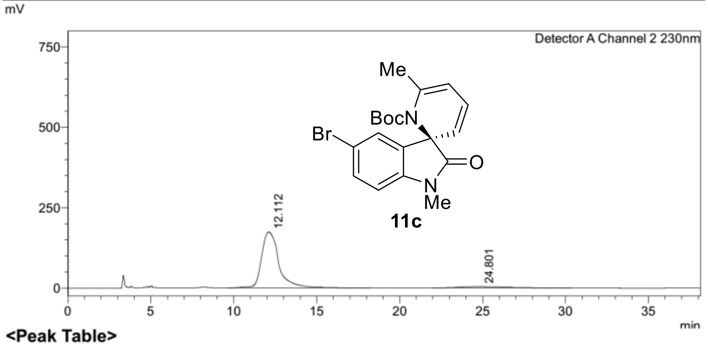

<Peak Table>

Detector A Channel 2 230nm

Peak#	Ret. Time	Area	Height	Conc.
1	12.112	12651960	173918	93.587
2	24.801	866924	4238	6.413
Total		13518884	178156	

Analysis Report

<Sample Information>

Sample Name	: cuy-cc-62-3-01-rac-ojh-95-5-1.0		
Sample ID	:		
Data Filename	: cuy-cc-62-3-01-rac-ojh-95-5-1.1.lcd		
Method Filename	: WAC-93-FANFA.lcm		
Batch Filename	:		
Vial #	: 1-1	Sample Type	: Unknown
Injection Volume	: 20 uL		
Date Acquired	: 2019/6/27 14:40:37	Acquired by	: System Administrator
Date Processed	: 2019/6/27 15:37:59	Processed by	: System Administrator

<Chromatogram>

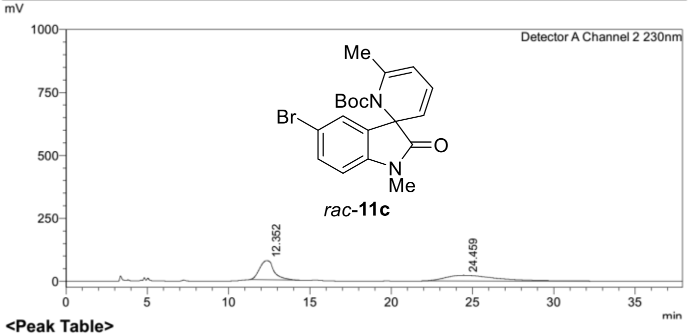

<Peak Table>

Detector A Channel 2 230nm

Peak#	Ret. Time	Area	Height	Conc.
1	12.352	4769509	76241	49.287
2	24.459	4907551	23055	50.713
Total		9677060	99296	

Supplementary Figure 217. HPLC analysis for compound **11c**

Analysis Report

<Sample Information>

Sample Name	: cuy-cc-123-1-02-asy-adh-99-1-1.0		
Sample ID	:		
Data Filename	: cuy-cc-123-1-02-asy-adh-99-1-1.1.lcd		
Method Filename	: WAC-93-FANFA.lcm		
Batch Filename	:		
Vial #	: 1-1	Sample Type	: Unknown
Injection Volume	: 20 uL		
Date Acquired	: 2019/6/28 17:10:25	Acquired by	: System Administrator
Date Processed	: 2019/6/28 17:50:50	Processed by	: System Administrator

<Chromatogram>

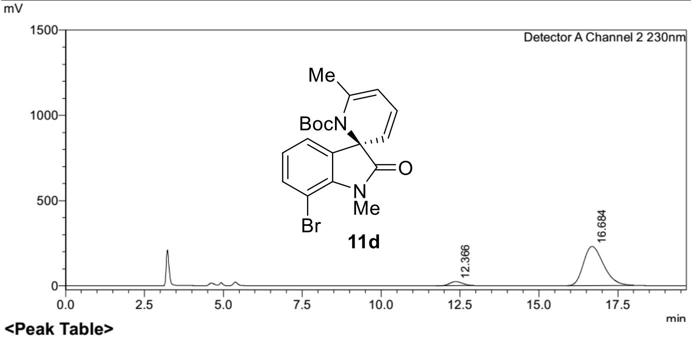

<Peak Table>

Detector A Channel 2 230nm

Peak#	Ret. Time	Area	Height	Conc.
1	12.366	678601	24166	6.269
2	16.684	10146275	229059	93.731
Total		10824876	253225	

Analysis Report

<Sample Information>

Sample Name	: cuy-cc-122-3-04-rac-adh-99-1-1.0		
Sample ID	:		
Data Filename	: cuy-cc-122-3-04-rac-adh-99-1-1.1.lcd		
Method Filename	: WAC-93-FANFA.lcm		
Batch Filename	:		
Vial #	: 1-1	Sample Type	: Unknown
Injection Volume	: 20 uL		
Date Acquired	: 2019/6/28 17:31:34	Acquired by	: System Administrator
Date Processed	: 2019/6/28 17:52:50	Processed by	: System Administrator

<Chromatogram>

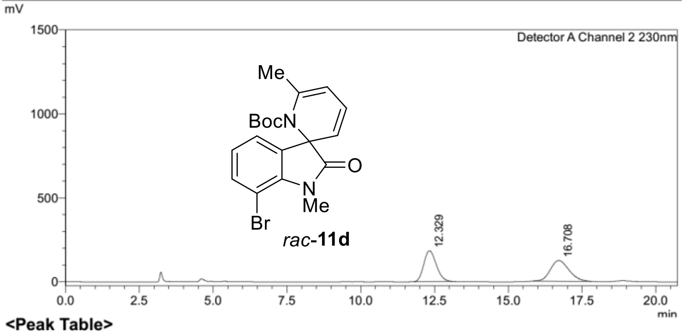

<Peak Table>

Detector A Channel 2 230nm

Peak#	Ret. Time	Area	Height	Conc.
1	12.329	5441937	183707	50.187
2	16.708	5401303	123143	49.813
Total		10843240	306850	

Supplementary Figure 218. HPLC analysis for compound **11d**

Analysis Report

<Sample Information>

Sample Name	: cuy-cc-123-2-01-rac-adh-99-1-1.0		
Sample ID	:		
Data Filename	: cuy-cc-123-2-01-asy-adh-99-1-1.1.lcd		
Method Filename	: WAC-93-FANFA.lcm		
Batch Filename	:		
Vial #	: 1-1	Sample Type	: Unknown
Injection Volume	: 20 uL		
Date Acquired	: 2019/6/28 16:29:49	Acquired by	: System Administrator
Date Processed	: 2019/6/28 17:07:09	Processed by	: System Administrator

<Chromatogram>

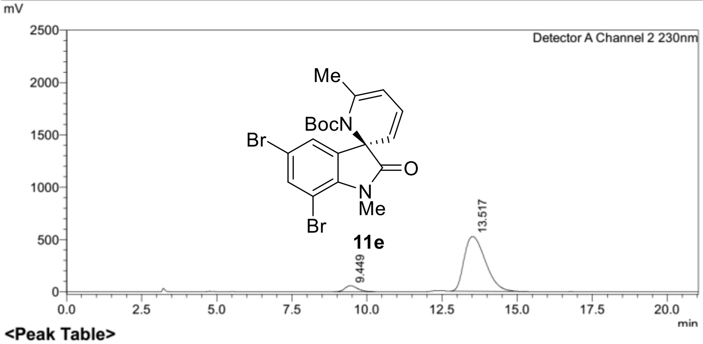

<Peak Table>

Detector A Channel 2 230nm

Peak#	Ret. Time	Area	Height	Conc.
1	9.449	1708936	57644	6.194
2	13.517	25882630	524618	93.806
Total		27591566	582262	

Analysis Report

<Sample Information>

Sample Name	: cuy-cc-122-3-03-rac-adh-99-1-1.0		
Sample ID	:		
Data Filename	: cuy-cc-122-3-03-rac-adh-99-1-1.1.lcd		
Method Filename	: WAC-93-FANFA.lcm		
Batch Filename	:		
Vial #	: 1-1	Sample Type	: Unknown
Injection Volume	: 20 uL		
Date Acquired	: 2019/6/28 16:52:02	Acquired by	: System Administrator
Date Processed	: 2019/6/28 17:12:22	Processed by	: System Administrator

<Chromatogram>

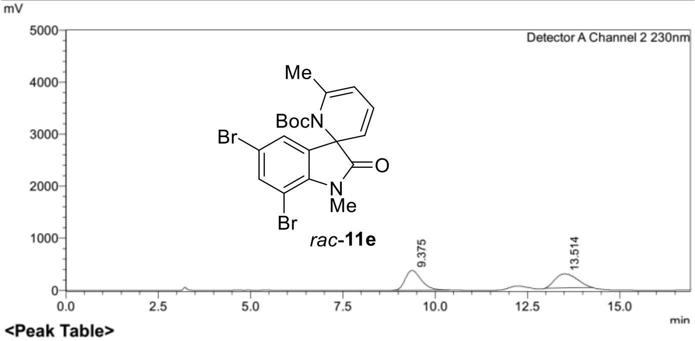

<Peak Table>

Detector A Channel 2 230nm

Peak#	Ret. Time	Area	Height	Conc.
1	9.375	11372053	377982	50.150
2	13.514	11303842	270887	49.850
Total		22675895	648869	

Supplementary Figure 219. HPLC analysis for compound **11e**

Analysis Report

<Sample Information>

Data Filename : mbs-me-89-adh-asy-99.5-0.5-1.0ml1.lcd
Method Filename : WAC-93-FANFA.lcm
Batch Filename :
Vial # : 1-1
Injection Volume : 20 uL
Date Acquired : 2019/8/24 10:50:20
Date Processed : 2019/8/24 10:58:00
Sample Type : Unknown
Acquired by : System Administrator
Processed by : System Administrator

<Chromatogram>

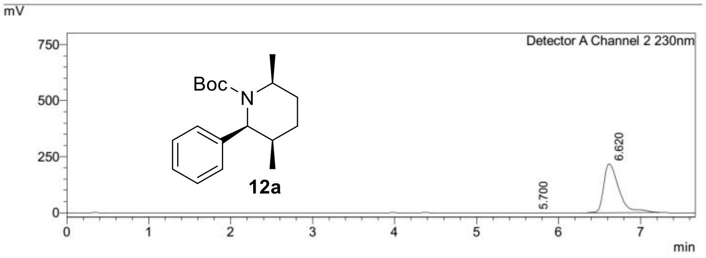

<Peak Table>

Detector A Channel 2 230nm

Peak#	Ret. Time	Area	Height	Conc.
1	5.700	6511	739	0.231
2	6.620	2808386	217408	99.769
Total		2814898	218147	

Analysis Report

<Sample Information>

Data Filename : mbs-mf-45-adh-rac-99.5-0.5-1.0ml2.lcd
Method Filename : WAC-93-FANFA.lcm
Batch Filename :
Vial # : 1-1
Injection Volume : 20 uL
Date Acquired : 2019/8/24 10:41:11
Date Processed : 2019/8/24 10:48:59
Sample Type : Unknown
Acquired by : System Administrator
Processed by : System Administrator

<Chromatogram>

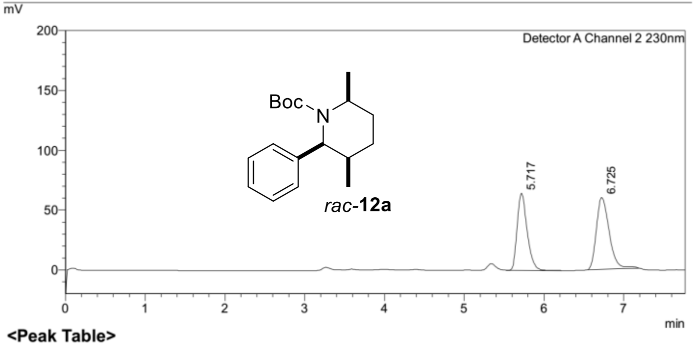

<Peak Table>

Detector A Channel 2 230nm

Peak#	Ret. Time	Area	Height	Conc.
1	5.717	557258	64214	45.398
2	6.725	670244	59896	54.602
Total		1227502	124110	

Supplementary Figure 220. HPLC analysis for compound **12a**

Analysis Report

<Sample Information>

Data Filename	: mbs-mh-38-asy-adh99.5-0.5-1.0ml3.lcd		
Method Filename	: 205 and 254nm.lcm		
Batch Filename	:		
Vial #	: 1-1	Sample Type	: Unknown
Injection Volume	: 20 uL		
Date Acquired	: 2020/5/9 21:37:11	Acquired by	: System Administrator
Date Processed	: 2020/5/9 21:57:50	Processed by	: System Administrator

<Chromatogram>

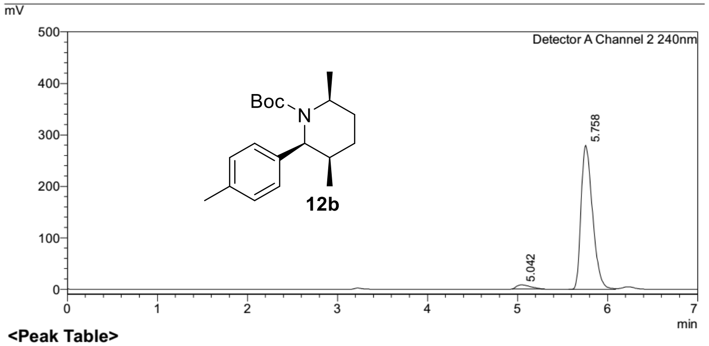

<Peak Table>

Detector A Channel 2 240nm

Peak#	Ret. Time	Area	Height	Conc.
1	5.042	90641	8233	3.513
2	5.758	2489696	279681	96.487
Total		2580337	287914	

Analysis Report

<Sample Information>

Data Filename	: mbs-mh-38-rac-adh-99.5-0.5-1.0ml3.lcd		
Method Filename	: 205 and 254nm.lcm		
Batch Filename	:		
Vial #	: 1-1	Sample Type	: Unknown
Injection Volume	: 20 uL		
Date Acquired	: 2020/5/9 21:05:04	Acquired by	: System Administrator
Date Processed	: 2020/5/10 15:51:58	Processed by	: System Administrator

<Chromatogram>

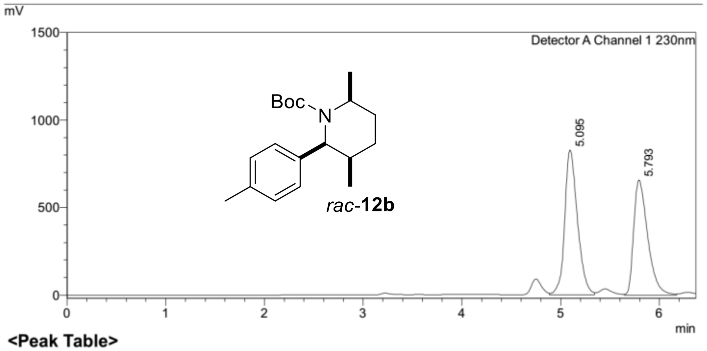

<Peak Table>

Detector A Channel 1 230nm

Peak#	Ret. Time	Area	Height	Conc.
1	5.095	7432264	827494	53.397
2	5.793	6486503	657551	46.603
Total		13918768	1485045	

Supplementary Figure 221. HPLC analysis for compound **12b**

Analysis Report

<Sample Information>

Data Filename	: mbs-me-95-asy-adh-99.5-0.5-1.0ml-3.lcd		
Method Filename	: WAC-93-FANFA.lcm		
Batch Filename	:		
Vial #	: 1-1	Sample Type	: Unknown
Injection Volume	: 20 uL		
Date Acquired	: 2019/8/15 10:58:02	Acquired by	: System Administrator
Date Processed	: 2019/8/15 11:06:42	Processed by	: System Administrator

<Chromatogram>

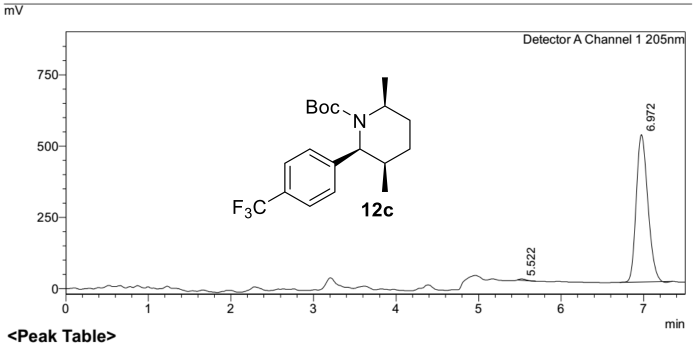

<Peak Table>

Detector A Channel 1 205nm

Peak#	Ret. Time	Area	Height	Conc.
1	5.522	24877	4729	0.490
2	6.972	5055434	515865	99.510
Total		5080310	520594	

Analysis Report

<Sample Information>

Data Filename	: mbs-mf-26-rac-adh-99.5-0.5-1.0ml-3.lcd		
Method Filename	: WAC-93-FANFA.lcm		
Batch Filename	:		
Vial #	: 1-1	Sample Type	: Unknown
Injection Volume	: 20 uL		
Date Acquired	: 2019/8/15 11:11:33	Acquired by	: System Administrator
Date Processed	: 2019/8/15 11:20:00	Processed by	: System Administrator

<Chromatogram>

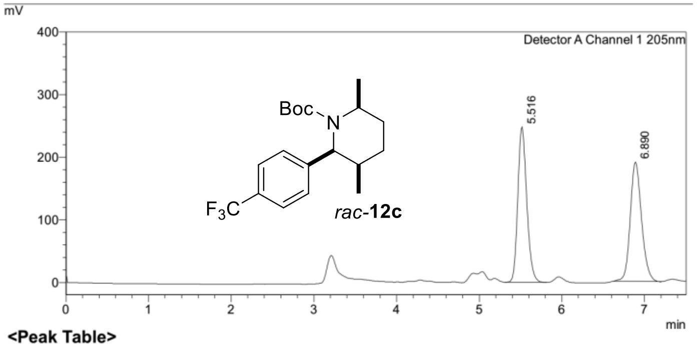

<Peak Table>

Detector A Channel 1 205nm

Peak#	Ret. Time	Area	Height	Conc.
1	5.516	1876381	247977	50.906
2	6.890	1809576	190115	49.094
Total		3685956	438092	

Supplementary Figure 222. HPLC analysis for compound **12c**

Analysis Report

<Sample Information>

Data Filename	: mbs-me-104-adh-asy-99.5-0.5-1.0ml1.lcd		
Method Filename	: 20170113.lcm		
Batch Filename	:		
Vial #	: 1-1	Sample Type	: Unknown
Injection Volume	: 20 uL		
Date Acquired	: 2019/8/31 9:49:11	Acquired by	: System Administrator
Date Processed	: 2019/8/31 9:56:46	Processed by	: System Administrator

<Chromatogram>

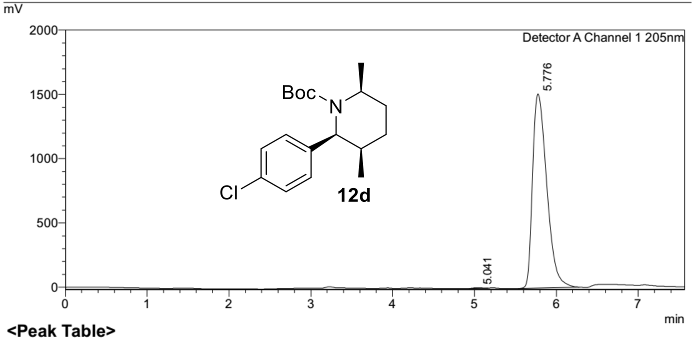

<Peak Table>

Detector A Channel 1 205nm

Peak#	Ret. Time	Area	Height	Conc.
1	5.041	36619	5347	0.202
2	5.776	18124862	1509303	99.798
Total		18161481	1514650	

Analysis Report

<Sample Information>

Data Filename	: mbs-mf-65-adh-rac-99.5-0.5-1.0ml2.lcd		
Method Filename	: 20170113.lcm		
Batch Filename	:		
Vial #	: 1-1	Sample Type	: Unknown
Injection Volume	: 20 uL		
Date Acquired	: 2019/8/31 9:05:01	Acquired by	: System Administrator
Date Processed	: 2019/8/31 9:57:08	Processed by	: System Administrator

<Chromatogram>

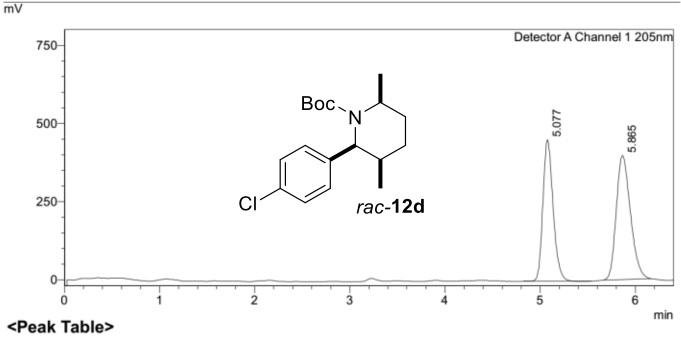

<Peak Table>

Detector A Channel 1 205nm

Peak#	Ret. Time	Area	Height	Conc.
1	5.077	3358360	451681	45.066
2	5.865	4093725	395963	54.934
Total		7452085	847645	

Supplementary Figure 223. HPLC analysis for compound **12d**

Analysis Report

<Sample Information>

Data Filename	: mbs-me-105-adh-asy-99.5-0.5-1.0ml1.lcd		
Method Filename	: 20170113.lcm		
Batch Filename	:		
Vial #	: 1-1	Sample Type	: Unknown
Injection Volume	: 20 uL		
Date Acquired	: 2019/8/31 10:53:35	Acquired by	: System Administrator
Date Processed	: 2019/8/31 11:00:47	Processed by	: System Administrator

<Chromatogram>

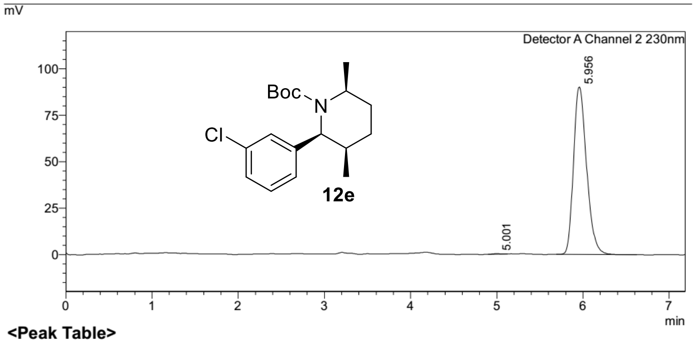

<Peak Table>

Detector A Channel 2 230nm

Peak#	Ret. Time	Area	Height	Conc.
1	5.001	1815	321	0.191
2	5.956	948403	90203	99.809
Total		950218	90523	

Analysis Report

<Sample Information>

Data Filename	: mbs-mf-66-adh-rac-99.5-0.5-1.0ml1.lcd		
Method Filename	: 20170113.lcm		
Batch Filename	:		
Vial #	: 1-1	Sample Type	: Unknown
Injection Volume	: 20 uL		
Date Acquired	: 2019/8/31 10:43:20	Acquired by	: System Administrator
Date Processed	: 2019/8/31 10:50:22	Processed by	: System Administrator

<Chromatogram>

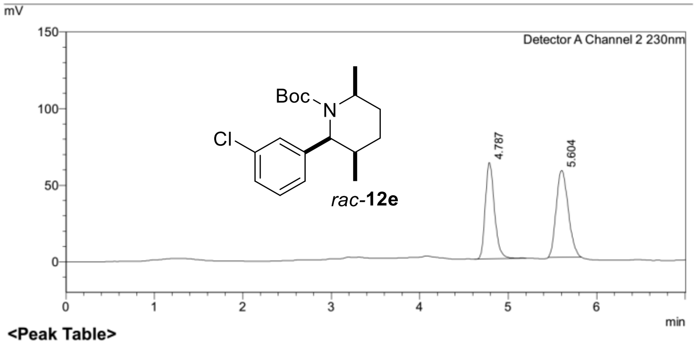

<Peak Table>

Detector A Channel 2 230nm

Peak#	Ret. Time	Area	Height	Conc.
1	4.787	453688	62734	45.733
2	5.604	538359	56700	54.267
Total		992047	119434	

Supplementary Figure 224. HPLC analysis for compound **12e**

Analysis Report

<Sample Information>

Data Filename : mbs-me-106-asy-ODH-99.8-0.2-0.8ml3.lcd
Method Filename : 205 and 254nm.lcm
Batch Filename :
Vial # : 1-1
Sample Type : Unknown
Injection Volume : 20 uL
Date Acquired : 2020/5/8 20:34:42
Acquired by : System Administrator
Date Processed : 2020/5/8 20:51:52
Processed by : System Administrator

<Chromatogram>

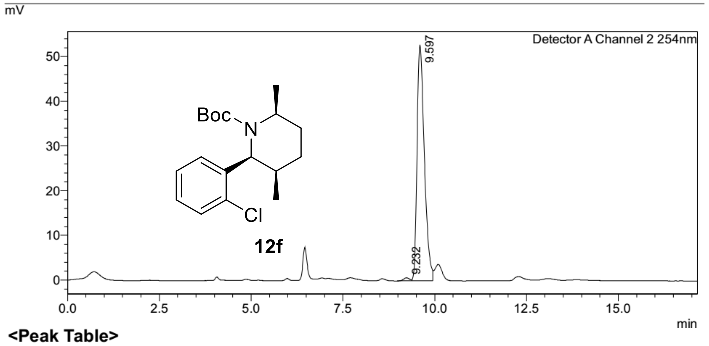

<Peak Table>

Detector A Channel 2 254nm

Peak#	Ret. Time	Area	Height	Conc.
1	9.232	7457	684	1.011
2	9.597	730398	52822	98.989
Total		737856	53505	

Analysis Report

<Sample Information>

Data Filename : mbs-me-106-rac-ODH-99.8-0.2-0.8ml2.lcd
Method Filename : 205 and 254nm.lcm
Batch Filename :
Vial # : 1-1
Sample Type : Unknown
Injection Volume : 20 uL
Date Acquired : 2020/5/8 19:48:26
Acquired by : System Administrator
Date Processed : 2020/5/8 19:59:52
Processed by : System Administrator

<Chromatogram>

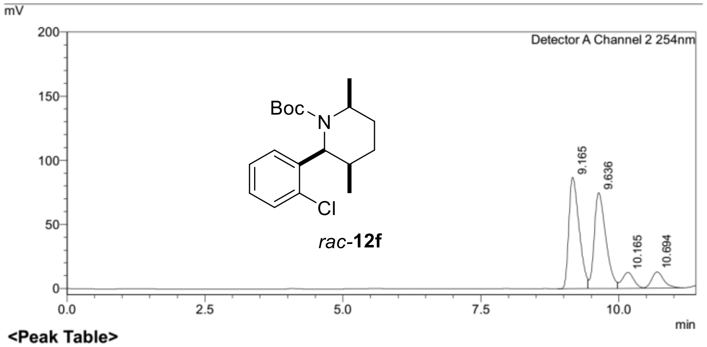

<Peak Table>

Detector A Channel 2 254nm

Peak#	Ret. Time	Area	Height	Conc.
1	9.165	1136319	86925	43.609
2	9.636	1095584	74797	42.046
3	10.165	183754	12459	7.052
4	10.694	190025	12630	7.293
Total		2605682	186812	

Supplementary Figure 225. HPLC analysis for compound **12f**

Analysis Report

<Sample Information>

Data Filename	: mbs-me-96-adh-asy-99.5-0.5-1.0ml1.lcd		
Method Filename	: 20170113.lcm		
Batch Filename	:		
Vial #	: 1-1	Sample Type	: Unknown
Injection Volume	: 20 uL		
Date Acquired	: 2019/8/31 11:16:11	Acquired by	: System Administrator
Date Processed	: 2019/8/31 11:24:04	Processed by	: System Administrator

<Chromatogram>

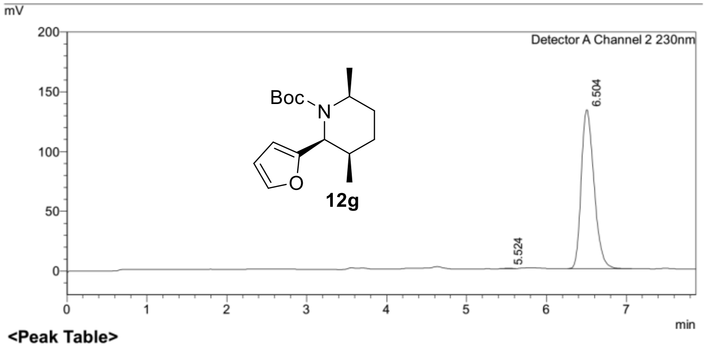

<Peak Table>

Detector A Channel 2 230nm

Peak#	Ret. Time	Area	Height	Conc.
1	5.524	2303	380	0.155
2	6.504	1483026	132962	99.845
Total		1485329	133342	

Analysis Report

<Sample Information>

Data Filename	: mbs-mf-68-adh-rac-99.5-0.5-1.0ml1.lcd		
Method Filename	: 20170113.lcm		
Batch Filename	:		
Vial #	: 1-1	Sample Type	: Unknown
Injection Volume	: 20 uL		
Date Acquired	: 2019/8/31 11:06:07	Acquired by	: System Administrator
Date Processed	: 2019/8/31 11:14:25	Processed by	: System Administrator

<Chromatogram>

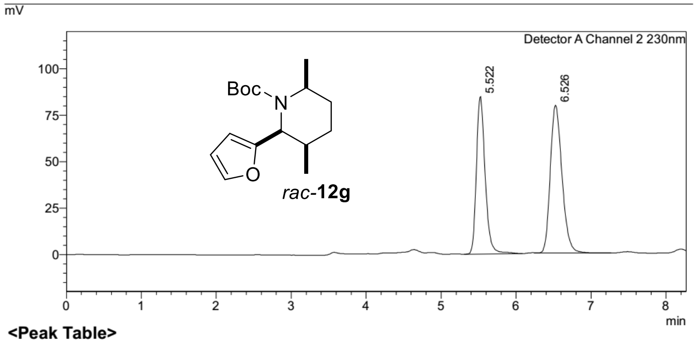

<Peak Table>

Detector A Channel 2 230nm

Peak#	Ret. Time	Area	Height	Conc.
1	5.522	691234	84752	44.519
2	6.526	861444	79448	55.481
Total		1552679	164201	

Supplementary Figure 226. HPLC analysis for compound **12g**

Analysis Report

<Sample Information>

Data Filename	: mbs-me-97-adh-asy-99.5-0.5-1.0ml1.lcd		
Method Filename	: WAC-93-FANFA.lcm		
Batch Filename	:		
Vial #	: 1-1	Sample Type	: Unknown
Injection Volume	: 20 uL		
Date Acquired	: 2019/8/24 11:29:42	Acquired by	: System Administrator
Date Processed	: 2019/8/24 11:37:49	Processed by	: System Administrator

<Chromatogram>

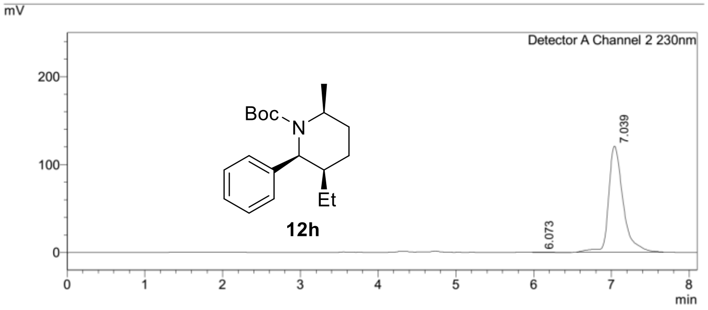

<Peak Table>

Detector A Channel 2 230nm

Peak#	Ret. Time	Area	Height	Conc.
1	6.073	7361	526	0.473
2	7.039	1549719	120467	99.527
Total		1557080	120994	

Analysis Report

<Sample Information>

Data Filename	: mbs-mf-46-adh-rac-99.5-0.5-1.0ml-true3.lcd		
Method Filename	: WAC-93-FANFA.lcm		
Batch Filename	:		
Vial #	: 1-1	Sample Type	: Unknown
Injection Volume	: 20 uL		
Date Acquired	: 2019/8/24 11:14:40	Acquired by	: System Administrator
Date Processed	: 2019/8/24 11:27:07	Processed by	: System Administrator

<Chromatogram>

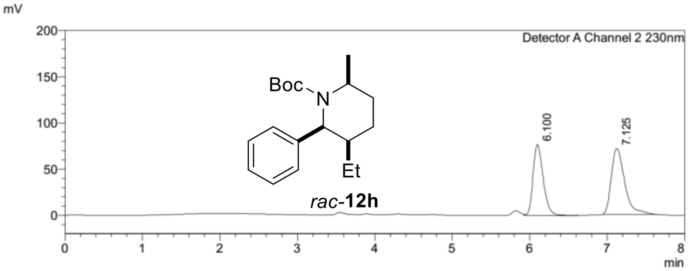

<Peak Table>

Detector A Channel 2 230nm

Peak#	Ret. Time	Area	Height	Conc.
1	6.100	703640	77031	45.359
2	7.125	847614	71210	54.641
Total		1551254	148241	

Supplementary Figure 227. HPLC analysis for compound **12h**

Operator:dell Timebase:U-3000 Sequence:Mu Boshuai

Page 1-1
2020-11-28 10:43 上午

311 mbs-me-93-asy-ash-99.5/0.5-210-1.0ml1

Sample Name:	**mbs-me-93-asy-ash-99.5/0.5-210-1.0ml1**	*Injection Volume:*	**20.0**
Vial Number:	**858**	*Channel:*	**UV_VIS_1**
Sample Type:	**standard**	*Wavelength:*	**210**
Control Program:	**Mu Boshuai**	*Bandwidth:*	**n.a.**
Quantif. Method:	**Mu Boshuai**	*Dilution Factor:*	**1.0000**
Run Time (min):	**12.39**	*Sample Amount:*	**1.0000**

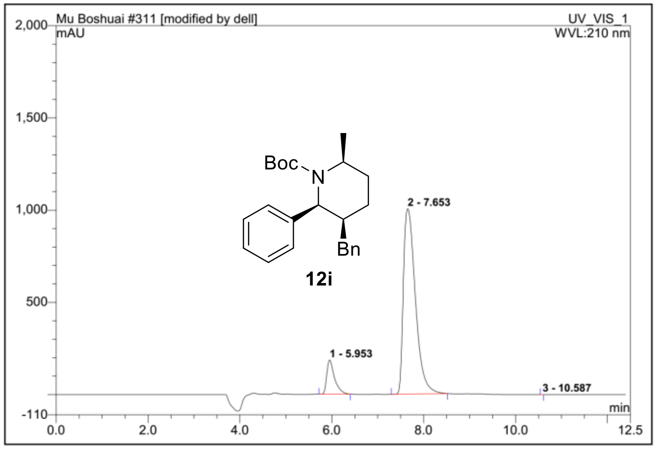

No.	Ret.Time min	Peak Name	Height mAU	Area mAU*min	Rel.Area %	Amount	Type
1	5.95	n.a.	185.125	36.073	10.61	n.a.	BMB*
2	7.65	n.a.	1005.400	303.993	89.39	n.a.	BMB*
3	10.59	n.a.	0.008	0.000	0.00	n.a.	BMB*
Total:			1190.532	340.066	100.00	0.000	

Mu/Integration

Chromeleon (c) Dionex 1996-2006
Version 6.80 SR8a Build 2643 (158225)

Operator:dell Timebase:U-3000 Sequence:Mu Boshuai

Page 1-1
2020-11-11 2:27 下午

310 mbs-mf-63-rac-ash-99.5/0.5-210-1.0ml7

Sample Name:	**mbs-mf-63-rac-ash-99.5/0.5-210-1.0ml7**	*Injection Volume:*	**20.0**
Vial Number:	**854**	*Channel:*	**UV_VIS_1**
Sample Type:	**standard**	*Wavelength:*	**210**
Control Program:	**Mu Boshuai**	*Bandwidth:*	**n.a.**
Quantif. Method:	**Mu Boshuai**	*Dilution Factor:*	**1.0000**
Run Time (min):	**13.59**	*Sample Amount:*	**1.0000**

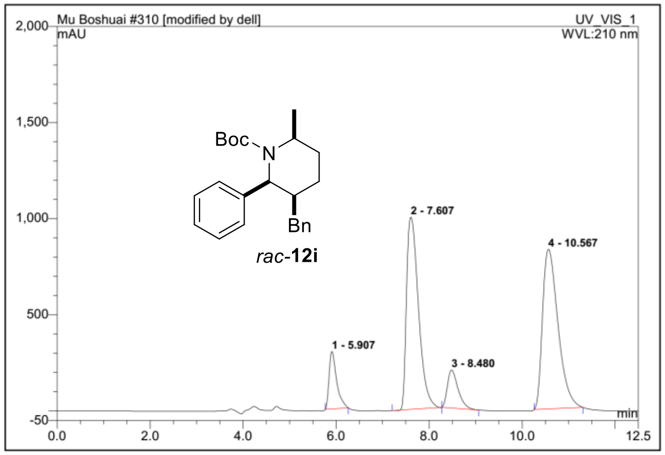

No.	Ret.Time min	Peak Name	Height mAU	Area mAU*min	Rel.Area %	Amount	Type
1	5.91	n.a.	299.083	56.328	7.75	n.a.	BMB*
2	7.61	n.a.	1000.129	296.702	40.84	n.a.	BMb*
3	8.48	n.a.	198.108	52.638	7.25	n.a.	bMB*
4	10.57	n.a.	830.804	320.762	44.16	n.a.	BMB*
Total:			2328.124	726.429	100.00	0.000	

Mu/Integration

Chromeleon (c) Dionex 1996-2006
Version 6.80 SR8a Build 2643 (158225)

Supplementary Figure 228. HPLC analysis for compound **12i**

Analysis Report

<Sample Information>

Data Filename	: mbs-me-101-IA-asy-99.5-0.5-1ml1.lcd		
Method Filename	: WAC-93-FANFA.lcm		
Batch Filename	:		
Vial #	: 1-1	Sample Type	: Unknown
Injection Volume	: 20 uL		
Date Acquired	: 2019/9/23 19:06:37	Acquired by	: System Administrator
Date Processed	: 2019/9/23 19:14:38	Processed by	: System Administrator

<Chromatogram>

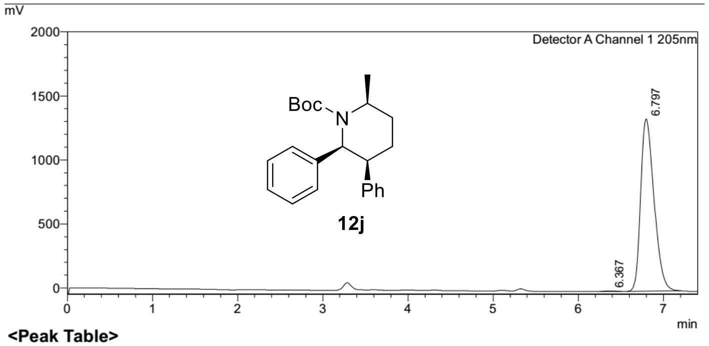

<Peak Table>

Detector A Channel 1 205nm

Peak#	Ret. Time	Area	Height	Conc.
1	6.367	28951	3841	0.195
2	6.797	14801641	1343945	99.805
Total		14830592	1347786	

Analysis Report

<Sample Information>

Data Filename	: mbs-mf-82-IA-asy-99.5-0.5-1mlnew.1.lcd		
Method Filename	: WAC-93-FANFA.lcm		
Batch Filename	:		
Vial #	: 1-1	Sample Type	: Unknown
Injection Volume	: 20 uL		
Date Acquired	: 2019/9/23 18:26:22	Acquired by	: System Administrator
Date Processed	: 2019/9/23 18:33:58	Processed by	: System Administrator

<Chromatogram>

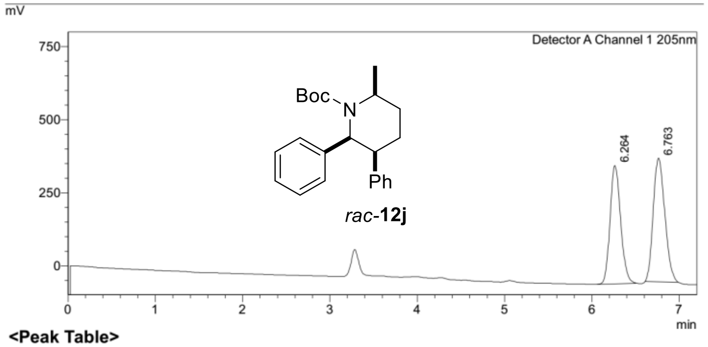

<Peak Table>

Detector A Channel 1 205nm

Peak#	Ret. Time	Area	Height	Conc.
1	6.264	3406514	404445	46.498
2	6.763	3919689	423344	53.502
Total		7326203	827789	

Supplementary Figure 229. HPLC analysis for compound **12j**

Analysis Report

<Sample Information>

Data Filename	: mbs-cxy-last-asy-ia-99.5-0.5		
Method Filename	: 205 and 254nm.lcm		
Batch Filename	:		
Vial #	: 1-1	Sample Type	: Unknown
Injection Volume	: 20 uL		
Date Acquired	: 2020/6/18 22:00:11	Acquired by	: System Administrator
Date Processed	: 2020/6/18 22:22:32	Processed by	: System Administrator

<Chromatogram>

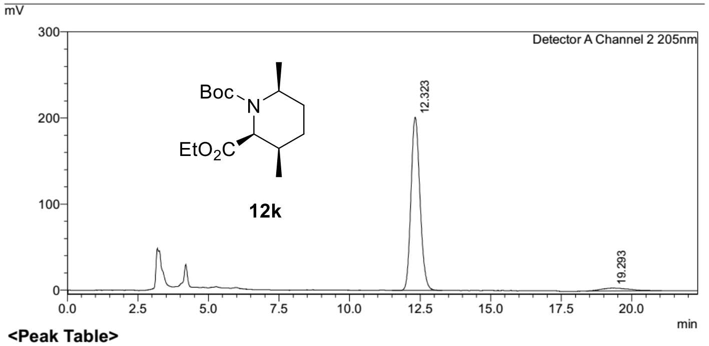

<Peak Table>

Detector A Channel 2 205nm

Peak#	Ret. Time	Area	Height	Conc.
1	12.323	4471260	201277	95.197
2	19.293	225568	3320	4.803
Total		4696828	204598	

Analysis Report

<Sample Information>

Data Filename	: mbs-cxy-last-rac-ia-99.5-0.5		
Method Filename	: 205 and 254nm.lcm		
Batch Filename	:		
Vial #	: 1-1	Sample Type	: Unknown
Injection Volume	: 20 uL		
Date Acquired	: 2020/6/18 21:06:34	Acquired by	: System Administrator
Date Processed	: 2020/6/18 21:28:58	Processed by	: System Administrator

<Chromatogram>

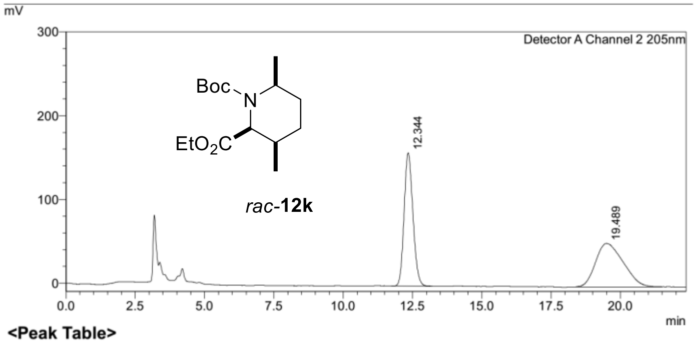

<Peak Table>

Detector A Channel 2 205nm

Peak#	Ret. Time	Area	Height	Conc.
1	12.344	3524631	159102	49.501
2	19.489	3595758	51953	50.499
Total		7120389	211055	

Supplementary Figure 230. HPLC analysis for compound **12k**

Analysis Report

<Sample Information>

Data Filename : mbs-mf-69-asy-IA-99.5-0.5-1ml.lcd
Method Filename : 278-254nm.lcm
Batch Filename :
Vial # : 1-1 Sample Type : Unknown
Injection Volume : 20 uL
Date Acquired : 2020/1/13 10:44:34 Acquired by : System Administrator
Date Processed : 2020/5/8 14:48:22 Processed by : System Administrator

<Chromatogram>

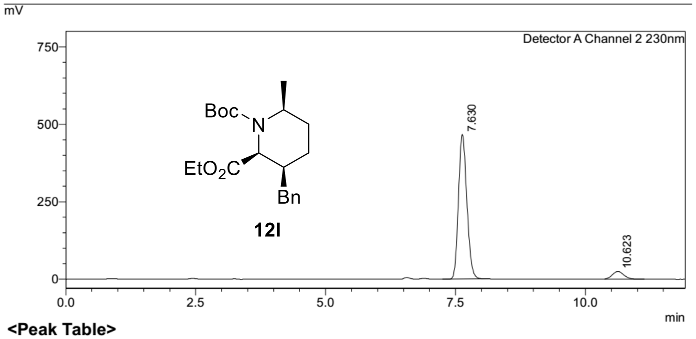

<Peak Table>

Detector A Channel 2 230nm

Peak#	Ret. Time	Area	Height	Conc.
1	7.630	5268739	466966	93.273
2	10.623	379979	24588	6.727
Total		5648717	491554	

Analysis Report

<Sample Information>

Data Filename : mbs-mf-69-rac-IA-99.5-0.5-1.0ml1.lcd
Method Filename : 278-254nm.lcm
Batch Filename :
Vial # : 1-1 Sample Type : Unknown
Injection Volume : 20 uL
Date Acquired : 2020/1/13 9:59:24 Acquired by : System Administrator
Date Processed : 2020/4/29 11:37:27 Processed by : System Administrator

<Chromatogram>

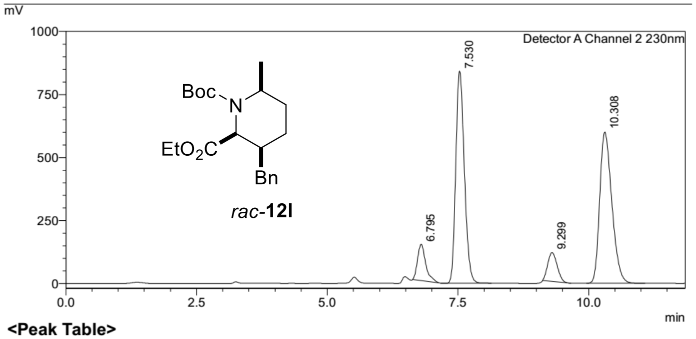

<Peak Table>

Detector A Channel 2 230nm

Peak#	Ret. Time	Area	Height	Conc.
1	6.795	1564227	143774	7.005
2	7.530	9614397	841621	43.059
3	9.299	1448903	114931	6.489
4	10.308	9701111	599657	43.447
Total		22328637	1699983	

Supplementary Figure 231. HPLC analysis for compound **12l**

Analysis Report

<Sample Information>

Data Filename	: mbs-me-100-IC-asy-99.5-0.5-1.0ml-3.lcd		
Method Filename	: WAC-93-FANFA.lcm		
Batch Filename	:		
Vial #	: 1-1	Sample Type	: Unknown
Injection Volume	: 20 uL		
Date Acquired	: 2019/9/19 16:03:35	Acquired by	: System Administrator
Date Processed	: 2019/9/19 16:19:34	Processed by	: System Administrator

<Chromatogram>

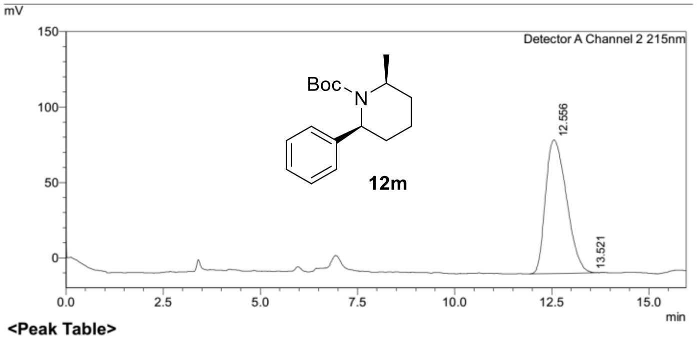

<Peak Table>

Detector A Channel 2 215nm

Peak#	Ret. Time	Area	Height	Conc.
1	12.556	3330099	88671	99.919
2	13.521	2713	554	0.081
Total		3332811	89225	

Analysis Report

<Sample Information>

Data Filename	: mbs-mf-84-IC-asy-99.5-0.5-1.0ml-3.lcd		
Method Filename	: WAC-93-FANFA.lcm		
Batch Filename	:		
Vial #	: 1-1	Sample Type	: Unknown
Injection Volume	: 20 uL		
Date Acquired	: 2019/9/19 15:04:43	Acquired by	: System Administrator
Date Processed	: 2019/9/19 15:20:05	Processed by	: System Administrator

<Chromatogram>

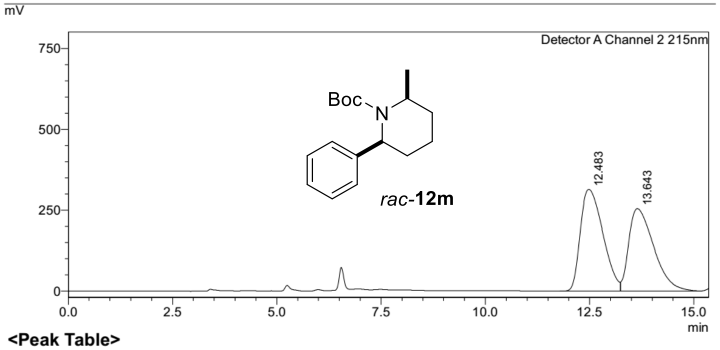

<Peak Table>

Detector A Channel 2 215nm

Peak#	Ret. Time	Area	Height	Conc.
1	12.483	11839454	314615	52.814
2	13.643	10577721	254861	47.186
Total		22417175	569476	

Supplementary Figure 232. HPLC analysis for compound **12m**

Analysis Report

<Sample Information>

Data Filename : mbs-cxy-fruan-asy-IC-9.5-0.5-1ml4.lcd
Method Filename : 205 and 254nm.lcm
Batch Filename :
Vial # : 1-1 Sample Type : Unknown
Injection Volume : 20 uL
Date Acquired : 2020/5/8 16:11:13 Acquired by : System Administrator
Date Processed : 2020/5/8 16:20:15 Processed by : System Administrator

<Chromatogram>

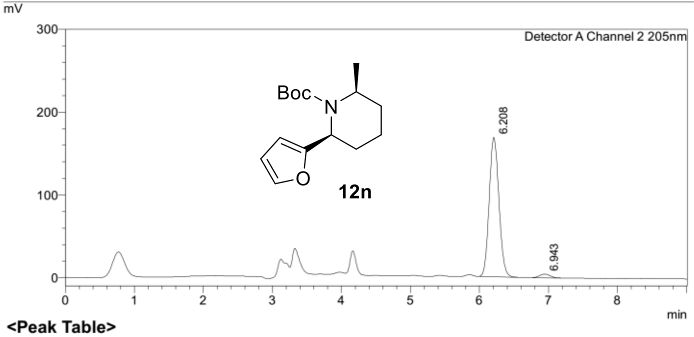

<Peak Table>

Detector A Channel 2 205nm

Peak#	Ret. Time	Area	Height	Conc.
1	6.208	1631102	168125	97.303
2	6.943	45218	4328	2.697
Total		1676320	172453	

Analysis Report

<Sample Information>

Data Filename : mbs-cxy-fruan-rac-IC-9.5-0.5-1ml3.lcd
Method Filename : 205 and 254nm.lcm
Batch Filename :
Vial # : 1-1 Sample Type : Unknown
Injection Volume : 20 uL
Date Acquired : 2020/5/8 15:48:34 Acquired by : System Administrator
Date Processed : 2020/5/8 15:56:09 Processed by : System Administrator

<Chromatogram>

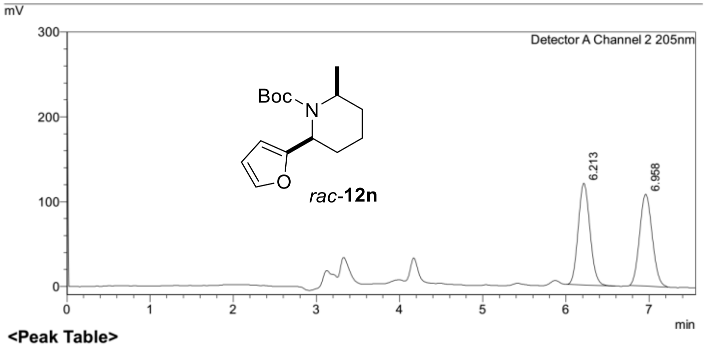

<Peak Table>

Detector A Channel 2 205nm

Peak#	Ret. Time	Area	Height	Conc.
1	6.213	1174484	119917	50.841
2	6.958	1135623	108252	49.159
Total		2310107	228169	

Supplementary Figure 233. HPLC analysis for compound **12n**

Analysis Report

<Sample Information>

Data Filename	: mbs-mf-15-odh-asy-99.5-0.5-1.0ml-4.lcd		
Method Filename	: WAC-93-FANFA.lcm		
Batch Filename	:		
Vial #	: 1-1	Sample Type	: Unknown
Injection Volume	: 20 uL		
Date Acquired	: 2019/8/24 16:01:34	Acquired by	: System Administrator
Date Processed	: 2019/8/24 16:11:07	Processed by	: System Administrator

<Chromatogram>

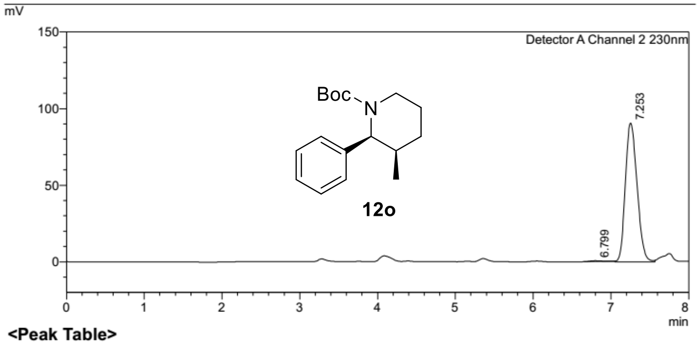

<Peak Table>

Detector A Channel 2 230nm

Peak#	Ret. Time	Area	Height	Conc.
1	6.799	4547	510	0.476
2	7.253	949830	90590	99.524
Total		954377	91100	

Analysis Report

<Sample Information>

Data Filename	: mbs-mf-47-odh-rac-99.5-0.5-1.0ml-4.lcd		
Method Filename	: WAC-93-FANFA.lcm		
Batch Filename	:		
Vial #	: 1-1	Sample Type	: Unknown
Injection Volume	: 20 uL		
Date Acquired	: 2019/8/24 15:37:26	Acquired by	: System Administrator
Date Processed	: 2019/8/24 15:47:45	Processed by	: System Administrator

<Chromatogram>

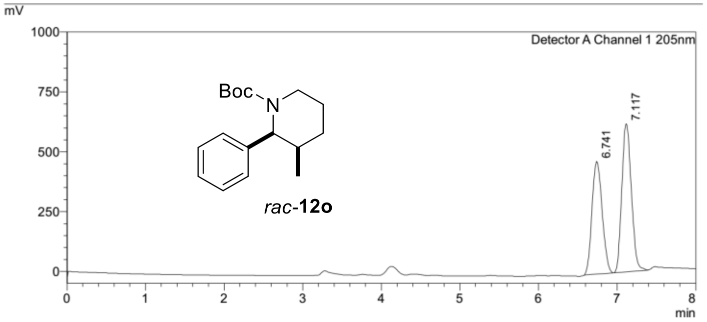

<Peak Table>

Detector A Channel 1 205nm

Peak#	Ret. Time	Area	Height	Conc.
1	6.741	4234813	470927	44.825
2	7.117	5212703	617993	55.175
Total		9447516	1088920	

Supplementary Figure 234. HPLC analysis for compound **12o**

Analysis Report

<Sample Information>

Data Filename	: mbs-mf-18-asy-IC-99.5-0.5-1ml8.lcd		
Method Filename	: 205 and 254nm.lcm		
Batch Filename	:		
Vial #	: 1-1	Sample Type	: Unknown
Injection Volume	: 20 uL		
Date Acquired	: 2020/5/7 19:52:58	Acquired by	: System Administrator
Date Processed	: 2020/5/7 20:04:21	Processed by	: System Administrator

<Chromatogram>

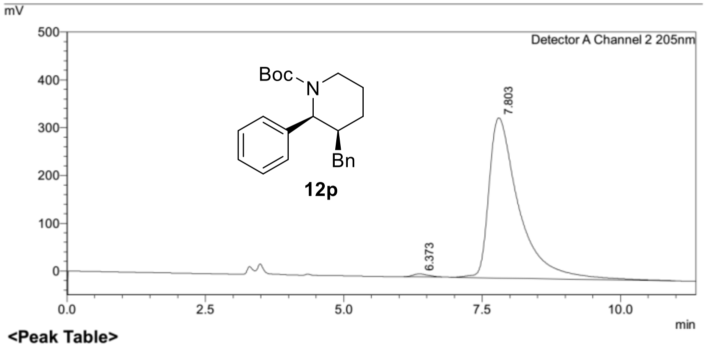

<Peak Table>

Detector A Channel 2 205nm

Peak#	Ret. Time	Area	Height	Conc.
1	6.373	116002	6010	0.906
2	7.803	12693120	335279	99.094
Total		12809123	341288	

Analysis Report

<Sample Information>

Data Filename	: mbs-mf-18-rac-IC-99.5-0.5-1ml7.lcd		
Method Filename	: 205 and 254nm.lcm		
Batch Filename	:		
Vial #	: 1-1	Sample Type	: Unknown
Injection Volume	: 20 uL		
Date Acquired	: 2020/5/7 19:33:04	Acquired by	: System Administrator
Date Processed	: 2020/5/7 19:50:23	Processed by	: System Administrator

<Chromatogram>

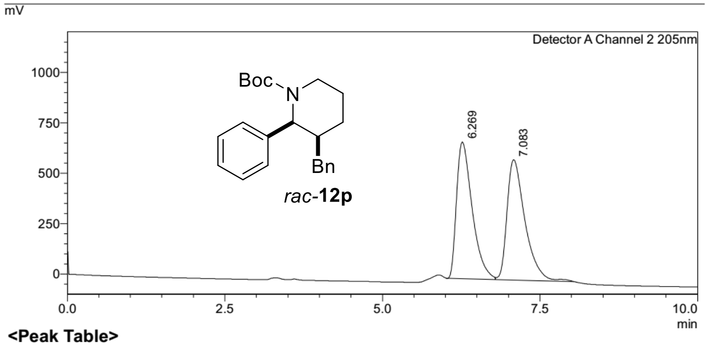

<Peak Table>

Detector A Channel 2 205nm

Peak#	Ret. Time	Area	Height	Conc.
1	6.269	11725113	676462	49.696
2	7.083	11868671	596246	50.304
Total		23593783	1272708	

Supplementary Figure 235. HPLC analysis for compound **12p**

Analysis Report

<Sample Information>

Data Filename	: mbs-me-134-asy-IC-99.5-0.5-1ml1.lcd		
Method Filename	: WAC-93-FANFA.lcm		
Batch Filename	:		
Vial #	: 1-1	Sample Type	: Unknown
Injection Volume	: 20 uL		
Date Acquired	: 2019/9/23 20:26:58	Acquired by	: System Administrator
Date Processed	: 2020/5/6 16:51:22	Processed by	: System Administrator

<Chromatogram>

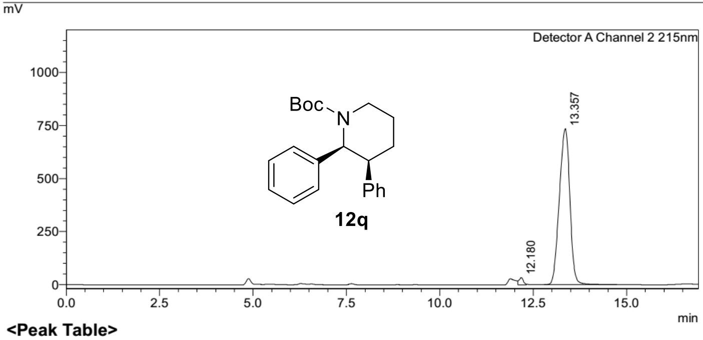

<Peak Table>

Detector A Channel 2 215nm

Peak#	Ret. Time	Area	Height	Conc.
1	12.180	275859	33695	1.891
2	13.357	14314260	734263	98.109
Total		14590119	767958	

Analysis Report

<Sample Information>

Data Filename	: mbs-mf-83-rac-IC-99.5-0.5-1mlnew.2.lcd		
Method Filename	: WAC-93-FANFA.lcm		
Batch Filename	:		
Vial #	: 1-1	Sample Type	: Unknown
Injection Volume	: 20 uL		
Date Acquired	: 2019/9/23 20:07:37	Acquired by	: System Administrator
Date Processed	: 2019/9/23 20:23:15	Processed by	: System Administrator

<Chromatogram>

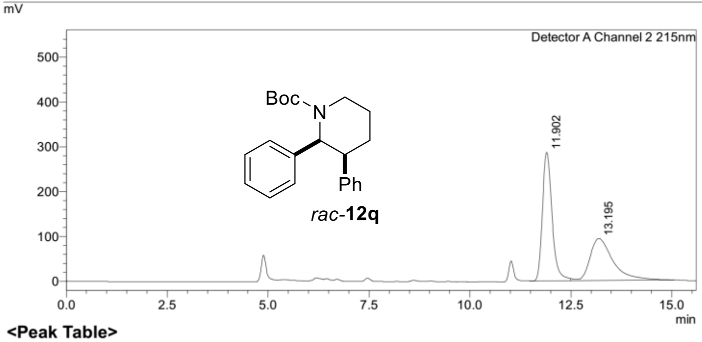

<Peak Table>

Detector A Channel 2 215nm

Peak#	Ret. Time	Area	Height	Conc.
1	11.902	4741063	286069	56.082
2	13.195	3712672	93233	43.918
Total		8453734	379302	

Supplementary Figure 236. HPLC analysis for compound **12q**

Analysis Report

<Sample Information>

Data Filename	: mbs-mf-14-ojh-asy-99.5-0.5-1.0ml-new1.lcd		
Method Filename	: WAC-93-FANFA.lcm		
Batch Filename	:		
Vial #	: 1-1	Sample Type	: Unknown
Injection Volume	: 20 uL		
Date Acquired	: 2019/9/18 15:13:21	Acquired by	: System Administrator
Date Processed	: 2019/9/18 15:22:18	Processed by	: System Administrator

<Chromatogram>

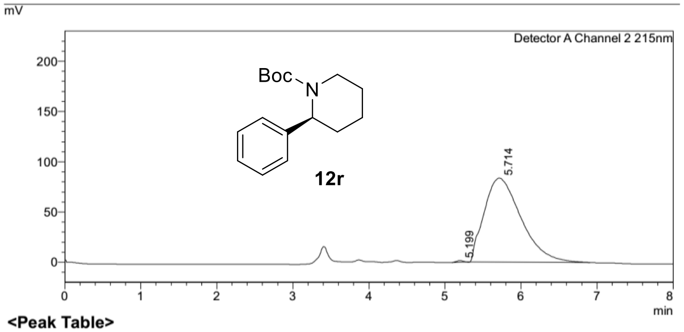

<Peak Table>

Detector A Channel 2 215nm

Peak#	Ret. Time	Area	Height	Conc.
1	5.199	11021	1754	0.387
2	5.714	2838167	83573	99.613
Total		2849189	85328	

Analysis Report

<Sample Information>

Data Filename	: mbs-mf-85-ojh-rac-99.5-0.5-1.0ml1.lcd		
Method Filename	: WAC-93-FANFA.lcm		
Batch Filename	:		
Vial #	: 1-1	Sample Type	: Unknown
Injection Volume	: 20 uL		
Date Acquired	: 2019/9/18 11:47:28	Acquired by	: System Administrator
Date Processed	: 2019/9/18 21:38:22	Processed by	: System Administrator

<Chromatogram>

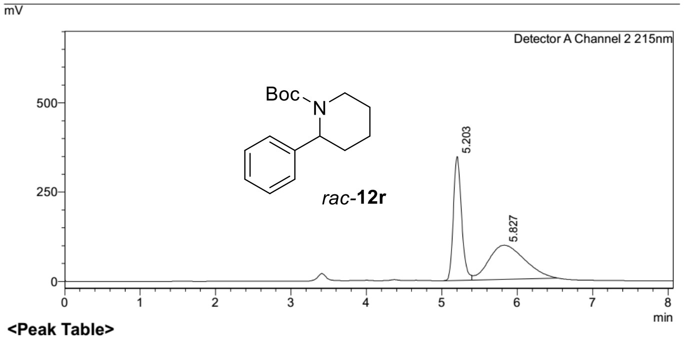

<Peak Table>

Detector A Channel 2 215nm

Peak#	Ret. Time	Area	Height	Conc.
1	5.203	2632899	347119	45.436
2	5.827	3161869	95336	54.564
Total		5794768	442456	

Supplementary Figure 237. HPLC analysis for compound **12r**

2020/11/12 9:53:07 Page 1 / 1

Analysis Report

<Sample Information>

Data Filename	: bm-ii-124-asy-adh-99.5-0.5-1.0ml		
Method Filename	: WAC-93-FANFA.lcm		
Batch Filename	:		
Vial #	: 1-1	Sample Type	: Unknown
Injection Volume	: 20 uL		
Date Acquired	: 2017/8/30 16:00:01	Acquired by	: System Administrator
Date Processed	: 2020/5/6 16:18:10	Processed by	: System Administrator

<Chromatogram>

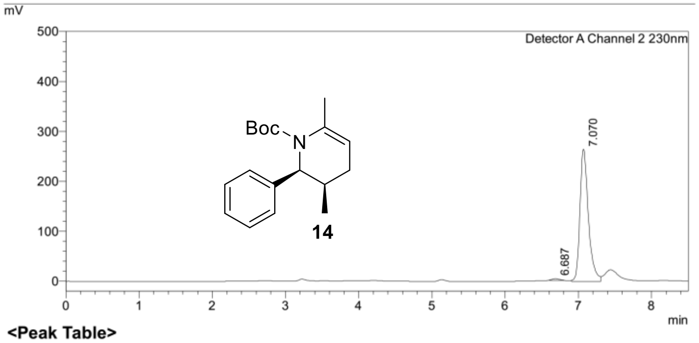

<Peak Table>

Detector A Channel 2 230nm

Peak#	Ret. Time	Area	Height	Conc.
1	6.687	25640	3326	1.190
2	7.070	2129747	264603	98.810
Total		2155387	267929	

2020/11/12 9:39:40 Page 1 / 1

Analysis Report

<Sample Information>

Data Filename	: bm-ii-124-rac-99.5-0.5-1.0ml		
Method Filename	: WAC-93-FANFA.lcm		
Batch Filename	:		
Vial #	: 1-1	Sample Type	: Unknown
Injection Volume	: 20 uL		
Date Acquired	: 2017/11/29 15:37:23	Acquired by	: System Administrator
Date Processed	: 2020/5/6 16:10:59	Processed by	: System Administrator

<Chromatogram>

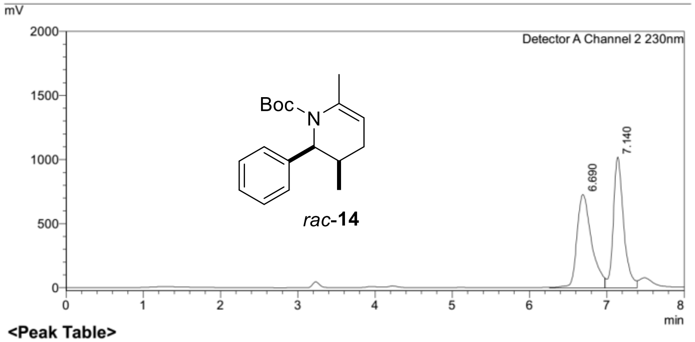

<Peak Table>

Detector A Channel 2 230nm

Peak#	Ret. Time	Area	Height	Conc.
1	6.690	9733647	730058	50.532
2	7.140	9528619	1022713	49.468
Total		19262266	1752771	

Supplementary Figure 238. HPLC analysis for compound **14**

309 mbs-mj-109-asy-adh-80/20-210-1.0ml6

Sample Name:	**mbs-mj-109-asy-adh-80/20-210-1.0ml6**	*Injection Volume:*	**20.0**
Vial Number:	**851**	*Channel:*	**UV_VIS_1**
Sample Type:	**standard**	*Wavelength:*	**210**
Control Program:	**Mu Boshuai**	*Bandwidth:*	**n.a.**
Quantif. Method:	**Mu Boshuai**	*Dilution Factor:*	**1.0000**
Recording Time:	**2020-11-8 17:09**	*Sample Weight:*	**1.0000**
Run Time (min):	**10.71**	*Sample Amount:*	**1.0000**

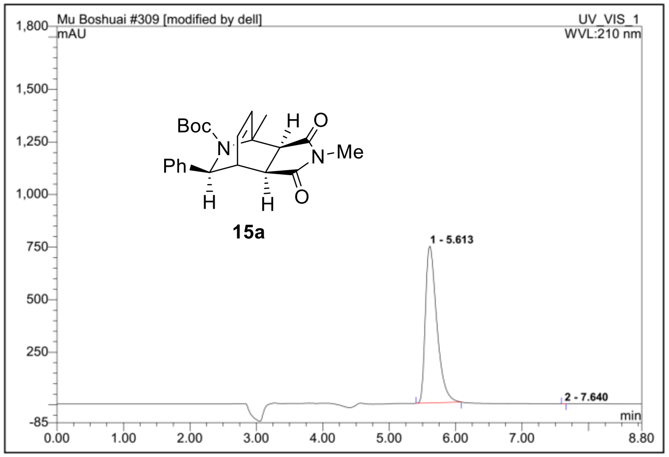

No.	Ret.Time min	Peak Name	Height mAU	Area mAU*min	Rel.Area %	Amount	Type
1	5.61	n.a.	745.233	149.014	100.00	n.a.	BMB*
2	7.64	n.a.	0.009	0.000	0.00	n.a.	BMB*
**Total:**			745.242	149.014	100.00	0.000	

308 mbs-mj-106-rac-adh-80/20-210-1.0ml6

Sample Name:	**mbs-mj-106-rac-adh-80/20-210-1.0ml6**	*Injection Volume:*	**20.0**
Vial Number:	**850**	*Channel:*	**UV_VIS_1**
Sample Type:	**standard**	*Wavelength:*	**210**
Control Program:	**Mu Boshuai**	*Bandwidth:*	**n.a.**
Quantif. Method:	**Mu Boshuai**	*Dilution Factor:*	**1.0000**
Recording Time:	**2020-11-8 16:40**	*Sample Weight:*	**1.0000**
Run Time (min):	**12.95**	*Sample Amount:*	**1.0000**

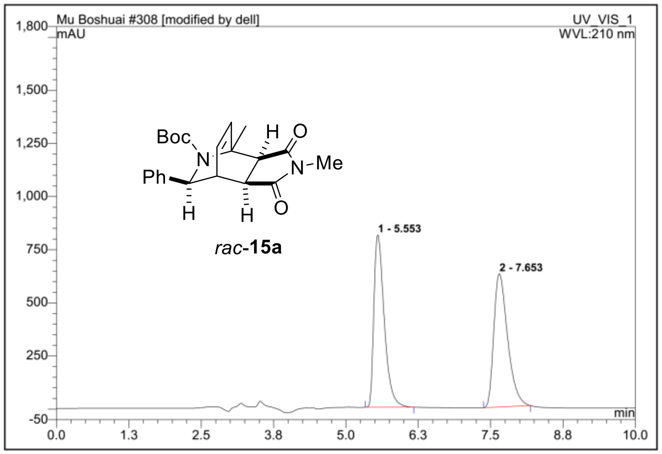

No.	Ret.Time min	Peak Name	Height mAU	Area mAU*min	Rel.Area %	Amount	Type
1	5.55	n.a.	810.627	168.427	49.24	n.a.	BMB*
2	7.65	n.a.	626.676	173.602	50.76	n.a.	BMB*
**Total:**			1437.303	342.029	100.00	0.000	

Supplementary Figure 239. HPLC analysis for compound **15a**

285 mbs-mj-107-asy-adh-80/20-254-1.0ml

Sample Name:	**mbs-mj-107-asy-adh-80/20-254-1.0ml**	*Injection Volume:*	**20.0**
Vial Number:	**820**	*Channel:*	**UV_VIS_1**
Sample Type:	**standard**	*Wavelength:*	**254**
Control Program:	**Mu Boshuai**	*Bandwidth:*	**n.a.**
Quantif. Method:	**Mu Boshuai**	*Dilution Factor:*	**1.0000**
Recording Time:	**2020-10-29 22:18**	*Sample Weight:*	**1.0000**
Run Time (min):	**10.94**	*Sample Amount:*	**1.0000**

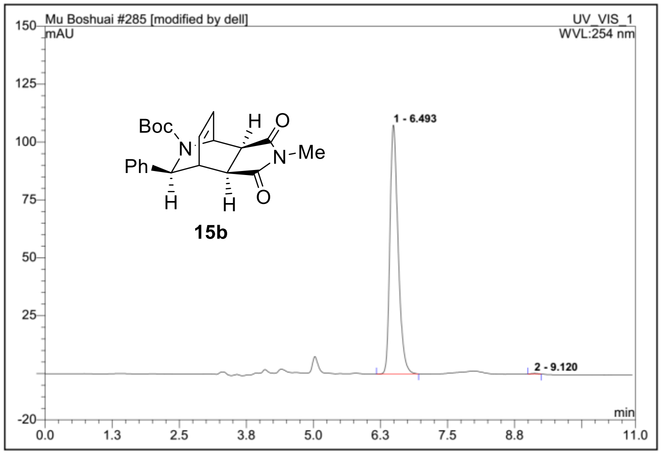

No.	Ret.Time min	Peak Name	Height mAU	Area mAU*min	Rel.Area %	Amount	Type
1	6.49	n.a.	107.504	19.044	99.78	n.a.	BMB*
2	9.12	n.a.	0.272	0.041	0.22	n.a.	BMB*
**Total:**			107.776	19.085	100.00	0.000	

284 mbs-mj-108-rac-adh-80/20-254-1.0ml

Sample Name:	**mbs-mj-108-rac-adh-80/20-254-1.0ml**	*Injection Volume:*	**20.0**
Vial Number:	**819**	*Channel:*	**UV_VIS_1**
Sample Type:	**standard**	*Wavelength:*	**254**
Control Program:	**Mu Boshuai**	*Bandwidth:*	**n.a.**
Quantif. Method:	**Mu Boshuai**	*Dilution Factor:*	**1.0000**
Recording Time:	**2020-10-29 21:16**	*Sample Weight:*	**1.0000**
Run Time (min):	**11.23**	*Sample Amount:*	**1.0000**

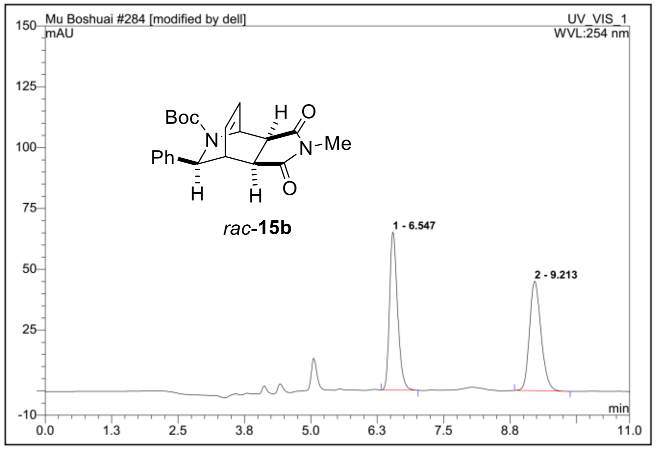

No.	Ret.Time min	Peak Name	Height mAU	Area mAU*min	Rel.Area %	Amount	Type
1	6.55	n.a.	64.849	11.391	50.02	n.a.	BMB*
2	9.21	n.a.	45.050	11.382	49.98	n.a.	BMB*
**Total:**			109.899	22.773	100.00	0.000	

Supplementary Figure 240. HPLC analysis for compound **15b**

Supplementary References

1. Wang, Q., Leutzsch, M., Gemmeren, M. van & List, B. Disulfonimide-catalyzed asymmetric synthesis of β^3-amino esters directly from *N*-Boc-amino sulfones. *J. Am. Chem. Soc.* **135**, 15334–15337 (2013).

2. Ruijter, E., Schültingkemper, H. & Wessjohann, L. A. Highly substituted tetrahydropyrones from hetero-Diels-Alder reactions of 2-alkenals with stereochemical inductionfrom chiral dienes. *J. Org. Chem.* **70**, 2820–2823 (2005).

3. Jiao, T., Wu, G., Zhang, Y., Shen, L., Lei, Y., Wang, C.-Y., Fahrenbach, A. C. & Li, H., Self-assembly in water with *N*-substituted imines. *Angew. Chem. Int. Ed.* **59**, 18350–18367 (2020).

4. Donohoe, T. J., Bower, J. F., Baker, D. B., Basutto, J. A., Chan, L. K. M. & Gallagher, P. Synthesis of 2,4,6-trisubstituted pyridines via an olefin cross-metathesis/Heck-cyclisation-elimination sequence. *Chem. Commun.* **47**, 10611–10613 (2011).

5. Denmark, S. E. & Beutner, G. L. Lewis base catalysis in organic synthesis. *Angew. Chem. Int. Ed.* **47**, 1560–1638 (2008).

6. Yang, J. W., Stadler, M. & List, B. Proline-catalyzed Mannich reaction of aldehydes with *N*-Boc-imines. *Angew. Chem. Int. Ed.* **46**, 609–611 (2007).

7. Yang, J. W., Chandler, C., Stadler, M., Kampen, D. & List, B. Proline-catalysed Mannich reactions of acetaldehyde. *Nature* **452**, 453–455 (2008).

8. Gianelli, C., Sambri, L., Carlone, A., Bartoli, G. & Melchiorre, P. Aminocatalytic enantioselective *anti*-Mannich reaction of aldehydes with in situ generated *N*-Cbz and *N*-Boc Imines. *Angew. Chem. Int. Ed.* **47**, 8700–8702 (2008).

9. Gnamm, C., Brçdner, K., Krauter, C. M., & Helmchen, G. A configurational switch based on Iridium-catalyzed allylic cyclization: application in asymmetric total syntheses of prosopis, dendrobate, and spruce alkaloids. *Chem. Eur. J.* **15**, 10514–10532 (2009).

10. Dai, J., Xiong, D., Yuan, T., Liu, J., Chen, T. & Shao, Z. Chiral primary amine catalysis for asymmetric Mannich reactions of aldehydes with ketimines: stereoselectivity and reactivity. *Angew. Chem. Int. Ed.* **56**, 12697–12701 (2017).

11. Padwa, A. & Kamigate, N. Photochemical transformations of small ring heterocyclic compounds. 82. Intramolecular dipolar cycloaddition reactions of unsaturated 2*H*-azirines. *J. Am. Chem. Soc*. **99**, 1871–1880 (1977).

12. Parthasarathy, K., Jeganmohan, M. & Cheng, C.-H. Rhodium-catalyzed one-pot synthesis of substituted pyridine derivatives from α,β-unsaturated ketoximes and alkynes. *Org. Lett.* **10**, 325–328 (2008).
